# 1,4-Pd Migration-Enabled Synthesis of Fused 4-Membered Rings

**DOI:** 10.1021/jacs.4c04701

**Published:** 2024-07-05

**Authors:** Maria Tsitopoulou, Antonin Clemenceau, Pierre Thesmar, Olivier Baudoin

**Affiliations:** Department of Chemistry, University of Basel, CH-4056 Basel, Switzerland

## Abstract

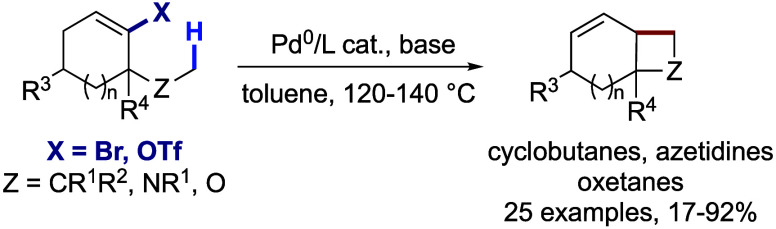

1,4-Palladium migration has been widely used for the functionalization of remote C–H bonds. However, this mechanism has been limited to aryl halide precursors. This work reports an unprecedented Pd^0^-catalyzed cyclobutanation protocol producing valuable fused cyclobutanes starting from cycloalkenyl (pseudo)halides. This reaction takes place via alkenyl-to-alkyl 1,4-Pd migration, followed by intramolecular Heck coupling. The method performs best with cyclohexenyl precursors, giving access to a variety of substituted bicyclo[4,2,0]octenes. Reactants containing an *N*-methyl or methoxy group give rise to fused azetidines or oxetanes, respectively, via the same mechanism. Kinetic and deuterium-labeling studies point to a rate-limiting C(sp^3^)–H activation step.

After its discovery by Heck in 1972,^1^ 1,4-palladium migration, also called 1,4-palladium shift, has been established as an original approach for the functionalization of remote C–H bonds, and has allowed access to complex polycyclic motifs.^2^ Since the seminal work of Dyker in 1992–94 showing the involvement of σ-alkylpalladium species generated from aryl iodides via C(sp^3^)–H activation-induced 1,4-Pd migration,^3^ these intermediates have been exploited in a variety of Pd^0^-catalyzed reactions.^4^ Recently, our group showed that such σ-alkylpalladium complexes are able to cleave a second C(sp^n^)–H bond (n = 2 or 3) to forge new C(sp^3^)–C(sp^n^) bonds, and furnish valuable carbo- and heterocyclic products.^5^ These compounds would be difficult to access via the direct reaction involving C(sp^3^)–H activation and reductive elimination.^6^ In particular, we reported that aryl halides containing geminal alkyl substituents undergo such a Pd^0^-catalyzed double C(sp^3^)–H/C(sp^3^)–H activation reaction to generate aryl cyclopropanes (Scheme 1a).^5b^ However, the developed methods have been so far limited to aryl precursors. To address this gap, we considered the use of cycloalkenyl electrophiles (Scheme 1b). These have been employed in direct C(sp^3^)–H alkenylation reactions,^7^ where they have demonstrated synthetic utility,^8^ but not in 1,4-Pd migration-based reactions. We hypothesized that cycloalkenyl bromide **1a** would undergo alkenyl-to-alkyl 1,4-Pd migration similar to its aryl analogue to generate σ-alkylpalladium intermediate **A**. In principle, **A** could undergo a second C(sp^3^)–H activation to produce cyclopropane **3a**, but this step generating a highly strained 4-membered palladacycle should occur with a high energy barrier.^5b^ In contrast, migratory insertion of the alkene in **A** into the Pd–C bond should be kinetically favored, and lead to fused cyclobutane **2a** upon β-hydride elimination.

**Scheme 1:**
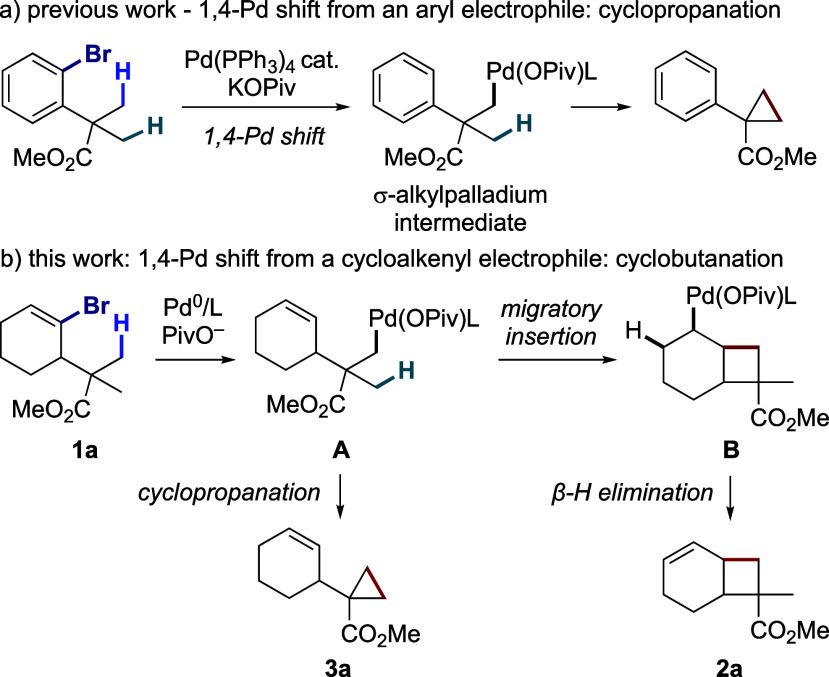
C(sp^3^)–H Activation-Mediated 1,4-Palladium Migration from Aryl and Cycloalkenyl Electrophiles

Fused cyclobutanes, azetidines and oxetanes, are present in numerous natural products and bioactive compounds (Figure 1).^9−11^ However, these four-membered carbo- and heterocycles are challenging to construct due to their inherent strain.^12^ In this context, C–H functionalization methods have recently emerged as step-economical alternatives for their synthesis.^13^ However, they have remained limited and challenging to develop, as most of them feature two high-energy steps, i. e. C–H activation and reductive elimination. Herein, we report an unprecedented, simple method to synthesize fused cyclobutanes, azetidines and oxetanes from cycloalkenyl (pseudo)halides through Pd^0^-catalyzed C(sp^3^)–H activation and 1,4-Pd migration.

**Figure 1:**
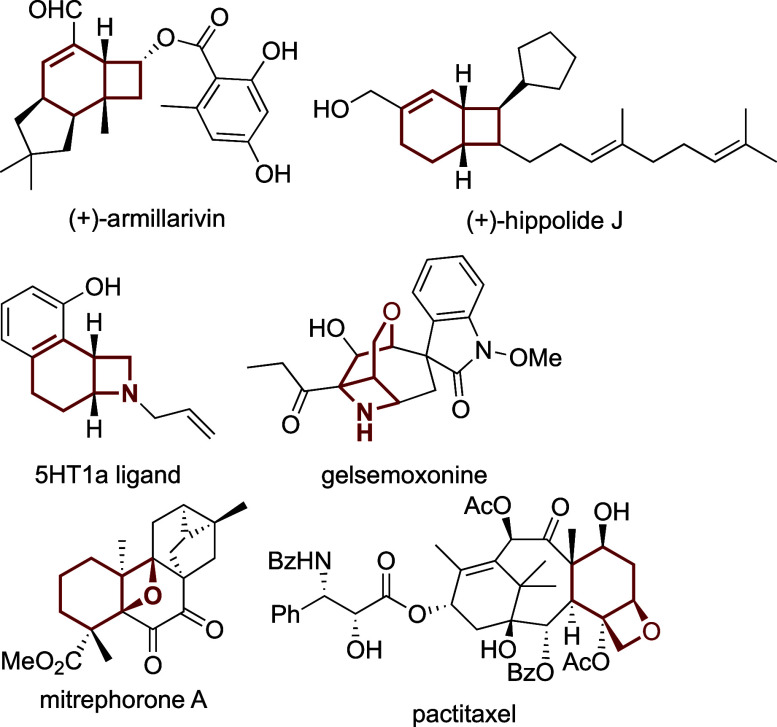
Fused cyclobutanes, azetidines, and oxetanes in natural products and bioactive molecules.

We started off by testing the conditions of the aforementioned cyclopropanation on prototypical substrate **1a** (Table 1).^5b^ To our delight, these conditions exclusively led to the fused cyclobutane product **2a** in 75% NMR yield (entry 1), hence validating our hypothesis that the putative σ-alkylpalladium intermediate **A** preferentially undergoes migratory insertion against a second C(sp^3^)–H activation (see Scheme 1b). To our knowledge, this is the first report of a direct alkenyl-to-alkyl 1,4-Pd migration. A slightly diminished yield was observed when potassium pivalate was replaced with cesium pivalate (entry 2). However, the yield drastically dropped when switching to potassium carbonate (entry 3), thereby confirming the positive effect of pivalate in 1,4-Pd migration.^5b^ Using potassium pivalate, we looked for a more suitable solvent (entries 4–8), and found that toluene furnished the highest yield (entry 6, see Table S1 for a full optimization).

**Table 1:**
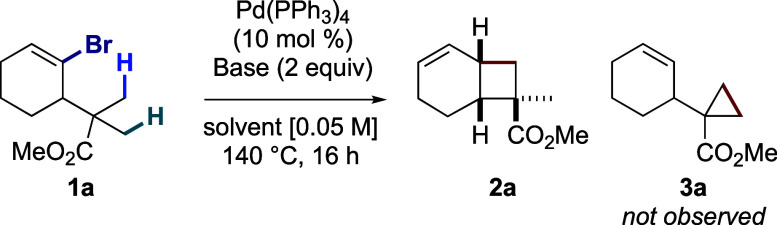
Optimization of the Cyclobutanation Reaction

Entry	Base (2 equiv)	Solvent	NMR Yield^a^
1	KOPiv	toluene/DMSO (20:1)	75%
2	CsOPiv	toluene/DMSO (20:1)	63%
3	K_2_CO_3_	toluene/DMSO (20:1)	39%
4	KOPiv	*m*-xylene	30%
5	KOPiv	mesitylene	68%
**6**	**KOPiv**	**toluene**	**90%**
7	KOPiv	DMF	15%
8	–	toluene	NR

a Determined using trichloroethylene as internal standard.

With the optimized conditions in hand, we investigated the scope of this cyclobutanation reaction (Scheme 2). Substrates bearing *gem*-dimethyl groups and an ester, nitrile, Weinreb amide, or protected primary alcohol on the quaternary carbon performed well (**2a**-**2e**), delivering the corresponding cylobutane products in moderate to high yield. As these *gem*-dimethyl groups are diastereotopic, products **2a**–**e** were obtained as diastereomeric mixtures with low to good diastereoselectivity. It seems that the latter correlates with the size of the functional group on the quaternary carbon, but firmer conclusions cannot be drawn as the d.r. could not be determined on the crude mixture. In addition, a monoterpene-like product (**2f**) was also accessed in 56% yield from the corresponding vinyl triflate precursor bearing a *t*-butyl group. Moreover, compound **2g** containing a *gem*-diester was obtained in high yield, including on a gram scale. Its X-ray diffraction analysis confirmed both the position of the double bond and the *cis* configuration of the ring junction. Next, the competition between primary (methyl) and secondary (ethyl/benzyl) C–H bonds was examined (**2h**, **2i**). In both cases a high selectivity for methyl C–H activation was observed, giving only traces of the ethyl/benzyl C–H activation product, consistent with our comparative kinetic study on these C–H bonds.^14^ Additional substituents on the six-membered ring were well tolerated (**2j**-**2n**), including protected alcohols and amines. Interestingly, substitution at the ring junction was also compatible with this method (**2o**). Moreover, this methodology can be also employed to access more complex polycyclic systems, as indicated by the generation of spiro bicyclo[4.2.0]octene **2p**.

**Scheme 2:**
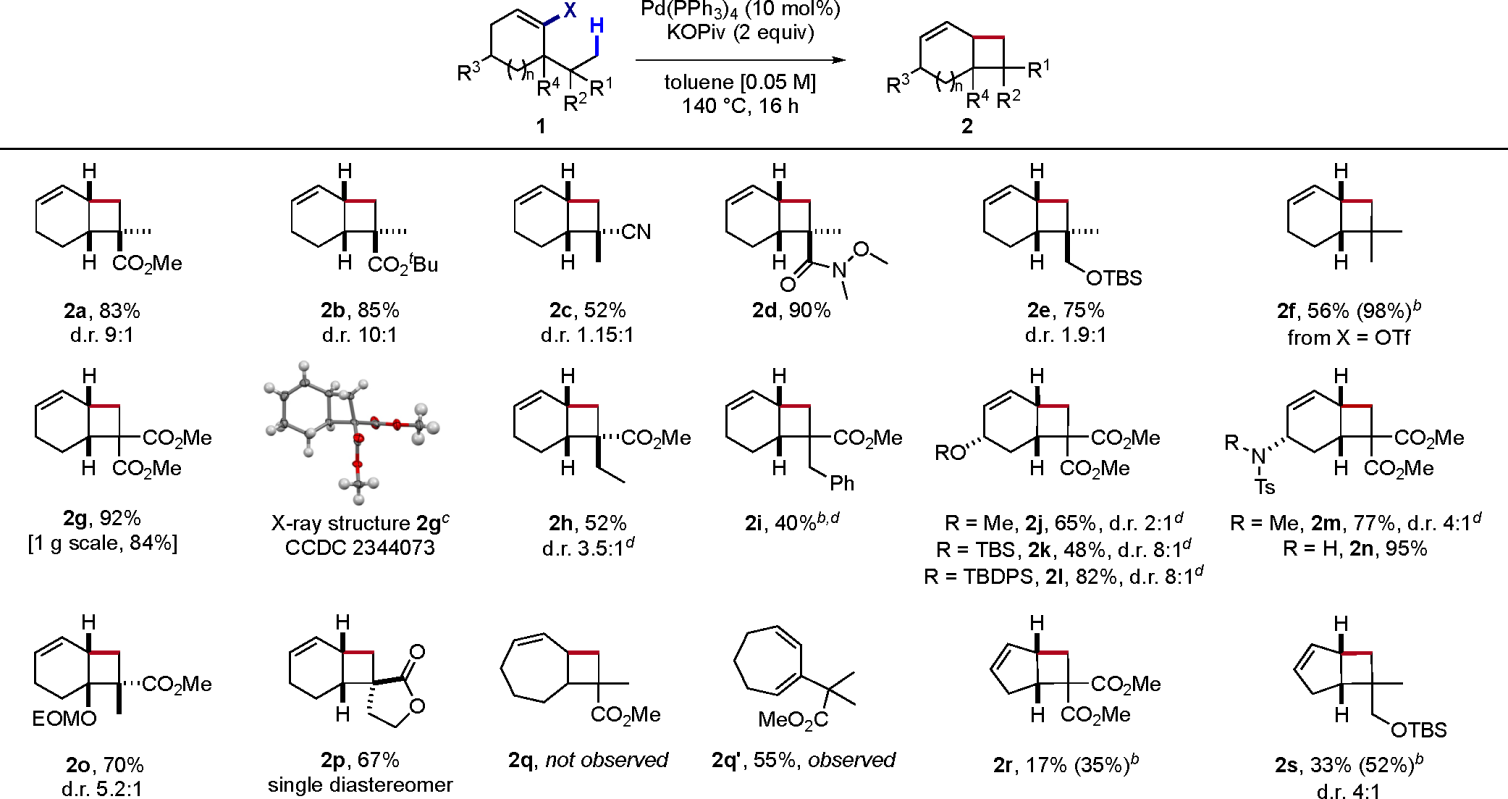
Scope of the Cyclobutanation Reaction The vinyl bromide precursor (X = Br) was used unless otherwise stated. Yields and diastereomeric ratios (d.r.) refer to isolated products. Relative configurations of the major diastereoisomers were determined by 2D NMR experiments. TBS = *tert*-butyldimethylsilyl; TBDPS = *tert*-butyldiphenylsilyl; Ts = tosyl; EOM = ethoxymethyl. NMR yield determined using trichloroethylene as internal standard. Thermal ellipsoids at 50% probability. Reaction conducted from an inseparable mixture of diastereoisomeric substrates.

In addition, the reactivity of other cycloalkenyl substrates was investigated. Cyclopentenyl bromides proved competent reactants, although they furnished lower yields than their cyclohexenyl analogues (**2r**, **2s**). Unfortunately, a cycloheptenyl substrate did not deliver the desired fused cyclobutane (**2q**), and formation of 1,3-diene **2q’** was observed instead. A similar transformation was reported by Frantz and co-workers from enol triflates.^15^ This result indicates that, after the initial oxidative addition of the C–Br bond to Pd^0^, β-H elimination is favored over C(sp^3^)–H activation for 7-membered ring substrates.

We recently reported that 1,4-Pd migration on an *N*-alkyl group is also feasible, and leads to interesting azacycles including indolines,^5a^ isoindolines and β-lactams.^5c^ Fused azetidines, due to their inherent ring strain and lack of practical syntheses, represent a relatively underexplored class of azacycles and an attractive target for synthetic chemistry.^12d^ By analogy to cyclobutanes, we considered accessing fused azetidines from cycloalkenyl electrophiles containing an *N*-methyl group (Scheme 3, top). In light of our previous work on the synthesis of benzoxazines via benzazetidine intermediates, we designed precursor **4a** bearing a 1-adamantylamide to 1. increase the steric bulk on the nitrogen atom and 2. prevent competitive reaction at this N-protecting group (Scheme 3).^16^ Our initial attempt with this substrate employed the optimal conditions for the cyclobutanation reaction, which unfortunately led to only 12% NMR yield. To our delight, using a combination of adamantoic acid (30 mol %) and K_2_CO_3_ (1.5 equiv) instead of KOPiv and PCy_3_ as ligand instead of PPh_3_, the desired azetidine **5a** was formed in 66% NMR yield (see Table S2 for a full optimization). Starting from a scalemic precursor (e.r. 73:27), **5a** was obtained in 65% yield without any racemization. Switching to a trifluoroacetyl protecting group drastically reduced the yield (**5b**), hence highlighting the importance of a bulky and stable protecting group at the nitrogen atom. Gratifyingly, ring substitution positively affected the efficiency of this transformation, leading to azetidines **5c** and **5d** in higher yields (88% and 74%, respectively). X-ray diffraction analysis of **5d** confirmed the azetidine formation.

**Scheme 3:**
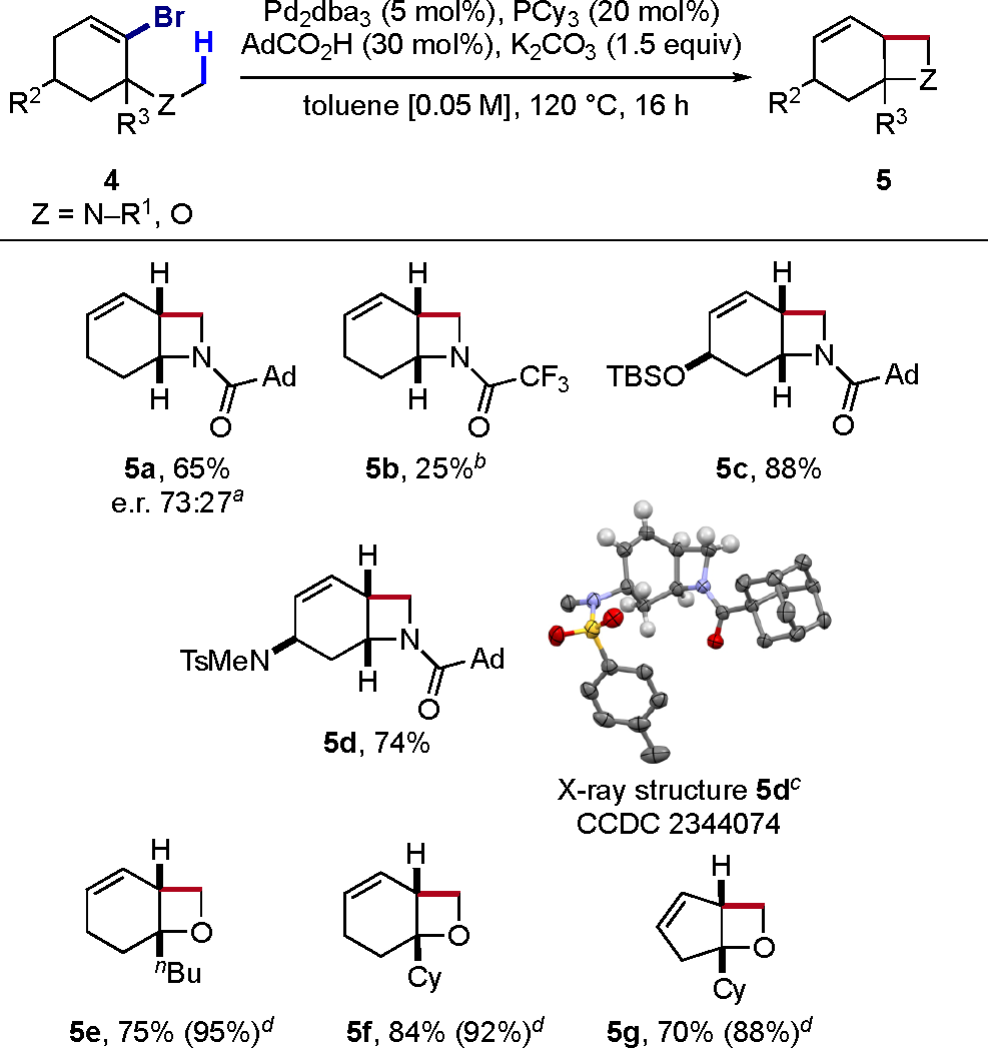
Synthesis of Azetidines and Oxetanes From a scalemic substrate (e.r. 73:27). Using 10 mol % of [Pd(PCy_3_)_2_]. Thermal ellipsoids at 50% probability. Most H atoms omitted for clarity. NMR yield determined using trichloroethylene as internal standard.

Oxetane rings are found in several bioactive molecules, due to their unique pharmacological properties.^12c^ Oxetanes have also been proposed as bioisosteres of *gem*-dimethyl and carbonyl groups.^17^ However, the high tortional strain of the oxetane ring makes it susceptible to opening with nucleophiles, ring expansions and rearrangements, and synthetic methods to construct this heterocycle are limited. As 1,4-Pd migration on a methoxy group was previously demonstrated from aryl halides,^3,5a^ we hypothesized that similar conditions to those leading to cyclobutanes and azetidines could be applied to form oxetanes. Indeed, the 6-substituted oxabicyclo[4.2.0]octenes **5e** and **5f** were obtained in 75% and 84% yield, respectively, under the above azetidination conditions (Scheme 3, bottom). Interestingly, a five-membered ring precursor performed better in this case, delivering the oxabicyclo[3.2.0]heptene **5g** in 70% yield. The high volatility of the products might contribute to the decreased isolated yields, as the NMR yields were consistently around 90% in all cases. Substitution at the ring fusion was mandatory, as complex mixtures were otherwise obtained (see the SI for unsuccessful substrates).

To provide mechanistic insights and study possible differences with previous 1,4-Pd migration-based reactions,^5^ a series of kinetic experiments were performed. First, the kinetic orders for the reaction of **1g** forming **2g** were determined using variable time normalization analysis (VTNA), a visual method developed by Burés and co-workers (Figure 2).^18,14^ This analysis indicated order 0 in **1g**, after a duplicate experiment, suggesting a facile oxidative addition. In addition, zero-order in KOPiv was observed. At the employed concentrations, pivalate is poorly soluble in toluene and the observed zero order likely reflects saturation kinetics, consistent with previous studies.^14,19,20^ This analysis also revealed a first-order rate dependence in Pd(PPh_3_)_4_, consistent with catalysis by a mononuclear Pd complex.

**Figure 2:**
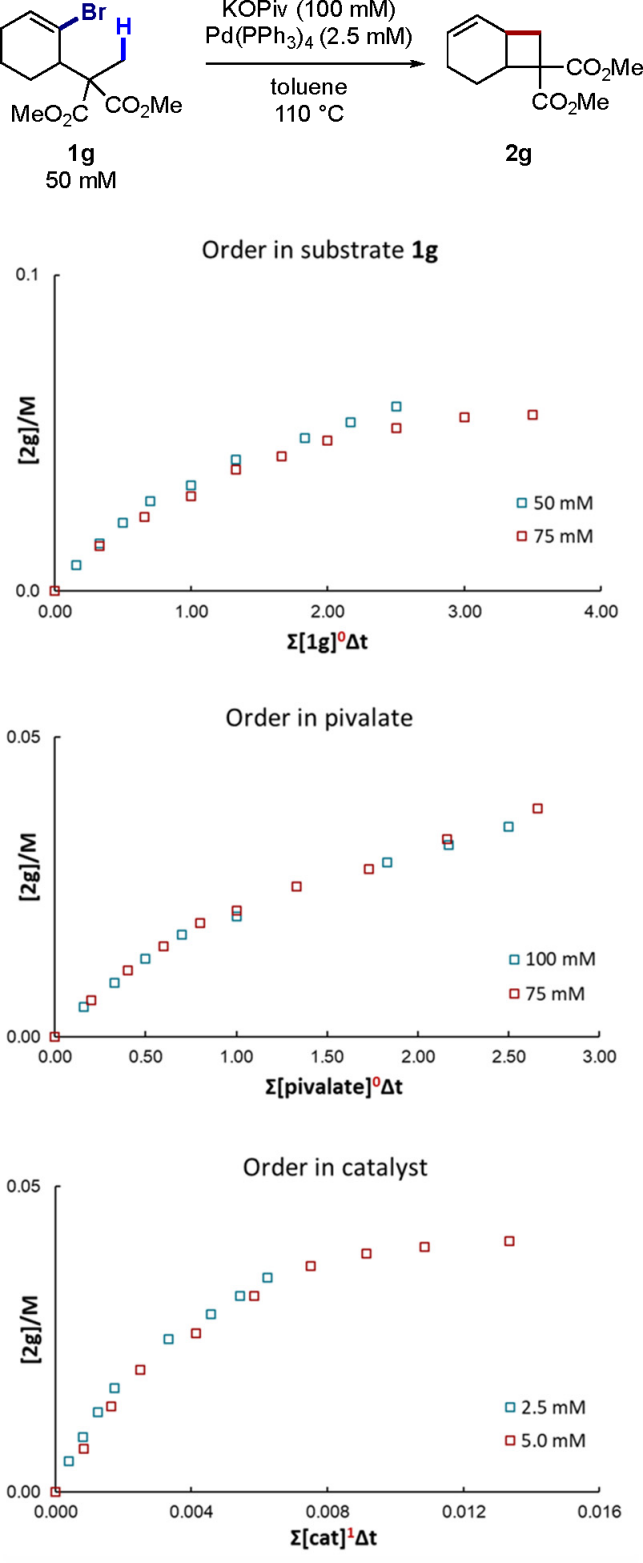
Determination of kinetic orders using VTNA.

Furthermore, parallel experiments were performed with substrates **1g** and *d*_**3**_**-1g** to determine the kinetic isotope effect (Scheme 4a). A primary KIE was calculated (*k*_H_/*k*_D_ 3.1), indicating that the rate-limiting step involves C–H bond cleavage or formation. Moreover, the cyclobutane-fused product arising from the standard reaction of *d*_**3**_**-1g** showed significant H/D scrambling (70% H) at the ring junction, but no H/D scrambling at the methylene position, which indicates that the C–H activation step is irreversible and rate-limiting (Scheme 4b).

**Scheme 4:**
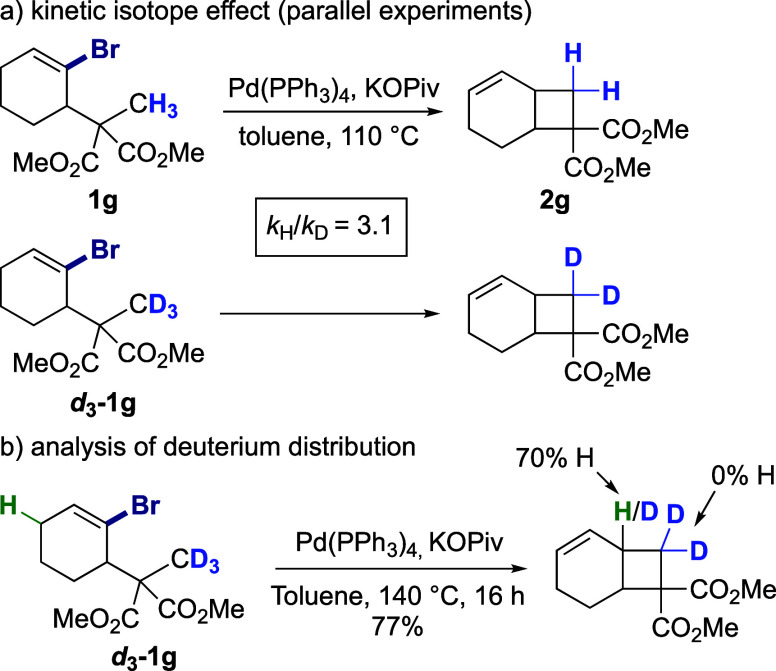
Deuterium Labeling and KIE

Taking the above data into consideration, a catalytic cycle is proposed (Scheme 5). Facile oxidative addition of the cycloalkenyl bromide to the putative Pd^0^L_2_ active catalyst furnishes complex **I**, which then undergoes fast ligand exchange to form σ-alkenylpalladium pivalate **II**,^5b^ which is presumably the catalyst resting state, based on the above kinetic data. In complex **II** pivalate likely coordinates in a κ^2^ mode, which according to previous work^20,14^ promotes the subsequent C–H activation step. Palladacycle **III** is then formed via base-mediated C(sp^3^)–H activation through the concerted metalation-deprotonation (CMD)/ambiphilic metal–ligand activation (AMLA) mechanism.^21^ The reductive elimination from **III**, producing a highly strained cyclobutane-fused cyclohexene **VI**, is energetically disfavored. Instead, the kinetically favored protonation of **III** by pivalic acid at the C(sp^2^) carbon^5b^ selectively forms the σ-alkylpalladium species **IV**, thereby accomplishing the two-step 1,4-Pd migration. The performed deuterium-labeling studies (Scheme 4) indicate that the C–H activation step forming **III** is irreversible (no H incorporation on the cyclobutane methylene) and rate-limiting (primary KIE), in accordance with previous kinetic studies on C(sp^3^)–H activation.^14^ Then, *syn*-stereospecific migratory insertion of the olefin in **IV** into the Pd–C bond leads to complex **V**, which undergoes *syn*-β-H elimination to generate the fused cyclobutane product, followed by reductive elimination to regenerate the active catalyst and produce PivOH. The latter is the probable source of H incorporation at the ring junction during the deuterium labeling experiment (see Scheme 4b).

**Scheme 5:**
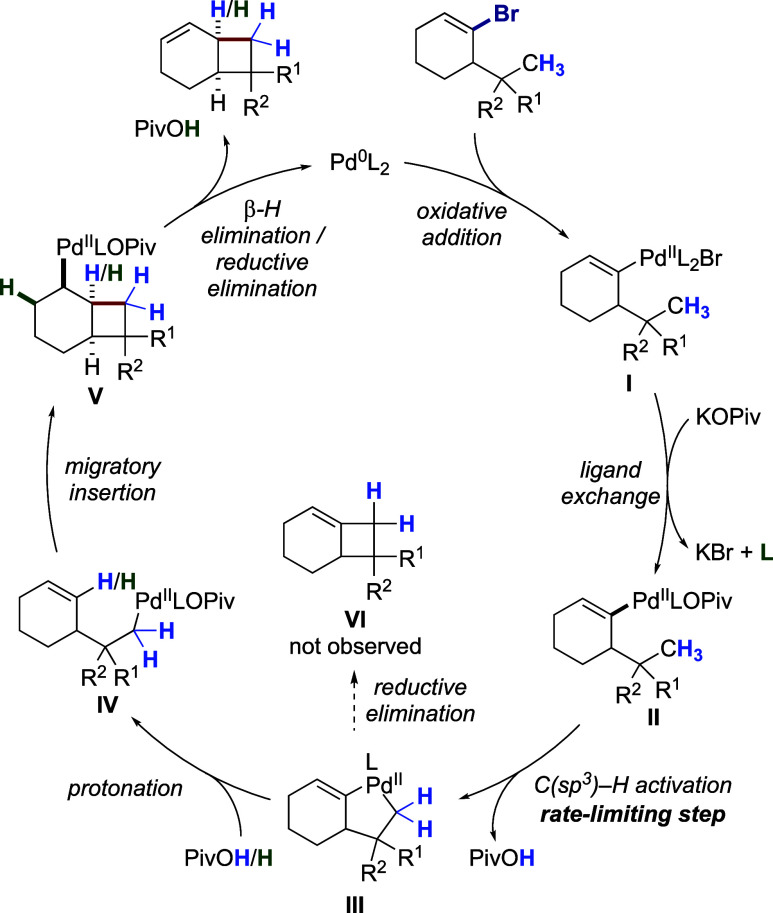
Proposed Catalytic Cycle (L = PPh_3_)

In summary, we reported a new straightforward method to synthesize fused cyclobutanes, azetidines and oxetanes from cycloalkenyl electrophiles via Pd^0^-catalyzed C(sp^3^)–H activation and alkenyl-to-alkyl 1,4-Pd migration. A kinetic study was performed and established the C–H activation as the rate-limiting step. This work sets a precedent for the use of alkenyl precursors in 1,4-Pd migration and further applications toward challenging motifs are currently under investigation.

## Supporting Information Available

Full optimization tables, crystallographic data, experimental procedures, and spectral data (PDF)
