# Supplementary material for: 1,4-Pd Migration-Enabled Synthesis of Fused 4-Membered Rings
Source: J Am Chem Soc. 2024 Jul 5;146(28):18811–6. doi: 10.1021/jacs.4c04701 (PMC11258686; doi:10.1021/jacs.4c04701)
Supplement: Supplementary file 1 — ja4c04701_si_001.pdf [file ja4c04701_si_001.pdf]

Supporting Information

1,4-Pd Migration-Enabled Synthesis of Fused 4-Membered Rings

Maria Tsitopoulou, Antonin Clemenceau, Pierre Thesmar, and Olivier Baudoin*

Department of Chemistry, University of Basel, CH-4056 Basel, Switzerland.

*olivier.baudoin@unibas.ch

Table of contents

General ... 3
Optimization of catalytic reaction ... 4
Cyclobutanation ... 4
Azeditination ... 5
General Procedures ... 6
Cyclopropanation (General Procedure A) ... 6
Alkylation (General Procedure B) ... 6
Alkylation (General Procedure C) ... 6
Alkylation (General Procedure D) ... 7
Synthesis of C–H activation substrates ... 8
C(sp^3)–H Activation ... 45
Cyclobutanation (General Procedure E) ... 45
Azetidination (General Procedure F) ... 45
Oxetanation (General Procedure G) ... 46
C–H activation products – Cyclobutanation ... 47
C–H activation products – Azetidination ... 61
C–H activation products – Oxetanation ... 65
Unsuccessful substrates ... 69
Kinetic Studies ... 71
Determination of Orders ... 71
Determination of Kinetic Isotope Effect (KIE) ... 75
Crystallographic data ... 77
References ... 81
^{1}H, ^{13}C and ^{19}F NMR spectra ... 82
C–H activation substrates ... 82
C–H activation products ... 140

General

Experimental Techniques

All reactions involving sensitive materials were performed in oven-dried glassware and under argon using Schlenk techniques or in an argon-filled glovebox unless stated otherwise. The progress of the reactions was monitored by thin layer chromatography (TLC) and TLC-MS analysis. TLCs were performed using Merck silica gel 60 F254 plates (0.25 mm) and visualized using potassium permanganate stain solution. Column chromatography was performed using Silicycle SiliaFlash P60 (40-63 µm) with the correspondent solvent system. Filtration under celite® was performed using Supelco celite® 545 (particle size 0.02-0.1 mm). C–H activation reactions were performed using Pyrex disposable tubes (16 x 100 mm) that were dried overnight in the oven (120 °C). Diastereomeric ratios are reported on isolated products and are determined by ^{1}H NMR.

Solvents and Chemicals

Anhydrous solvents were purchased from Thermo Fisher Scientific over molecular sieves or were obtained from a solvent purification system using activated alumina columns and were further degassed with argon for one hour and stored in an argon-filled glovebox. Chemical reagents were purchased from Acros Organics, Alfa Aesar, Apollo Scientific, Combi-Blocks, Fluorochem and Merck (Sigma-Aldrich) and used without further purification.

Instrumentation

TLC-MS analyses was performed in an Advion Plate Express TM TLC Plate Reader in combination with an Advion Expression LCMS (solvent: MeOH, APCI-Source). Melting points were measured on a Büchi M-565 instrument. IR spectra were recorded on an ATR Varian Scimitar 800 and are reported in reciprocal centimeters (cm^{-1}). Nuclear Magnetic Resonance (NMR) spectra were recorded on a Bruker Avance 400 (400 MHz) and a Bruker Avance 500 (500 MHz) in $CDCl_3$ (reference signals at δ 7.26 ppm for ^{1}H and δ 77.16 ppm for ^{13}C). Chemical shifts are reported in ppm and the signals are described as follows: s = singlet, d = doublet, t = triplet, 't' = apparent triplet, q = quartet, p = pentet, m = multiplet, br = broad. Coupling constants (*J*) are reported in Hz. ^{19}F NMR spectra were recorded with ^{1}H decoupling. High Resolution Mass Spectra (HRMS) were recorded by Dr. M. Pfeffer (Department of Chemistry, University of Basel) on a Bruker maXis 4G QTOF ESI mass spectrometer. For non-ionizable compounds the GCMS spectra were recorded on a Shimadzu GCMS system (model: GCMS-QP2010 SE) with an Rtx®-5MS column (30 m x 0.25 mm ID) and H_2 as carrier gas. Preparative HPLC was performed with a preparative Shimadzu HPLC system with a Gemini 10 µm NX-C18, LC Column 150 × 30 mm and the correspondent solvent system. X-ray analyses were performed by Dr. A. Prescimone (Department of Chemistry, University of Basel). Optical rotations were measured on a Perkin Elmer 341 Polarimeter with a 0.7 mL sample cell (cell length 100 mm) with NaD-Line (λ = 589 nm) at 20 °C with c = 1.00 (g/100 ml). The enantiomeric excesses were determined on a Shimadzu GC-FID system (model: GC-2010 Plus) with a HYDRODEX β-6TBDM column (25 m x 0.25 mm ID) and H_2 as carrier gas.

Optimization of catalytic reaction

Cyclobutanation

Table S1. Optimization of reaction conditions for cyclobutanation

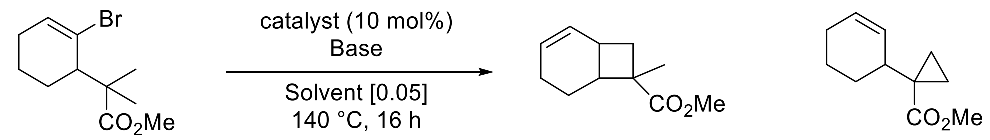

1a **2a** **3a not observed**

No.	Base (2 eq)	Solvent	Catalyst	NMR yield
1	KOPiv	*m*-xylene	$Pd(PPh_3)_4$	30%
2	K_2CO_3	*m*-xylene		12%
3	KOPiv	*m*-xylene	$Pd(PCy_3)_2$	25%
4	KOPiv	Toluene/DMSO (20:1)		75%
5	CsOPiv	Toluene/DMSO (20:1)		63%
6	K_2CO_3	Toluene/DMSO (20:1)		75%
7	PivOH (0.1 eq)/ K_2CO_3	Toluene/DMSO (20:1)		75%
8	KOPiv	DMF		15%
9	KOPiv	Mesitylene		68%
**10**	**KOPiv**	**Toluene**	$Pd(PPh_3)_4$	**90%**
1	CsOPiv	Toluene		60%
12	KOPiv	Toluene		78%[a]
13	KOPiv	Toluene		80%[b]
14	KOPiv	Toluene		60%[c]
15	KOPiv	Toluene		70%[d]
16	-	Toluene		NR
17	KOPiv	Toluene	-	NR

[a]at 120 °C. [b]for 5 h. [c]for 24 h. [d]with 1 eq of KOPiv.

Azeditination

Table S2. Optimization of reaction conditions for azetidination.

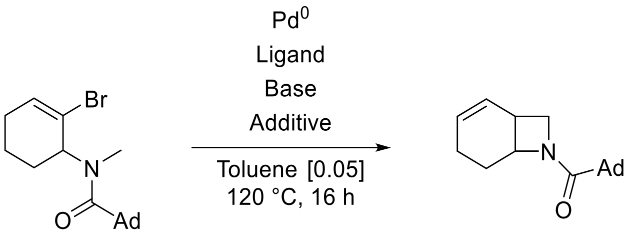

No.	Pd^0 (10 mol%)	Ligand (20 mol%)	Base (1.5 eq)	Additive (30 mol%)	NMR yield
1	Pd(PPh_3)$_4$	-	KOPiv (2 eq)	-	18%
2	Pd(PCy_3)$_2$	-	Rb_2CO_3	$AdCO_2H$	53%
3	[Pd(π-cinnamyl)Cl]$_2$	IBioxMe$_4$	Cs_2CO_3	-	12%
4	[Pd(π-allyl)Cl]$_2$	IMesCl	Cs_2CO_3	CsOPiv	8% (50% SM)
5	Pd(PCy_3)$_2$	-	KOPiv (2 eq)	-	33%
6	Pd(PPh_3)$_4$	-	Rb_2CO_3	$AdCO_2H$	12%
7	Pd(PCy_3)$_2$	-	Rb_2CO_3	PivOH	50%
8	Pd(PCy_3)$_2$	-	Cs_2CO_3	$AdCO_2H$	44%
9	Pd(PCy_3)$_2$	-	K_2CO_3	$AdCO_2H$	56%
**10**	**Pd_2dba_3**	**PCy_3**	**K_2CO_3**	**$AdCO_2H$**	**66%**
11	Pd(P*t*Bu$_3$)$_2$	-	K_2CO_3	$AdCO_2H$	13% (26% SM)
12	Pd_2dba_3	P*t*Bu$_3$-HBF$_4$	K_2CO_3 (1.7 eq)	$AdCO_2H$	11% (28% SM)
13	Pd_2dba_3	P(*o*-tolyl)$_3$	K_2CO_3	$AdCO_2H$	5% (68% SM)
14	Pd_2dba_3	P(*o*-furyl)$_3$	K_2CO_3	$AdCO_2H$	6% (44% SM)
15	Pd_2dba_3	CataCXium	K_2CO_3	$AdCO_2H$	33%
16	Pd_2dba_3	PCy_3	K_2CO_3	$AdCO_2H$	40% (34% SM)[a]
17	Pd_2dba_3	PCy_3	K_2CO_3	$AdCO_2H$	16% (66% SM)[b]
18	Pd_2dba_3	PCy_3	K_2CO_3	$AdCO_2H$	26% (51% SM)[c]
19	Pd_2dba_3	PCy_3	K_2CO_3	$AdCO_2H$	53% (19% SM)[d]

[a] at 110 °C. [b] at 100 °C. [c] for 5 h. [d] for 10 h.

General Procedures

Cyclopropanation (General Procedure A)

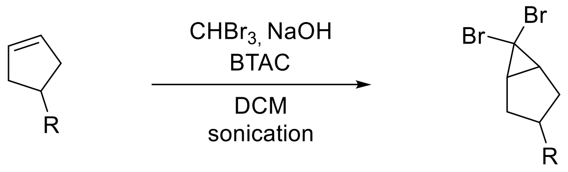

S1, S6, S11, S14

To a flask charged with powdered sodium hydroxide (5.7 eq) in DCM (1.8 M) were added benzyltriethylammonium chloride (0.02 eq), the corresponding cyclopentene (1 eq) and bromoform (1.9 eq) and the flask was fitted to a reflux condenser and subjected to sonication for 1h. The cooled reaction mixture was filtered through a short pad of celite® and then washed with DCM. The combined filtrates were concentrated under reduced pressure. The crude was purified by column chromatography on silica gel. Concentration of the appropriate fractions afforded the desired product.

Alkylation (General Procedure B)

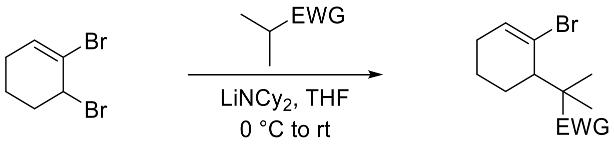

S2 **1a-c**

To a dry flask charged with dicyclohexylamine (1.2 eq) under inert atmosphere n-BuLi (2.5 M in THF, 1.2 eq) was added at 0 °C. After 10 min a solution of the corresponding nucleophile (1.2 eq, 0.25 M in THF) was added dropwise at 0 °C. After 30 min a solution of the corresponding 1,6-dibromocyclohex-1-ene (1 eq, 0.5 M in THF) was added dropwise and the reaction was left to reach room temperature. After completion the reaction mixture was quenched with sat. NH_4Cl solution and extracted x3 with EtOAc. The combined organic layers were then washed x1 with brine, dried over anhydrous Na_2SO_4 and evaporated under reduced pressure. The crude was purified by column chromatography on silica gel. Concentration of the appropriate fractions afforded the desired product.

Alkylation (General Procedure C)

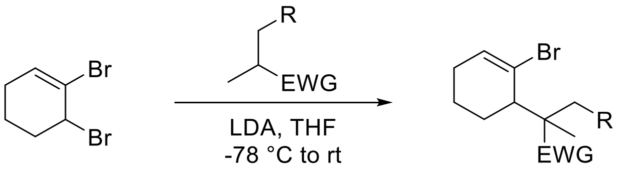

S2 **1h,1i**

To a dry flask charged with a solution of diisopropylamine (1.2 eq, 1 M in THF) under inert atmosphere n-BuLi (1.6 M in Hexanes, 1.2 eq) was added at -78 °C. After 10 min a solution of the corresponding nucleophile (1.2 eq, 0.25 M in THF) was added dropwise at -78 °C. After 30 min a solution of 1,6-dibromocyclohex-1-ene (1 eq, 0.5 M in THF) was added dropwise and the reaction was left to reach room temperature. After completion the reaction mixture was quenched with sat. NH_4Cl solution and extracted x3 with EtOAc. The combined organic layers were then washed x1 with brine, dried over

anhydrous Na_2SO_4 and evaporated under reduced pressure. The crude was purified by column chromatography on silica gel. Concentration of the appropriate fractions afforded the desired product.

Alkylation (General Procedure D)

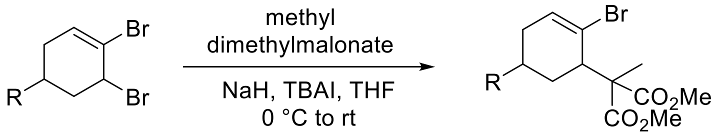

S2 **1g, 1j-m, S15**

To a dry flask NaH (1.2 eq, 60% in mineral oil), TBAI (0.4 eq) and THF (0.625 M) were added and the flask was placed at -10 °C. After 10 min a solution of methyl dimethylmalonate (1.2 eq, 0.2 M in THF) was added dropwise and the flask was left for 30 min at 0°C. Then a solution of the corresponding alkylating agent (1 eq, 0.5 M in THF) was added and the flask was left to reach room temperature. After completion the reaction mixture was quenched with distilled water and extracted x3 with EtOAc. The combined organic layers were then washed x2 with NaOH 1 M and x1 with brine, dried over anhydrous Na_2SO_4 and evaporated under reduced pressure. The crude was purified by column chromatography on silica gel. Concentration of the appropriate fractions afforded the desired product.

Synthesis of C–H activation substrates

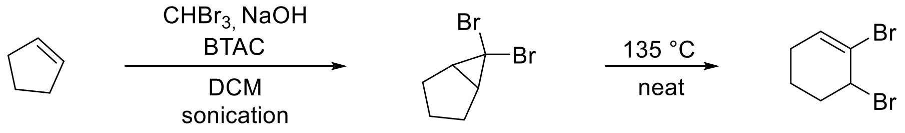

S1 **S2**

Following general procedure A: using sodium hydroxide (16.4 g, 411 mmol, 5.7 eq) in 40 ml of DCM, benzyltriethylammonium chloride (328 mg, 1.44 mmol, 0.02 eq), cyclopentene (4.91 g, 72.1 mmol, 1 eq) and bromoform (12 ml, 137 mmol, 1.9 eq). The flask was fitted to a reflux condenser and subjected to sonication for 1 h. The cooled reaction mixture was filtered through a short pad of celite and then washed with 100 ml DCM. The combined filtrates were concentrated under reduced pressure to afford a brown oil. The crude was purified by column chromatography on silica gel (100% petroleum ether). Concentration of the appropriate fractions afforded **S1** as a colorless oil. A flask containing 6,6-dibromobicyclo[3.1.0]hexane (**S1**, 16.8 g, 70.1 mmol) under inert atmosphere was placed neat at 135 °C. After 1 h the cooled crude mixture was subjected to column chromatography on silica (100% petroleum ether). Concentration of the appropriate fractions afforded **S2** as a clear, light yellow oil. ^{1}H and ^{13}C NMR data match those reported in the literature.[1]

Yield: 16 g, 95% over two steps.

Rf: 0.5 (pure petroleum ether).

Aspect: light yellow oil.

^{1}H NMR (250 MHz, $CDCl_3$): δ (ppm) 6.21 – 6.17 (m, 1H), 4.80 – 4.77 (m, 1H), 2.36 – 2.20 (ddddt, 3H), 2.17 – 2.08 (m, 1H), 2.07 – 1.95 (dddt, 1H), 1.80 – 1.71 (m, 1H).

^{13}C NMR (126 MHz, $CDCl_3$): δ (ppm) 133.9, 122.5, 54.0, 33.8, 27.5, 16.5.

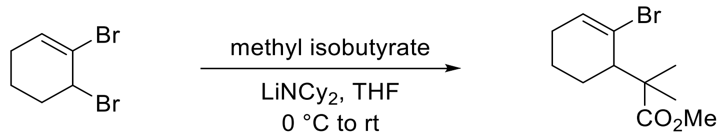

S2 **1a**

Following general procedure B: using dicyclohexylamine (2.79 g, 15.4 mmol, 1.2 eq), n-BuLi (6.14 ml, 2.5 M in THF, 1.2 eq), methyl isobutyrate (1.57 g, 15.4 mmol, 1.2 eq, 0.25 M in THF) and 1,6-dibromocyclohex-1-ene (**S2**, 3.06 g, 12.8 mmol, 0.5 M in THF). After 16 h the reaction mixture was quenched with 10 ml of sat. NH_4Cl solution and extracted x3 with EtOAc. The combined organic layers were then washed x1 with brine, dried over anhydrous Na_2SO_4 and evaporated under reduced pressure to afford a yellow oil. The crude was purified by column chromatography on silica gel (Cyclohexane/EtOAc 100:0 to 98:2). Concentration of the appropriate fractions afforded **1a** as a light yellow oil.

Yield: 3 g, 89.7%.

Rf: 0.34 (80:20 Cyclohexane/EtOAc)

Aspect: light yellow oil.

^{1}H NMR (500 MHz, CDCl$_3$): δ (ppm) 6.24 – 6.18 (m, 1H), 3.69 (s, 3H), 3.26 – 3.21 (m, 1H), 2.05 – 2.00 (m, 2H), 1.95 – 1.89 (m, 1H), 1.79 (dddd, J = 10.0, 6.8, 4.6, 3.1 Hz, 1H), 1.59 – 1.53 (m, 1H), 1.53 – 1.46 (m, 1H), 1.20 (s, 3H), 1.16 (s, 3H).

^{13}C NMR (126 MHz, CDCl$_3$): δ (ppm) 179.3, 133.8, 125.3, 52.2, 47.3, 45.5, 28.0, 26.7, 26.0, 21.4, 19.0.

IR (ATR): ν_{max} 2981, 2945, 1734, 1248, 650 cm^{-1}.

HRMS: (ESI) m/z [M + Na]$^+$ calculated for $C_{11}H_{17}BrNaO_2$: 283.0304; found: 283.0295.

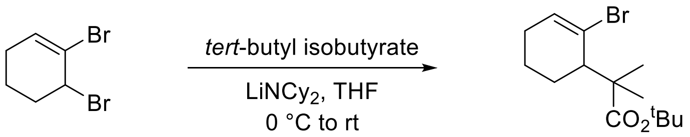

S2 **1b**

Following general procedure B: using dicyclohexylamine (454 g, 2.51 mmol, 1.5 eq), n-BuLi (1.57 ml, 1.6 M in THF, 1.5 eq), *tert*-butyl isobutyrate (361 mg, 2.51 mmol, 1.5 eq, 0.25 M in THF) and 1,6-dibromocyclohex-1-ene (**S2**, 400 mg, 1.37 mmol, 0.5 M in THF). After 16 h the reaction mixture was quenched with 10 ml of sat. NH_4Cl solution and extracted x3 with EtOAc. The combined organic layers were then washed x1 with brine, dried over anhydrous Na_2SO_4 and evaporated under reduced pressure to afford a yellow oil. The crude was purified by column chromatography on silica gel (Cyclohexane/EtOAc 100:0 to 99:1). Concentration of the appropriate fractions afforded **1b** as a light yellow oil

Yield: 280 mg, 55.3%.

Rf: 0.4 (80:20 Cyclohexane/EtOAc).

Aspect: light yellow oil.

^{1}H NMR (400 MHz, CDCl$_3$): δ (ppm) 6.20 ('td', J = 4.3, 1.8 Hz, 1H), 3.21 –3.12 (m, 1H), 2.05 – 1.98 (m, 2H), 1.95 – 1.86 (m, 1H), 1.83 – 1.74 (m, 1H), 1.62 – 1.48 (m, 2H), 1.46 (s, 9H), 1.16 (s, 3H), 1.11 (s, 3H).

^{13}C NMR (126 MHz, CDCl$_3$): δ (ppm) 177.6, 133.5, 125.8, 80.1, 46.9, 46.1, 28.0, 27.1, 26.0, 21.3, 19.5.

IR (ATR): ν_{max} 2978, 2936, 1725, 1252, 1150, 1124 cm^{-1}.

HRMS: (ESI) m/z [M + Na]$^+$ calculated $C_{14}H_{23}BrNaO_2$: 325.0774; found: 325.0773.

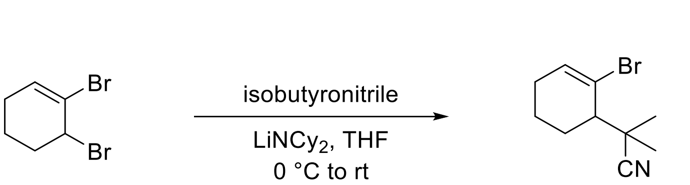

S2 **1c**

Following general procedure B: using dicyclohexylamine (566 g, 3.12 mmol, 1.5 eq), n-BuLi (1.95 ml, 1.6 M in THF, 1.5 eq), isobutyronitrile (172 mg, 2.50 mmol, 1.5 eq, 0.25 M in THF) and 1,6-dibromoclohex-1-ene (500 mg, 2.08 mmol, 0.5 M in THF). After 16 h the reaction mixture was quenched with 10 ml of sat. NH_4Cl solution and extracted x3 with EtOAc. The combined organic layers

were then washed x1 with brine, dried over anhydrous Na_2SO_4 and evaporated under reduced pressure to afford a yellow oil. The crude was purified by column chromatography on silica gel (Cyclohexane/EtOAc 98:2). Concentration of the appropriate fractions afforded **1c** as a colorless oil.

Yield: 314 mg, 66%.

Rf: 0.31 (90:10 Cyclohexane/EtOAc).

Aspect: colorless oil.

^{1}H NMR (400 MHz, $CDCl_3$): δ (ppm) 6.34 ('t', *J* = 4.2 Hz, 1H), 2.59 (td, *J* = 5.3, 2.1 Hz, 1H), 2.14 (pd, *J* = 4.2, 1.5 Hz, 2H), 1.94 – 1.87 (m, 2H), 1.84 (ddd, *J* = 13.2, 6.4, 3.3 Hz, 1H), 1.67 – 1.59 (m, 1H), 1.56 (s, 3H), 1.50 (s, 3H).

^{13}C NMR (101 MHz, $CDCl_3$): δ (ppm) 135.3, 125.4, 120.5, 48.6, 36.1, 27.9, 27.5, 27.2, 26.1, 18.3.

IR (ATR): ν_{max} 2978, 2942, 2364, 2339, 1461, 658 cm^{-1}.

HRMS: (ESI) m/z $[M + Ag]^+$ calculated for $C_{10}H_{14}AgBrN$: 333.9355; found: 333.9355.

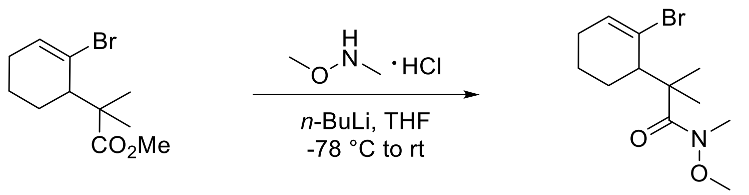

1a **1d**

To a dry flask charged with a solution of N,O-dimethylhydroxylamine hydrochloride (95 mg, 0.97 mmol, 2.55 eq, 1 M in THF) under inert atmosphere n-BuLi (0.78 ml, 1.95 mmol, 5.1 eq, 2.5 M in Hexanes) was added dropwise at -78 °C. The resulting solution was warmed to room temperature, stirred for 15 min, and cooled back to -78°C. Then a solution of methyl 2-(2-bromocyclohex-2-en-1-yl)-2-methylpropanoate (**1a**, 100 mg, 0.38 mmol, 1 eq, 0.5 M in THF) was added dropwise at -78 °C and the resulting mixture was stirred for 3h at -78°C. After 16 h the reaction mixture was quenched with 5 ml of sat. NH_4Cl solution and extracted x3 with EtOAc. The combined organic layers were then washed x1 with brine, dried over anhydrous Na_2SO_4 and evaporated under reduced pressure to afford a yellow oil. The crude was purified by column chromatography on silica gel (Cyclohexane/EtOAc 9:1 to 4:1). Concentration of the appropriate fractions afforded **1d** as a clear colorless oil.

Yield: 108 mg, 97%.

Rf: 0.1 (90:10 Cyclohexane/EtOAc).

Aspect: colorless oil.

^{1}H NMR (500 MHz, $CDCl_3$) δ (ppm) 6.25 – 6.19 (m, 1H), 3.71 (d, *J* = 1.0 Hz, 3H), 3.42 – 3.35 (m, 1H), 3.18 (d, *J* = 1.0 Hz, 3H), 2.06 – 1.98 (m, 2H), 1.83 – 1.69 (m, 2H), 1.58 – 1.46 (m, 2H), 1.43 (s, 3H), 1.21 (s, 3H).

^{13}C NMR (126 MHz, $CDCl_3$) δ (ppm) 179.7, 130.9, 127.2, 60.4, 45.7, 38.0, 37.5, 33.7, 28.1, 22.7, 21.0, 19.7.

IR (ATR): ν_{max} 2977, 2938, 1651, 1462, 1352, 997, 634 cm^{-1}.

HRMS: (ESI) m/z [M + H]+ calculated for $C_{12}H_{21}BrNO_2$: 290.0750; found: 290.0748

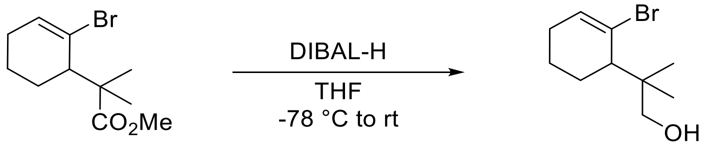

1a **S3**

To a dry flask charged with a solution of 2-(2-bromocyclohex-2-en-1-yl)-2-methylpropanoate (**1a**, 1.54 g, 5.9 mmol, 1 eq, 0.2 M in DCM) under inert atmosphere DIBAL-H (13 ml, 13 mmol, 2.2 eq, 1 M in THF) was added dropwise at -78 °C and left to come at room temperature overnight. After 16 h the reaction mixture was quenched with 15 ml sat. Rochelle salt aq. solution and was left to stir for 1 h followed by extraction x3 with EtOAc. The combined organic layers were then washed x1 with brine, dried over anhydrous Na_2SO_4 and evaporated under reduced pressure to afford a yellow oil. The crude was purified by column chromatography on silica gel (Cyclohexane/EtOAc 95:5). Concentration of the appropriate fractions afforded **S3** as a clear colorless oil.

Yield: 1.29 g, 94%.

Rf: 0.1 (95:5 Cyclohexane/EtOAc).

Aspect: colorless oil.

^{1}H NMR (400 MHz, CDCl$_3$): δ (ppm) 6.24 ('t', J = 4.2 Hz, 1H), 3.86 (d, *J* = 11.2 Hz, 1H), 3.39 (d, *J* = 11.2 Hz, 1H), 2.61 (m, 1H), 2.14 – 2.03 (m, 2H), 1.95 – 1.88 (m, 1H), 1.80 – 1.69 (m, 2H), 1.68 (br, 1H), 1.62 – 1.54 (m, 1H), 1.03 (s, 3H), 1.01 (s, 3H).

^{13}C NMR (101 MHz, CDCl$_3$): δ (ppm) 133.4, 124.1, 71.8, 45.7, 39.4, 27.6, 27.5, 24.6, 23.6, 19.2.

IR (ATR): ν_{max} 3376, 2960, 2938, 2875, 1469, 1038, 664 cm^{-1}.

HRMS: data for this compound could not be obtained due to poor ionization. Instead the GC/MS spectrum is provided among with major fragments.

GC/MS: Rt = 8.508 min

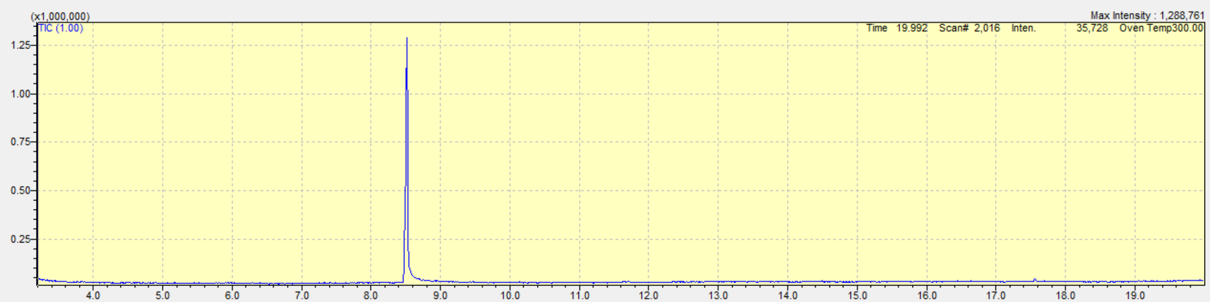

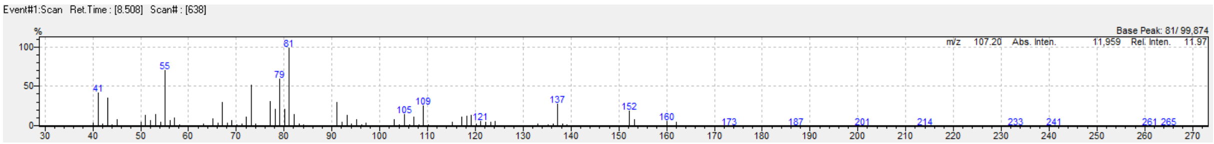

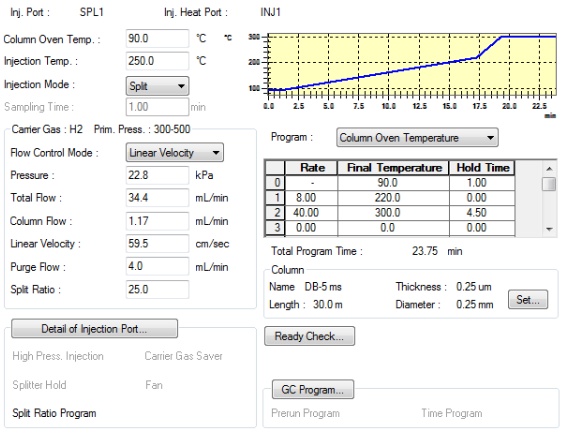

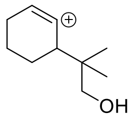

Chemical Formula: $C_{10}H_{17}O^{+}$
Exact Mass: 153.1274

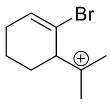

Chemical Formula: $C_9H_{14}Br^{+}$
Exact Mass: 201.0273

Br
Exact Mass: 78.9183

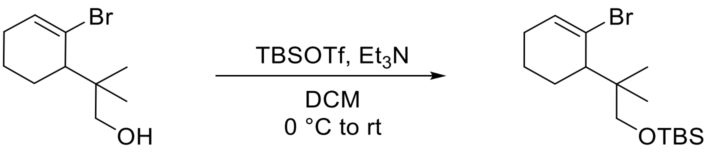

S3 **1e**

To a dry flask charged with a solution of 2-(2-bromocyclohex-2-en-1-yl)-2-methylpropan-1-ol (**S3**, 1.25 g, 5.36 mmol, 1 eq, 0.2 M in DCM), Et_3N (1.11 ml, 814 mg, 8.0 mmol, 1.5 eq) and *tert*-butyldimethylsilyl trifluoromethanesulfonate (1.48 ml, 1.7 g, 6.43 mmol, 1.2 eq) were added at 0 °C and left to reach room temperature overnight. After 16 h the reaction mixture was quenched with 15 ml of 2 M HCl solution. The combined organic layers were then washed x1 with HCl 2 M and x1 with brine, dried over anhydrous Na_2SO_4 and evaporated under reduced pressure to afford a yellow oil. The crude was purified by column chromatography on silica gel (pure Cyclohexane). Concentration of the appropriate fractions afforded **1e** as a clear colorless oil.

Yield: 1.34 g, 75%.

Rf: 0.5 (pure Cyclohexane).

Aspect: colorless oil.

^{1}H NMR (400 MHz, $CDCl_3$): δ (ppm) 6.19 ('t', J = 4.1 Hz, 1H), 3.61 (d, J = 9.5 Hz, 1H), 3.43 (d, J = 9.5 Hz, 1H), 2.60 (td, J = 4.7, 1.9 Hz, 1H), 2.11 – 2.03 (m, 2H), 1.92 (ddt, J = 11.2, 9.5, 4.0 Hz, 1H), 1.76 – 1.62 (m, 2H), 1.62 – 1.55 (m, 1H), 1.06 (s, 3H), 0.95 (s, 3H), 0.90 (s, 9H), 0.05 (s, 3H), 0.04 (s, 3H).

^{13}C NMR (101 MHz, $CDCl_3$): δ (ppm) 132.58, 124.91, 71.72, 45.84, 39.28, 27.74, 27.07, 26.07, 24.93, 24.09, 19.13, 18.45, -5.22, -5.34.

IR (ATR): ν_{max} 2955, 2932, 1254, 1098, 852, 837, 776 cm^{-1}.

HRMS: (ESI) m/z $[M + Ag]^{+}$ calculated for $C_{16}H_{31}AgBrOSi$: 453.0373; found: 453.0365.

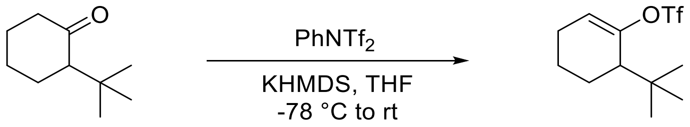

1f

A dry flask charged with KHMDS (15.6 ml, 7.78 mmol, 1.2 eq, 0.5 in THF) was placed at -78 °C under inert atmosphere. After 20 min a solution of 2-tert-butylcyclohexanone (1 g, 6.48 mmol, 1 eq, 0.2 M in THF) was added dropwise. After 30 min a solution of $PhNTf_2$ (2.78 g, 7.78 mmol, 1 eq, 0.3 M in THF) was added dropwise and the mixture was allowed to warm to rt over a period of 4 h and stirred for additional 12 h. After completion the reaction mixture was quenched with 10 ml of NH_4Cl and extracted x3 with EtOAc. The combined organic layers were then washed x1 with brine, dried over anhydrous Na_2SO_4 and evaporated under reduced pressure to afford a yellow oil. The crude was purified by column chromatography on silica gel (pure Cyclohexane). Concentration of the appropriate fractions afforded **1f** as a yellow oil.

Yield: 1.48 g, 80%.

Rf: 0.25 (pure Cyclohexane).

Aspect: colorless oil.

^{1}H NMR (500 MHz, CDCl$_3$): δ (ppm) 5.89 (ddd, *J* = 5.4, 3.3, 1.0 Hz, 1H), 2.33 (ddt, *J* = 7.2, 6.1, 1.4 Hz, 1H), 2.22 – 2.15 (m, 1H), 2.15 – 2.06 (m, 1H), 1.90 – 1.82 (m, 1H), 1.75 – 1.69 (m, 1H), 1.69 – 1.63 (m, 1H), 1.53 – 1.45 (m, 1H), 1.01 (s, 9H).

^{13}C NMR (126 MHz, CDCl$_3$): δ (ppm) 152.2, 121.8, 118.7 (q, *J* = 320.3 Hz), 47.4, 34.2, 29.1, 26.8, 24.5, 20.4.

^{19}F NMR (376 MHz, CDCl$_3$): δ (ppm) -73.8.

IR (ATR): ν_{max} 2963, 1417, 1209, 1144 cm^{-1}.

HRMS: data for this compound could not be obtained due to poor ionization. Instead the GC/MS trace is provided among with major fragments.

GC/MS: Rt = 4.972 min

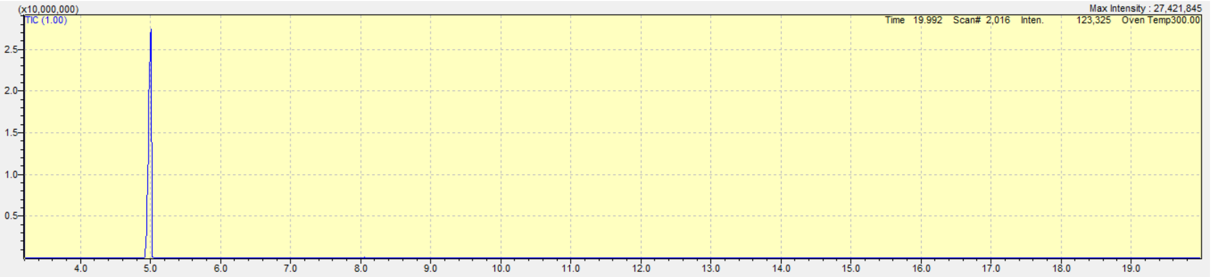

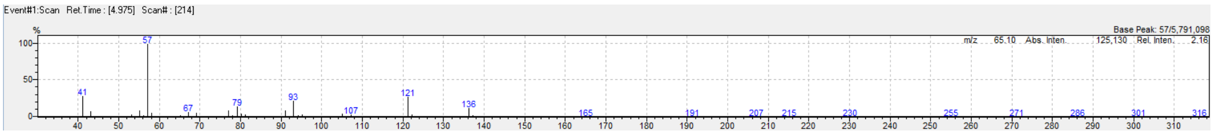

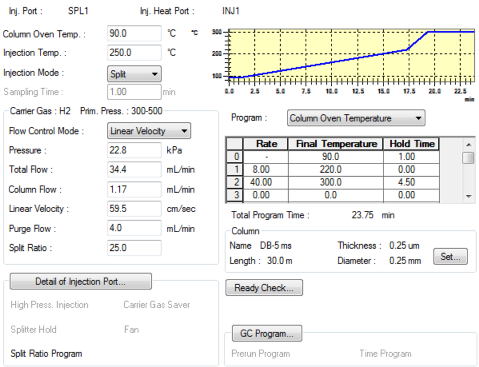

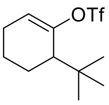

Chemical Formula: $C_{11}H_{17}F_3O_3S$
Exact Mass: 286.0851

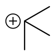

Chemical Formula: $C_4H_9^+$
Exact Mass: 57.0699

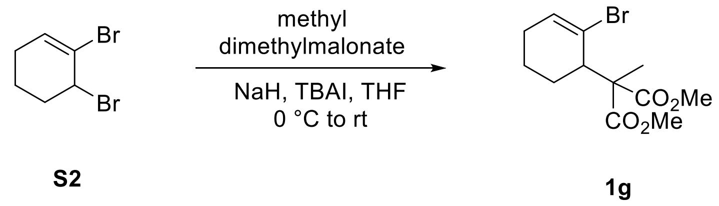

Following general procedure D: using NaH (600 mg, 15 mmol, 1.2 eq, 60% in mineral oil), TBAI (1.85 g, 5 mmol, 0.4 eq) in 20 ml of THF (0.625 M) to react with methyl dimethylmalonate (2.19 g, 15 mmol, 1.2 eq, 0.2 M in THF) and 1,6-dibromoclohex-1-ene (3 g, 12.5 mmol, 1 eq, 0.5 M in THF). After 16 h the reaction mixture was quenched with 10 ml of distilled water and extracted x3 with EtOAc. The combined organic layers were then washed x2 with NaOH 1 M and x1 with brine, dried over anhydrous Na_2SO_4 and evaporated under reduced pressure to afford a yellow oil. The crude was purified by column chromatography on silica gel (Cyclohexane/EtOAc 100:0 to 98:2). Concentration of the appropriate fractions afforded **1g** as a white solid.

Yield: 2.64 g, 68%.

Rf: 0.21 (90:10 Cyclohexane/EtOAc).

Aspect: white solid; m.p. 53.2 – 54.4 °C

^{1}H NMR (400 MHz, $CDCl_3$): δ (ppm) 6.28 ('td', J = 4.3, 1.8 Hz, 1H), 3.76 (s, 3H), 3.72 (s, 3H), 3.72 (m, 1H), 2.07 – 2.01 (m, 2H), 1.79 – 1.76 (m, 1H), 1.74 (q, J = 5.8, 4.7 Hz, 1H), 1.60 – 1.48 (m, 2H), 1.47 (s, 3H).

^{13}C NMR (126 MHz, $CDCl_3$): δ (ppm) 171.8, 171.2, 135.3, 122.7, 58.0, 53.04, 52.97, 45.8, 27.9, 27.5, 21.1, 15.5.

IR (ATR): ν_{max} 2951, 1737, 1434, 1246, 1229 cm^{-1}.

HRMS: (ESI) m/z $[M + H]^+$ calculated for $C_{12}H_{18}BrO_4$: 305.0383; found: 305.0379

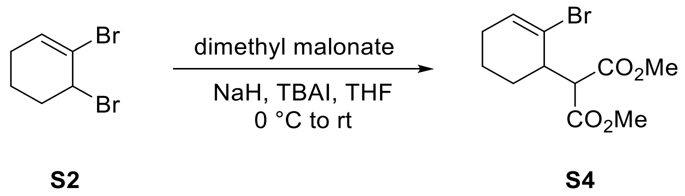

Following general procedure D: using NaH (667 mg, 16.7 mmol, 1 eq, 60% in mineral oil), TBAI (2.46 g, 6.67 mmol, 0.4 eq) in 26.7 ml of THF (0.625 M) to react with methyl dimethylmalonate (2.64 g, 20 mmol, 1.2 eq, 0.2 M in THF) and 1,6-dibromoclohex-1-ene (4 g, 16.7 mmol, 1 eq, 0.5 M in THF). After 16 h the reaction mixture was quenched with 10 ml of distilled water and extracted x3 with EtOAc. The combined organic layers were then washed x2 with NaOH 1 M and x1 with brine, dried over anhydrous Na_2SO_4 and evaporated under reduced pressure to afford a yellow oil. The crude was purified by column chromatography on silica gel (Cyclohexane/EtOAc 100:0 to 98:2). Concentration of the appropriate fractions afforded **S4** as a colorless oil. ^{1}H and ^{13}C NMR data match those reported in the literature.[2]

Yield: 1.92 g, 40%.

^{1}H NMR (400 MHz, CDCl$_3$): δ (ppm) 6.22 (ddd, J = 5.2, 3.5, 1.8 Hz, 1H), 4.04 (d, J = 5.4 Hz, 1H), 3.76 (s, 3H), 3.73 (s, 3H), 3.18 – 3.09 (m, 1H), 2.17 – 2.04 (m, 2H), 2.03 – 1.95 (m, 1H), 1.94 – 1.84 (m, 1H), 1.72 – 1.63 (m, 1H), 1.64 – 1.50 (m, 1H).

^{13}C NMR (126 MHz, CDCl$_3$): δ (ppm) 169.2, 168.5, 133.2, 123.5, 54.2, 52.8, 52.4, 42.5, 27.6, 26.8, 20.1.

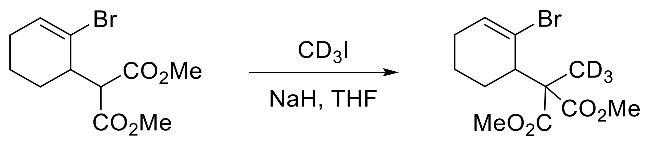

S4 ***d*₃-1g**

To a dry flask NaH (260 mg, 1 eq, 60% in mineral oil) and THF (0.625 M) were added and the flask was placed at -10 °C. After 10 min a solution of dimethyl 2-(2-bromocyclohex-2-en-1-yl)malonate (**S4**, 1.9 g, 1 eq, 0.2 M in THF) was added dropwise and the flask was left for 30 min at 0°C. Then CD_3I (1.14 mg, 0.48 ml, 1.2 eq, 0.5 M in THF) was added dropwise and the flask was left to reach room temperature. After completion the reaction mixture was quenched with distilled water and extracted x3 with EtOAc. The combined organic layers were then washed x1 with brine, dried over anhydrous Na_2SO_4 and evaporated under reduced pressure. The crude was purified by column chromatography on silica gel (Cyclohexane/EtOAc 100:0 to 98:2). Concentration of the appropriate fractions afforded ***d*$_3$-1g** as a white solid.

Yield: 1.71 g, 85%.

Rf: 0.21 (90:10 Cyclohexane/EtOAc).

Aspect: white solid; m.p. 50.8 – 52.5 °C

^{1}H NMR (500 MHz, CDCl$_3$): δ (ppm) 6.29 – 6.25 (m, 1H), 3.75 (s, 3H), 3.74 – 3.69 (m, 1H), 3.72 (s, 3H), 2.05 (m, 1H), 2.04 – 2.01 (m, 1H), 1.78 – 1.75 (m, 1H), 1.75 – 1.72 (m, 1H), 1.62 – 1.52 (m, 1H), 1.52 – 1.44 (m, 1H).

^{13}C NMR (126 MHz, CDCl$_3$): δ (ppm) 171.7, 171.1, 135.2, 122.7, 57.7, 53.0, 52.9, 45.7, 27.8, 27.4, 21.0, 14.8-14.5 (m, CD$_3$).

IR (ATR): ν_{max} 2953, 1738, 1713, 1435, 1265, 1252 cm^{-1}.

HRMS: (ESI) m/z [M + H]$^+$ calculated for $C_{12}H_{15}BrD_3O_4$: 308.0571; found: 308.0567

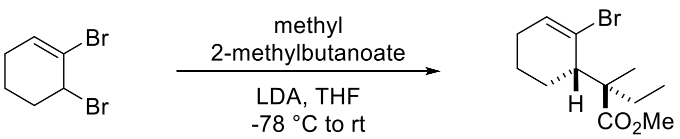

S2

d.r. 1.2:1
1h

To a dry flask charged with a solution of diisopropylamine (253 mg, 2.5 mmol, 1.2 eq, 1 M in THF) under inert atmosphere n-BuLi (1.56 ml, 1.6 M in Hexanes, 1.2 eq) was added at -78 °C. After 30 min a solution of methyl 2-methylbutanoate (290 mg, 2.5 mmol, 1.2 eq, 0.25 M in THF) was added dropwise at -78 °C. After 30 min a solution of 1,6-dibromocyclohex-1-ene (**S2**, 500 mg, 2.08 mmol, 1 eq, 0.5 M in THF)

was added dropwise and the reaction was left to reach room temperature. After 16 h the reaction mixture was quenched with 10 ml of sat. NH_4Cl solution and extracted x3 with EtOAc. The combined organic layers were then washed x1 with brine, dried over anhydrous Na_2SO_4 and evaporated under reduced pressure to afford a yellow oil. The crude was purified by column chromatography on silica gel (Cyclohexane/EtOAc 100:0 to 99:1). Concentration of the appropriate fractions afforded **1h** as a yellow oil (mixture of two diastereomers, d.r. 1.2:1).

Yield: 220 mg, 38%.

Rf: 0.46 (90:10 Cyclohexane/EtOAc).

Aspect: yellow oil.

^{1}H NMR (500 MHz, $CDCl_3$): δ (ppm) 6.27 (dd, *J* = 5.0, 3.6 Hz, 0.53H, *major*), 6.22 (dd, *J* = 5.2, 3.6, Hz, 0.47H, *minor*), 3.69 (s, 1.4H, *minor*), 3.68 (s, 1.6H, *major*), 3.25 (m, 0.48H, *minor*), 2.98 (m, 0.54H, *major*), 2.05 – 2.01 (m, 1H), 2.01 – 1.95 (m, 1H), 1.94 – 1.88 (m, 1H), 1.83 – 1.77 (m, 1H), 1.77 – 1.71 (m, 1H), 1.58 – 1.50 (m, 2H), 1.49 – 1.42 (m, 1H), 1.15 (s, 1.66H, *major*), 1.12 (s, 1.51H, *minor*), 0.88 (t, J = 7.4 Hz, 1.8H, *major*), 0.78 (t, J = 7.5 Hz, 1.56H, *minor*).

^{13}C NMR (126 MHz, $CDCl_3$): δ (ppm) *major diastereomer*: 177.6, 134.5, 123.5, 51.7, 50.5, 48.9, 29.8, 28.4, 27.8, 20.3, 19.0, 9.9. *minor diastereomer*: 177.9, 134.0, 125.6, 51.9, 49.4, 47.7, 31.7, 28.0, 26.5, 21.5, 15.3, 9.2

IR (ATR): ν_{max} 2971, 2945, 1729, 1458, 1236, 631 cm^{-1}.

HRMS: (ESI) m/z $[M + H]^+$ calculated for $C_{12}H_{19}BrNaO_2$: 297.0461; found: 297.0460.

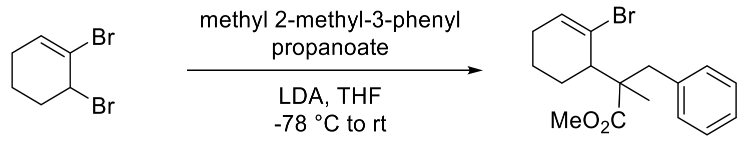

S2

d.r. 1.6:1
1i

Following general procedure C: using diisopropylamine (342 mg, 3.4 mmol, 1.2 eq, 1 M in THF), n-BuLi (2.11 ml, 3.4 mmol, 1.2 eq, 1.6 M in Hexanes), methyl 2-methyl-3-phenylpropanoate (600 mg, 3.4 mmol, 1.2 eq, 0.25 M in THF) and 1,6-dibromocyclohex-1-ene (**S2**, 674 mg, 2.8 mmol, 1 eq, 0.5 M in THF). After 16 h the reaction mixture was quenched with 10 ml of sat. NH_4Cl solution and extracted x3 with EtOAc. The combined organic layers were then washed x1 with brine, dried over anhydrous Na_2SO_4 and evaporated under pressure to afford a yellow oil. The crude was purified by column chromatography on silica gel (Cyclohexane/EtOAc 97:3). Concentration of the appropriate fractions afforded **1i** as an impure mixture (d.r. 1.6:1). **1i** was used to the next step without further purification. The ^{1}H NMR spectrum of the impure mixture is given as proof of the diastereomeric ratio (key ^{1}H peaks are integrated).

Rf: 0.42 (90:10 Cyclohexane/EtOAc).

IR (ATR): ν_{max} 2365, 2358, 1735 cm^{-1}.

HRMS: (ESI) m/z $[M + Na]^+$ calculated for $C_{17}H_{21}BrNaO_2$: 359.0617; found: 359.0617.

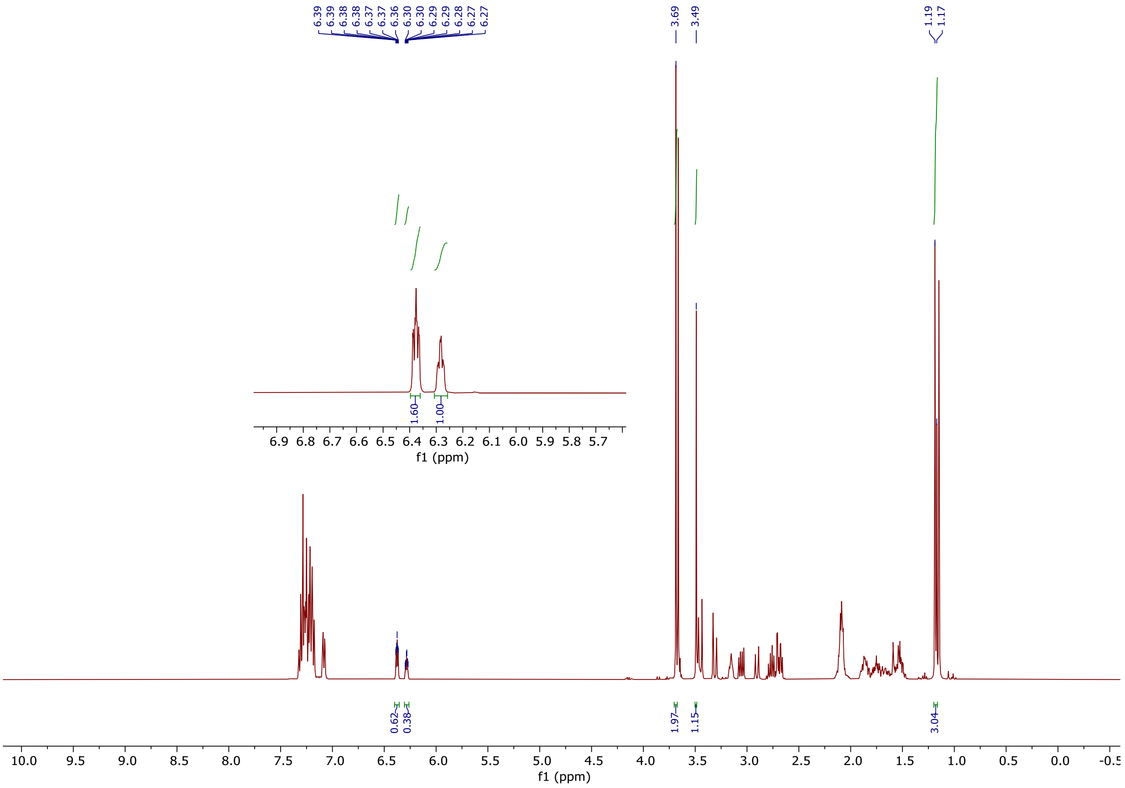

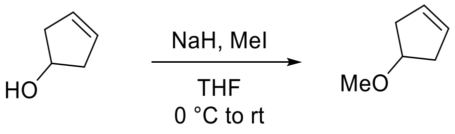

S5

To a dry flask NaH (1.14 g, 1.2 eq, 60% in mineral oil) and THF (1M) were added and the flask was placed at -10 °C. After 10 min a solution of 3-cyclopenten-1-ol (2 g, 23.8 mmol, 1 eq, 0.4 M in THF) was added dropwise and the flask was left for 30 min at 0°C. Then iodomethane (3.4 g, 1.5 ml, 1 eq) was added dropwise and the flask was left to reach room temperature. After completion the reaction mixture was quenched with distilled water and extracted x3 with EtOAc. The combined organic layers were then washed x1 with brine, dried over anhydrous Na_2SO_4 and evaporated under reduced pressure (minimum 200 mbar). The crude was used in the next step without purification.

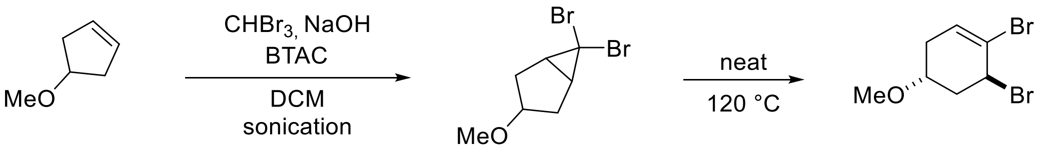

S5 **S6** **S7**

Following general procedure A: using sodium hydroxide (2.45 g, 61.2 mmol, 6 eq) in 5.6 ml of DCM, benzyltriethylammonium chloride (116 mg, 0.51 mmol, 0.05 eq), 4-methoxycyclopent-1-ene (**S5**, 1 g, 10.2 mmol, 1 eq) and bromoform (5.16 ml, 20.4 mmol, 2 eq). The flask was fitted to a reflux condenser and subjected to sonication for 1 h. The cooled reaction mixture was filtered through a short pad of celite and then washed with 40 ml DCM. The combined filtrates were concentrated under reduced

pressure and the obtained brown oil was subjected to the next step. A sealed flask containing **S6** was heated at 110 °C under neat conditions for 2h. The crude was purified by column chromatography on silica gel (Cyclohexane/EtOAc 100:0 to 95:5). Concentration of the appropriate fractions afforded **S7** as a dark yellow oil (single diastereomer).

Note: Compound **S7** is prone to elimination and aromatization and should be stored in the freezer.

Yield: 1.45 g, 53% (over two steps).

Rf: 0.3 (90:10 Cyclohexane/EtOAc).

Aspect: yellow oil.

^{1}H NMR (500 MHz, CDCl3): δ (ppm) 6.09 (dd, *J* = 5.7, 2.8 Hz, 1H), 4.84 – 4.81 (m, 1H), 3.94 – 3.87 (m, 1H), 3.40 (s, 3H), 2.69 (dtdd, *J* = 17.9, 5.7, 1.7, 0.6 Hz, 1H), 2.57 (dddd, *J* = 14.0, 3.3, 2.7, 1.6 Hz, 1H), 2.18 – 2.14 (m, 1H), 2.14 – 2.10 (m, 1H).

^{13}C NMR (126 MHz, $CDCl_3$): δ (ppm) 131.5, 122.1, 71.1, 56.4, 52.7, 38.8, 34.0.

IR (ATR): ν_{max} 2953, 2921, 652 cm^{-1}.

HRMS: data for this compound could not be obtained due to poor ionization. Instead the GC/MS spectrum is provided among with major fragments.

GC/MS: Rt = 6.825 min

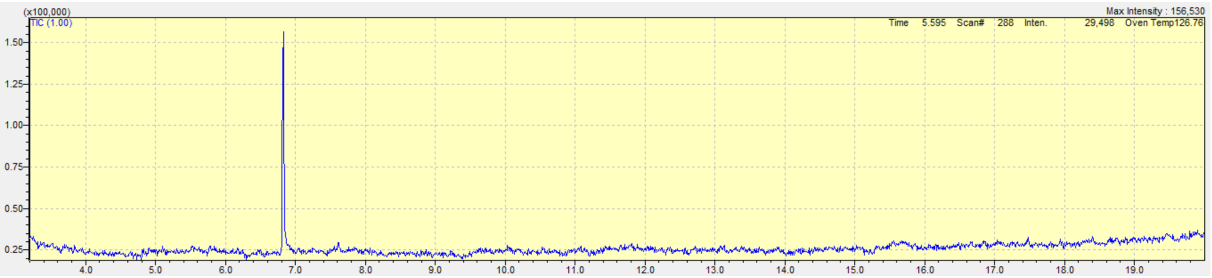

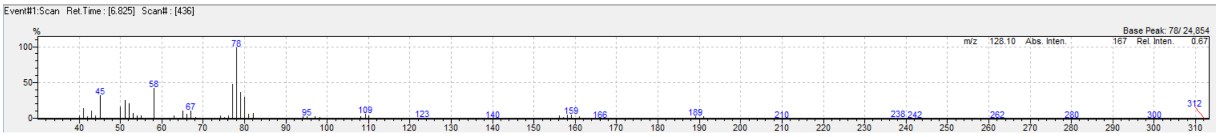

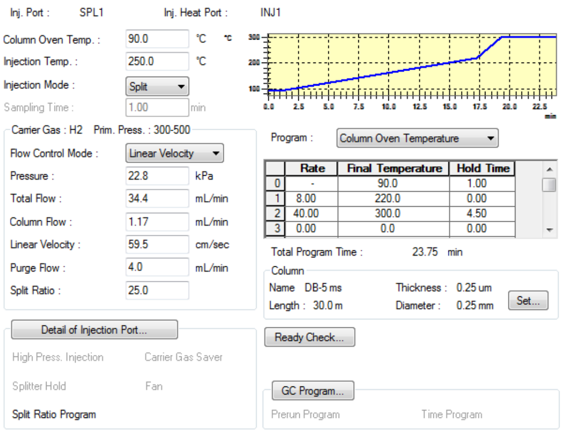

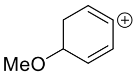

Chemical Formula: $C_7H_9O^+$
Exact Mass: 109.0648

Br
Exact Mass: 78.9183

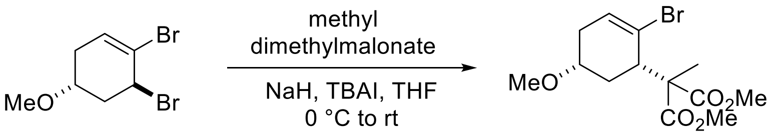

S7

d.r. 2:1
1j

Following general procedure D: using NaH (74 mg, 1.85 mmol, 1.2 eq, 60% in mineral oil), TBAI (273 g, 0.74 mmol, 0.4 eq) in 10 ml of THF (0.125 M) to react with methyl dimethylmalonate (270 mg, 1.85 mmol, 1.2 eq, 0.2 M in THF) and 1,6-dibromo-4-methoxycyclohex-1-ene (**S7**, 500 g, 1.85 mmol, 1 eq, 0.5 M in THF). After 16 h the reaction mixture was quenched with 10 ml of distilled water and extracted x3 with EtOAc. The combined organic layers were then washed x2 with NaOH 1 M and x1 with brine, dried over anhydrous Na_2SO_4 and evaporated under reduced pressure to afford a yellow oil. The crude was purified by column chromatography on silica gel (Cyclohexane/EtOAc 100:0 to 90:10). Concentration of the appropriate fractions afforded **1j** as a yellow oil (d.r. 2:1).

Yield: 320 mg, 52%.

Rf: 0.1 (90:10 Cyclohexane/EtOAc).

Aspect: yellow oil

^{1}H NMR (500 MHz, $CDCl_3$): δ (ppm) 6.14 – 6.09 (m, 1H), 3.84 (dddd, *J* = 11.4, 6.1, 4.8, 2.8 Hz, 1H), 3.75 (s, 3H), 3.73 (s, 1H), 3.72 (s, 2H), 3.63 (m, 0.34H, *minor*), 3.44 (m, 0.66H, *major*), 3.33 (s, 3H), 2.48 – 2.41 (m, 0.66H, *major*), 2.32 – 2.25 (m, 0.34H, *minor*), 2.18 (dddt, *J* = 18.1, 5.5, 3.8, 2.0 Hz, 0.34H, *minor*), 2.02 – 1.97 (m, 0.66H, *major*), 1.96 (ddd, *J* = 5.5, 3.1, 1.5 Hz, 0.34H, *minor*), 1.94 – 1.90 (m, 0.66H, *major*), 1.65 – 1.57 (m, 0.34H, *minor*), 1.49 (s, 1H), 1.46 (s, 2H), 1.40 (q, *J* = 11.6 Hz, 0.66H, *major*).

^{13}C NMR (126 MHz, $CDCl_3$): δ (ppm) *major diastereomer*: 171.3, 170.7, 131.8, 122.5, 74.7, 57.8, 56.19, 53.12, 53.10, 46.2, 33.6, 32.7, 14.8. *minor diastereomer*: 171.6, 171.2, 131.1, 121.9, 72.2, 57.6, 56.15, 53.0 (2C), 42.5, 33.0, 30.8, 16.2.

IR (ATR): ν_{max} 2952, 1736, 1453, 1435, 1251, 1225, 1108, 635 cm^{-1}.

HRMS: (ESI) m/z [M + Na]$^+$ calculated for $C_{13}H_{19}BrNaO_5$: 357.0308; found: 357.0308

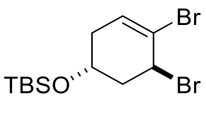

d.r. 10:1
S8

S8 was synthesized according to literature[3] and isolated as a mixture of diastereomers (10:1).

^{1}H NMR (500 MHz, $CDCl_3$): δ (ppm) 6.15 (ddd, *J* = 5.9, 3.1, 1.0 Hz, 0.1H, *minor*), 6.07 (dd, *J* = 5.7, 2.7 Hz, 0.9H, *major*), 4.80 (ddd, *J* = 4.2, 2.4, 1.3 Hz, 1H), 4.36 (dddd, *J* = 11.1, 9.2, 5.7, 3.4 Hz, 0.9H, *major*), 3.91 (dddd, *J* = 10.1, 8.0, 5.0, 3.3 Hz, 0.1H, *minor*), 2.59 – 2.55 (m, 0.1H, *minor*), 2.51 (dtd, *J* = 18.0, 5.7, 1.7 Hz, 0.9H, *major*), 2.41 – 2.35 (m, 1H), 2.25 – 2.21 (m, 1H), 2.19 (dddd, *J* = 11.9, 8.1, 2.6, 1.2 Hz, 1H), 0.90 (s, 8.2H, *major*), 0.89 (s, 0.8H, *minor*), 0.10 (d, *J* = 1.7 Hz, 5.4H, *major*), 0.07 (d, *J* = 6.0 Hz, 0.6H, *minor*).

^{13}C NMR (126 MHz, CDCl$_3$): δ (ppm) *major diastereomer*: 132.2, 122.0, 63.3, 53.5, 42.7, 37.5, 26.0, 18.2, -4.58, -4.61.

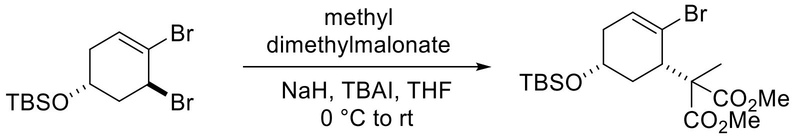

d.r. 10:1
S8

d.r. 6.4:1
1k

Following general procedure D: using NaH (97 mg, 2.42 mmol, 1.2 eq, 60% in mineral oil), TBAI (298 g, 0.81 mmol, 0.4 eq) in 16 ml of THF (0.125 M) to react with methyl dimethylmalonate (354 mg, 2.42 mmol, 1.2 eq, 0.2 M in THF) and *tert*-butyl((4,5-dibromocyclohex-3-en-1-yl)oxy)dimethylsilane (**S8**, 1 g, 2.02 mmol, 1 eq, 0.5 M in THF). After 16 h the reaction mixture was quenched with 10 ml of distilled water and extracted x3 with EtOAc. The combined organic layers then washed x2 with NaOH 1 M and x1 with brine, dried over anhydrous Na_2SO_4 and evaporated under reduced pressure to afford a yellow oil. The crude was purified by column chromatography on silica gel (Cyclohexane/EtOAc 95:5). Concentration of the appropriate fractions afforded **1k** as a white solid (d.r. 6.4:1).

Yield: 875 mg, 77%.

Rf: 0.2 (Cyclohexane/EtOAc 95:5).

Aspect: white solid; m.p. 70.7 – 73.2 °C

^{1}H NMR (500 MHz, CDCl$_3$): δ (ppm) 6.10 ('dt', *J* = 6.8, 2.1 Hz, 1H), 4.13 – 4.07 (m, 0.14H, *minor*), 3.91 – 3.86 (m, 0.86H, *major*), 3.86 – 3.80 (m, 1H), 3.76 (s, 3H), 3.72 (s, 3H), 2.32 – 2.27 (m, 0.14H, *minor*), 2.26 – 2.19 (m, 0.86H, *major*), 2.02 (dddd, *J* = 19.0, 9.8, 4.3, 2.3 Hz, 1H), 1.78 (dddd, *J* = 12.3, 6.1, 3.7, 2.6 Hz, 0.86H, *major*), 1.75 – 1.71 (m, 0.14H, *minor*), 1.61 – 1.56 (m, 0.14H, *minor*), 1.55 – 1.47 (m, 0.86H, *major*), 1.47 (s, 0.4H, *minor*), 1.46 (s, 2.6H, *major*), 0.88 (s, 1.22H, *minor*), 0.87 (s, 7.78H, *major*), 0.06 (s, 0.82H, *minor*), 0.04 (s, 5.18H, *major*).

^{13}C NMR (126 MHz, CDCl$_3$): δ (ppm) *major diastereomer*: 171.4, 170.7, 132.4, 122.2, 67.0, 57.8, 53.1, 53.0, 46.4, 37.4, 36.5, 26.0, 18.3, 14.7, -4.5, -4.6. *minor diastereomer*: 171.7, 171.2, 131.3, 121.5, 64.0, 57.6, 53.0, 52.9, 43.2, 36.9, 35.1, 25.9, 18.2, 16.5, -4.6, -4.7.

IR (ATR): ν_{max} 2953, 2931, 1739, 1253, 1224, 1107, 837, 778, 670 cm^{-1}.

HRMS: (ESI) m/z [M + H]$^+$ calculated for $C_{18}H_{32}BrO_5Si$: 435.1197; found: 435.1192

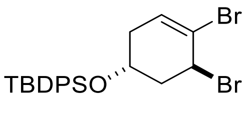

d.r. 4.4:1
S9

S9 was synthesized according to literature[3] and isolated as a mixture of diastereomers (4.4:1).

^{1}H NMR (400 MHz, CDCl$_3$): δ (ppm) 7.67 (ddd, *J* = 8.1, 5.1, 1.6 Hz, 4H), 7.47 – 7.36 (m, 6H), 6.04 (ddd, *J* = 5.8, 3.3, 0.9 Hz, 0.18H, *minor*), 5.96 (dd, *J* = 5.5, 2.8 Hz, 0.82H, *major*), 4.69 (t, *J* = 3.8 Hz, 0.82H, *minor*),

4.66 – 4.60 (m, 0.18H, *minor*), 4.39 (dddd, *J* = 10.8, 9.3, 5.7, 3.6 Hz, 0.82H, *major*), 3.90 (m, 0.18H, *minor*), 2.55 – 2.32 (m, 2H), 2.32 – 2.03 (m, 2H), 1.08 (s, 9H).

^{13}C NMR (126 MHz, CDCl$_3$): δ (ppm) *major diastereomer*: 135.9, 135.8, 132.0, 127.90, 127.85, 121.8, 64.2, 52.8, 42.2, 37.2, 27.1, 19.3. *minor diastereomer*: 134.0, 133.8, 131.3, 130.00, 129.97, 122.8, 66.3, 50.0, 43.5, 36.6, 27.0, 19.2

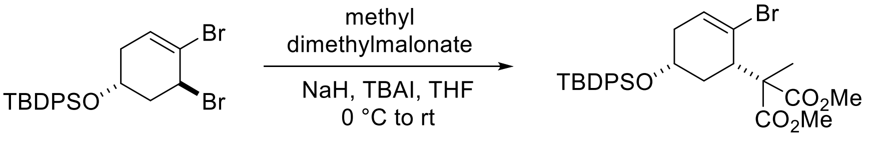

d.r. 4.4:1 **S9** d.r. 5.3:1 **1l**

Following general procedure D: using NaH (97 mg, 2.42 mmol, 1.2 eq, 60% in mineral oil), TBAI (298 g, 0.81 mmol, 0.4 eq) in 16 ml of THF (0.125 M) to react with methyl dimethylmalonate (354 mg, 2.42 mmol, 1.2 eq, 0.2 M in THF) and *tert*-butyl((4,5-dibromocyclohex-3-en-1-yl)oxy)diphenylsilane (**S9**, 1 g, 2.02 mmol, 1 eq, 0.5 M in THF). After 16 h the reaction mixture was quenched with 10 ml of distilled water and extracted x3 with EtOAc. The combined organic layers were then washed x2 with NaOH 1 M and x1 with brine, dried over anhydrous Na_2SO_4 and evaporated under reduced pressure to afford a yellow oil. The crude was purified by column chromatography on silica gel (Cyclohexane/EtOAc 100:0 to 97:3). Concentration of the appropriate fractions afforded **1l** as a yellow oil (d.r. 5.3:1).

Yield: 875 mg, 77%.

Rf: 0.4 (85:15 Cyclohexane/EtOAc).

Aspect: yellow oil

^{1}H NMR (400 MHz, CDCl$_3$): δ (ppm) 7.63 (ddd, *J* = 6.6, 3.9, 1.7 Hz, 4H), 7.44 (dddd, *J* = 8.8, 6.4, 3.0, 1.5 Hz, 2H), 7.40 – 7.34 (m, 4H), 6.08 (ddd, *J* = 5.2, 3.2, 1.8 Hz, 0.16H, *minor*), 5.98 (dt, *J* = 6.4, 2.5 Hz, 0.84H, *major*), 4.15 – 4.10 (m, 0.16H, *minor*), 4.01 (s, 0.16H, *minor*), 3.89 (dddd, *J* = 11.5, 9.3, 5.8, 3.8 Hz, 0.84H, *major*), 3.74 (s, 0.47H, *minor*), 3.71 (s, 2.52H, *major*), 3.65 – 3.60 (m, 0.84H, *major*), 3.62 (s, 0.47H, *minor*), 3.59 (s, 2.52H, *major*), 2.20 – 2.04 (m, 2H), 1.77 (dddd, *J* = 12.0, 6.0, 3.7, 2.1 Hz, 1H), 1.57 (q, *J* = 11.6 Hz, 1H), 1.43 (s, 2.52H, *major*), 1.34 (s, 0.47H, *minor*), 1.07 (s, 1.43H, *minor*), 1.04 (s, 7.57H, *major*).

^{13}C NMR (126 MHz, CDCl$_3$): δ (ppm) *major diastereomer*: 171.3, 170.6, 135.81, 135.80, 132.2, 129.94, 129.91, 127.83, 127.82, 122.1, 67.7, 57.7, 53.03, 52.97, 46.1, 37.1, 36.2, 27.0, 19.2, 14.8.

IR (ATR): ν_{max} 2952, 2933, 1736, 1251, 1224, 1108, 736, 706, 613 cm^{-1}.

HRMS: (ESI) m/z [M + H]$^+$ calculated for $C_{28}H_{36}BrO_5Si$: 559.1510; found: 559.1497

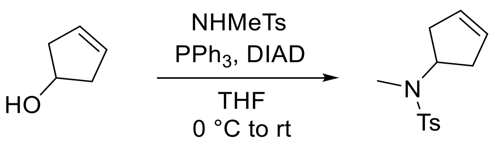

S10

To a dry flask charged with a solution of cyclopent-3-en-1-ol (500 mg, 5.94 mmol, 1 eq, 0.2 M in THF), N-methyl-4-methylbenzenesulfonamide (1.2 g, 6.53 mmol, 1.1 eq) and triphenylphosphine (1.7 g, 6.53 mmol., 1.1 eq) at 0°C diethyl azodicarboxylate (1.3 mL, 1.32 g, 6.53 mmol, 1.1 eq) was added dropwise during a period of 10 min and the reaction was stirred overnight at room temperature. After 16 h the reaction mixture was quenched with 10 ml of H_2O and extracted x3 with EtOAc. The combined organic layers were then washed x1 with brine, dried over anhydrous Na_2SO_4 and evaporated under pressure to afford a yellow oil. The crude was purified by column chromatography on silica gel (Cyclohexane/EtOAc 100:0 to 95:5). Concentration of the appropriate fractions afforded **S10** as a white solid.

Yield: 700 mg, 47%.

Rf: 0.34 (80:20 Cyclohexane/EtOAc).

Aspect: white solid; m.p. 39.2 – 43.2 °C

^{1}H NMR (400 MHz, $CDCl_3$): δ (ppm) 7.69 (d, J = 8.3 Hz, 2H), 7.30 (d, J = 7.9 Hz, 2H), 5.61 – 5.57 (m, 2H), 4.78 (tt, J = 8.8, 4.2 Hz, 1H), 2.60 (s, 3H), 2.50 – 2.43 (m, 2H), 2.43 (s, 3H), 2.08 – 2.05 (m, 1H), 2.02 – 2.00 (m, 1H).

^{13}C NMR (101 MHz, $CDCl_3$): δ (ppm) 143.3, 136.4, 129.8, 129.1, 127.4, 55.4, 35.9, 28.6, 21.7.

IR (ATR): ν_{max} 2921, 2853, 1456, 1338, 1159 cm^{-1}.

HRMS: (ESI) m/z $[M + H]^+$ calculated for $C_{13}H_{18}NO_2S$: 252.1053; found: 252.1048.

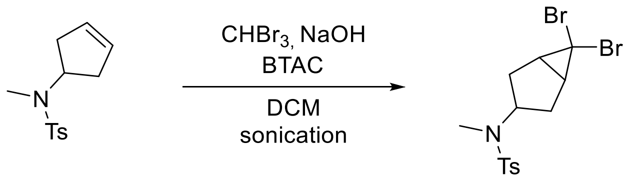

S10 **S11**

Following general procedure A: using sodium hydroxide (667 mg, 16.7 mmol, 6 eq) in 1.5 ml of DCM, benzyltriethylammonium chloride (32 mg, 0.14 mmol, 0.05 eq), N-(cyclopent-3-en-1-yl)-N,4-dimethylbenzenesulfonamide (**S10**, 700 mg, 2.78 mmol, 1 eq) and bromoform (0.49 ml, 5.56 mmol, 2 eq). The flask was fitted to a reflux condenser and subjected to sonication for 1 h. The cooled reaction mixture was filtered through a short pad of celite and then washed with 10 ml DCM. The combined filtrates were concentrated under reduced pressure to afford a yellow oil. The crude was purified by column chromatography on silica gel (Cyclohexane/EtOAc 100:0 to 90:10). Concentration of the appropriate fractions afforded **S11** as a yellow solid.

Yield: 1.45 g, 53%.

Rf: 0.16 (80:20 Cyclohexane/EtOAc).

Aspect: yellow solid; m.p. 124.8 – 127.5 °C

^{1}H NMR (400 MHz, $CDCl_3$): δ (ppm) 7.65 (d, *J* = 8.4 Hz, 2H), 7.31 (d, *J* = 8.0 Hz, 2H), 4.65 (tt, *J* = 8.8, 5.6 Hz, 1H), 2.66 (s, 3H), 2.44 (s, 3H), 2.22 (m, 1H), 2.21 – 2.18 (m, 1H), 1.98 (d, *J* = 8.9 Hz, 1H), 1.94 (d, *J* = 8.9 Hz, 1H), 1.80 – 1.76 (m, 1H), 1.74 (m, 1H).

^{13}C NMR (101 MHz, $CDCl_3$): δ (ppm) 143.6, 135.7, 129.9, 127.4, 60.4, 39.3, 37.6, 32.3, 28.8, 21.7.

IR (ATR): ν_{max} 1138, 1160, 665, 625 cm^{-1}.

HRMS: (ESI) m/z [M + Na]$^+$ calculated for $C_{14}H_{17}Br_2NNaO_2S$: 443.9239; found: 443.9234.

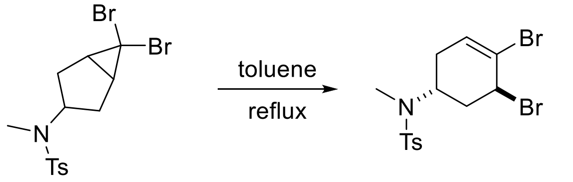

S11 **S12** single diastereomer

A flask charged with a solution of N-(6,6-dibromobicyclo[3.1.0]hexan-3-yl)-N,4-dimethylbenzenesulfonamide (**S11**, 330 mg, 0.78 mmol, 1 eq) in 3.9 ml of toluene (0.2 M) was refluxed overnight. After 16 h toluene was evaporated under reduced pressure and the yellow solid was triturated with Cyclohexane/EtOAc 9:1 to obtain **S12** as a white solid (single diastereomer).

Yield: 200 mg, 61%.

Rf: 0.1 (90:10 Petroleum ether/EtOAc).

Aspect: white solid; m.p. 147.0 – 148.8 °C

1H NMR (500 MHz, CDCl$_3$): δ (ppm) 7.77 (d, *J* = 8.3 Hz, 2H), 7.32 (d, *J* = 7.6 Hz, 2H), 6.10 – 6.06 (m, 1H), 4.70 (dd, *J* = 4.3, 2.2 Hz, 1H), 4.68 – 4.60 (m, 1H), 2.74 (s, 3H), 2.43 (s, 3H), 2.39 – 2.34 (m, 2H), 2.15 (ddd, *J* = 13.7, 12.7, 4.2 Hz, 1H), 1.83 – 1.78 (m, 1H).

^{13}C NMR (126 MHz, CDCl$_3$): δ (ppm) 143.7, 136.4, 132.1, 130.0, 127.4, 121.6, 52.7, 48.3, 36.2, 31.6, 29.1, 21.7.

IR (ATR): ν_{max} 2923, 1336, 1162, 662 cm^{-1}.

HRMS: (ESI) m/z [M + Na]$^+$ calculated for $C_{14}H_{17}Br_2NNaO_2S$: 443.9239; found: 443.9234.

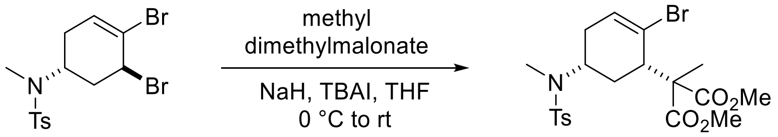

S12 d.r. 4.6:1 **1m**

Following general procedure D: using NaH (23 mg, 0.57 mmol, 1.2 eq, 60% in mineral oil), TBAI (70 g, 0.19 mmol, 0.4 eq) in 6 ml of THF (0.125 M) to react with methyl dimethylmalonate (83 mg, 0.57 mmol, 1.2 eq, 0.2 M in THF) and N-(4,5-dibromocyclohex-3-en-1-yl)-N,4-dimethylbenzenesulfonamide (**S12**, 200 mg, 0.48 mmol, 1 eq, 0.5 M in THF). After 16 h the reaction mixture was quenched with 10 ml of distilled water and extracted x3 with EtOAc. The combined organic layers were then washed x2 with NaOH 1 M and x1 with brine, dried over anhydrous Na_2SO_4 and evaporated under reduced pressure to afford a yellow oil. The crude was purified by column chromatography on silica gel (Cyclohexane/EtOAc 100:0 to 85:15). Concentration of the appropriate fractions afforded **1m** as a yellow oil (d.r. 4.6:1).

Yield: 162 mg, 70%.

Rf: 0.16 (75:25 Cyclohexane/EtOAc).

Aspect: yellow oil

^{1}H NMR (500 MHz, CDCl$_3$): δ (ppm) 7.68 (d, *J* = 8.3 Hz, 2H), 7.31 (d, *J* = 7.8 Hz, 2H), 6.14 (td, *J* = 4.2, 1.0 Hz, 0.18H. *minor*), 6.11 (dt, *J* = 6.8, 2.1 Hz, 0.82H, *major*), 4.28 (dtd, *J* = 11.8, 7.9, 3.6 Hz, 0.18H, *minor*), 4.16 (dddd, *J* = 12.3, 11.1, 5.0, 3.2 Hz, 0.82H, *major*), 3.84 (dtd, *J* = 10.7, 4.1, 2.1 Hz, 0.82H, *major*), 3.75 (s, 0.54H, *minor*), 3.75 (s, 2.46H, *major*), 3.73 – 3.71 (m, 0.18H, *minor*), 3.71 (s, 0.54H, *minor*), 3.59 (s, 2.46H, *major*), 2.71 (s, 0.54H, *minor*), 2.71 (s, 2.46H, *major*), 2.42 (s, 3H), 2.10 (dddd, *J* = 17.4, 11.2, 4.2, 2.3 Hz, 0.82H, *major*), 2.05 (dd, *J* = 4.2, 1.7 Hz, 0.18H, *minor*), 2.03 (d, *J* = 1.7 Hz, 0.18H, *minor*), 2.02 – 1.95 (m, 0.18H, *minor*), 1.88 (dddt, *J* = 16.6, 7.0, 4.7, 2.2 Hz, 0.82H, *major*), 1.66 (ddd, *J* = 14.0, 3.6, 2.3 Hz, 0.18H, *minor*), 1.50 (s, 0.54H, *minor*), 1.55 – 1.44 (m, 0.82H, *major*), 1.42 (dq, *J* = 5.9, 3.0 Hz, 0.82H, *major*), 1.40 (s, 2.46H, *major*).

^{13}C NMR (126 MHz, CDCl$_3$): δ (ppm) *major diastereomer*: 171.1, 170.5, 143.5, 136.9, 132.4, 130.0, 127.1, 122.1, 57.6, 53.1, 52.0, 46.4, 30.7, 30.6, 28.8, 21.6, 15.2.

IR (ATR): ν_{max} 2952, 1733, 1336, 1251, 1222, 1159, 664, 632 cm^{-1}.

HRMS: (ESI) m/z [M + H]$^+$ calculated for $C_{20}H_{27}BrNO_6S$: 488.0737; found: 488.0733.

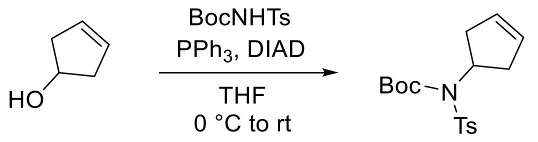

S13

To a dry flask charged with a solution of cyclopent-3-en-1-ol (1 g, 11.9 mmol, 1 eq, 0.2 M in THF), *tert*-butyl tosylcarbamate (3.55 g, 13.1 mmol, 1.1 eq) and triphenylphosphine (3.43 g, 13.1 mmol, 1.1 eq) at 0°C diethyl azodicarboxylate (2.58 mL, 2.65 g, 13.1 mmol, 1.1 eq) was added dropwise during a period of 10 min and the reaction was stirred overnight at room temperature. After 16 h the reaction mixture was quenched with 10 ml of H_2O and extracted x3 with EtOAc. The combined organic layers were then washed x1 with brine, dried over anhydrous Na_2SO_4 and evaporated under reduced pressure to afford a yellow oil. The crude was purified by column chromatography on silica gel (Cyclohexane/EtOAc 3:2). Concentration of the appropriate fractions afforded **S13** as a white solid.

Yield: 1.9 g, 47%.

Rf: 0.5 (60:40 Cyclohexane/EtOAc).

Aspect: white solid; m.p. 39.2-43.2 °C

^{1}H NMR (400 MHz, CDCl$_3$): δ (ppm) 7.77 (d, J = 8.4 Hz, 2H), 7.30 (d, J = 8.0 Hz, 2H), 5.68 (s, 2H), 5.33 – 5.24 (m, 1H), 2.73 (d, J = 1.4 Hz, 2H), 2.71 (s, 2H), 2.44 (s, 3H), 1.33 (s, 9H).

^{13}C NMR (126 MHz, CDCl$_3$): δ (ppm) 150.7, 144.0, 137.9, 129.4, 128.9, 127.7, 84.4, 55.8, 37.9, 28.1, 21.8.

IR (ATR): ν_{max} 1728, 1355, 1154, 631 cm^{-1}.

HRMS: (ESI) m/z [M + Na]$^+$ calculated for $C_{17}H_{23}NNaO_4S$: 360.1245; found: 360.1240.

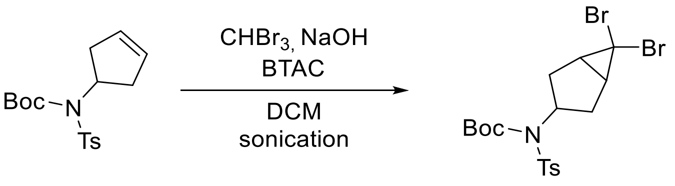

S13 **S14**

Following general procedure A: using sodium hydroxide (1.35 g, 33.8 mmol, 6 eq) in 3.1 ml of DCM, benzyltriethylammonium chloride (64 mg, 0.28 mmol, 0.05 eq), *tert*-butyl cyclopent-3-en-1-yl(tosyl)carbamate (**S13**, 1.89 g, 5.63 mmol, 1 eq) and bromoform (0.98 ml, 11.30 mmol, 2 eq). The flask was fitted to a reflux condenser and subjected to sonication for 1 h. The cooled reaction mixture was filtered through a short pad of celite and then washed with 10 ml DCM. The combined filtrates were concentrated under reduced pressure to afford a yellow oil. The crude was purified by column chromatography on silica gel (Cyclohexane/EtOAc 100:0 to 90:10). Concentration of the appropriate fractions afforded **S14** as a white solid.

Yield: 1.38 g, 68%.

Rf: 0.8 (60:40 Cyclohexane/EtOAc).

Aspect: white solid; decomposed at 142 °C

^{1}H NMR (400 MHz, $CDCl_3$): δ (ppm) 7.71 (d, *J* = 8.4 Hz, 2H), 7.30 (d, *J* = 8.0 Hz, 2H), 5.07 (tt, *J* = 8.6, 6.5 Hz, 1H), 2.44 (s, 3H), 2.43 – 2.39 (m, 2H), 2.38 – 2.27 (m, 4H), 1.34 (s, 9H).

^{13}C NMR (126 MHz, $CDCl_3$): δ (ppm) 150.5, 144.3, 137.6, 129.5, 127.7, 84.8, 59.6, 39.5, 38.3, 35.5, 28.1, 21.8.

IR (ATR): ν_{max} 1722, 1357, 1153, 660 cm^{-1}.

HRMS: (ESI) m/z [M + Na]$^+$ calculated for $C_{18}H_{23}Br_2NNaO_4S$: 529.9607; found: 529.9607.

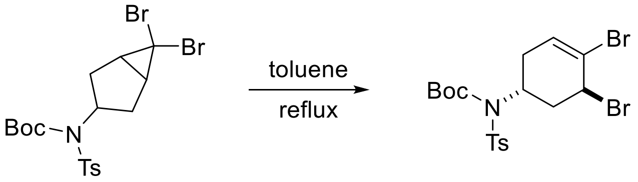

S14 **S15**

A flask charged with a solution of *tert*-butyl (6,6-dibromobicyclo[3.1.0]hexan-3-yl)(tosyl)carbamate (**S14**, 2.87 g, 5.64 mmol, 1 eq) in 28.2 ml of toluene (0.2 M) was refluxed overnight. After 16 h toluene was evaporated under reduced pressure to afford a brown solid. The crude was purified by column chromatography on silica gel (Cyclohexane/EtOAc 3:2). Concentration of the appropriate fractions afforded **S15** as an impure mixture. **S15** was used to the next step without further purification.

Rf: 0.7 (60:40 Cyclohexane/EtOAc).

IR (ATR): ν_{max} 1067, 646 cm^{-1}.

HRMS: (ESI) m/z [M + Na]$^+$ calculated for $C_{18}H_{23}Br_2NNaO_4S$: 529.9607; found: 529.9607

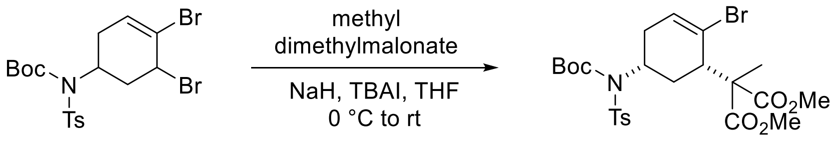

S15 **S16**

Following general procedure D: using NaH (53 mg, 1.34 mmol, 1.2 eq, 60% in mineral oil), TBAI (165 mg, 0.45 mmol, 0.4 eq) in 9 ml of THF (0.125 M) to react with methyl dimethylmalonate (196 mg, 1.34 mmol, 1.2 eq, 0.2 M in THF) and *tert*-butyl (4,5-dibromocyclohex-3-en-1-yl)(tosyl)carbamate (**S15**, 570 mg, 1.12 mmol, 1 eq, 0.5 M in THF). After 16 h the reaction mixture was quenched with 10 ml of distilled water and extracted x3 with EtOAc. The combined organic layers were then washed x2 with NaOH 1M and x1 with brine, dried over anhydrous Na_2SO_4 and evaporated under reduced pressure to afford a yellow oil. The crude was purified by column chromatography on silica gel (Cyclohexane/EtOAc 4:1 to 3:2). Concentration of the appropriate fractions afforded **S16** as an impure mixture. **S16** was used to the next step without further purification.

Rf: 0.25 (80:20 Cyclohexane/EtOAc).

IR (ATR): ν_{max} 1732, 1360, 1254, 1153, 756 cm^{-1}.

HRMS: (ESI) m/z $[M + Na]^+$ calculated for $C_{24}H_{32}BrNNaO_8S$: 596.0924; found: 596.0931

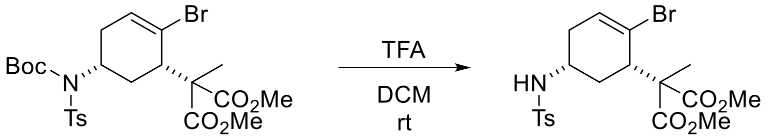

S16 **1n**

To a dry flask charged with a solution of dimethyl 2-(2-bromo-5-((N-(*tert*-butoxycarbonyl)-4-methylphenyl)sulfonamido)cyclohex-2-en-1-yl)-2-methylmalonate (**S16**, 399 mg, 0.69 mmol, 1 eq) in 6.9 ml of DCM (0.1 M) trifluoroacetic acid (0.261 ml, 3.48 mmol, 5 eq) was added dropwise. After completion the reaction mixture was quenched with 10 ml of sat. $NaHCO_3$ and extracted x3 with EtOAc. The combined organic layers were then washed x1 with brine, dried over anhydrous Na_2SO_4 and evaporated under reduced pressure to afford a yellow oil. The crude was purified by column chromatography on silica gel (Cyclohexane/EtOAc 60:40). Concentration of the appropriate fractions afforded **1n** as a white solid (single diastereomer).

Yield: 640 mg, 99%.

Rf: 0.2 (70:30 Cyclohexane/EtOAc).

Aspect: white solid; m.p. 63.2 – 66.7 °C

^{1}H NMR (500 MHz, $CDCl_3$): δ (ppm) 7.74 (d, *J* = 8.3 Hz, 2H), 7.32 (d, *J* = 8.3 Hz, 2H), 6.05 (dt, *J* = 6.8, 2.1 Hz, 1H), 4.49 (d, *J* = 7.7 Hz, 1H), 3.81 – 3.72 (m, 1H), 3.74 (s, 3H), 3.64 (s, 3H), 3.41 (dddd, *J* = 12.4, 10.9, 5.0, 3.4 Hz, 1H), 2.44 (s, 3H), 2.20 (dddt, *J* = 16.6, 7.0, 4.8, 2.2 Hz, 1H), 1.89 (dddd, *J* = 17.0, 10.7, 4.2, 2.2 Hz, 1H), 1.80 (ddt, *J* = 12.2, 6.0, 3.0 Hz, 1H), 1.40 (s, 3H), 1.36 (m, 1H).

^{13}C NMR (126 MHz, $CDCl_3$): δ (ppm) 171.1, 170.5, 143.8, 137.9, 131.8, 130.0, 127.1, 122.6, 57.5, 53.2, 48.8, 46.1, 35.0, 34.4, 21.7, 15.1.

IR (ATR): ν_{max} 1215, 753, 632 cm^{-1}.

HRMS: (ESI) m/z [M + H]$^+$ calculated for $C_{19}H_{25}BrNO_6S$: 474.0573; found: 474.0580

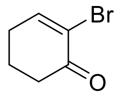

S17

S17 was synthesized according to literature.[4]

^{1}H NMR (400 MHz, CDCl$_3$): δ (ppm) 7.43 (‘t’, *J* = 4.4 Hz, 1H), 2.67 – 2.61 (m, 2H), 2.46 (td, *J* = 6.0, 4.5 Hz, 2H), 2.08 (p, *J* = 6.2 Hz, 2H).

^{13}C NMR (126 MHz, CDCl$_3$): δ (ppm) 191.4, 151.3, 124.0, 38.5, 28.5, 22.8.

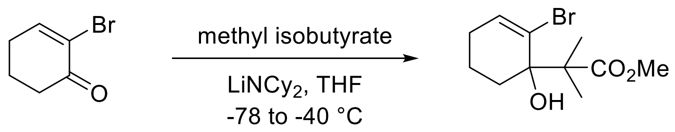

S17 **S18**

To a dry flask charged with dicyclohexylamine (400 mg, 2.2 mmol, 1.1 eq, 1 M in THF) under inert atmosphere n-BuLi (0.88 ml, 2.5 M in THF, 1.1 eq) was added at -78 °C. After 15 min a solution of methyl isobutyrate (225 mg, 1.1 eq, 0.25 M in THF) was added dropwise at -78 °C. After 15 min a solution of 2-bromocyclohex-2-en-1-one (**S17**, 350 mg, 0.5 M in THF, 1 eq) was added dropwise and the reaction mixture was left to reach -40 °C overnight. The reaction mixture was quenched with sat. NH_4Cl solution and extracted x3 with EtOAc. The combined organic layers were then washed x2 with H_2O, x1 with brine, dried over anhydrous Na_2SO_4 and evaporated under reduced pressure. The crude was purified by column chromatography on silica gel (Cyclohexane/EtOAc 100:0 to 97:3). Concentration of the appropriate fractions afforded **S18** as a colorless oil.

Yield: 237 mg, 43%.

Rf: 0.45 (80:20 Cyclohexane/EtOAc)

Aspect: colorless oil.

^{1}H NMR (500 MHz, CDCl$_3$): δ (ppm) 6.23 (ddd, *J* = 6.5, 2.4, 0.9 Hz, 1H), 6.00 (d, *J* = 1.8 Hz, 1H), 3.69 (s, 3H), 2.10 – 2.02 (m, 1H), 2.00 – 1.89 (m, 2H), 1.84 – 1.75 (m, 1H), 1.75 – 1.68 (m, 1H), 1.64 (m, 1H), 1.21 (s, 3H), 1.20 (s, 3H).

^{13}C NMR (126 MHz, CDCl$_3$): δ (ppm) 180.4, 135.2, 128.8, 77.5, 52.8, 47.4, 33.8, 28.2, 22.8, 20.4, 18.4.

IR (ATR): ν_{max} 3393, 2987, 2950, 1694, 1282, 1150 cm^{-1}.

HRMS: (ESI) m/z [M + Na]$^+$ calculated for $C_{11}H_{17}BrNaO_3$: 299.0253; found: 299.0251.

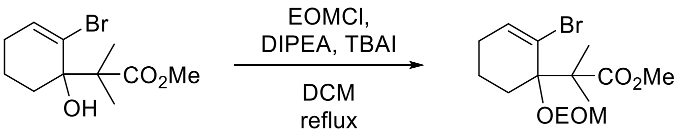

S18 **1o**

To a stirred solution of methyl 2-(2-bromo-1-hydroxycyclohex-2-en-1-yl)-2-methylpropanoate (**S18**, 237 mg, 0.85 mmol, 1 eq), N,N-diisopropylethylamine (332 mg, 2.57 mmol, 3 eq) and tetrabutylammonium iodide (63 mg, 0.17 mmol, 0.2 eq) in anhydrous DCM (0.25 M), chloromethyl ethyl ether (404 mg, 4.28 mmol, 5 eq) was added dropwise and the reaction mixture was refluxed overnight. The reaction mixture was quenched with water and extracted x3 with EtOAc. The combined organic layers were then washed x2 with water, x1 with brine, dried over anhydrous Na_2SO_4 and evaporated under reduced pressure. The crude was purified by column chromatography on silica gel (Cyclohexane/EtOAc 100:0 to 99:1). Concentration of the appropriate fractions afforded **1o** as a colorless oil.

Yield: 52 mg, 18%.

Rf: 0.55 (80:20 Cyclohexane/EtOAc)

Aspect: colorless oil.

^{1}H NMR (500 MHz, CDCl$_3$): δ (ppm) 6.61 (dd, J = 6.2, 2.9 Hz, 1H), 4.82 (d, J = 7.6 Hz, 1H), 4.77 (d, J = 7.6 Hz, 1H), 3.87 (dq, J = 9.6, 7.1 Hz, 1H), 3.67 (s, 3H), 3.55 (dq, J = 9.6, 7.1 Hz, 1H), 2.15 – 2.07 (m, 2H), 2.02 – 1.95 (m, 1H), 1.94 – 1.85 (m, 1H), 1.70 – 1.63 (m, 2H), 1.47 (s, 3H), 1.31 (s, 3H), 1.21 (t, J = 7.1 Hz, 3H).

^{13}C NMR (126 MHz, CDCl$_3$): δ (ppm) 176.8, 139.8, 124.9, 91.1, 80.0, 63.6, 52.3, 51.8, 34.5, 27.7, 22.8, 22.7, 19.6, 15.3.

IR (ATR): ν_{max} 2948, 2879, 1728, 1280, 1149, 1024, 631 cm^{-1}.

HRMS: (ESI) m/z $[M + H]^+$ calculated for $C_{14}H_{24}BrO_4$: 335.0852; found: 335.0853.

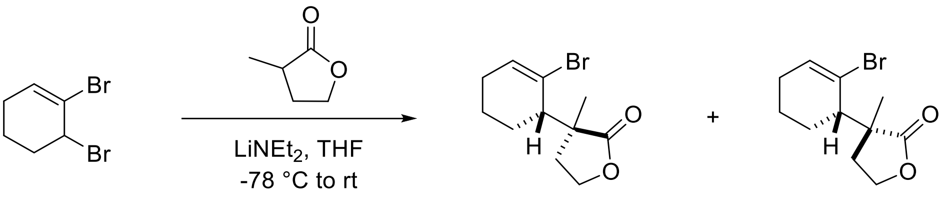

S2 **1p** major diastereomer **1p'** minor diastereomer

Following general procedure C: using diethylamine (671 mg, 9.2 mmol, 1.1 eq, 1 M in THF), n-BuLi (3.7 ml, 9.2 mmol, 1.1 eq, 2.5 M in Hexanes), 3-methyldihydrofuran-2(3H)-one (918 mg, 9.2 mmol, 1.1 eq, 0.25 M in THF) and 1,6-dibromocyclohex-1-ene (**S2**, 2 g, 8.3 mmol, 1 eq, 0.5 M in THF). After completion the reaction mixture was quenched with 10 ml of sat. NH_4Cl solution and extracted x3 with EtOAc. The combined organic layers were then washed x1 with brine, dried over anhydrous Na_2SO_4 and evaporated under reduced pressure to afford a yellow oil. The crude was purified by column chromatography on silica gel (Cyclohexane/EtOAc 97:3). Concentration of the appropriate fractions afforded the two diastereomers **1p** and **1p'**.

Yield: 1.12 g, 52% (*major diastereomer*: 783 mg, *minor diastereomer*: 337 mg)

Rf: *major diastereomer*: 0.17, *minor diastereomer*: 0.08 (90:10 Cyclohexane/EtOAc),

Aspect: *major diastereomer*: white solid; m.p. 49.2 – 52.8 °C, *minor diastereomer*: off-white solid; m.p. 116.6 – 119.6 °C

^{1}H NMR (500 MHz, $CDCl_3$): δ (ppm) *major diastereomer*: 6.36 – 6.31 (m, 1H), 4.30 ('td', *J* = 9.3, 3.7 Hz, 1H), 4.24 (td, *J* = 9.0, 7.9 Hz, 1H), 2.80 (dddt, *J* = 6.0, 4.4, 3.2, 1.7 Hz, 1H), 2.44 (dt, *J* = 13.4, 9.1 Hz, 1H), 2.19 – 2.12 (m, 2H), 2.10 – 2.00 (m, 2H), 1.74 – 1.69 (m, 1H), 1.68 (s, 3H), 1.57 – 1.46 (m, 2H). *minor diastereomer*: 6.29 (ddd, *J* = 4.9, 3.4, 1.5 Hz, 1H), 4.44 (ddd, *J* = 9.6, 9.1, 3.1 Hz, 1H), 4.29 (td, *J* = 9.1, 8.0 Hz, 1H), 3.09 (m, 1H), 2.53 – 2.43 (m, 1H), 2.14 – 2.04 (m, 2H), 2.02 – 1.95 (m, 1H), 1.84 (ddd, *J* = 12.8, 8.0, 3.1 Hz, 1H), 1.69 – 1.58 (m, 3H), 1.26 (s, 3H).

^{13}C NMR (101 MHz, $CDCl_3$): δ (ppm) *major diastereomer*: 180.9, 135.8, 122.5, 65.4, 47.4, 46.9, 31.9, 28.6, 28.0, 25.9, 20.4. *minor diastereomer*: 182.8, 134.6, 124.0, 65.2, 46.3, 45.4, 31.1, 28.0, 27.5, 23.4, 20.2.

IR (ATR): ν_{max} 2931, 1765, 1195, 1097 cm^{-1}.

HRMS: *major diastereomer*: (ESI) m/z $[M + H]^+$ calculated for $C_{11}H_{16}BrO_2$: 259.0328; found: 259.0325.

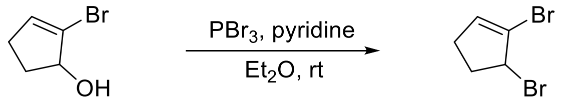

S19

2-bromocyclopent-2-en-1-ol was synthesized according to literature.[4,5] To a dry flask charged with a solution of 1,5-dibromocyclopent-1-ene (**S19**, 2 g, 12.3 mmol, 1 eq) in 50 ml of Et_2O (0.25 M), pyridine (0.146 mg, 1.84 mmol, 0.15 eq) and phosphorus tribromide (3.33 g, 12.3 mmol, 1 eq) was added dropwise. After completion the reaction mixture was quenched with 10 ml of water and extracted x3 with EtOAc. The combined organic layers were then washed x1 with brine, dried over anhydrous Na_2SO_4 and evaporated under reduced pressure to afford an orange oil. The crude was purified by column chromatography on silica gel (pure Petroleum ether). Concentration of the appropriate fractions afforded **S19** as an orange oil. ^{1}H and ^{13}C NMR data match those reported in the literature.[6]

^{1}H NMR (500 MHz, $CDCl_3$): δ (ppm) 6.17 – 6.14 (m, 1H), 4.98 – 4.93 (m, 1H), 2.57 (dddd, *J* = 12.0, 10.4, 5.4, 2.7 Hz, 2H), 2.52 – 2.43 (m, 1H), 2.36 – 2.26 (m, 1H).

^{13}C NMR (126 MHz, $CDCl_3$): δ (ppm) 137.3, 123.6, 60.9, 35.6, 31.3.

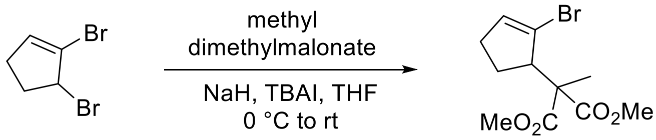

S19 **1r**

Following general procedure D: using NaH (100 mg, 2.65 mmol, 1.2 eq, 60% in mineral oil), TBAI (327 mg, 0.88 mmol, 0.4 eq) in 18 ml of THF (0.125 M) to react with methyl dimethylmalonate (388 mg, 2.65

mmol, 1.2 eq, 0.2 M in THF) and 1,5-dibromoclopent-1-ene (**S19**, 500 mg, 2.21 mmol, 1 eq, 0.5 M in THF). After 16 h the reaction mixture was quenched with 10 ml of distilled water and extracted x3 with EtOAc. The combined organic layers were then washed x2 with NaOH 1M and x1 with brine, dried over anhydrous Na_2SO_4 and evaporated under reduced pressure to afford a yellow oil. The crude was purified by column chromatography on silica gel (Cyclohexane/EtOAc 100:0 to 98:2). Concentration of the appropriate fractions afforded **1r** as a yellow oil.

Yield: 558 mg, 87%.

Rf: 0.21 (90:10 Cyclohexane/EtOAc).

Aspect: yellow oil

^{1}H NMR (400 MHz, CDCl$_3$): δ (ppm) 6.01 ('dt', *J* = 3.2, 1.6 Hz, 1H), 3.91 – 3.83 (m, 1H), 3.74 (s, 3H), 3.71 (s, 3H), 2.29 – 2.20 (m, 3H), 1.75 (m, 1H), 1.39 (s, 3H).

^{13}C NMR (126 MHz, CDCl$_3$): δ (ppm) 171.5, 171.1, 135.7, 120.5, 57.1, 53.3, 52.9, 52.8, 31.0, 27.0, 14.9.

IR (ATR): ν_{max} 2953, 1737, 1248, 1228, 650 cm^{-1}.

HRMS: (ESI) m/z [M + Na]$^+$ calculated for $C_{11}H_{15}BrNaO_4$: 313.0046; found: 313.0042.

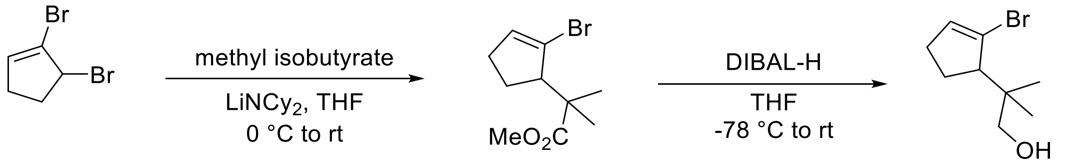

S19 **S19'** **S20**

Following general procedure B: using dicyclohexylamine (1.2 g, 6.65 mmol, 1.5 eq), n-BuLi (1.81 ml, 2.5 M in hexanes, 1.5 eq), methyl isobutyrate (543 mg, 5.32 mmol, 1.2 eq, 0.25 M in THF) and 1,5-dibromocyclopent-1-ene (**S19**, 1 g, 4.43 mmol, 1 eq, 0.5 M in THF). After 16 h the reaction mixture was quenched with 10 ml of sat. NH_4Cl solution and extracted x3 with EtOAc. The combined organic layers were then washed x1 with brine, dried over anhydrous Na_2SO_4 and evaporated under reduced pressure to afford a yellow oil. The crude was purified by column chromatography on silica gel (Cyclohexane/EtOAc 98:2). Concentration of the appropriate fractions afforded **S19'** as an impure mixture. **S19'** was used to the next step without further purification. To a dry flask charged with a solution of methyl 2-(2-bromocyclopent-2-en-1-yl)-2-methylpropanoate (**S19'**, 500 mg, 2.02 mmol, 1 eq, 0.2 M in DCM) under inert atmosphere DIBAL-H (4.45 ml, 4.45 mmol, 2.2 eq, 1 M in THF) was added dropwise at -78 °C and left to come at room temperature overnight. After 16 h the reaction mixture was quenched with 15 ml sat. Rochelle salt aq. solution and was left to stir for 1 h followed by extraction x3 with EtOAc. The combined organic layers were then washed x1 with brine, dried over anhydrous Na_2SO_4 and evaporated under reduced pressure to afford a yellow oil. The crude was purified by column chromatography on silica gel (Cyclohexane/EtOAc 95:5). Concentration of the appropriate fractions afforded **S20** as a clear colorless oil.

Yield: 280 mg, 43% over two steps.

Rf: 0.15 (90:10 Cyclohexane/EtOAc).

Aspect: colorless oil.

^{1}H NMR (400 MHz, CDCl$_3$): δ (ppm) 6.02 (tdd, *J* = 2.5, 1.8, 0.9 Hz, 1H), 3.74 (dd, *J* = 11.2, 4.4 Hz, 1H), 3.38 (dd, *J* = 11.2, 6.4 Hz, 1H), 2.85 (dddt, *J* = 8.9, 4.5, 3.1, 1.6 Hz, 1H), 2.28 – 2.20 (m, 1H), 2.20 – 2.13 (m, 1H), 2.11 – 2.04 (m, 1H), 1.95 – 1.88 (m, 1H), 1.68 – 1.63 (m, 1H), 0.97 (s, 3H), 0.94 (s, 3H).

^{13}C NMR (126 MHz, CDCl$_3$): δ (ppm) 135.2, 122.6, 70.9, 54.0, 39.6, 31.3, 26.7, 23.4, 21.1.

IR (ATR): ν_{max} 3414, 3344, 2969, 2940, 1031, 676 cm^{-1}.

HRMS: data for this compound could not be obtained due to poor ionization. Instead the GC/MS spectrum is provided among with major fragments.

GC/MS: Rt = 6.383 min

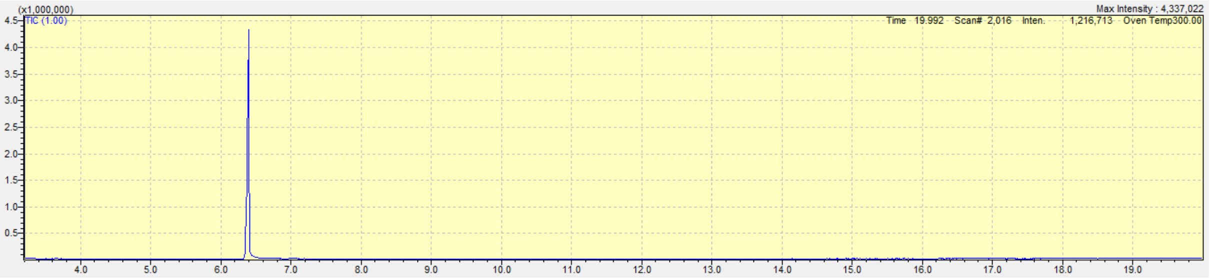

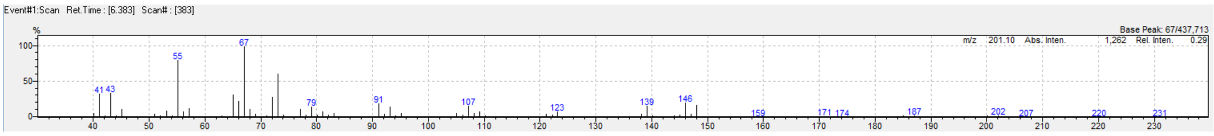

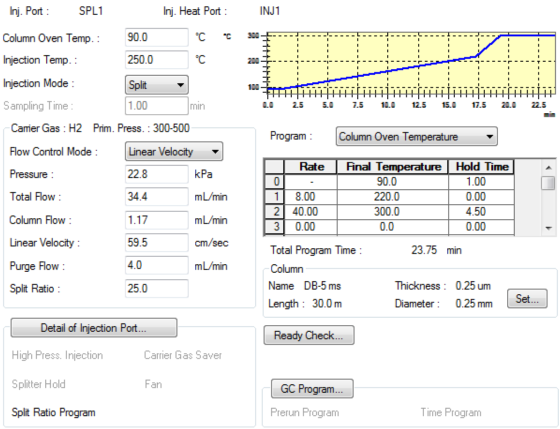

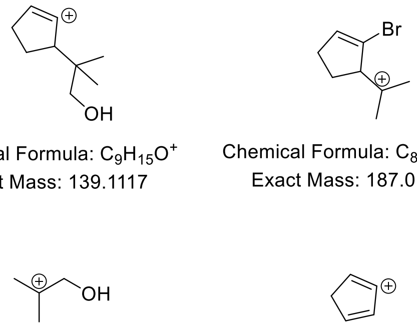

Chemical Formula: $C_9H_{15}O^+$ Exact Mass: 139.1117

Chemical Formula: $C_8H_{12}Br^+$ Exact Mass: 187.0117

Chemical Formula: $C_4H_9O^+$ Exact Mass: 73.0648

Chemical Formula: $C_5H_5^+$ Exact Mass: 65.0386

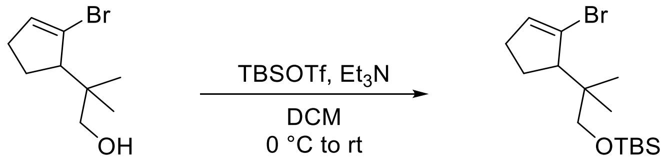

S20 **1s**

To a dry flask charged with a solution of 2-(2-bromocyclopent-2-en-1-yl)-2-methylpropan-1-ol (**S20**, 100 mg, 0.46 mmol, 1 eq, 0.2 M in DCM), Et$_3$N (0.09 ml, 71 mg, 0.69 mmol, 1.5 eq) and *tert*-butyldimethylsilyl trifluoromethanesulfonate (0.126 ml, 145 mg, 0.58 mmol, 1.2 eq) were added at 0 °C and left to reach room temperature overnight. After 16 h the reaction mixture was quenched with

15 ml of 2 M HCl solution. The combined organic layers were then washed x1 with HCl 2 M and x1 with brine, dried over anhydrous Na_2SO_4 and evaporated under reduced pressure to afford a yellow oil. The crude was purified by column chromatography on silica gel (pure Cyclohexane). Concentration of the appropriate fractions afforded **1s** as a clear colorless oil.

Yield: 148 mg, 97%.

Rf: 0.5 (pure Cyclohexane).

Aspect: colorless oil.

^{1}H NMR (500 MHz, CDCl$_3$): δ (ppm) 5.99 ('q', *J* = 2.3 Hz, 1H), 3.52 (d, *J* = 9.5 Hz, 1H), 3.42 (d, *J* = 9.6 Hz, 1H), 2.89 – 2.85 (m, 1H), 2.26 – 2.18 (m, 1H), 2.17 – 2.10 (m, 1H), 2.02 (dtdd, *J* = 12.9, 9.1, 6.2, 0.6 Hz, 1H), 1.90 (ddt, *J* = 13.3, 8.6, 4.8 Hz, 1H), 1.01 (s, 3H), 0.90 (s, 6H), 0.88 (s, 3H), 0.04 (s, 3H), 0.03 (s, 3H).

^{13}C NMR (126 MHz, CDCl$_3$): δ (ppm) 134.8, 123.5, 71.0, 54.0, 39.4, 31.1, 26.6, 26.1, 22.4, 22.3, 18.4, -5.3, -5.4.

IR (ATR): ν_{max} 2956, 2931, 2856, 1253, 1097, 852, 837, 776 cm^{-1}.

HRMS: (ESI) m/z $[M + Ag]^+$ calculated for $C_{15}H_{29}AgBrOSi$: 439.0216; found: 439.0212.

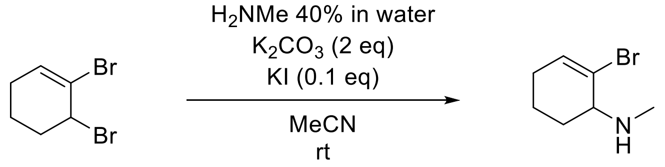

S2 **S21-*rac***

To a flask charged with a solution of 1,6-dibromocyclohex-1-ene (**S2**, 2 g, 8.34 mmol, 1 eq, 0.35 M in MeCN), methylamine (1.3 ml, 16.7 mmol, 2 eq, 40% in water), potassium carbonate (2.31 g, 16.7 mmol, 2 eq) and potassium iodide (138 mg, 0.83 mmol, 0.1 eq) were added at room temperature and left overnight. After 16 h MeCN was evaporated and the resulting oil was diluted in EtOAc. The organic phase was then washed x1 with distilled water and x1 with brine, dried over anhydrous Na_2SO_4 and evaporated under reduced pressure to afford a yellow oil. The crude was purified by column chromatography on silica gel (Cyclohexane/EtOAc 90:10 to 80:20). Concentration of the appropriate fractions afforded **S21-*rac*** as a yellow oil.

Yield: 1.2 g, 76%.

Rf: 0.25 (Cyclohexane/EtOAc 80:20).

Aspect: yellow oil.

^{1}H NMR (500 MHz, CDCl$_3$): δ (ppm) 6.18 ('td', *J* = 4.1, 1.1 Hz, 1H), 3.20 ('tq', *J* = 5.2, 1.6 Hz, 1H), 2.40 (s, 3H), 2.13 – 1.98 (m, 2H), 1.88 – 1.76 (m, 2H), 1.75 – 1.67 (m, 1H), 1.65 – 1.62 (br, 1H), 1.63 – 1.54 (m, 1H).

^{13}C NMR (126 MHz, CDCl$_3$): δ (ppm) 132.6, 126.6, 60.1, 33.3, 28.6, 28.0, 18.6.

IR (ATR): ν_{max} 2929, 1641, 1449, 644 cm^{-1}.

HRMS: (ESI) m/z $[M + H]^+$ calculated for $C_7H_{13}BrN$: 190.0226; found: 190.0229.

For the preparation of the scalemic mixture:

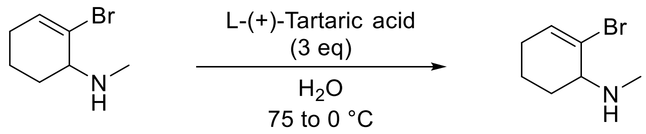

S21-*rac*

S21
e.r. 73:27

To a flask charged with 2-bromo-N-methylcyclohex-2-en-1-amine (**S21-*rac***, 800 mg, 4.21 mmol, 1 eq) and 10 ml of water, L-(+)-tartaric acid (632 mg, 4.21 mmol, 1 eq) was added and the mixture was stirred at 75 °C until complete dissolution occurred. While the mixture was still warm more L-(+)-tartaric acid (1.2 g, 8.42 mmol, 2 eq) was added and was slowly dissolved upon moderate heating. The saturated solution was first left to slowly cool down until rt and then placed in an ice bath. After 4h the white crystalline white precipitate was filtered off and the yellow filtrate was treated with 1M NaOH. The aqueous phase was extracted x4 with EtOAc. The combined organic phases were washed x1 with 1M NaOH, x1 with distilled water and x1 with brine, dried over anhydrous Na_2SO_4 and evaporated under reduced pressure to afford the enantioenriched amine **S21** as a yellow oil. The ^{1}H and ^{13}C spectra matched those of **S21-*rac***. The enantiomeric ratio was determined through chiral GC-FID.

Yield: 280 mg, 35%.

$[\alpha]_D^{20}$ = 12.3 (c = 1, $CDCl_3$).

e.r. = 73:27; HYDRODEX β-6TBDM column (25 m x 0.25 mm ID) – H_2 as carrier gas

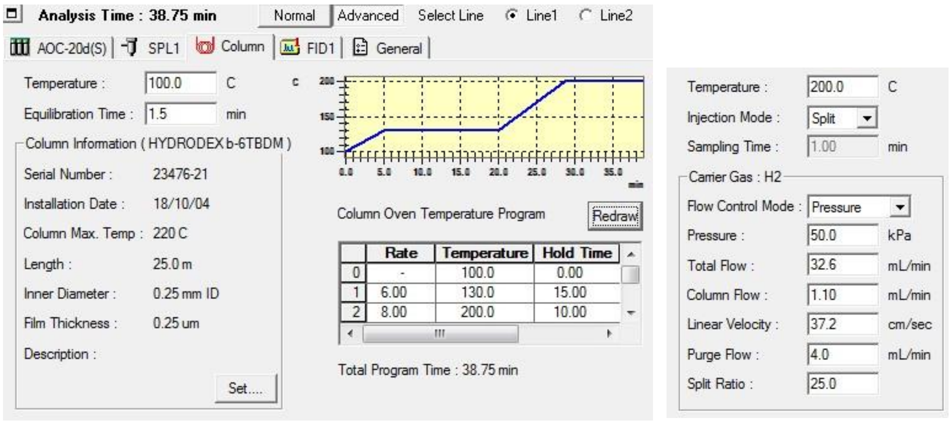

Spectrum of racemic mixture (**S21-*rac***):

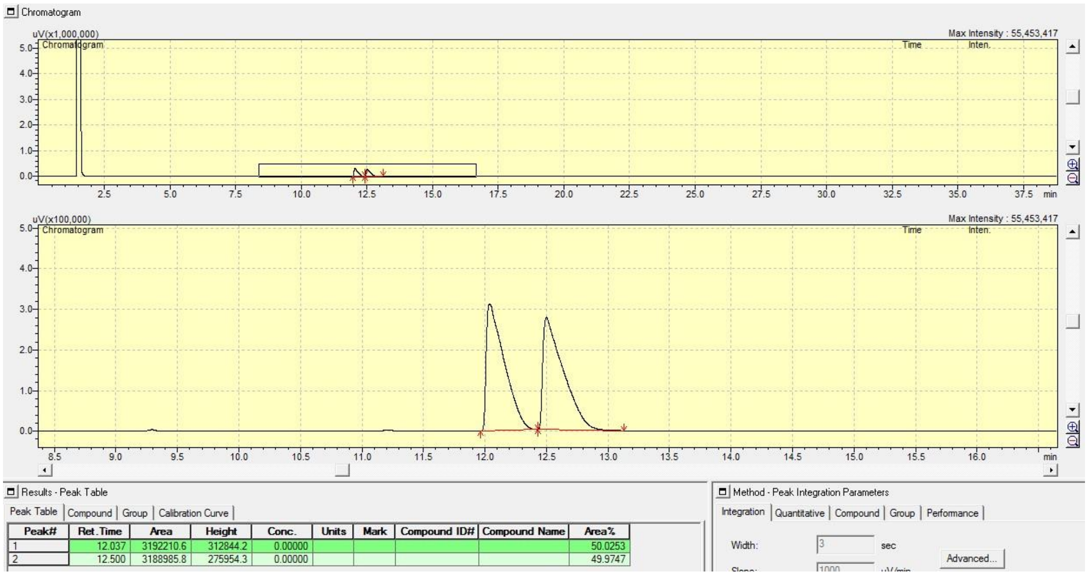

Peak#	Ret.Time	Area	Height	Conc.	Units	Mark	Compound ID#	Compound Name	Area%
1	12.037	3192210.6	312844.2	0.00000					50.0253
2	12.500	3188985.8	275954.3	0.00000					49.9747

Spectrum of scalemic mixture (**S21**):

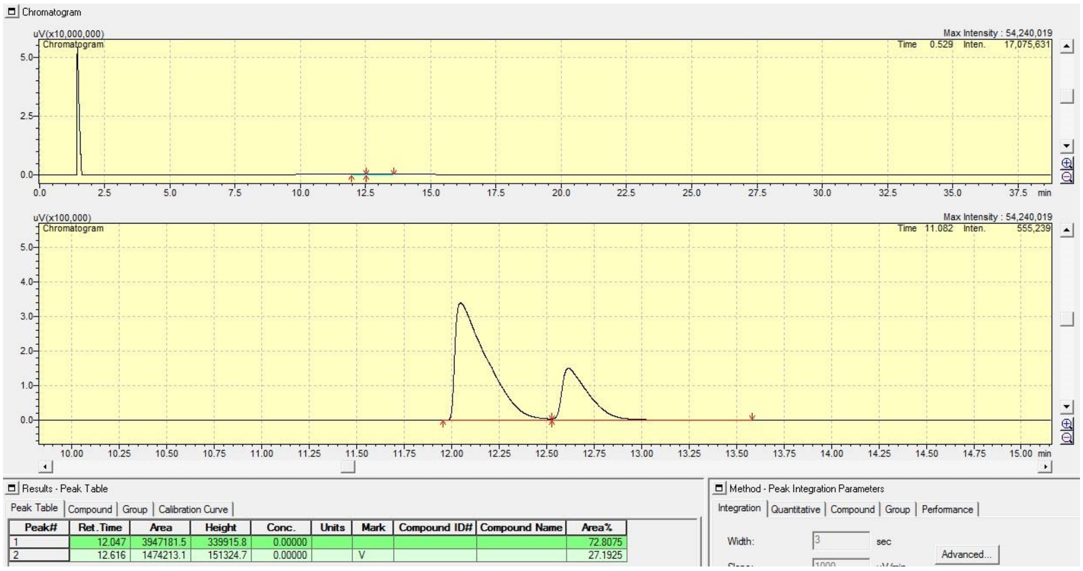

Peak#	Ret.Time	Area	Height	Conc.	Units	Mark	Compound ID#	Compound Name	Area%
1	12.047	3947181.5	339915.8	0.00000					72.8075
2	12.616	1474213.1	151324.7	0.00000		V			27.1925

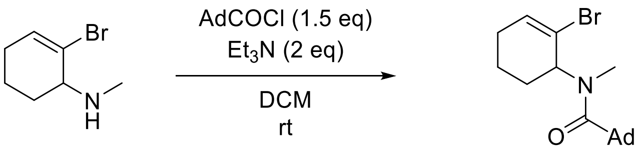

S21-*rac* **4a-*rac***

To a flask charged with a solution of 2-bromo-N-methylcyclohex-2-en-1-amine (**S21-*rac***, 210 mg, 1.10 mmol, 1 eq, 0.15 M in DCM), adamantane-1-carbonyl chloride (329 mg, 1.66 mmol, 1.5 eq) and triethylamine (0.3 ml, 2.21 mmol, 2 eq) were added at room temperature and left overnight. After 16 h the reaction mixture was washed x2 with 1 M HCl, x1 with distilled water and x1 with brine, dried over anhydrous Na_2SO_4 and evaporated under reduced pressure to afford a yellow oil. The crude was purified by column chromatography on silica gel (Cyclohexane/EtOAc 90:10 to 80:20). Concentration of the appropriate fractions afforded **4a-*rac*** as a yellow oil.

Yield: 216 mg, 56%.

Rf: 0.28 (Cyclohexane/EtOAc 90:10).

Aspect: yellow oil.

^{1}H NMR (400 MHz, $CDCl_3$): δ (ppm) 6.33 ('dt', J = 5.2, 2.5 Hz, 1H), 5.25 (br s, 1H), 2.90 (br s, 3H), 2.13 – 2.07 (m, 2H), 2.07 – 2.00 (m, 8H), 2.00 – 1.95 (m, 2H), 1.84 – 1.75 (m, 1H), 1.75 – 1.64 (m, 8H).

^{13}C NMR (126 MHz, $CDCl_3$): δ (ppm) 177.2, 134.7, 124.0, 57.2, 42.5, 39.2, 36.8, 31.4, 28.7, 27.6, 21.2.

IR (ATR): ν_{max} 2904, 2850, 1620, 1380, 1298, 1051, 627 cm^{-1}.

HRMS: (ESI) m/z $[M + H]^+$ calculated for $C_{18}H_{27}BrNO$: 352.1271; found: 352.1270.

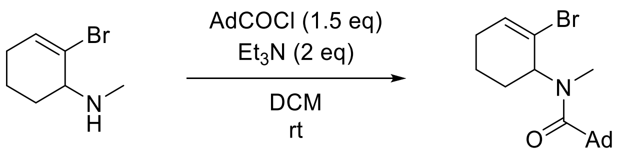

S21
e.r. 73:27

4a
e.r. 73:27

To a flask charged with a solution of 2-bromo-N-methylcyclohex-2-en-1-amine (**S21**, 250 mg, 1.30 mmol, 1 eq, 0.15 M in DCM), adamantane-1-carbonyl chloride (390 mg, 1.97 mmol, 1.5 eq) and triethylamine (0.37 ml, 2.61 mmol, 2 eq) were added at room temperature and left overnight. After 16 h the reaction mixture was washed x2 with 1 M HCl, x1 with distilled water and x1 with brine, dried over anhydrous Na_2SO_4 and evaporated under reduced pressure to afford a yellow oil. The crude was purified by column chromatography on silica gel (Cyclohexane/EtOAc 90:10 to 80:20). Concentration of the appropriate fractions afforded **4a** as a yellow oil. The ^{1}H and ^{13}C spectra matched those of ***4a-rac***.

Yield: 380 mg, 82%.

$[\alpha]_D^{20}$ = 1.7 (c = 1, $CDCl_3$).

e.r. = 73:27. NOTE: at this stage the enantiomeric excess couldn't be determined by chiral HPLC (no UV absorption) or chiral GC (high molecular weight). However, during the amide formation there should be no change in the enantiomeric ratio.

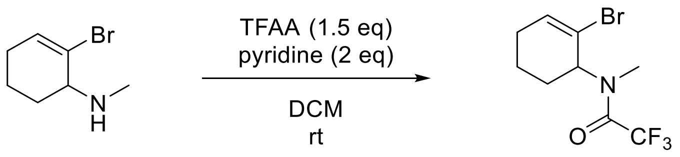

S21-*rac*

4b

To a flask charged with a solution of 2-bromo-N-methylcyclohex-2-en-1-amine (**S21-*rac***, 520 mg, 2.74 mmol, 1 eq, 0.2 M in DCM), trifluoroacetic anhydride (0.57 ml, 4.11 mmol, 1.5 eq) and pyridine (0.44 ml, 5.48 mmol, 2 eq) were added at room temperature and left overnight. After 16 h the reaction mixture was washed x2 with 1M HCl, x1 with distilled water and x1 with brine, dried over anhydrous Na_2SO_4 and evaporated under reduced pressure to afford a yellow oil. The crude was purified by

column chromatography on silica gel (Cyclohexane/EtOAc 90:10). Concentration of the appropriate fractions afforded **4b** as a yellow oil (mixture of two conformers).

Yield: 470 mg, 60%.

Rf: 0.23 (Cyclohexane/EtOAc 95:5).

Aspect: yellow oil.

^{1}H NMR (250 MHz, CDCl$_3$): *mixture of conformers*: δ (ppm) 6.45 (ddd, *J* = 5.1, 3.3, 1.9 Hz, 1H), 5.25 (d, *J* = 9.4 Hz, 0.66H, *major*), 4.61 (ddt, *J* = 9.2, 6.5, 2.9 Hz, 0.34H, *minor*), 3.01 (q, *J* = 1.8 Hz, 2H, *major*), 2.88 (d, *J* = 0.9 Hz, 1H, *minor*), 2.21 – 2.09 (m, 2H), 2.08 – 1.99 (m, 1H), 1.91 – 1.82 (m, 1H), 1.81 – 1.71 (m, 2H).

^{13}C NMR (126 MHz, CDCl$_3$): δ (ppm) *major conformer*: 157.60 (q, *J* = 35.6 Hz), 136.59, 120.41, 116.60 (q, *J* = 288.1 Hz), 58.91 (q, *J* = 3.4 Hz), 29.75, 27.54, 27.37, 20.50. *minor conformer*: 157.42 (q, *J* = 35.6 Hz), 136.56, 120.50, 116.65 (q, *J* = 288.1 Hz), 56.97, 29.94, 29.34, 27.26, 20.77.

^{19}F {^{1}H} NMR (376 MHz, CDCl$_3$): δ (ppm) -67.8 (*minor conformer*), -70.0 (*major conformer*).

IR (ATR): ν_{max} 2945, 1694, 1244, 1187, 1141, 1083 cm^{-1}.

HRMS: (ESI) m/z [M + Na]$^+$ calculated for $C_9H_{11}BrF_3NNaO$: 307.9868; found: 307.9865.

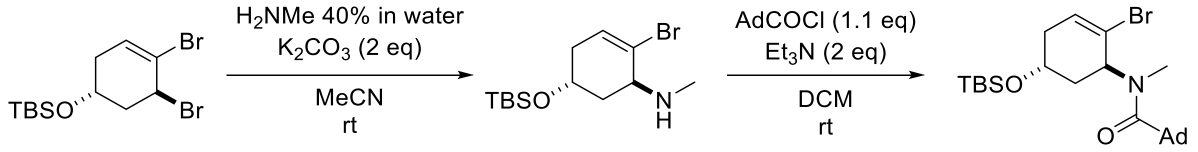

d.r. 10:1
S9

S22
single diastereomer

4c
single diastereomer

To a flask charged with a solution of tert-butyl((4,5-dibromocyclohex-3-en-1-yl)oxy)dimethylsilane (**S9**, 188 g, 0.508 mmol, 1 eq, 0.35 M in MeCN), methylamine (0.13 ml, 1.52 mmol, 3 eq, 40% in water) and potassium carbonate (140 mg, 1.02 mmol, 2 eq) were added at room temperature and left overnight. After 16 h MeCN was evaporated and the resulting oil was subjected to the next step. The crude was diluted in DCM (0.15 M) and adamantane-1-carbonyl chloride (111 mg, 0.56 mmol, 1.1 eq) and triethylamine (0.14 ml, 1.02 mmol, 2 eq) were added at room temperature and left overnight. After 16 h the reaction mixture was washed x2 with 1M HCl, x1 with distilled water and x1 with brine, dried over anhydrous Na_2SO_4 and evaporated under reduced pressure to afford a yellow oil. The crude was purified by column chromatography on silica gel (Cyclohexane/EtOAc 90:10 to 80:20). Concentration of the appropriate fractions afforded **4c** as a white solid (single diastereomer).

Yield: 165 mg, 67.2% (over two steps).

Rf: 0.13 (Cyclohexane/EtOAc 90:10).

Aspect: white solid; m.p. 114.2 – 116.5 °C

^{1}H NMR (500 MHz, CDCl$_3$): δ (ppm) 6.18 (s, 1H), 5.39 (br, 1H), 4.24 (br, 1H), 2.81 (br, 3H), 2.35 (dq, J = 17.9, 3.4 Hz, 1H), 2.11 – 2.00 (m, 11H), 1.87 (br, 1H), 1.71 (d, J = 3.0 Hz, 6H), 0.89 (d, J = 1.4 Hz, 9H), 0.06 (d, J = 1.5 Hz, 6H).

^{13}C NMR (126 MHz, CDCl$_3$): δ (ppm) 177.3, 130.5, 123.2, 65.6, 54.9, 42.4, 39.5, 36.7, 36.3, 31.5, 28.7, 26.0, 18.4, -4.6, -4.7.

IR (ATR): ν_{max} 2930, 2905, 2853, 1626, 1378, 1249, 1080, 835, 777 cm^{-1}.

HRMS: (ESI) m/z [M + H]$^+$ calculated for $C_{24}H_{41}BrNO_2Si$: 482.2084; found: 482.2085.

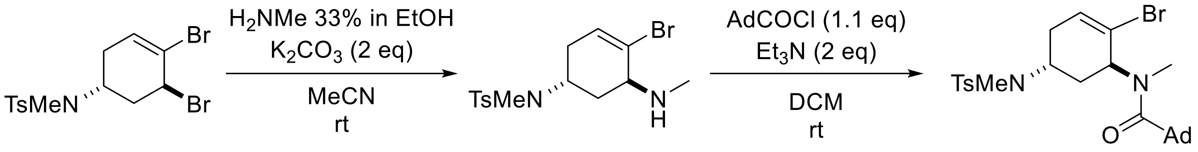

S12 **S23** single diastereomer **4d** single diastereomer

To a flask charged with a solution of N-(4,5-dibromocyclohex-3-en-1-yl)-N,4-dimethylbenzenesulfonamide (**S12**, 400 mg, 0.945 mmol, 1 eq, 0.35 M in MeCN), methylamine (0.34 ml, 2.84 mmol, 3 eq, 33% in abs. ethanol) and potassium carbonate (261 mg, 1.9 mmol, 2 eq) were added at room temperature and left overnight. After 16 h MeCN was evaporated and the resulting oil was subjected to the next step. The crude was diluted in DCM (0.15 M) and adamantane-1-carbonyl chloride (205 mg, 1.03 mmol, 1.1 eq) and triethylamine (0.26 ml, 1.88 mmol, 2 eq) were added at room temperature and left overnight. After 16 h the reaction mixture was washed x2 with 1M HCl, x1 with distilled water and x1 with brine, dried over anhydrous Na_2SO_4 and evaporated under reduced pressure to afford a yellow oil. The crude was purified by column chromatography on silica gel (Cyclohexane/EtOAc 90:10 to 80:20). Concentration of the appropriate fractions afforded **4d** as a yellow oil (single diastereomer).

Yield: 290 mg, 58% (over two steps).

Rf: 0.23 (Cyclohexane/EtOAc 80:20).

Aspect: white solid; m.p. 171.5 – 173.4 °C

^{1}H NMR (500 MHz, CDCl$_3$): δ (ppm) 7.64 (d, *J* = 8.3 Hz, 2H), 7.28 (d, *J* = 8.3 Hz, 2H), 6.29 (ddd, *J* = 5.8, 2.7, 1.3 Hz, 1H), 5.33 – 5.26 (br, 1H), 4.34 – 4.22 (m, 1H), 3.13 (s, 3H), 2.73 (s, 3H), 2.42 (s, 3H), 2.27 – 2.20 (m, 1H), 2.14 (ddt, *J* = 17.4, 10.2, 2.6 Hz, 1H), 2.09 – 2.04 (m, 4H), 2.03 – 2.02 (m, 3H), 2.01 – 1.95 (m, 3H), 1.73 (dd, *J* = 11.6, 8.2 Hz, 6H), 1.53 (ddt, *J* = 13.7, 3.7, 1.8 Hz, 1H).

^{13}C NMR (126 MHz, CDCl$_3$): δ (ppm) 177.0, 143.6, 136.8, 132.7, 130.0, 127.0, 121.7, 56.2, 49.0, 42.5, 38.9, 36.7, 33.8, 33.1, 30.9, 28.6, 28.4, 21.6.

IR (ATR): ν_{max} 2904, 2851, 1618, 1452, 1337, 1159, 663 cm^{-1}.

HRMS: (ESI) m/z [M + H]$^+$ calculated for $C_{26}H_{36}BrN_2O_3S$: 535.1625; found: 535.1621.

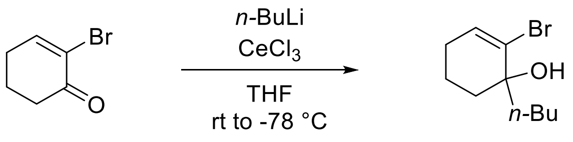

S17 **S24**

A flask charged with a solution of anhydrous $CeCl_3$ (915 mg, 3.7 mmol, 1.3 eq) in THF (8.6 ml, 0.43 M) was stirred at room temperature (suspension) under Ar. After 2 h the flask was placed at -78 °C and *n*-BuLi (1.37 ml, 3.43 mmol, 1.2 eq, 2.5 M in hexanes) was added dropwise. After stirring for 1.5 h at -78 °C a solution of 2-bromocyclohex-2-en-1-one (**S17**, 500 mg, 2.86 mmol, 1 eq) in THF (14.3 ml, 0.2 M) was added dropwise and left overnight. After 16 h the reaction mixture was quenched at -78 °C with 10 ml of sat. NH_4Cl solution and extracted x3 with EtOAc. The combined organic layers were filtered through celite® and then washed x1 with brine, dried over anhydrous Na_2SO_4 and evaporated under reduced pressure to afford a dark yellow oil. The crude was purified by column chromatography on silica gel (Cyclohexane/EtOAc 98:2). Concentration of the appropriate fractions afforded **S24** as a colorless oil.

Yield: 260 mg, 39%.

Rf: 0.27 (Cyclohexane/EtOAc 90:10).

Aspect: colorless oil

^{1}H NMR (500 MHz, CDCl$_3$): δ (ppm) 6.20 (dd, *J* = 5.1, 3.2 Hz, 1H), 2.11 (dqd, *J* = 17.8, 5.1, 1.0 Hz, 1H), 2.01 (dddd, *J* = 17.6, 8.6, 5.5, 3.3 Hz, 1H), 1.97 – 1.88 (m, 1H), 1.91 (s, 1H), 1.89 – 1.82 (m, 1H), 1.78 – 1.71 (m, 1H), 1.71 – 1.62 (m, 3H), 1.37 – 1.31 (m, 2H), 1.31 – 1.22 (m, 2H), 0.92 (t, *J* = 7.1 Hz, 3H).

^{13}C NMR (126 MHz, CDCl$_3$): δ (ppm) 132.9, 131.5, 73.5, 41.0, 35.0, 28.2, 26.2, 23.2, 19.0, 14.2.

IR (ATR): ν_{max} 2956, 2938, 1457, 973, 629 cm^{-1}.

HRMS: (ESI) m/z $[M + H]^+$ calculated for $C_{10}H_{17}AgBrO$: 338.9508; found: 338.9503.

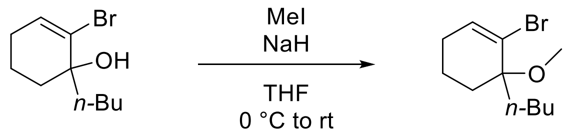

S24 **4e**

To a dry flask charged with a solution of NaH (67 mg, 1.67 mmol, 1.5 eq, 60% dispension in oil) in THF (1.35 ml, 1.24 M) a solution of 2-bromo-1-butylcyclohex-2-en-1-ol (**S24**, 260 mg, 1.12 mmol, 1 eq) in THF (2.8 ml, 0.4 M) was added dropwise at -10 °C. After 30 min iodomethane (0.1 ml, 1.67 mmol, 1.5 eq) was added dropwise and the flask was left at rt overnight. After 16 h the reaction mixture was quenched with with 10 ml of distilled water and extracted x3 with EtOAc. The combined organic layers were washed x1 with distilled water and x1 with brine, dried over anhydrous Na_2SO_4 and evaporated under reduced pressure to afford a dark yellow oil. The crude was purified by column chromatography on silica gel (Cyclohexane/EtOAc 100:0 to 99:1). Concentration of the appropriate fractions afforded **4e** as a yellow oil.

Yield: 187 mg, 68%.

Rf: 0.50 (Cyclohexane/EtOAc 90:10).

Aspect: yellow oil

^{1}H NMR (500 MHz, CDCl$_3$): δ (ppm) 6.38 (‘t’, *J* = 4.2 Hz, 1H), 3.24 (s, 3H), 2.15 – 2.07 (m, 1H), 2.04 – 1.95 (m, 2H), 1.83 (dddd, *J* = 13.7, 8.7, 3.8, 0.9 Hz, 1H), 1.78 – 1.71 (m, 1H), 1.70 – 1.63 (m, 3H), 1.37 – 1.28 (m, 3H), 1.27 – 1.20 (m, 1H), 0.90 (t, *J* = 7.1 Hz, 3H).

^{13}C NMR (126 MHz, CDCl$_3$): δ (ppm) 135.6, 129.4, 78.2, 50.9, 39.1, 30.5, 28.0, 26.0, 23.3, 20.2, 14.2.

IR (ATR): ν_{max} 2905, 2853, 1624, 1389, 1091, 874, 837 cm^{-1}.

HRMS: (ESI) m/z [M + H]$^+$ calculated for $C_{11}H_{19}AgBrO$: 352.9665; found: 352.9662.

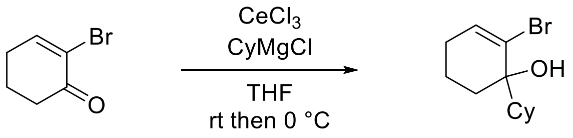

S17 **S25**

To a dry flask charged with a solution of anhydrous $CeCl_3$ (549 mg, 2.23 mmol, 1.3 eq) in THF (0.33 M, 5.2 mL) 2-bromocyclohex-2-en-1-one (**S17**, 300 mg, 1.71 mmol, 1 eq) was added neat and the resulting mixture was stirred for 1 h at rt. Then the reaction mixture was cooled to 0 °C and cyclohexylmagnesium chloride (1.2 ml, 2.4 mmol, 1.4 eq, 2 M solution in THF) was added dropwise and the reaction mixture was allowed to stir at rt overnight. After 16 h the reaction mixture was quenched with 5 ml of NH_4Cl and extracted x3 with EtOAc. The combined organic layers were washed x1 with distilled water and x1 with brine, dried over anhydrous Na_2SO_4 and evaporated under reduced pressure to afford a yellow oil. The crude was purified by column chromatography on silica gel (Cyclohexane/EtOAc 100:0 to 98:2). Concentration of the appropriate fractions afforded **S25** as a colorless oil.

Yield: 202 mg, 46%.

Rf: 0.25 (Cyclohexane/EtOAc 95:5).

Aspect: colorless oil

^{1}H NMR (400 MHz, CDCl$_3$): δ (ppm) 6.26 (dd, J = 6.2, 2.5 Hz, 1H), 2.13 – 2.03 (m, 1H), 2.01 – 1.89 (m, 2H), 1.89 – 1.82 (m, 2H), 1.81 – 1.78 (m, 1H), 1.77 – 1.62 (m, 6H), 1.60 – 1.52 (m, 1H), 1.29 – 1.19 (m, 2H), 1.17 – 0.97 (m, 2H), 0.92 – 0.82 (m, 1H).

^{13}C NMR (126 MHz, CDCl$_3$): δ (ppm) 134.04, 131.97, 75.32, 47.61, 31.40, 28.67, 28.23, 26.85, 26.78, 26.75, 25.76, 18.62.

IR (ATR): ν_{max} 2930, 2853, 1450, 975 cm^{-1}.

HRMS: (ESI) m/z [M + Na]$^+$ calculated for $C_{12}H_{19}BrNaO$: 281.0511; found: 281.0514

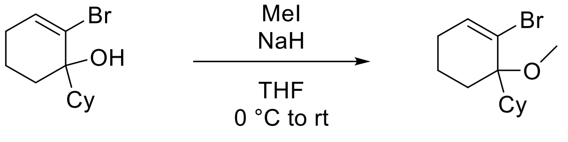

S25 **4f**

To a dry flask charged with a solution of NaH (47 mg, 1.17 mmol, 1.5 eq, 60% dispension in oil) in THF (0.63 ml, 1.24 M) a solution of 2-bromo-[1,1'-bi(cyclohexan)]-2-en-1-ol (**S25**, 200 mg, 0.78 mmol, 1 eq) in THF (0.55 ml, 0.4 M) was added dropwise at -10 °C. After 30 min iodomethane (0.07 ml, 1.17 mmol, 1.5 eq) was added dropwise and the flask was left at rt overnight. After 16 h the reaction mixture was

quenched with 5 ml of distilled water and extracted x3 with EtOAc. The combined organic layers were washed x1 with distilled water and x1 with brine, dried over anhydrous Na_2SO_4 and evaporated under reduced pressure to afford a yellow oil. The crude was purified by column chromatography on silica gel (Cyclohexane/EtOAc 100:0 to 99:1). Concentration of the appropriate fractions afforded **4f** as a yellow oil.

Yield: 96 mg, 45%.

Rf: 0.43 (Cyclohexane/EtOAc 95:5).

Aspect: yellow oil

^{1}H NMR (400 MHz, CDCl$_3$): δ (ppm) 6.54 (dd, *J* = 5.9, 2.9 Hz, 1H), 3.28 (s, 3H), 2.15 – 2.07 (m, 1H), 1.97 – 1.88 (m, 2H), 1.88 – 1.81 (m, 2H), 1.81 – 1.77 (m, 1H), 1.77 – 1.61 (m, 5H), 1.62 – 1.51 (m, 1H), 1.34 – 1.20 (m, 2H), 1.11 (tt, *J* = 13.0, 3.5 Hz, 1H), 0.99 (qd, *J* = 12.5, 3.5 Hz, 1H), 0.93 – 0.78 (m, 1H).

^{13}C NMR (126 MHz, CDCl$_3$): δ (ppm) 137.2, 129.2, 79.4, 51.8, 46.3, 29.5, 28.4, 27.7, 27.0, 26.9, 26.7, 25.8, 20.7.

IR (ATR): ν_{max} 2926, 2853, 1450, 1096, 633 cm^{-1}.

HRMS: data for this compound could not be obtained due to poor ionization. Instead the GC/MS spectrum is provided among with major fragments.

GC/MS: Rt = 11.242 min

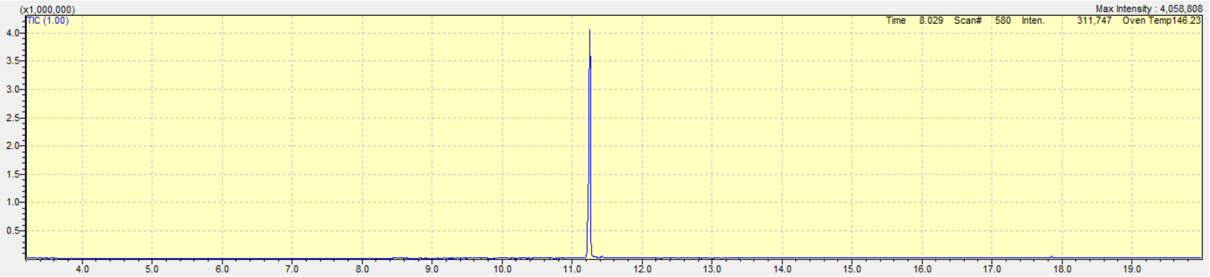

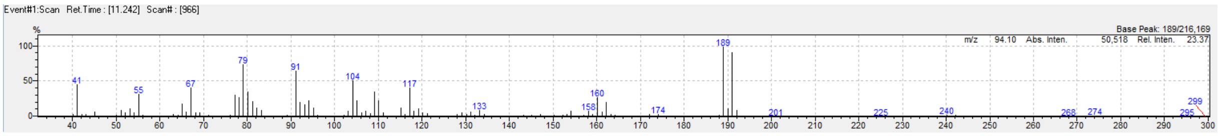

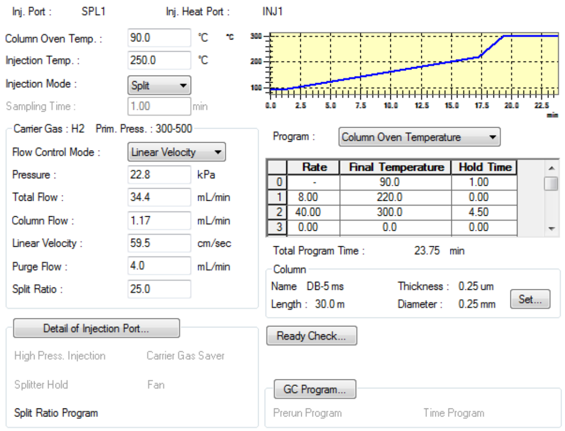

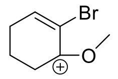

Chemical Formula: $C_7H_{10}BrO^+$
Exact Mass: 188.9910

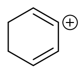

Chemical Formula: $C_6H_7^+$
Exact Mass: 79.0542

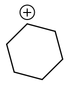

Chemical Formula: $C_6H_{11}^+$
Exact Mass: 83.0855

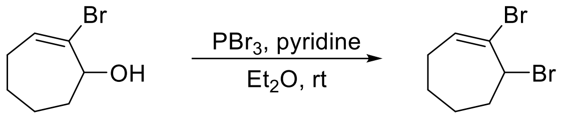

S26

2-bromocyclohept-2-en-1-ol was synthesized according to literature.[7] To a dry flask charged with a solution of 2-bromocyclohept-2-en-1-ol (2.18 g, 11.4 mmol, 1 eq) in 46 ml of Et_2O (0.25 M), pyridine (0.135 mg, 1.71 mmol, 0.15 eq) and phosphorus tribromide (3.09 g, 11.4 mmol, 1 eq) were added dropwise. After completion the reaction mixture was quenched with 10 ml of water and extracted x3 with EtOAc. The combined organic layers were then washed x1 with brine, dried over anhydrous Na_2SO_4 and evaporated under reduced pressure to afford an orange oil. The crude was purified by column chromatography on silica gel (pure Cyclohexane). Concentration of the appropriate fractions afforded **S26** as a yellow oil.

Yield: 2.46 g, 85%.

Rf: 0.42 (pure Cyclohexane)

Aspect: yellow oil.

^{1}H NMR (500 MHz, CDCl$_3$): δ (ppm) 6.33 (ddt, *J* = 6.9, 5.9, 0.9 Hz, 1H), 4.98 (‘t’, *J* = 3.6 Hz, 1H), 2.29 – 2.14 (m, 2H), 2.13 – 2.03 (m, 3H), 1.99 – 1.91 (m, 1H), 1.88 – 1.79 (m, 1H), 1.53 – 1.47 (m, 1H).

^{13}C NMR (126 MHz, CDCl$_3$): δ (ppm) 138.8, 124.6, 60.1, 34.9, 29.2, 25.8, 25.6.

IR (ATR): ν_{max} 2930, 2858, 1632, 1186, 637 cm^{-1}.

HRMS: data for this compound could not be obtained due to poor ionization. Instead the GC/MS spectrum is provided among with major fragments.

GC/MS: Rt = 6.192 min

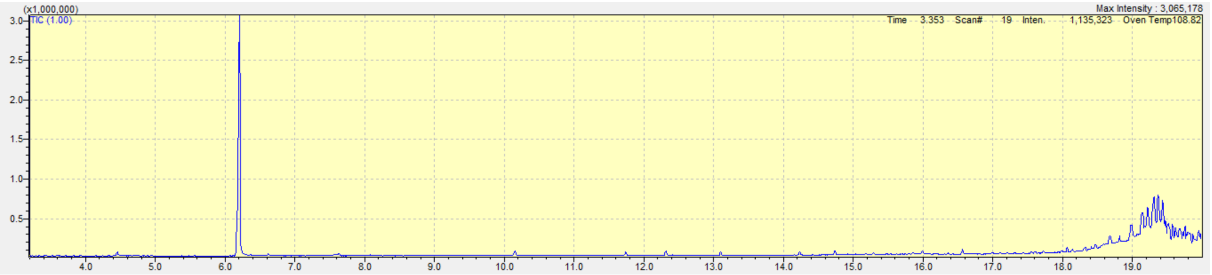

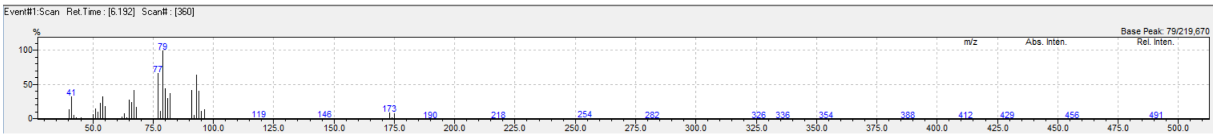

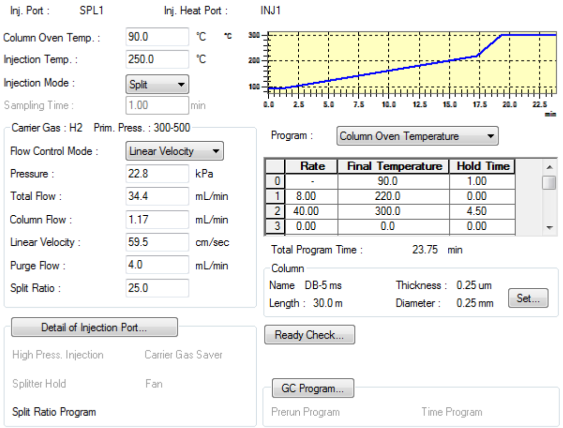

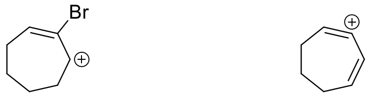

Chemical Formula: $C_7H_{10}Br^+$
Exact Mass: 172.9960

Chemical Formula: $C_7H_9^+$
Exact Mass: 93.0699

Br

Exact Mass: 78.9183

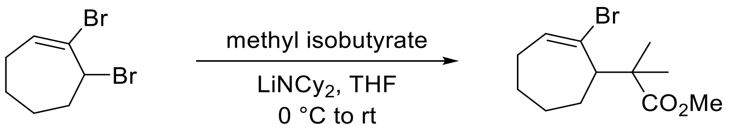

S26 **1q**

Following general procedure B: using dicyclohexylamine (1.32 g, 7.29 mmol, 1.85 eq), n-BuLi (2.92 ml, 2.5 M in THF, 1.85 eq), methyl isobutyrate (744 mg, 7.29 mmol, 1.85 eq, 0.25 M in THF) and 1,7-dibromocyclohept-1-ene (**S26**, 1 g, 3.94 mmol, 0.5 M in THF). After 16 h the reaction mixture was quenched with 10 ml of sat. NH_4Cl solution and extracted x3 with EtOAc. The combined organic layers were then washed x1 with brine, dried over anhydrous Na_2SO_4 and evaporated under reduced pressure to afford a yellow oil. The crude was purified by column chromatography on silica gel (Cyclohexane/EtOAc 100:0 to 98:2). Concentration of the appropriate fractions afforded **1q** as a light yellow oil.

Yield: 610 mg, 56.3%.

Rf: 0.25 (90:10 Cyclohexane/EtOAc)

Aspect: light yellow oil.

^{1}H NMR (400 MHz, $CDCl_3$): δ (ppm) 6.26 (ddd, J = 8.9, 7.0, 0.8 Hz, 1H), 3.67 (s, 3H), 3.17 – 3.09 (m, 1H), 2.30 – 2.17 (m, 1H), 1.92 (ddt, J = 14.8, 8.6, 4.2 Hz, 1H), 1.82 (ddd, J = 9.5, 7.3, 4.6 Hz, 1H), 1.70 – 1.63 (m, 1H), 1.62 – 1.54 (m, 2H), 1.54 – 1.44 (m, 2H), 1.33 (s, 3H), 1.23 (s, 3H).

^{13}C NMR (126 MHz, $CDCl_3$): δ (ppm) 178.1, 134.0, 126.1, 55.8, 52.0, 46.7, 25.73, 25.68, 24.6, 24.13, 24.10, 22.6.

IR (ATR): ν_{max} 2945, 2865, 1735, 1464, 1252, 662 cm^{-1}.

HRMS: (ESI) m/z $[M + H]^+$ calculated for $C_{12}H_{20}BrO_2$: 275.0641; found: 275.0639.

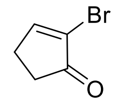

S27

S27 was synthesized according to literature.[3]

^{1}H NMR (400 MHz, CDCl$_3$): δ (ppm) 7.77 ('t', J = 3.0 Hz, 1H), 2.69 (ddd, J = 7.3, 3.0, 1.9 Hz, 2H), 2.54 – 2.48 (m, 2H).

^{13}C NMR (126 MHz, CDCl$_3$): δ (ppm) 201.8, 161.9, 126.4, 32.5, 28.1.

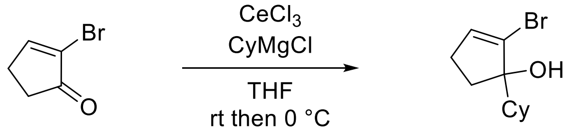

S27 **S28**

To a dry flask charged with a solution of anhydrous $CeCl_3$ (995 mg, 4.04 mmol, 1.3 eq) in THF (0.33 M, 9.4 mL) 2-bromocyclopent-2-en-1-one (**S27**, 500 mg, 3.11 mmol, 1 eq) was added neat and the resulting mixture was stirred for 1h at rt. Then the reaction mixture was cooled to 0 °C and cyclohexylmagnesium chloride (2.17 ml, 4.35 mmol, 1.4 eq, 2 M solution in THF) was added dropwise and the reaction mixture was allowed to stir at rt overnight. After 16 h the reaction mixture was quenched with 10 ml of NH_4Cl and extracted x3 with EtOAc. The combined organic layers were washed x1 with distilled water and x1 with brine, dried over anhydrous Na_2SO_4 and evaporated under reduced pressure to afford a yellow oil. The crude was purified by column chromatography on silica gel (Cyclohexane/EtOAc 100:0 to 98:2). Concentration of the appropriate fractions afforded **S28** as an impure mixture. **S28** was used to the next step without further purification.

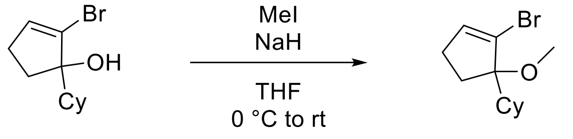

S28 **4g**

To a dry flask charged with a solution of NaH (202 mg, 1.41 mmol, 1.5 eq, 60% dispension in oil) in THF (0.75 ml, 1.24 M) a solution of 2-bromo-[1,1'-bi(cyclopentan)]-2-en-1-ol (**S28**, 230 mg, 0.94 mmol, 1 eq) in THF (2.35 ml, 0.4 M) was added dropwise at -10 °C. After 30 min iodomethane (0.09 ml, 1.41 mmol, 1.5 eq) was added dropwise and the flask was left at rt overnight. After 16 h the reaction mixture was quenched with 5 ml of distilled water and extracted x3 with EtOAc. The combined organic layers were washed x1 with distilled water and x1 with brine, dried over anhydrous Na_2SO_4 and evaporated under reduced pressure to afford a yellow oil. The crude was purified by column chromatography on silica gel (Cyclohexane/EtOAc 100:0 to 99:1). Concentration of the appropriate fractions afforded **4g** as a yellow oil.

Yield: 88 mg, 36%.

Rf: 0.43 (Cyclohexane/EtOAc 95:5).

Aspect: yellow oil

^{1}H NMR (500 MHz, CDCl$_3$): δ (ppm) 6.18 ('t', *J* = 2.6 Hz, 1H), 3.12 (s, 3H), 2.32 (dddd, *J* = 16.9, 9.3, 5.0, 2.5 Hz, 1H), 2.21 (dddd, *J* = 17.0, 9.4, 3.6, 2.6 Hz, 1H), 2.06 – 2.00 (m, 1H), 2.00 – 1.95 (m, 1H), 1.83 (ddd, *J* = 14.8, 9.3, 3.6 Hz, 1H), 1.79 – 1.73 (m, 2H), 1.73 – 1.65 (m, 2H), 1.47 (dtt, *J* = 12.2, 3.4, 1.5 Hz, 1H), 1.33 – 1.27 (m, 1H), 1.25 (ddt, *J* = 8.8, 6.5, 3.3 Hz, 1H), 1.17 – 1.05 (m, 1H), 0.95 (tdd, *J* = 12.8, 11.8, 3.7 Hz, 1H), 0.82 (qd, *J* = 12.6, 3.6 Hz, 1H).

^{13}C NMR (126 MHz, CDCl$_3$): δ (ppm) 135.8, 125.6, 93.4, 50.0, 44.2, 31.0, 27.8, 26.9, 26.5, 26.4, 26.4, 24.8.

IR (ATR): ν_{max} 2927, 2854, 1449, 1080, 634 cm^{-1}.

HRMS: data for this compound could not be obtained due to poor ionization. Instead the GC/MS spectrum is provided among with major fragments.

GC/MS: Rt = 9.092 min

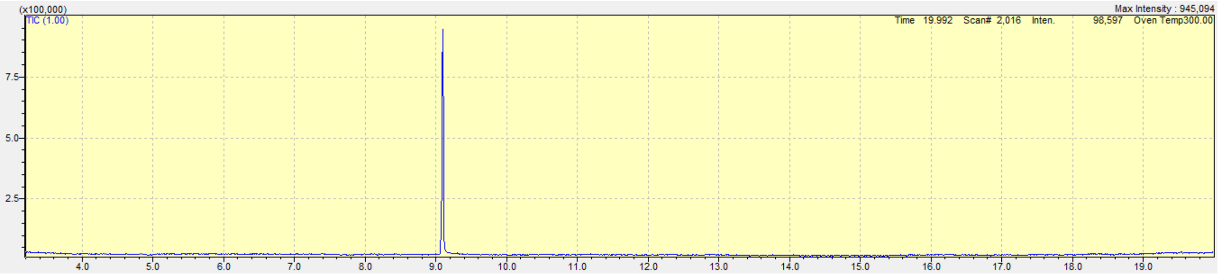

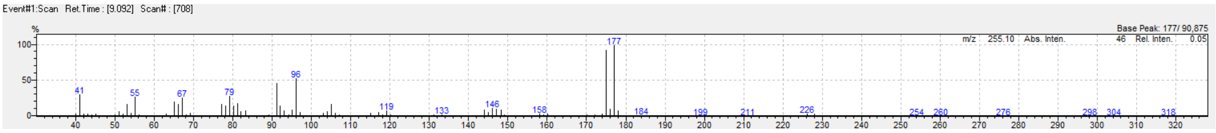

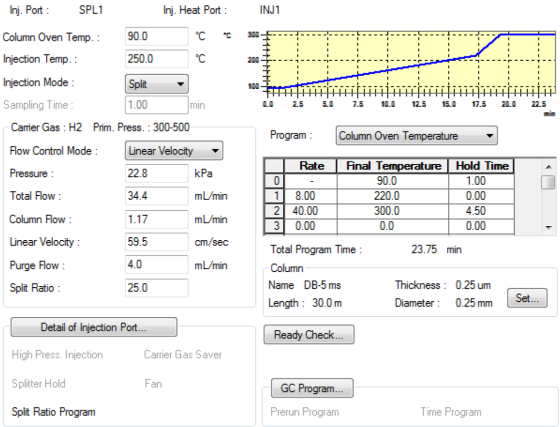

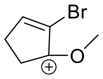

Chemical Formula: $C_6H_8BrO^+$
Exact Mass: 174.9753

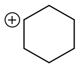

Chemical Formula: $C_6H_{11}^+$
Exact Mass: 83.0855

Br

Exact Mass: 78.9183

C(sp^3)–H Activation

Cyclobutanation (General Procedure E)

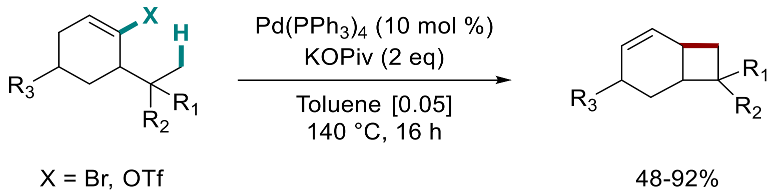

A 10 ml Pyrex tube containing the corresponding C–H activation substrate (0.1 mmol, 1 eq) was transferred in an argon-filled glovebox where $Pd(PPh_3)_4$ (11.6 mg, 0.01 mmol, 10 mol %), potassium pivalate (28.0 mg, 0.2 mmol, 2 eq) and toluene (2 ml, 0.05 M) were added. The tube was sealed with a screw cup and placed at a heating block preheated at 140 °C at 800 rpm for 16 h. After cooled to room temperature the crude mixture was filtered through celite®. The celite® was washed with 10 ml of EtOAc and the liquors were washed x2 with NaOH 1M, x1 with brine, dried over anhydrous Na_2SO_4 and evaporated under reduced pressure. The crude was purified by column chromatography on silica gel.

Note: visualization of C–H activation products during column chromatography was performed using $KMnO_4$ stain. All C–H activation products stain yellow.

Gram scale: A 250ml Ace round-bottom pressure flask containing **1g** (1.43 g, 4.7 mmol, 1 eq) was transferred in an argon-filled glovebox where $Pd(PPh_3)_4$ (541 mg, 0.01 mmol, 10 mol %), potassium pivalate (1.3 g, 0.2 mmol, 2 eq) and toluene (94 ml, 0.05 M) were added. The flask was sealed with a screw cup and placed in a preheated oil bath at 140 °C at 800 rpm for 20 h. After cooled to room temperature the crude mixture was filtered through celite®. The celite® was washed with 50 ml of EtOAc and the liquors were washed x2 with NaOH 1M, x1 with brine, dried over anhydrous Na_2SO_4 and evaporated under reduced pressure. The crude was purified by column chromatography on silica gel (Pentane/DEE 100:0 to 95:5). Concentration of the appropriate fractions afforded **2g** as a white solid (883 mg, 84%). *Crystal growth for x-ray crystallography*: 150 mg of **2g** were placed in a vial and were dissolved in 0.3 ml of warm *n*-Heptane. The vial was placed in the fridge (5 °C). After two weeks, clear needle-like crystals were formed and submitted for x-ray diffraction analysis.

Azetidination (General Procedure F)

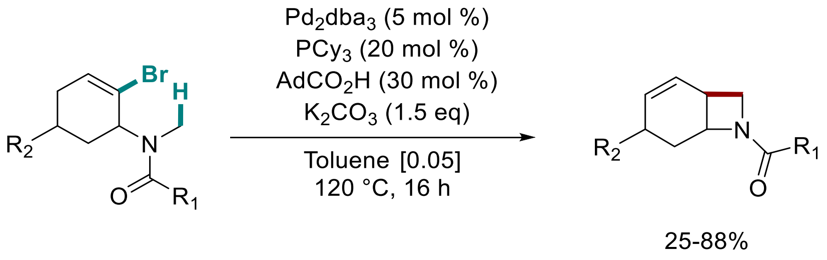

A 10 ml Pyrex tube containing the corresponding C–H activation substrate (0.1 mmol, 1 eq) and adamantine-1-carboxylic acid (5.41 mg, 0.03 mmol, 30 mol %) was transferred in an argon-filled glovebox where Pd_2dba_3 (4.58 mg, 0.005 mmol, 5 mol %), tricyclohexylphosphine (5.61 mg, 0.02 mmol, 20 mol %), potassium carbonate (20.7 mg, 0.15 mmol, 1.5 eq) and toluene (2 ml, 0.05 M) were added. The tube was sealed with a screw cup and placed at a heating block preheated at 120 °C at 800 rpm for 16 h. After cooled to room temperature the crude mixture was filtered through celite®. The celite®

was washed with 10 ml of EtOAc and the liquors were washed x2 with NaOH 1 M, x1 with brine, dried over anhydrous Na_2SO_4 and evaporated under reduced pressure. The crude was purified by column chromatography on silica gel.

Note: visualization of C–H activation products during column chromatography was performed using $KMnO_4$ stain. All C–H activation products stain yellow.

Oxetanation (General Procedure G)

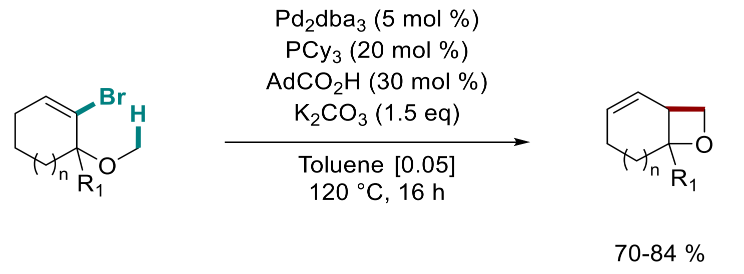

A 10 ml Pyrex tube containing the corresponding C–H activation substrate (0.1 mmol, 1 eq) and adamantine-1-carboxylic acid (5.41 mg, 0.03 mmol, 30 mol %) was transferred in an argon-filled glovebox where Pd_2dba_3 (4.58 mg, 0.005 mmol, 5 mol %), tricyclohexylphosphine (5.61 mg, 0.02 mmol, 20 mol %), potassium carbonate (20.7 mg, 0.15 mmol, 1.5 eq) and toluene (2 ml, 0.05 M) were added. The tube was sealed with a screw cup and placed at a heating block preheated at 120 °C at 800 rpm for 16 h. After cooled to room temperature the crude mixture was filtered through celite®. The celite® was washed with 10 ml of EtOAc and the liquors were washed x2 with NaOH 1 M, x1 with brine, dried over anhydrous Na_2SO_4 and evaporated under reduced pressure. The crude was purified by column chromatography on silica gel.

Note: visualization of C–H activation products during column chromatography was performed using $KMnO_4$ stain. All C–H activation products stain yellow.

C–H activation products – Cyclobutanation

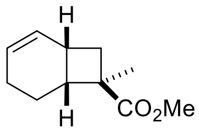

2a
d.r. 9:1

General procedure E: from **1a** (26.1 mg, 0.1 mmol)

Yield: 15 mg, 83%, Eluent: Pentane/DEE 100:0 to 95:5. Column chromatography was performed using 10% w/w $AgNO_3$ coated silica.

Rf: 0.3 (Pentane/DEE 97:3)

Aspect: colorless oil.

^{1}H NMR (500 MHz, $CDCl_3$): δ (ppm) *major diastereomer*: 5.80 (dddd, *J* = 10.0, 5.2, 2.9, 1.7 Hz, 1H), 5.69 (dddd, *J* = 10.0, 3.7, 2.4, 1.4 Hz, 1H), 3.71 (s, 3H), 2.81 – 2.76 (m, 1H), 2.73 (ddd, *J* = 8.8, 6.3, 2.8 Hz, 1H), 2.62 (ddd, *J* = 11.5, 8.5, 3.1 Hz, 1H), 2.11 – 2.03 (m, 1H), 1.95 – 1.86 (m, 1H), 1.77 – 1.66 (m, 3H), 1.26 (s, 3H).

^{13}C NMR (126 MHz, $CDCl_3$): δ (ppm) *major diastereomer*: 179.2, 130.2, 127.9, 52.1, 44.9, 39.4, 37.9, 29.1, 22.6, 21.6, 19.6.

IR (ATR): ν_{max} 3020, 2973, 2935, 1729, 1458, 1436, 1283, 1195, 1192 cm^{-1}.

HRMS: (ESI) m/z $[M + H]^+$ calculated for $C_{11}H_{17}O_2$: 181.1223; found: 181.1222.

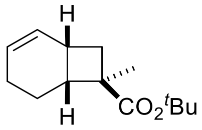

2b
d.r. 10:1

General procedure E: from **1b** (30.3 mg, 0.1 mmol)

Yield: 18.8 mg, 85% (major diastereomer), Eluent: Pentane/DEE 100:0 to 99:1. Column chromatography was performed using 10% w/w $AgNO_3$ coated silica. NOTE: the d.r. of 10:1 was reported in the crude mixture. Only the major diastereomer was isolated.

Rf: 0.5 (Pentane/DEE 95:5)

Aspect: colorless oil.

^{1}H NMR (250 MHz, $CDCl_3$): δ (ppm) *major diastereomer*: 5.80 (dddd, *J* = 10.1, 5.2, 2.9, 1.9 Hz, 1H), 5.69 (dddd, *J* = 10.0, 3.8, 2.4, 1.3 Hz, 1H), 2.78 – 2.72 (m, 1H), 2.68 ('tdd', *J* = 9.1, 6.6, 2.8 Hz, 1H), 2.56 (ddd, *J* = 11.5, 8.7, 2.9 Hz, 1H), 2.11 – 2.02 (m, 1H), 1.95 – 1.87 (m, 1H), 1.75 – 1.66 (m, 2H), 1.66 – 1.61 (m, 1H), 1.47 (s, 9H), 1.22 (s, 3H).

^{13}C NMR (126 MHz, CDCl$_3$): δ (ppm) *major diastereomer*: 178.1, 130.4, 127.9, 79.8, 45.6, 39.4, 37.9, 29.2, 28.2, 22.7, 21.7, 19.7.

IR (ATR): ν_{max} 3019, 2977, 2933, 1721, 1144, 1130 cm^{-1}.

HRMS: (ESI) m/z [M + H]$^+$ calculated for $C_{14}H_{23}O_2$: 223.1693; found: 223.1693.

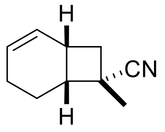

2c
d.r. 1.15:1

General procedure E: from **1c** (22.8 mg, 0.1 mmol)

Yield: 7.7 mg, 52%, Eluent: Pentane/DEE 100:0 to 95:5. Column chromatography was performed using 10% w/w $AgNO_3$ coated silica.

Rf: 0.2 (Pentane/DEE 98:2)

Aspect: yellow oil.

^{1}H NMR (250 MHz, CDCl$_3$): δ (ppm) *mixture of diastereomers*: 5.90 (ddt, *J* = 10.1, 5.9, 2.3 Hz, 0.54H, *major*), 5.84 (ddt, *J* = 10.2, 5.8, 2.3 Hz, 0.46H, *minor*), 5.70 – 5.63 (m, 1H), 3.05 (dd, *J* = 11.6, 7.1 Hz, 0.46H, *minor*), 2.90 (dddd, *J* = 14.6, 8.5, 3.9, 2.1 Hz, 0.54H, *major*), 2.86 – 2.78 (m, 0.46H, *minor*), 2.62 (ddd, *J* = 11.5, 8.6, 3.1 Hz, 0.46H, *minor*), 2.37 – 2.29 (m, 1H), 2.23 – 2.15 (m, 1H), 2.11 – 2.04 (m, 0.54H, *major*), 2.00 (ddd, *J* = 9.9, 6.0, 1.7 Hz, 0.56H, *major*), 1.97 – 1.91 (m, 1H), 1.89 – 1.83 (m, 0.56H, *major*), 1.89 – 1.82 (m, 0.54H, *major*), 1.77 (ddt, *J* = 13.3, 6.5, 4.3 Hz, 0.46H, *minor*), 1.70 – 1.63 (m, 0.46H, *minor*), 1.60 (s, 1.60H, *major*), 1.38 (s, 1.40H, *minor*).

^{13}C NMR (126 MHz, CDCl$_3$): δ (ppm) *mixture of diastereomers*: 129.0, 128.8, 128.7, 128.4, 126.9, 123.4, 42.0, 41.4, 39.7, 39.1, 32.5, 32.0, 29.6, 28.4, 24.7, 24.2, 22.13, 22.06, 21.0, 19.7.

IR (ATR): ν_{max} 3025, 2928, 2855, 2364, 2337, 1450 cm^{-1}.

HRMS: (ESI) m/z [M + Na]$^+$ calculated for $C_{10}H_{13}NNa$: 170.0940; found: 170.0943.

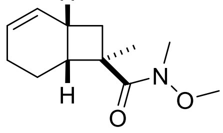

2d
major diastereomer

General procedure E: from **1d** (29.0 mg, 0.1 mmol)

Yield: 19 mg, 90%, Eluent: Pentane/DEE 100:0 to 80:20. Column chromatography was performed using 10% w/w $AgNO_3$ coated silica. NOTE: although a diastereomeric mixture is formed only the major diastereomer was isolated.

Rf: 0.12 (Pentane/DEE 75:25)

Aspect: yellow oil

^{1}H NMR (500 MHz, CDCl$_3$): δ (ppm) *major* diastereomer: 5.82 – 5.76 (m, 1H), 5.77 – 5.71 (m, 1H), 3.67 (s, 3H), 3.19 (s, 3H), 2.86 – 2.81 (m, 1H), 2.78 (ddt, *J* = 11.4, 9.5, 1.4 Hz, 1H), 2.61 – 2.53 (m, 1H), 2.17 – 2.06 (m, 1H), 2.01 – 1.90 (m, 1H), 1.83 – 1.75 (m, 1H), 1.75 – 1.68 (m, 1H), 1.61 (ddd, *J* = 11.5, 6.8, 1.0 Hz, 1H), 1.30 (s, 3H).

^{13}C NMR (126 MHz, CDCl$_3$): δ (ppm) *major diastereomer*: 179.6, 130.9, 127.2, 60.4, 45.7, 38.0, 37.5, 33.7, 28.1, 22.7, 21.0, 19.7.

IR (ATR): ν_{max} 3015, 2931, 1655, 1461, 1370, 1006 cm^{-1}.

HRMS: (ESI) m/z [M + H]$^+$ calculated for $C_{12}H_{20}NO_2$: 210.1489; found: 210.1488.

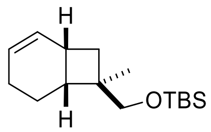

2e
d.r. 1.9:1

General procedure E: from **1e** (34.7 mg, 0.1 mmol)

Yield: 20.1 mg, 75%, Eluent: pure Pentane. Column chromatography was performed using 10% w/w $AgNO_3$ coated silica.

Rf: 0.5 (pure Pentane)

Aspect: colorless oil

^{1}H NMR (500 MHz, CDCl$_3$): δ (ppm) 5.79 (dddd, *J* = 10.2, 5.9, 2.7, 1.9 Hz, 1H), 5.69 (ddtd, *J* = 10.1, 3.8, 2.6, 1.1 Hz, 1H), 3.61 (d, *J* = 9.8 Hz, 0.35H, *minor*), 3.49 (s, 1.31H, *major*), 3.29 (d, *J* = 9.8 Hz, 0.35H, *minor*), 2.78 – 2.70 (m, 0.35H, *minor*), 2.69 – 2.61 (m, 0.65H, *major*), 2.14 – 2.12 (m, 0.65H, *major*), 2.06 – 2.04 (m, 0.35H, *minor*), 2.03 – 1.95 (m, 1.65H), 1.89 – 1.78 (m, 1H), 1.76 – 1.72 (m, 0.35H, *minor*), 1.72 – 1.61 (m, 2H), 1.54 – 1.51 (m, 0.35H, *minor*), 1.51 – 1.45 (m, 0.65H, *major*), 1.22 (s, 1.03H, *minor*), 0.96 (s, 1.96H, *major*), 0.90 (s, 5.89H, *major*), 0.88 (s, 3.11H, *minor*), 0.05 (s, 3.93H, *major*), 0.02 (d, *J* = 1.3 Hz, 2.06H, *minor*).

^{13}C NMR (126 MHz, CDCl$_3$): δ (ppm) *major diastereomer*: 131.2, 127.8, 71.3, 41.3, 37.7, 36.41, 28.7, 26.1, 23.0, 22.4, 19.7, 18.5, -5.22, -5.24. *minor diastereomer*: 131.0, 128.1, 68.6, 40.0, 39.2, 36.35, 28.0, 26.0, 25.1, 23.0, 22.0, 18.4, -5.3, -5.4.

IR (ATR): ν_{max} 2929, 2953, 2856, 1463, 1254, 1096, 837, 775 cm^{-1}.

HRMS: (ESI) m/z [M + Ag]$^+$ calculated for $C_{16}H_{30}AgOSi$: 373.1111; found: 373.1105

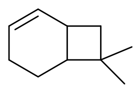

2f

General procedure E: from **1f** (57.2 mg, 0.2 mmol, with 0.02 mmol of $Pd(PPh_3)_4$, 0.4 mmol of KOPiv and 4ml of *d*$_8$-toluene.

Yield: 15.4 mg, 56% (94% NMR yield), Eluent: pure Pentane. Column chromatography was performed using 10% w/w $AgNO_3$ coated silica. NOTE: **2f** has a low boiling point. Removal of pentane after column chromatography was not possible without major loss of the product. Isolated yield was calculated using trichloroethylene as external standard.

Rf: 0.7 (pure Pentane)

Aspect: white oil

^{1}H NMR (500 MHz, $CDCl_3$): δ (ppm) 5.78 (ddt, *J* = 10.2, 6.0, 2.2 Hz, 1H), 5.69 (dddd, *J* = 9.9, 3.7, 2.6, 1.0 Hz, 1H), 2.77 – 2.70 (m, 1H), 2.04 – 1.94 (m, 2H), 1.87 – 1.79 (m, 2H), 1.70 – 1.58 (m, 3H), 1.19 (s, 3H), 0.96 (s, 3H).

^{13}C NMR (126 MHz, $CDCl_3$): δ (ppm) 131.1, 127.9, 42.5, 40.9, 34.7, 29.8, 28.4, 24.4, 23.1, 22.7.

HRMS: data for this compound could not be obtained due to poor ionization. Instead the GC/MS trace is provided among with major fragments.

GC/MS: Rt = 4.217 min

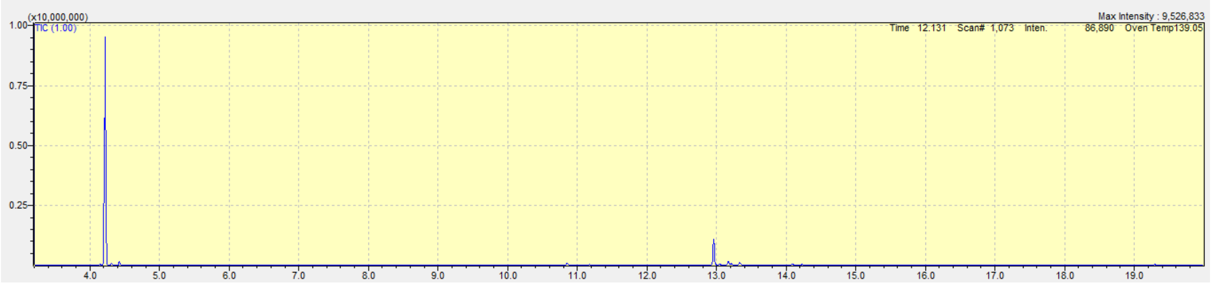

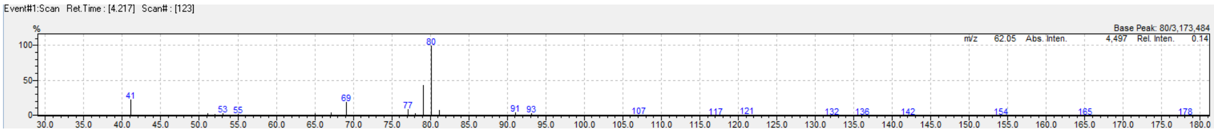

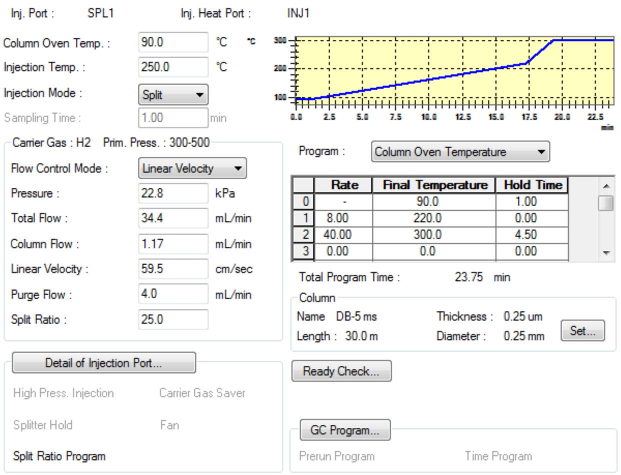

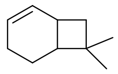

Chemical Formula: $C_{10}H_{16}$
Exact Mass: 136.1252

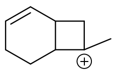

Chemical Formula: $C_9H_{13}^+$
Exact Mass: 121.1012

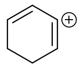

Chemical Formula: $C_6H_7^+$
Exact Mass: 79.0542

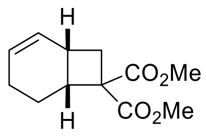

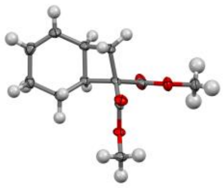

2g

General procedure E: from **1g** (61 mg, 0.2 mmol, with 0.02 mmol of $Pd(PPh_3)_4$ and 0.4 mmol of KOPiv)

Yield: 41 mg, 92%, Eluent: Pentane/DEE 100:0 to 95:5.

Rf: 0.2 (Pentane/DEE 96:4)

Aspect: white solid, m.p. 48.3 – 50.8 °C (crystallized from *n*-Heptane)

^{1}H NMR (400 MHz, $CDCl_3$): δ (ppm) 5.82 ('ddt', *J* = 10.3, 5.2, 2.3 Hz, 1H), 5.64 (dddd, *J* = 9.9, 3.8, 2.4, 1.2 Hz, 1H), 3.76 (s, 3H), 3.72 (s, 3H), 3.17 (dddd, *J* = 11.0, 8.8, 6.4, 2.9 Hz, 1H), 2.89 (d, *J* = 9.6 Hz, 1H), 2.62 (ddd, *J* = 12.1, 9.5, 0.9 Hz, 1H), 2.41 (ddd, *J* = 11.9, 8.9, 3.0 Hz, 1H), 2.01 – 1.87 (m, 2H), 1.81 (m, 1H), 1.54 – 1.43 (m, 1H).

^{13}C NMR (101 MHz, $CDCl_3$): δ (ppm) 172.5, 170.4, 128.8, 128.4, 54.1, 52.9, 52.3, 39.5, 34.6, 28.5, 22.5, 22.0.

IR (ATR): ν_{max} 3025, 2958, 2939, 1726, 1453, 1431, 1255, 1116, 704, 686 cm^{-1}.

HRMS: (ESI) m/z $[M + H]^+$ calculated for $C_{12}H_{16}NaO_4$: 247.0941; found: 247.0941.

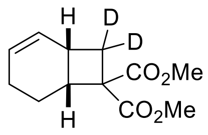

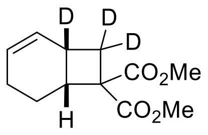

7:3

***d*$_2$-2g** ***d*$_3$-2g**

General procedure E: from ***d*$_3$-1g** (30.8 mg, 0.1 mmol)

Yield: 17.4 mg, 78%, Eluent: Pentane/DEE 100:0 to 98:2.

Rf: 0.2 (Pentane/DEE 96:4)

Aspect: yellow oil

^{1}H NMR (400 MHz, $CDCl_3$): δ (ppm) 5.85 – 5.80 (m, 1H), 5.64 (dddd, *J* = 10.0, 3.8, 2.5, 1.2 Hz, 1H), 3.76 (s, 3H), 3.72 (s, 3H), 3.16 (ddd, *J* = 11.2, 8.4, 6.5 Hz, 1H), 2.87 (d, *J* = 8.3 Hz, 0.7H), 1.96 (dq, *J* = 16.2, 4.9 Hz, 1H), 1.92 – 1.85 (m, 1H), 1.80 (ddt, *J* = 12.8, 6.5, 4.1 Hz, 1H), 1.49 (dddd, *J* = 13.0, 11.2, 10.2, 4.9 Hz, 1H).

^{13}C NMR (101 MHz, $CDCl_3$): δ (ppm) *major product*: 172.6, 170.4, 128.8, 128.4, 54.0, 52.9, 52.3, 39.5, 31.7, 28.2, 22.5, 22.0.

IR (ATR): ν_{max} 2953, 2921, 1735, 1436, 1262 cm^{-1}.

HRMS: (ESI) m/z $[M + Na]^+$ calculated for $C_{12}H_{14}D_2NaO_4$: 249.1066; found: 249.1064 and for $C_{12}H_{13}D_3NaO_4$: 250.1129; found: 250.1120

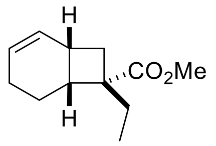

2h
d.r. 3.5:1

General procedure E: from **1h** (55 mg, 0.2 mmol, with 0.02 mmol of $Pd(PPh_3)_4$ and 0.4 mmol of KOPiv)

Yield: 20.8 mg, 52% – 13.1 mg *major diastereomer*, 7.7 mg mixture of diastereomers (d.r. 1:1.5). Eluent: Pentane/DEE 99:1. Column chromatography was performed using 10% w/w $AgNO_3$ coated silica.

Rf: 0.25 (Pentane/DEE 90:10)

Aspect: yellow oil

^{1}H NMR (500 MHz, $CDCl_3$): δ (ppm) *major diastereomer*: 5.82 – 5.77 (m, 1H), 5.67 (dddd, *J* = 9.8, 3.7, 2.4, 1.0 Hz, 1H), 3.67 (s, 3H), 2.75 – 2.66 (m, 1H), 2.32 – 2.24 (m, 2H), 2.02 – 1.97 (m, 1H), 1.97 – 1.91 (m, 2H), 1.86 – 1.82 (m, 1H), 1.83 – 1.75 (m, 2H), 1.43 – 1.37 (m, 1H), 0.82 (t, *J* = 7.4 Hz, 3H).

^{13}C NMR (126 MHz, $CDCl_3$): δ (ppm) *major diastereomer*: 176.0, 129.9, 128.0, 51.3, 49.6, 42.4, 33.0, 30.5, 27.9, 23.6, 22.5, 9.6.

IR (ATR): ν_{max} 2964, 2929, 1736 cm^{-1}.

HRMS: (ESI) m/z $[M + H]^+$ calculated for $C_{12}H_{19}O_2$: 195.1380; found: 195.1378.

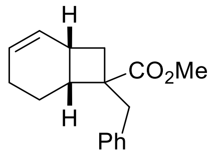

2i
d.r. 1.25:1

General procedure E: from **1i** (33.7 mg, 0.1 mmol)

Yield: 40% NMR yield using trichloroethylene (TCE) as internal standard (The crude ^{1}H NMR spectrum in $CDCl_3$ is given below as proof of conversion). Attempts to purify the product were unsuccessful. The ^{1}H NMR spectrum of the cleanest fraction in $CDCl_3$ is given below as proof of the C–H activation preference for the primary vs the secondary C–H bond and also as proof of the reported diastereomeric ratio (key ^{1}H peaks are integrated).

Rf: 0.15 (Pentane/DEE 97.5:2.5)

^{1}H NMR (500 MHz, $CDCl_3$) key peaks are integrated: δ (ppm) 7.25 – 7.15 (m, 3H), 7.13 – 7.11 (m, 1H), 7.06 – 7.03 (m, 1H), 5.87 – 5.77 (m, 1H), 5.71 – 5.65 (m, 1H), 3.63 (s, 1.35H, *minor*), 3.62 (s, 1.65H, *major)*, 3.32 (dd, *J* = 13.7, 1.1 Hz, 0.45H, *minor*), 3.14 (d, *J* = 14.0 Hz, 0.55H, *major*), 3.02 ('dd', *J* = 13.8, 7.2 Hz, 1H).

IR (ATR): ν_{max} 2926, 2854, 1733, 1454, 1198, 1177 cm^{-1}.

HRMS: (ESI) m/z $[M + H]^+$ calculated for $C_{17}H_{21}O_2$: 257.1536; found: 257.1531.

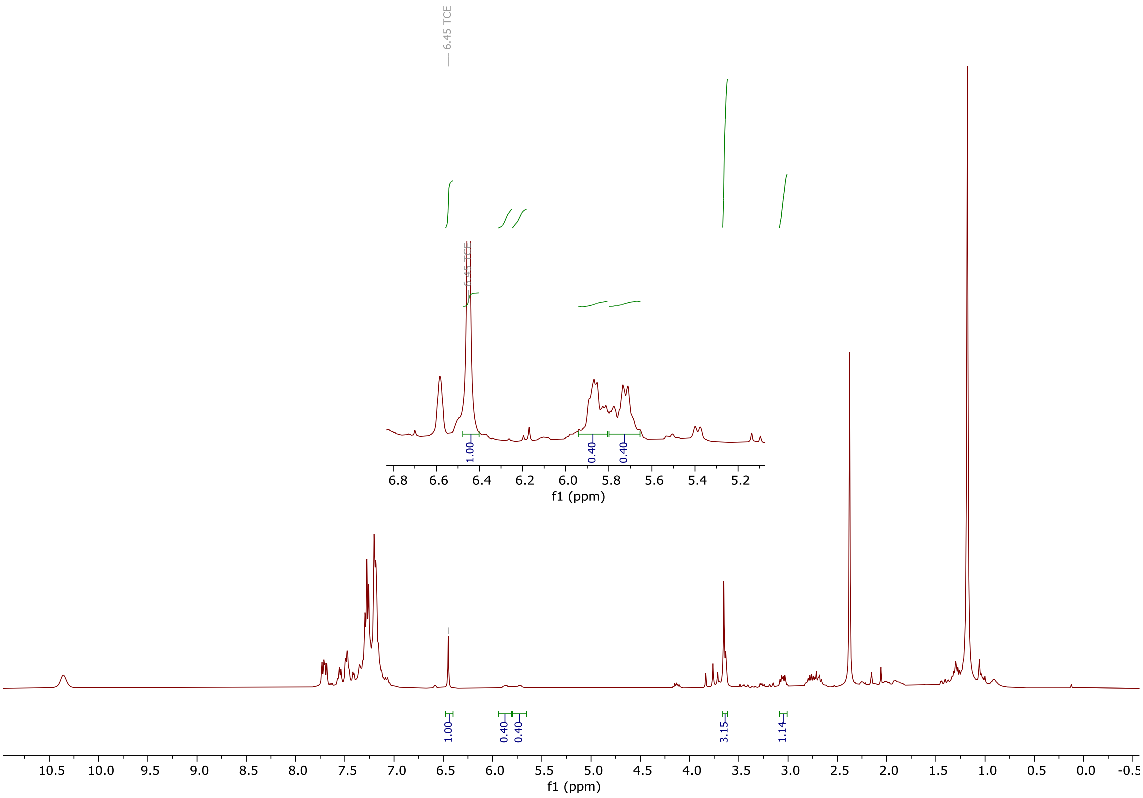

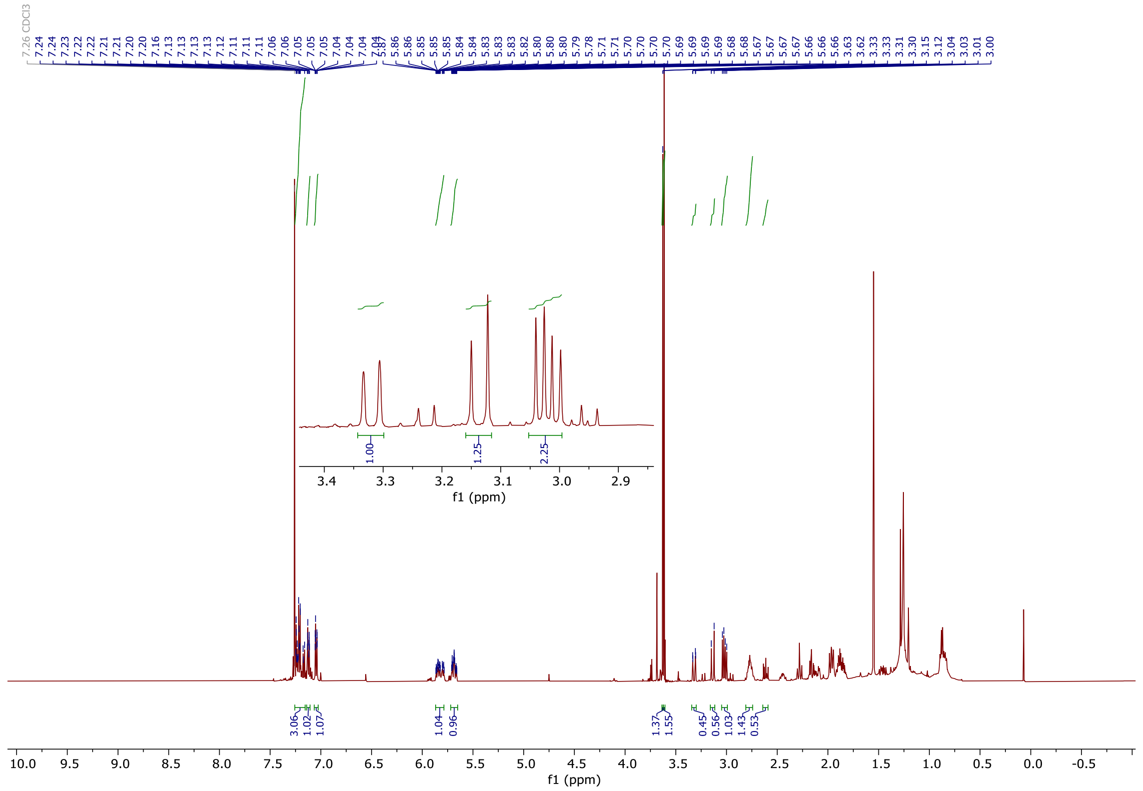

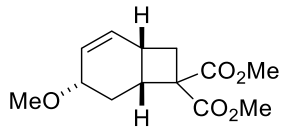

2j
d.r. 2:1

General procedure E: from **1j** (33.5 mg, 0.1 mmol)

Yield: 19.8 mg, 65% – 10.4 mg *major diastereomer*, 2.3 mg *minor diastereomer*, 7.1 mg mixture of diastereomers (d.r. 1:1.25). Eluent: Pentane/DEE 100:0 to 92:8.

Major diastereomer

Yield: 10.4 mg, 41%

Rf: 0.32 (Pentane/DEE 65:35)

Aspect: yellow oil

^{1}H NMR (500 MHz, CDCl$_3$): δ (ppm) 5.84 (dd, *J* = 10.1, 1.7 Hz, 1H), 5.63 (ddd, *J* = 10.1, 3.4, 2.3 Hz, 1H), 3.77 (s, 3H), 3.72 (s, 3H), 3.72 – 3.68 (m, 1H), 3.38 (s, 3H), 3.16 – 3.10 (m, 1H), 2.94 – 2.86 (m, 1H), 2.63 (ddd, *J* = 12.0, 10.4, 0.9 Hz, 1H), 2.42 (ddd, *J* = 12.1, 8.9, 3.3 Hz, 1H), 2.24 (m, 1H), 1.33 (m, 1H).

^{13}C NMR (101 MHz, CDCl$_3$): δ (ppm) 172.3, 169.9, 131.3, 129.1, 74.1, 56.1, 53.0, 52.7, 52.4, 37.7, 34.0, 29.1, 28.7.

IR (ATR): ν_{max} 2954, 1735, 1455, 1436, 1266 cm^{-1}.

HRMS: (ESI) m/z [M + Na]$^+$ calculated for $C_{13}H_{18}NaO_5$: 277.1046; found: 277.1046.

Minor diastereomer

Yield: 2.3 mg, 10%

Rf: 0.26 (Pentane/DEE 65:35)

Aspect: yellow oil

^{1}H NMR (500 MHz, CDCl$_3$): δ (ppm) 5.97 (ddd, *J* = 10.0, 4.8, 1.8 Hz, 1H), 5.86 (ddd, *J* = 10.0, 4.1, 0.7 Hz, 1H), 3.76 (s, 3H), 3.74 (s, 3H), 3.73 – 3.72 (m, 1H), 3.44 – 3.39 (m, 1H), 3.34 (s, 3H), 3.01 – 2.94 (m, 1H), 2.59 (ddd, *J* = 12.1, 9.2, 0.9 Hz, 1H), 2.45 (ddd, *J* = 11.9, 9.1, 2.7 Hz, 1H), 1.95 (ddd, *J* = 13.7, 6.9, 4.8 Hz, 1H), 1.68 (ddd, *J* = 13.7, 10.1, 3.7 Hz, 1H).

^{13}C NMR (126 MHz, CDCl$_3$): δ (ppm) 172.3, 170.4, 132.5, 127.8, 71.0, 56.4, 53.5, 53.0, 52.4, 35.4, 33.5, 28.1, 26.6.

IR (ATR): ν_{max} 2954, 2926, 1735, 1436, 1268 cm^{-1}.

HRMS: (ESI) m/z [M + Na]$^+$ calculated for $C_{13}H_{18}NaO_5$: 277.1046; found: 277.1046.

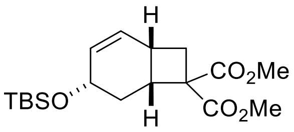

2k
d.r. 8:1

General procedure E: from **1k** (43.5 mg, 0.1 mmol)

Yield: 17 mg, 48%, Eluent: Pentane/DEE 100:0 to 95:5.

Rf: 0.23 (Pentane/DEE 90:10)

Aspect: yellow oil

^{1}H NMR (500 MHz, $CDCl_3$): δ (ppm) 5.82 (ddd, *J* = 10.1, 4.0, 1.4 Hz, 0.11H, *minor*), 5.75 (ddd, *J* = 10.0, 4.3, 1.1 Hz, 0.11H, *minor*), 5.71 (m, 0.89H, *major*), 5.55 (ddd, *J* = 10.1, 3.4, 2.2 Hz, 0.89H, *major*), 4.19 – 4.12 (m, 1H), 3.76 (s, 2.67H, *major*), 3.75 (s, 0.33H, *minor*), 3.73 (s, 0.33H, *minor*), 3.73 (s, 2.67H, *major*), 3.48 (m, 0.11H, *minor*), 3.16 – 3.08 (m, 0.89H, *major*), 2.95 – 2.90 (m, 0.11H, *minor*), 2.86 (tddd, *J* = 10.7, 8.3, 4.2, 2.1 Hz, 0.89H, *major*), 2.63 (ddd, *J* = 11.3, 10.4, 0.8 Hz, 0.89H, *major*), 2.55 (m, 0.11H, *minor*), 2.47 – 2.44 (m, 0.11H, *minor*), 2.41 (ddd, *J* = 12.0, 8.9, 3.3 Hz, 0.89H, *major*), 2.04 (m 0.89H, *major*), 1.80 (ddd, *J* = 13.6, 7.8, 4.5 Hz, 0.11H, *minor*), 1.68 (ddd, *J* = 13.4, 7.0, 6.1 Hz, 0.11H, *minor*), 1.46 – 1.38 (m, 0.89H, *major*), 0.89 (s, 8H, *major*), 0.87 (s, 1H, *minor*), 0.07 (d, *J* = 5.3 Hz, 5.34H, *major*), 0.05 (d, *J* = 1.9 Hz, 0.66H, *minor*).

^{13}C NMR (126 MHz, $CDCl_3$): δ (ppm) *major diastereomer*: 172.4, 169.9, 135.1, 128.1, 66.0, 53.02, 52.6, 52.4, 38.0, 34.0, 33.2, 28.4, 26.03, 18.4, -4.41, -4.6. *minor diastereomer*: 172.5, 170.6, 131.4, 130.4, 63.3, 53.4, 52.96, 52.5, 35.9, 31.1, 30.5, 27.5, 26.01, 18.3, -4.43, -4.5

IR (ATR): ν_{max} 2954, 2933, 1737, 1259, 1080, 836, 778 cm^{-1}.

HRMS: (ESI) m/z $[M + Na]^+$ calculated for $C_{18}H_{30}NaO_5Si$: 377.1755; found: 377.1757.

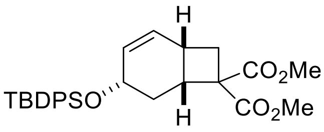

2l
d.r. 8:1

General procedure E: from **1l** (56 mg, 0.1 mmol)

Yield: 38 mg, 80%, Eluent: Pentane/DEE 100:0 to 90:10.

Rf: 0.18 (Pentane/DEE 90:10)

Aspect: yellow oil

^{1}H NMR (500 MHz, $CDCl_3$): δ (ppm) 7.68 – 7.64 (m, 4H), 7.44 – 7.40 (m, 2H), 7.39 – 7.34 (m, 4H), 5.77 (m, 0.89H, *major*), 5.69 (d, *J* = 3.7 Hz, 0.11H, *minor*), 5.67 (dd, *J* = 4.2, 1.3 Hz, 0.11H, *minor*), 5.49 (ddd, *J* = 10.1, 3.4, 2.3 Hz, 0.89H, *major*), 4.20 – 4.15 (m, 1H), 3.76 (s, 0.33H, *minor*), 3.72 (s, 2.67H, *major*), 3.68 (s, 2.67H, *major*), 3.59 – 3.53 (m, 0.11H, *minor*), 3.49 (s, 0.33H, *minor*), 2.99 – 2.87 (m, 1H), 2.76

(dddd, *J* = 11.8, 10.3, 5.8, 2.2 Hz, 0.89H, *major*), 2.62 (ddd, *J* = 11.3, 10.3, 0.7 Hz, 0.89H, *major*), 2.51 (ddd, *J* = 12.1, 8.7, 0.9 Hz, 0.11H, *minor*), 2.41 (dd, *J* = 9.3, 2.6 Hz, 0.11H, *minor*), 2.37 (ddd, *J* = 11.9, 8.8, 3.3 Hz, 0.89H, *major*), 1.96 (dddd, *J* = 12.2, 6.6, 4.5, 1.3 Hz, 0.89H, *major*), 1.83 (ddd, *J* = 13.6, 6.8, 5.2 Hz, 0.11H, *minor*), 1.67 (ddd, *J* = 13.4, 9.3, 4.0 Hz, 0.11H, *minor*), 1.47 (ddd, *J* = 13.4, 11.9, 10.2 Hz, 0.89H, *major*), 1.07 (s, 8H, *major*), 1.04 (s, 1H, *minor*).

^{13}C NMR (126 MHz, $CDCl_3$): δ (ppm) *major diastereomer*: 172.3, 170.0, 135.95, 135.91, 134.86, 134.43, 134.0, 129.8, 129.75, 127.94, 127.76, 127.67, 66.9, 53.0, 52.5, 52.3, 37.7, 33.9, 32.9, 28.3, 27.1, 19.26. *minor diastereomer*: 172.5, 170.4, 136.0, 135.94, 134.93, 134.5, 134.36, 130.7, 130.4, 127.85, 127.72, 127.64, 63.8, 53.5, 52.9, 52.2, 35.7, 33.4, 30.7, 27.9, 26.7, 19.33.

IR (ATR): ν_{max} 2954, 1734, 1265, 1110, 738, 706, 614 cm^{-1}.

HRMS: (ESI) m/z $[M + Na]^+$ calculated for $C_{28}H_{34}NaO_5Si$: 501.2068; found: 501.2063

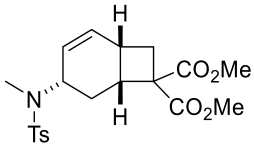

2m
d.r. 4:1

<u>General procedure E</u>: from **1m** (48.8 mg, 0.1 mmol)

Yield: 30.6 mg, 75%, Eluent: Cyclohexane/EtOAc 100:0 to 90:10 (*major diastereomer*: 22.4 mg, 58%).

Rf: 0.23 (Cyclohexane/EtOAc 75:25)

Aspect: colorless oil

^{1}H NMR (500 MHz, $CDCl_3$): δ (ppm) *major diastereomer*: 7.68 (d, *J* = 8.3 Hz, 2H), 7.29 (d, *J* = 8.1 Hz, 2H), 5.64 (ddd, *J* = 10.1, 3.7, 2.7 Hz, 1H), 5.24 (dq, *J* = 10.1, 1.7 Hz, 1H), 4.44 (ddq, *J* = 11.0, 4.2, 2.3 Hz, 1H), 3.75 (s, 3H), 3.65 (s, 3H), 3.23 – 3.13 (m, 1H), 3.23 – 3.13 (m, 1H), 2.69 (s, 3H), 2.56 (ddd, *J* = 11.4, 10.5, 0.8 Hz, 1H), 2.41 (s, 3H), 2.38 (ddd, *J* = 12.1, 8.9, 3.3 Hz, 1H), 1.77 (dddd, *J* = 11.9, 6.5, 4.1, 1.3 Hz, 1H), 1.46 – 1.37 (m, 1H).

^{13}C NMR (126 MHz, $CDCl_3$): δ (ppm) *major diastereomer*: 171.9, 169.9, 143.3, 136.9, 132.1, 129.8, 129.5, 127.2, 53.1, 52.7, 52.4, 52.1, 38.3, 34.1, 29.1, 28.1, 27.7, 21.6.

IR (ATR): ν_{max} 2654, 1730, 1453, 1435, 1337, 1266, 1165, 661 cm^{-1}.

HRMS: (ESI) m/z $[M + H]^+$ calculated for $C_{20}H_{25}NO_6S$: 408.1475; found: 408.1469.

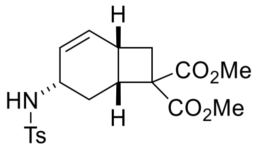

2n

<u>General procedure E</u>: from **1n** (47.4 mg, 0.1 mmol)

Yield: 37.4 mg, 95%, Cyclohexane/EtOAc 100:0 to 85:15

Rf: 0.13 (Cyclohexane/EtOAc 75:25)

Aspect: colorless oil

^{1}H NMR (500 MHz, CDCl$_3$): δ (ppm) 7.75 (d, *J* = 8.3 Hz, 2H), 7.30 (d, *J* = 7.8 Hz, 2H), 5.59 (ddd, *J* = 10.0, 3.6, 2.6 Hz, 1H), 5.44 (dq, *J* = 10.0, 1.6 Hz, 1H), 4.62 (d, *J* = 8.9 Hz, 1H), 3.76 – 3.71 (m, 1H), 3.74 (s, 3H), 3.65 (s, 3H), 3.14 – 3.06 (m, 1H), 2.83 (tddd, *J* = 10.6, 8.3, 4.1, 2.3 Hz, 1H), 2.55 (ddd, *J* = 11.2, 10.4, 0.9 Hz, 1H), 2.42 (s, 3H), 2.37 (ddd, *J* = 12.0, 8.9, 3.2 Hz, 1H), 2.01 (dddd, *J* = 12.2, 6.5, 4.3, 1.2 Hz, 1H), 1.27 – 1.23 (m, 1H).

^{13}C NMR (126 MHz, CDCl$_3$): δ (ppm) 171.9, 169.8, 143.6, 138.2, 130.9, 130.7, 129.9, 127.2, 53.1, 52.7, 52.5, 48.4, 38.0, 33.8, 31.2, 28.0, 21.7.

IR (ATR): ν_{max} 3279, 2955, 1727, 1435, 1328, 1267, 1158, 669 cm^{-1}.

HRMS: (ESI) m/z [M + Na]$^+$ calculated for $C_{19}H_{23}NNaO_6S$: 416.1138; found: 416.1142

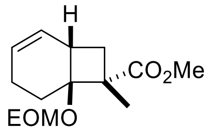

2o
d.r. 5.2:1

General procedure E: from **1o** (33.5 mg, 0.1 mmol)

Yield: 17.9 mg, 70%, Eluent: Pentane/DEE 100:0 to 90:10.

Rf: 0.17 (Pentane/DEE 90:10)

Aspect: colorless oil

^{1}H NMR (500 MHz, CDCl$_3$): δ (ppm) 5.86 – 5.81 (m, 1H), 5.66 (dddd, *J* = 9.9, 3.9, 2.7, 1.0 Hz, 1H), 5.06 (d, *J* = 7.3 Hz, 0.16H, *minor*), 4.87 (d, *J* = 7.7 Hz, 0.84H, *major*), 4.72 (d, *J* = 7.3 Hz, 0.16H, *minor*), 4.55 (d, *J* = 7.7 Hz, 0.84H, *major*), 3.82 (dq, *J* = 9.4, 7.1 Hz, 0.16H, *minor*), 3.73 (s, 2.52H, *major*), 3.72 – 3.69 (m, 0.84H, *major*), 3.68 (s, 0.48H, *minor*), 3.49 – 3.45 (m, 0.16H, *minor*), 3.41 (dq, *J* = 9.4, 7.1 Hz, 0.84H, *major*), 2.99 – 2.92 (m, 0.84H, *major*), 2.70 – 2.64 (m, 0.16H, *minor*), 2.60 (dd, *J* = 10.7, 9.8 Hz, 0.84H, *major*), 2.17 – 2.07 (m, 2H), 2.01 – 1.94 (m, 1H), 1.85 (t, *J* = 10.6 Hz, 0.16H, *minor*), 1.78 (dd, *J* = 11.1, 9.8 Hz, 0.16H, *minor*), 1.70 (ddd, *J* = 14.3, 12.2, 4.8 Hz, 0.84H, *major*), 1.48 – 1.41 (m, 0.16H, *minor*), 1.38 (s, 0.48H, *minor*), 1.32 (s, 2.52H, *major*), 1.21 – 1.18 (m, 0.48H, *minor*), 1.16 (t, *J* = 7.1 Hz, 2.52H, *major*), 1.08 (dd, *J* = 10.7, 9.9 Hz, 0.84H, *major*).

^{13}C NMR (126 MHz, CDCl$_3$): δ (ppm) *major diastereomer*: 175.7, 128.11, 128.09, 91.0, 80.9, 63.8, 52.2, 52.1, 38.5, 33.1, 26.4, 20.1, 19.5, 15.18. *minor diastereomer*: 176.0, 128.14, 127.7, 91.1, 79.7, 63.9, 51.8, 50.6, 36.1, 31.8, 27.6, 20.2, 19.7, 15.23.

IR (ATR): ν_{max} 3023, 2974, 2949, 1729, 1457, 1438, 1288, 1150, 1041, 1008 cm^{-1}.

HRMS: (ESI) m/z [M + Na]$^+$ calculated for $C_{14}H_{22}NaO_4$: 277.1410; found: 277.1410.

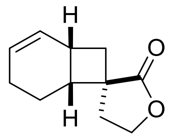

2p
major diastereomer

General procedure E: from **1p** (25.9 mg, 0.1 mmol)

Yield: 12 mg, 67%, Eluent: Pentane/DEE 100:0 to 90:10.

Rf: 0.3 (Pentane/DEE 60:40)

Aspect: yellow oil

^{1}H NMR (250 MHz, CDCl$_3$): δ (ppm) 5.84 (dddd, *J* = 9.9, 4.9, 3.2, 1.7 Hz, 1H), 5.75 – 5.67 (m, 1H), 4.27 – 4.17 (m, 1H), 4.17 – 4.09 (m, 1H), 3.10 (qd, *J* = 8.4, 1.6 Hz, 1H), 2.89 – 2.76 (m, 1H), 2.61 – 2.47 (m, 1H), 2.46 – 2.34 (m, 1H), 2.15 – 2.08 (m, 1H), 2.04 (ddd, *J* = 6.7, 2.9, 1.4 Hz, 1H), 2.02 – 1.95 (m, 1H), 1.94 – 1.84 (m, 1H), 1.83 – 1.74 (m, 1H), 1.73 – 1.60 (m, 1H).

^{13}C NMR (126 MHz, CDCl$_3$): δ (ppm) 182.3, 129.7, 127.9, 65.5, 44.1, 37.7, 36.8, 31.8, 28.5, 22.3, 22.1.

IR (ATR): ν_{max} 3020, 2970, 2928, 1761, 1166, 1023 cm^{-1}.

HRMS: (ESI) m/z [M + Na]$^+$ calculated for $C_{11}H_{14}NaO_2$: 201.0886; found: 201.0884

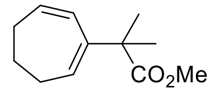

2q'

General procedure E: from **1q** (27.5 mg, 0.1 mmol)

Yield: 10.7 mg, 55%, Eluent: Pentane/DEE 100:0 to 98:2.

Rf: 0.2 (Pentane/DEE 98:2)

Aspect: colorless oil

^{1}H NMR (400 MHz, CDCl$_3$): δ (ppm) 5.84 – 5.80 (m, 2H), 5.70 (dd, *J* = 4.8, 2.4 Hz, 1H), 3.66 (s, 3H), 2.35 – 2.29 (m, 2H), 2.21 – 2.14 (m, 2H), 1.86 – 1.78 (m, 2H), 1.32 (s, 6H).

^{13}C NMR (126 MHz, CDCl$_3$): δ (ppm) 177.6, 147.9, 133.5, 125.0, 118.9, 52.1, 49.5, 32.6, 31.9, 27.5, 24.7.

IR (ATR): ν_{max} 2980, 2928, 1734, 1464, 1258, 1141 cm^{-1}.

HRMS: (ESI) m/z [M + H]$^+$ calculated for $C_{12}H_{19}O_2$: 195.1380; found: 195.1375.

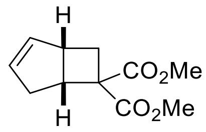

2r

General procedure E: from **1r** (58.2 mg, 0.2 mmol, with 0.02 mmol of $Pd(PPh_3)_4$ and 0.4 mmol of KOPiv)

Yield: 7 mg, 17%, Eluent: Pentane/DEE 98:2. Column chromatography was performed using 10% w/w $AgNO_3$ coated silica.

Rf: 0.18 (Pentane/DEE 80:20)

Aspect: yellow oil

^{1}H NMR (500 MHz, $CDCl_3$): δ (ppm) 5.77 – 5.75 (m, 1H), 5.75 – 5.73 (m, 1H), 3.74 (s, 3H), 3.72 (s, 3H), 3.72 – 3.71 (m, 1H), 3.28 – 3.22 (m, 1H), 2.65 (ddd, *J* = 12.6, 4.4, 1.0 Hz, 1H), 2.61 – 2.54 (m, 1H), 2.49 (ddd, *J* = 12.6, 8.7, 1.1 Hz, 1H), 2.37 – 2.31 (m, 1H).

^{13}C NMR (126 MHz, $CDCl_3$): δ (ppm) 172.9, 170.5, 133.4, 131.6, 55.0, 52.9, 52.4, 42.0, 41.1, 35.9, 34.7.

IR (ATR): ν_{max} 2921, 2852, 1720, 1260 cm^{-1}.

HRMS: (ESI) m/z $[M + Na]^+$ calculated for $C_{11}H_{14}NaO_4$: 201.0886; found: 201.0884.

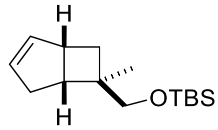

2s
d.r. 4:1

General procedure E: from **1s** (66.7 mg, 0.2 mmol, with 0.02 mmol of $Pd(PPh_3)_4$ and 0.4 mmol of KOPiv)

Yield: 16.7 mg, 33% (50% nmry), Eluent: pure Pentane. Column chromatography was performed using 10% w/w $AgNO_3$ coated silica.

Rf: 0.37 (pure Pentane)

Aspect: colorless oil

^{1}H NMR (400 MHz, $CDCl_3$): δ (ppm) 5.81 – 5.77 (m, 0.8H, *major*), 5.75 – 5.72 (m, 1.2H), 3.59 (d, *J* = 9.8 Hz, 0.2H, *minor*), 3.35 (s, 1.6H, *major*), 3.21 (d, *J* = 9.8 Hz, 0.2H, *minor*), 3.18 – 3.11 (m, 0.2H, *minor*), 3.10 – 3.03 (m, 0.8H, *major*), 2.71 (ddd, *J* = 7.6, 6.1, 4.6 Hz, 0.8H, *major*), 2.62 – 2.55 (m, 0.2H, *minor*), 2.52 ('d', *J* = 8.6 Hz, 0.2H, *minor*), 2.43 – 2.35 (m, 1.8H), 2.09 (dd, *J* = 11.7, 8.8 Hz, 0.8H, *major*), 1.83 (dd, *J* = 11.9, 8.8 Hz, 0.2H, *minor*), 1.36 (dd, *J* = 12.0, 4.2 Hz, 0.2H, *minor*), 1.29 (ddd, *J* = 11.7, 4.0, 0.9 Hz, 0.8H, *major*), 1.15 (s, 0.6H, *minor*), 0.94 (s, 2.4H, *major*), 0.90 (s, 7.2H, *major*), 0.88 (s, 1.8H, *minor*), 0.05 (s, 4.8H, *major*), 0.01 (s, 0.6H, *minor*), 0.00 (s, 0.6H, *minor*).

^{13}C NMR (126 MHz, $CDCl_3$): δ (ppm) *major diastereomer*: 135.6, 131.6, 72.6, 40.5, 40.1, 40.0, 36.1, 34.3, 26.1, 20.3, 18.5, -5.21, -5.23. *minor diastereomer*: 134.8, 131.8, 68.6, 43.6, 39.4, 39.1, 37.1, 34.5, 27.4, 26.0, 18.4, -5.4.

IR (ATR): ν_{max} 2955, 2930, 2858, 1092, 834, 775 cm^{-1}.

HRMS: data for this compound could not be obtained due to poor ionization. Instead the GC/MS spectrum is provided among with major fragments.

GC/MS: *minor diastereomer:* Rt = 6.625 min, *major diastereomer:* Rt = 6.933 min

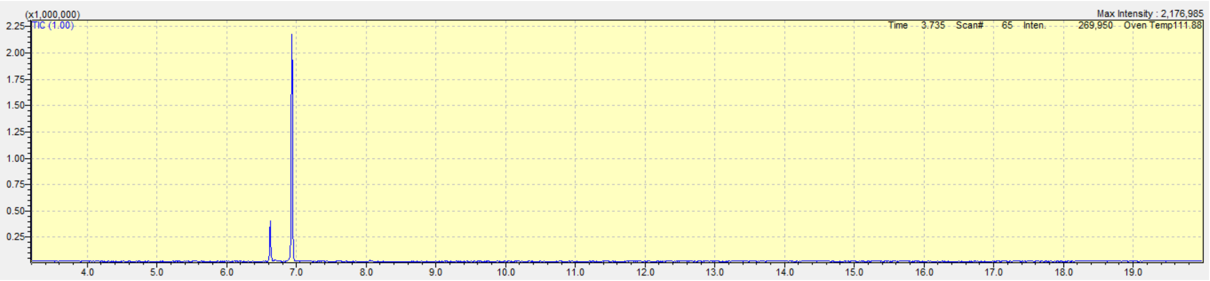

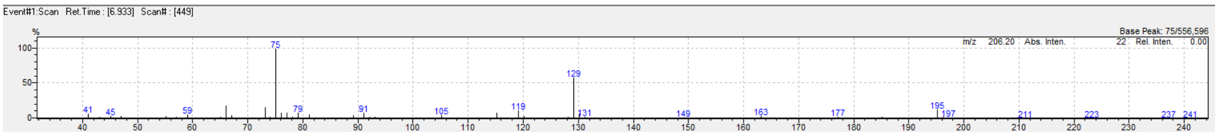

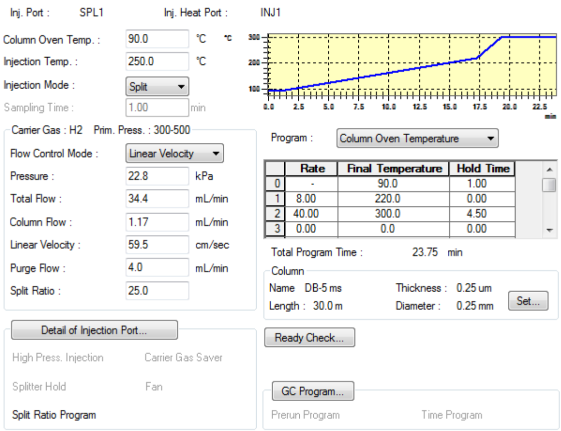

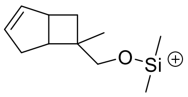

Chemical Formula: $C_{11}H_{19}OSi^+$
Exact Mass: 195.1200

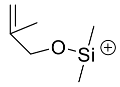

Chemical Formula: $C_6H_{13}OSi^+$
Exact Mass: 129.0730

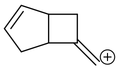

Chemical Formula: $C_8H_9^+$
Exact Mass: 105.0699

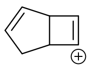

Chemical Formula: $C_7H_7^+$
Exact Mass: 91.0542

C–H activation products – Azetidination

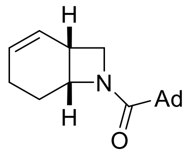

5a
e.r. 73:27

<u>General procedure F</u>: from scalemic **4a** (35.2 mg, 0.1 mmol)

Yield: 17.7 mg, 65%, Eluent: Cyclohexane/EtOAc 100:0 to 90:10.

Rf: 0.12 (Cyclohexane/EtOAc 85:15)

Aspect: yellow oil

^{1}H NMR (500 MHz, $CDCl_3$): δ (ppm) **5a-*rac***: 6.02 (ddd, J = 9.4, 6.2, 2.7 Hz, 1H), 5.81 (ddd, J = 10.0, 5.1, 2.6 Hz, 1H), 4.75 (dt, J = 7.6, 3.7 Hz, 1H), 4.53 (t, J = 8.1 Hz, 1H), 3.84 (t, J = 6.0 Hz, 1H), 2.95 (tt, J = 8.3, 4.7 Hz, 1H), 2.43 – 2.33 (m, 1H), 2.18 – 2.09 (m, 1H), 2.05 – 2.01 (m, 1H), 2.00 – 1.98 (m, 3H), 1.93 – 1.90 (m, 1H), 1.90 – 1.89 (m, 2H), 1.89 – 1.86 (m, 2H), 1.85 – 1.84 (m, 1H), 1.72 – 1.71 (m, 1H), 1.71 – 1.68 (m, 4H), 1.67 – 1.65 (m, 1H), 1.38 (t, J = 3.2 Hz, 1H).

^{13}C NMR (126 MHz, $CDCl_3$): δ (ppm) **5a-*rac***: 178.2, 130.5, 127.9, 60.9, 60.2, 41.6, 38.4, 36.8, 28.4, 28.3, 23.9, 20.3.

^{1}H NMR (500 MHz, $CDCl_3$): δ (ppm) **5a**: 6.01 (ddd, *J* = 9.4, 6.3, 2.7 Hz, 1H), 5.81 (ddd, *J* = 10.0, 5.2, 2.7 Hz, 1H), 4.74 (dt, *J* = 7.6, 3.6 Hz, 1H), 4.53 (t, *J* = 8.3 Hz, 1H), 3.84 (d, *J* = 7.1 Hz, 1H), 2.95 (tt, *J* = 8.4, 4.6 Hz, 1H), 2.43 – 2.34 (m, 1H), 2.18 – 2.08 (m, 1H), 2.04 – 2.01 (m, 1H), 2.00 – 1.97 (m, 3H), 1.92 – 1.90 (m, 1H), 1.89 – 1.88 (m, 2H), 1.88 – 1.86 (m, 2H), 1.86 – 1.83 (m, 1H), 1.73 – 1.70 (m, 1H), 1.70 – 1.67 (m, 4H), 1.67 – 1.64 (m, 1H), 1.44 – 1.33 (m, 1H).

^{13}C NMR (126 MHz, $CDCl_3$): δ (ppm) **5a**: 178.2, 130.6, 127.9, 60.9, 60.2, 41.6, 38.4, 36.8, 28.4, 28.3, 23.8, 20.4.

IR (ATR): ν_{max} 2906, 2851, 1620, 1402, 1386, 1168 cm^{-1}.

HRMS: (ESI) m/z $[M + H]^+$ calculated for $C_{18}H_{26}NO$: 272.2009; found: 272.2009.

$[\alpha]_D^{20}$ = -49.2 (c = 1.5, $CDCl_3$).

e.r. = 73:27; HYDRODEX β-6TBDM column (25 m x 0.25 mm ID) – H_2 as carrier gas

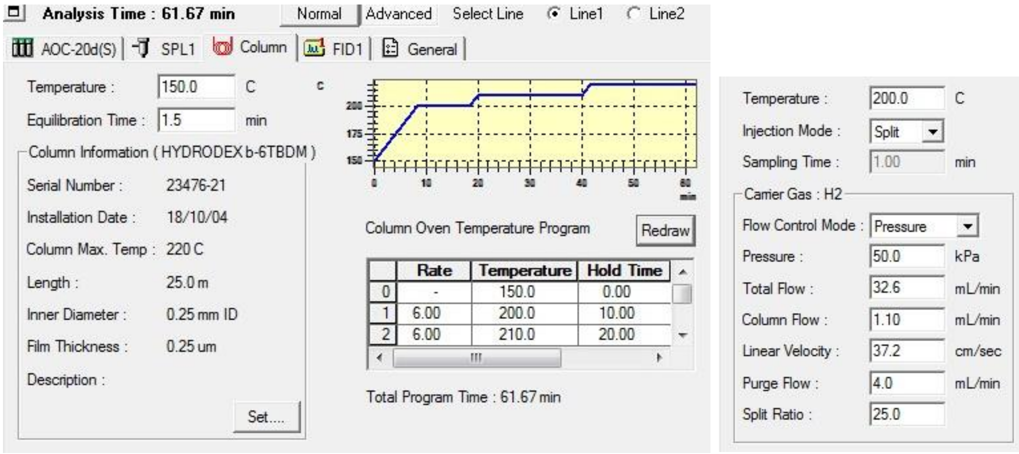

Spectrum of racemic mixture (**5a-*rac***):

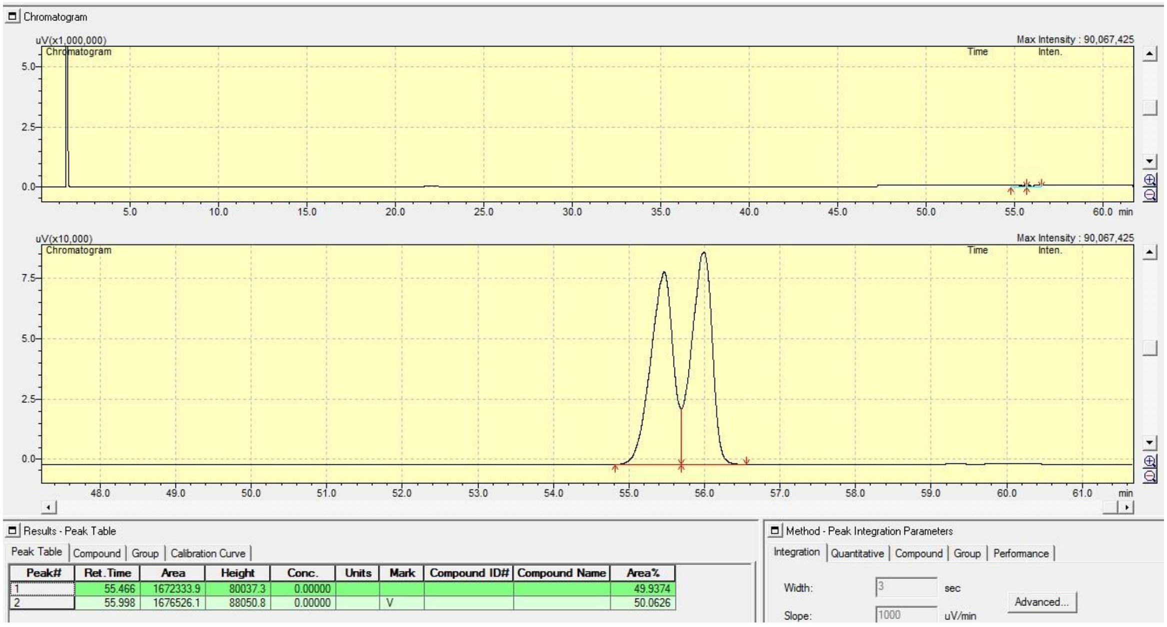

Spectrum of scalemic mixture (**5a**):

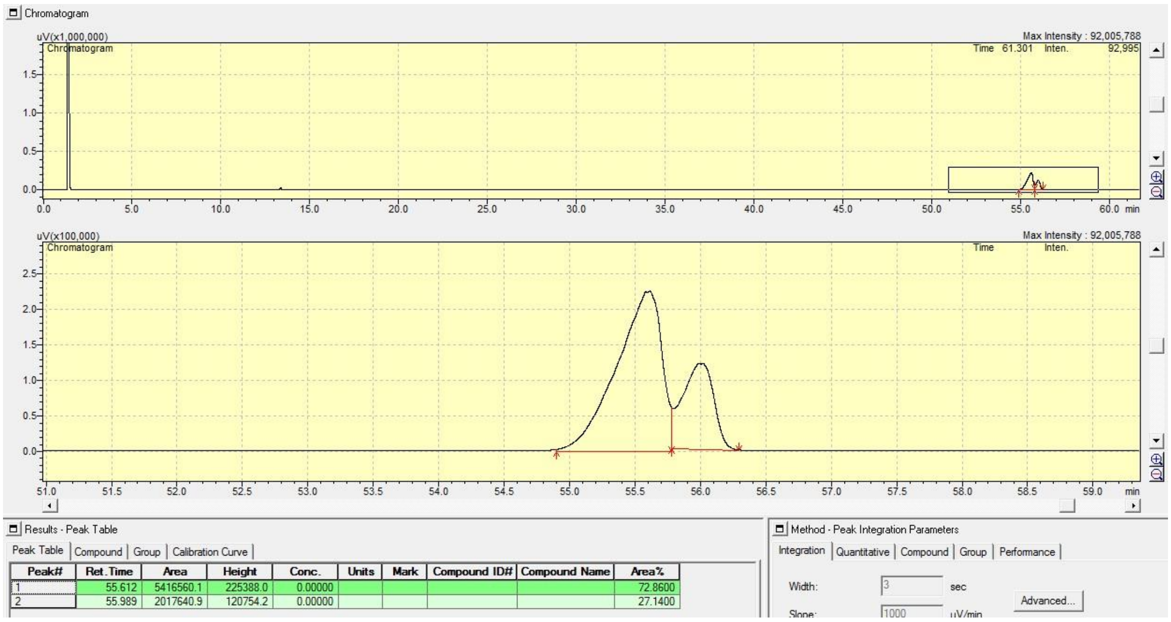

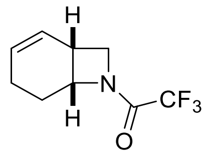

5b

General procedure F: from **4b** (57.2 mg, 0.2 mmol) with 10 mol% of [$Pd(PCy_3)_2$].

Yield: 10.1 mg, 25% (mixture of two conformers), Eluent: Pentane/DEE 98:2 to 97:3. Column chromatography was performed using 10% w/w $AgNO_3$ coated silica.

Rf: 0.18 (Pentane/DEE 95:5)

Aspect: yellow oil

Odor: floral

^{1}H NMR (500 MHz, $CDCl_3$): *mixture of conformers*: δ (ppm) 6.13 – 6.05 (m, 1H), 5.85 (dddd, *J* = 10.0, 5.3, 2.5, 1.0 Hz, 1H), 5.02 – 4.97 (m, 0.25H, *minor*), 4.90 ('dt', *J* = 7.6, 3.7 Hz, 0.75H, *major*), 4.54 (dddd, *J* = 9.1, 7.8, 1.3, 0.8 Hz, 0.75H, *major*), 4.31 ('t', *J* = 9.5 Hz, 0.25H, *minor*), 3.84 – 3.81 (m, 0.75H, *major*), 3.63 (dd, *J* = 10.2, 5.4 Hz, 0.25H, *minor*), 3.19 – 3.15 (m, 0.25H, *minor*), 3.14 – 3.08 (m, 0.75H, *major*), 2.47 – 2.42 (m, 0.75H, *major*), 2.21 – 2.08 (m, 2.25H), 1.72 – 1366 (m, 0.25H, *minor*), 1.47 (dddd, *J* = 14.7, 10.9, 5.8, 4.2 Hz, 0.75H, *major*).

^{13}C NMR (126 MHz, $CDCl_3$): δ (ppm) *major conformer*: 156.8 (q, *J* = 36.8 Hz), 131.0, 126.8, 116.1 (q, *J* = 288.7 Hz), 61.6, 58.6 (q, *J* = 2.1 Hz), 28.1, 22.9, 20.0.

^{19}F {^{1}H} NMR (235 MHz, $CDCl_3$): δ (ppm) -72.6 (*minor conformer*), -73.2 (*major conformer*).

IR (ATR): ν_{max} 2927, 1691, 1460, 1242, 1198, 1141 cm^{-1}.

HRMS: (ESI) m/z $[M + H]^+$ calculated for $C_9H_{10}F_3NNaO$: 228.0607; found: 228.0603.

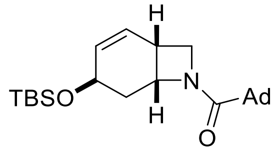

5c

General procedure F: from **4c** (96.5 mg, 0.2 mmol) with 0.06 mmol adamantine-1-carboxylic acid, 0.010 mmol Pd_2dba_3, 0.04 mmol tricyclohexylphosphine and 0.3 mmol potassium carbonate.

Yield: 71 mg, 88%, Eluent: (Cyclohexane/EtOAc 96:4). Column chromatography was performed using 10% w/w $AgNO_3$ loaded silica.

Rf: 0.15 (Cyclohexane/EtOAc 85:15)

Aspect: yellow oil

^{1}H NMR (500 MHz, $CDCl_3$): δ (ppm) 5.97 ('dt', J = 10.2, 1.5 Hz, 1H), 5.78 (ddd, J = 10.3, 5.4, 2.4 Hz, 1H), 4.78 ('dt', J = 7.2, 3.3 Hz, 1H), 4.50 (t, J = 8.2 Hz, 1H), 4.43 – 4.37 (m, 1H), 3.82 – 3.07 (m, 1H), 2.98 – 2.92 (m, 1H), 2.74 (dddd, J = 13.6, 5.4, 2.8, 1.3 Hz, 1H), 2.02 – 1.97 (m, 3H), 1.86 ('dt', J = 3.6, 1.8 Hz, 6H), 1.74 – 1.65 (m, 6H), 1.31 – 1.23 (m, 1H), 0.90 (s, 9H), 0.09 (s, 3H), 0.08 (s, 3H).

^{13}C NMR (126 MHz, CDCl$_3$): δ (ppm) 178.1, 137.0, 127.0, 64.6, 60.0, 58.9, 41.6, 38.5, 36.7, 33.6, 28.3, 28.1, 26.0, 18.4, -4.4, -4.7.

IR (ATR): ν_{max} 2905, 2853, 1623, 1389, 1091, 874, 837 cm^{-1}.

HRMS: (ESI) m/z [M + NH$_4$]$^+$ calculated for $C_{24}H_{43}N_2O_2Si$: 419.3088; found: 419.3098.

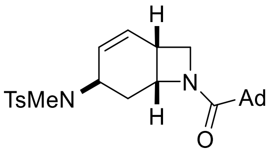

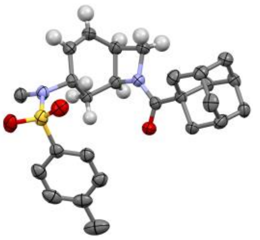

5d

General procedure F: from **4d** (53.6 mg, 0.1 mmol)

Yield: 33.6 mg, 74%, Eluent: Cyclohexane/EtOAc 95:5 to 80:20)

Rf: 0.17 (Cyclohexane/EtOAc 60:40)

Aspect: off-white solid, m.p. 150.2 – 154.0 °C

^{1}H NMR (500 MHz, CDCl$_3$): δ (ppm) 7.65 (d, *J* = 8.3 Hz, 2H), 7.31 (d, *J* = 8.0 Hz, 2H), 5.92 (ddd, *J* = 10.3, 5.4, 2.9 Hz, 1H), 5.81 (dt, *J* = 10.3, 1.6 Hz, 1H), 4.73 (ddt, *J* = 9.6, 4.7, 1.8 Hz, 1H), 4.69 (dt, *J* = 6.9, 3.1 Hz, 1H), 4.50 (t, *J* = 8.1 Hz, 1H), 3.81 (dd, *J* = 7.9, 3.9 Hz, 1H), 2.90 (dddd, *J* = 9.2, 7.2, 3.8, 1.4 Hz, 1H), 2.67 (s, 3H), 2.41 (s, 3H), 2.23 (dddd, *J* = 13.3, 4.7, 2.7, 1.3 Hz, 1H), 2.03 – 2.00 (m, 3H), 1.93 – 1.91 (m, 1H), 1.90 (dt, *J* = 3.5, 2.0 Hz, 2H), 1.88 (dt, *J* = 3.6, 2.0 Hz, 2H), 1.86 – 1.82 (m, 1H), 1.74 – 1.73 (m, 1H), 1.71 – 1.68 (m, 4H), 1.68 – 1.66 (m, 1H), 1.11 (ddd, *J* = 13.2, 11.4, 3.7 Hz, 1H).

^{13}C NMR (126 MHz, CDCl$_3$): δ (ppm) 178.5, 143.4, 136.5, 132.9, 130.8, 129.9, 127.5, 60.0, 58.5, 50.7, 41.6, 39.4, 38.5, 36.7, 29.4, 28.2, 27.8, 21.7.

IR (ATR): ν_{max} 2906, 2852, 1616, 1453, 1336, 1164, 732, 655 cm^{-1}.

HRMS: (ESI) m/z [M + H]$^+$ calculated for $C_{26}H_{35}N_2O_3S$: 455.2363; found: 455.2364.

C–H activation products – Oxetanation

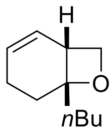

5e

General procedure F: from **4e** (49.4 mg, 0.2 mmol with 0.06 mmol AdCOOH, 0.01 mmol Pd_2dba_3, 0.04 mmol PCy_3 and 0.3 mmol K_2CO_3)

Yield: 25 mg, 75% (95% nmry), Eluent: Pentane/DEE 100:0 to 95:5. NOTE: **5e** has a low boiling point. Removal of solvents after column chromatography was performed with co-evaporation with $CDCl_3$ at least four times on a rotary evaporator at min 350 mbar pressure and at max 45 °C. Isolated yield was calculated using trichloroethylene as external standard.

Rf: 0.37 (Pentane/DEE 90:10)

Aspect: yellow oil

^{1}H NMR (500 MHz, $CDCl_3$): δ (ppm) 6.05 (ddd, *J* = 9.5, 6.6, 2.5 Hz, 1H), 5.86 (ddd, *J* = 9.8, 5.8, 3.0 Hz, 1H), 4.76 (dd, *J* = 7.9, 5.3 Hz, 1H), 3.97 (t, *J* = 5.1 Hz, 1H), 2.96 – 2.90 (m, 1H), 2.47 – 2.38 (m, 1H), 2.16 – 2.08 (m, 1H), 1.82 – 1.76 (m, 1H), 1.74 – 1.72 (m, 1H), 1.71 – 1.69 (m, 1H), 1.37 (dddd, *J* = 11.7, 6.7, 5.8, 3.4 Hz, 4H), 1.23 (ddd, *J* = 14.1, 11.5, 5.1 Hz, 1H), 0.92 (t, *J* = 7.0 Hz, 3H).

^{13}C NMR (101 MHz, $CDCl_3$): δ (ppm) 130.0, 128.5, 88.1, 75.2, 41.3, 37.1, 30.6, 25.6, 23.4, 21.4, 14.2.

IR (ATR): ν_{max} 3028, 2956, 2933, 973 cm^{-1}.

HRMS: data for this compound could not be obtained due to poor ionization. Instead the GC/MS trace is provided among with major fragments.

GC/MS: Rt = 4.433 min

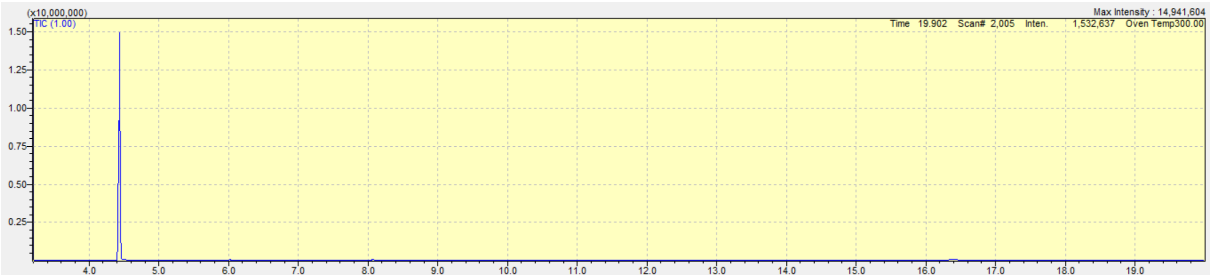

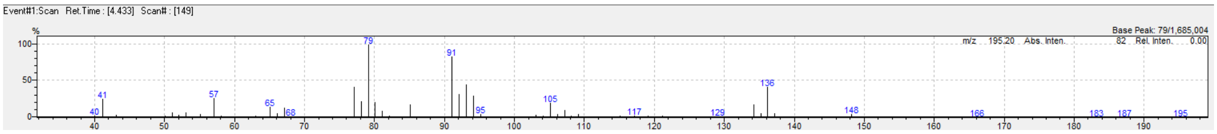

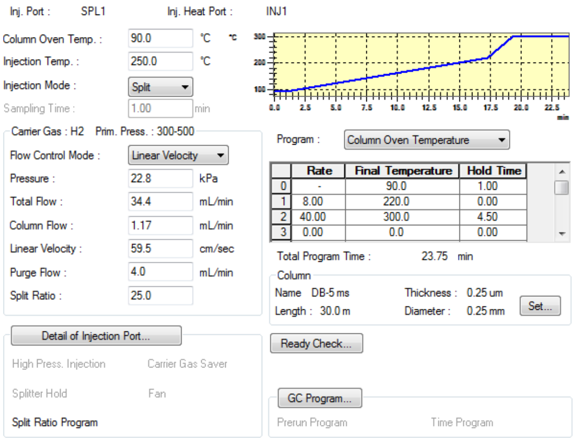

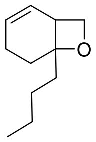

Chemical Formula: $C_{11}H_{18}O$
Exact Mass: 166.1358

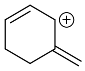

Chemical Formula: $C_7H_9^+$
Exact Mass: 93.0699

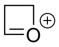

Chemical Formula: $C_3H_5O^+$
Exact Mass: 57.0335

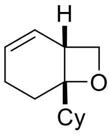

5f

General procedure F: from **4f** (27.3 mg, 0.1 mmol)

Yield: 16.2 mg, 84% (92% nmry), Eluent: Pentane/DEE 100:0 to 98:2. NOTE: **5f** has a low boiling point. Removal of solvents after column chromatography was performed with co-evaporation with $CDCl_3$ at least four times on a rotary evaporator at min 160 mbar pressure and at max 45 °C. Isolated yield was calculated using trichloroethylene as external standard.

Rf: 0.23 (Pentane/DEE 95:5)

Aspect: yellow oil

^{1}H NMR (500 MHz, $CDCl_3$): δ (ppm) 6.07 (ddd, J = 9.6, 6.9, 2.4 Hz, 1H), 5.88 (ddd, J = 9.6, 6.1, 3.1 Hz, 1H), 4.72 (dd, J = 8.1, 5.3 Hz, 1H), 3.98 (t, J = 5.2 Hz, 1H), 2.98 (dddd, J = 7.9, 6.2, 5.0, 1.1 Hz, 1H), 2.50 – 2.37 (m, 1H), 2.15 (dddd, J = 16.4, 7.2, 5.1, 2.5 Hz, 1H), 1.88 – 1.84 (m, 1H), 1.82 – 1.80 (m, 1H), 1.80 – 1.75 (m, 2H), 1.73 – 1.68 (m, 2H), 1.66 – 1.61 (m, 1H), 1.29 – 1.26 (m, 1H), 1.25 – 1.24 (m, 1H), 1.24 – 1.19 (m, 1H), 1.17 – 1.13 (m, 1H), 1.06 – 0.99 (m, 1H), 0.98 – 0.91 (m, 1H).

^{13}C NMR (126 MHz, $CDCl_3$): δ (ppm) 130.50, 128.87, 90.54, 75.59, 48.18, 35.85, 26.82 (2C), 26.78, 26.66, 26.59, 26.20, 21.33.

IR (ATR): ν_{max} 3028, 2924, 2853, 975 cm^{-1}.

HRMS: data for this compound could not be obtained due to poor ionization. Instead the GC/MS trace is provided among with major fragments.

GC/MS: Rt = 8.258 min

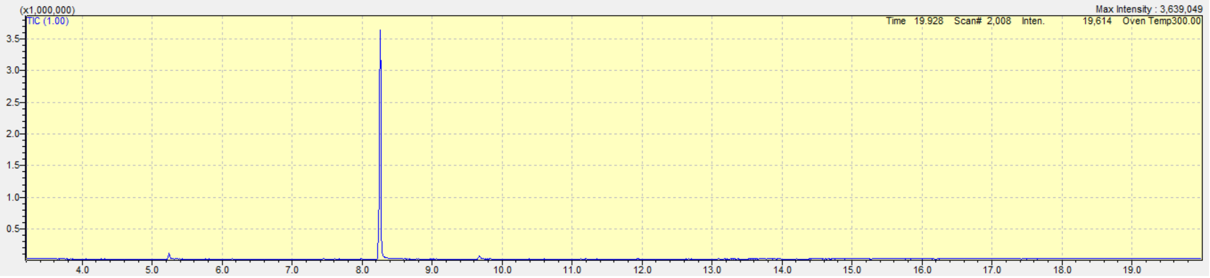

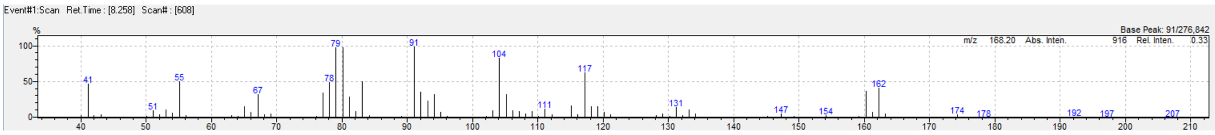

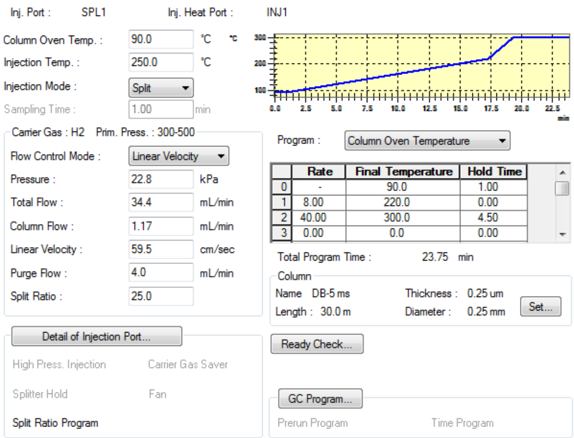

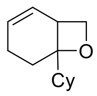

Chemical Formula: $C_{13}H_{20}O$
Exact Mass: 192.1514

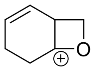

Chemical Formula: $C_7H_9O^+$
Exact Mass: 109.0648

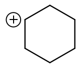

Chemical Formula: $C_6H_{11}^+$
Exact Mass: 83.0855

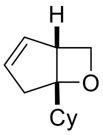

5g

General procedure F: from **4g** (51.8 mg, 0.2 mmol with 0.06 mmol AdCOOH, 0.01 mmol Pd_2dba_3, 0.04 mmol PCy_3 and 0.3 mmol K_2CO_3)

Yield: 27.5 mg, 70% (88% nmry), Eluent: Pentane/DEE 100:0 to 98:2. Column chromatography was performed using 10% w/w $AgNO_3$ coated silica. NOTE: **5g** has a low boiling point. Removal of solvents after column chromatography was performed with co-evaporation with $CDCl_3$ at least four times on a rotary evaporator at min 200 mbar pressure and at max 45 °C. Isolated yield was calculated using trichloroethylene as external standard.

Rf: 0.28 (Pentane/DEE 90:10)

Aspect: yellow oil

^{1}H NMR (500 MHz, $CDCl_3$): δ (ppm) 5.95 – 5.93 (m, 1H), 5.87 – 5.84 (m, 1H), 4.74 – 4.71 (m, 1H), 4.09 (dd, J = 5.5, 3.5 Hz, 1H), 3.34 – 3.30 (m, 1H), 2.55 – 2.49 (m, 1H), 2.45 – 2.40 (m, 1H), 1.93 –1.88 (m, 1H), 1.84 – 1.78 (m, 2H), 1.75 (dt, J = 11.9, 3.1 Hz, 1H), 1.73 – 1.67 (m, 2H), 1.32 – 1.27 (m, 1H), 1.27 – 1.23 (m, 1H), 1.22 – 1.12 (m, 2H), 1.09 – 1.01 (m, 1H).

^{13}C NMR (126 MHz, $CDCl_3$): δ (ppm) 132.4, 131.6, 98.6, 76.4, 46.6, 44.6, 43.3, 26.8, 26.8, 26.6, 26.4, 26.3.
IR (ATR): ν_{max} 3052, 2926, 2855, 972 cm^{-1}.

HRMS: data for this compound could not be obtained due to poor ionization. Instead the GC/MS trace is provided among with major fragments.

GC/MS: Rt = 6.325 min

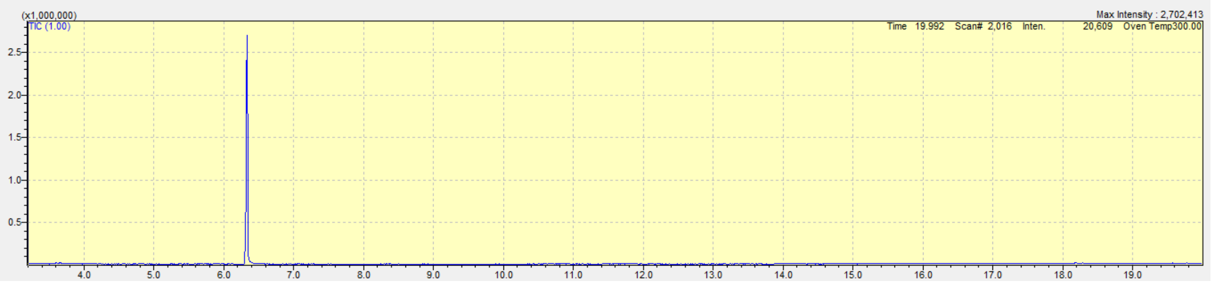

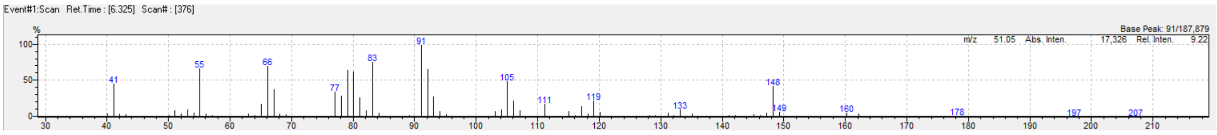

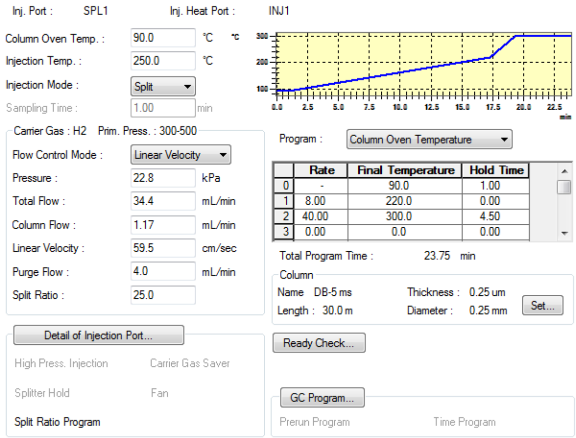

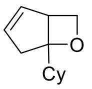

Chemical Formula: $C_{12}H_{18}O$
Exact Mass: 178.1358

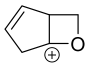

Chemical Formula: $C_6H_7O^+$
Exact Mass: 95.0491

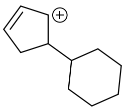

Chemical Formula: $C_{11}H_{17}^+$
Exact Mass: 149.1325

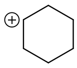

Chemical Formula: $C_6H_{11}^+$
Exact Mass: 83.0855

Unsuccessful substrates

- In the case of cycloheptenyl precursors no C(sp^3)–H activation was observed. Instead a 1,3-diene was formed as described below.

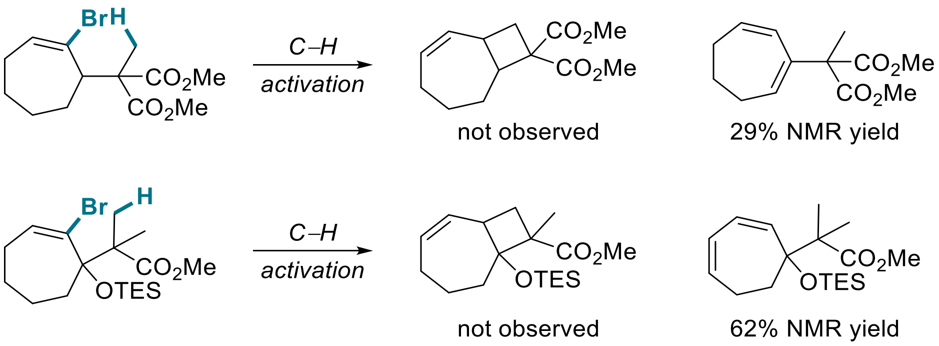

- In the case of cyclohexenyl precursors bearing an α-electron donating group in respect to the CH_3 no C(sp^3)–H activation was observed. As before, a 1,3-diene was formed.

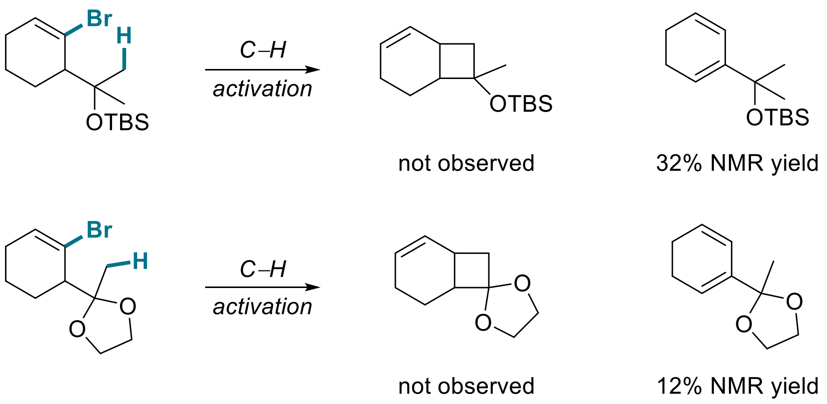

- C(sp^3)–H methylene activation proceeded with quite low yields (~20%) and many side products (mostly the protodehalogenated analogue) were formed.

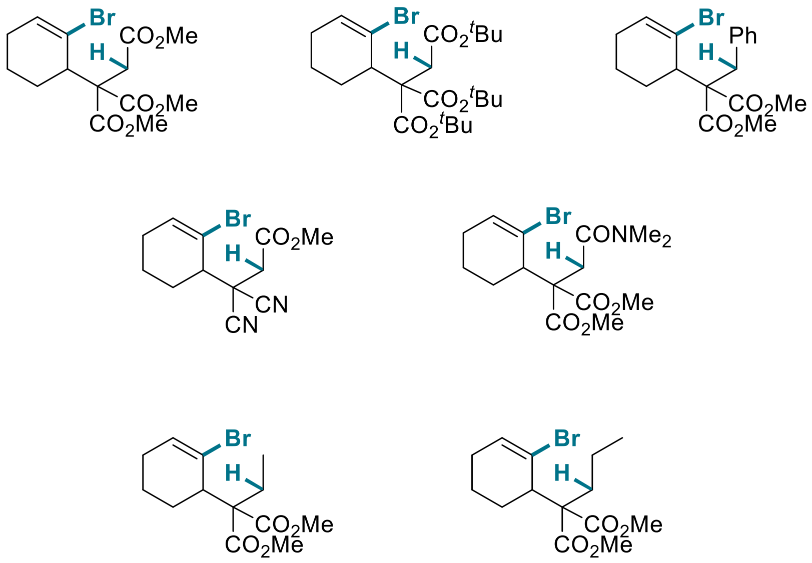

- Oxetanes

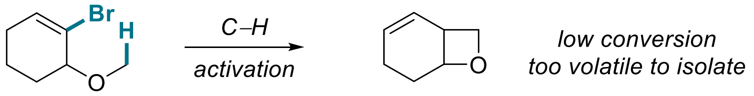
Br
H
O
C–H
activation
O
low conversion
too volatile to isolate

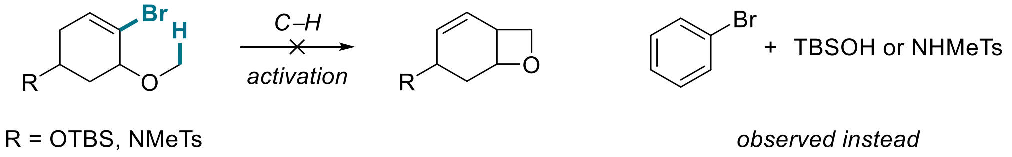
Br
H
R
O
C–H
activation
R
O
Br
+ TBSOH or NHMeTs
R = OTBS, NMeTs
observed instead

Kinetic Studies

Determination of Orders

General reaction for Kinetic Studies

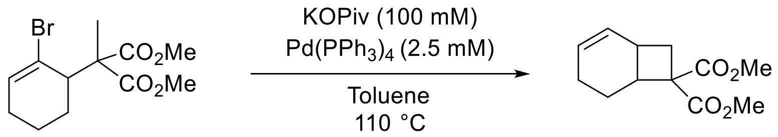

1g 50 mM **2g**

Potassium pivalate (28 mg, 0.20 mmol, 2 eq,) was weighted out in nine 10 ml Pyrex tubes (28 mg/each) in an argon filled glovebox. Two stock solutions were prepared as follows: A. In an argon filled glovebox **1g** (305 mg, 1.0 mmol, 10 eq) was weighted out in a 10 ml volumetric flask, toluene was added up to 10 ml and the flask was left to stir until complete dilution. B. In an argon filled glovebox $Pd(PPh_3)_4$ (57.8 mg, 0.05 mmol, 50 mol %) was weighted out in a 10 ml volumetric flask, toluene was added up to 10 ml and the flask was left to stir until complete dilution. With the use of 1 ml syringes 1 ml out of each stock solution (A and B) were added into each tube containing potassium pivalate (total of 2 ml of solution/tube). The tubes were sealed with a screw cup and placed in a heating block preheated at 110 °C at 800 rpm. Tubes were removed from the heat in different time points throughout the first 3 h of the reaction (after removal each tube was submerged into an ice bath). Each crude mixture was filtered through celite®. The celite® was washed with 10 ml of EtOAc and the liquors were evaporated under reduced pressure. A stock solution of internal standard was prepared: C. 1,3,5-Trimethoxybenzene (138 mg, 1 mmol, 10 eq) was weighted out in a 10 ml volumetric flask, CD_3Cl was added up to 10 ml and the flask was left to stir until complete dilution. Each evaporated crude mixture was dissolved into 1 ml of stock solution C. The progress of the reaction was then monitored via ^1H NMR, using 1,3,5-trimethoxybenzene as internal standard.

Determination of Order in 1g

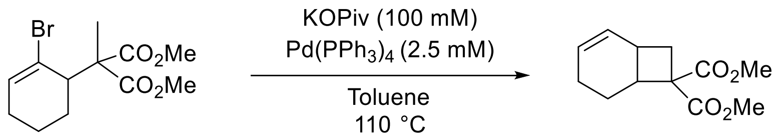

1g **50 mM/75 mM** **2g**

The order in **1g** has been determined using normalized time scale analysis. Two sets of reactions were carried out with two different concentrations of starting material (50 and 75 mM) and their temporal profiles were normalized according to **1g** raised to the power of the order in **1g**. Curves of different orders were plotted together and the correct order is the one that leads to overlaying curves. The overlaying curves in the reaction profiles in Figure S1(b) indicates an order of 0 in **1g** (experiments for 75 mM were run in duplicate, standard deviation of the two runs is indicated with error bars).

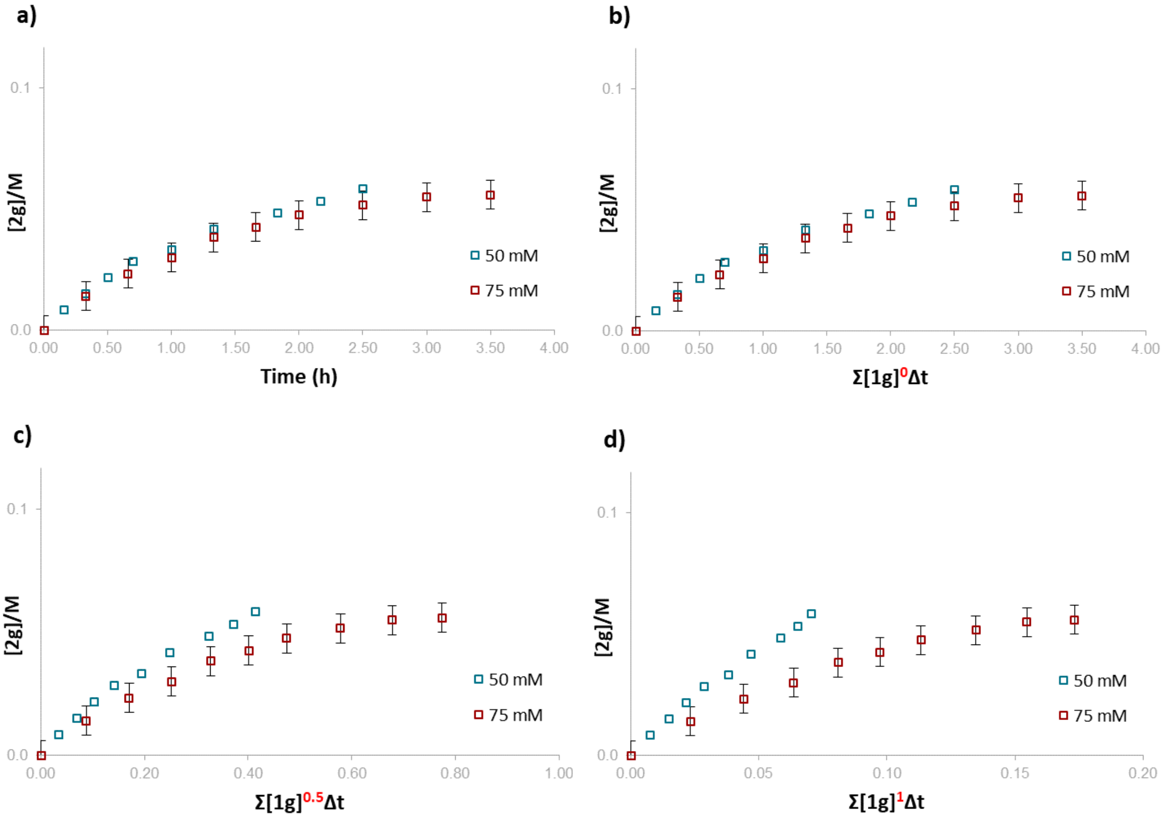

Figure S1. Order in 1g (standard deviation of the two runs is indicated with error bars). (a) Reaction profiles for the reactions carried out with 50/75 mM of 1g. (b) Normalized time scale profile for order 0 in [1g]. (c) Normalized time scale profile for order 0.5 in [1g]. (d) Normalized time scale profile for order 1 in [1g].

Table S3. VTNA data for the calculation of order in 1g.

Time (h)	[2g] for [1g]=50 mM (mM)	Time (h)	[2g] for [1g]=75 mM (mM)*
0.16	**5**	0.33	**8.5**
0.33	**9**	0.66	**14**
0.50	**13**	1.00	**18**
0.70	**17**	1.33	**23**
1.00	**20**	1.66	**25.5**
1.33	**25**	2.00	**28.5**
1.83	**29**	2.50	**31**
2.17	**32**	3.00	**33**
2.50	**35**	3.50	**33.5**

*average of two runs

Determination of Order in catalyst

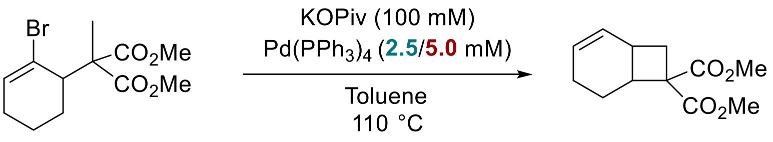

1g 50 mM   **2g**

The order in catalyst has been determined using normalized time scale analysis. Two sets of reactions were carried out with two different concentrations of catalyst (2.5 and 5.0 mM) and their temporal profiles were normalized according to catalyst raised to the power of the order in catalyst. Curves of

different orders were plotted together and the correct order is the one that leads to overlaying curves. The overlaying curves in the reaction profiles in Figure S2(d) indicates an order of 1 in catalyst

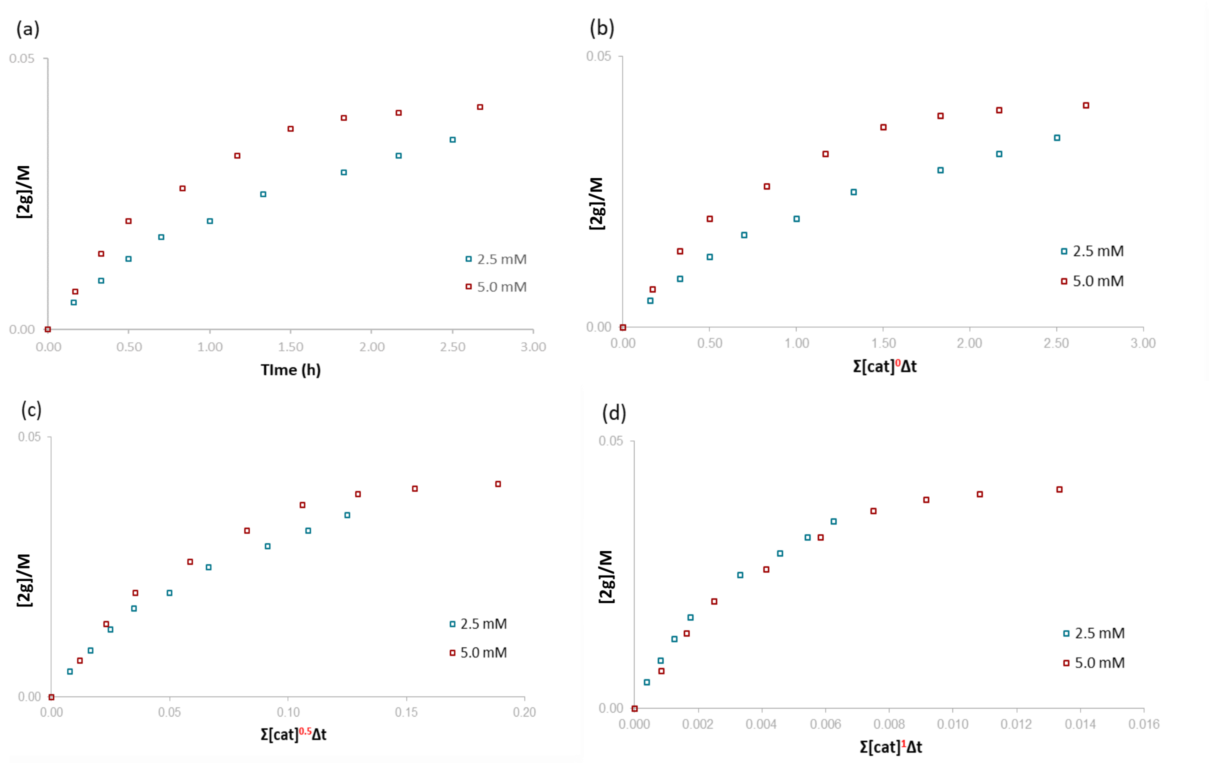

Figure S2. Order in catalyst. (a) Reaction profiles for the reactions carried out with 2.5/5.0 mM of catalyst. (b) Normalized time scale profile for order 0 in [cat]. (c) Normalized time scale profile for order 0.5 in [cat]. (d) Normalized time scale profile for order 1 in [cat].

Table S4. VTNA data for the calculation of order in catalyst.

Time (h)	[2g] for [cat]=2.5 mM (mM)	Time (h)	[2g] for [cat]=5.0 mM (mM)
0.16	**5**	0.17	**7**
0.33	**9**	0.33	**14**
0.50	**13**	0.50	**20**
0.70	**17**	0.83	**26**
1.00	**20**	1.17	**32**
1.33	**25**	1.50	**37**
1.83	**29**	1.83	**39**
2.17	**32**	2.17	**40**
2.50	**35**	2.67	**41**

Determination of Order in base

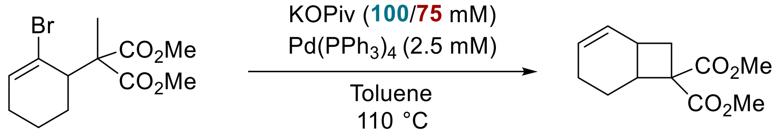

1g
50 mM

2g

The order in base has been determined using normalized time scale analysis. Two sets of reactions were carried out with two different concentrations of base (100 and 75 mM) and their temporal profiles were normalized according to base raised to the power of the order in base. Curves of different

orders were plotted together and the correct order is the one that leads to overlaying curves. The overlaying curves in the reaction profiles in Figure S3(b) indicates an order of 0 in base. However, this is only an apparent order attributed to low solubility of KOPiv in toluene. Two tubes containing only toluene and KOPiv in two different concentrations (100 and 75 mM) were placed at 120 °C at 800 rpm. In both cases the solutions were turbid even after 16 h of heating.

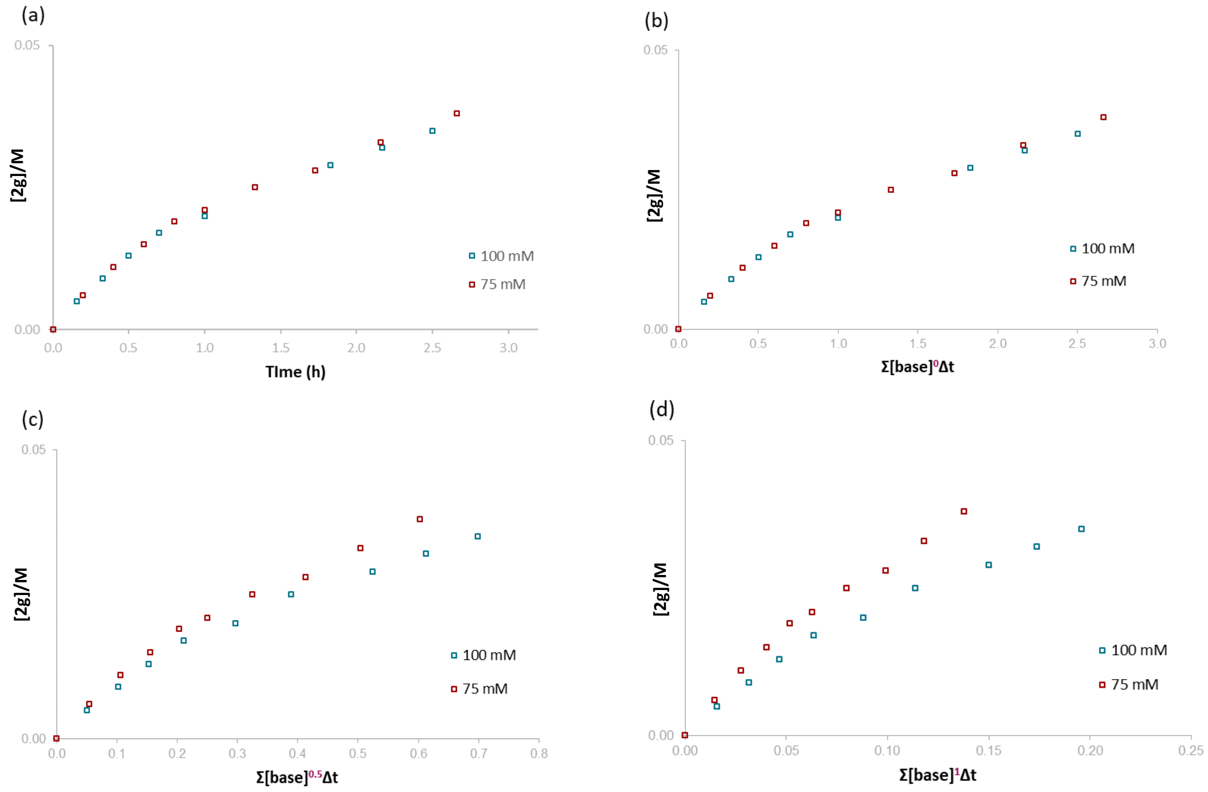

Figure S3. Order in base. (a) Reaction profiles for the reactions carried out with 100/75 mM of base. (b) Normalized time scale profile for order 0 in [base]. (c) Normalized time scale profile for order 0.5 in [base]. (d) Normalized time scale profile for order 1 in [base].

Table S5. VTNA data for the calculation of order in base.

Time (h)	[2g] for [base]=100 mM (mM)	Time (h)	[2g] for [base]=75 mM (mM)
0.16	**5**	0.20	**6**
0.33	**9**	0.40	**11**
0.50	**13**	0.60	**15**
0.70	**17**	0.80	**19**
1.00	**20**	1.00	**21**
1.33	**25**	1.33	**25**
1.83	**29**	1.73	**28**
2.17	**32**	2.16	**33**
2.50	**35**	2.66	**38**

Determination of Kinetic Isotope Effect (KIE)

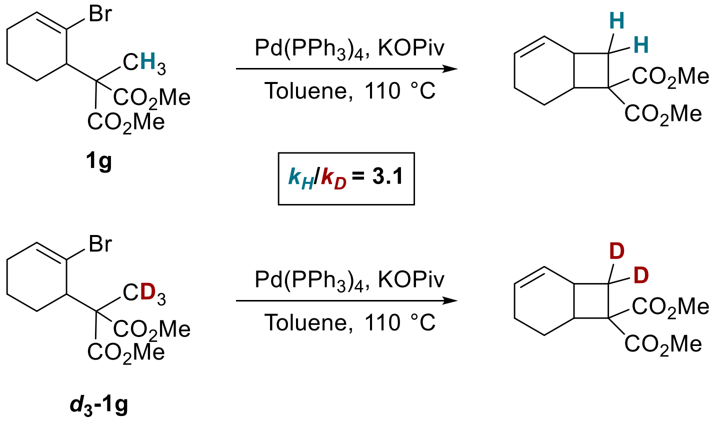

The KIE in starting material has been determined using initial reaction profiles of two parallel experiments. Two sets of reactions were carried out with two different starting materials (**1g** and ***d*$_3$-1g**). The same protocol as in the calculation of orders was followed. The same protocol was repeated for ***d*$_3$-1g** (308 mg, 1.0 mmol, 10 eq). Tubes were removed from the heat in different time points throughout the first 30 min of the reaction (after removal each tube was submerged into an ice bath). Each crude mixture was filtered through celite®. The celite® was washed with 10 ml of EtOAc and the liquors were evaporated under reduced pressure. A stock solution of internal standard was prepared: D. Trichloroethylene (0.09 ml, 1.0 mmol, 10 eq) was added in a 10 ml volumetric flask, CD_3Cl was added up to 10ml and the flask was left to slowly stir. Each evaporated crude mixture was dissolved into 1 ml of stock solution D. The progress of each set of reactions was then monitored via ^{1}H NMR, using trichloroethylene as internal standard. KIE was calculated from the linear equations of the initial rate kinetic profiles of the two substrates but also using normalized time scale analysis. Curves of different values of KIE were plotted together and the correct KIE is the one that leads to overlaying curves. The overlaying curves in the reaction profiles in Figure S4(b) indicates a KIE value of 3. A similar number was calculated through the linear equations (KIE = 3.1). Strong primary KIE indicates that C–H activation is the rate limiting step.

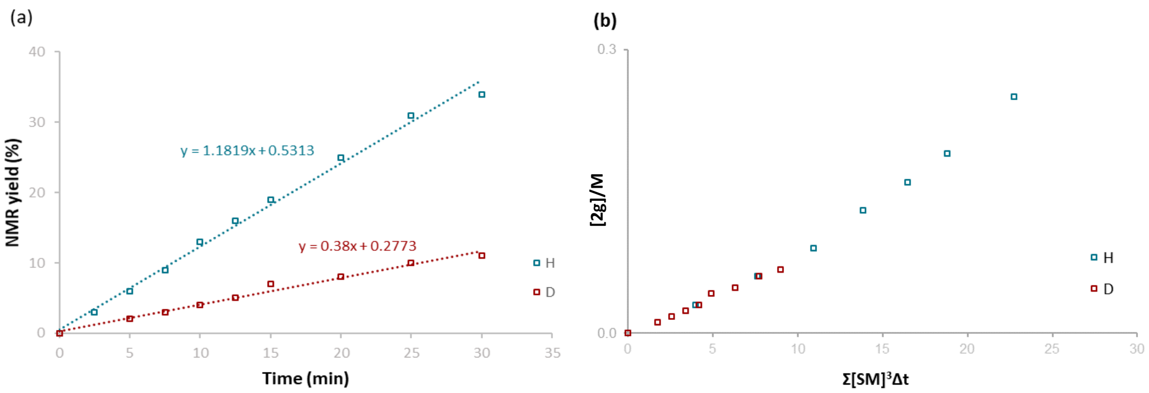

Figure S4. Determination of KIE in starting material (SM). (a) Initial reaction profiles for 1g and d_3-1g. (b) Normalized time scale profile for KIE = 3 in [SM].

Table S6. Initial rate kinetic data for the KIE.

Time (min)	NMR yield for 2g (%)	NMR yield for d_2-2g (%)
2.5	**3**	**-**
5.0	**6**	**2**
7.5	**9**	**3**
10	**13**	**4**
12.5	**16**	**5**
15	**19**	**7**
20	**25**	**8**
25	**31**	**10**
30	**34**	**11**

Crystallographic data

CheckCIF/PLATON report of:

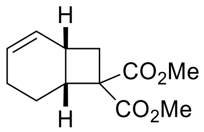

2g

```
Bond precision:    C-C = 0.0022 A            Wavelength=1.34143

Cell:              a=5.7275(1)      b=14.6915(3)       c=13.6745(2)
                   alpha=90         beta=91.884(1)     gamma=90
Temperature:       150 K

                   Calculated                 Reported
Volume             1150.03(4)                 1150.03(4)
Space group        P 21/c                     P 1 21/c 1
Hall group         -P 2ybc                    -P 2ybc
Moiety formula     C12 H16 O4                 C12 H16 O4
Sum formula        C12 H16 O4                 C12 H16 O4
Mr                 224.25                     224.25
Dx,g cm-3          1.295                      1.295
Z                  4                          4
Mu (mm-1)          0.511                      0.511
F000               480.0                      480.0
F000'              481.23
h,k,lmax           7,18,16                    7,18,16
Nref               2239                       2224
Tmin,Tmax          0.862,0.955                0.025,0.263
Tmin'              0.849

Correction method= # Reported T Limits: Tmin=0.025 Tmax=0.263
AbsCorr = MULTI-SCAN

Data completeness= 0.993            Theta(max)= 55.629

R(reflections)= 0.0636( 2007)                     wR2(reflections)=
                                                  0.1829( 2224)
S = 1.048                    Npar= 147
```

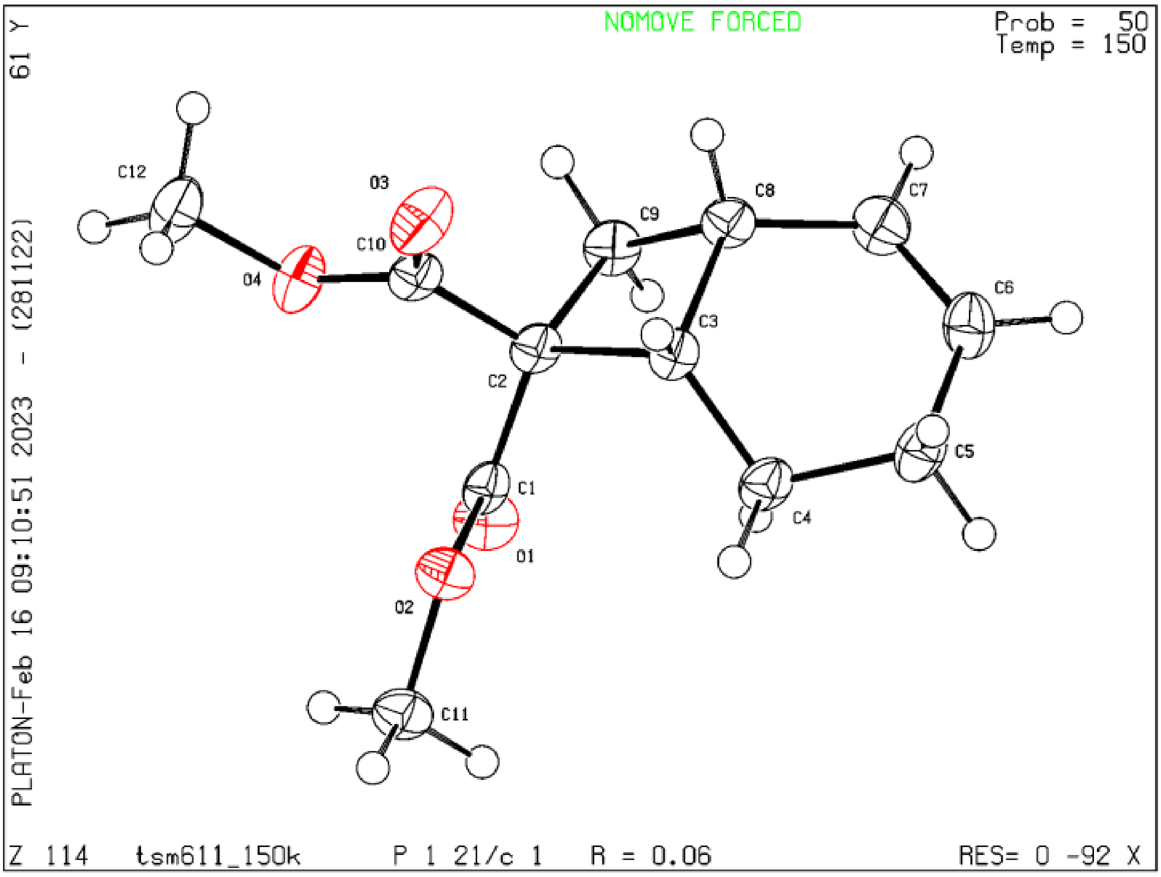
NOMOVE FORCED
Prob = 50
Temp = 150
61 Y
PLATON-Feb 16 09:10:51 2023 - (281122)
C12
O3
C10
O4
C9
C8
C7
C6
C3
C2
C5
C1
C4
O1
O2
C11
Z 114
tsm611_150k
P 1 21/c 1
R = 0.06
RES= 0 -92 X

CheckCIF/PLATON report of:

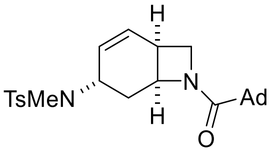

5d

Bond precision:	C-C = 0.0026 A		Wavelength=1.54186
Cell:	a=19.1522(3)	b=11.6030(1)	c=21.5455(4)
	alpha=90	beta=106.219(1)	gamma=90
Temperature:	150 K		

	Calculated	Reported
Volume	4597.35(12)	4597.35(12)
Space group	I 2/a	I 1 2/a 1
Hall group	-I 2ya	-I 2ya
Moiety formula	C26 H33 N2 O3 S	C26 H33 N2 O3 S
Sum formula	C26 H33 N2 O3 S	C26 H33 N2 O3 S
Mr	453.60	453.60
Dx,g cm-3	1.311	1.311
Z	8	8
Mu (mm-1)	1.494	1.494
F000	1944.0	1944.0
F000'	1951.84	
h,k,lmax	23,14,26	23,14,26
Nref	4387	4351
Tmin,Tmax	0.756,0.799	0.026,0.129
Tmin'	0.686	

Correction method= # Reported T Limits: Tmin=0.026 Tmax=0.129
AbsCorr = MULTI-SCAN

Data completeness= 0.992 Theta(max)= 70.289

R(reflections)= 0.0465(4046) wR2(reflections)= 0.1388(4351)

S = 1.081 Npar= 364

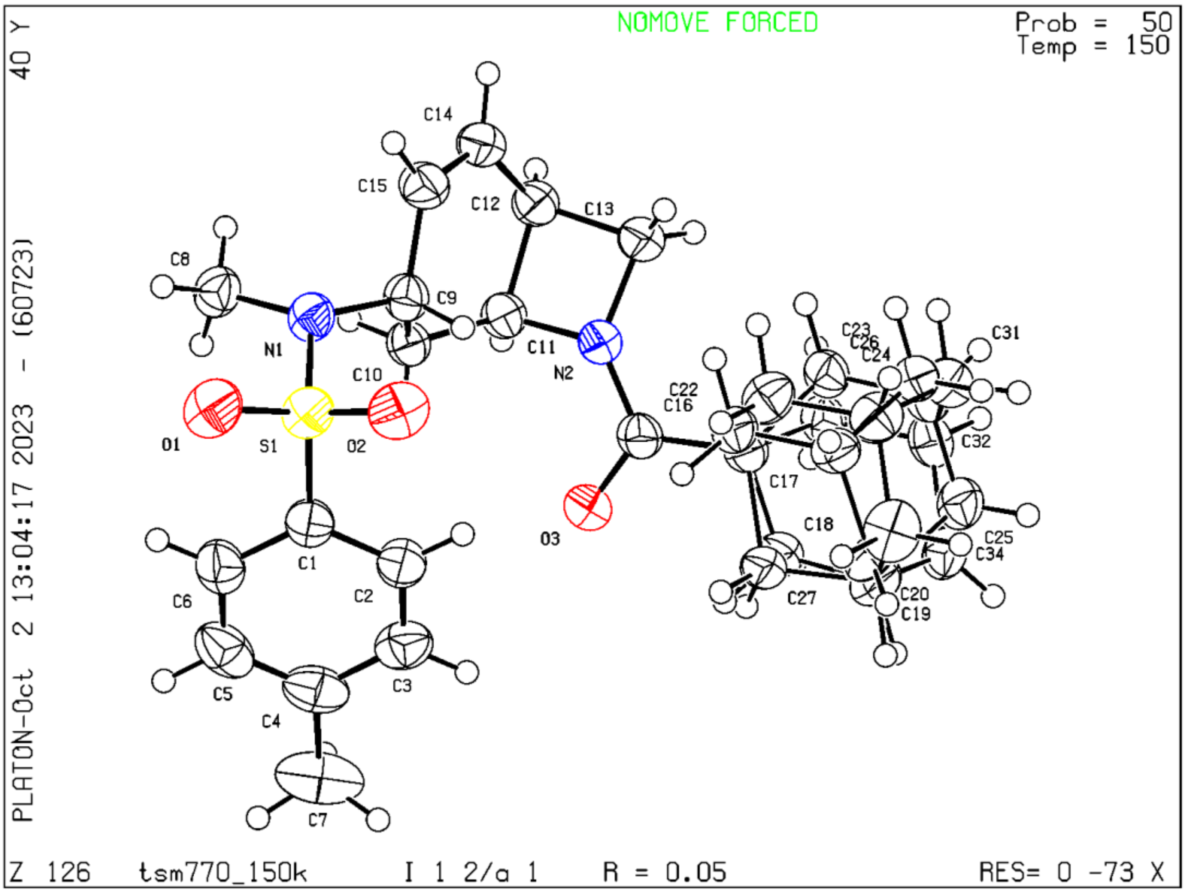

NOMOVE FORCED
Prob = 50
Temp = 150
40 Y
PLATON-Oct 2 13:04:17 2023 - (60723)
C14
C15
C12
C13
C8
C9
N1
C11
N2
C10
C22
C16
C23
C26
C24
C31
O1
S1
O2
C32
C17
O3
C18
C25
C34
C1
C6
C2
C27
C20
C19
C5
C3
C4
C7
Z 126 tsm770_150k I 1 2/a 1 R = 0.05 RES= 0 -73 X

References

(1) Thesmar, P.; Coomar, S.; Prescimone A.; Häussinger, D.; Gillingham, D.; Baudoin, O. Divergent Synthesis of Bioactive Dithiodiketopiperazine Natural Products Based on a Double C(sp^3)–H Activation Strategy. *Chem. Eur. J.* **2020**, *26*, 15298–15312.

(2) Demircan, A. A Study of Palladium Catalyzed Intra/Intermolecular Cascade Cross Coupling/Cyclizations Involving Bicyclopropylidene. *Molecules* **2014**, *19*, 6058–6069.

(3) Dailler, D.; Danoun, G.; Ourri, B.; Baudoin, O. Divergent Synthesis of Aeruginosins Based on a C(sp^3)–H Activation Strategy. *Chem. Eur. J.* **2015**, *21*, 9370–9379.

(4) Li, K.; Alexakis, A. Asymmetric Conjugate Addition to α-Haloenones: Dramatic Effect of Styrene on the Enantioselectivity. *Angew. Chem. Int. Ed*. **2006**, *45*, 7600–7603.

(5) Ramadhar, R. T.; Kawakami, J.; Batey A. R. Sequential O-Arylation/Lanthanide(III)-Catalyzed [3,3]-Sigmatropic Rearrangement of Bromo-Substituted Allylic Alcohols. *Synlett* **2017**, *28*, 2865–2870.

(6) Algia, F.; Hökelekc, T.; Balci, M. Addition of dibromocarbene to cyclobutene: characterisation and mechanism of formation of the products. *J. Chem. Res.* **2004**, *10*, 658–660.

^{1}H, ^{13}C and ^{19}F NMR spectra

C–H activation substrates

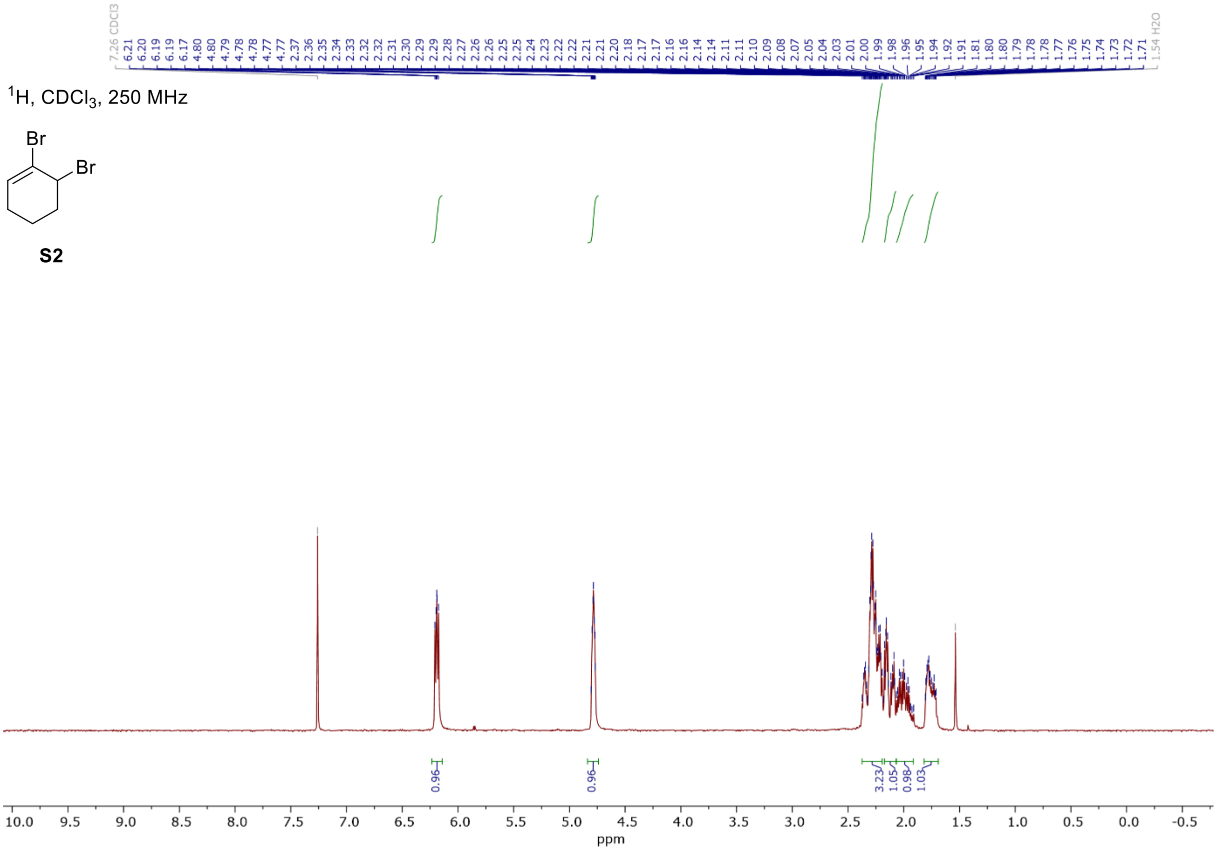

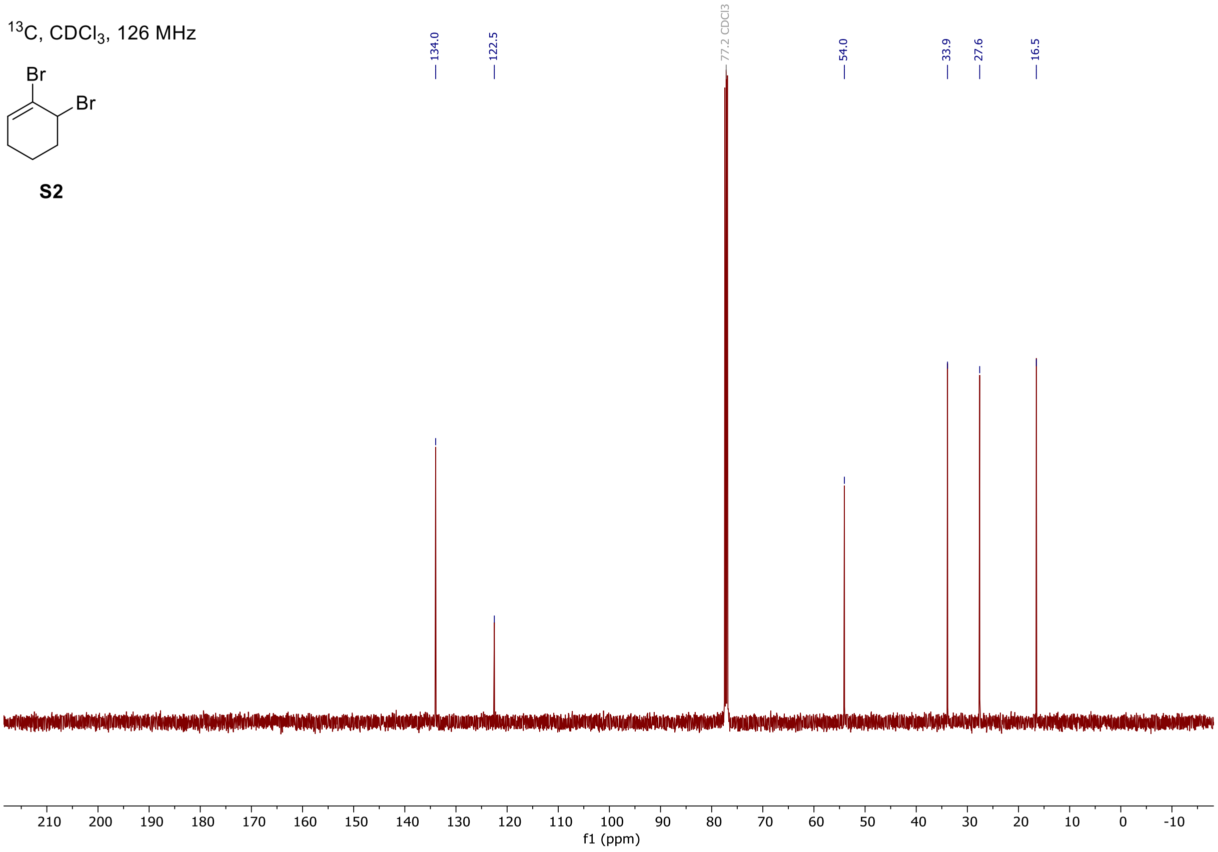

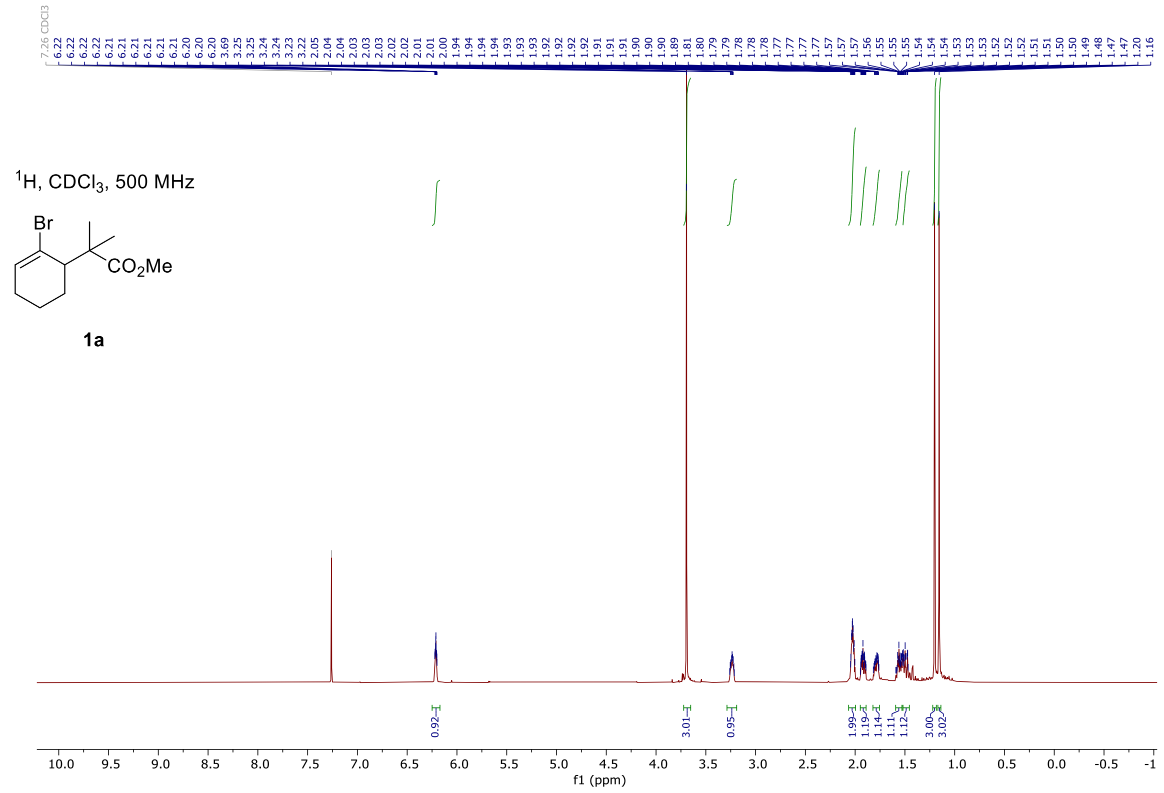
1H, CDCl3, 500 MHz
Br
CO2Me
1a
7.26 CDCl3
0.92
3.01
0.95
1.99
1.19
1.14
1.11
1.12
3.00
3.02
f1 (ppm)

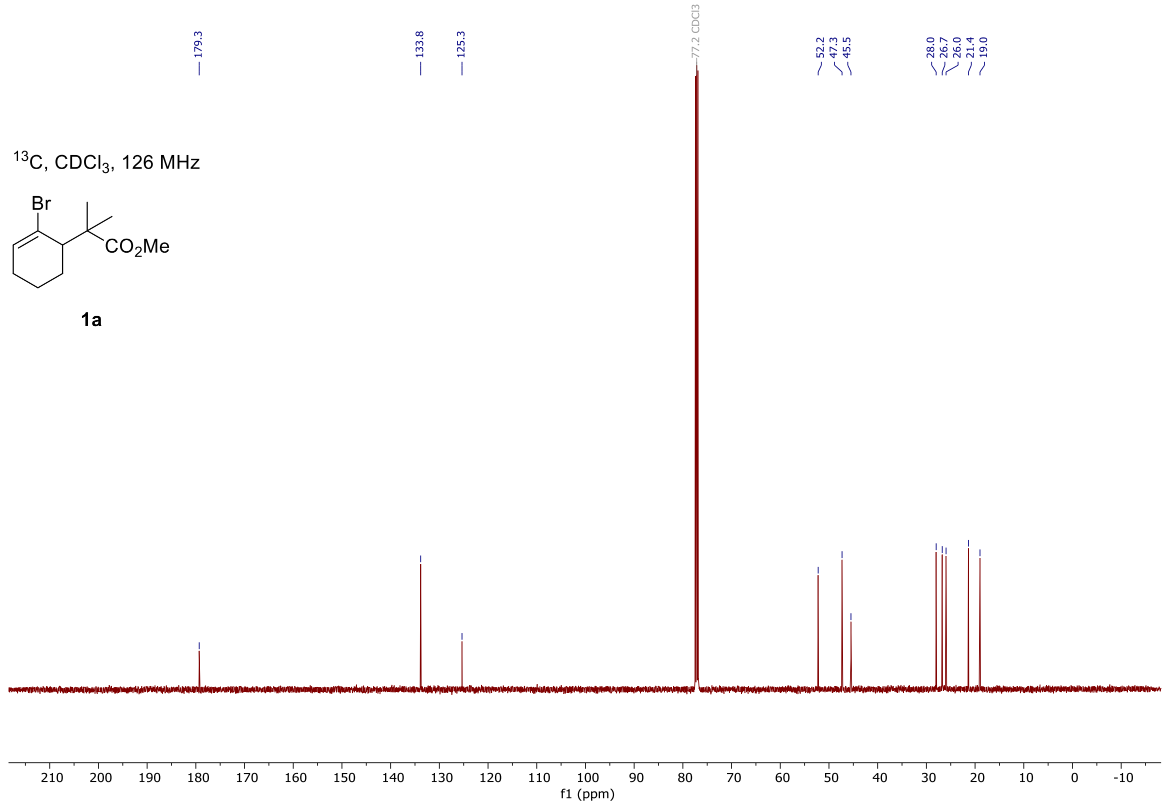
13C, CDCl3, 126 MHz
Br
CO2Me
1a
179.3
133.8
125.3
77.2 CDCl3
52.2
47.3
45.5
28.0
26.7
26.0
21.4
19.0
f1 (ppm)

^{1}H, $CDCl_3$, 400 MHz

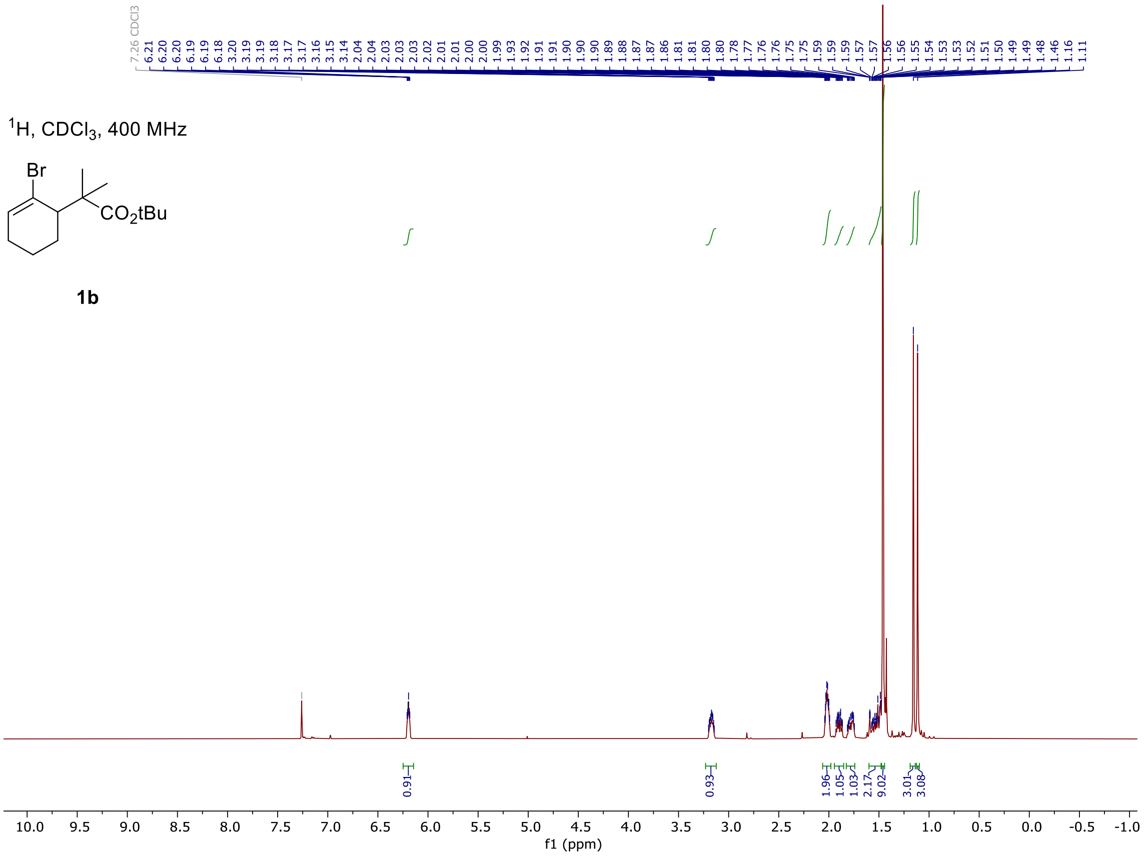

^{13}C, $CDCl_3$, 126 MHz

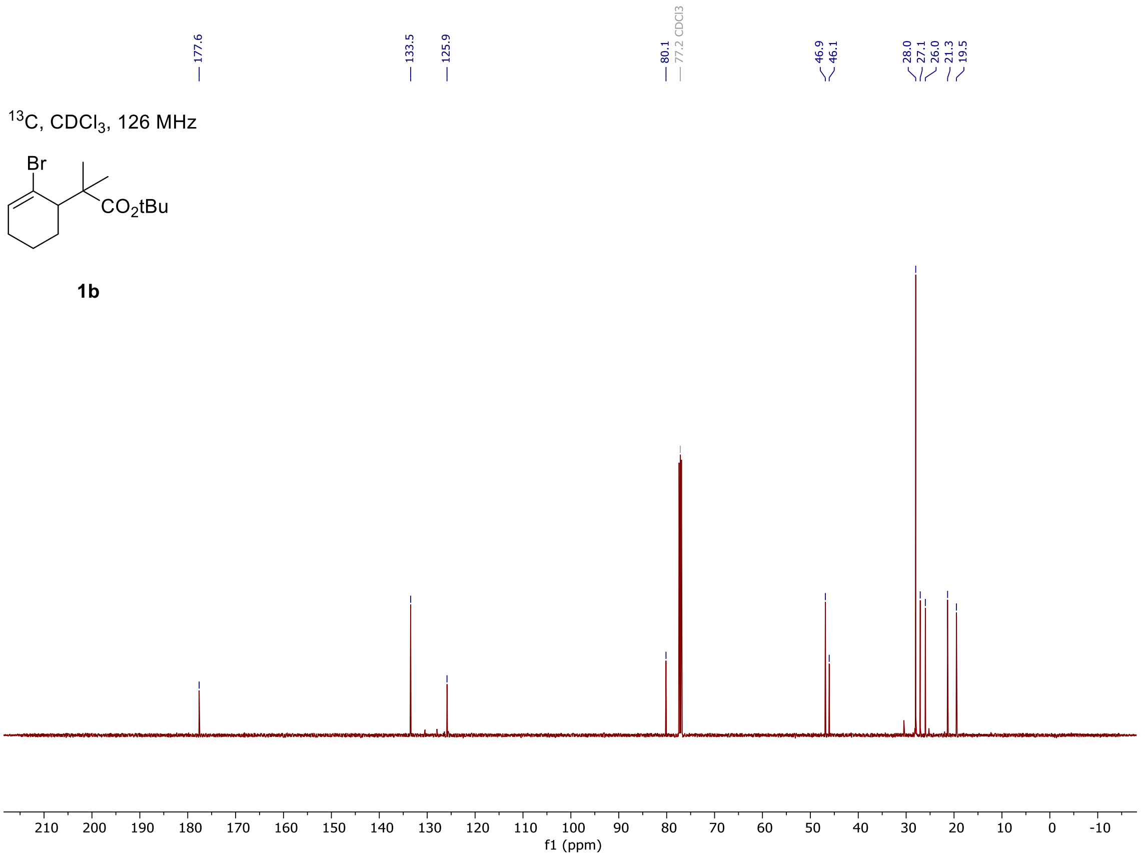

^{1}H, $CDCl_3$, 400 MHz

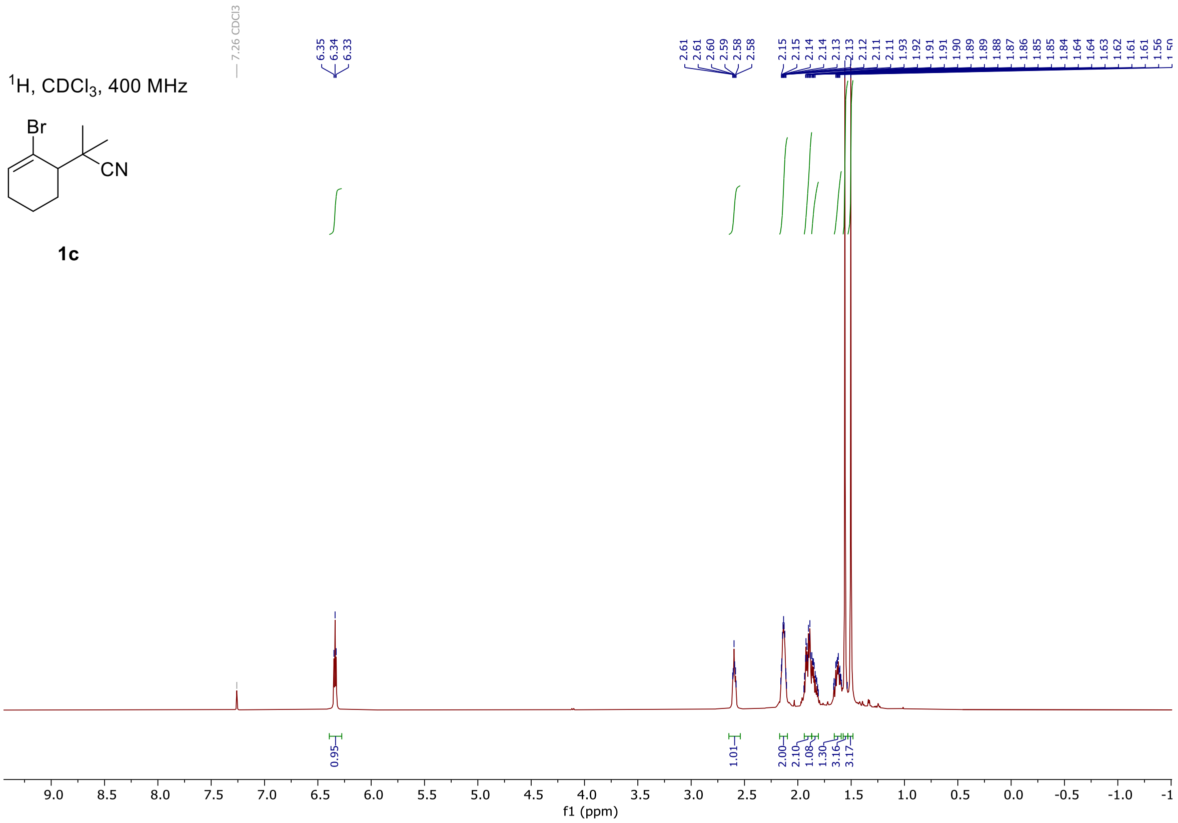

^{13}C, $CDCl_3$, 101 MHz

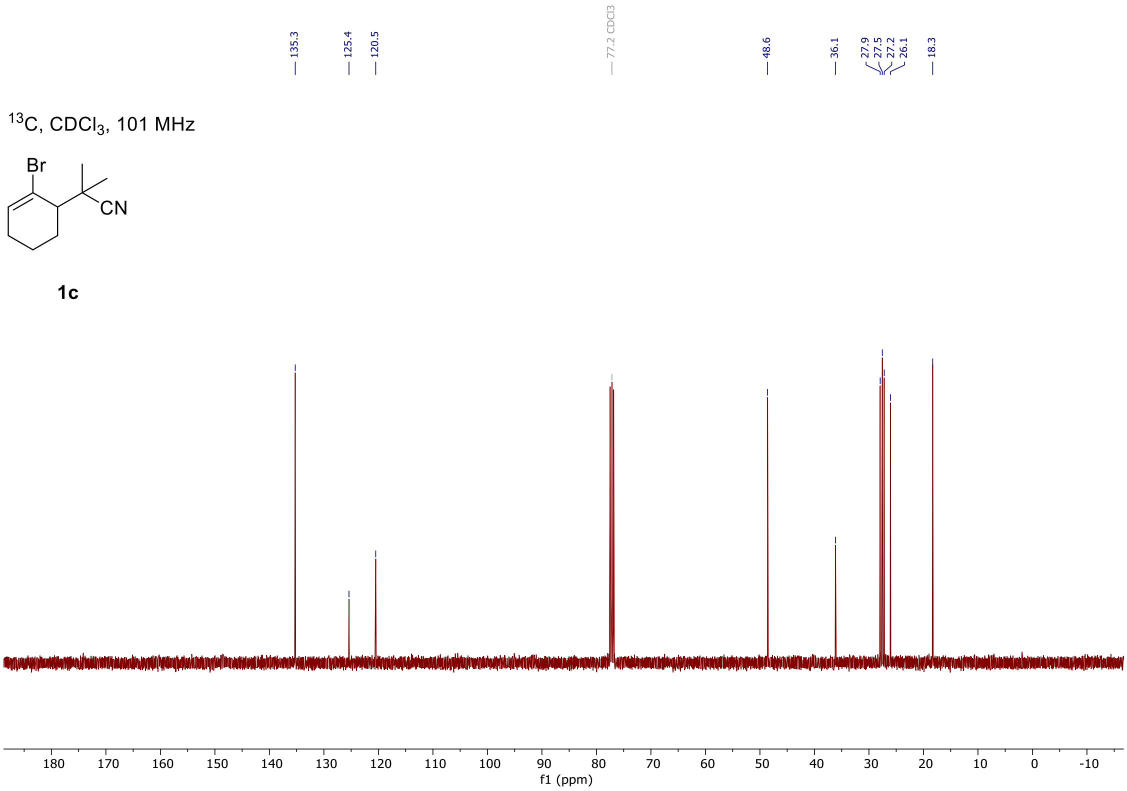

^{1}H, CDCl$_3$, 500 MHz

1d

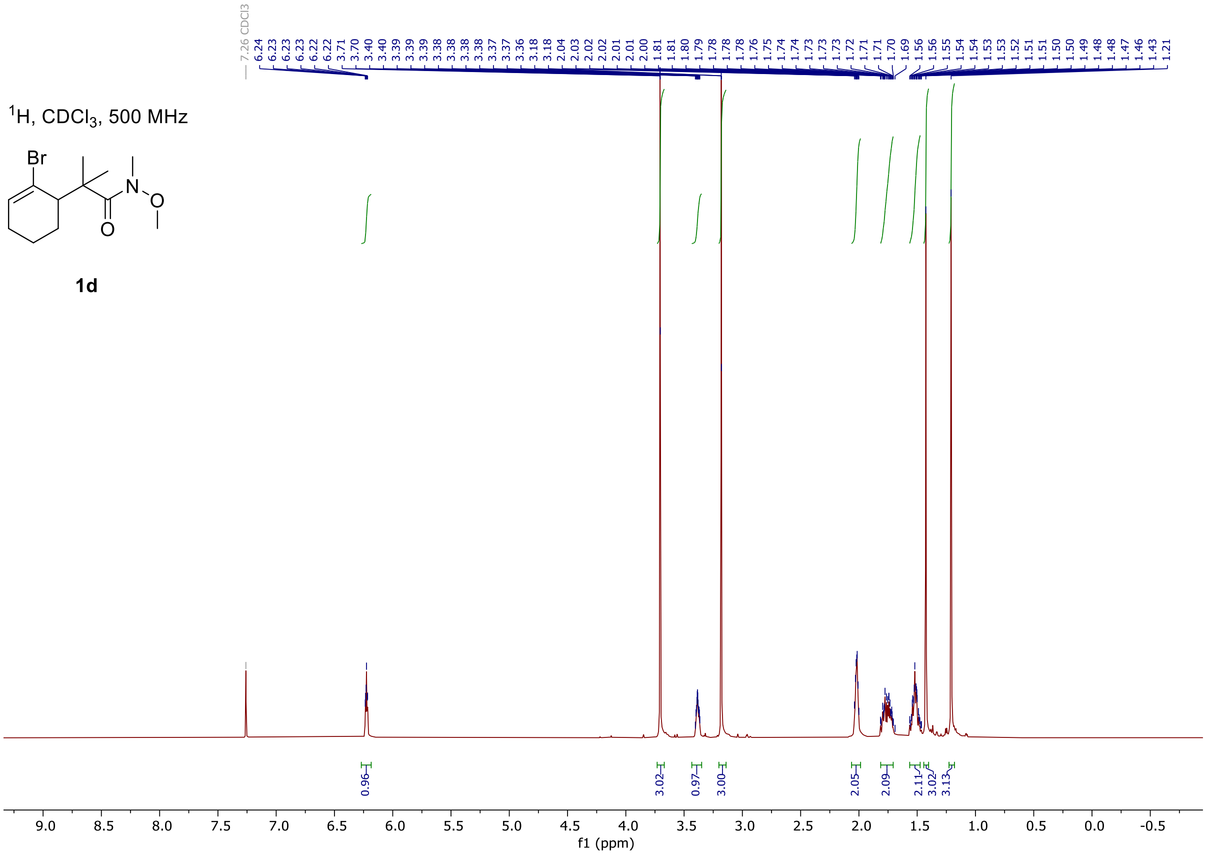

^{13}C CDCl$_3$, 126 MHz

1d

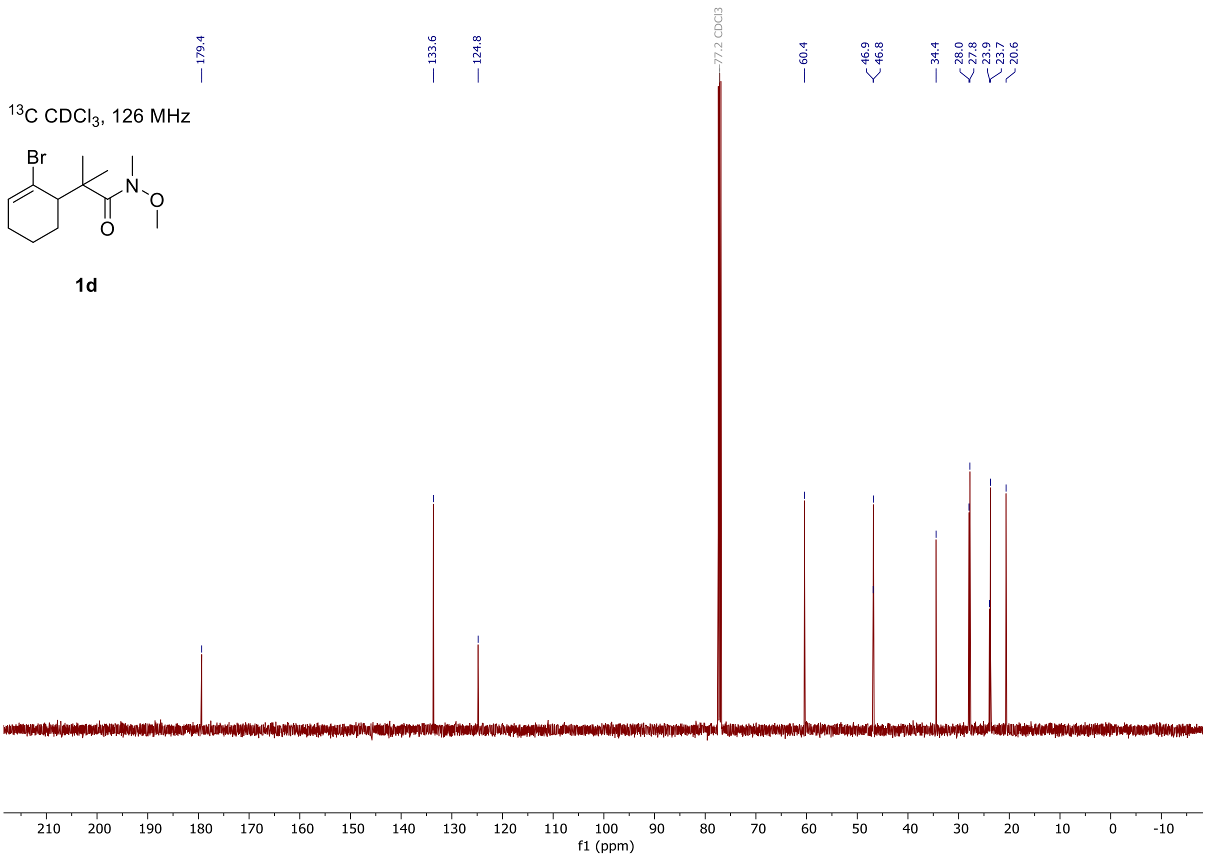

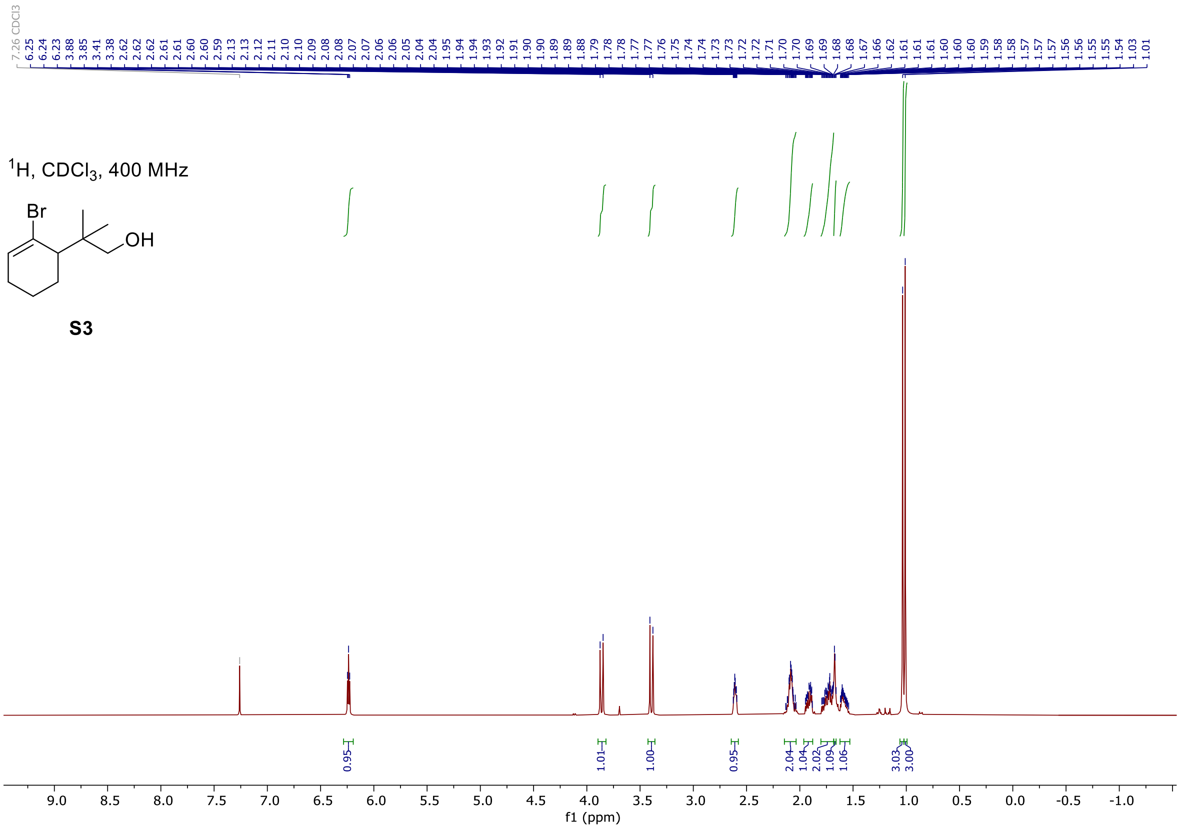

1H, CDCl3, 400 MHz
Br
OH
S3
7.26 CDCl3
6.25 6.24 6.23 3.88 3.85 3.41 3.38 2.62 2.62 2.61 2.61 2.60 2.60 2.59 2.13 2.13 2.12 2.11 2.10 2.10 2.09 2.08 2.08 2.07 2.07 2.06 2.06 2.05 2.04 2.04 1.95 1.94 1.94 1.93 1.92 1.91 1.90 1.90 1.89 1.89 1.88 1.79 1.78 1.78 1.77 1.77 1.76 1.75 1.74 1.74 1.73 1.73 1.72 1.72 1.71 1.70 1.70 1.69 1.69 1.68 1.68 1.67 1.66 1.62 1.61 1.61 1.61 1.60 1.60 1.60 1.59 1.58 1.58 1.57 1.57 1.57 1.56 1.56 1.55 1.55 1.54 1.03 1.01
0.95 1.01 1.00 0.95 2.04 1.04 2.02 1.09 1.06 3.03 3.00
9.0 8.5 8.0 7.5 7.0 6.5 6.0 5.5 5.0 4.5 4.0 3.5 3.0 2.5 2.0 1.5 1.0 0.5 0.0 -0.5 -1.0
f1 (ppm)

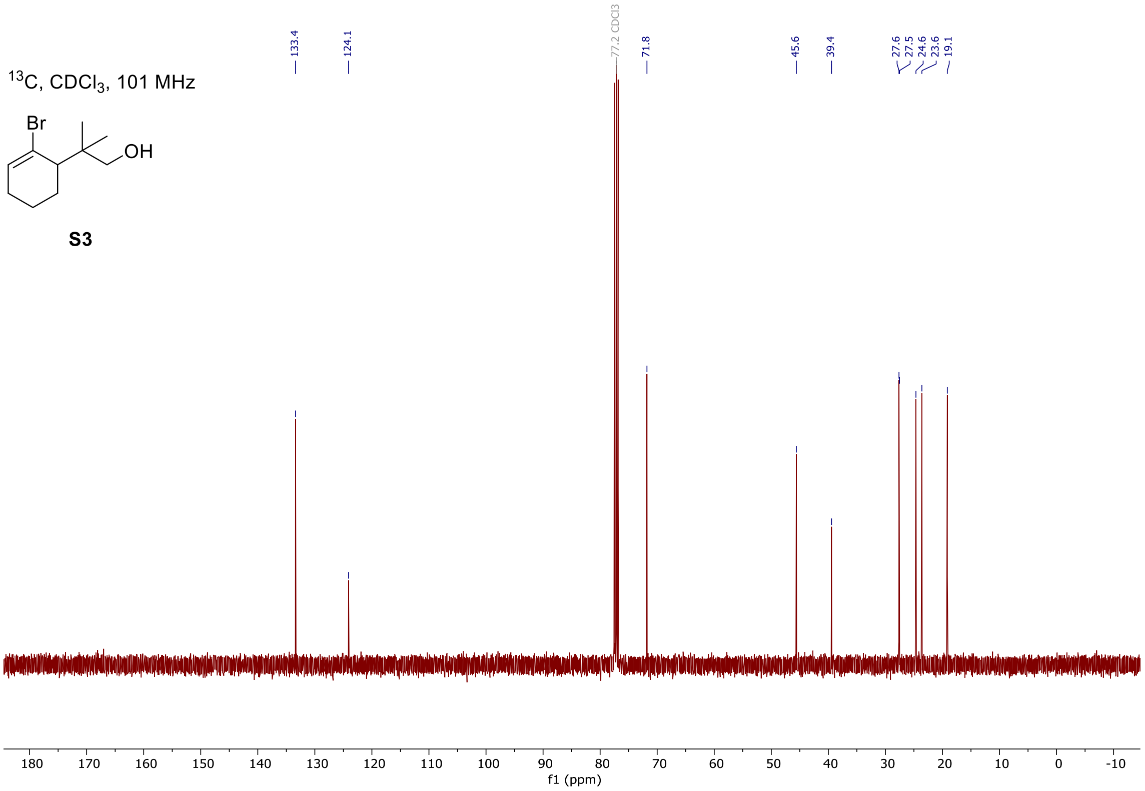

13C, CDCl3, 101 MHz
Br
OH
S3
133.4 124.1 77.2 CDCl3 71.8 45.6 39.4 27.6 27.5 24.6 23.6 19.1
180 170 160 150 140 130 120 110 100 90 80 70 60 50 40 30 20 10 0 -10
f1 (ppm)

^{1}H, $CDCl_3$, 250 MHz

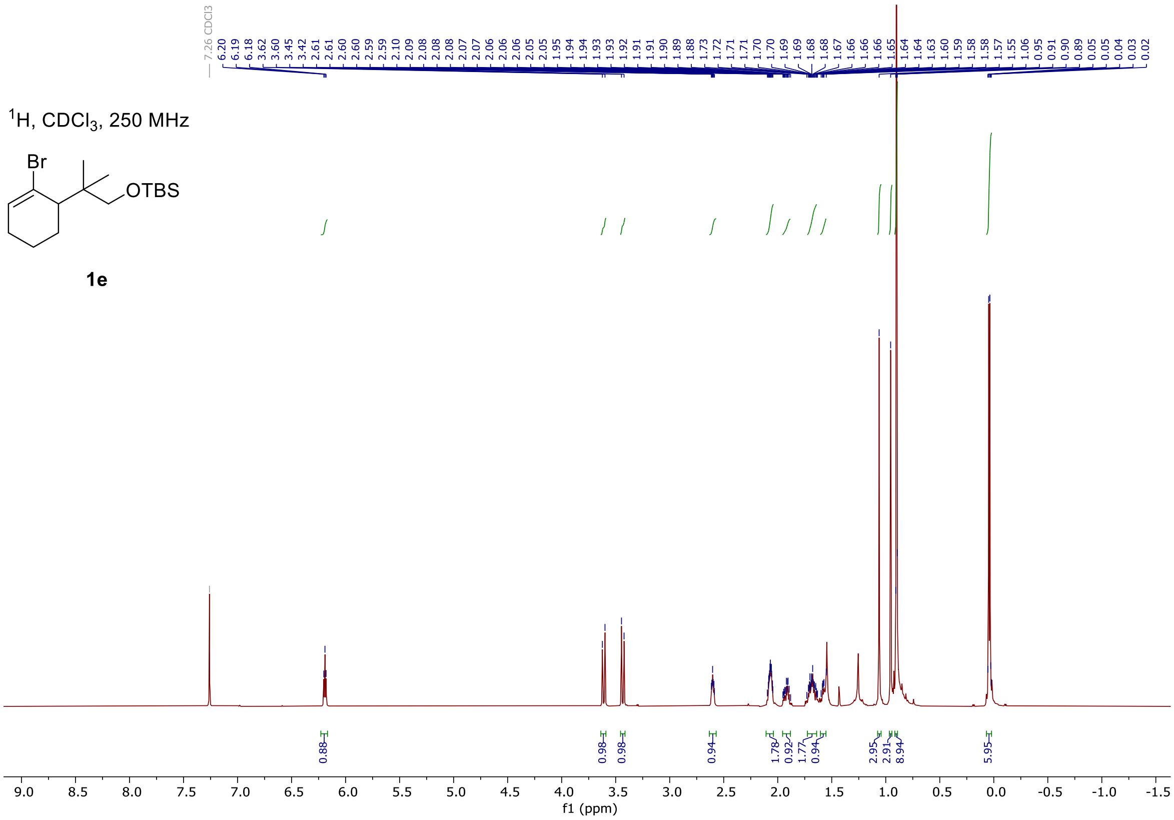

1e

^{13}C, $CDCl_3$, 126 MHz

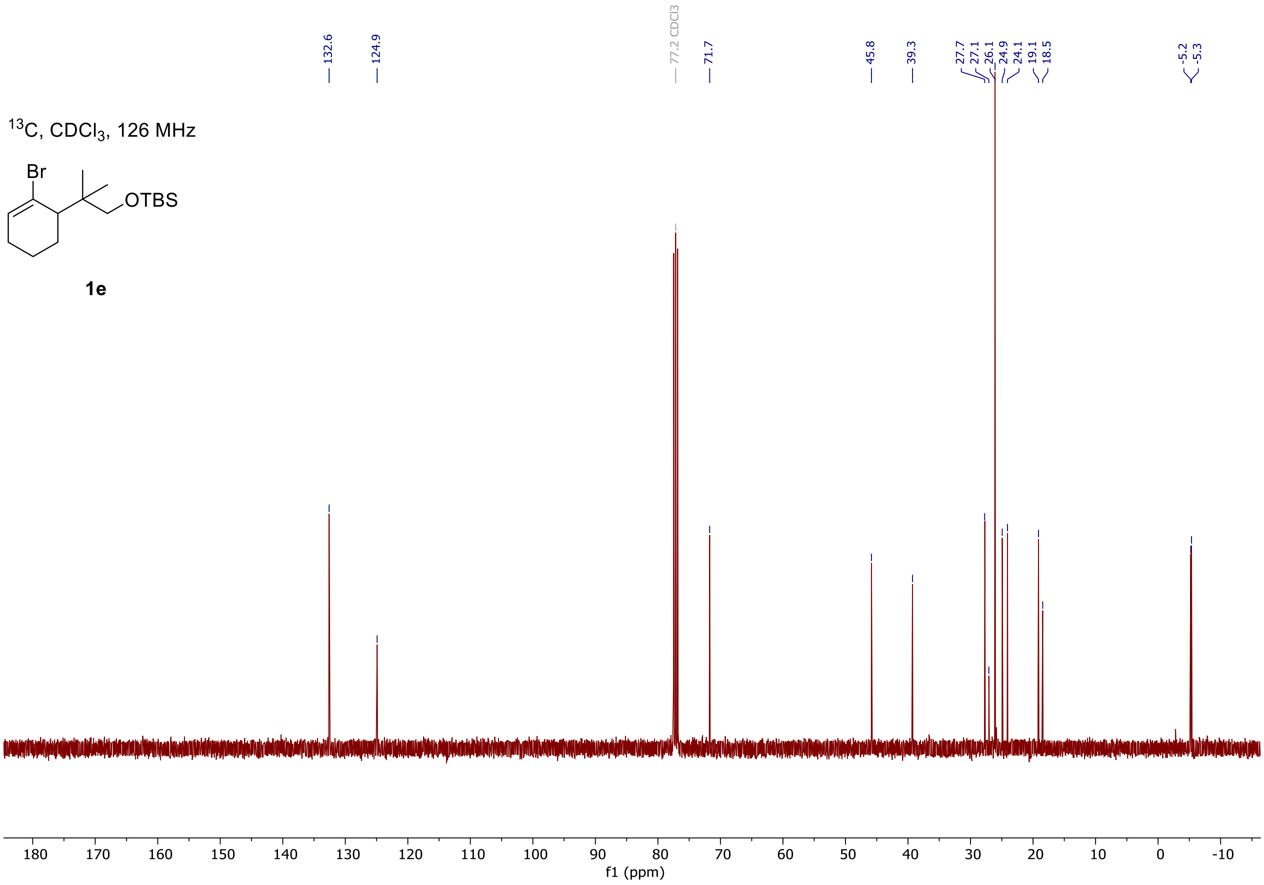

1e

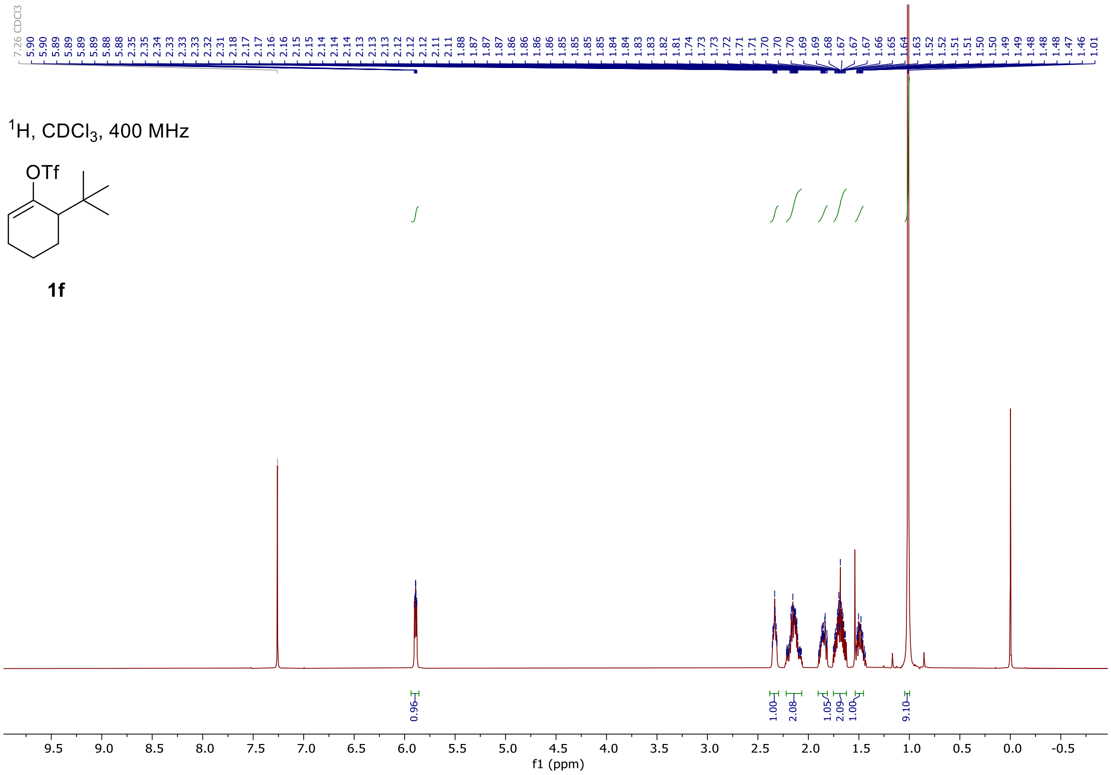

^{1}H, CDCl$_3$, 400 MHz
OTf
1f
7.26 CDCl3
5.90 5.90 5.89 5.89 5.89 5.89 5.88 5.88 2.35 2.35 2.34 2.33 2.33 2.32 2.31 2.18 2.17 2.17 2.16 2.16 2.15 2.15 2.14 2.14 2.14 2.13 2.13 2.13 2.12 2.12 2.12 2.11 2.11 1.88 1.87 1.87 1.87 1.86 1.86 1.86 1.85 1.85 1.85 1.85 1.84 1.84 1.83 1.83 1.82 1.81 1.74 1.73 1.73 1.72 1.71 1.71 1.70 1.70 1.69 1.69 1.68 1.67 1.67 1.67 1.66 1.65 1.64 1.63 1.52 1.52 1.51 1.51 1.50 1.50 1.49 1.49 1.48 1.48 1.47 1.46 1.01
0.96
1.00
2.08
1.05
2.09
1.00
9.10
9.5 9.0 8.5 8.0 7.5 7.0 6.5 6.0 5.5 5.0 4.5 4.0 3.5 3.0 2.5 2.0 1.5 1.0 0.5 0.0 -0.5
f1 (ppm)

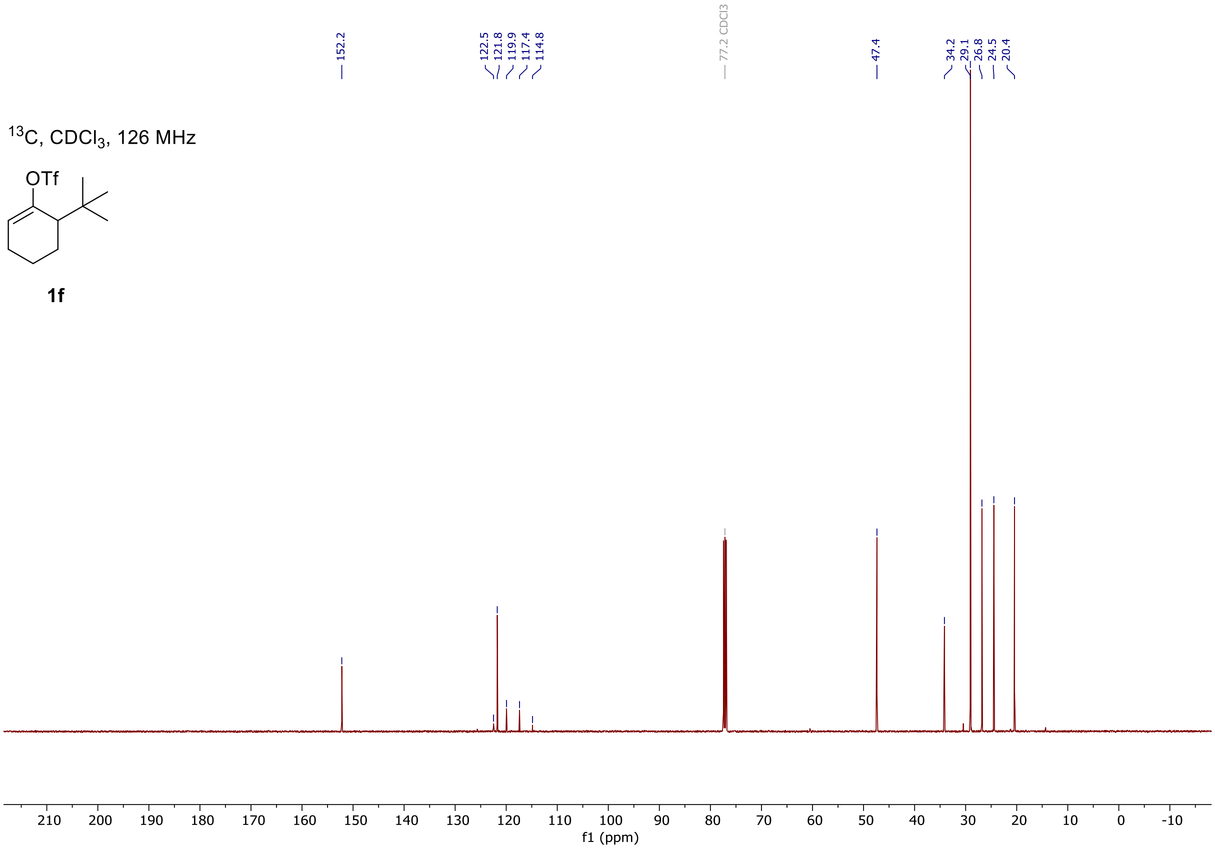

^{13}C, CDCl$_3$, 126 MHz
OTf
1f
152.2
122.5
121.8
119.9
117.4
114.8
77.2 CDCl3
47.4
34.2
29.1
26.8
24.5
20.4
210 200 190 180 170 160 150 140 130 120 110 100 90 80 70 60 50 40 30 20 10 0 -10
f1 (ppm)

$^{19}F\{^{1}H\}$, $CDCl_3$, 376 MHz

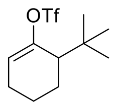

1f

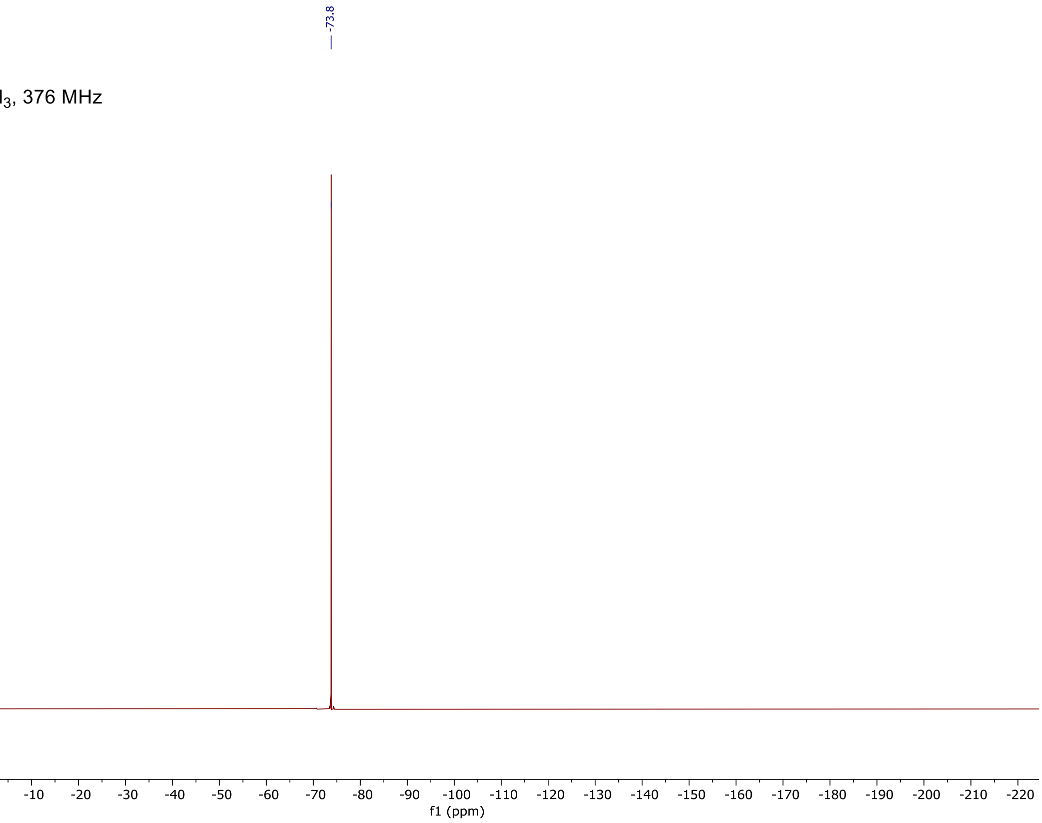

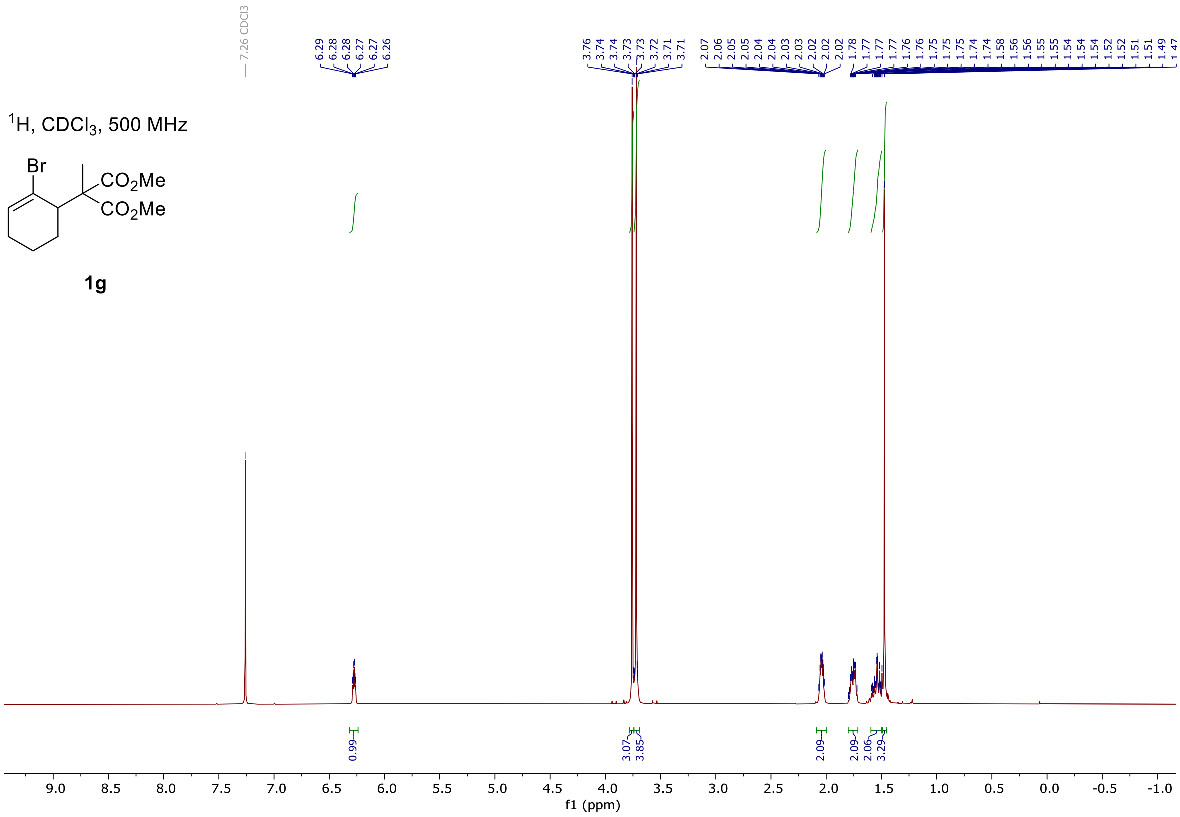
^{1}H, CDCl$_3$, 500 MHz
Br
CO$_2$Me
CO$_2$Me
1g
f1 (ppm)

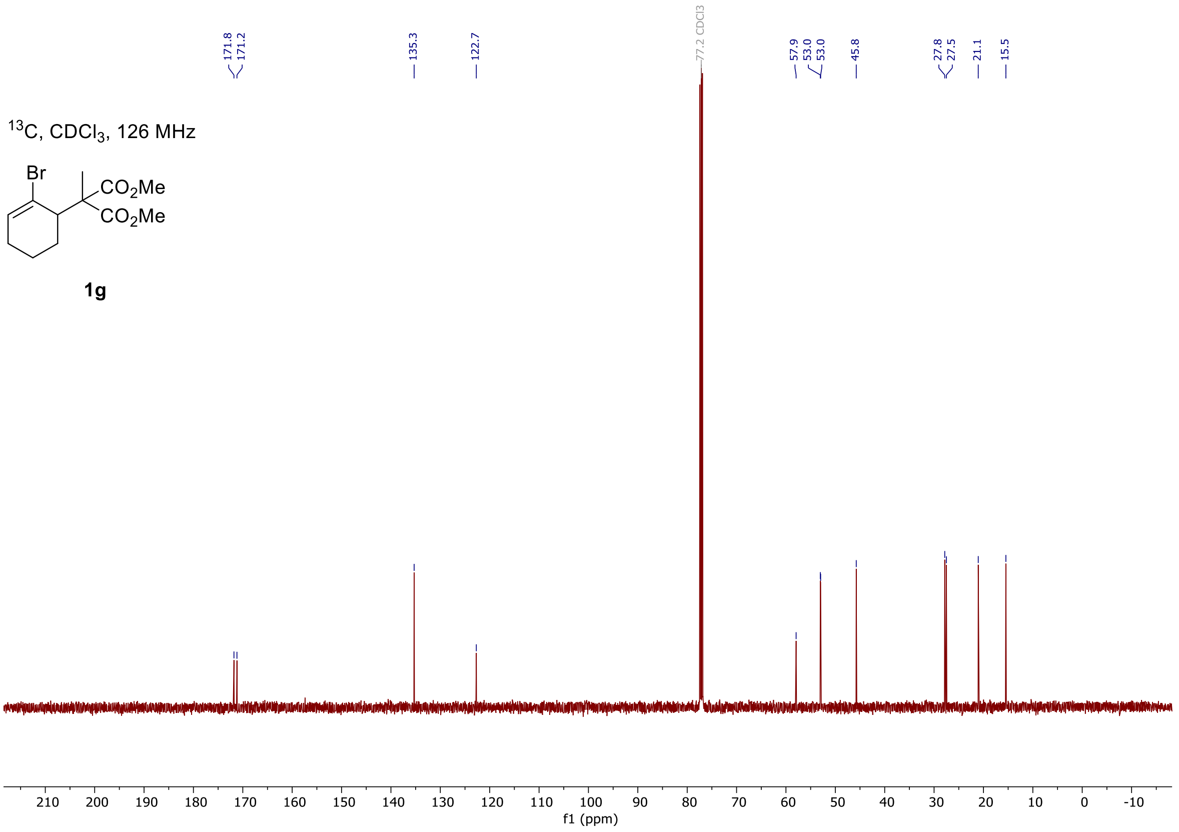
^{13}C, CDCl$_3$, 126 MHz
Br
CO$_2$Me
CO$_2$Me
1g
f1 (ppm)

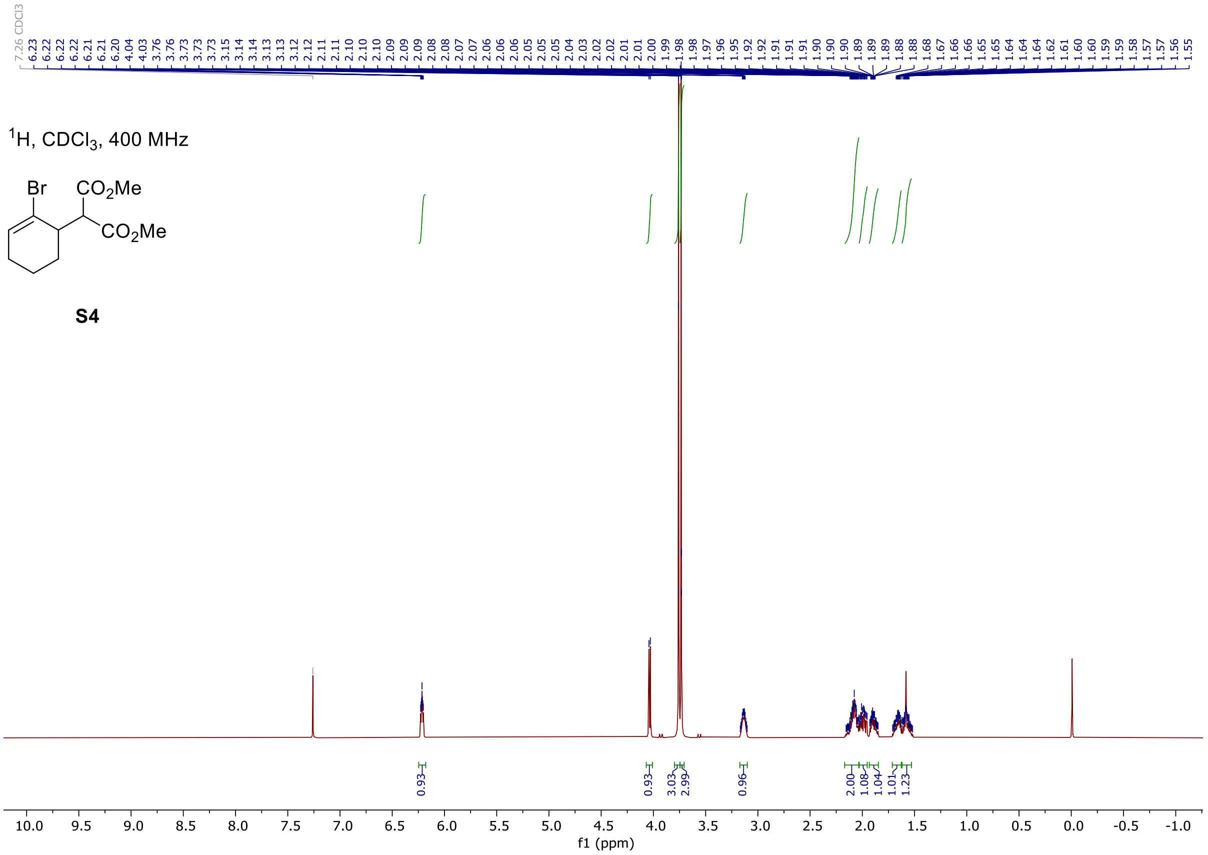

¹H, CDCl₃, 400 MHz
Br
CO₂Me
CO₂Me
S4
f1 (ppm)

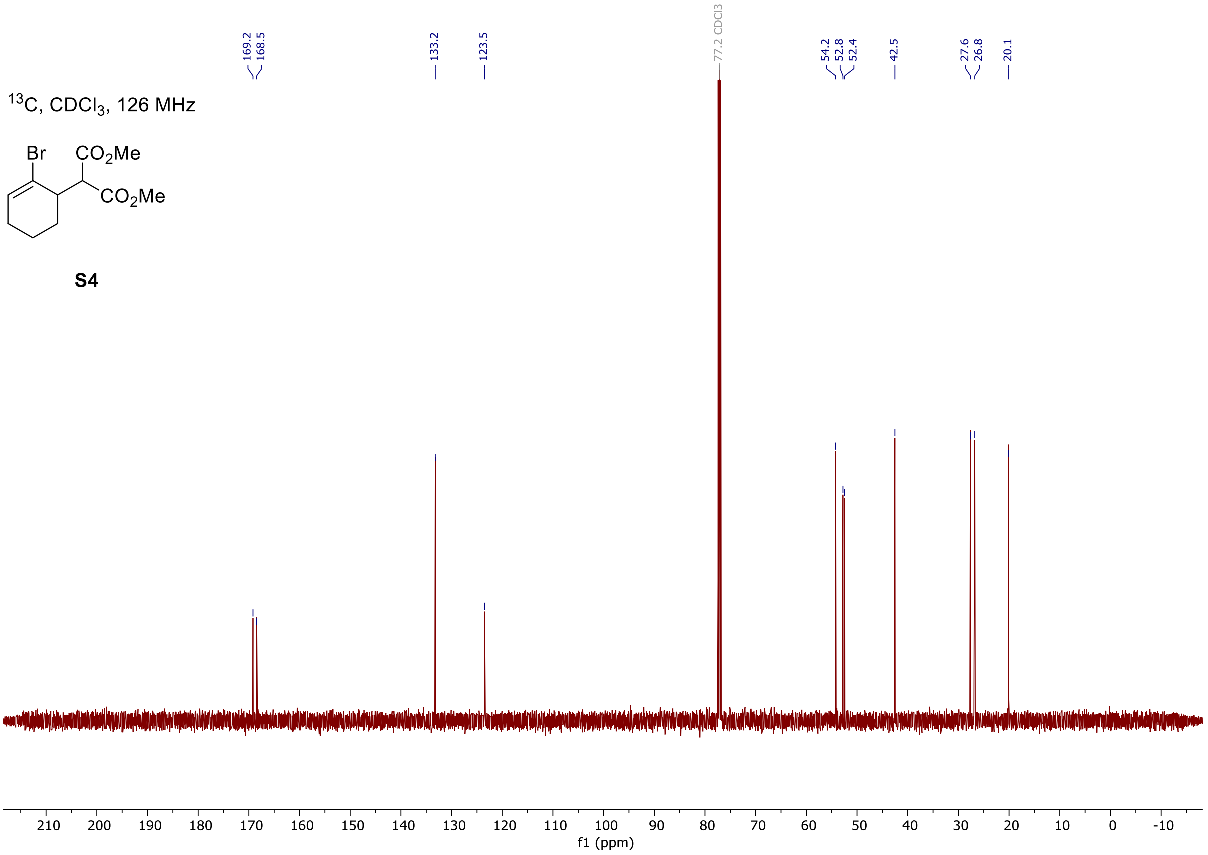

¹³C, CDCl₃, 126 MHz
Br
CO₂Me
CO₂Me
S4
169.2
168.5
133.2
123.5
77.2 CDCl3
54.2
52.8
52.4
42.5
27.6
26.8
20.1
f1 (ppm)

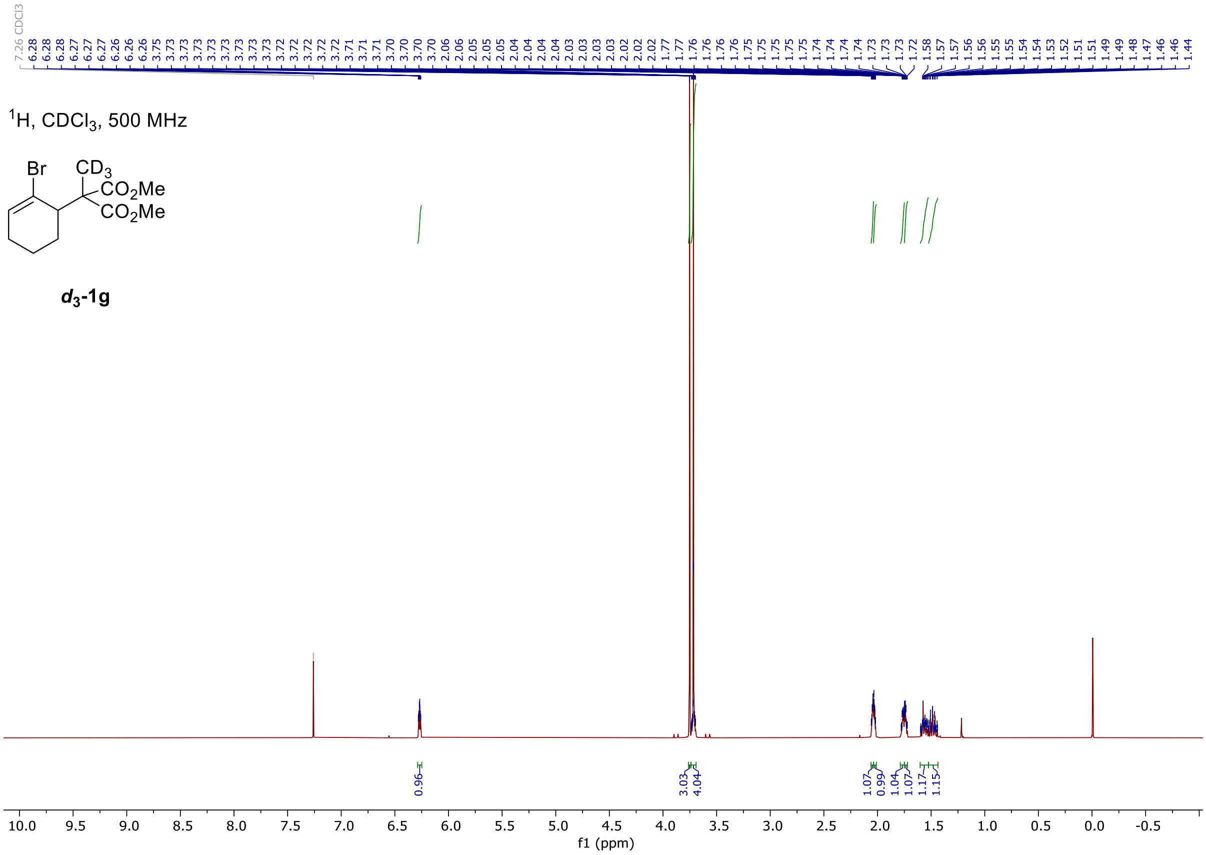

¹H, CDCl₃, 500 MHz
Br
CD₃
CO₂Me
CO₂Me
d₃-1g
0.96
3.03
4.04
1.07
0.99
1.04
1.07
1.17
1.15
f1 (ppm)

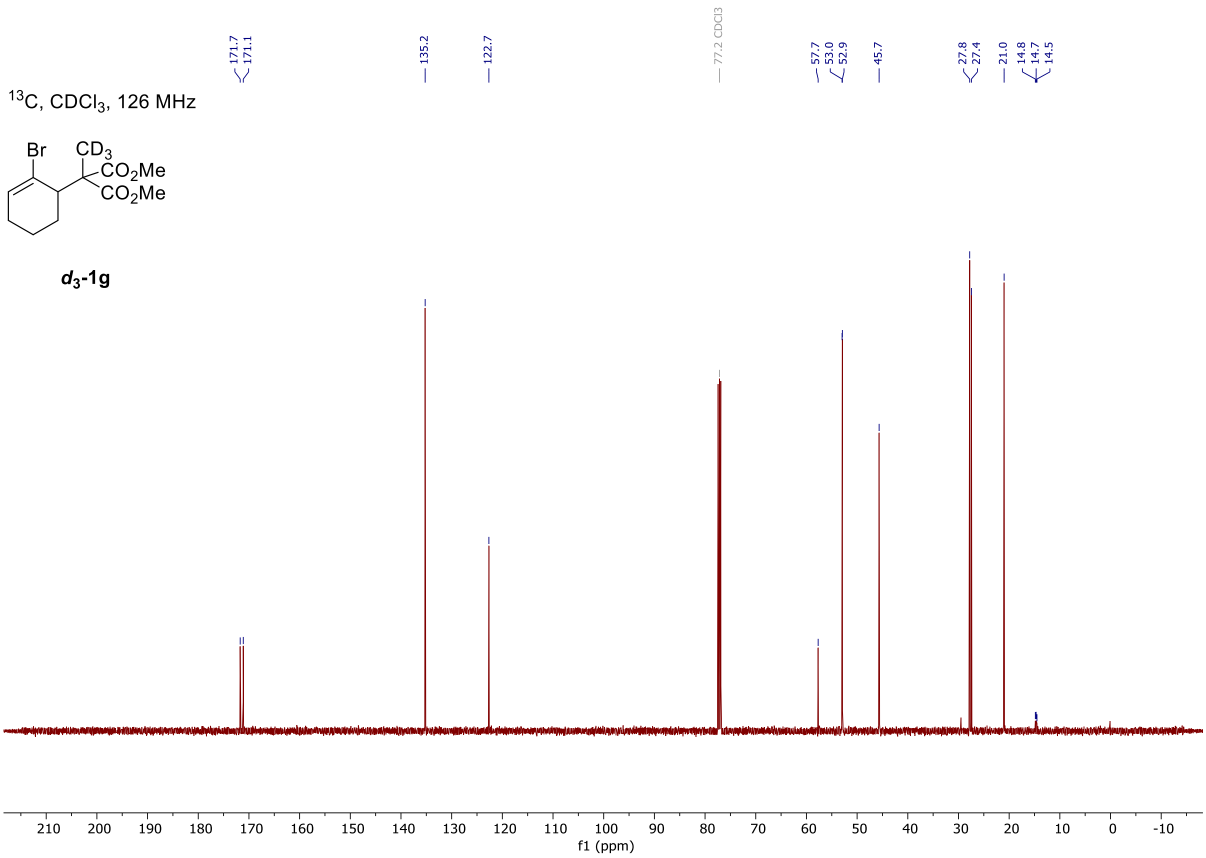

¹³C, CDCl₃, 126 MHz
171.7
171.1
135.2
122.7
77.2 CDCl3
57.7
53.0
52.9
45.7
27.8
27.4
21.0
14.8
14.7
14.5
Br
CD₃
CO₂Me
CO₂Me
d₃-1g
f1 (ppm)

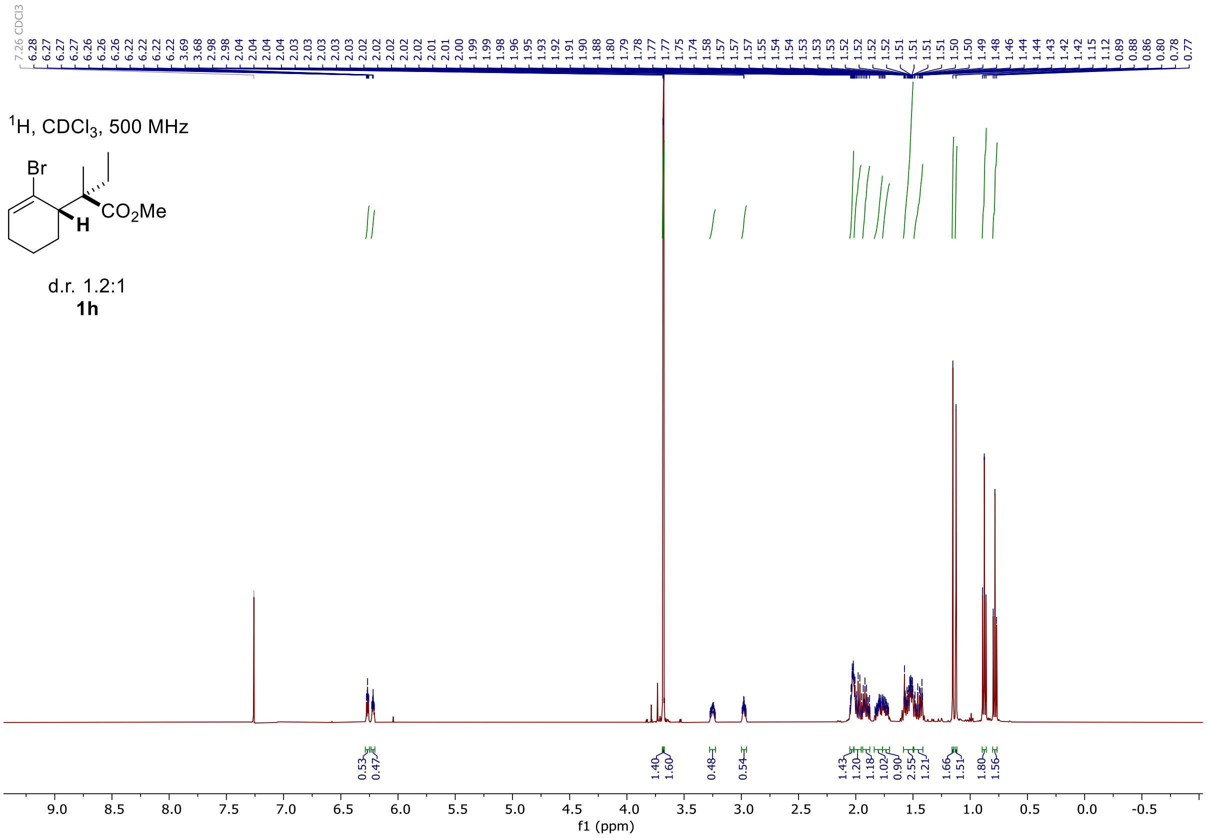

^{1}H, CDCl$_3$, 500 MHz
Br
CO$_2$Me
H
d.r. 1.2:1
1h
f1 (ppm)

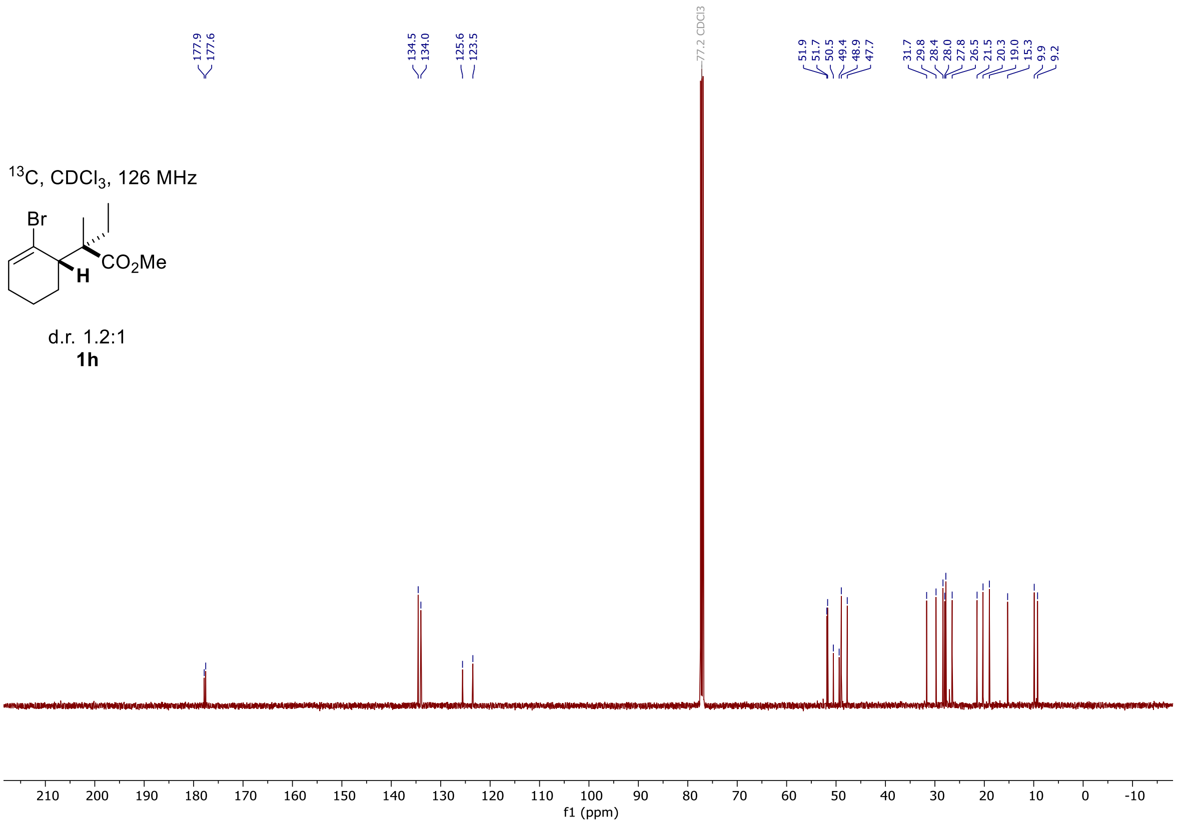

^{13}C, CDCl$_3$, 126 MHz
Br
CO$_2$Me
H
d.r. 1.2:1
1h
f1 (ppm)

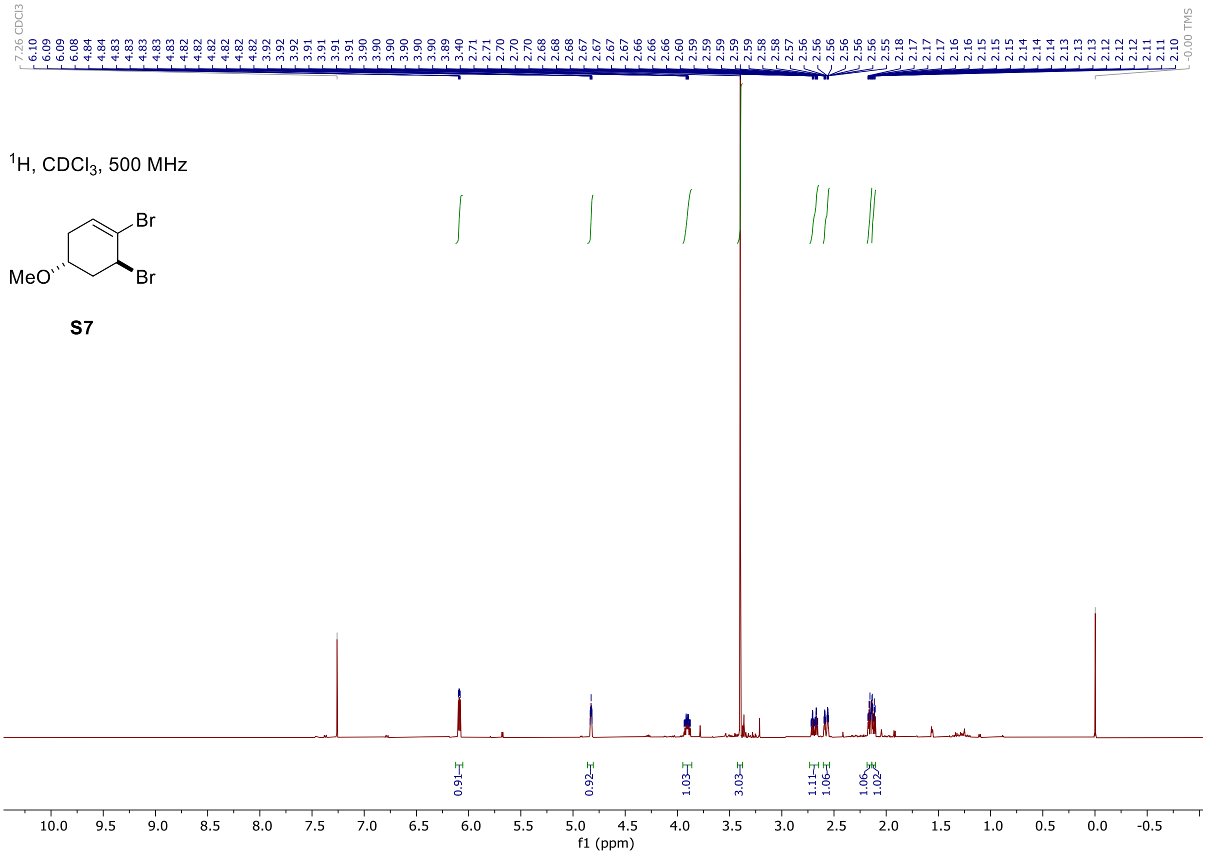

^{1}H, CDCl$_3$, 500 MHz
MeO
Br
Br
S7
f1 (ppm)

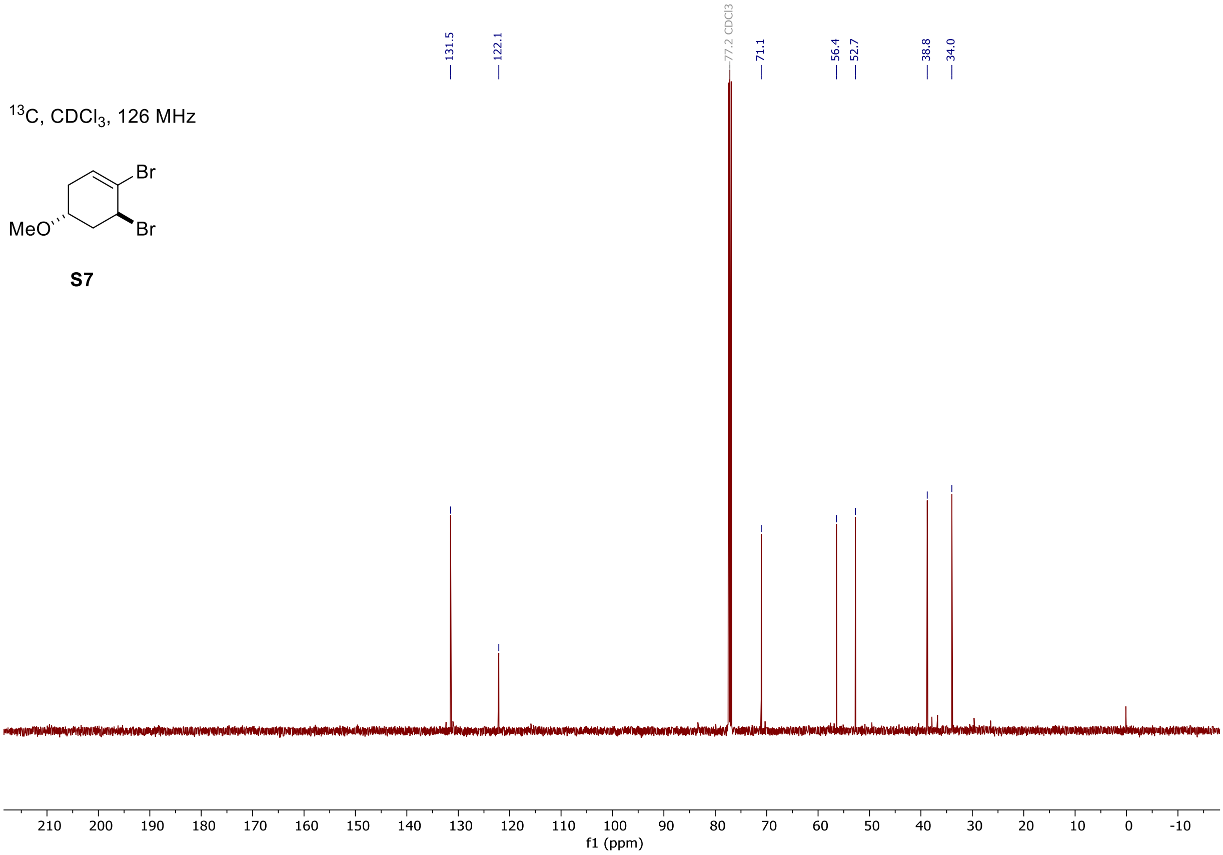

131.5
122.1
77.2 CDCl3
71.1
56.4
52.7
38.8
34.0
^{13}C, CDCl$_3$, 126 MHz
MeO
Br
Br
S7
f1 (ppm)

NOESY of **S7**.

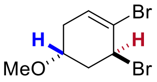

S7

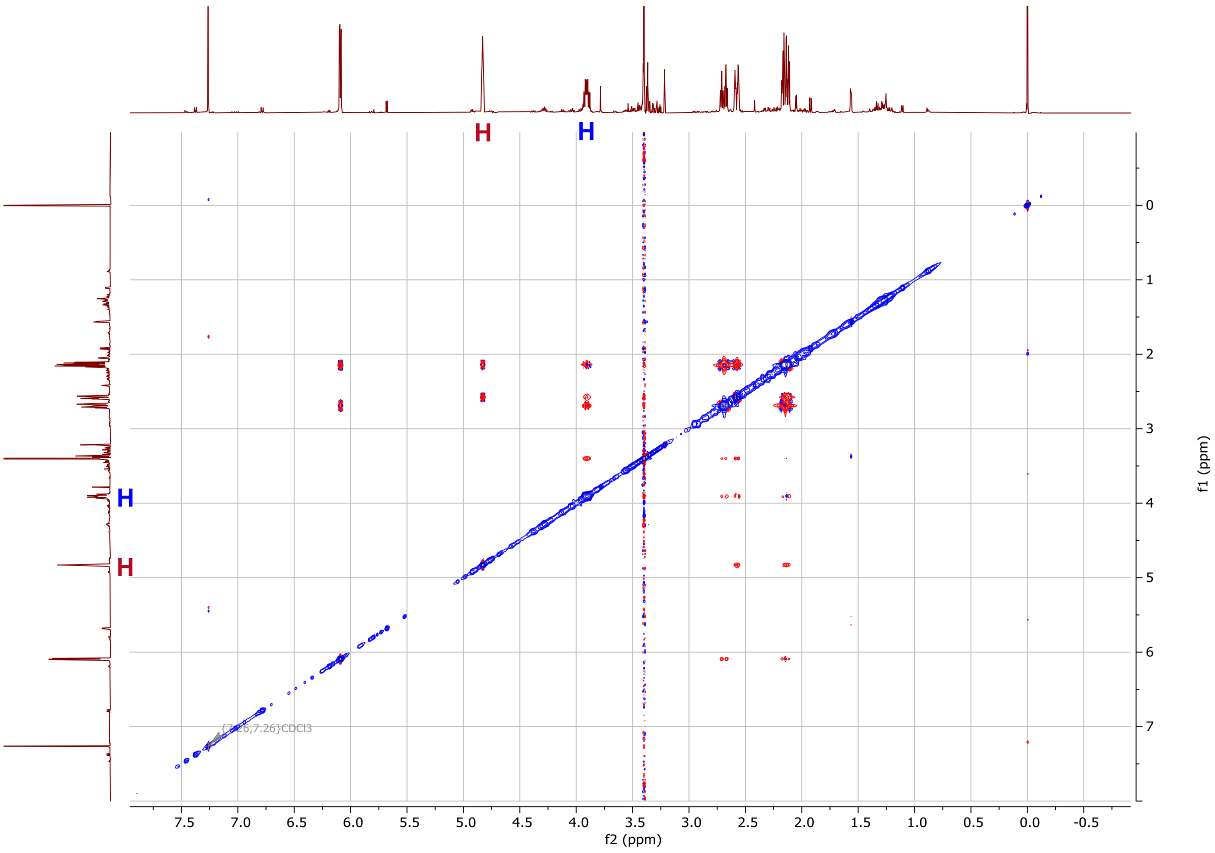

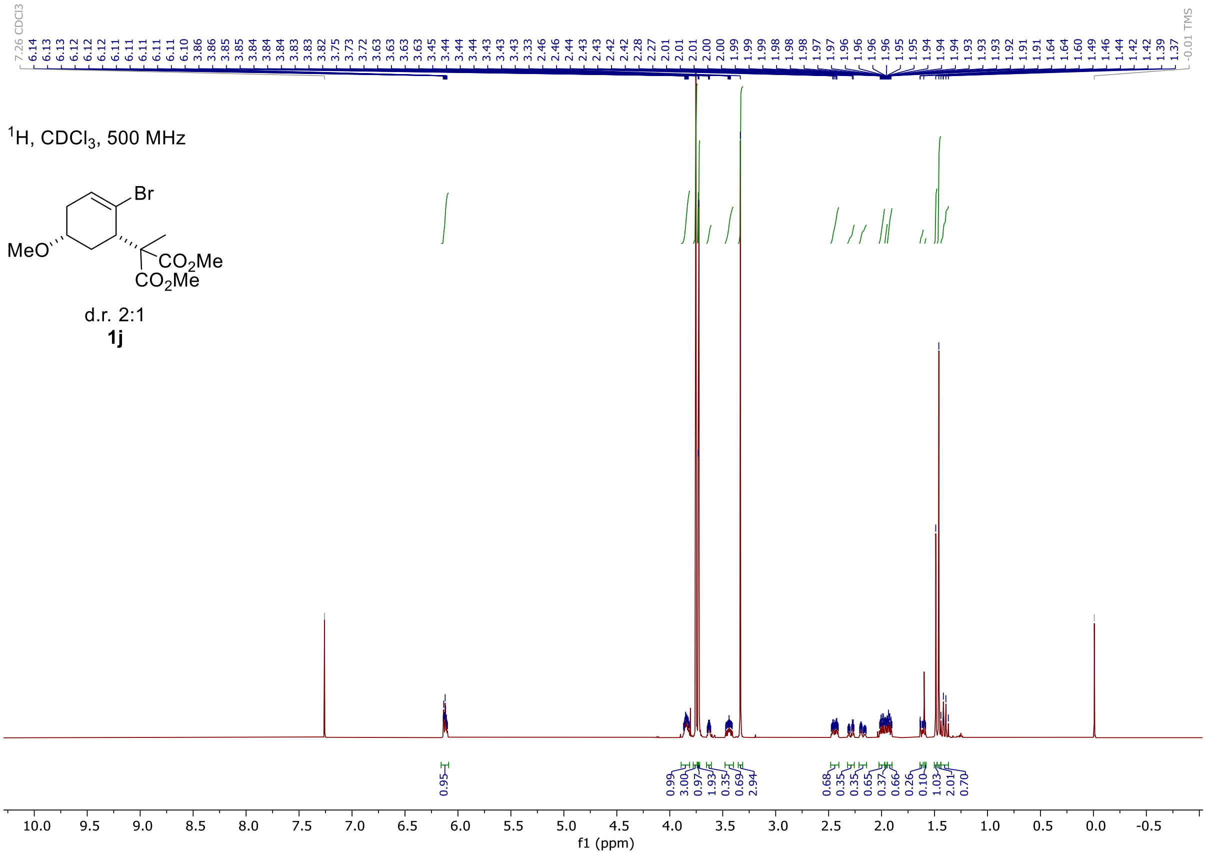
7.26 CDCl3
6.14 6.13 6.13 6.12 6.12 6.12 6.11 6.11 6.11 6.11 6.11 6.10 3.86 3.86 3.85 3.85 3.84 3.84 3.84 3.83 3.83 3.82 3.75 3.73 3.72 3.63 3.63 3.63 3.63 3.45 3.44 3.44 3.44 3.43 3.43 3.43 3.33 2.46 2.44 2.43 2.43 2.42 2.42 2.28 2.27 2.01 2.01 2.00 1.99 1.99 1.99 1.98 1.98 1.98 1.97 1.96 1.96 1.96 1.96 1.95 1.95 1.94 1.94 1.94 1.93 1.93 1.93 1.92 1.91 1.91 1.64 1.64 1.60 1.49 1.46 1.44 1.42 1.39 1.37
-0.01 TMS
^{1}H, CDCl$_3$, 500 MHz
Br
MeO
CO$_2$Me
CO$_2$Me
d.r. 2:1
1j
0.95
0.99 3.00 0.97 1.93 0.35 0.69 2.94
0.68 0.35 0.35 0.65 0.37 0.66 0.26 0.10 1.03 2.01 0.70
10.0 9.5 9.0 8.5 8.0 7.5 7.0 6.5 6.0 5.5 5.0 4.5 4.0 3.5 3.0 2.5 2.0 1.5 1.0 0.5 0.0 -0.5
f1 (ppm)

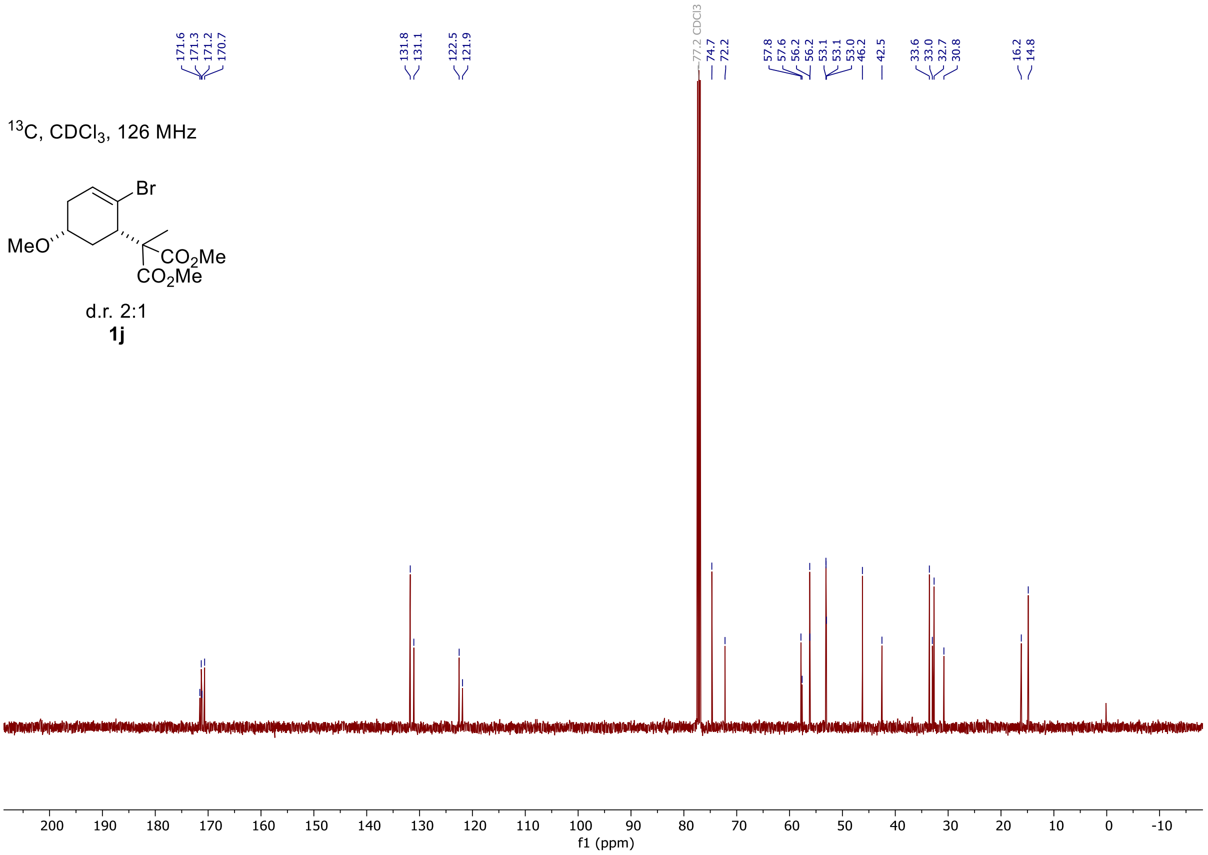
171.6 171.3 171.2 170.7
131.8 131.1
122.5 121.9
77.2 CDCl3
74.7 72.2
57.8 57.6 56.2 53.1 53.0 46.2 42.5
33.6 33.0 32.7 30.8
16.2 14.8
^{13}C, CDCl$_3$, 126 MHz
Br
MeO
CO$_2$Me
CO$_2$Me
d.r. 2:1
1j
200 190 180 170 160 150 140 130 120 110 100 90 80 70 60 50 40 30 20 10 0 -10
f1 (ppm)

NOESY of **1j**.

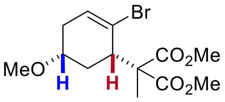

1j

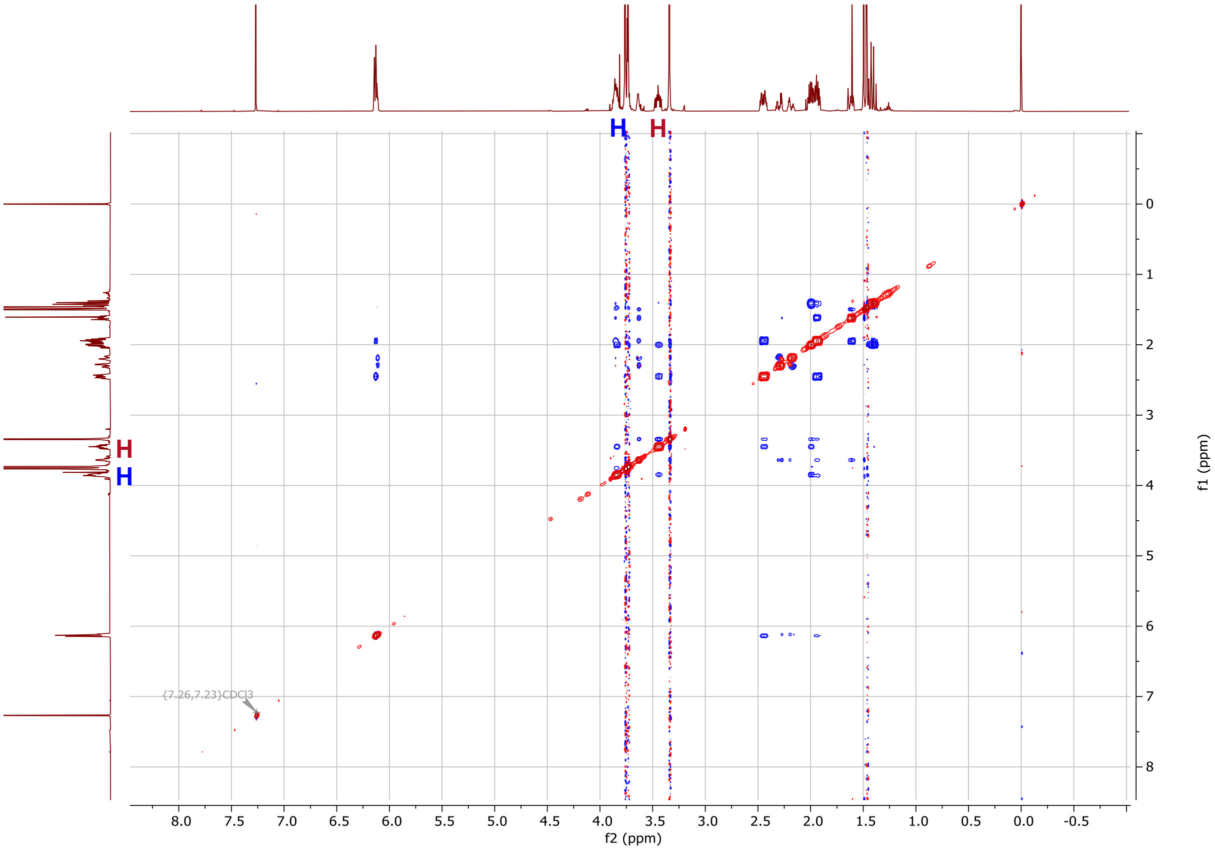

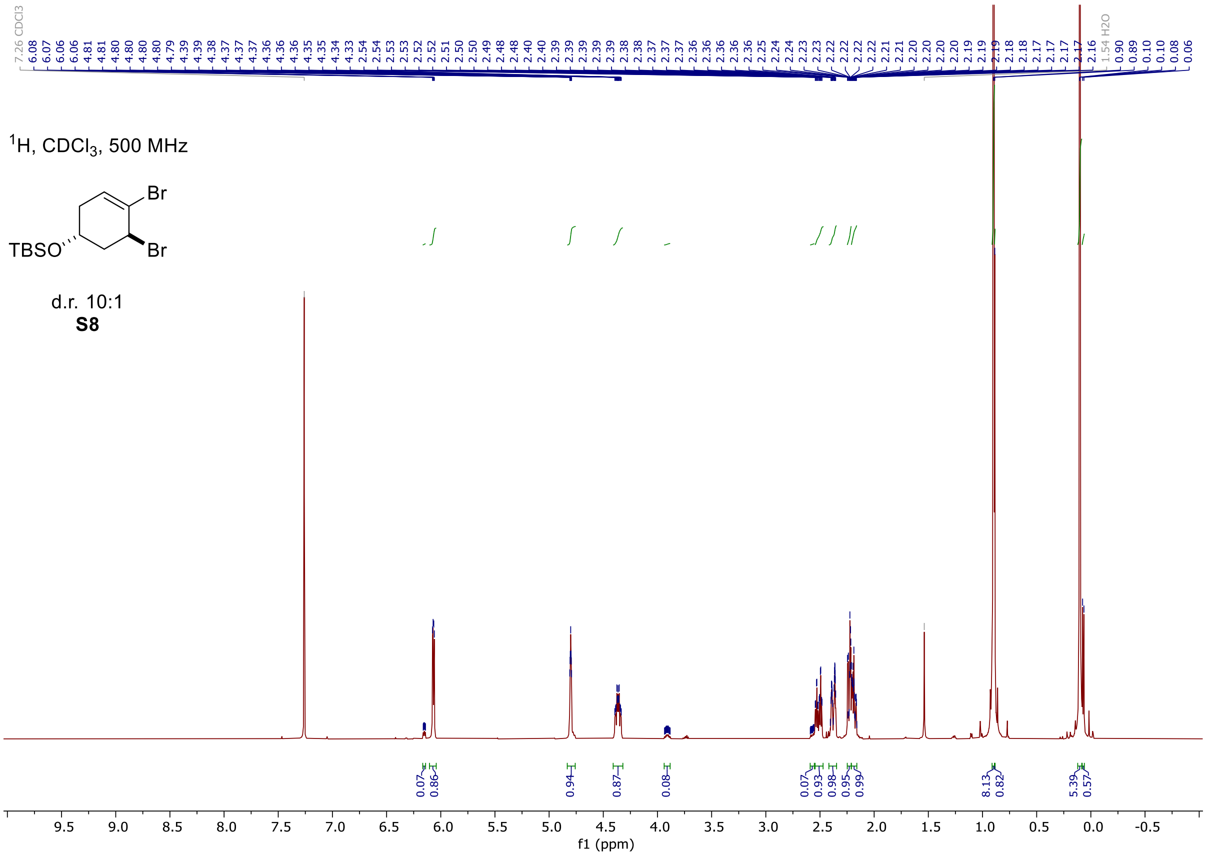
7.26 CDCl3
6.08
6.07
6.06
6.06
4.81
4.81
4.80
4.80
4.80
4.80
4.79
4.39
4.39
4.38
4.37
4.37
4.37
4.36
4.36
4.36
4.35
4.35
4.34
4.33
2.54
2.54
2.53
2.53
2.52
2.52
2.51
2.50
2.50
2.49
2.48
2.48
2.40
2.40
2.39
2.39
2.39
2.39
2.39
2.38
2.38
2.37
2.37
2.37
2.36
2.36
2.36
2.36
2.36
2.25
2.24
2.24
2.23
2.23
2.22
2.22
2.22
2.22
2.21
2.21
2.20
2.20
2.20
2.20
2.19
2.19
2.19
2.18
2.18
2.17
2.17
2.17
2.16
1.54 H2O
0.90
0.89
0.10
0.08
0.06
1H, CDCl3, 500 MHz
Br
TBSO
Br
d.r. 10:1
S8
0.07
0.86
0.94
0.87
0.08
0.07
0.93
0.98
0.95
0.99
8.13
0.82
5.39
0.57
9.5 9.0 8.5 8.0 7.5 7.0 6.5 6.0 5.5 5.0 4.5 4.0 3.5 3.0 2.5 2.0 1.5 1.0 0.5 0.0 -0.5
f1 (ppm)

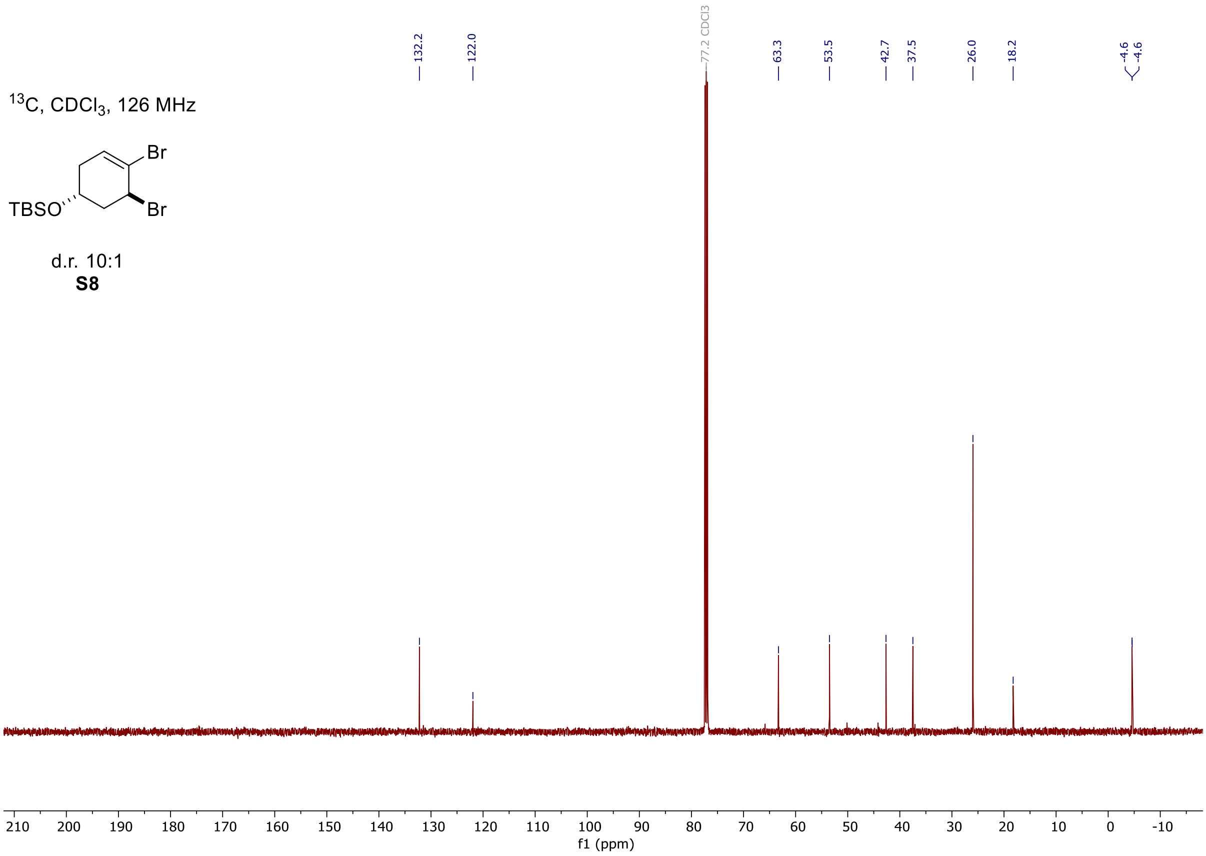
132.2
122.0
77.2 CDCl3
63.3
53.5
42.7
37.5
26.0
18.2
-4.6
-4.6
13C, CDCl3, 126 MHz
Br
TBSO
Br
d.r. 10:1
S8
210 200 190 180 170 160 150 140 130 120 110 100 90 80 70 60 50 40 30 20 10 0 -10
f1 (ppm)

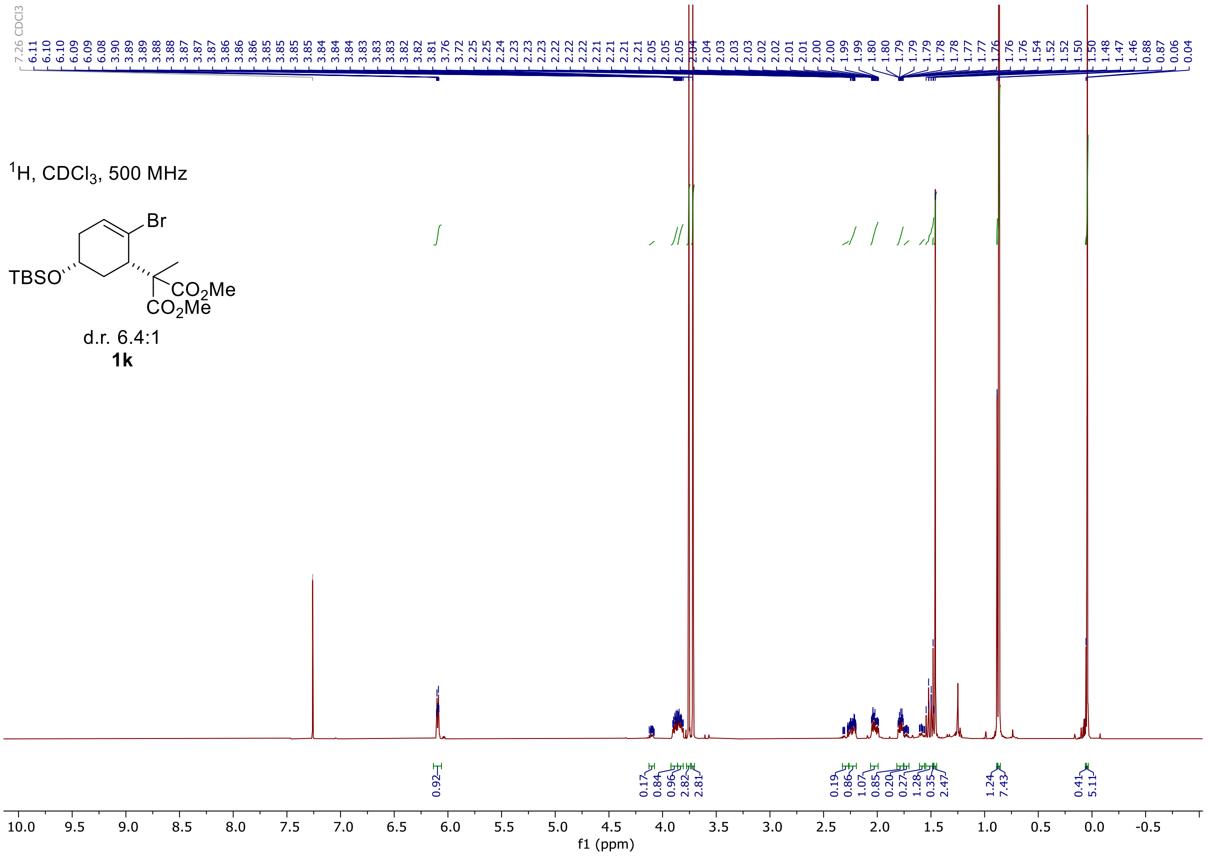

^{1}H, CDCl$_3$, 500 MHz
Br
TBSO
CO$_2$Me
CO$_2$Me
d.r. 6.4:1
1k
f1 (ppm)

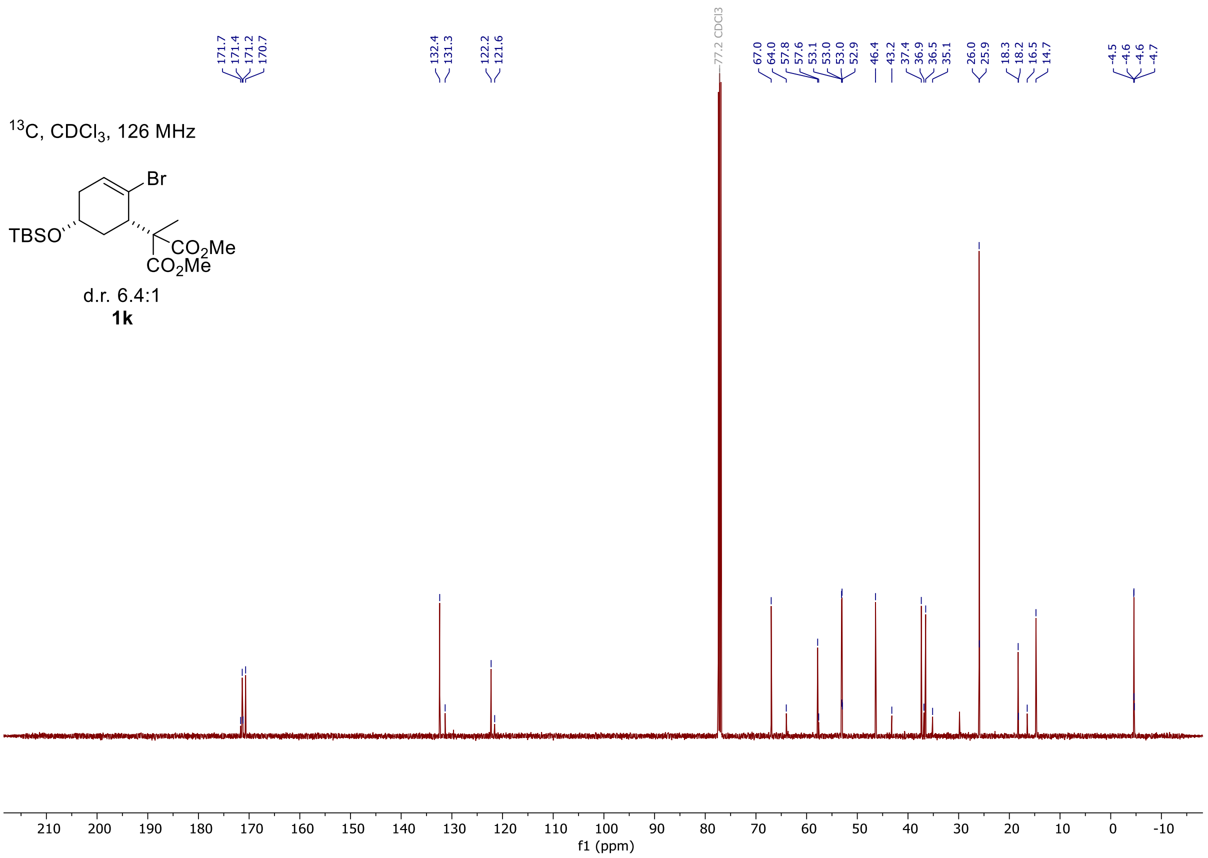

^{13}C, CDCl$_3$, 126 MHz
Br
TBSO
CO$_2$Me
CO$_2$Me
d.r. 6.4:1
1k
f1 (ppm)

NOESY of **1k**.

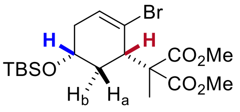

1k

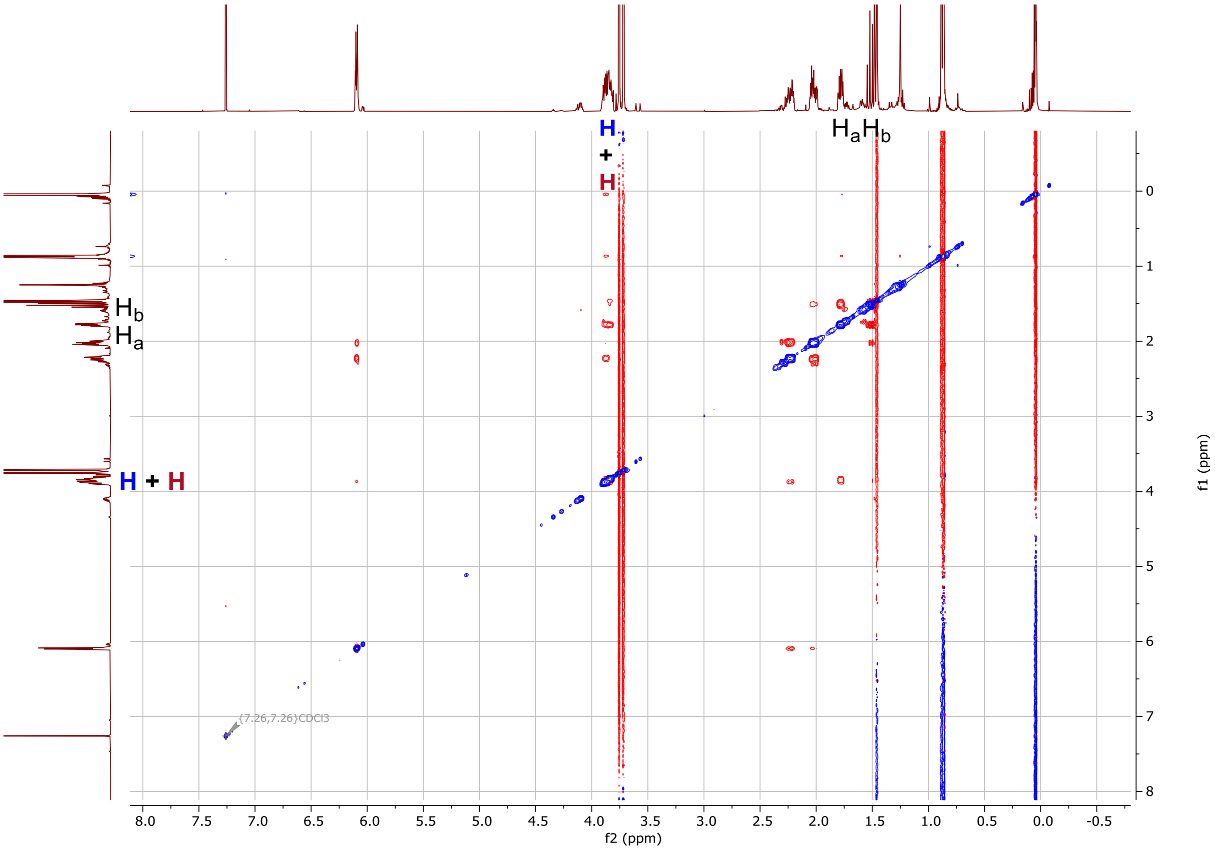

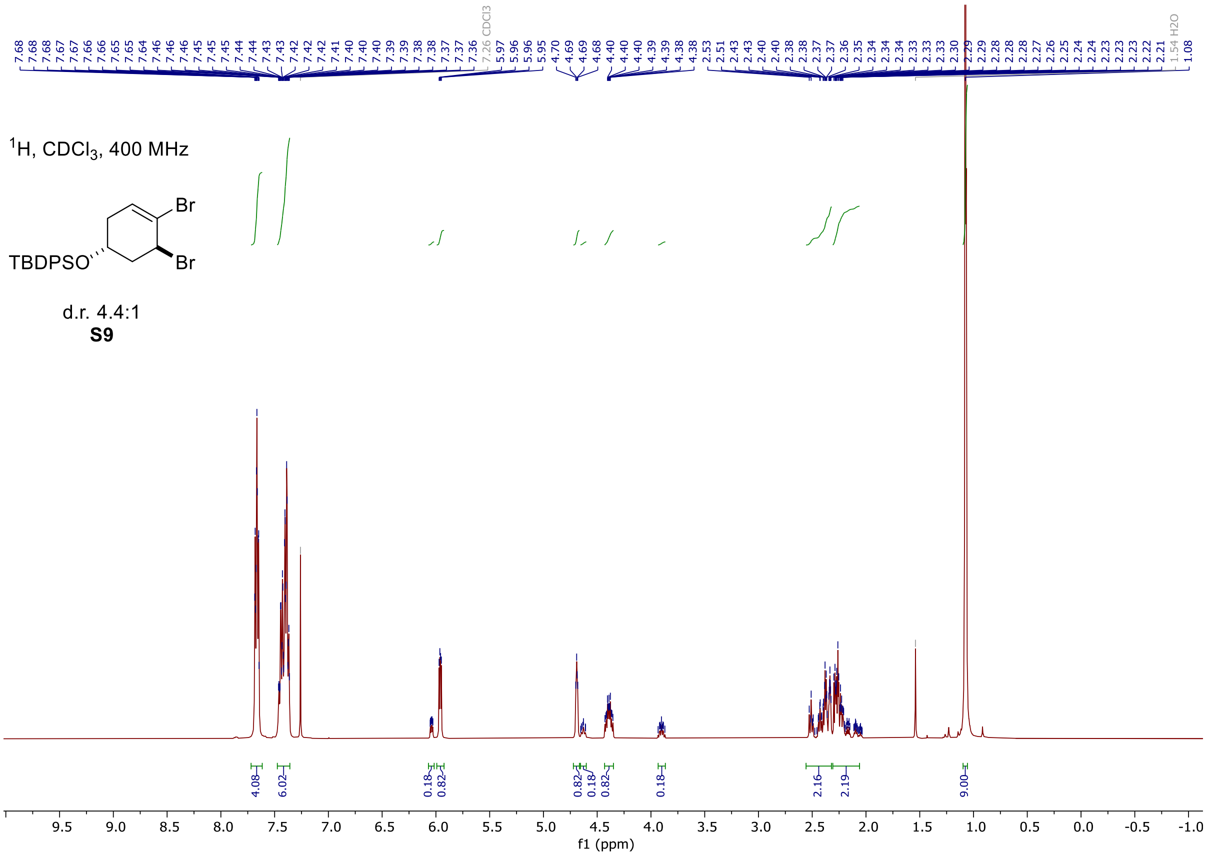

^{1}H, CDCl$_3$, 400 MHz
TBDPSO
Br
Br
d.r. 4.4:1
S9
7.68 7.68 7.68 7.67 7.67 7.66 7.66 7.65 7.65 7.64 7.46 7.46 7.46 7.45 7.45 7.45 7.44 7.44 7.43 7.43 7.42 7.42 7.42 7.41 7.40 7.40 7.40 7.39 7.39 7.38 7.38 7.37 7.37 7.36
7.26 CDCl3
5.97 5.96 5.96 5.95 4.70 4.69 4.69 4.68 4.40 4.40 4.40 4.39 4.39 4.38 4.38 2.53 2.51 2.43 2.43 2.40 2.40 2.38 2.38 2.37 2.37 2.36 2.35 2.34 2.34 2.34 2.33 2.33 2.33 2.30 2.29 2.29 2.28 2.28 2.28 2.27 2.26 2.25 2.24 2.24 2.23 2.23 2.23 2.22 2.21
1.54 H2O
1.08
4.08 6.02 0.18 0.82 0.82 0.18 0.82 0.18 2.16 2.19 9.00
9.5 9.0 8.5 8.0 7.5 7.0 6.5 6.0 5.5 5.0 4.5 4.0 3.5 3.0 2.5 2.0 1.5 1.0 0.5 0.0 -0.5 -1.0
f1 (ppm)

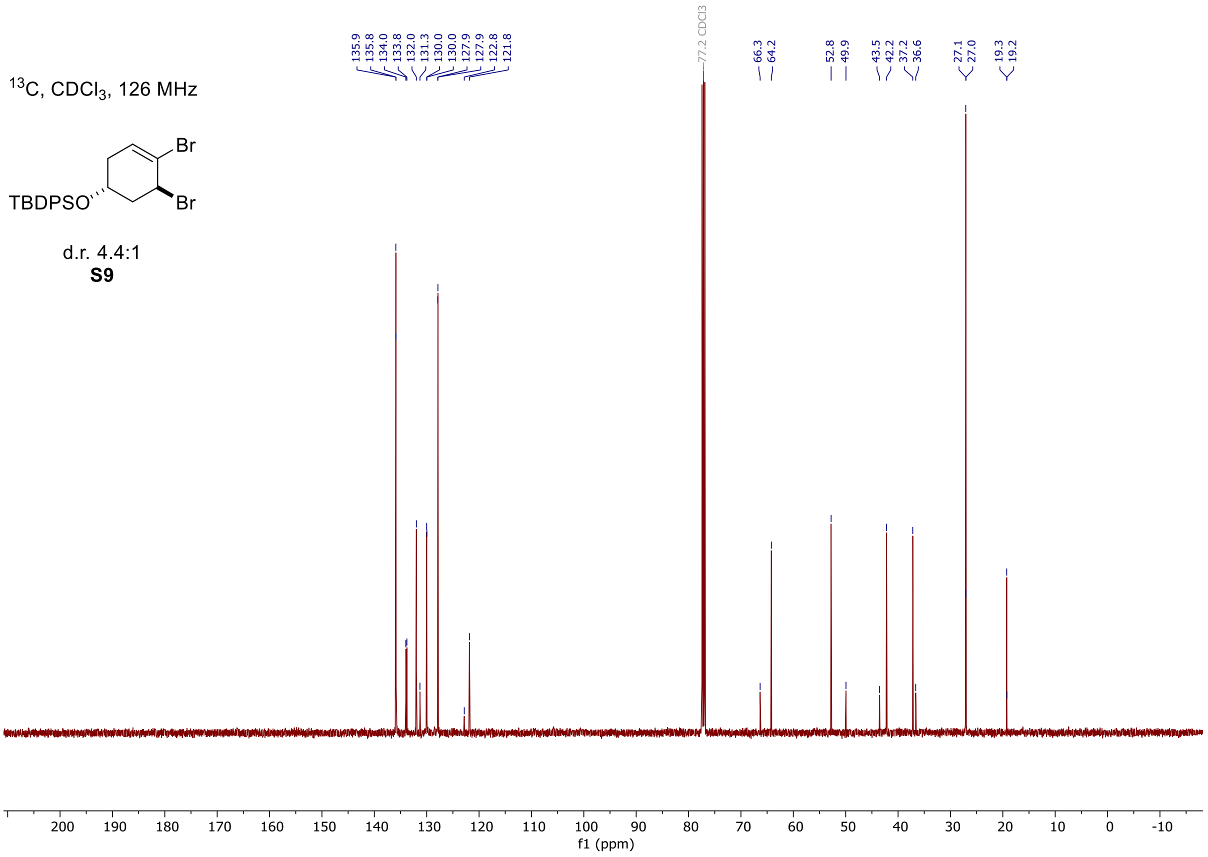

^{13}C, CDCl$_3$, 126 MHz
TBDPSO
Br
Br
d.r. 4.4:1
S9
135.9 135.8 134.0 133.8 132.0 131.3 130.0 130.0 127.9 127.9 122.8 121.8
77.2 CDCl3
66.3 64.2 52.8 49.9 43.5 42.2 37.2 36.6 27.1 27.0 19.3 19.2
200 190 180 170 160 150 140 130 120 110 100 90 80 70 60 50 40 30 20 10 0 -10
f1 (ppm)

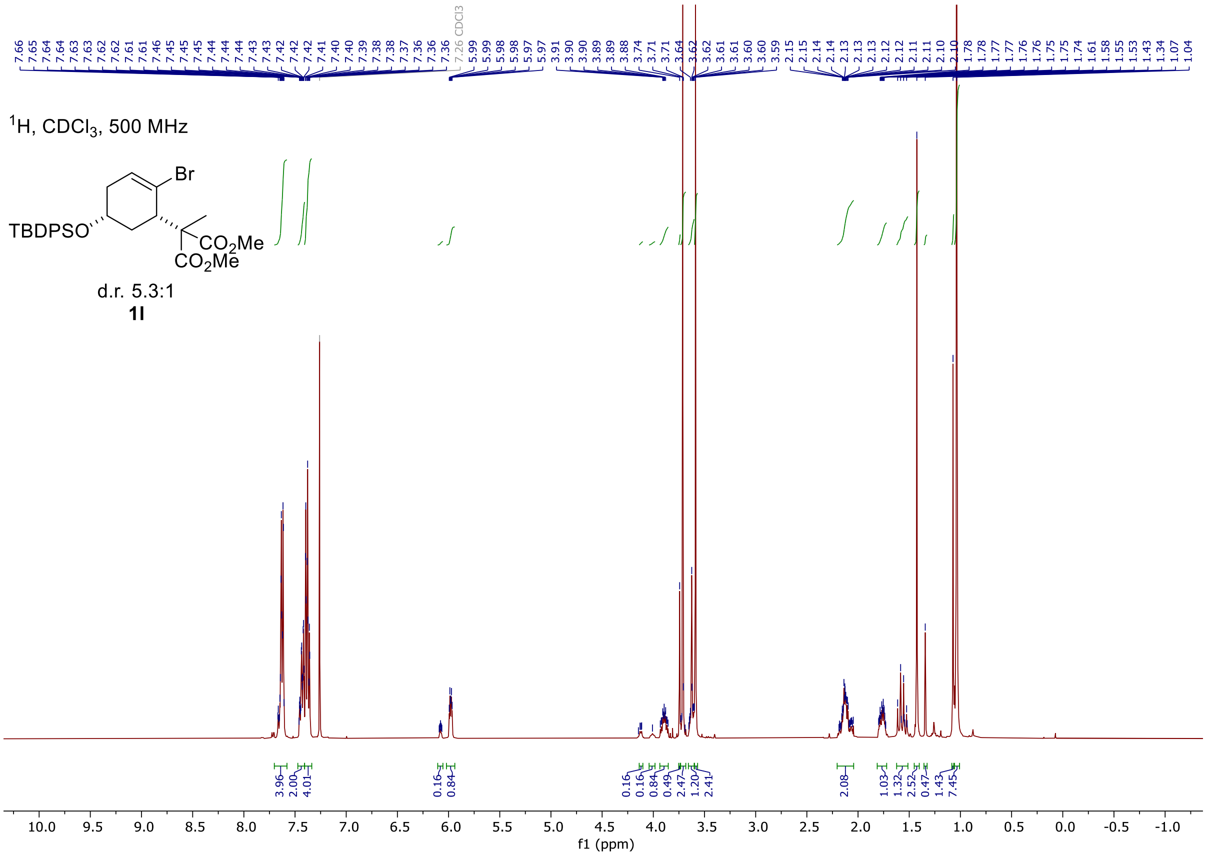

1H, CDCl3, 500 MHz
TBDPSO
Br
CO2Me
CO2Me
d.r. 5.3:1
1l
f1 (ppm)

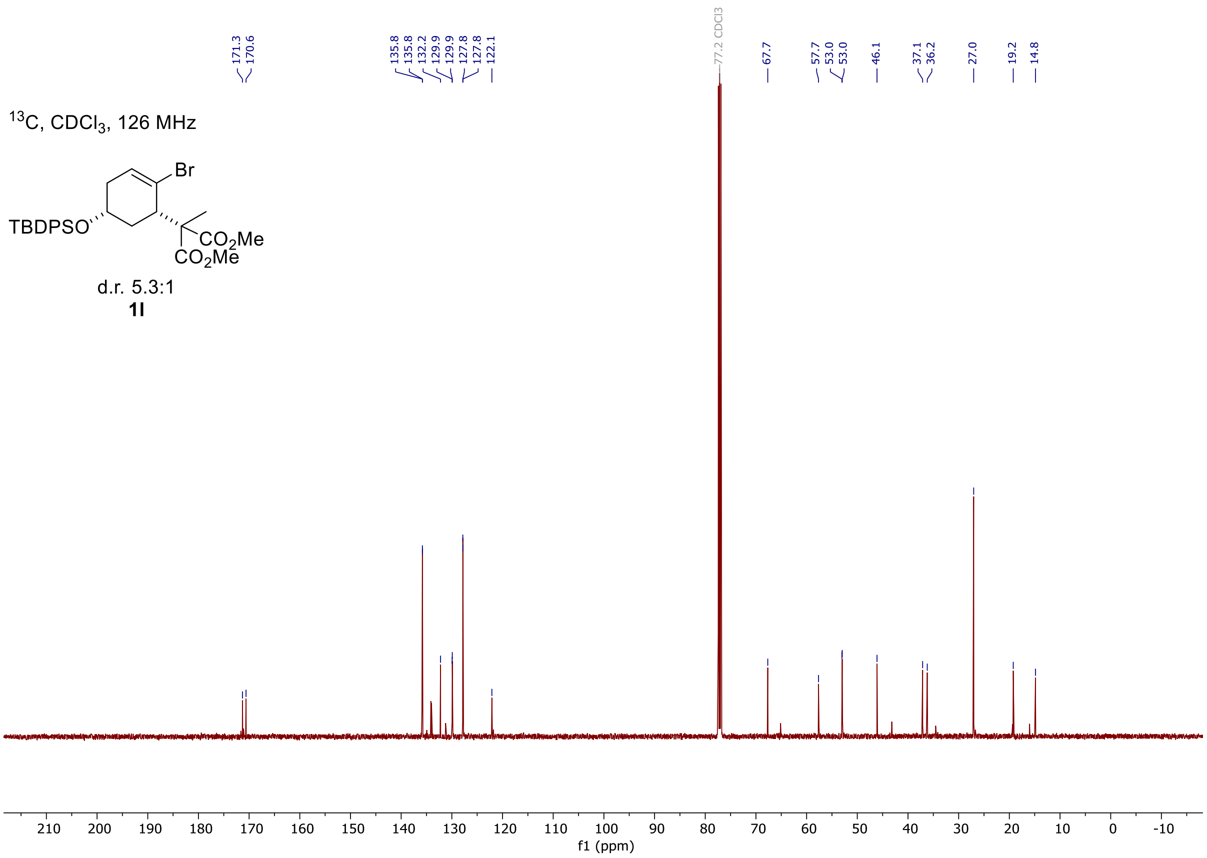

13C, CDCl3, 126 MHz
TBDPSO
Br
CO2Me
CO2Me
d.r. 5.3:1
1l
171.3
170.6
135.8
135.8
132.2
129.9
129.9
127.8
127.8
122.1
77.2 CDCl3
67.7
57.7
53.0
53.0
46.1
37.1
36.2
27.0
19.2
14.8
f1 (ppm)

NOESY of **1l**.

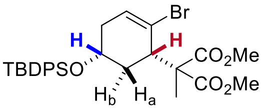

1l

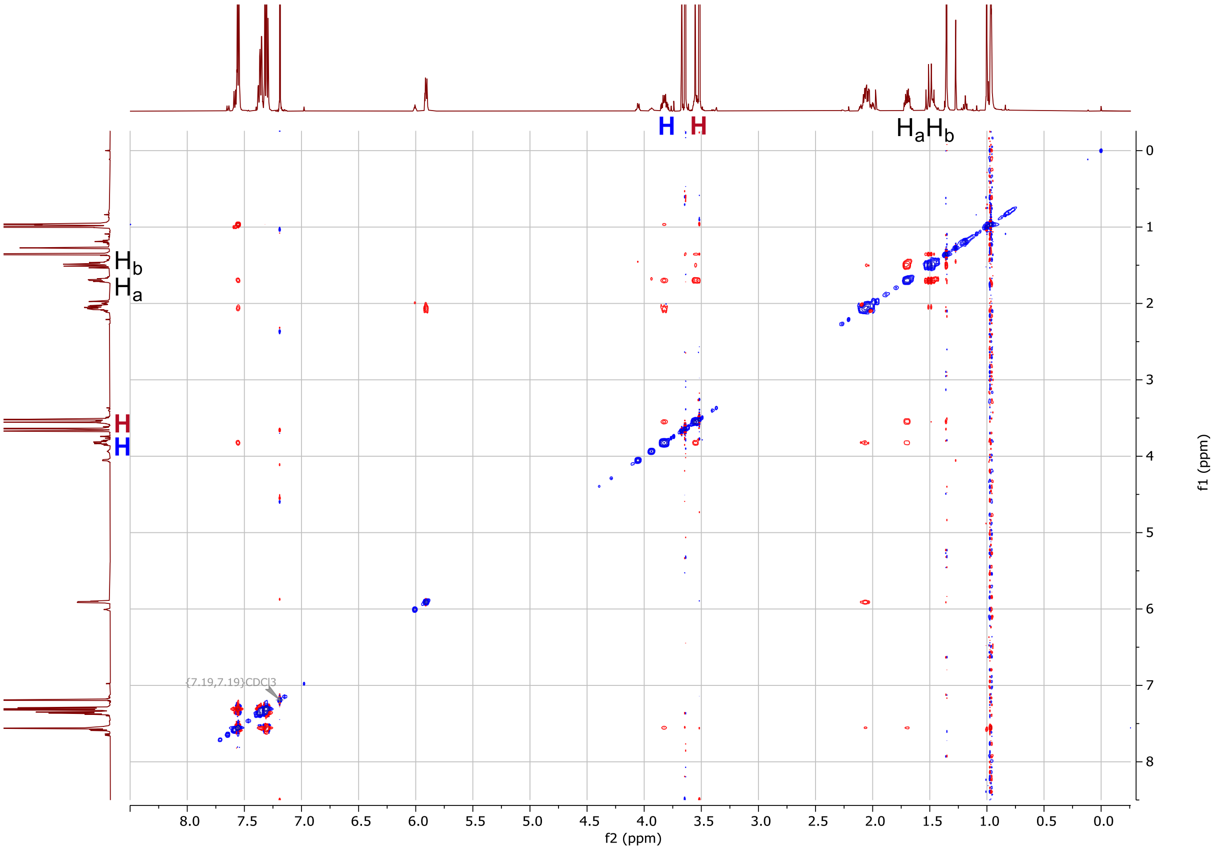

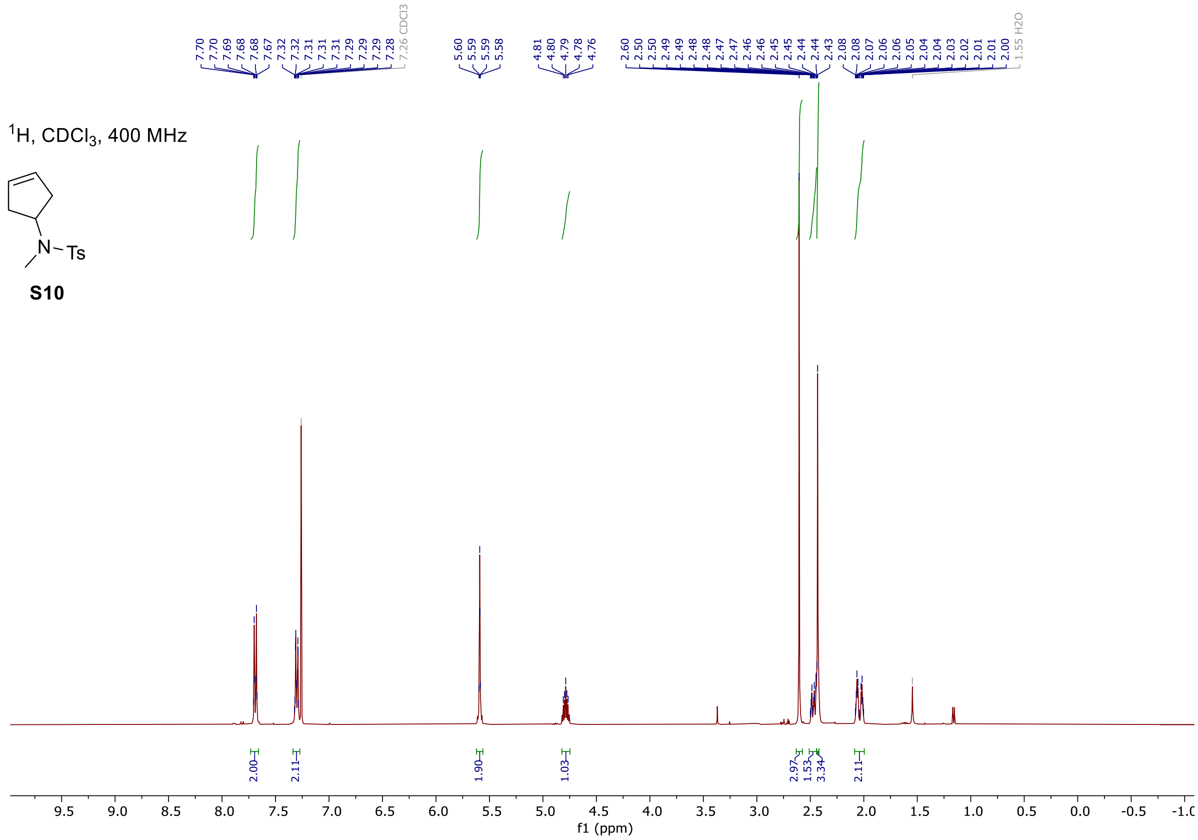
1H, CDCl3, 400 MHz
S10
f1 (ppm)

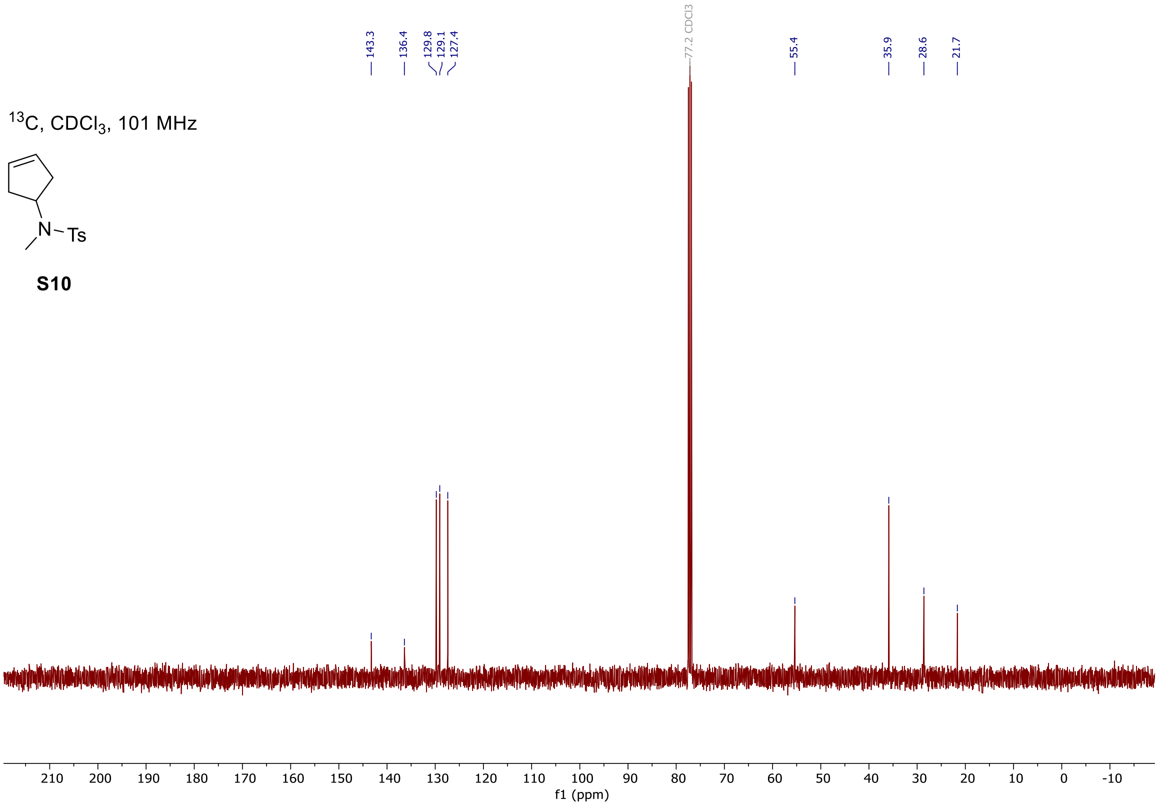
13C, CDCl3, 101 MHz
S10
143.3
136.4
129.8
129.1
127.4
77.2 CDCl3
55.4
35.9
28.6
21.7
f1 (ppm)

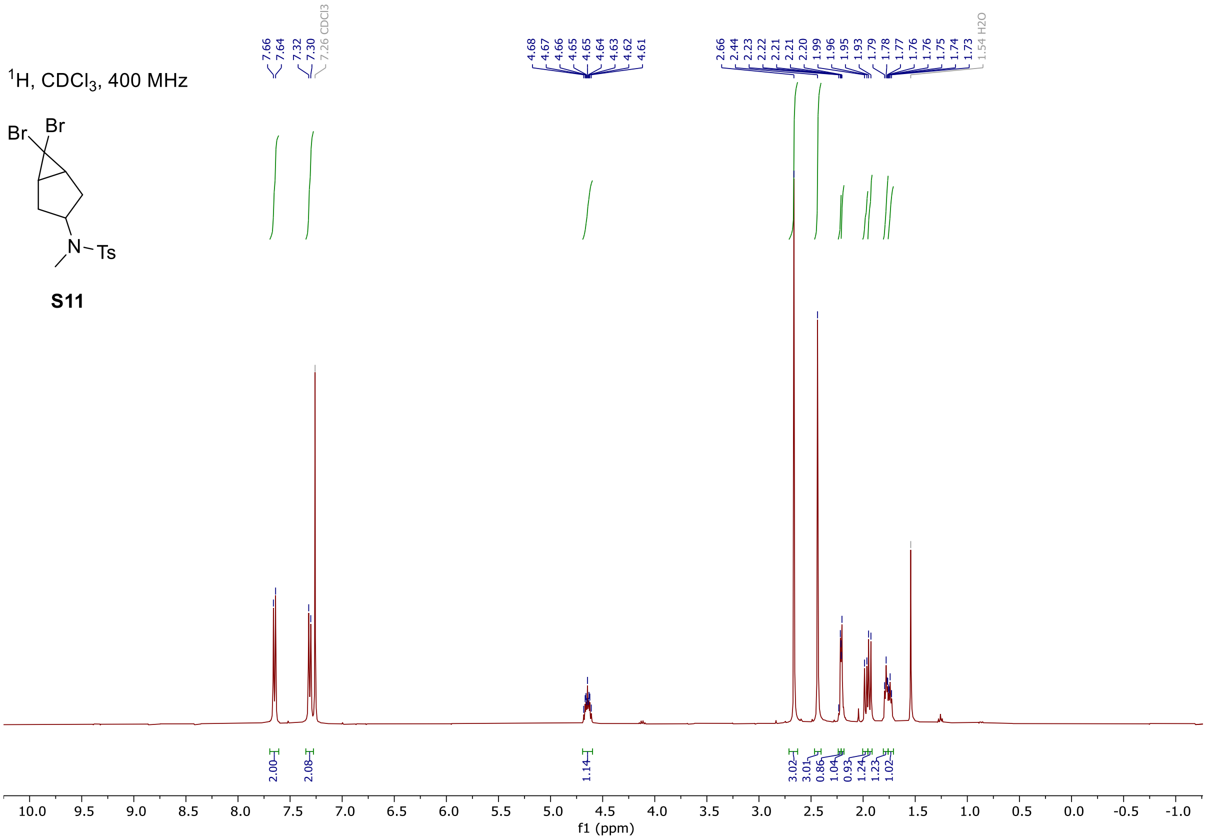

^{1}H, $CDCl_3$, 400 MHz
Br
Br
N
Ts
S11
7.66
7.64
7.32
7.30
7.26 CDCl3
4.68
4.67
4.66
4.65
4.65
4.64
4.63
4.62
4.61
2.66
2.44
2.23
2.22
2.21
2.21
2.20
1.99
1.96
1.95
1.93
1.79
1.78
1.77
1.76
1.76
1.75
1.74
1.73
1.54 H2O
2.00
2.08
1.14
3.02
3.01
0.86
1.04
0.93
1.24
1.23
1.02
f1 (ppm)

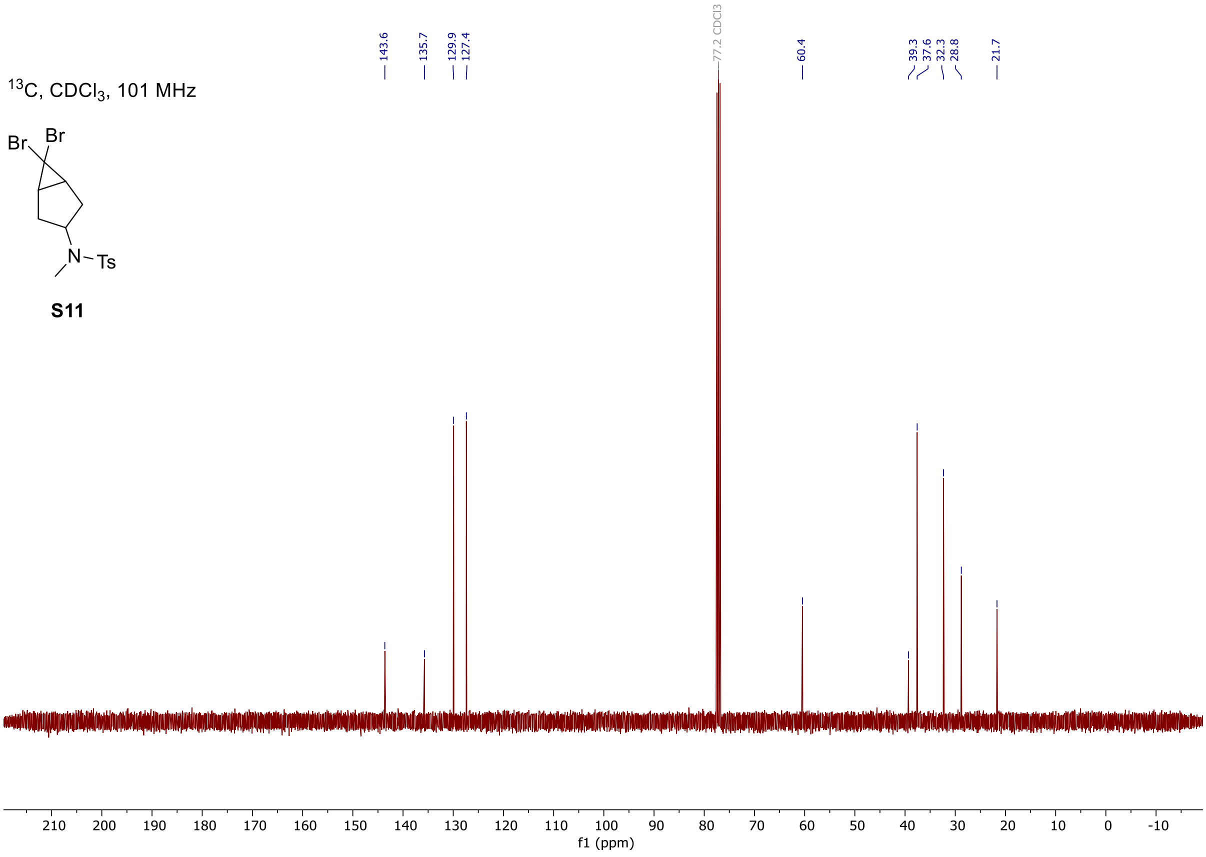

^{13}C, $CDCl_3$, 101 MHz
Br
Br
N
Ts
S11
143.6
135.7
129.9
127.4
77.2 CDCl3
60.4
39.3
37.6
32.3
28.8
21.7
f1 (ppm)

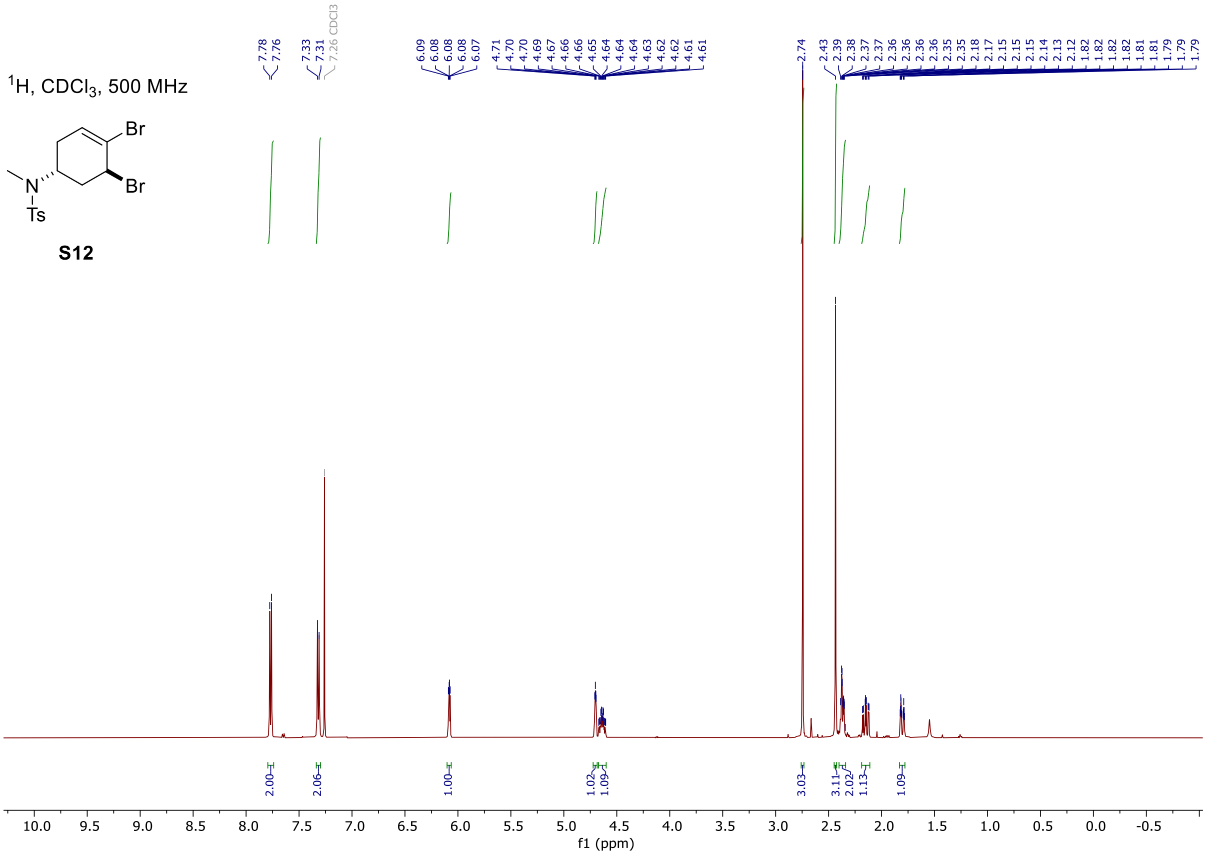
7.78
7.76
7.33
7.31
7.26 CDCl3
6.09
6.08
6.08
6.07
4.71
4.70
4.69
4.67
4.66
4.65
4.64
4.64
4.63
4.62
4.61
4.61
2.74
2.43
2.39
2.38
2.37
2.37
2.36
2.36
2.36
2.35
2.18
2.17
2.15
2.15
2.14
2.13
2.12
1.82
1.82
1.82
1.81
1.81
1.79
1.79
^{1}H, CDCl$_3$, 500 MHz
Br
Br
N
Ts
2.00
2.06
1.00
1.02
1.09
3.03
3.11
2.02
1.13
1.09
f1 (ppm)

S12

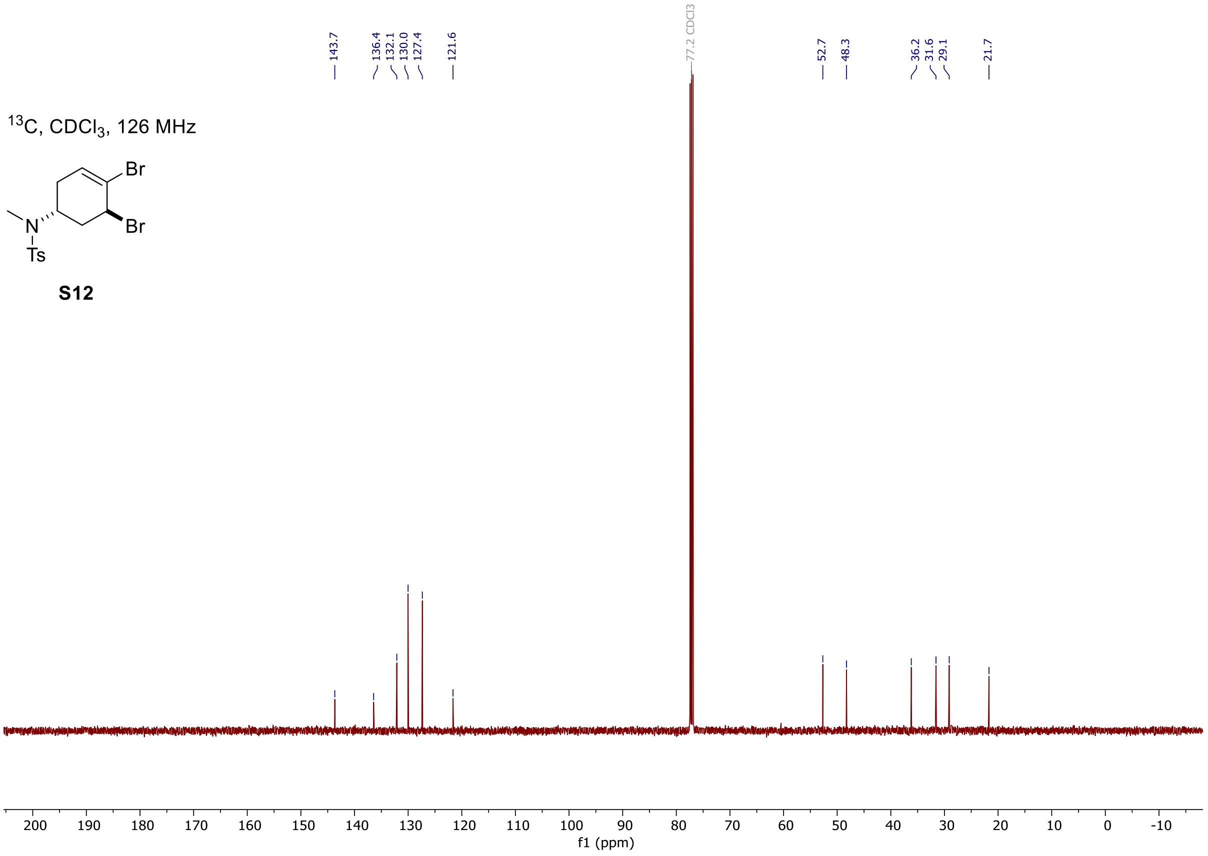
143.7
136.4
132.1
130.0
127.4
121.6
77.2 CDCl3
52.7
48.3
36.2
31.6
29.1
21.7
^{13}C, CDCl$_3$, 126 MHz
Br
Br
N
Ts
f1 (ppm)

S12

NOESY of **S12**.

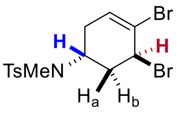

S12

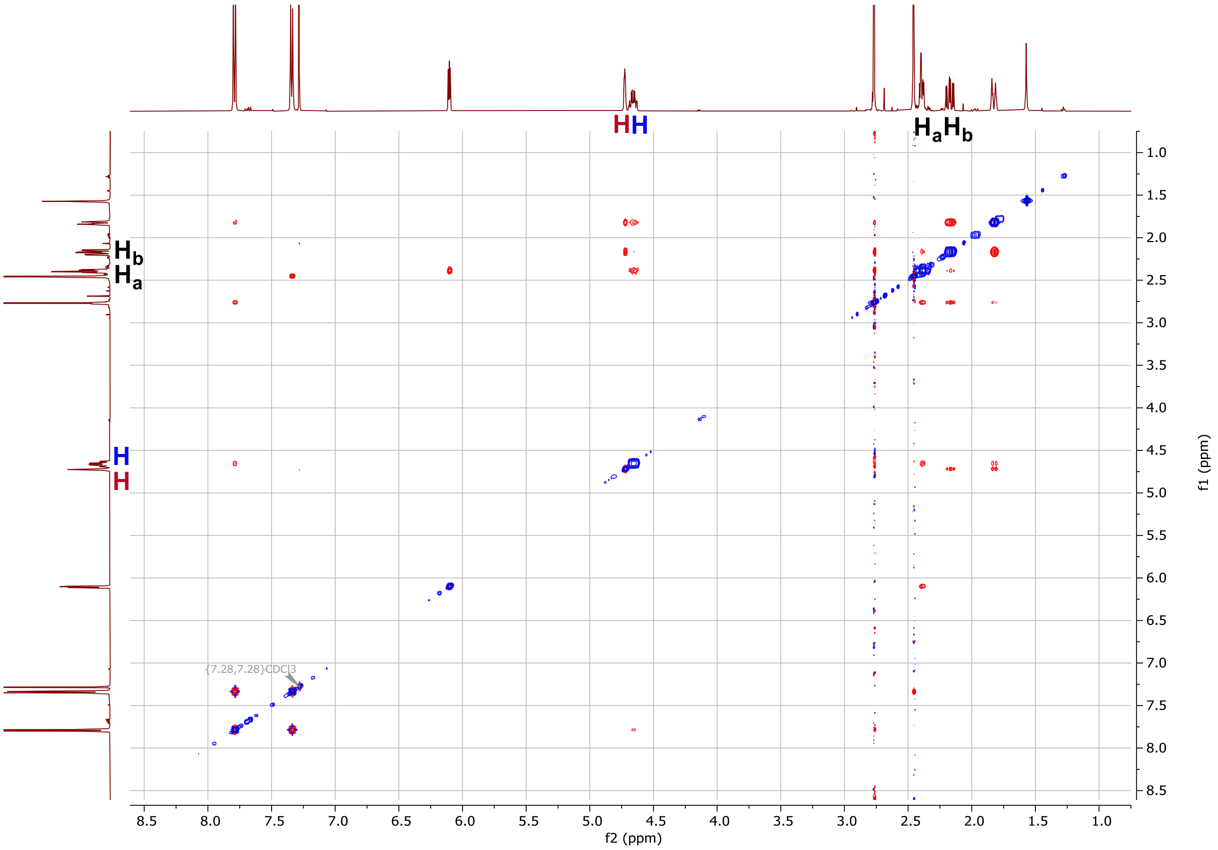

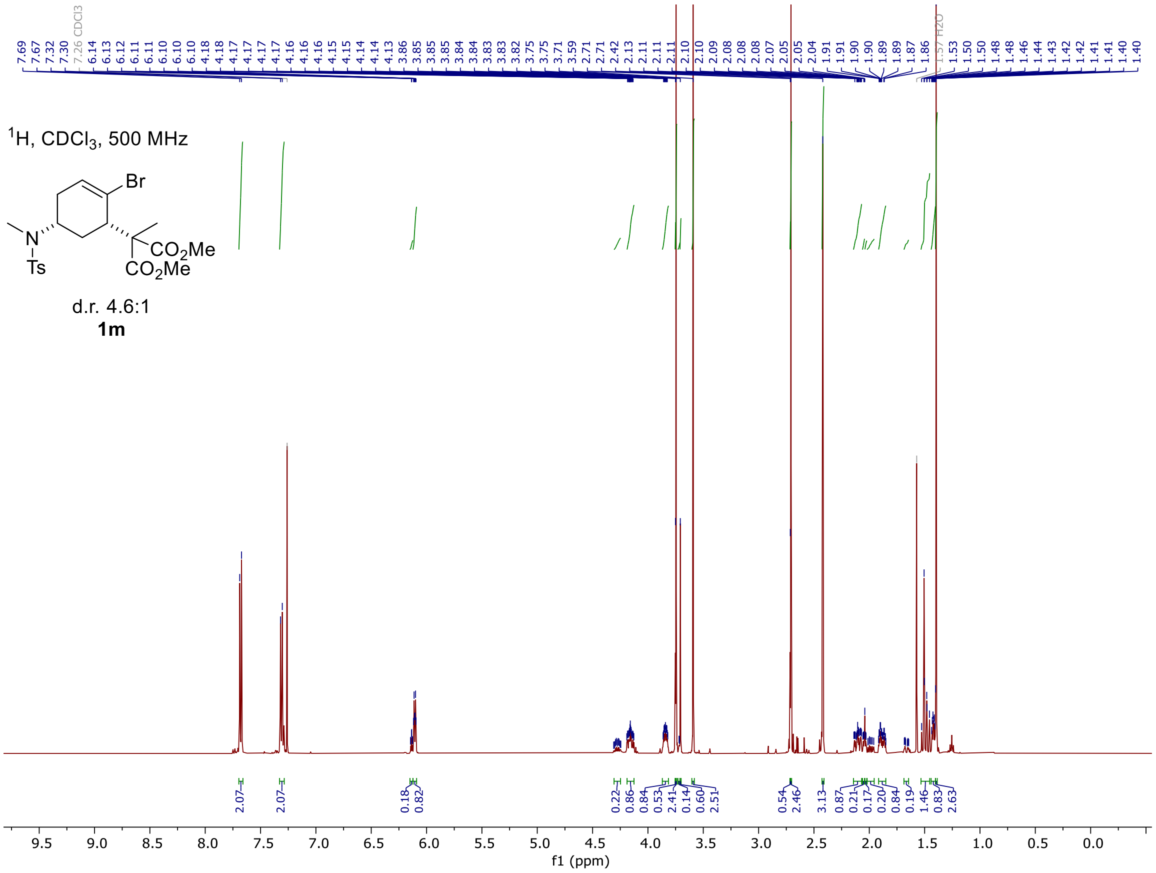
7.69 7.67 7.32 7.30 7.26 CDCl3 6.14 6.13 6.12 6.11 6.11 6.10 6.10 6.10 4.18 4.18 4.17 4.17 4.17 4.16 4.16 4.15 4.15 4.14 4.14 4.13 3.86 3.85 3.85 3.84 3.84 3.83 3.83 3.82 3.75 3.75 3.71 3.59 2.71 2.71 2.42 2.13 2.11 2.11 2.11 2.10 2.10 2.09 2.08 2.08 2.08 2.07 2.05 2.05 2.04 1.91 1.91 1.90 1.90 1.89 1.89 1.87 1.86 1.57 H2O 1.53 1.50 1.50 1.48 1.46 1.44 1.43 1.42 1.42 1.41 1.41 1.40 1.40
^{1}H, $CDCl_3$, 500 MHz
Br
N
Ts
CO_2Me
CO_2Me
d.r. 4.6:1
1m
2.07 2.07 0.18 0.82 0.22 0.86 0.84 0.53 2.41 0.14 0.60 2.51 0.54 2.46 3.13 0.87 0.21 0.17 0.20 0.84 0.19 1.46 0.83 2.63
9.5 9.0 8.5 8.0 7.5 7.0 6.5 6.0 5.5 5.0 4.5 4.0 3.5 3.0 2.5 2.0 1.5 1.0 0.5 0.0
f1 (ppm)

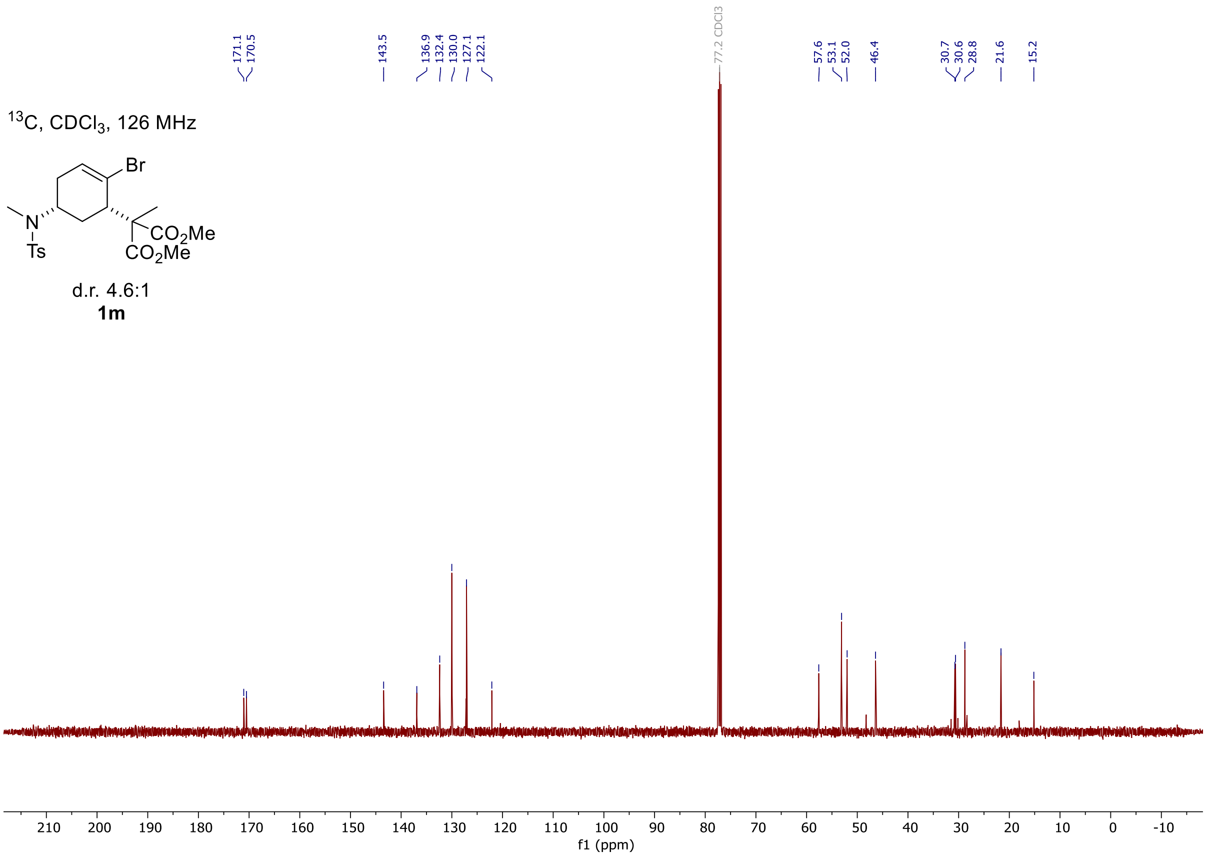
171.1 170.5 143.5 136.9 132.4 130.0 127.1 122.1 77.2 CDCl3 57.6 53.1 52.0 46.4 30.7 30.6 28.8 21.6 15.2
^{13}C, $CDCl_3$, 126 MHz
Br
N
Ts
CO_2Me
CO_2Me
d.r. 4.6:1
1m
210 200 190 180 170 160 150 140 130 120 110 100 90 80 70 60 50 40 30 20 10 0 -10
f1 (ppm)

NOESY of **1m**.

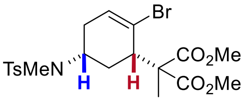

1m

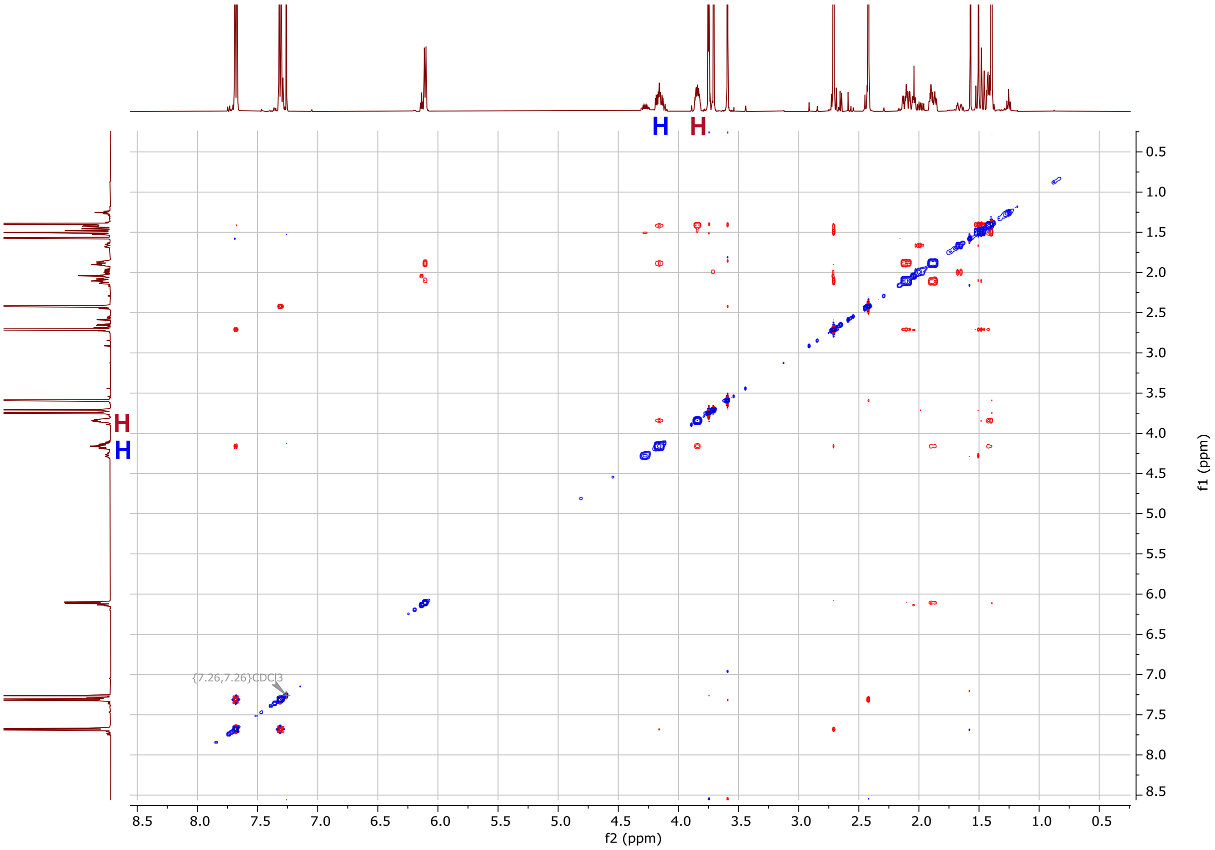

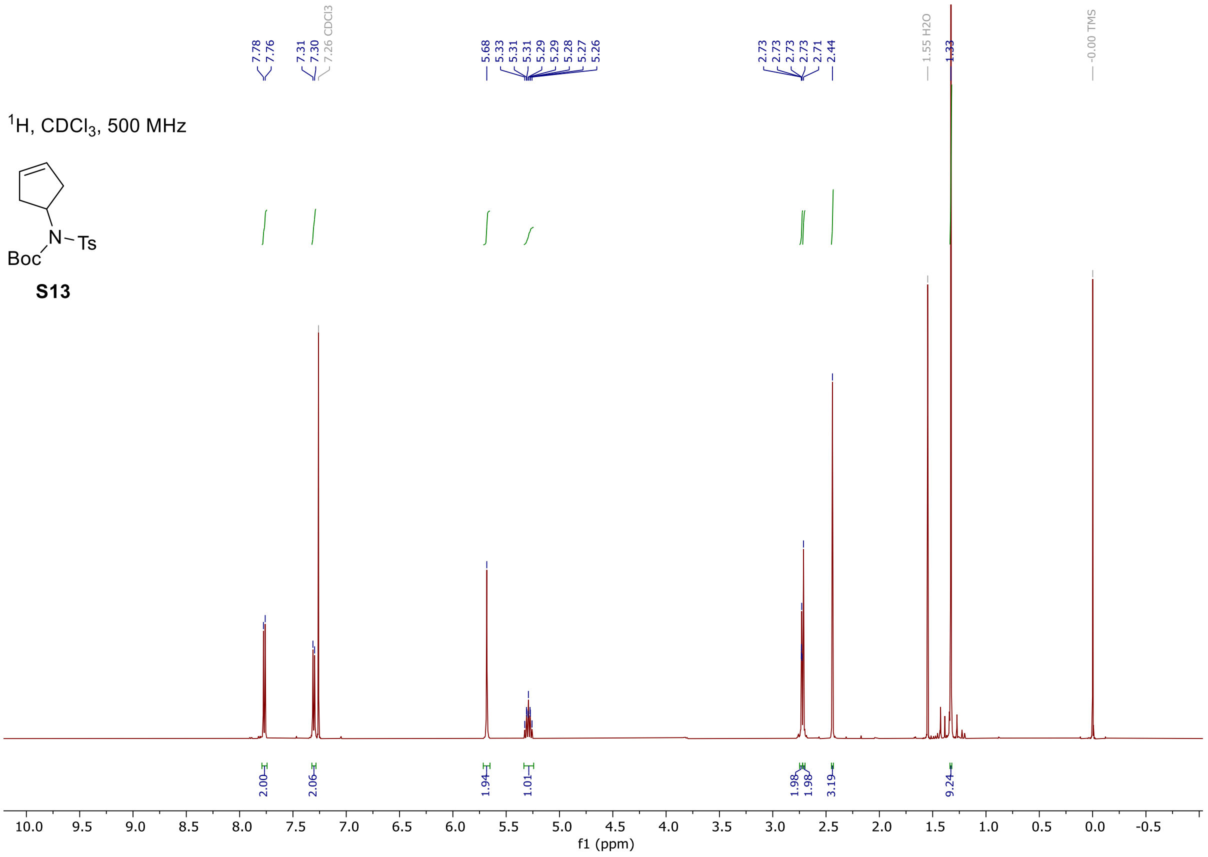
1H, CDCl3, 500 MHz
N
Ts
Boc
S13
7.78
7.76
7.31
7.30
7.26 CDCl3
5.68
5.33
5.31
5.31
5.29
5.29
5.28
5.27
5.26
2.73
2.73
2.73
2.73
2.71
2.44
1.55 H2O
1.33
-0.00 TMS
2.00
2.06
1.94
1.01
1.98
1.98
3.19
9.24
10.0 9.5 9.0 8.5 8.0 7.5 7.0 6.5 6.0 5.5 5.0 4.5 4.0 3.5 3.0 2.5 2.0 1.5 1.0 0.5 0.0 -0.5
f1 (ppm)

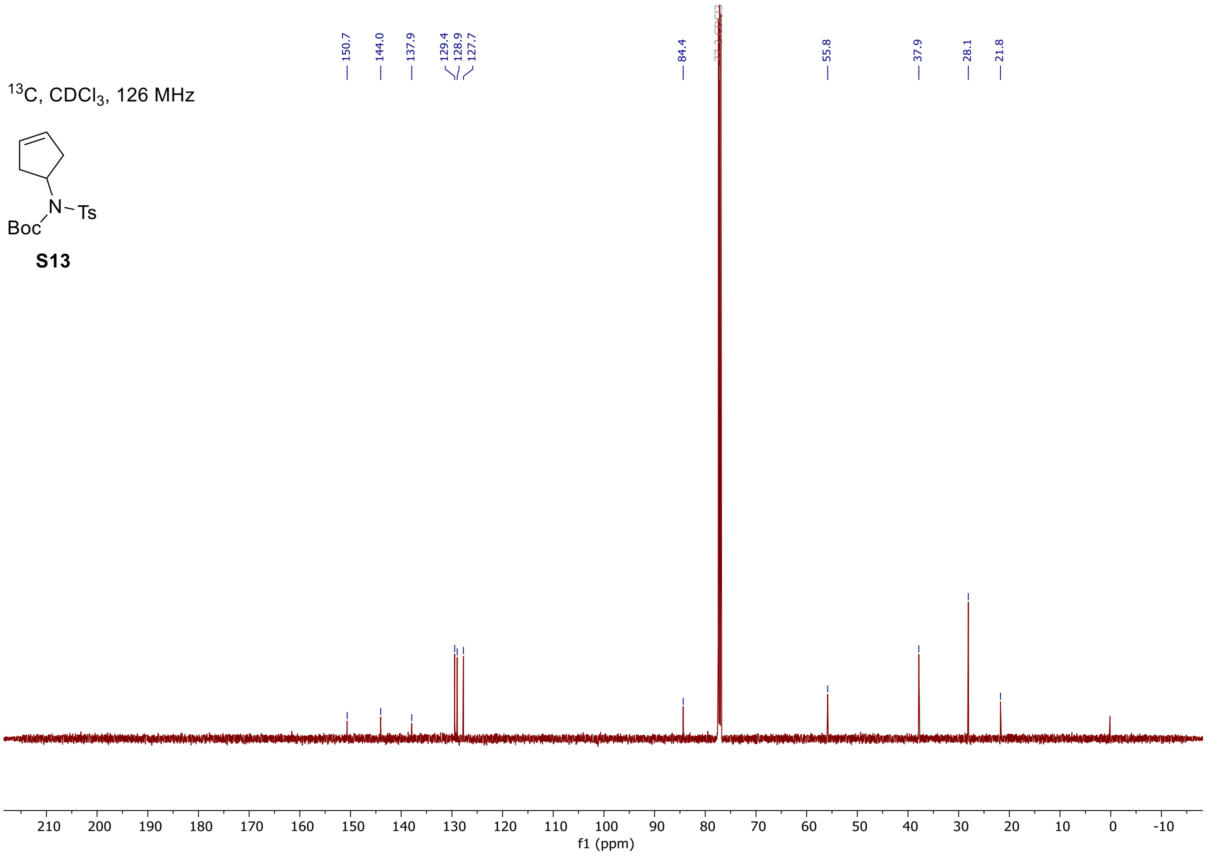
13C, CDCl3, 126 MHz
N
Ts
Boc
S13
150.7
144.0
137.9
129.4
128.9
127.7
84.4
77.2 CDCl3
55.8
37.9
28.1
21.8
210 200 190 180 170 160 150 140 130 120 110 100 90 80 70 60 50 40 30 20 10 0 -10
f1 (ppm)

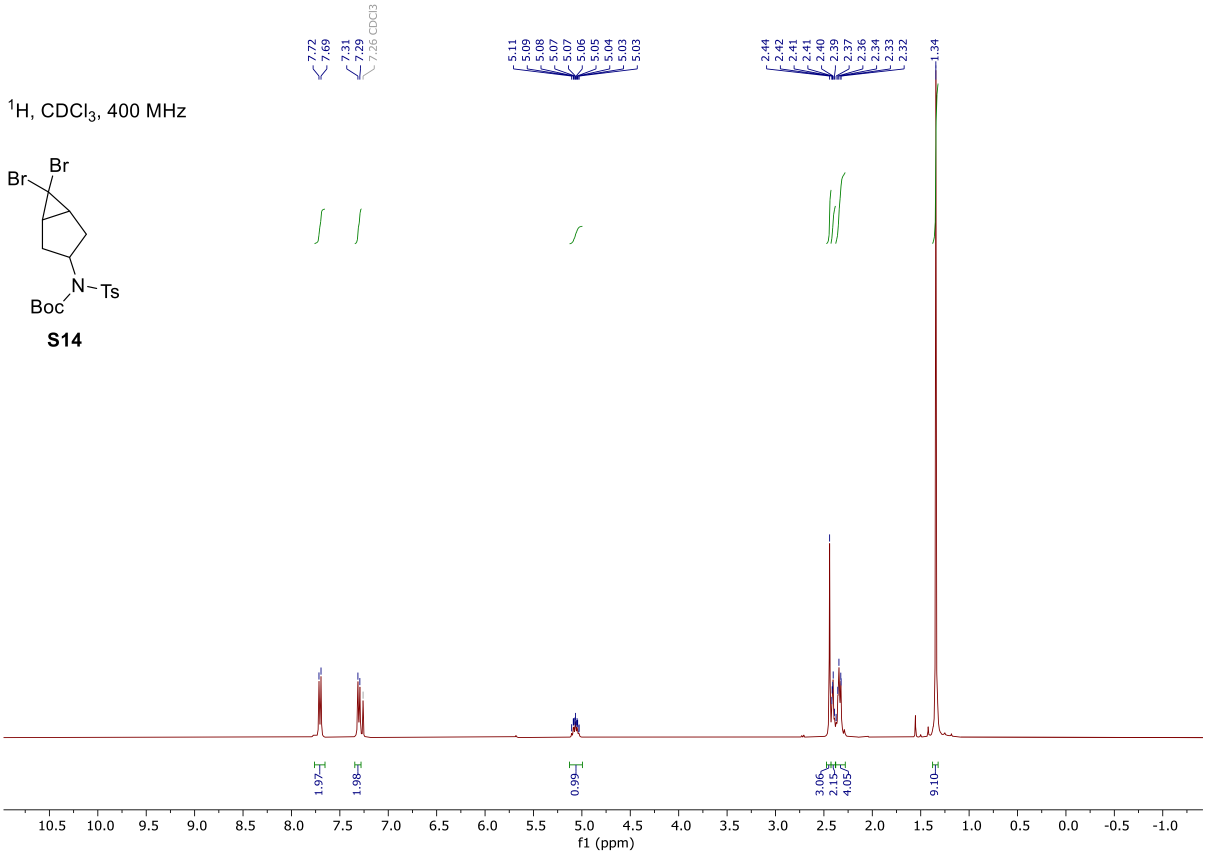
1H, CDCl3, 400 MHz
Br
Br
N
Ts
Boc
S14
7.72
7.69
7.31
7.29
7.26 CDCl3
5.11
5.09
5.08
5.07
5.07
5.06
5.05
5.04
5.03
5.03
2.44
2.42
2.41
2.41
2.40
2.39
2.37
2.36
2.34
2.33
2.32
1.34
1.97
1.98
0.99
3.06
2.15
4.05
9.10
f1 (ppm)

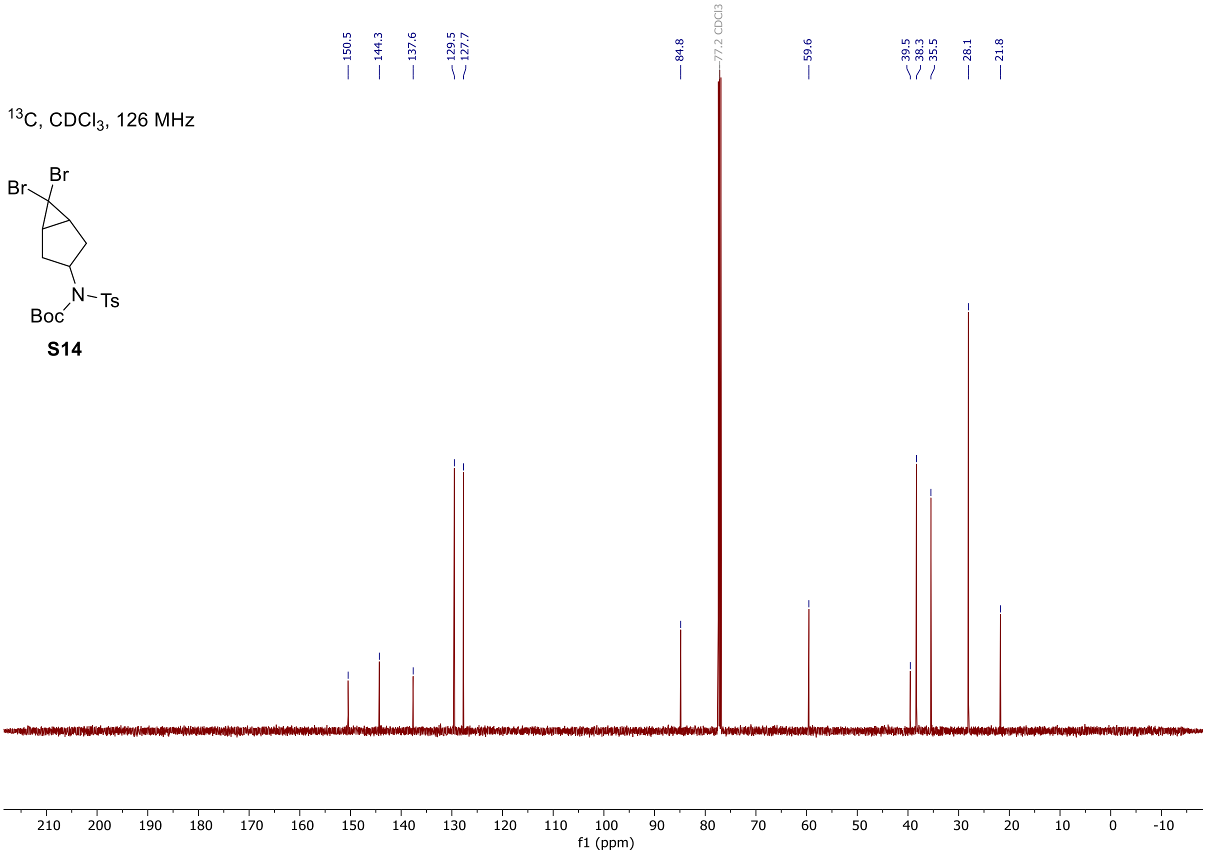
13C, CDCl3, 126 MHz
Br
Br
N
Ts
Boc
S14
150.5
144.3
137.6
129.5
127.7
84.8
77.2 CDCl3
59.6
39.5
38.3
35.5
28.1
21.8
f1 (ppm)

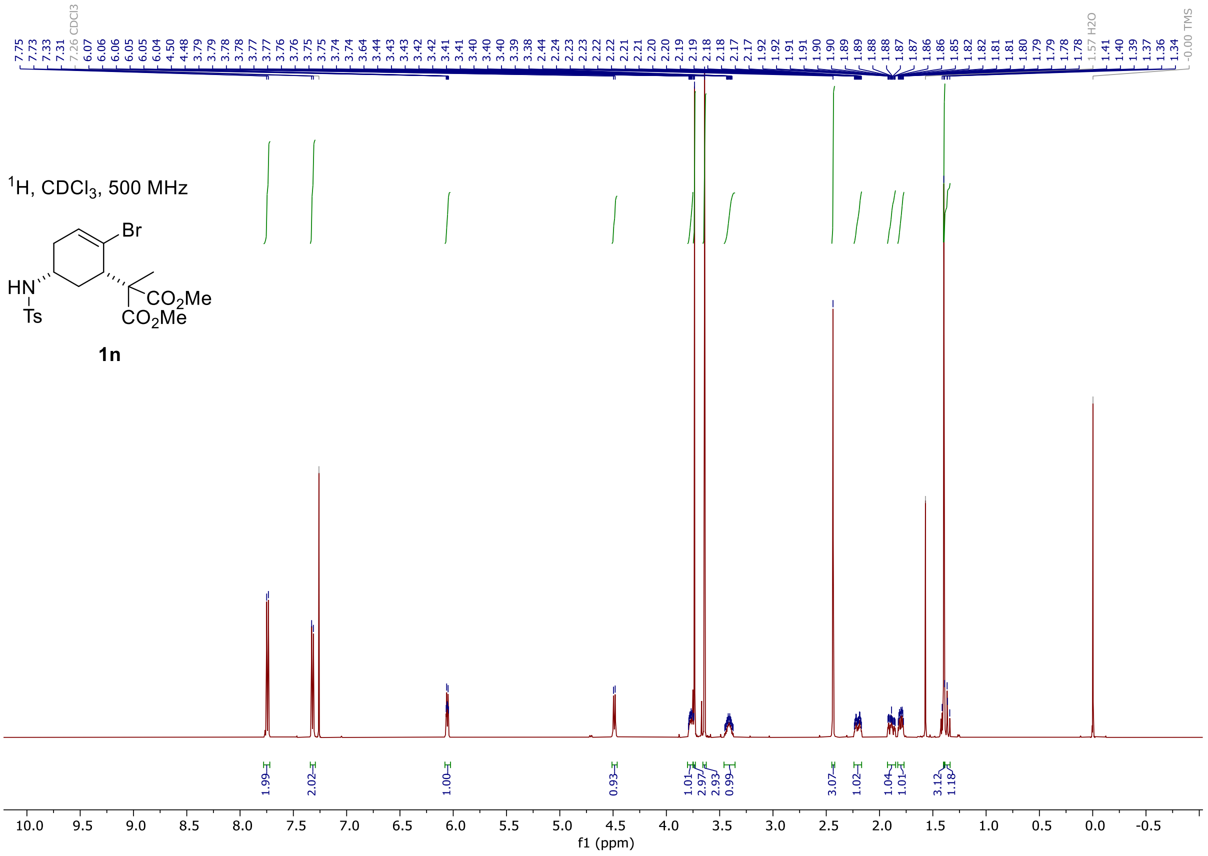

^{1}H, $CDCl_3$, 500 MHz
Br
HN
Ts
CO_2Me
CO_2Me
1n
f1 (ppm)

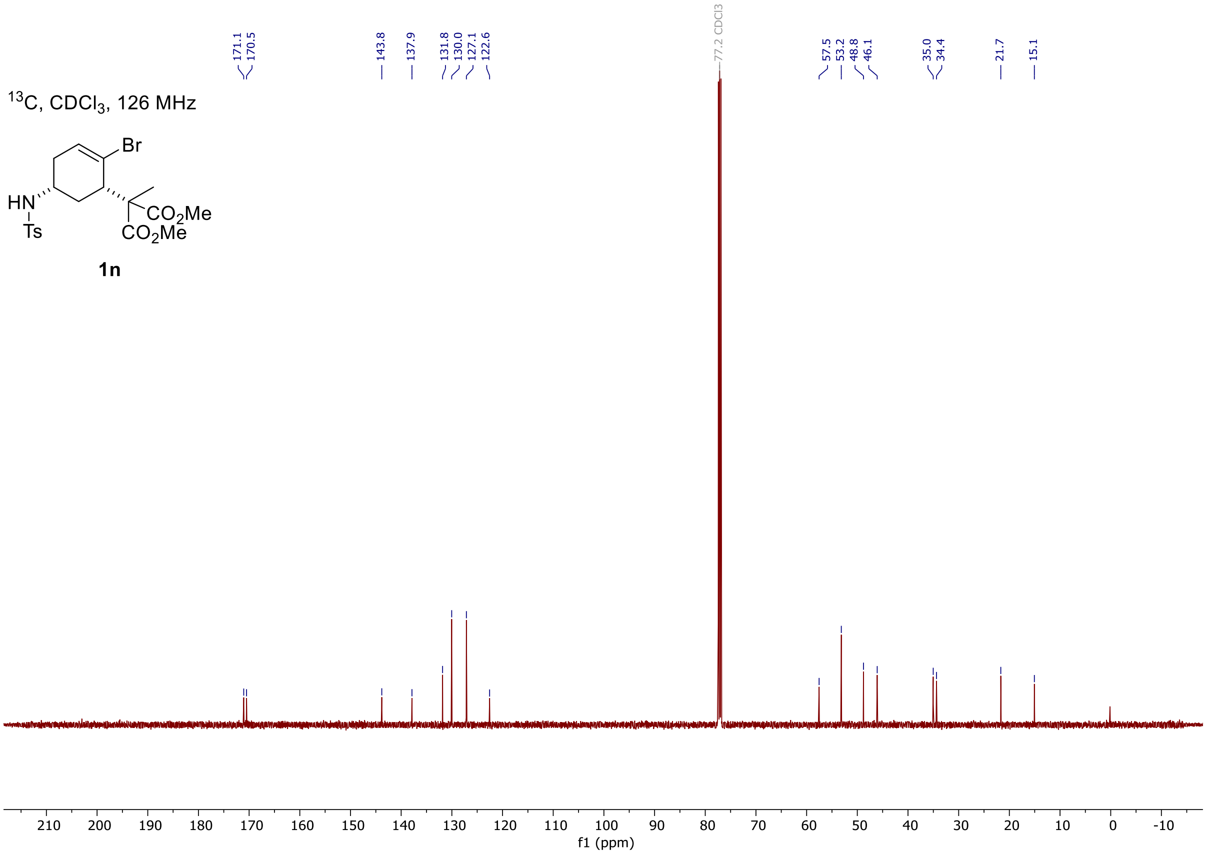

^{13}C, $CDCl_3$, 126 MHz
Br
HN
Ts
CO_2Me
CO_2Me
1n
171.1
170.5
143.8
137.9
131.8
130.0
127.1
122.6
77.2 CDCl3
57.5
53.2
48.8
46.1
35.0
34.4
21.7
15.1
f1 (ppm)

NOESY of **1n**.

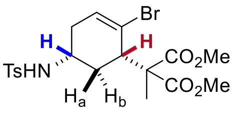

1n

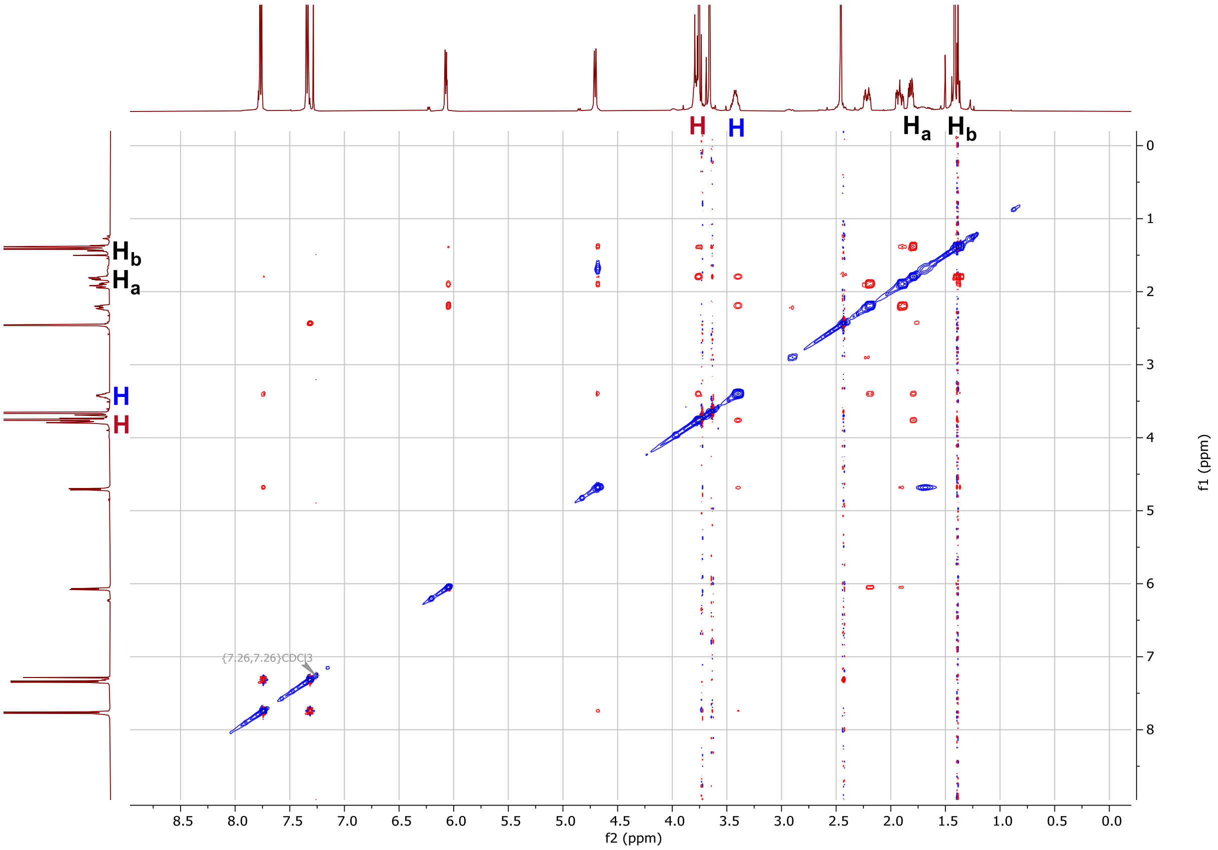

^{1}H, $CDCl_3$, 400 MHz

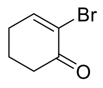

S17

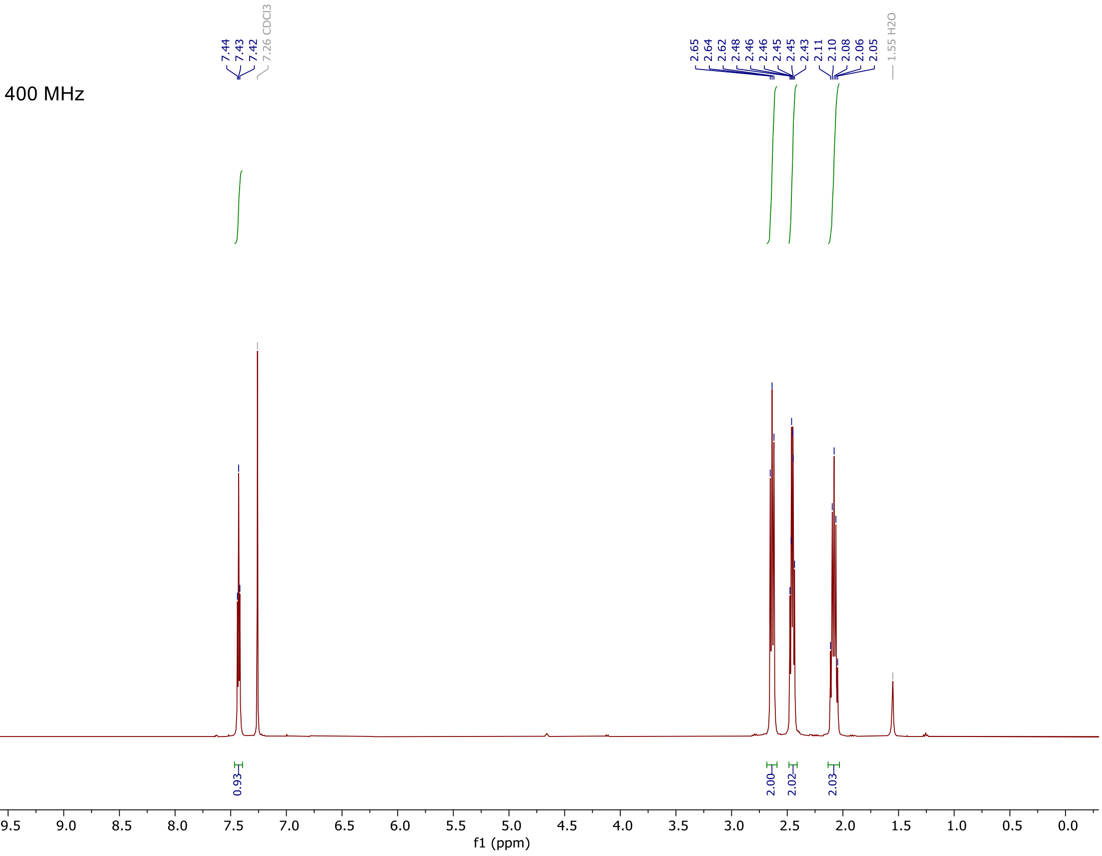

^{13}C, $CDCl_3$, 126 MHz

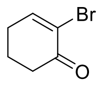

S17

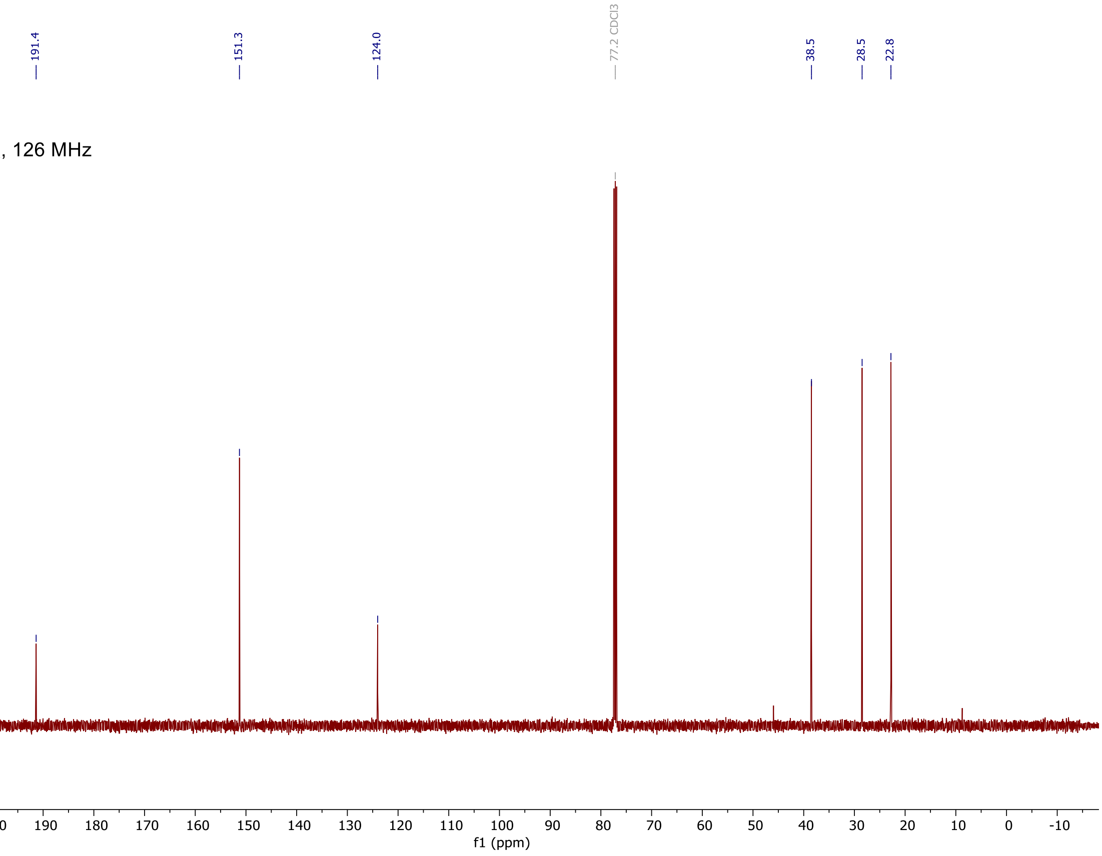

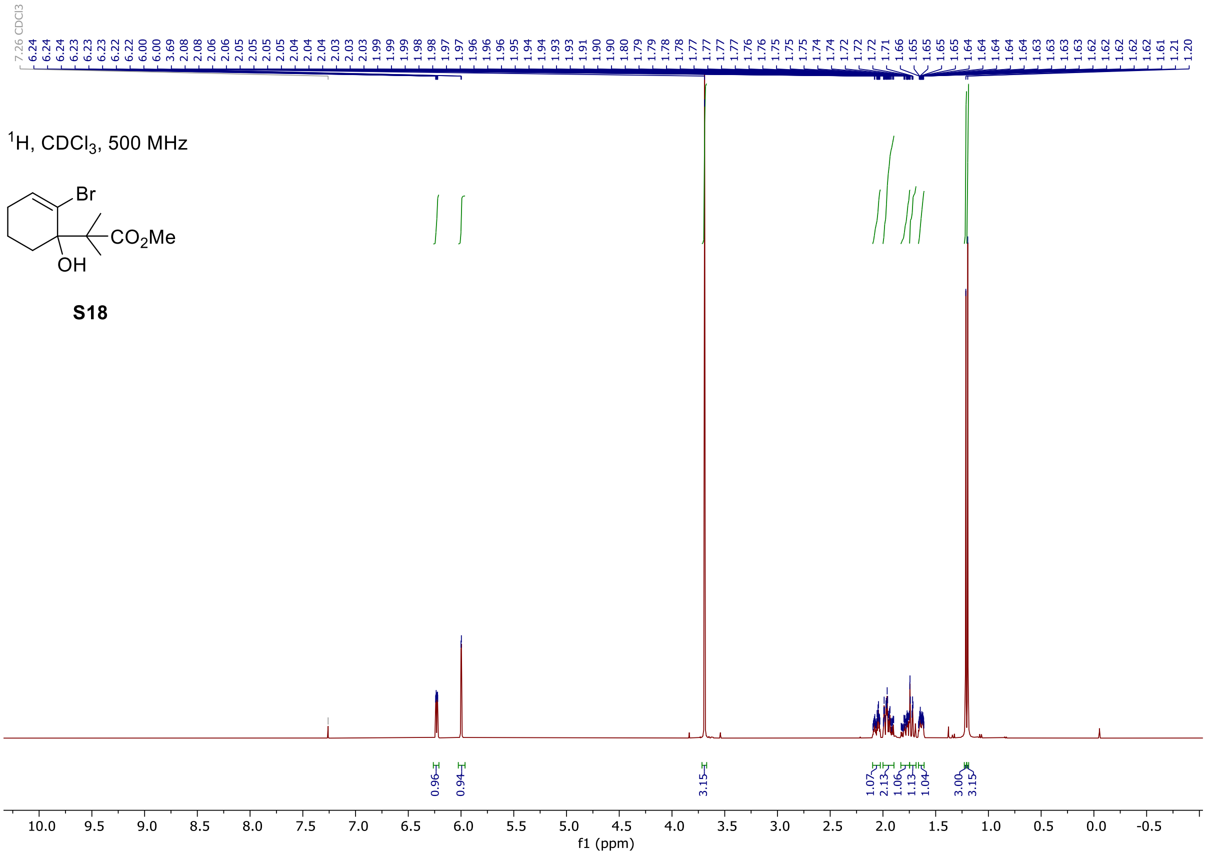
7.26 CDCl3
6.24 6.24 6.24 6.23 6.23 6.23 6.22 6.22 6.00 3.69 2.08 2.08 2.06 2.06 2.05 2.05 2.05 2.05 2.04 2.04 2.04 2.03 2.03 2.03 1.99 1.99 1.99 1.99 1.98 1.98 1.97 1.97 1.96 1.96 1.96 1.95 1.94 1.94 1.93 1.93 1.91 1.90 1.90 1.80 1.79 1.79 1.78 1.78 1.78 1.77 1.77 1.77 1.77 1.76 1.76 1.75 1.75 1.75 1.74 1.74 1.72 1.72 1.72 1.71 1.66 1.65 1.65 1.65 1.65 1.64 1.64 1.64 1.64 1.64 1.63 1.63 1.63 1.63 1.62 1.62 1.62 1.62 1.61 1.21 1.20
1H, CDCl3, 500 MHz
Br
CO2Me
OH
S18
0.96 0.94 3.15 1.07 2.13 1.06 1.13 1.04 3.00 3.15
10.0 9.5 9.0 8.5 8.0 7.5 7.0 6.5 6.0 5.5 5.0 4.5 4.0 3.5 3.0 2.5 2.0 1.5 1.0 0.5 0.0 -0.5
f1 (ppm)

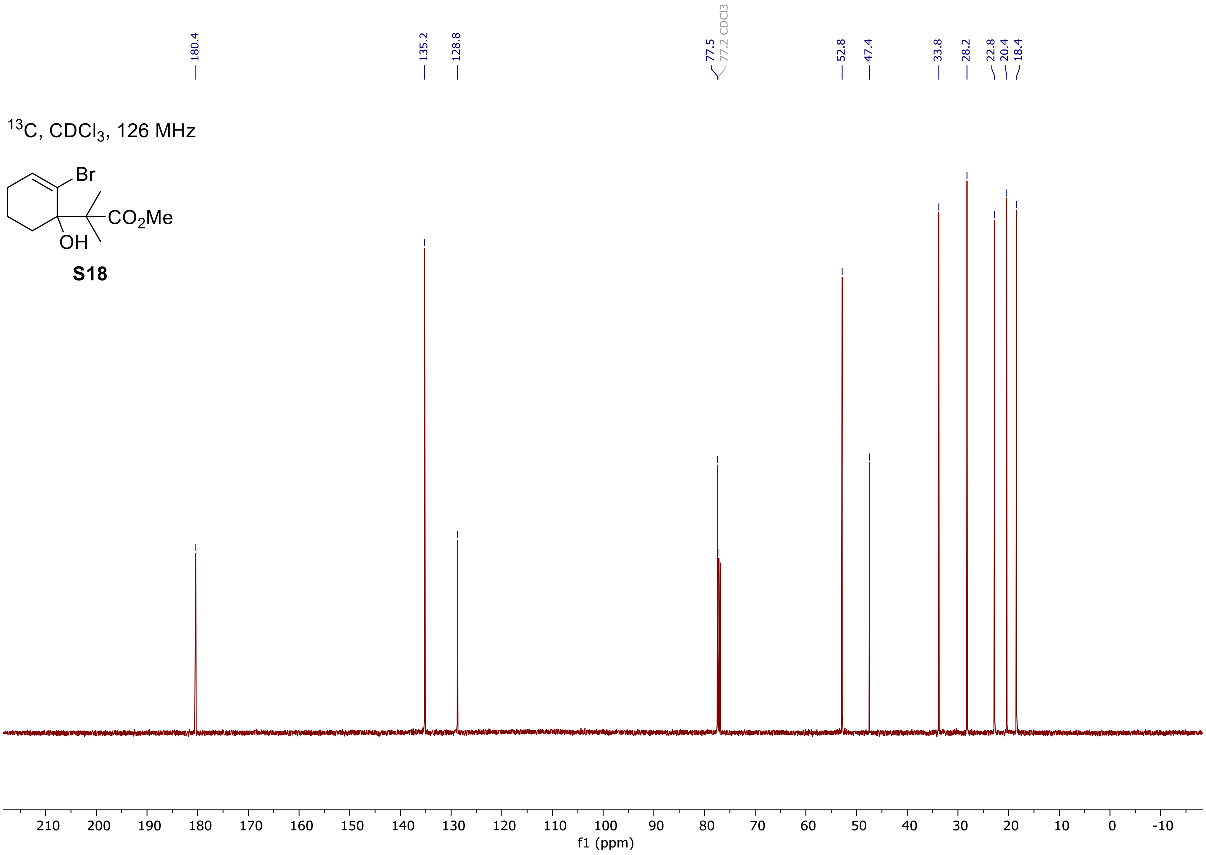
180.4
135.2
128.8
77.5
77.2 CDCl3
52.8
47.4
33.8
28.2
22.8
20.4
18.4
13C, CDCl3, 126 MHz
Br
CO2Me
OH
S18
210 200 190 180 170 160 150 140 130 120 110 100 90 80 70 60 50 40 30 20 10 0 -10
f1 (ppm)

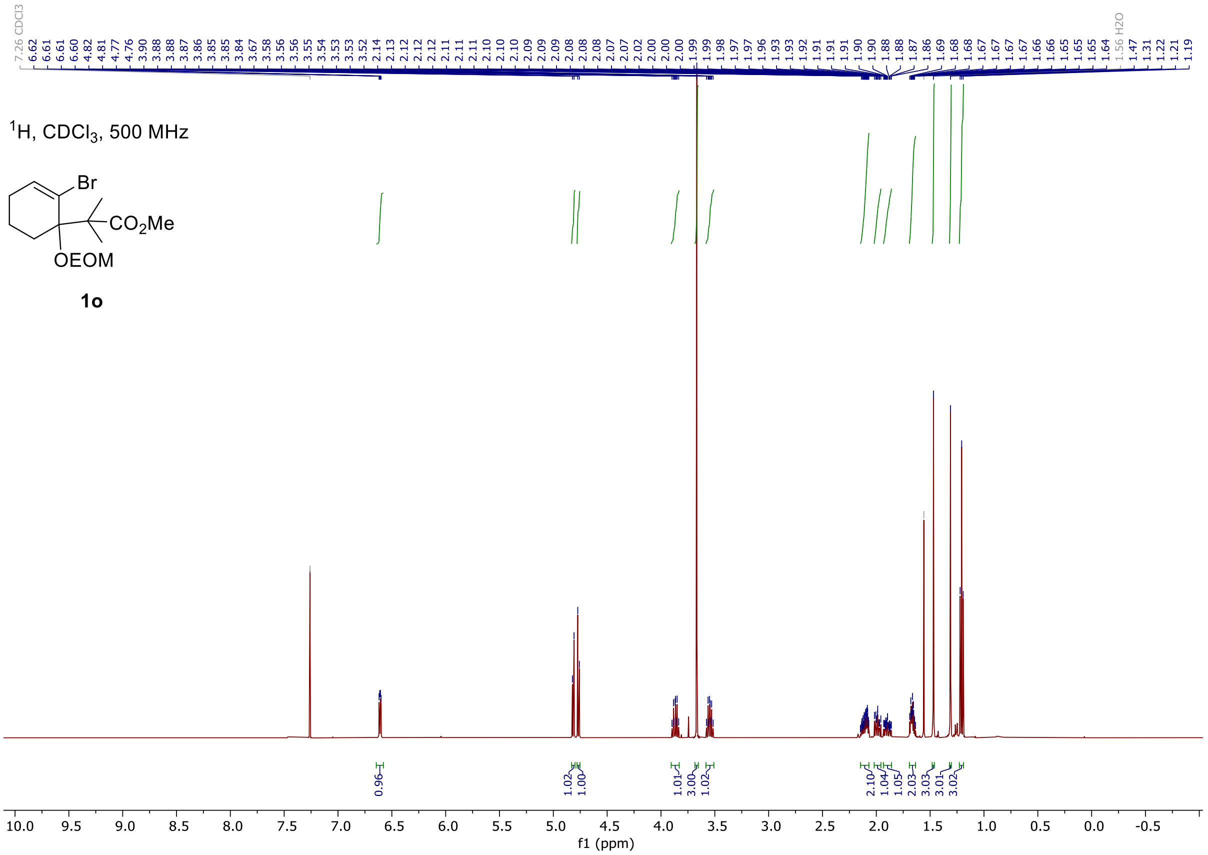
7.26 CDCl3
6.62
6.61
6.61
6.60
4.82
4.81
4.77
4.76
3.90
3.88
3.88
3.87
3.86
3.85
3.85
3.84
3.67
3.58
3.56
3.56
3.55
3.54
3.53
3.53
3.52
2.14
2.13
2.12
2.12
2.12
2.11
2.11
2.11
2.10
2.10
2.10
2.09
2.09
2.09
2.08
2.08
2.08
2.07
2.07
2.02
2.00
2.00
1.99
1.99
1.98
1.97
1.97
1.96
1.93
1.93
1.92
1.91
1.91
1.91
1.90
1.90
1.88
1.88
1.87
1.86
1.69
1.68
1.68
1.67
1.67
1.67
1.67
1.66
1.66
1.65
1.65
1.65
1.64
1.56 H2O
1.47
1.31
1.22
1.21
1.19
^{1}H, CDCl$_3$, 500 MHz
Br
CO$_2$Me
OEOM
1o
0.96
1.02
1.00
1.01
3.00
1.02
2.10
1.04
1.05
2.03
3.03
3.01
3.02
10.0 9.5 9.0 8.5 8.0 7.5 7.0 6.5 6.0 5.5 5.0 4.5 4.0 3.5 3.0 2.5 2.0 1.5 1.0 0.5 0.0 -0.5
f1 (ppm)

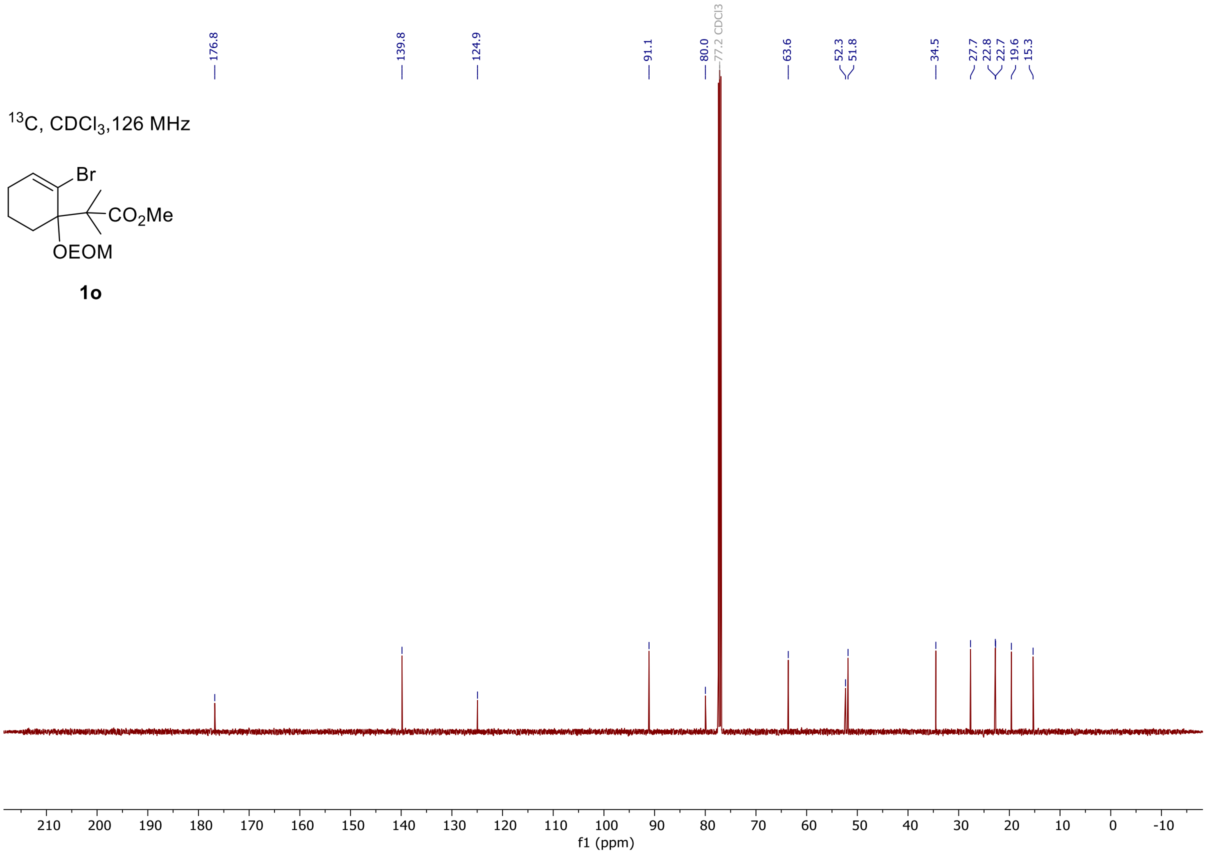
176.8
139.8
124.9
91.1
80.0
77.2 CDCl3
63.6
52.3
51.8
34.5
27.7
22.8
22.7
19.6
15.3
^{13}C, CDCl$_3$,126 MHz
Br
CO$_2$Me
OEOM
1o
210 200 190 180 170 160 150 140 130 120 110 100 90 80 70 60 50 40 30 20 10 0 -10
f1 (ppm)

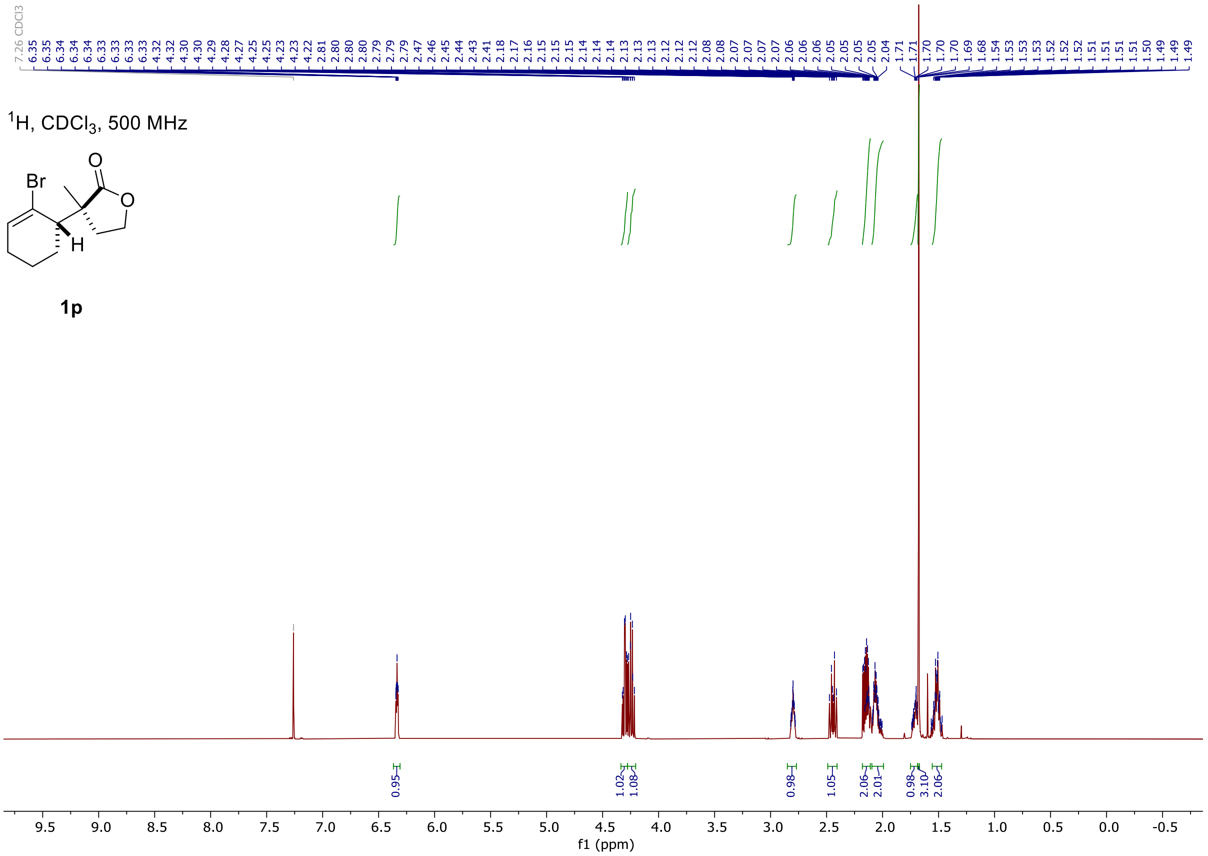

7.26 CDCl3
6.35 6.35 6.34 6.34 6.34 6.33 6.33 6.33 6.33 4.32 4.30 4.30 4.29 4.28 4.27 4.25 4.25 4.23 4.23 4.22 2.81 2.80 2.80 2.80 2.79 2.79 2.47 2.46 2.45 2.44 2.43 2.41 2.18 2.17 2.16 2.15 2.15 2.15 2.14 2.14 2.13 2.13 2.13 2.12 2.12 2.12 2.08 2.08 2.07 2.07 2.07 2.06 2.06 2.06 2.05 2.05 2.05 2.05 2.04 1.71 1.71 1.70 1.70 1.69 1.68 1.54 1.53 1.53 1.52 1.52 1.52 1.51 1.51 1.51 1.50 1.49 1.49
^{1}H, CDCl$_3$, 500 MHz
Br
O
O
H
1p
0.95
1.02
1.08
0.98
1.05
2.06
2.01
0.98
3.10
2.06
9.5 9.0 8.5 8.0 7.5 7.0 6.5 6.0 5.5 5.0 4.5 4.0 3.5 3.0 2.5 2.0 1.5 1.0 0.5 0.0 -0.5
f1 (ppm)

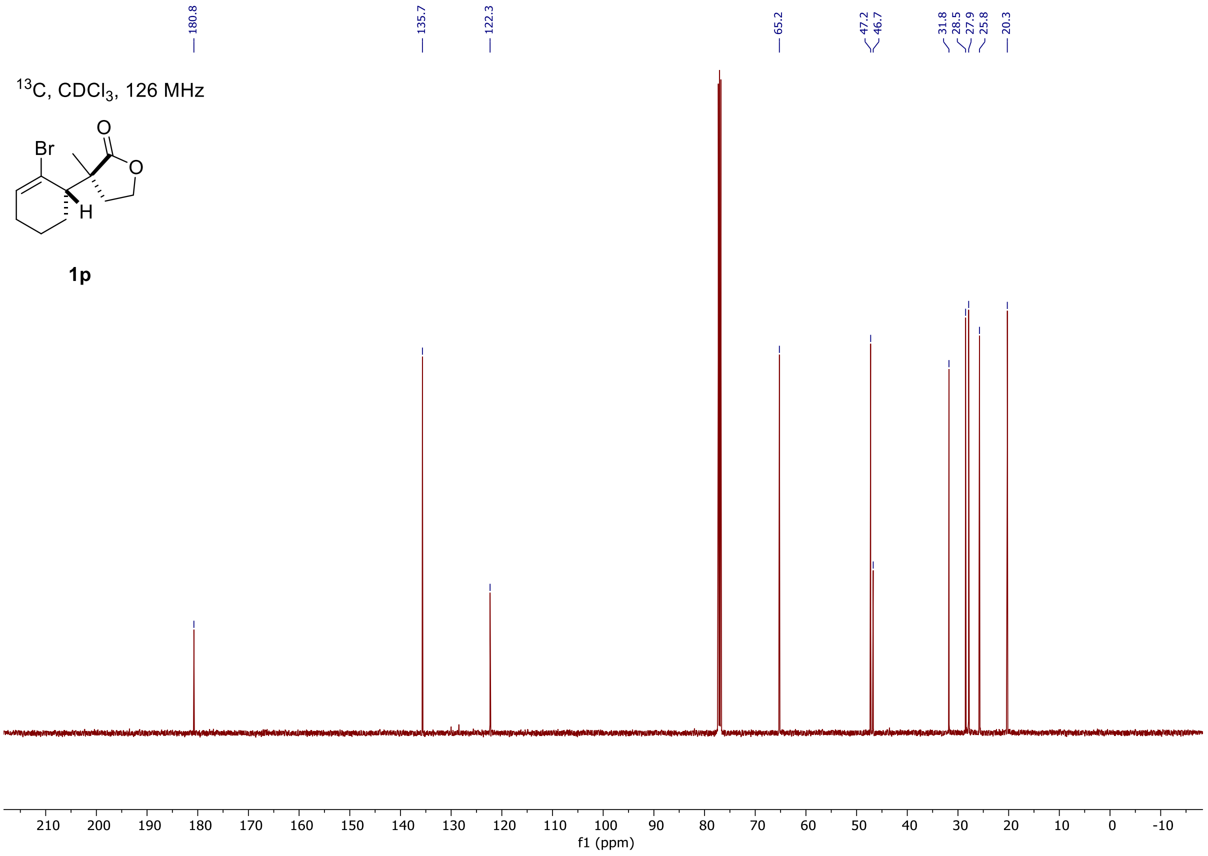

180.8
135.7
122.3
65.2
47.2
46.7
31.8
28.5
27.9
25.8
20.3
^{13}C, CDCl$_3$, 126 MHz
Br
O
O
H
1p
210 200 190 180 170 160 150 140 130 120 110 100 90 80 70 60 50 40 30 20 10 0 -10
f1 (ppm)

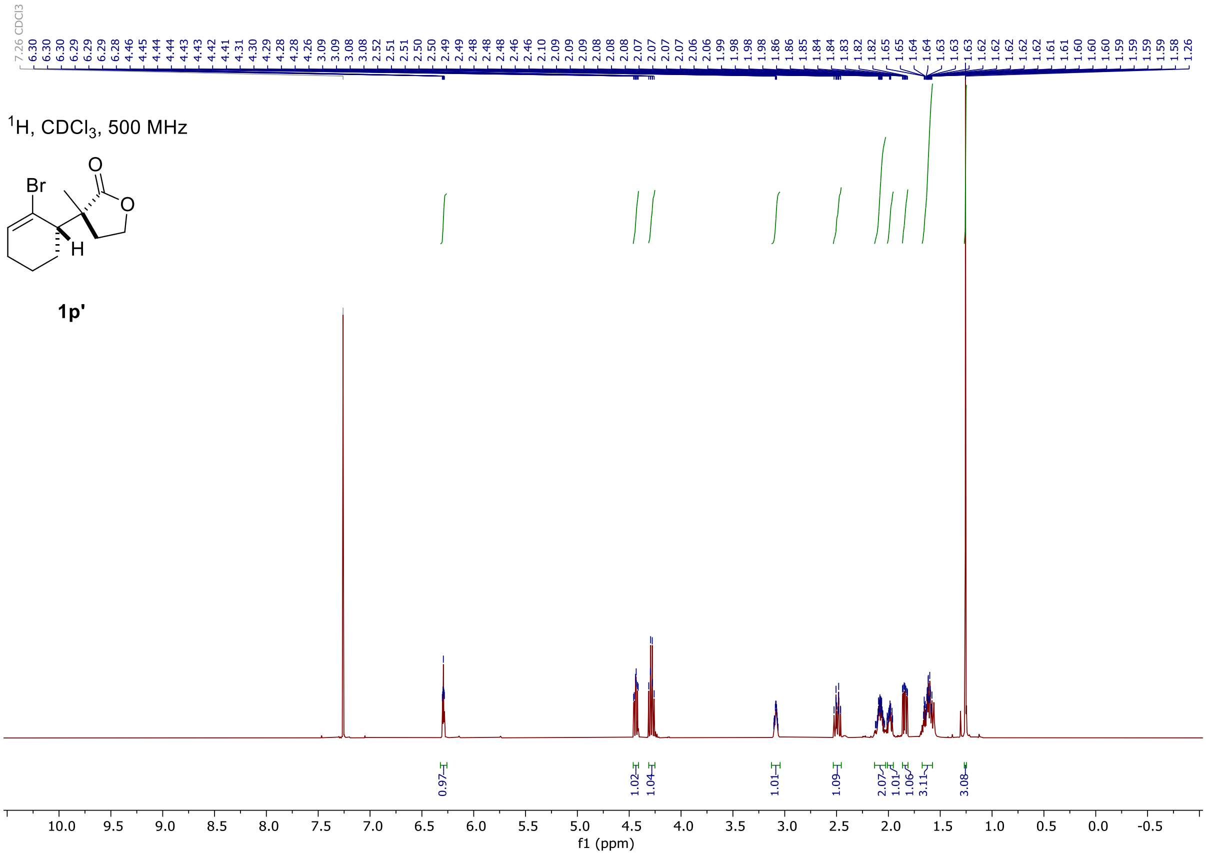

^{1}H, CDCl$_3$, 500 MHz
Br
O
O
H
1p'
0.97
1.02
1.04
1.01
1.09
2.07
1.01
1.06
3.11
3.08
f1 (ppm)

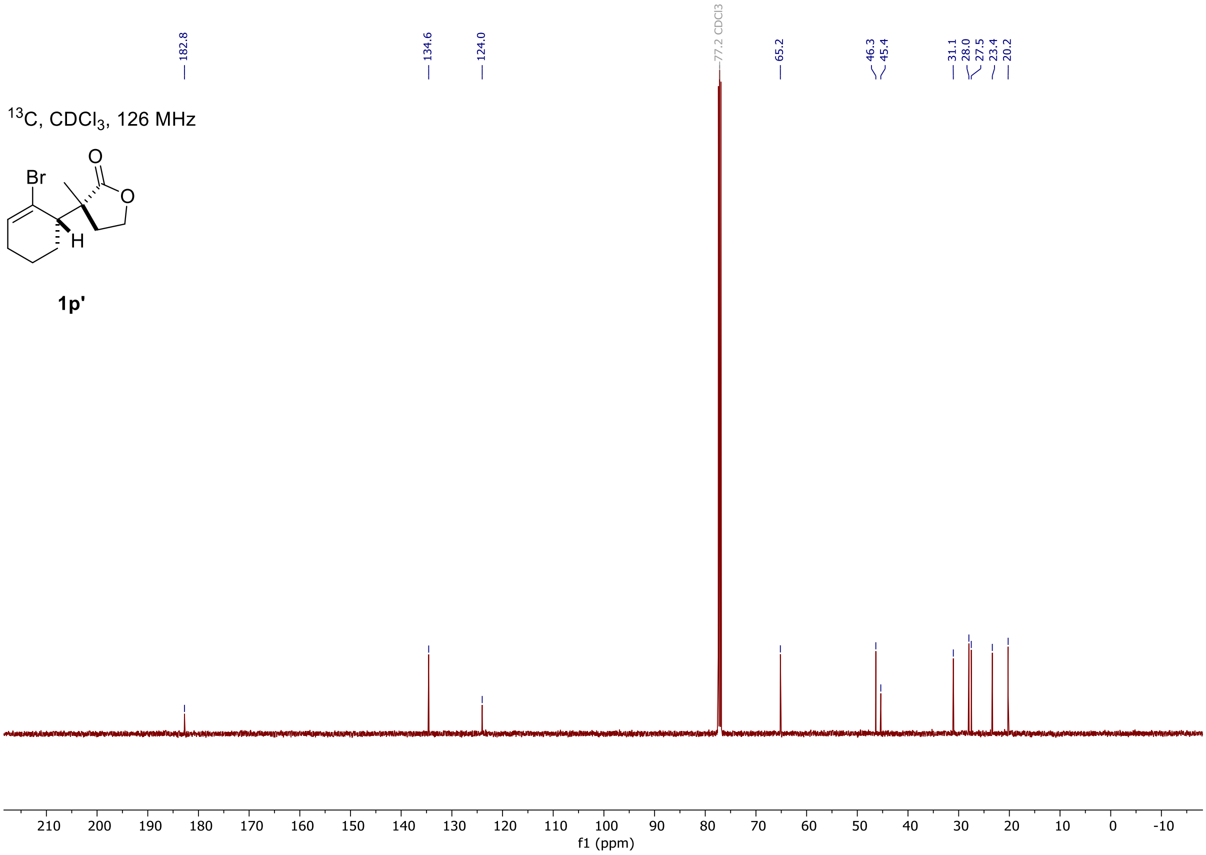

182.8
134.6
124.0
77.2 CDCl3
65.2
46.3
45.4
31.1
28.0
27.5
23.4
20.2
^{13}C, CDCl$_3$, 126 MHz
Br
O
O
H
1p'
f1 (ppm)

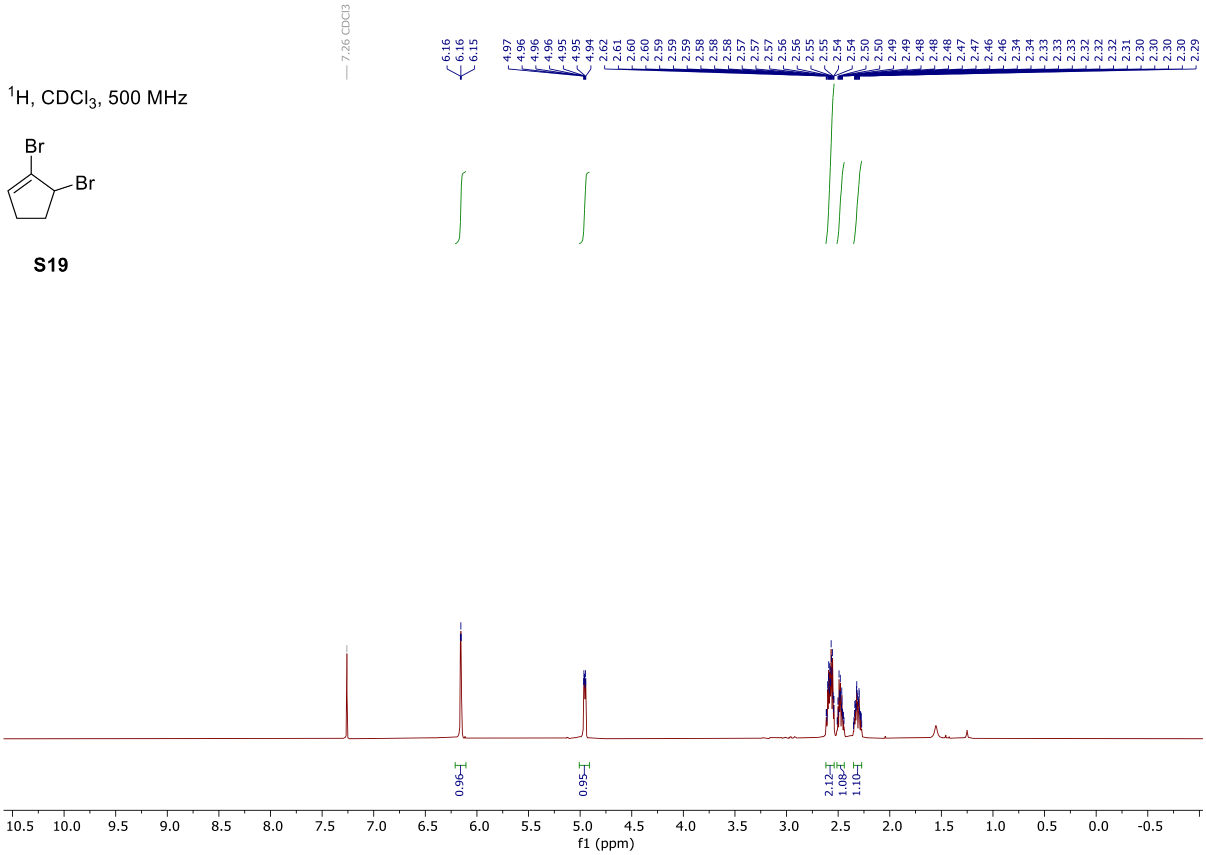

^{1}H, $CDCl_3$, 500 MHz
Br
Br
S19
7.26 CDCl3
6.16
6.16
6.15
4.97
4.96
4.96
4.96
4.95
4.95
4.94
0.96
0.95
2.12
1.08
1.10
f1 (ppm)

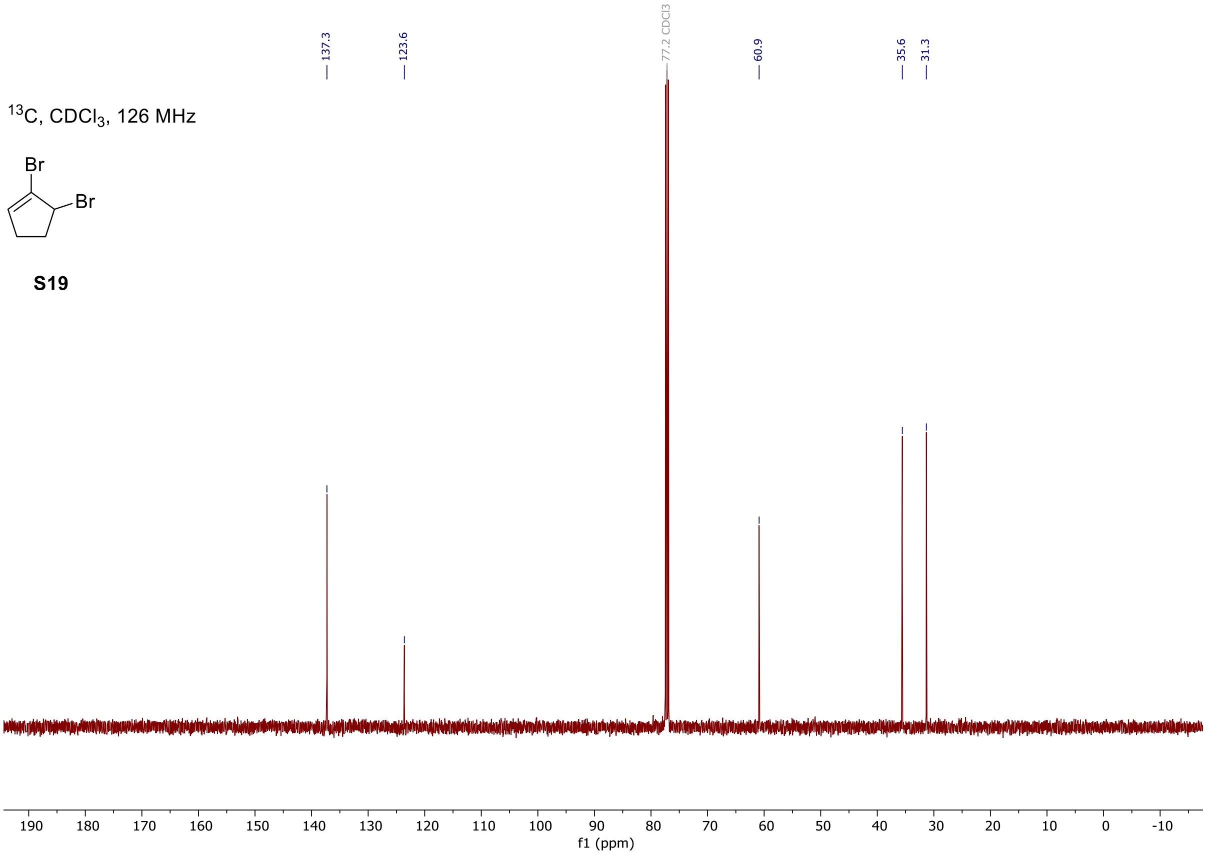

^{13}C, $CDCl_3$, 126 MHz
Br
Br
S19
137.3
123.6
77.2 CDCl3
60.9
35.6
31.3
f1 (ppm)

^1H, $CDCl_3$, 400 MHz

1r

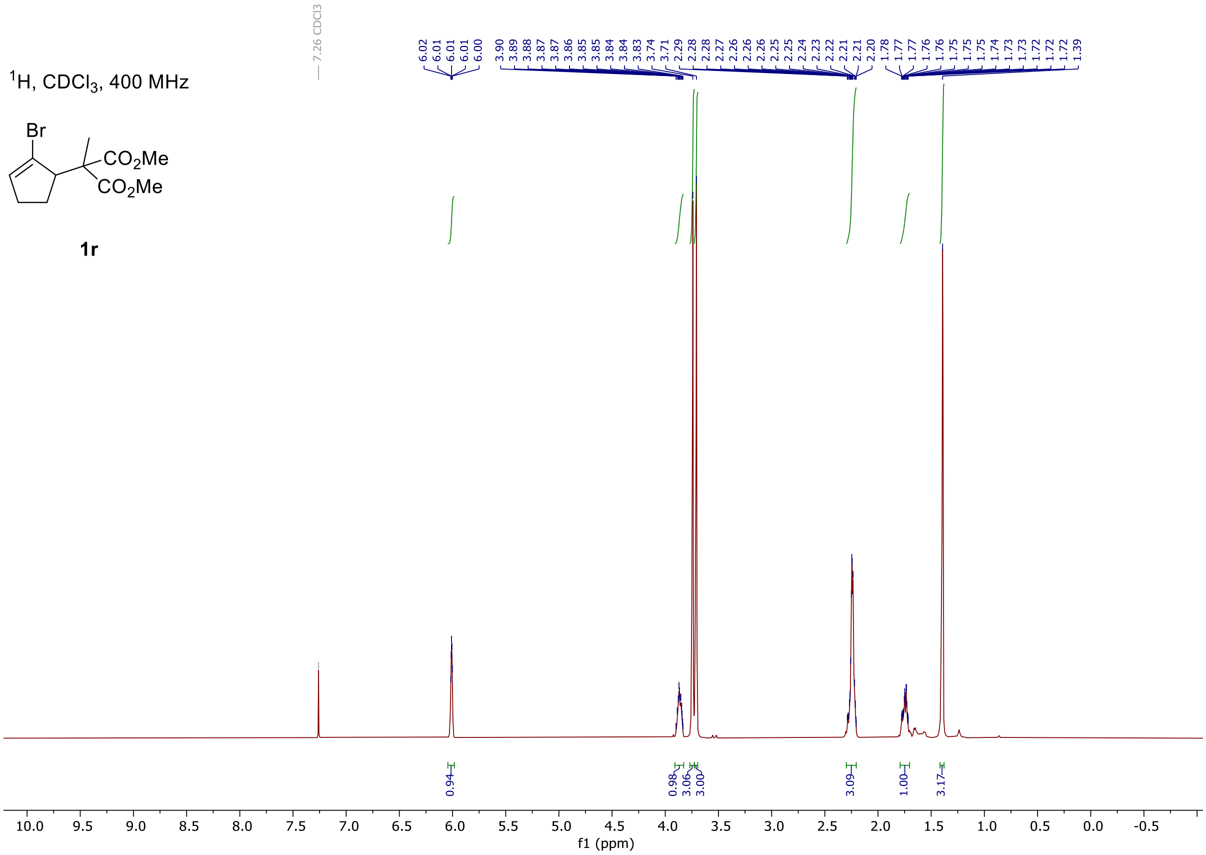

^{13}C, $CDCl_3$, 126 MHz

1r

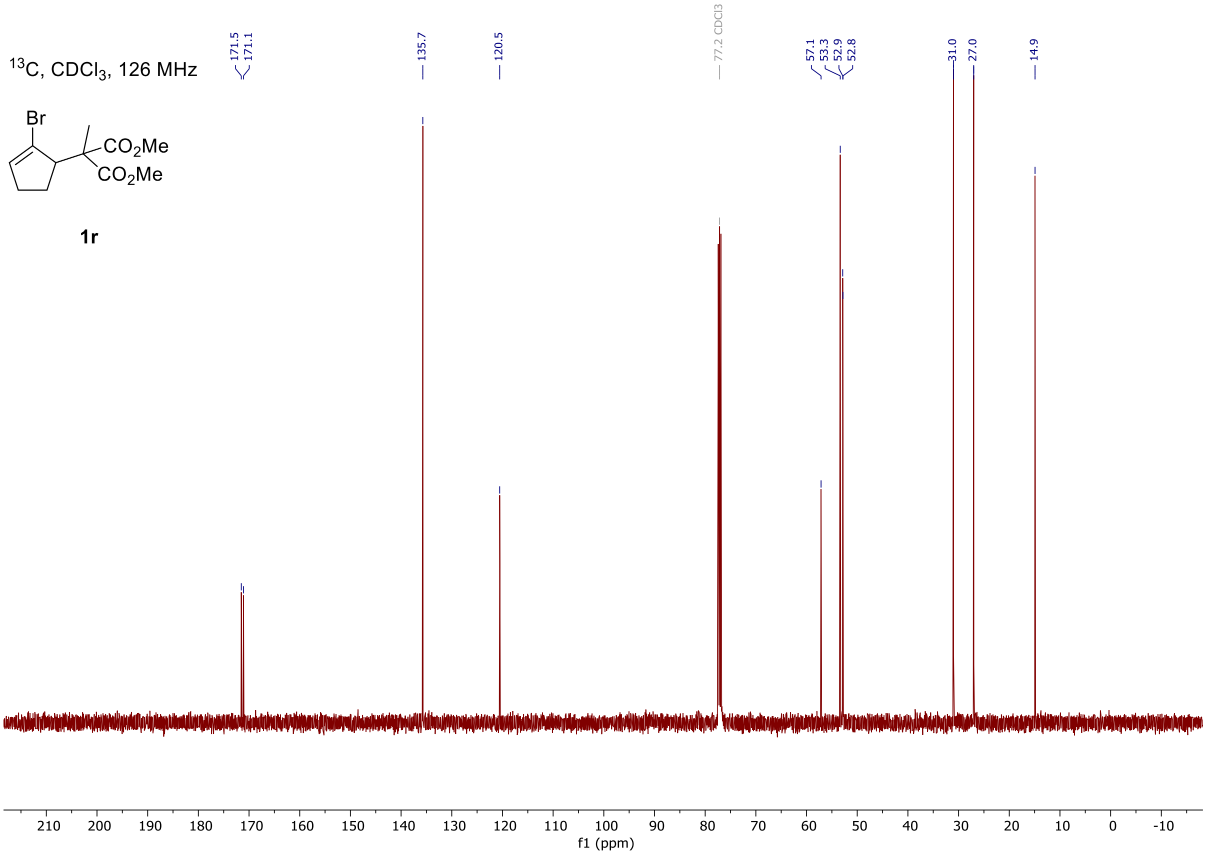

^1H, $CDCl_3$, 500 MHz

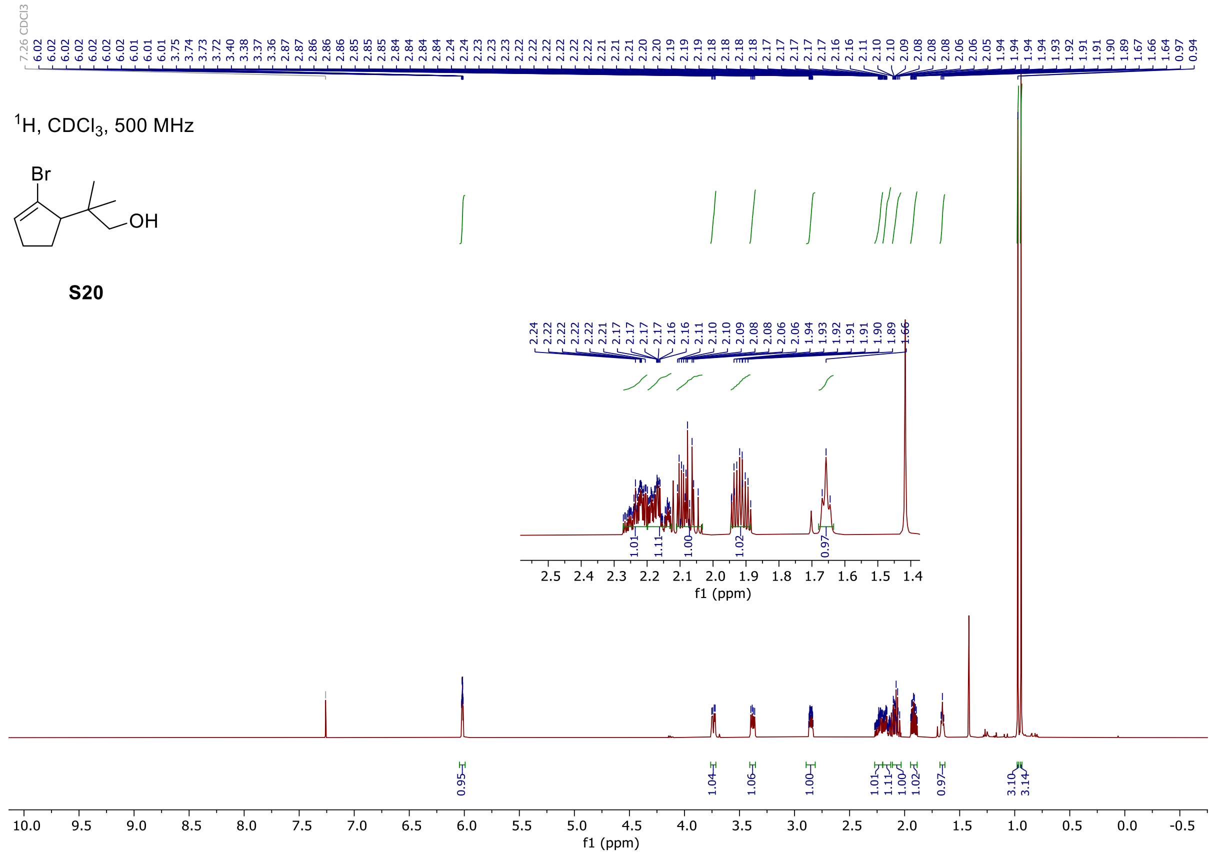

S20

^{13}C, $CDCl_3$, 126 MHz

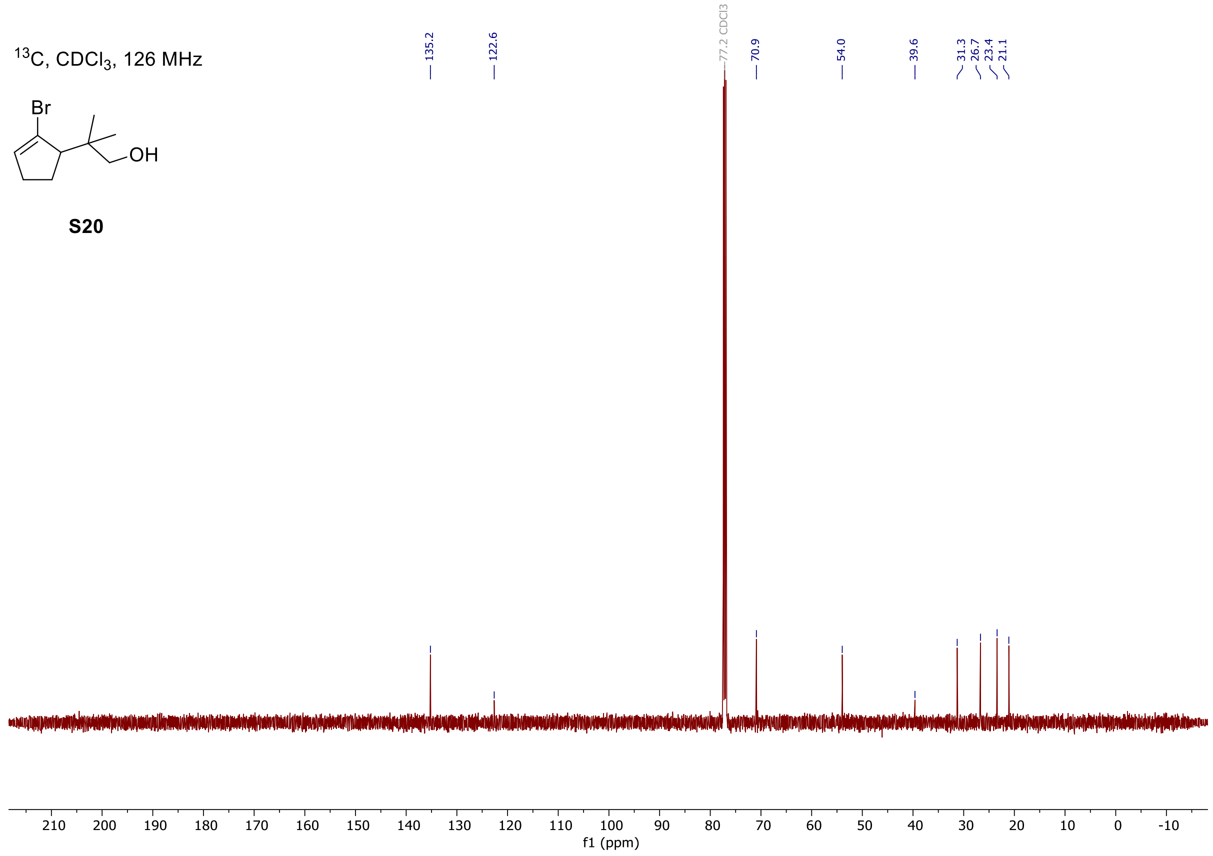

S20

^{1}H, CDCl$_3$, 500 MHz

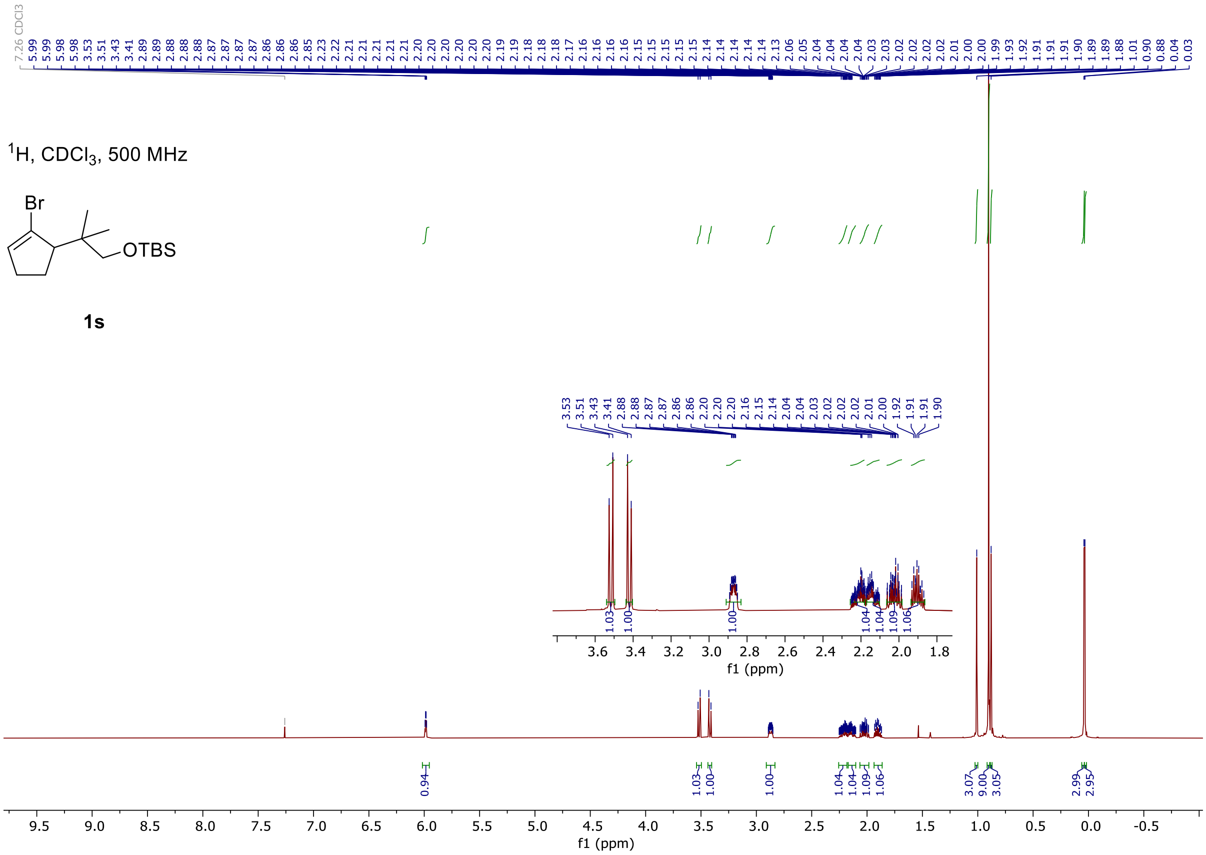

1s

^{13}C, CDCl$_3$, 126 MHz

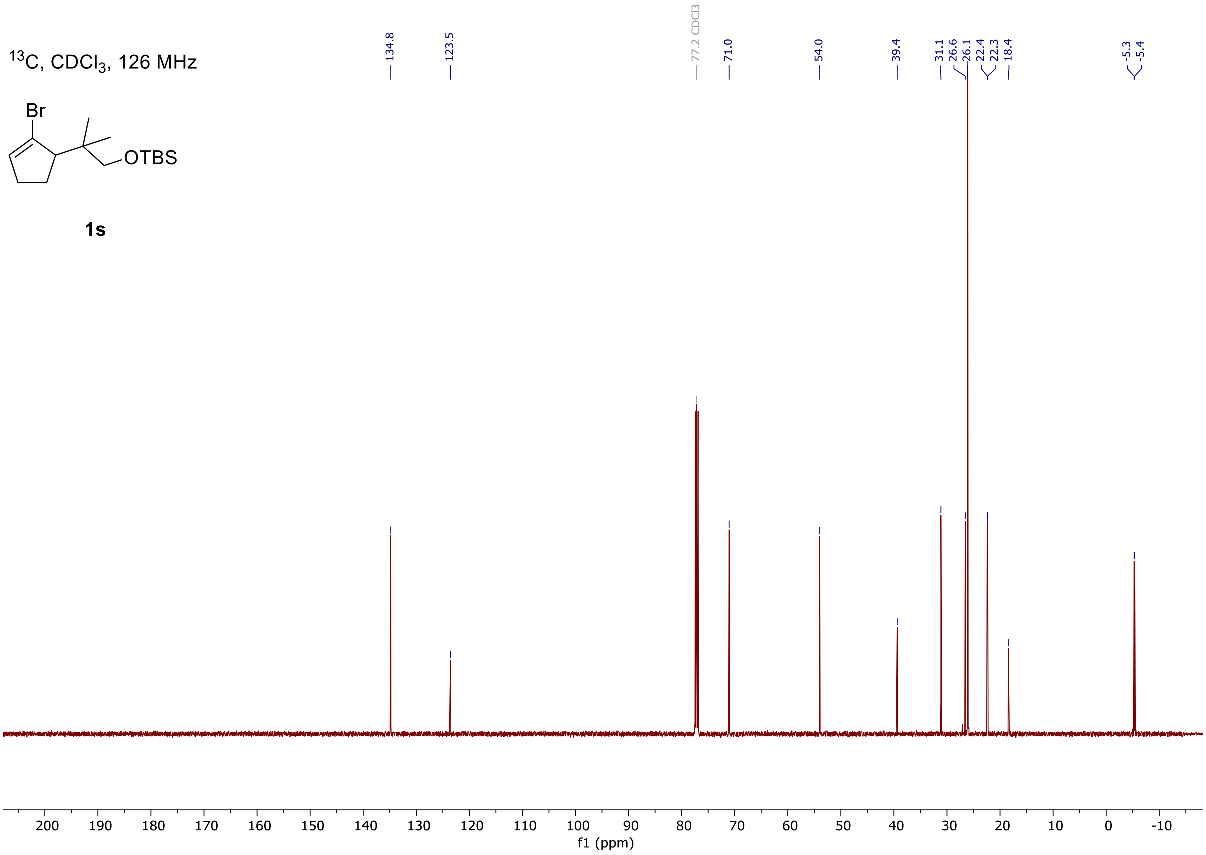

1s

^1H, $CDCl_3$, 500 MHz

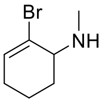

S21-*rac*

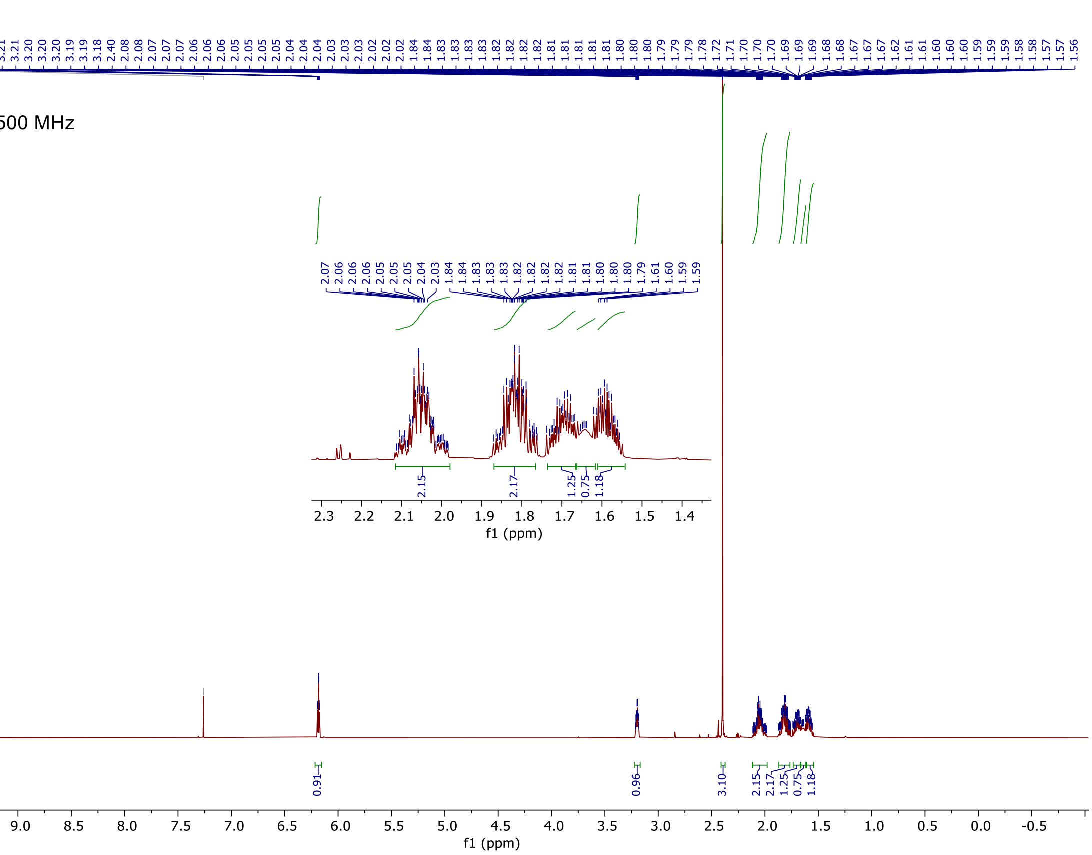

^{13}C, $CDCl_3$, 126 MHz

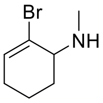

S21-*rac*

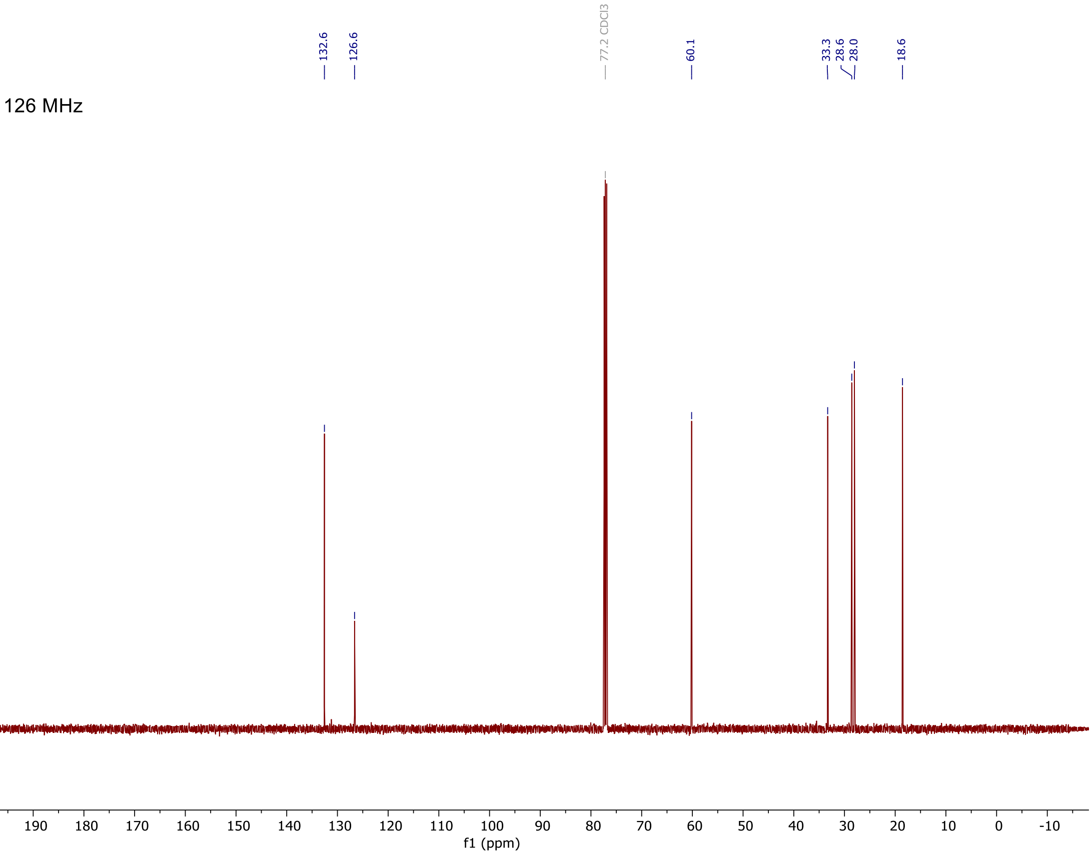

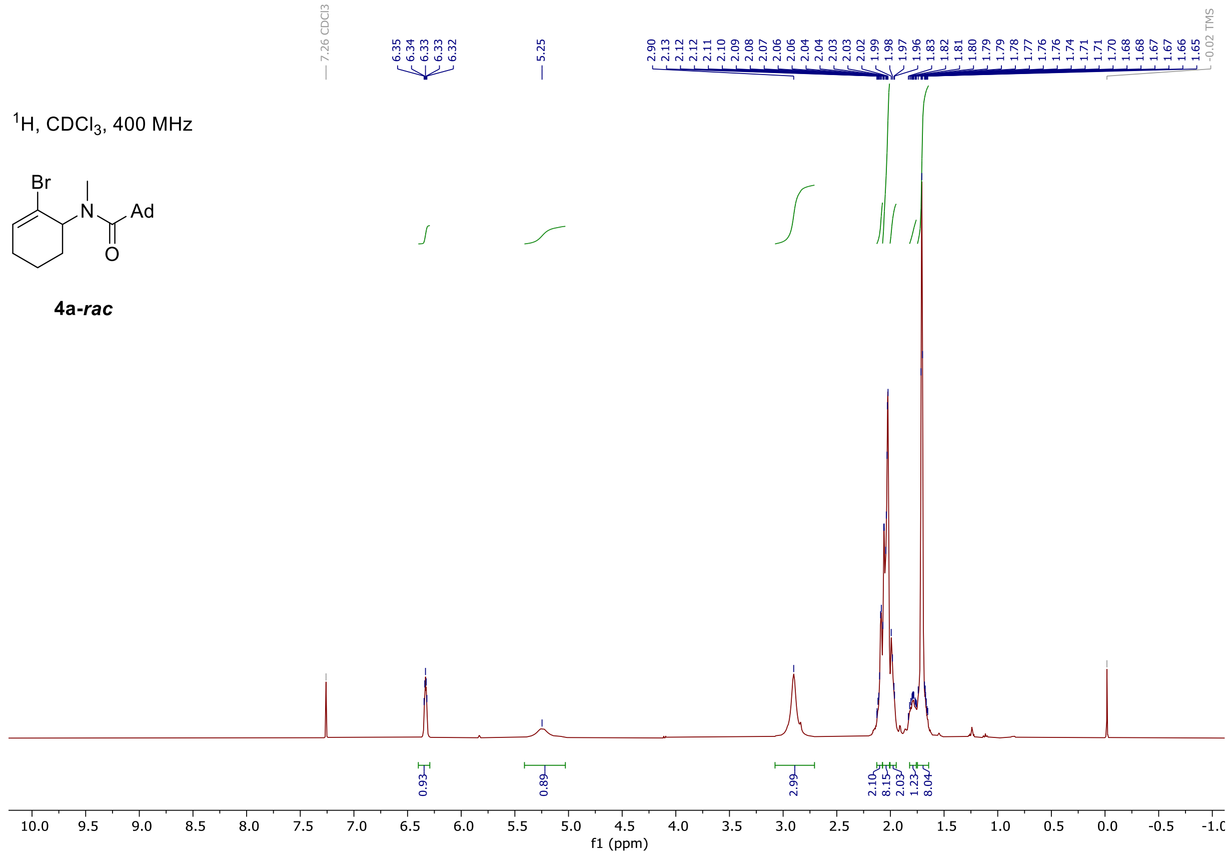

7.26 CDCl3
6.35 6.34 6.33 6.33 6.32
5.25
2.90 2.13 2.12 2.11 2.10 2.09 2.08 2.07 2.06 2.06 2.04 2.04 2.03 2.03 2.02 1.99 1.98 1.97 1.96 1.83 1.82 1.81 1.80 1.79 1.79 1.78 1.77 1.76 1.76 1.74 1.71 1.70 1.68 1.67 1.66 1.65
-0.02 TMS
^{1}H, CDCl$_3$, 400 MHz
Br
N
Ad
O
4a-*rac*
0.93
0.89
2.99
2.10
8.15
2.03
1.23
8.04
f1 (ppm)

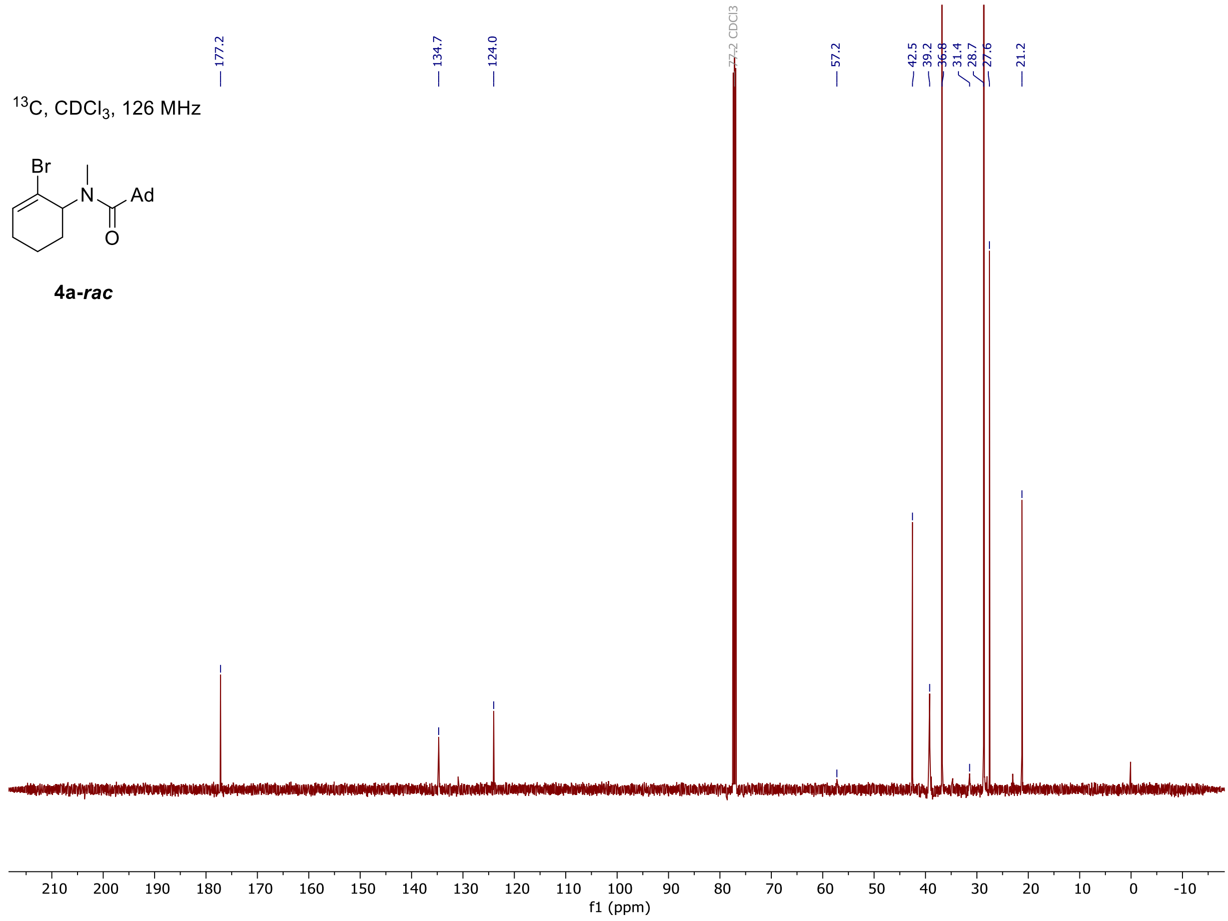

177.2
134.7
124.0
77.2 CDCl3
57.2
42.5
39.2
36.8
31.4
28.7
27.6
21.2
^{13}C, CDCl$_3$, 126 MHz
Br
N
Ad
O
4a-*rac*
f1 (ppm)

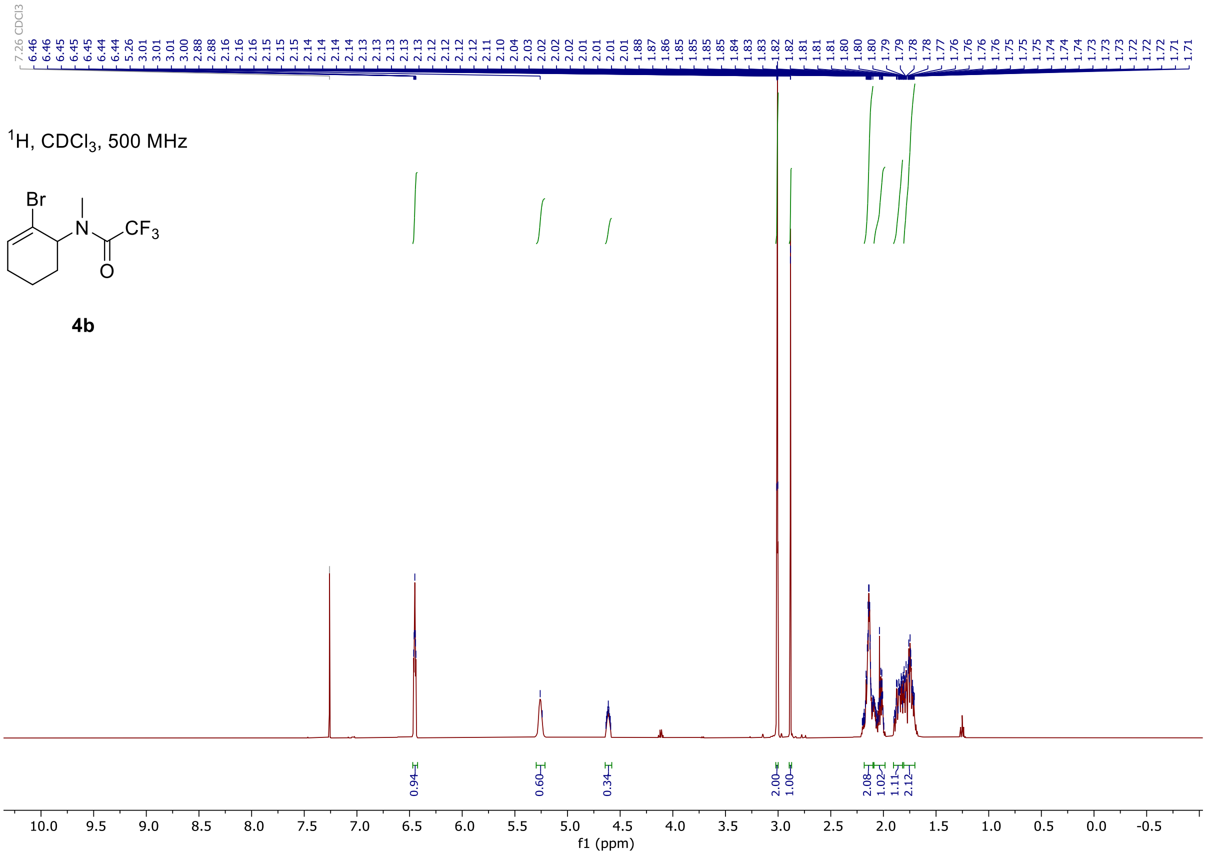
1H, CDCl3, 500 MHz
Br
N
CF3
O
4b
f1 (ppm)

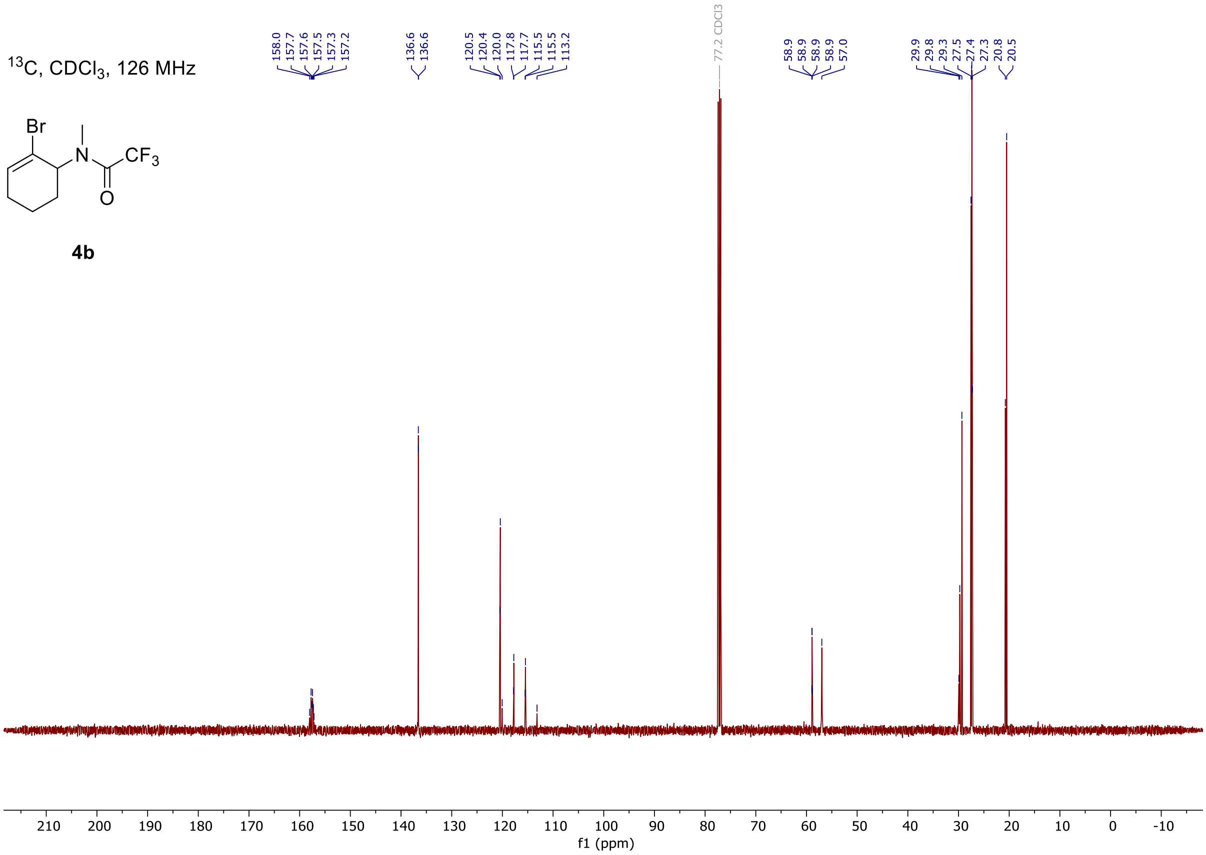
13C, CDCl3, 126 MHz
Br
N
CF3
O
4b
f1 (ppm)

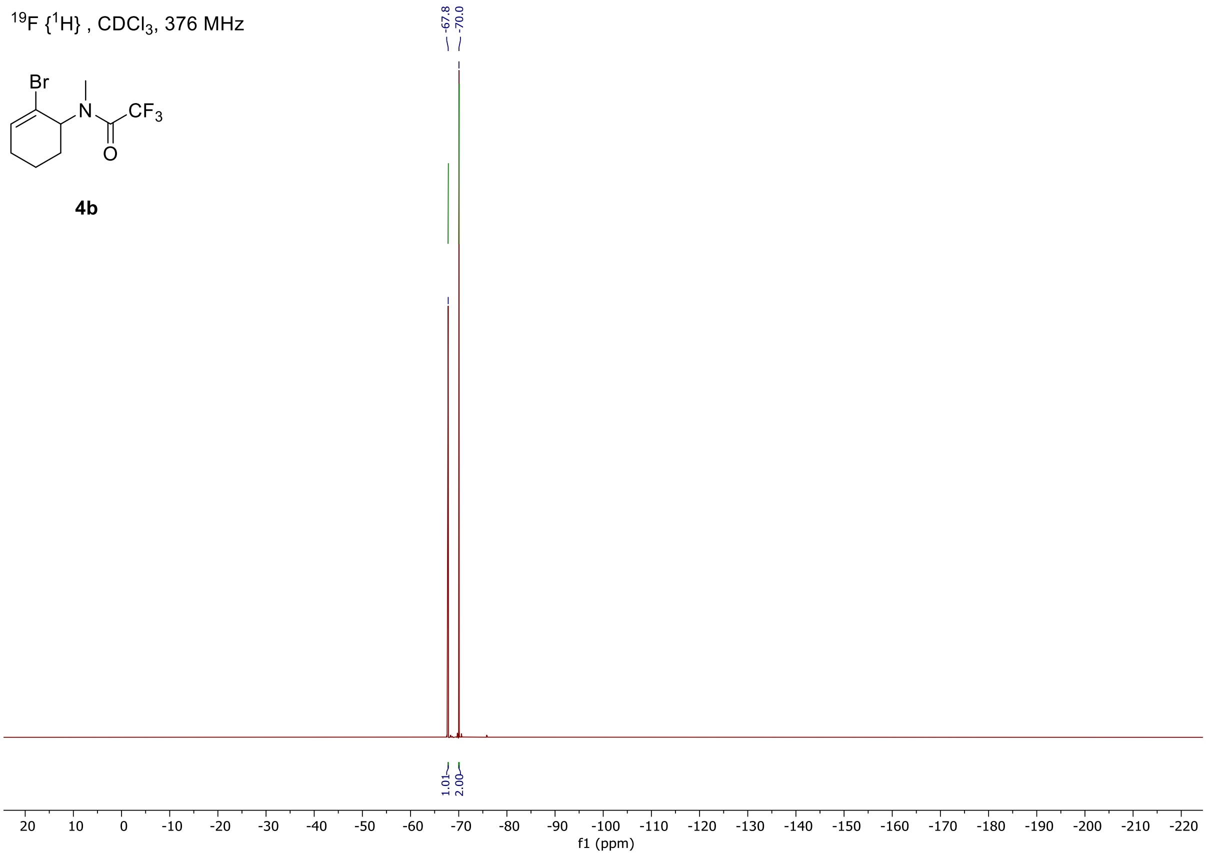

19F {1H} , CDCl3, 376 MHz
Br
N
CF3
O
4b
-67.8
-70.0
1.01
2.00
20 10 0 -10 -20 -30 -40 -50 -60 -70 -80 -90 -100 -110 -120 -130 -140 -150 -160 -170 -180 -190 -200 -210 -220
f1 (ppm)

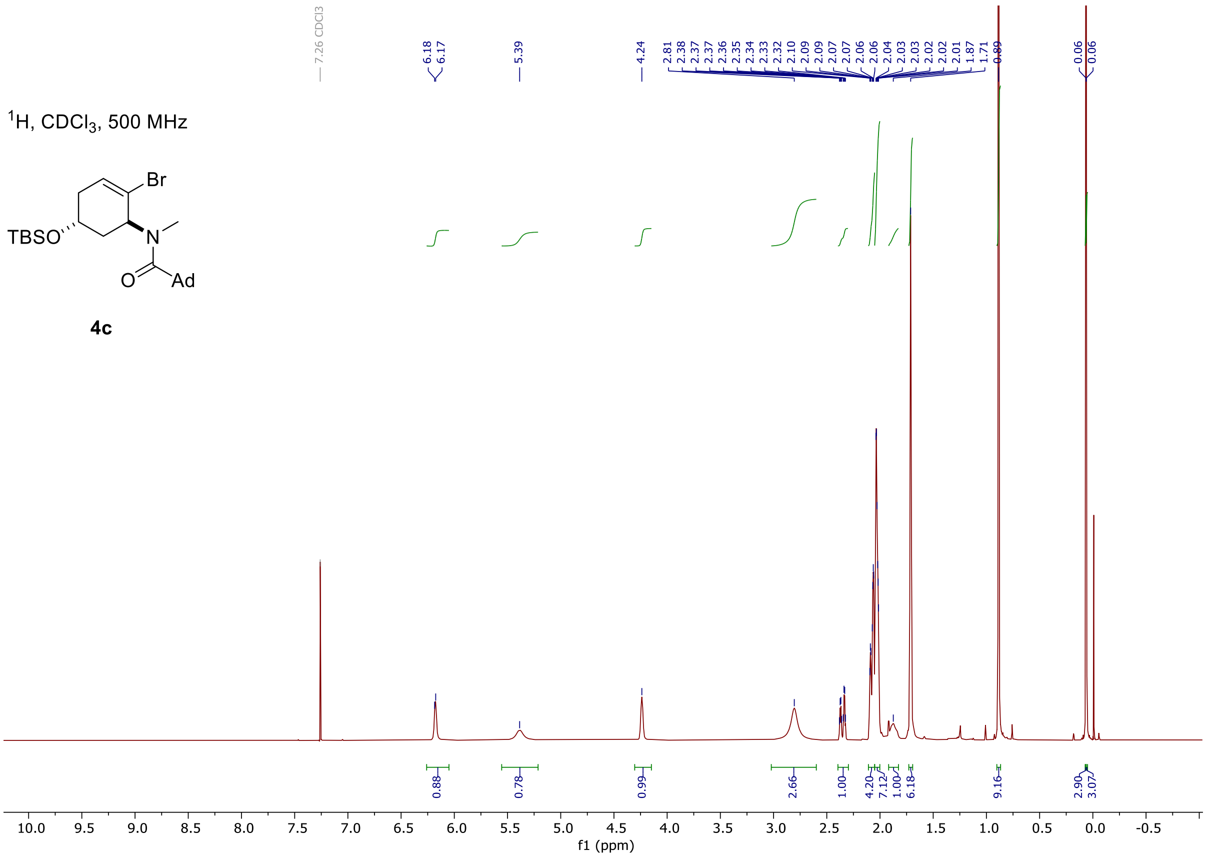

7.26 CDCl3
6.18
6.17
5.39
4.24
2.81 2.38 2.37 2.37 2.36 2.35 2.34 2.33 2.32 2.10 2.09 2.09 2.07 2.07 2.06 2.06 2.04 2.03 2.03 2.02 2.02 2.01 1.87 1.71 0.89
0.06
0.06
^{1}H, CDCl$_3$, 500 MHz
Br
TBSO
N
O
Ad
4c
0.88
0.78
0.99
2.66
1.00
4.20
7.12
1.00
6.18
9.16
2.90
3.07
f1 (ppm)

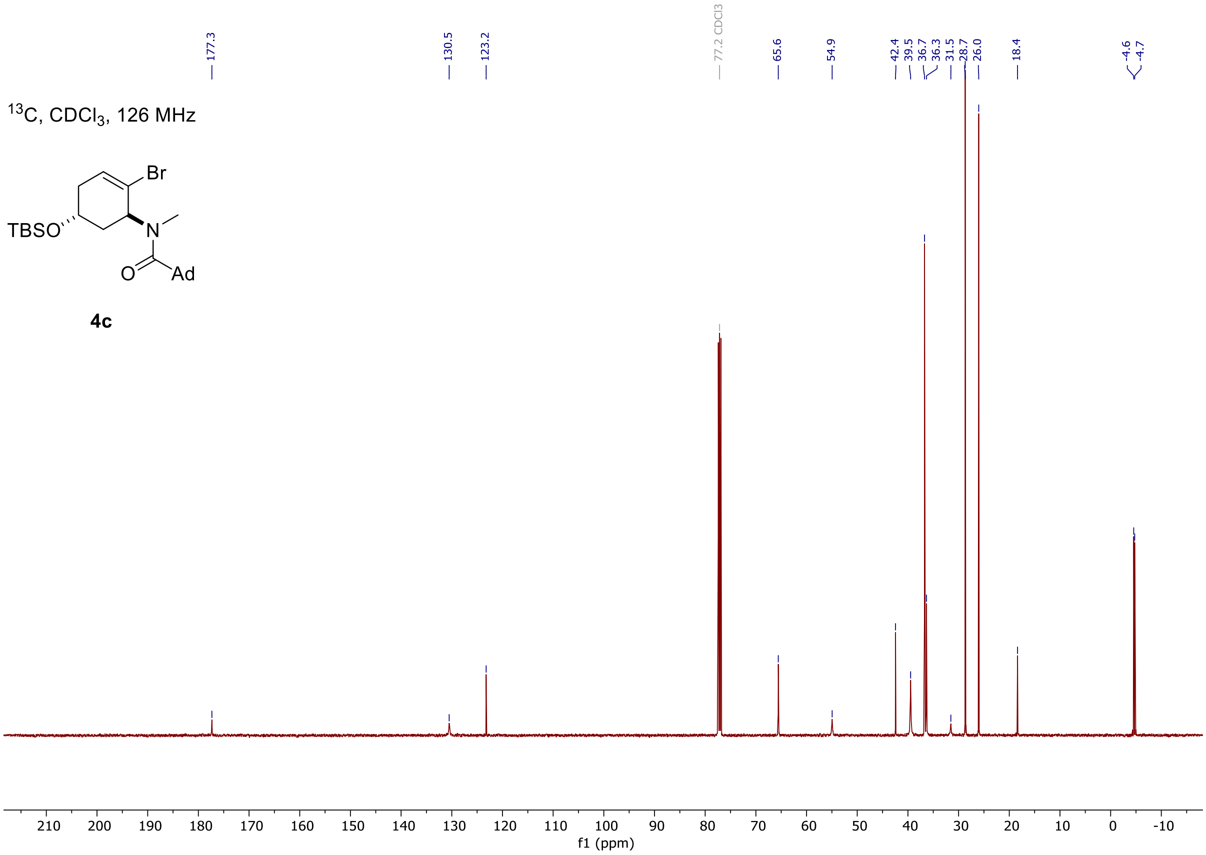

177.3
130.5
123.2
77.2 CDCl3
65.6
54.9
42.4
39.5
36.7
36.3
31.5
28.7
26.0
18.4
-4.6
-4.7
^{13}C, CDCl$_3$, 126 MHz
Br
TBSO
N
O
Ad
4c
f1 (ppm)

NOESY of **4c**.

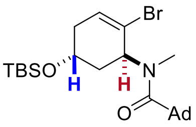

4c

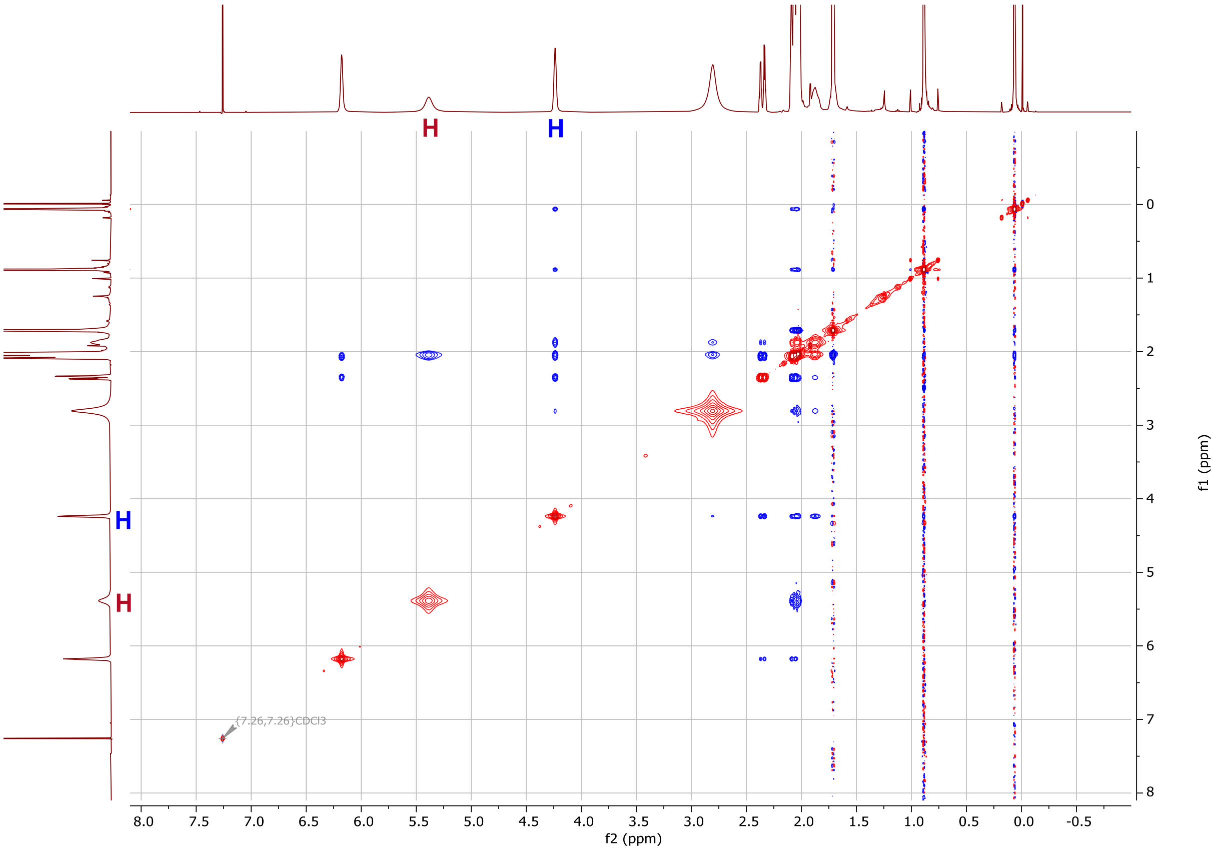

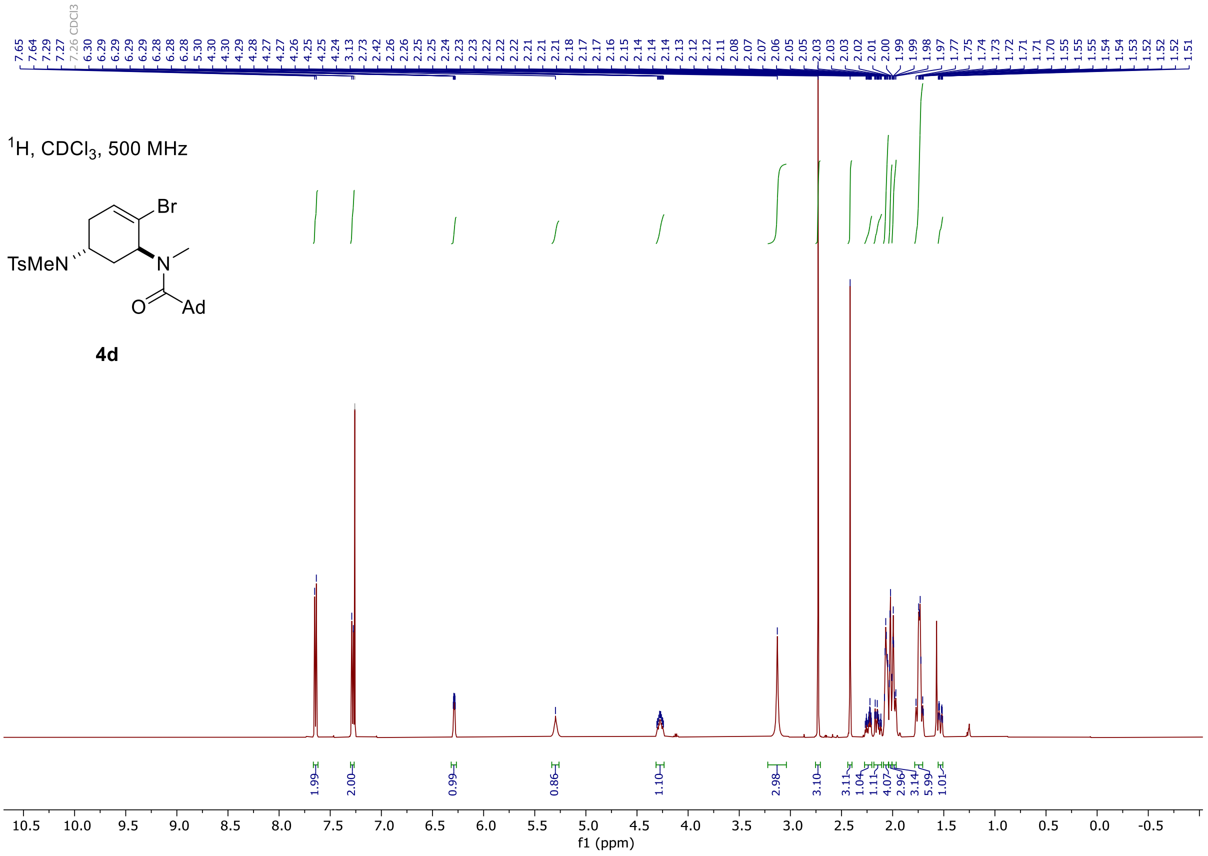
1H, CDCl3, 500 MHz
Br
TsMeN
N
O
Ad
4d
f1 (ppm)

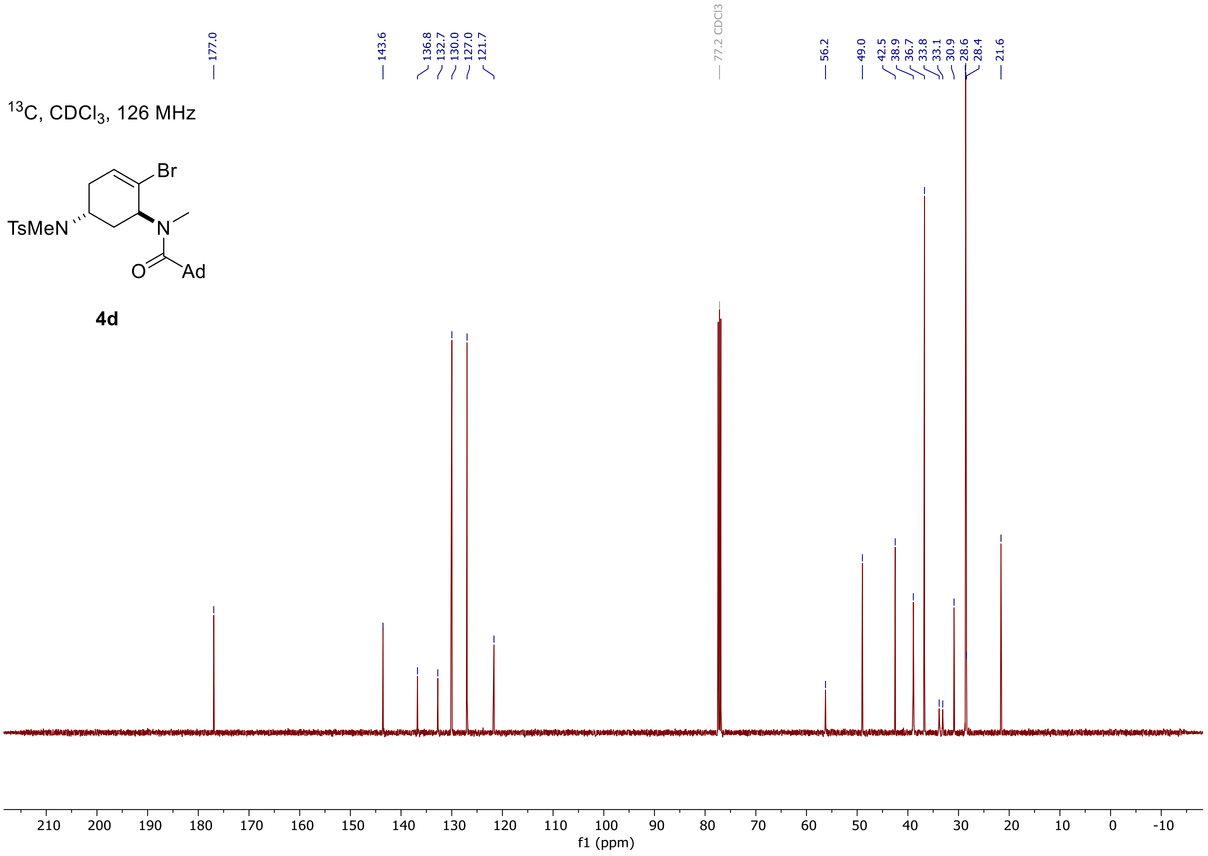
177.0
143.6
136.8
132.7
130.0
127.0
121.7
77.2 CDCl3
56.2
49.0
42.5
38.9
36.7
33.8
33.1
30.9
28.6
28.4
21.6
13C, CDCl3, 126 MHz
Br
TsMeN
N
O
Ad
4d
f1 (ppm)

NOESY of **4d**.

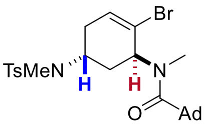

4d

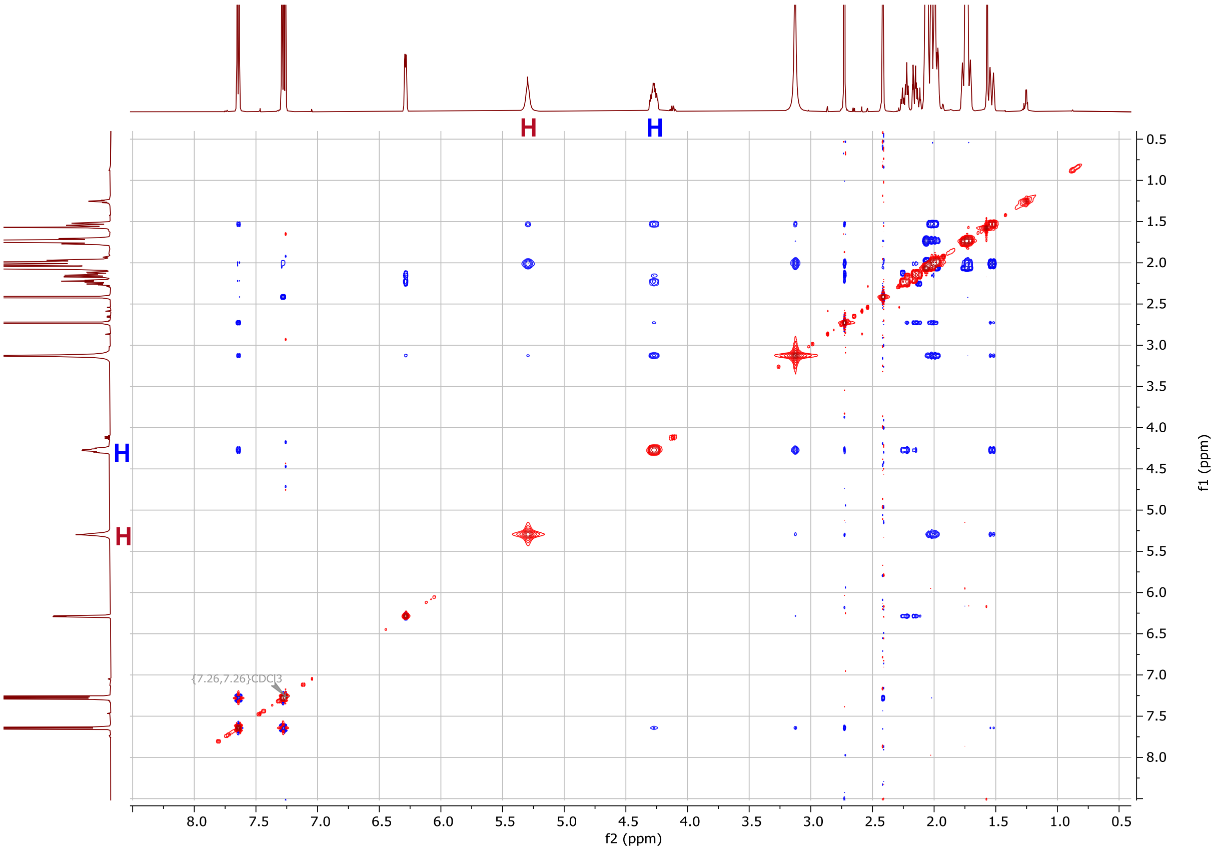

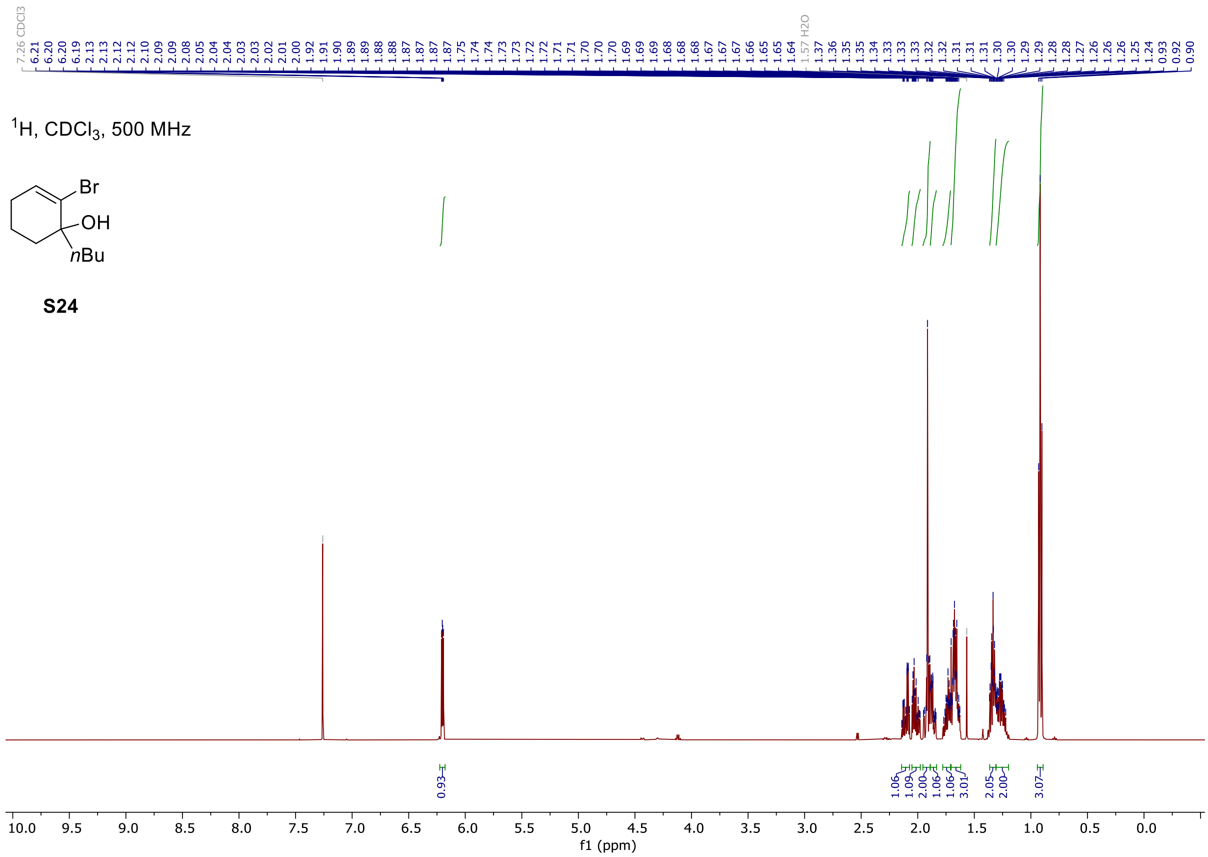
7.26 CDCl3
6.21
6.20
6.20
6.19
2.13
2.13
2.12
2.12
2.10
2.09
2.09
2.08
2.05
2.04
2.04
2.03
2.03
2.02
2.01
2.00
1.92
1.91
1.90
1.89
1.89
1.88
1.88
1.87
1.87
1.87
1.87
1.75
1.74
1.74
1.73
1.73
1.72
1.72
1.71
1.71
1.70
1.70
1.70
1.69
1.69
1.69
1.68
1.68
1.67
1.67
1.67
1.66
1.65
1.65
1.64
1.57 H2O
1.37
1.36
1.35
1.35
1.34
1.33
1.33
1.33
1.32
1.32
1.31
1.31
1.31
1.30
1.30
1.29
1.29
1.28
1.28
1.27
1.26
1.26
1.25
1.24
0.93
0.92
0.90
1H, CDCl3, 500 MHz
Br
OH
nBu
S24
0.93
1.06
1.09
2.00
1.06
1.06
3.01
2.05
2.00
3.07
10.0 9.5 9.0 8.5 8.0 7.5 7.0 6.5 6.0 5.5 5.0 4.5 4.0 3.5 3.0 2.5 2.0 1.5 1.0 0.5 0.0
f1 (ppm)

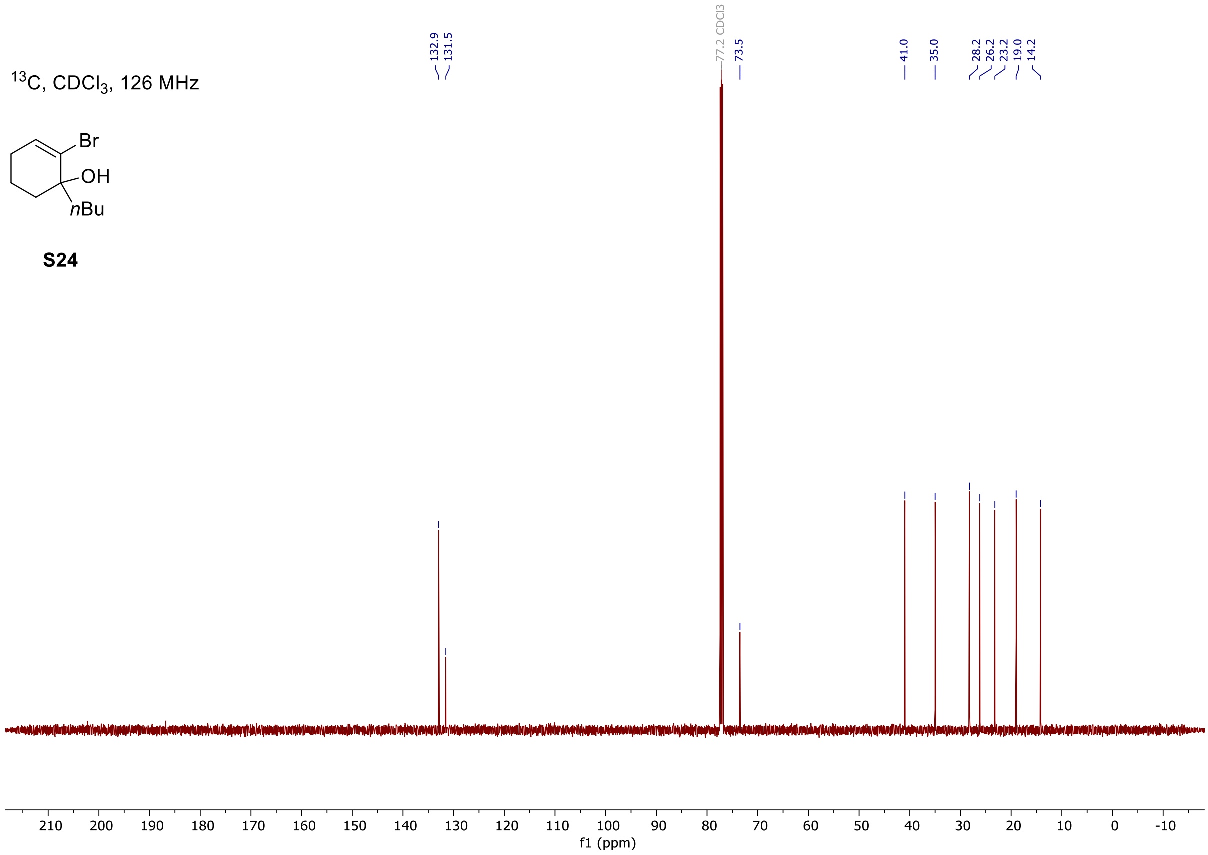
132.9
131.5
77.2 CDCl3
73.5
41.0
35.0
28.2
26.2
23.2
19.0
14.2
13C, CDCl3, 126 MHz
Br
OH
nBu
S24
210 200 190 180 170 160 150 140 130 120 110 100 90 80 70 60 50 40 30 20 10 0 -10
f1 (ppm)

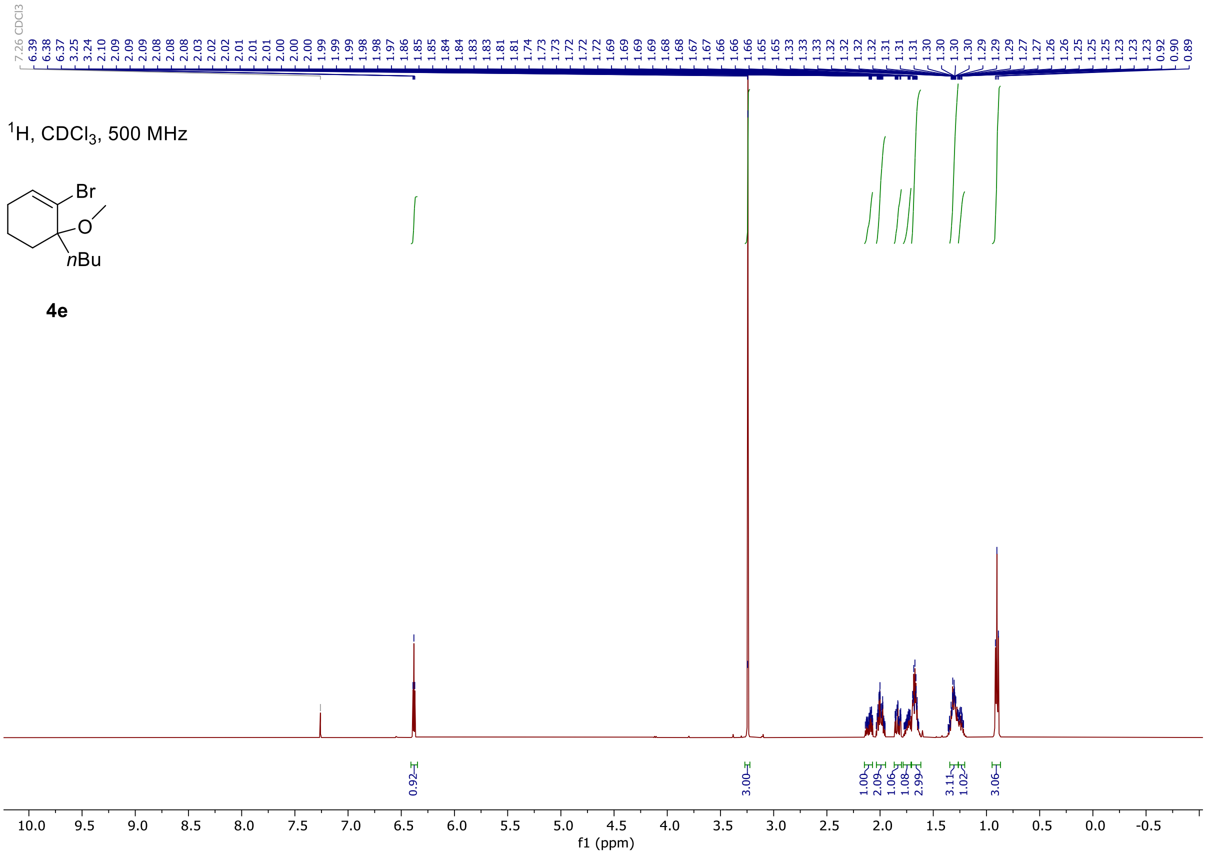
1H, CDCl3, 500 MHz
Br
O
nBu
4e
0.92
3.00
1.00
2.09
1.06
1.08
2.99
3.11
1.02
3.06
f1 (ppm)

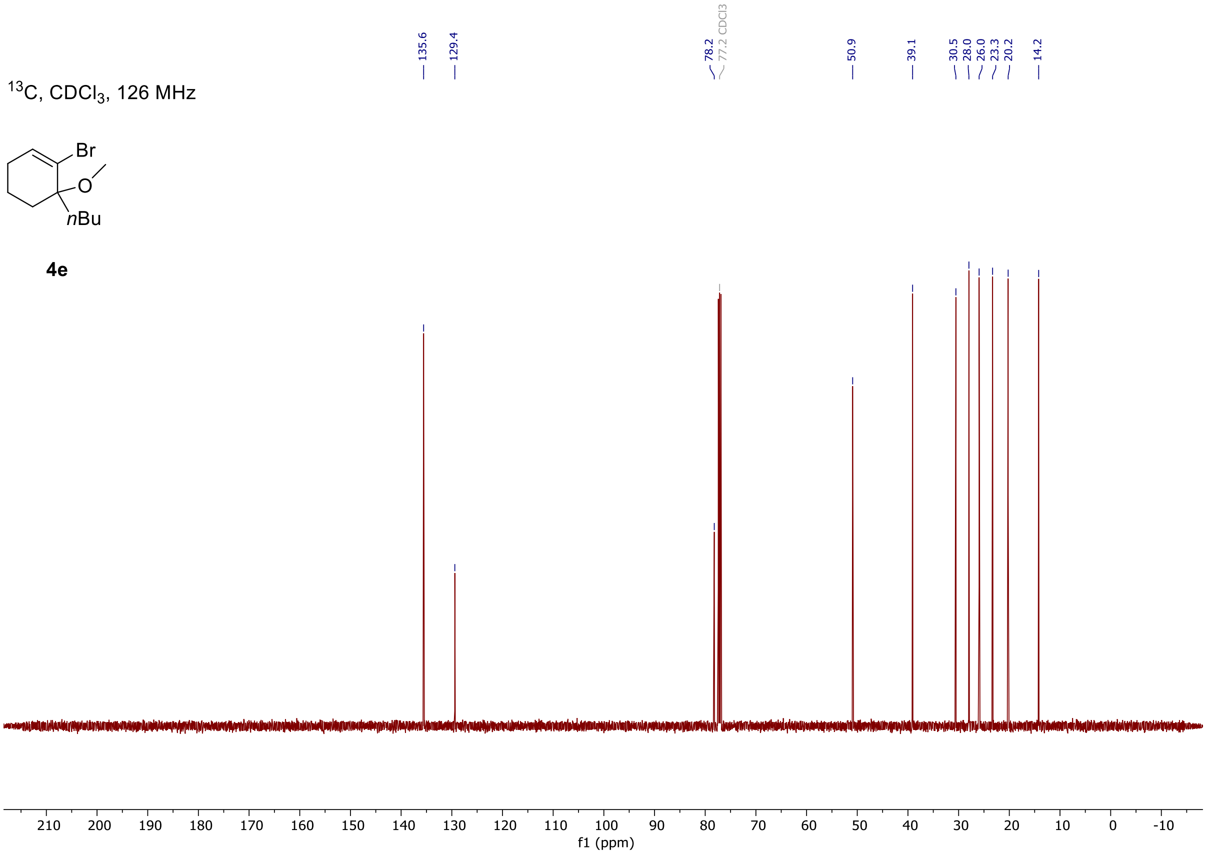
135.6
129.4
78.2
77.2 CDCl3
50.9
39.1
30.5
28.0
26.0
23.3
20.2
14.2
13C, CDCl3, 126 MHz
Br
O
nBu
4e
f1 (ppm)

^{1}H, $CDCl_3$, 500 MHz

S25

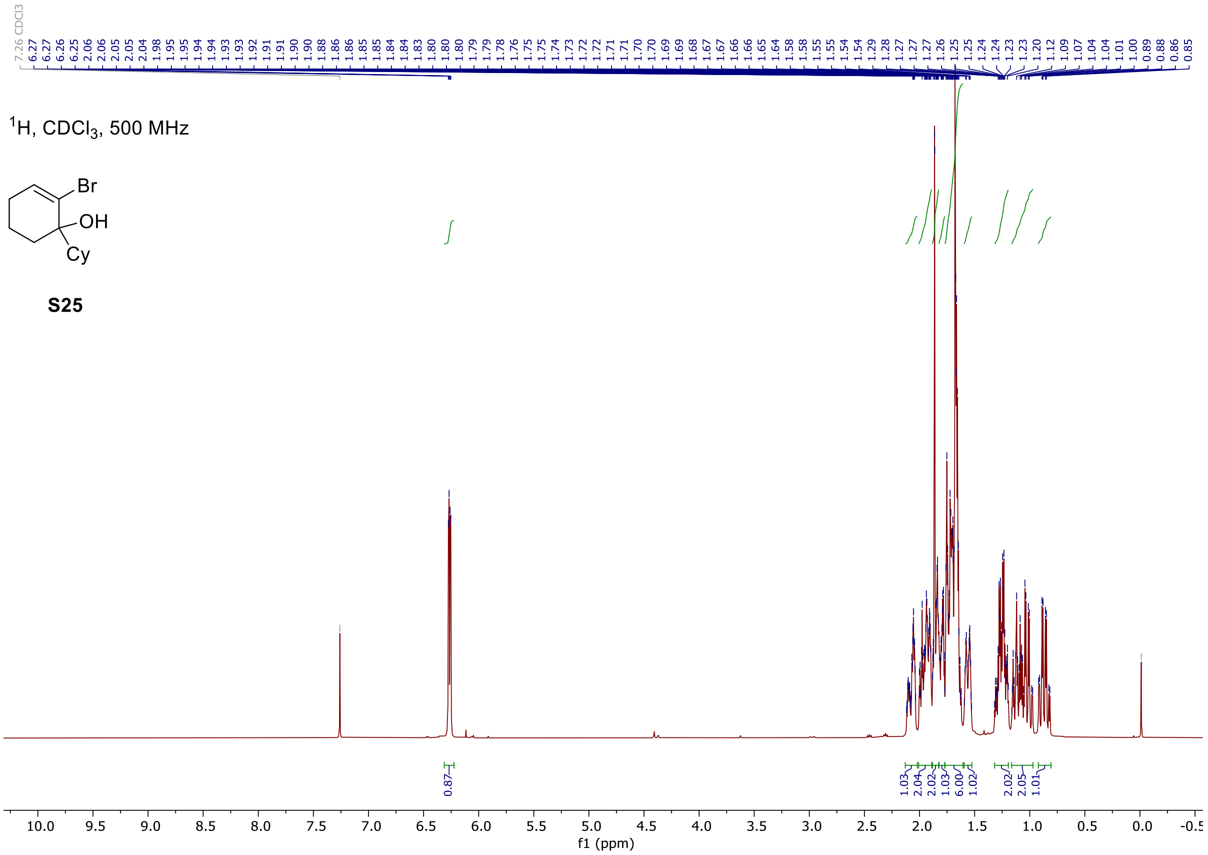

^{13}C, $CDCl_3$, 126 MHz

S25

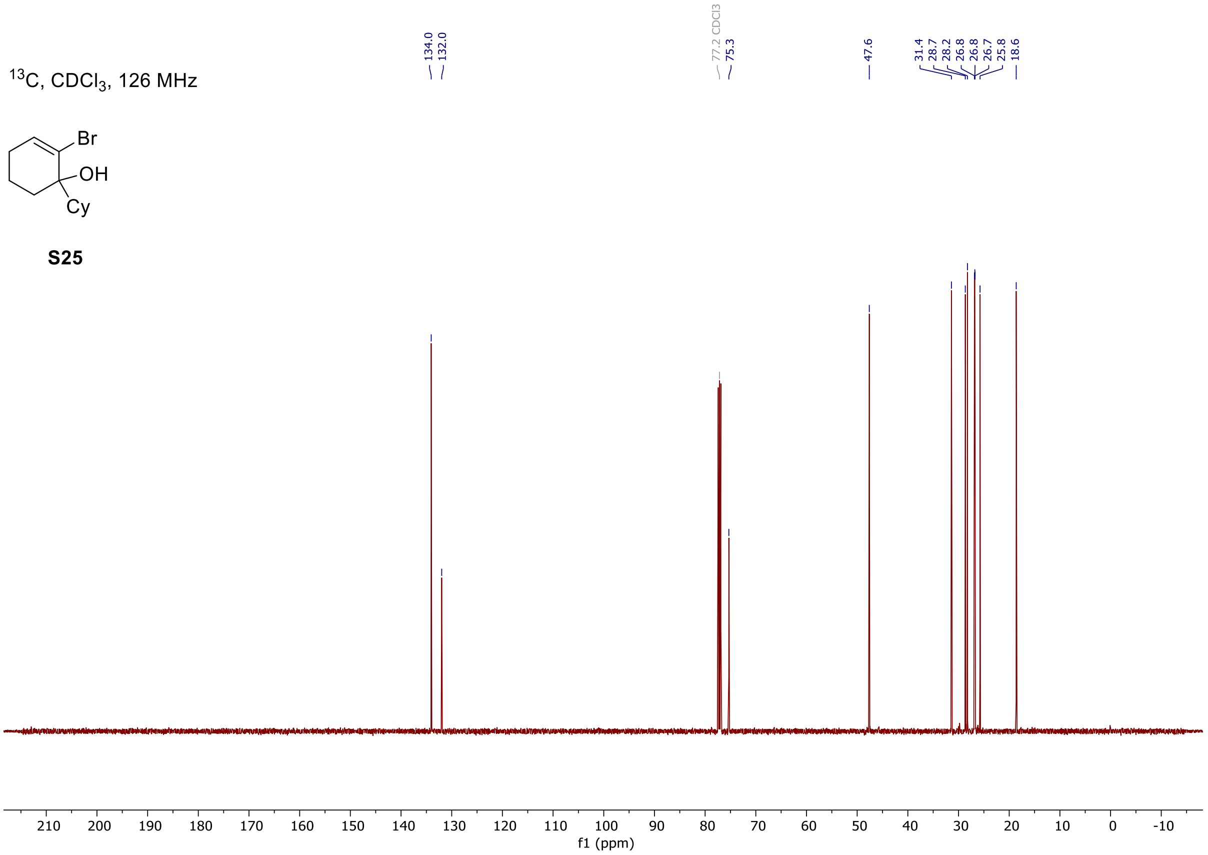

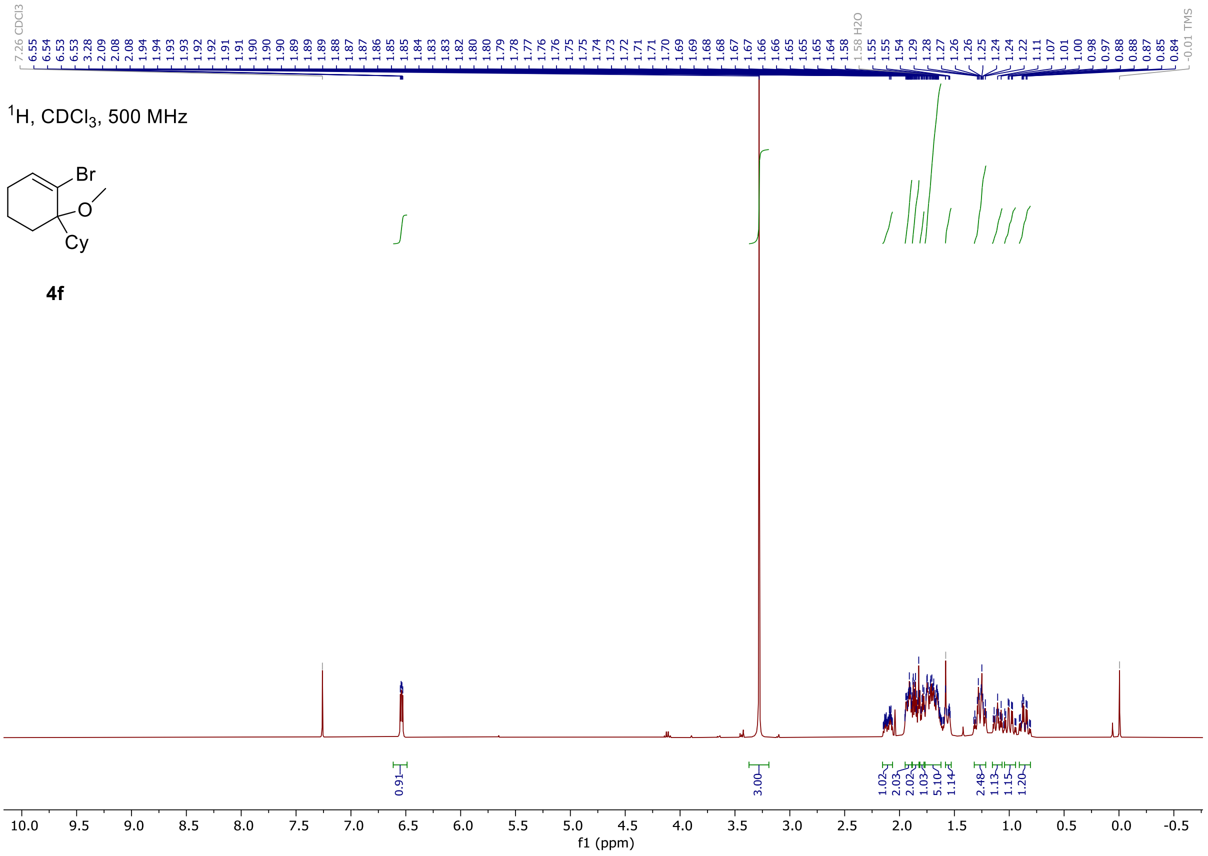
7.26 CDCl3
6.55 6.54 6.53 6.53 3.28 2.09 2.08 2.08 1.94 1.94 1.93 1.93 1.92 1.92 1.91 1.91 1.90 1.90 1.90 1.89 1.89 1.89 1.88 1.87 1.87 1.86 1.85 1.85 1.84 1.83 1.83 1.82 1.80 1.80 1.79 1.78 1.77 1.76 1.76 1.75 1.75 1.74 1.73 1.72 1.71 1.71 1.70 1.69 1.69 1.68 1.68 1.67 1.67 1.66 1.66 1.65 1.65 1.64 1.58
1.58 H2O
1.55 1.55 1.54 1.29 1.28 1.27 1.26 1.26 1.25 1.24 1.24 1.22 1.11 1.07 1.01 1.00 0.98 0.97 0.88 0.88 0.87 0.85 0.84
-0.01 TMS
^{1}H, $CDCl_3$, 500 MHz
Br
O
Cy
4f
0.91
3.00
1.02 2.03 2.02 1.03 5.10 1.14 2.48 1.13 1.15 1.20
10.0 9.5 9.0 8.5 8.0 7.5 7.0 6.5 6.0 5.5 5.0 4.5 4.0 3.5 3.0 2.5 2.0 1.5 1.0 0.5 0.0 -0.5
f1 (ppm)

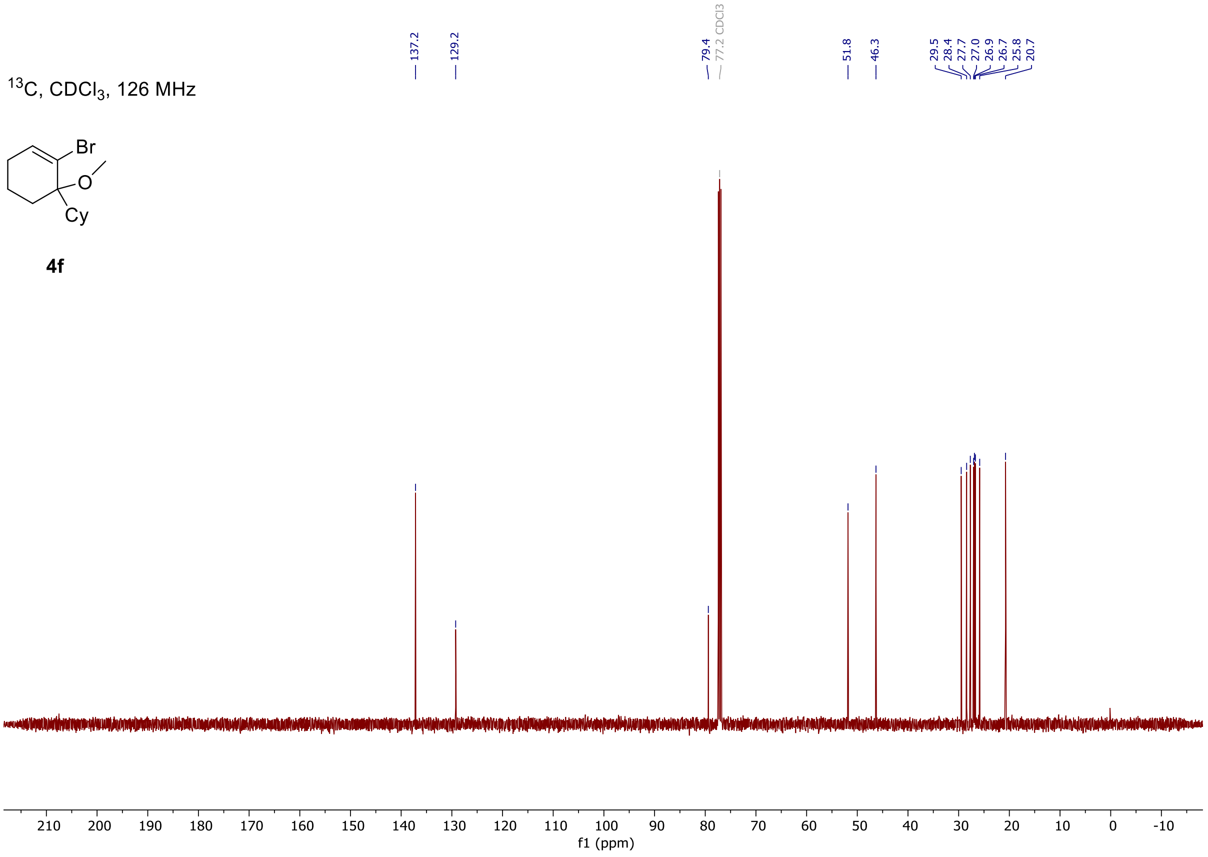
137.2
129.2
79.4
77.2 CDCl3
51.8
46.3
29.5 28.4 27.7 27.0 26.9 26.7 25.8 20.7
^{13}C, $CDCl_3$, 126 MHz
Br
O
Cy
4f
210 200 190 180 170 160 150 140 130 120 110 100 90 80 70 60 50 40 30 20 10 0 -10
f1 (ppm)

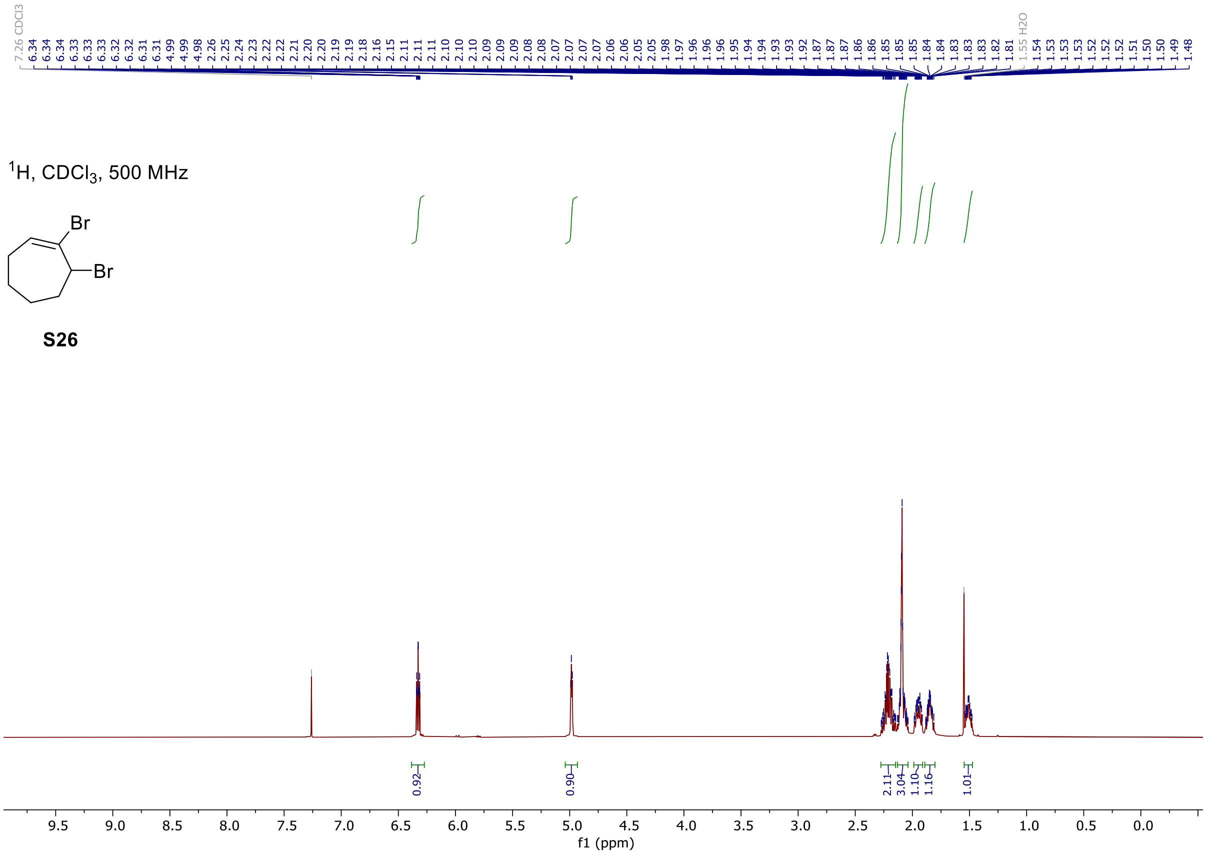

1H, CDCl3, 500 MHz
Br
Br
S26
f1 (ppm)

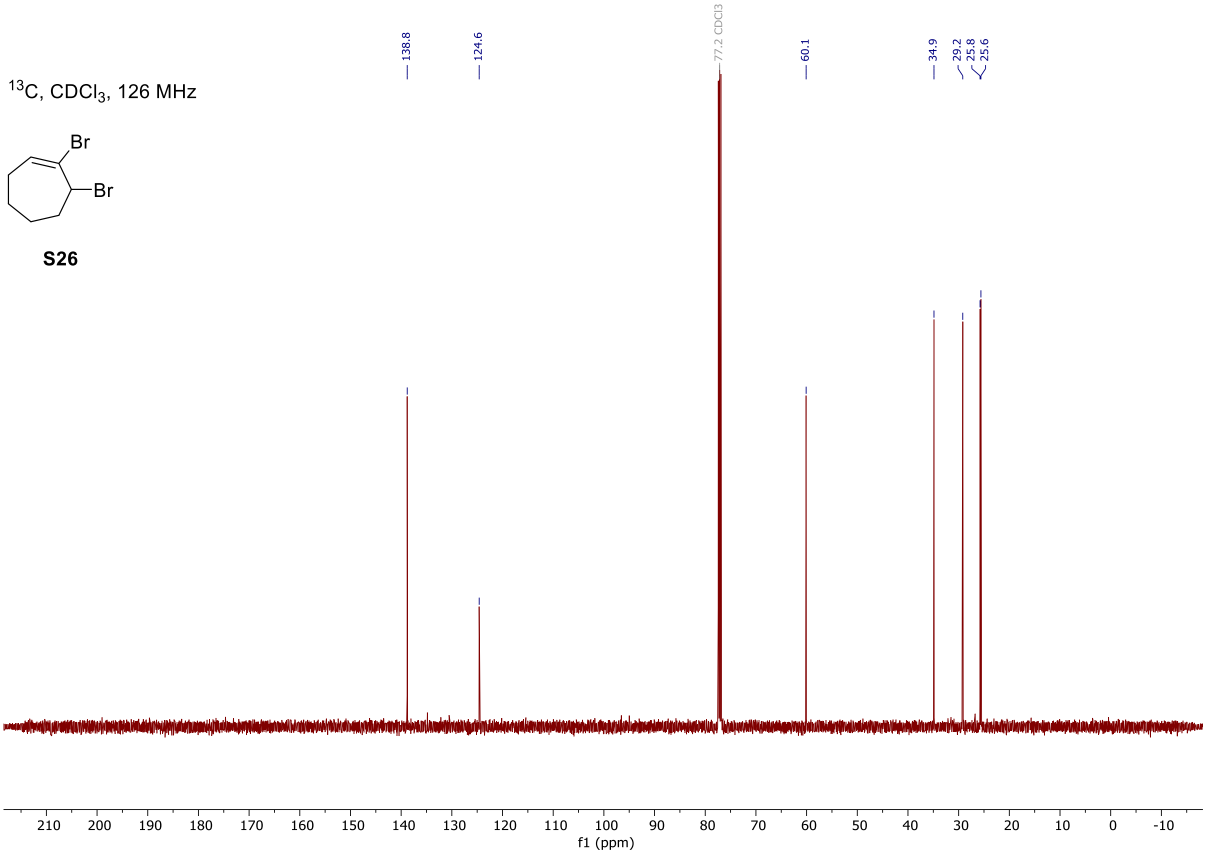

13C, CDCl3, 126 MHz
138.8
124.6
77.2 CDCl3
60.1
34.9
29.2
25.8
25.6
Br
Br
S26
f1 (ppm)

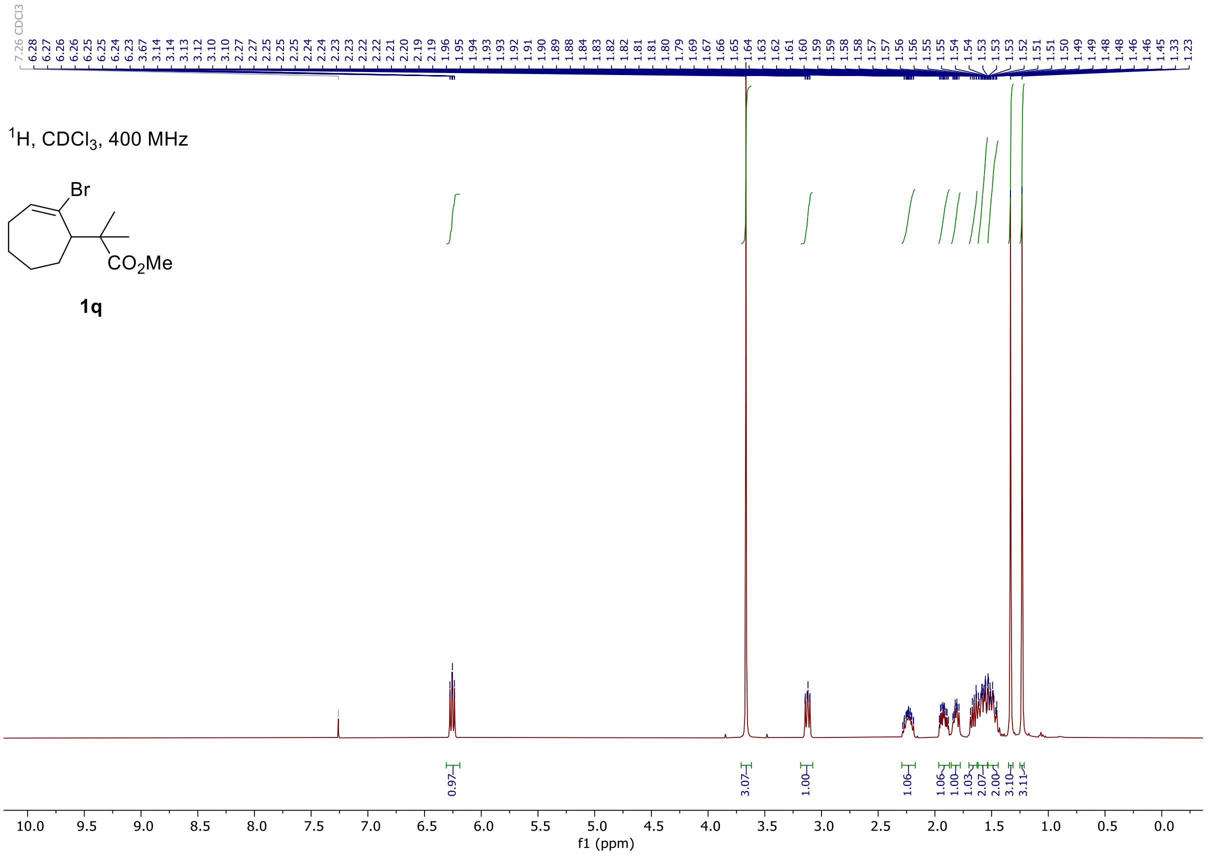
7.26 CDCl3
6.28 6.27 6.26 6.26 6.25 6.25 6.24 6.23 3.67 3.14 3.13 3.13 3.12 3.10 3.10 2.27 2.27 2.25 2.25 2.24 2.24 2.23 2.23 2.22 2.22 2.21 2.20 2.19 2.19 1.96 1.95 1.94 1.93 1.93 1.92 1.91 1.90 1.89 1.88 1.84 1.83 1.82 1.82 1.81 1.81 1.80 1.79 1.69 1.67 1.66 1.65 1.64 1.63 1.62 1.61 1.60 1.59 1.59 1.58 1.58 1.57 1.57 1.56 1.56 1.55 1.55 1.54 1.54 1.53 1.53 1.53 1.52 1.51 1.51 1.50 1.49 1.48 1.46 1.45 1.33 1.23
1H, CDCl3, 400 MHz
Br
CO2Me
1q
0.97
3.07
1.00
1.06
1.06
1.00
1.03
2.07
2.00
3.10
3.11
10.0 9.5 9.0 8.5 8.0 7.5 7.0 6.5 6.0 5.5 5.0 4.5 4.0 3.5 3.0 2.5 2.0 1.5 1.0 0.5 0.0
f1 (ppm)

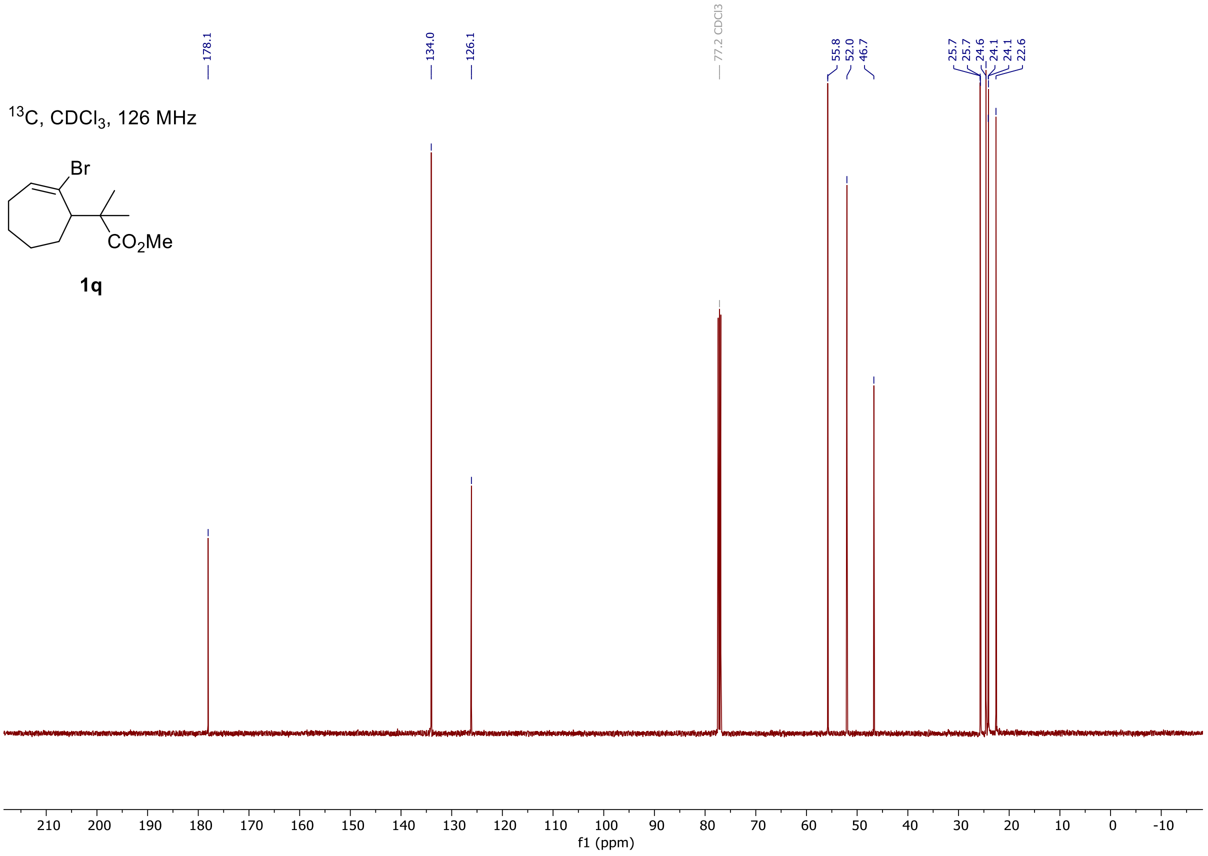
178.1
134.0
126.1
77.2 CDCl3
55.8
52.0
46.7
25.7
25.7
24.6
24.1
24.1
22.6
13C, CDCl3, 126 MHz
Br
CO2Me
1q
210 200 190 180 170 160 150 140 130 120 110 100 90 80 70 60 50 40 30 20 10 0 -10
f1 (ppm)

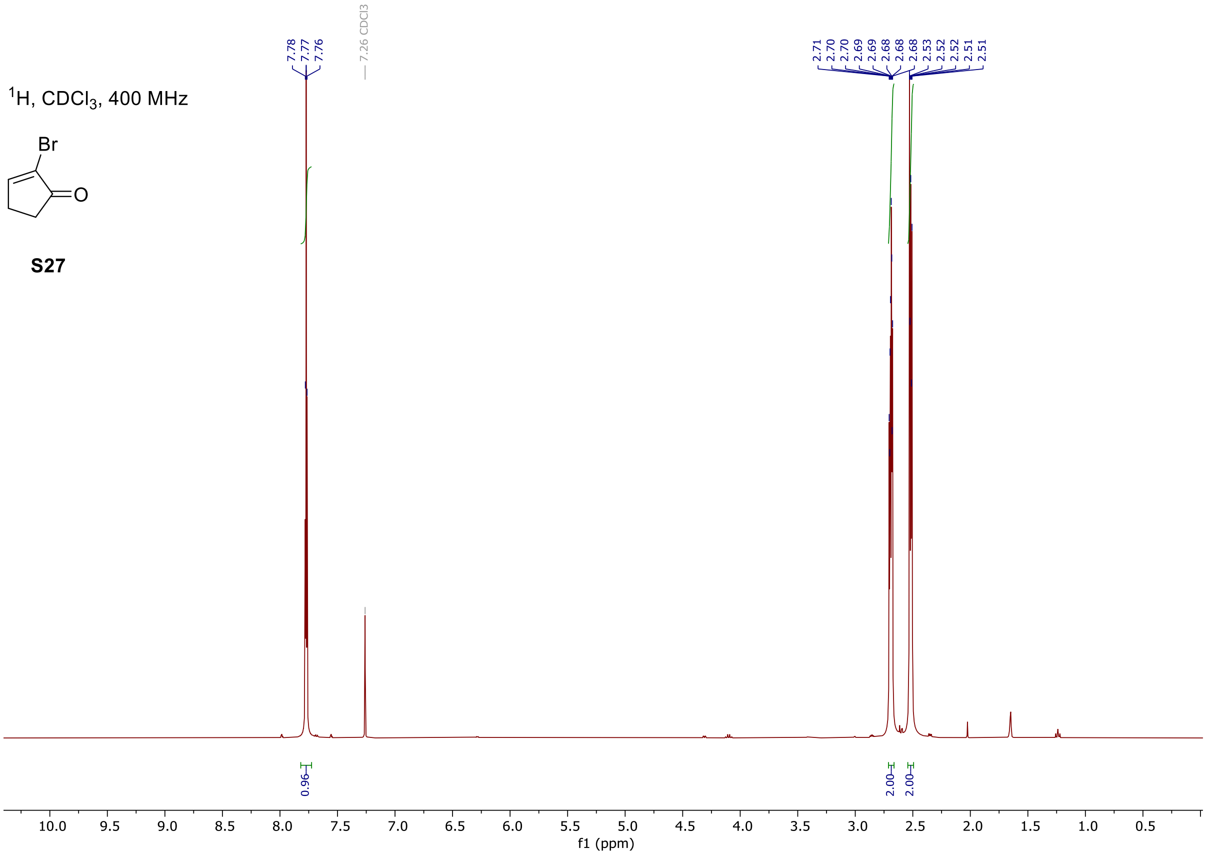

^{1}H, $CDCl_3$, 400 MHz
Br
O
S27
7.78
7.77
7.76
7.26 CDCl3
2.71
2.70
2.70
2.69
2.69
2.68
2.68
2.68
2.53
2.52
2.52
2.51
2.51
0.96
2.00
2.00
f1 (ppm)

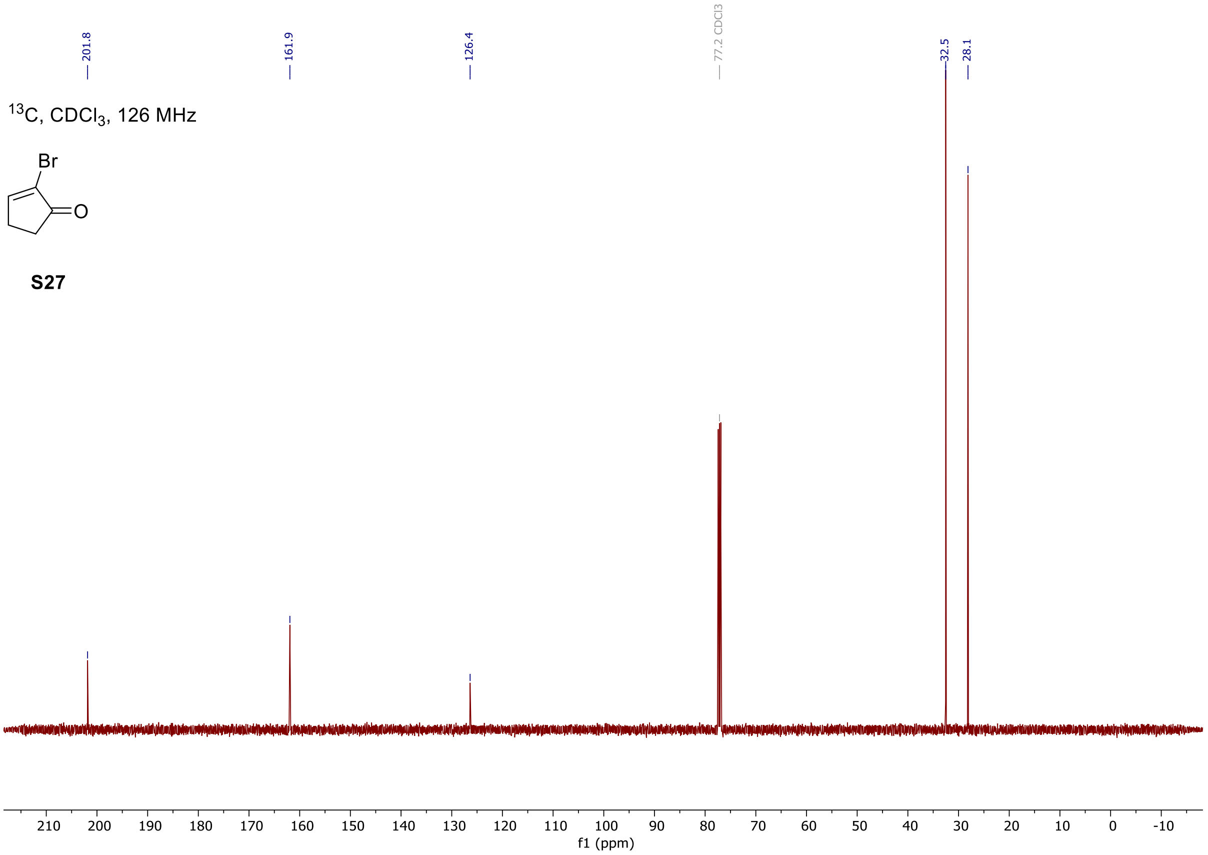

^{13}C, $CDCl_3$, 126 MHz
Br
O
S27
201.8
161.9
126.4
77.2 CDCl3
32.5
28.1
f1 (ppm)

^{1}H, $CDCl_3$, 500 MHz

4g

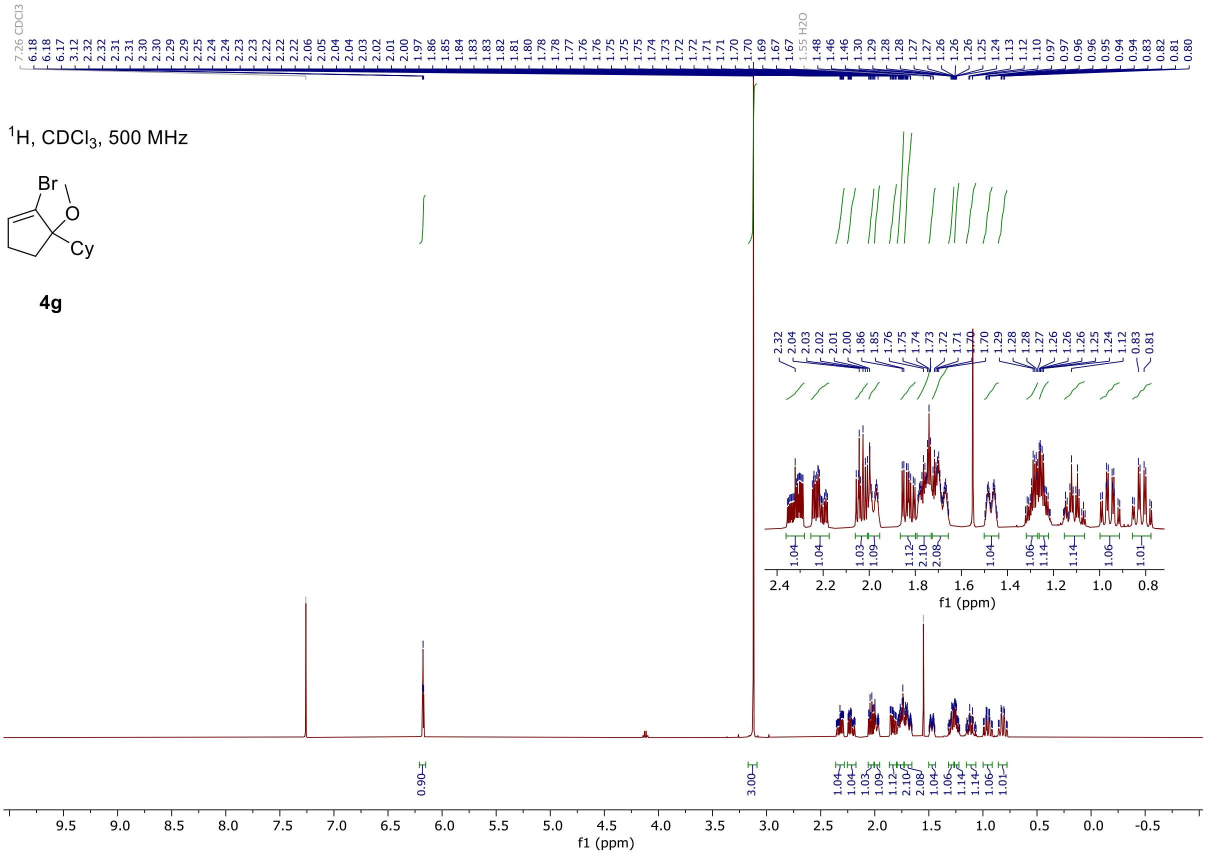

^{13}C, $CDCl_3$, 126 MHz

4g

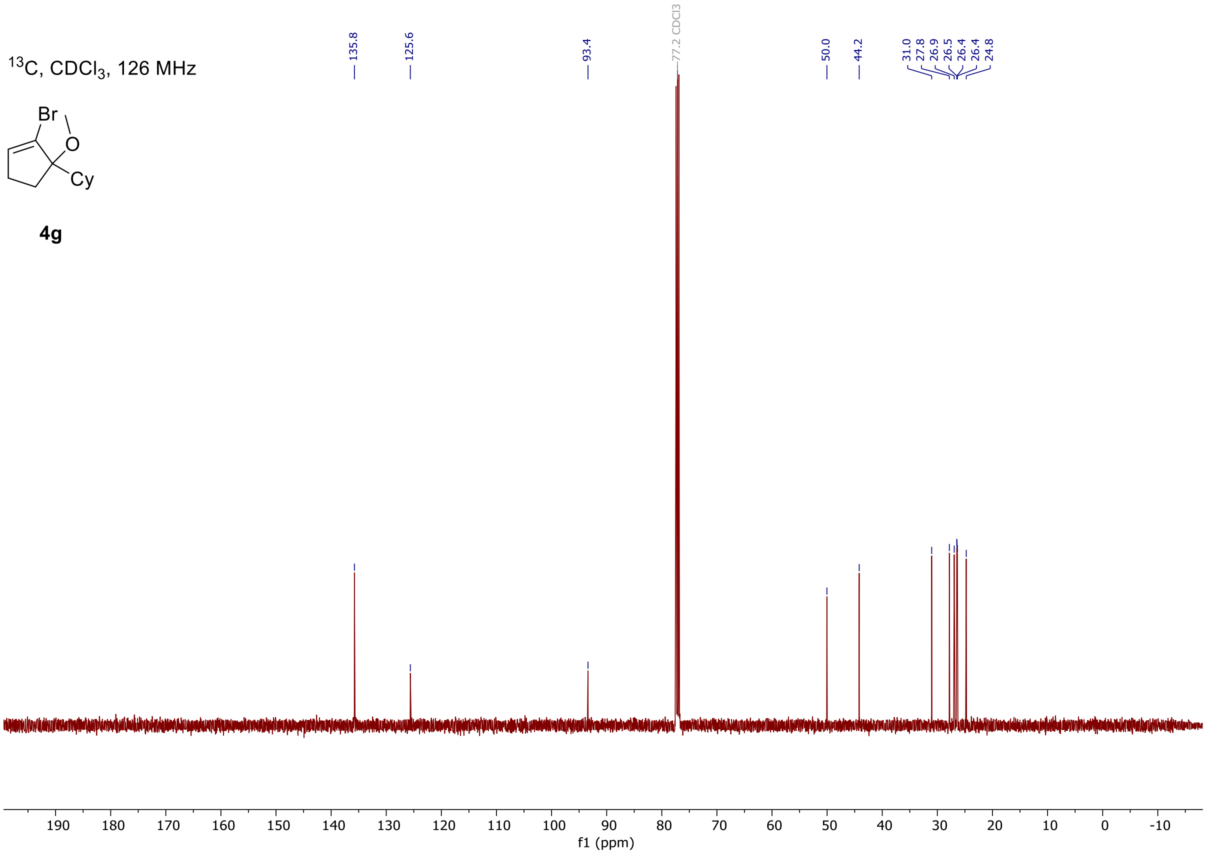

C–H activation products

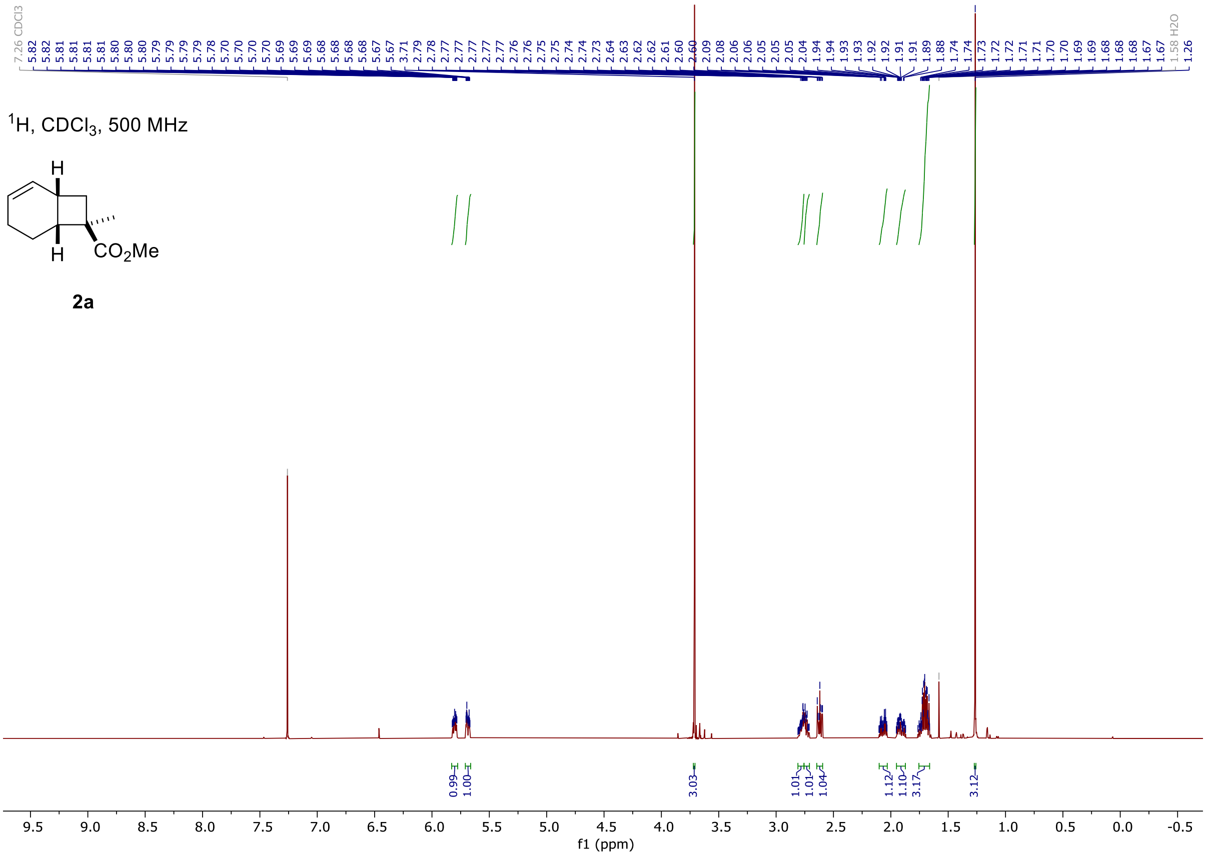

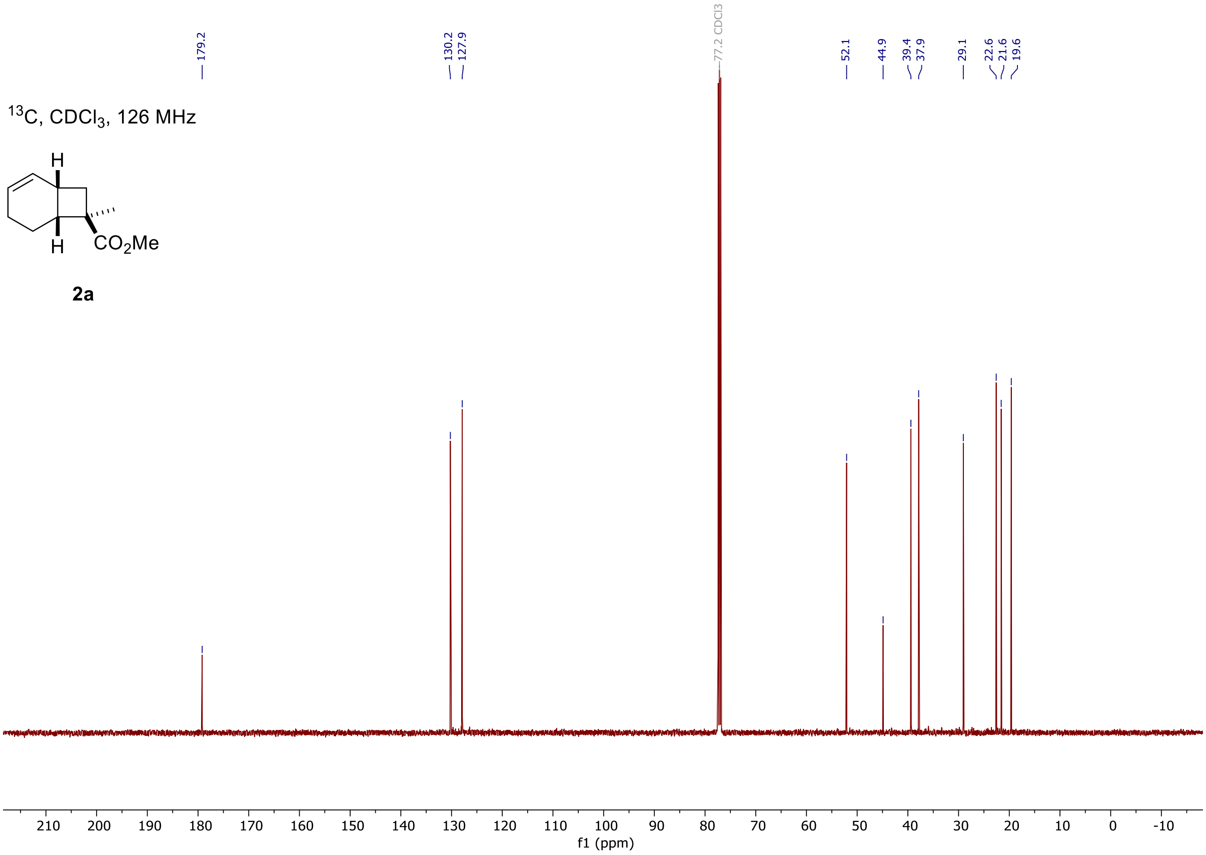

NOESY of **2a** (zoomed in).

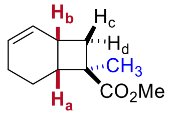

2a

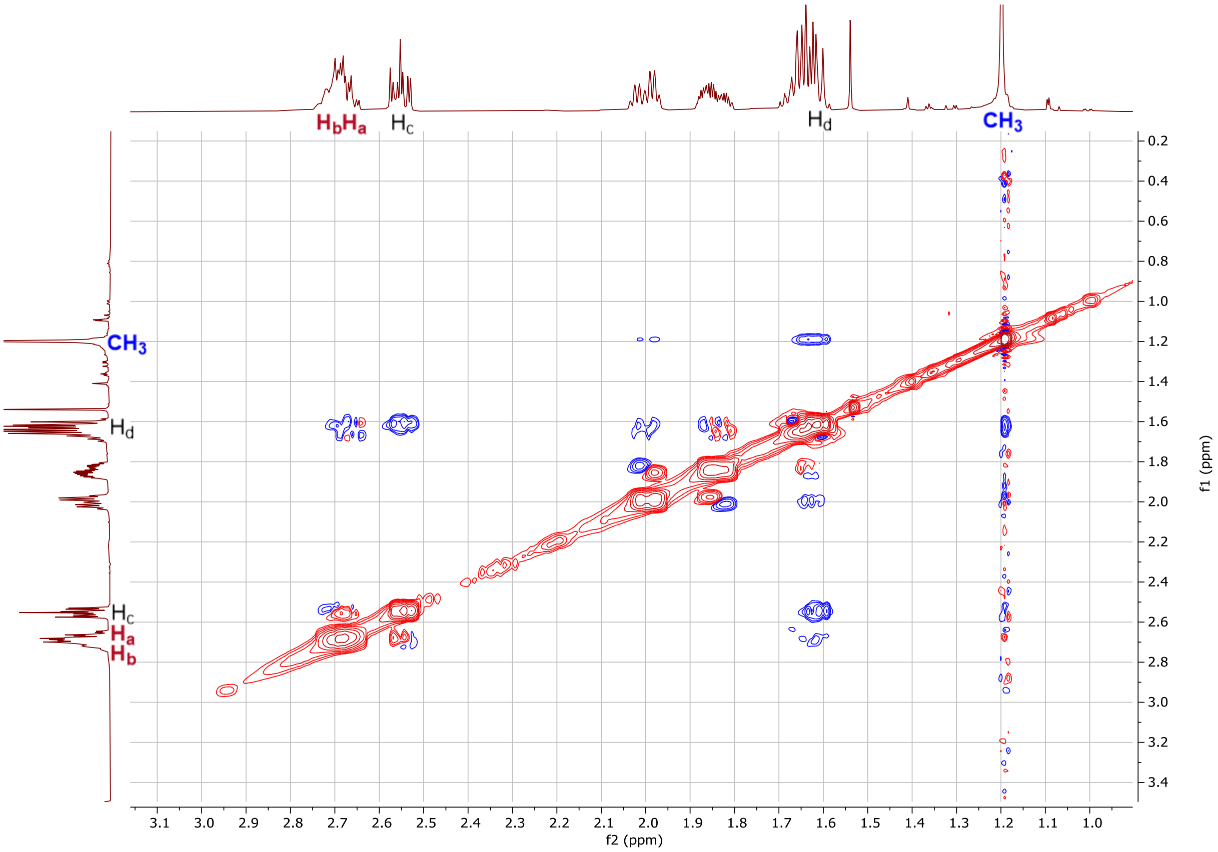

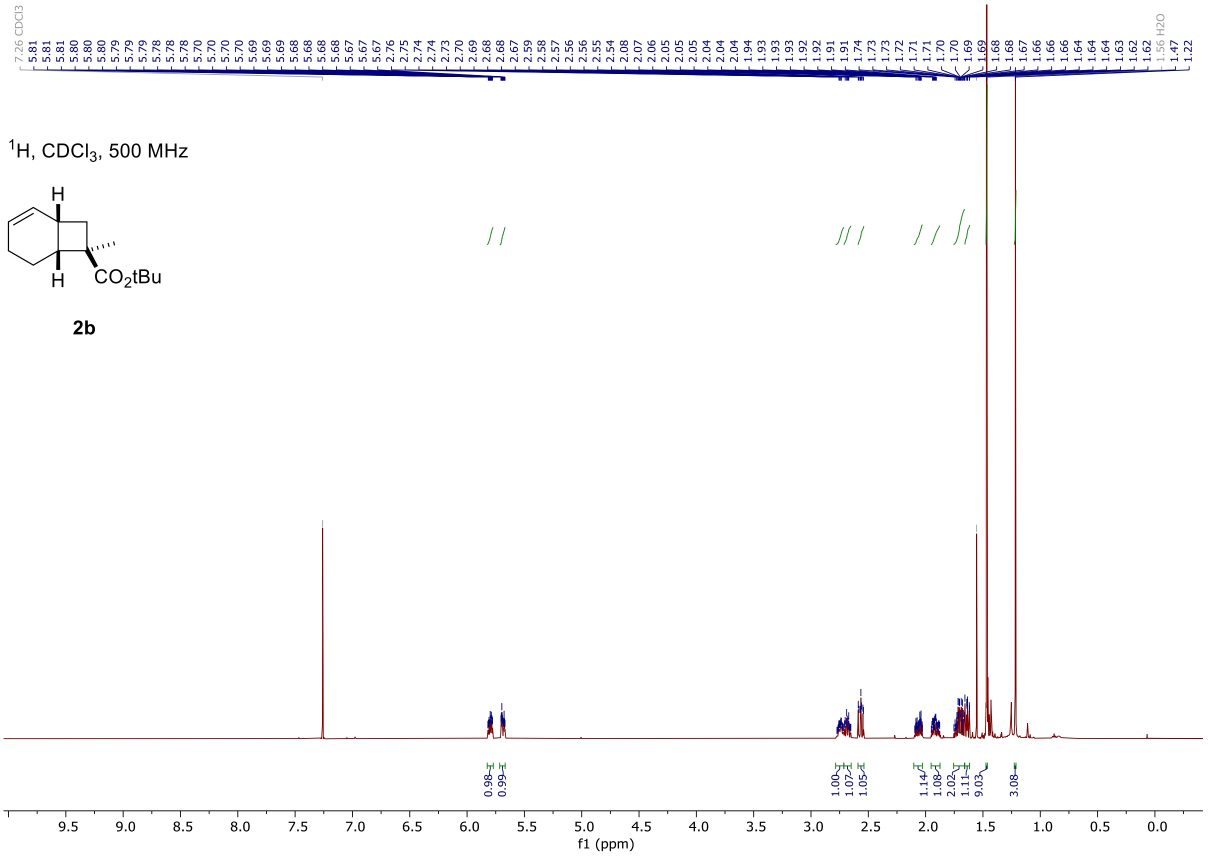
7.26 CDCl3
5.81 5.81 5.81 5.80 5.80 5.80 5.79 5.79 5.79 5.79 5.78 5.78 5.78 5.70 5.70 5.70 5.69 5.69 5.69 5.68 5.68 5.68 5.68 5.67 5.67 5.67 2.76 2.75 2.74 2.73 2.70 2.69 2.68 2.68 2.67 2.59 2.58 2.57 2.56 2.56 2.55 2.54 2.08 2.07 2.06 2.05 2.05 2.04 2.04 2.04 1.94 1.93 1.93 1.93 1.92 1.92 1.91 1.91 1.74 1.73 1.73 1.72 1.71 1.71 1.70 1.70 1.69 1.68 1.68 1.67 1.66 1.66 1.64 1.64 1.63 1.62 1.62
1.56 H2O
1.47 1.22
^{1}H, CDCl$_3$, 500 MHz
H
H
CO$_2$tBu
2b
0.98 0.99 1.00 1.07 1.05 1.14 1.08 2.02 1.11 9.03 3.08
9.5 9.0 8.5 8.0 7.5 7.0 6.5 6.0 5.5 5.0 4.5 4.0 3.5 3.0 2.5 2.0 1.5 1.0 0.5 0.0
f1 (ppm)

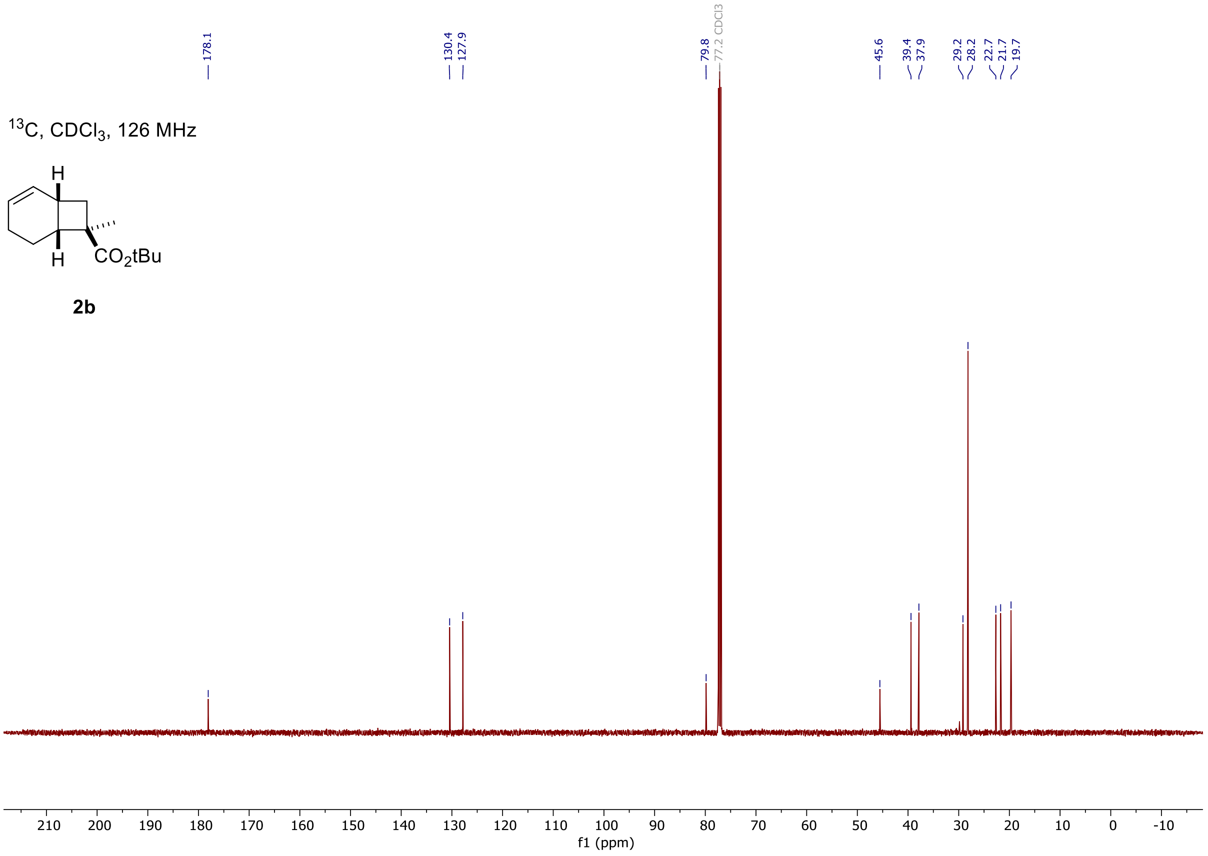
178.1
130.4 127.9
79.8 77.2 CDCl3
45.6
39.4 37.9
29.2 28.2
22.7 21.7 19.7
^{13}C, CDCl$_3$, 126 MHz
H
H
CO$_2$tBu
2b
210 200 190 180 170 160 150 140 130 120 110 100 90 80 70 60 50 40 30 20 10 0 -10
f1 (ppm)

NOESY of **2b** (zoomed in).

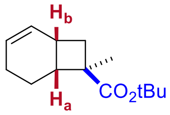

2b

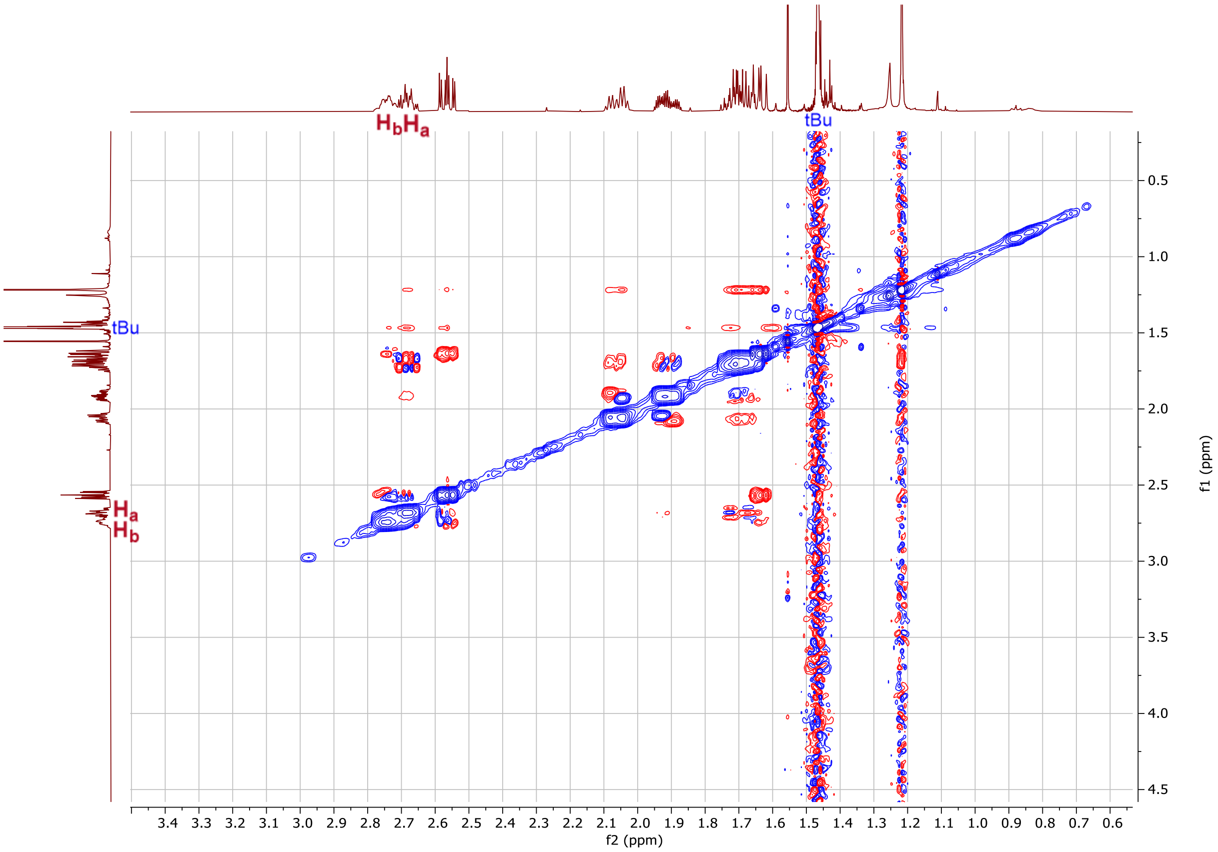

^{1}H, $CDCl_3$, 500 MHz

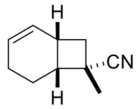

d.r. 1.15:1
2c

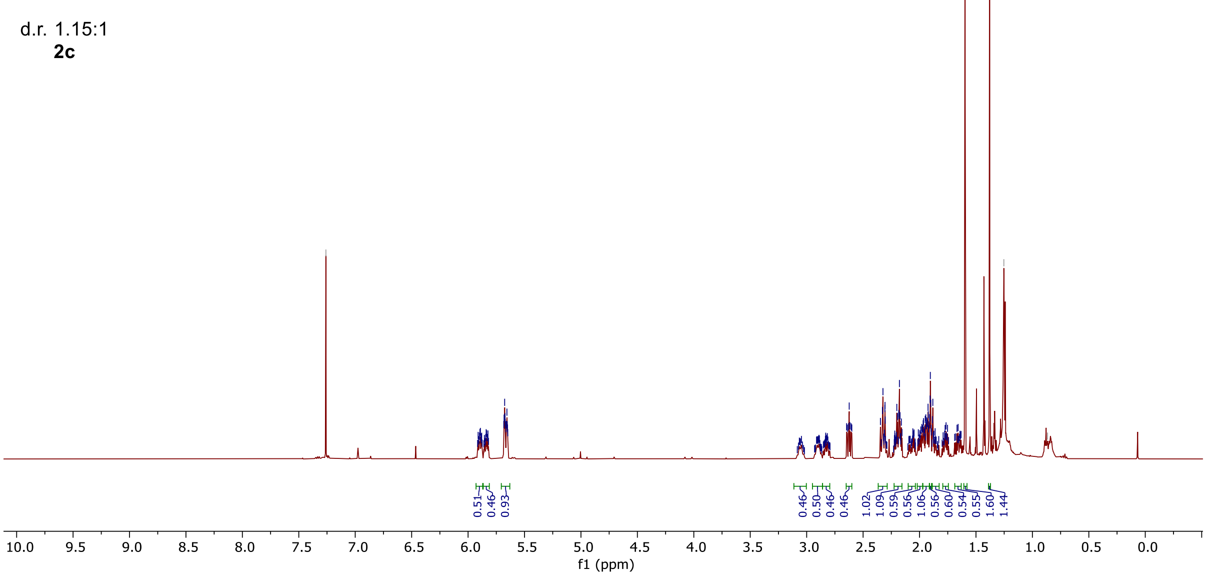

^{13}C, $CDCl_3$, 126 MHz

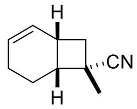

d.r. 1.15:1
2c

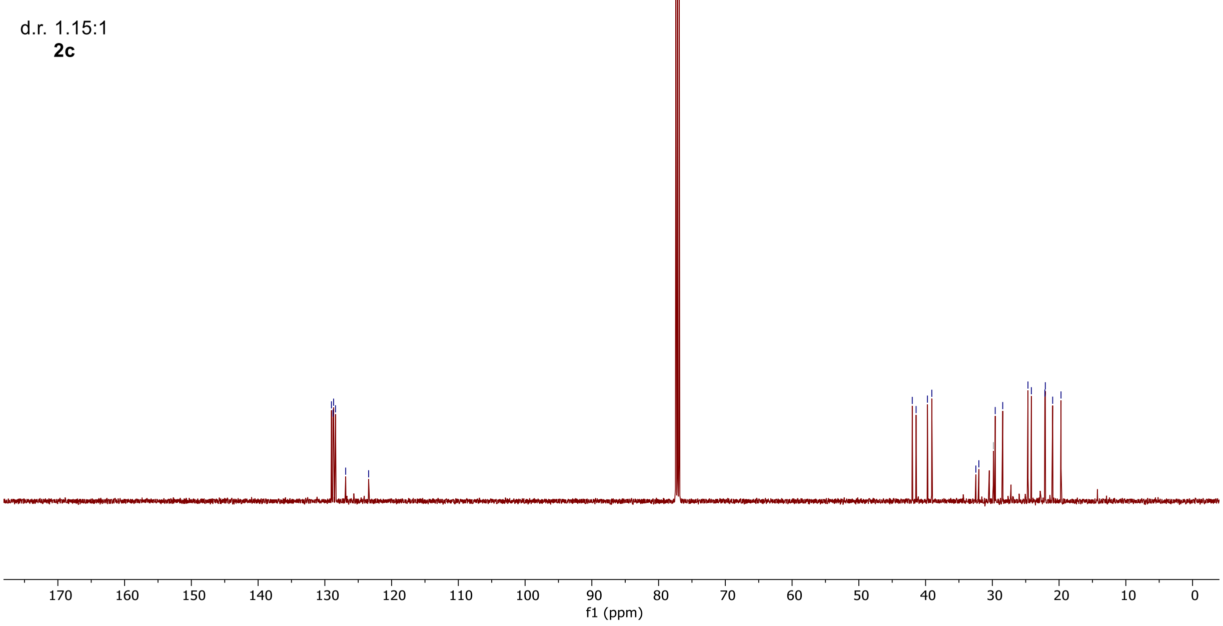

NOESY of **2c**.

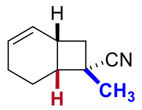

2c

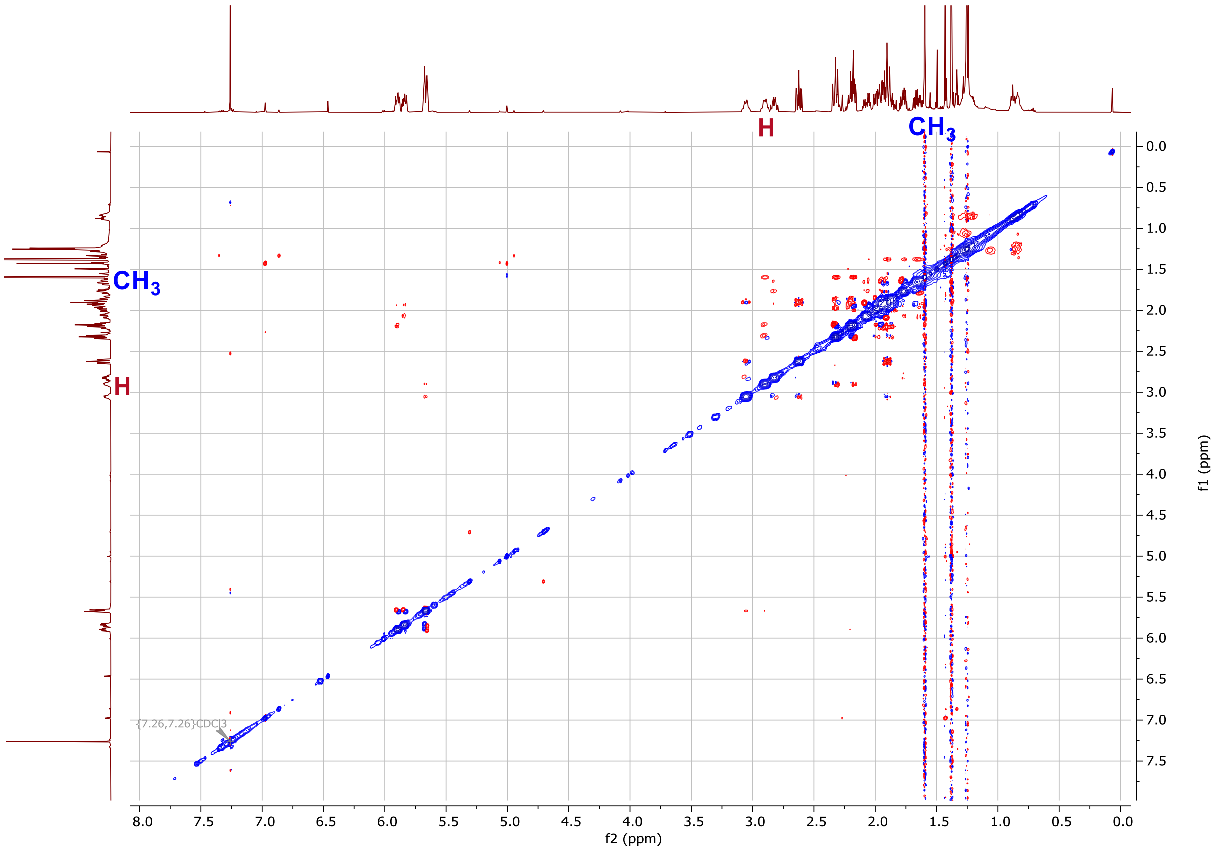

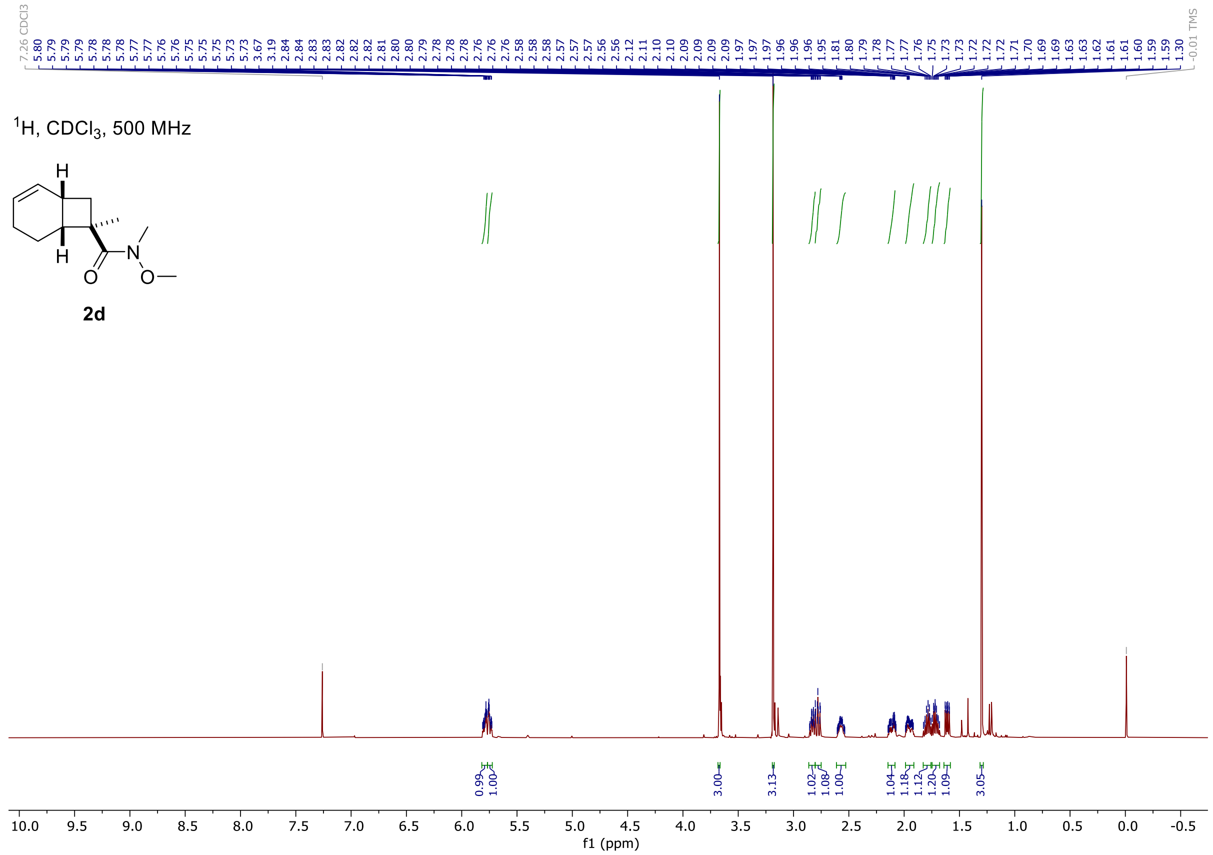
7.26 CDCl3
5.80 5.79 5.79 5.79 5.78 5.78 5.78 5.77 5.77 5.76 5.76 5.75 5.75 5.75 5.73 5.73 3.67 3.19 2.84 2.84 2.83 2.83 2.82 2.82 2.82 2.81 2.80 2.80 2.79 2.78 2.78 2.78 2.76 2.76 2.76 2.58 2.58 2.58 2.57 2.57 2.56 2.56 2.12 2.11 2.10 2.10 2.09 2.09 2.09 2.09 1.97 1.97 1.97 1.96 1.96 1.96 1.95 1.81 1.80 1.79 1.78 1.77 1.77 1.76 1.75 1.75 1.73 1.73 1.72 1.72 1.72 1.71 1.70 1.69 1.69 1.63 1.63 1.62 1.61 1.61 1.60 1.59 1.59 1.30
-0.01 TMS
^{1}H, CDCl$_3$, 500 MHz
H
H
N
O
O
2d
0.99 1.00 3.00 3.13 1.02 1.08 1.00 1.04 1.18 1.12 1.20 1.09 3.05
10.0 9.5 9.0 8.5 8.0 7.5 7.0 6.5 6.0 5.5 5.0 4.5 4.0 3.5 3.0 2.5 2.0 1.5 1.0 0.5 0.0 -0.5
f1 (ppm)

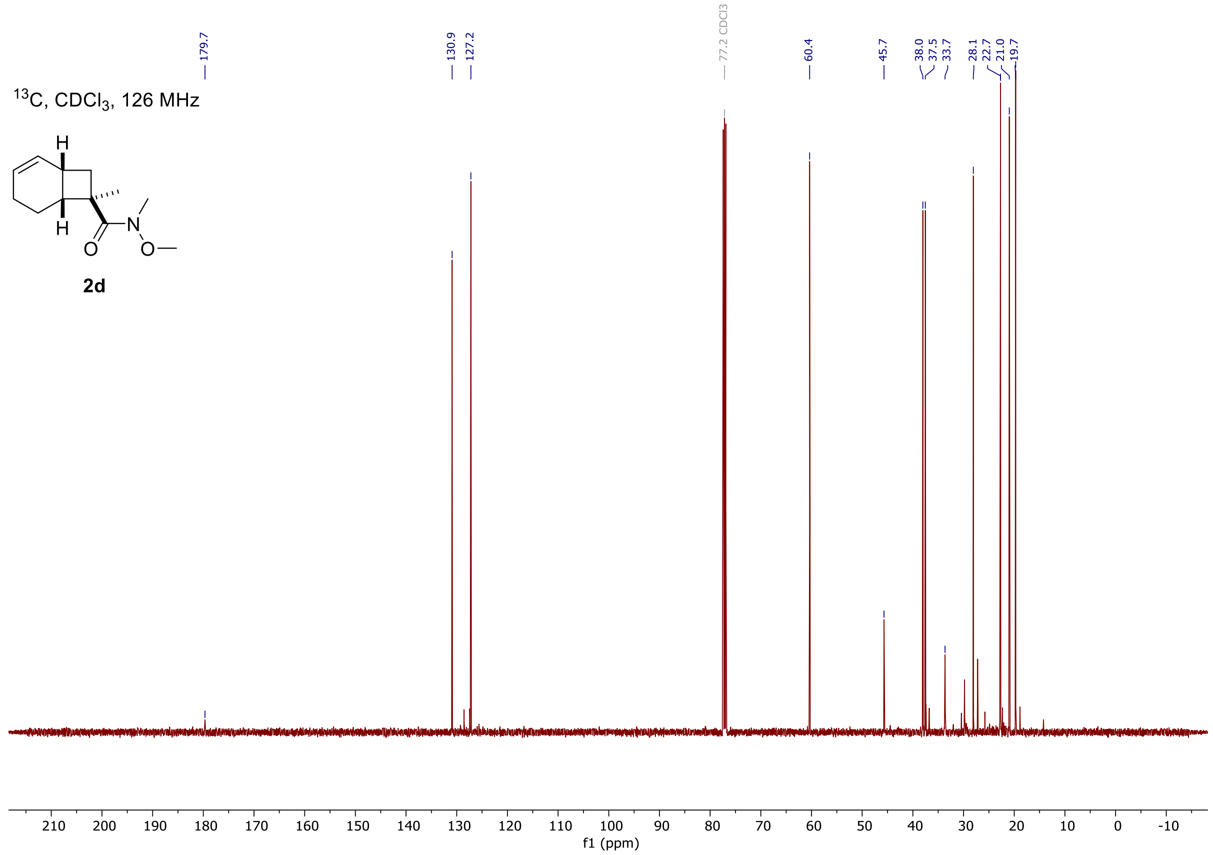
179.7
130.9
127.2
77.2 CDCl3
60.4
45.7
38.0
37.5
33.7
28.1
22.7
21.0
19.7
^{13}C, CDCl$_3$, 126 MHz
H
H
N
O
O
2d
210 200 190 180 170 160 150 140 130 120 110 100 90 80 70 60 50 40 30 20 10 0 -10
f1 (ppm)

NOESY of **2d**.

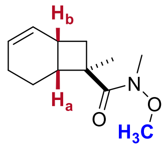

2d

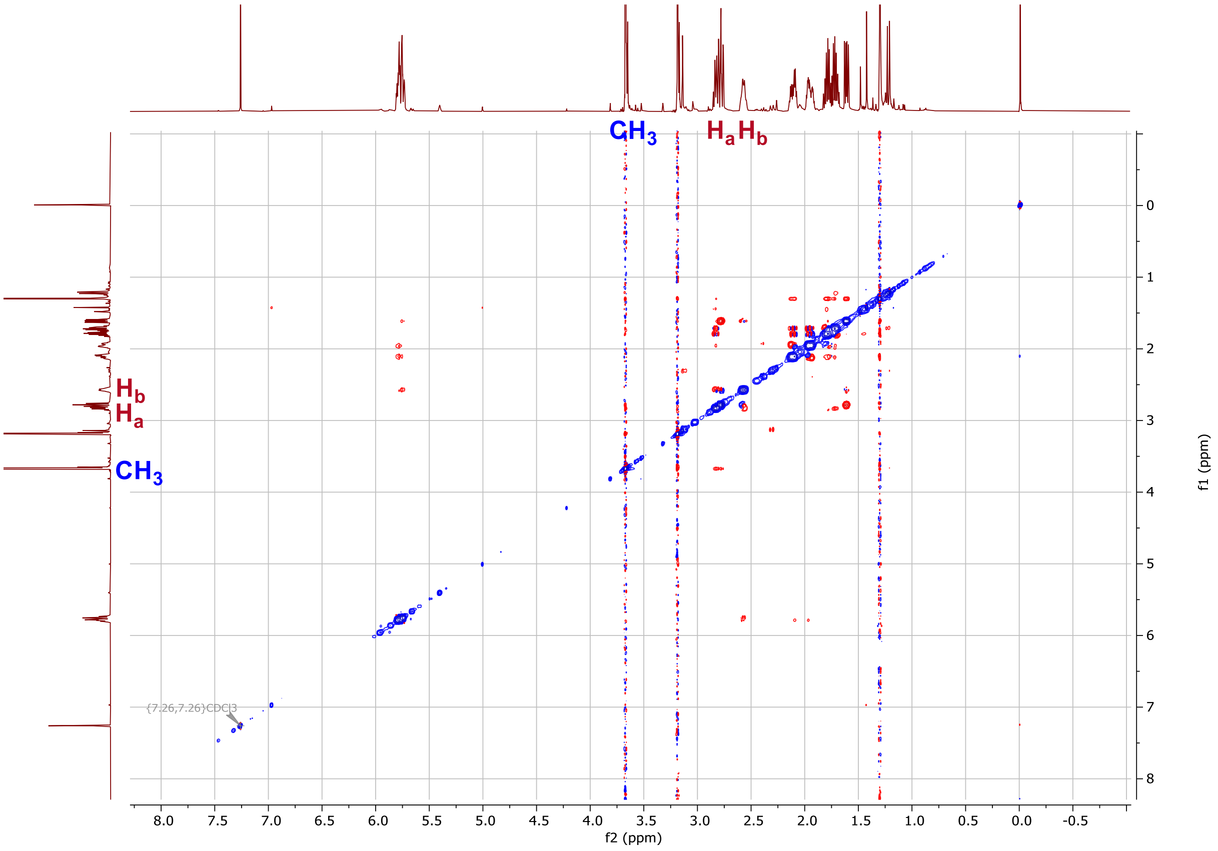

^{1}H, $CDCl_3$, 500 MHz

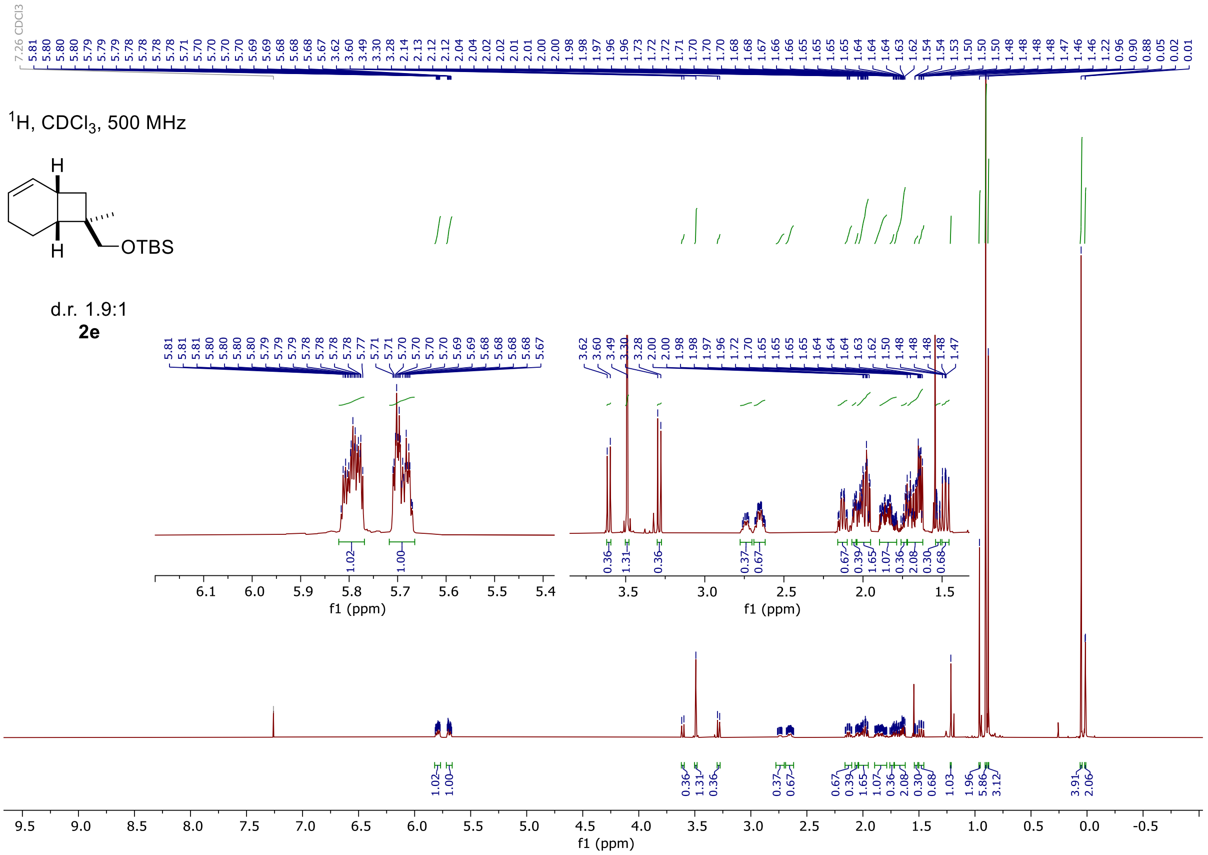

d.r. 1.9:1
2e

^{13}C, $CDCl_3$, 126 MHz

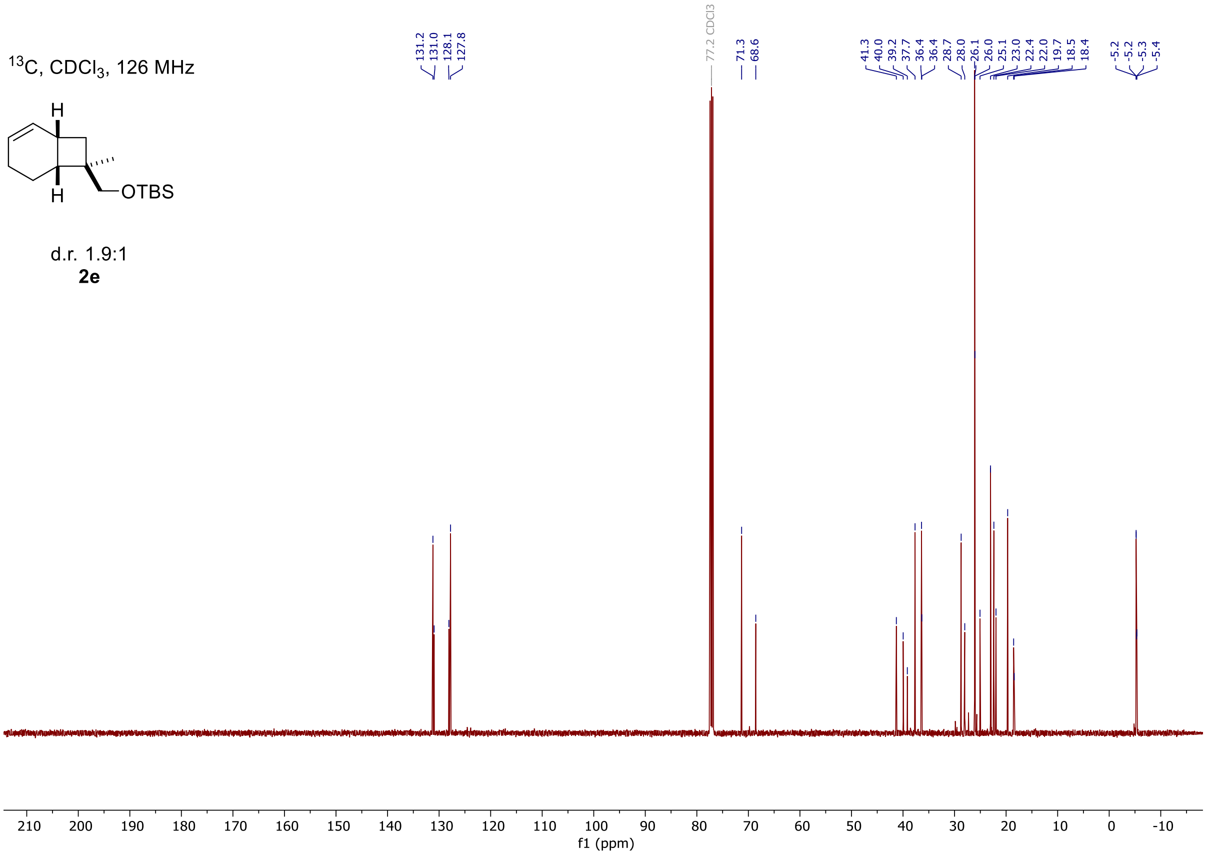

d.r. 1.9:1
2e

NOESY of **2e** (zoomed in).

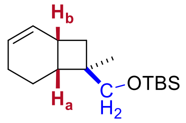

2e

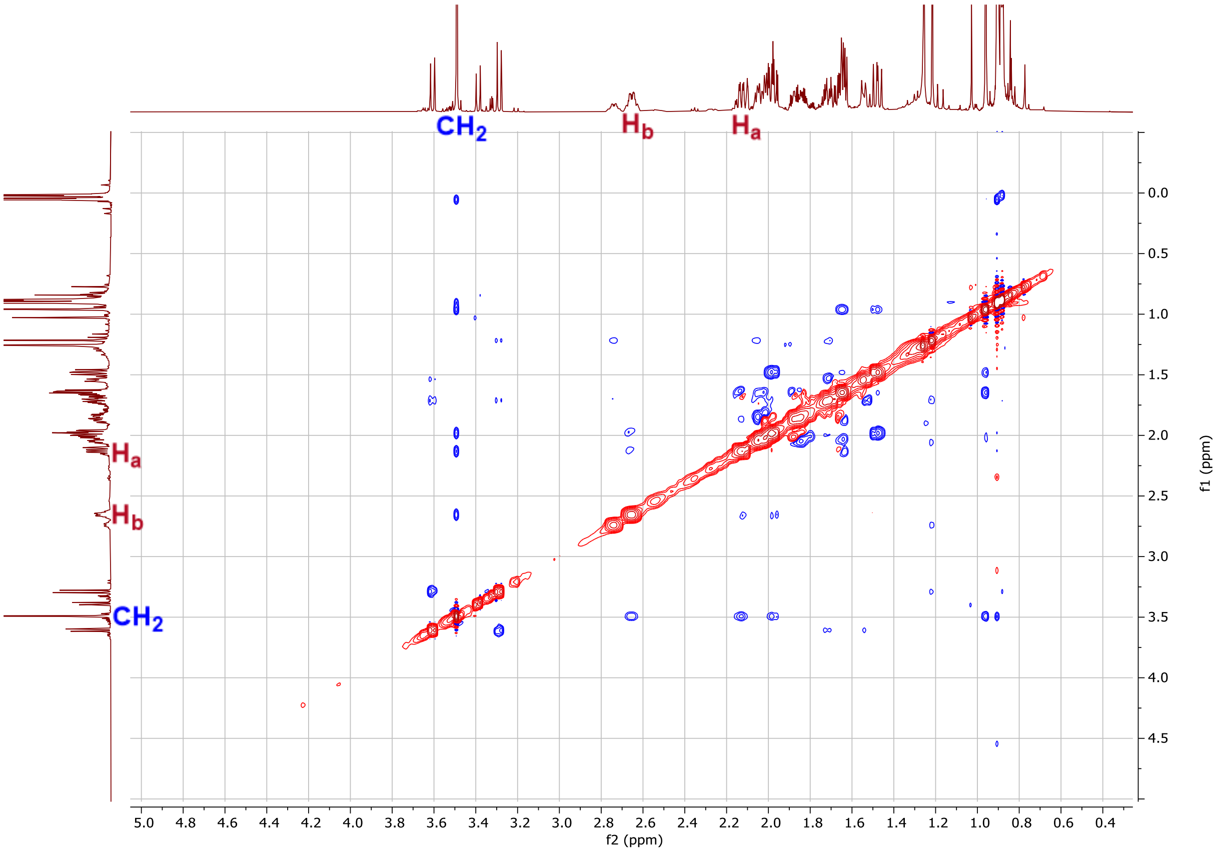

^{1}H, $CDCl_3$, 500 MHz

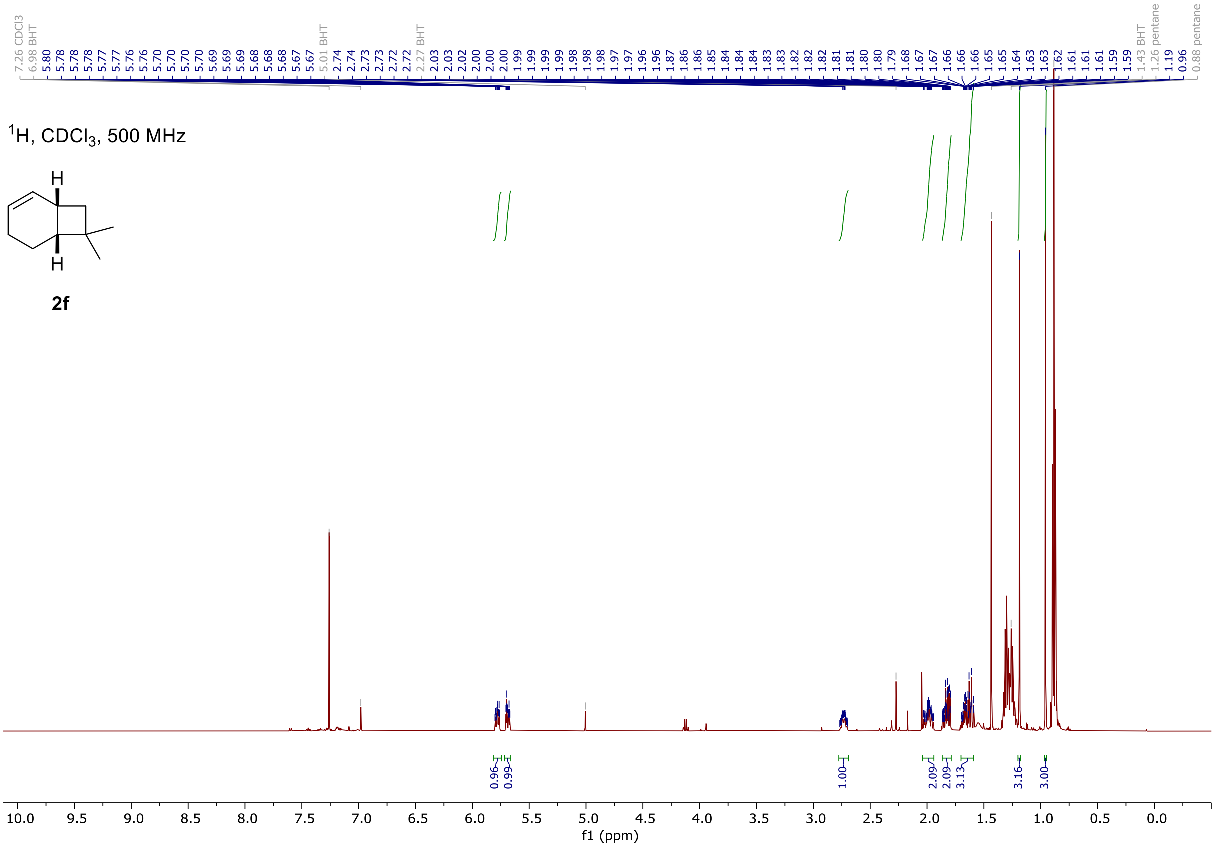

^{13}C, $CDCl_3$, 126 MHz

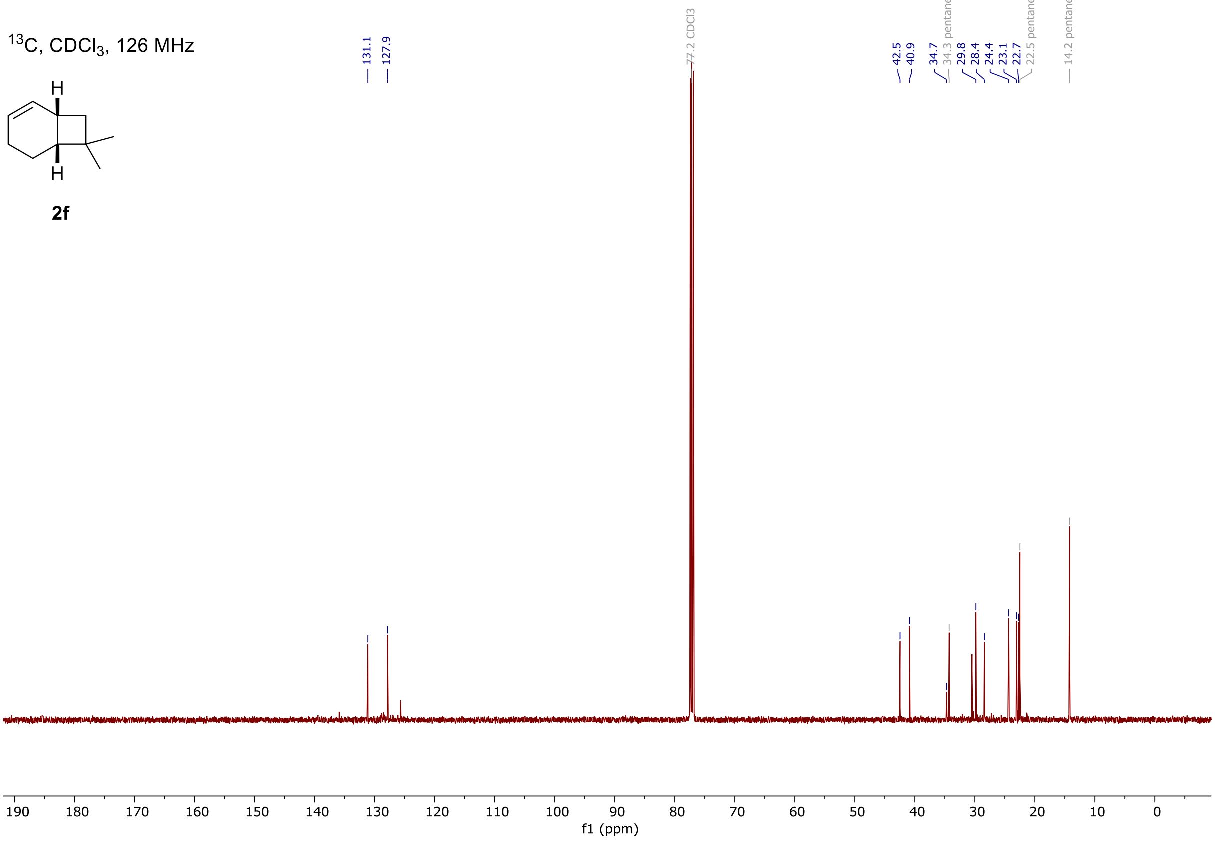

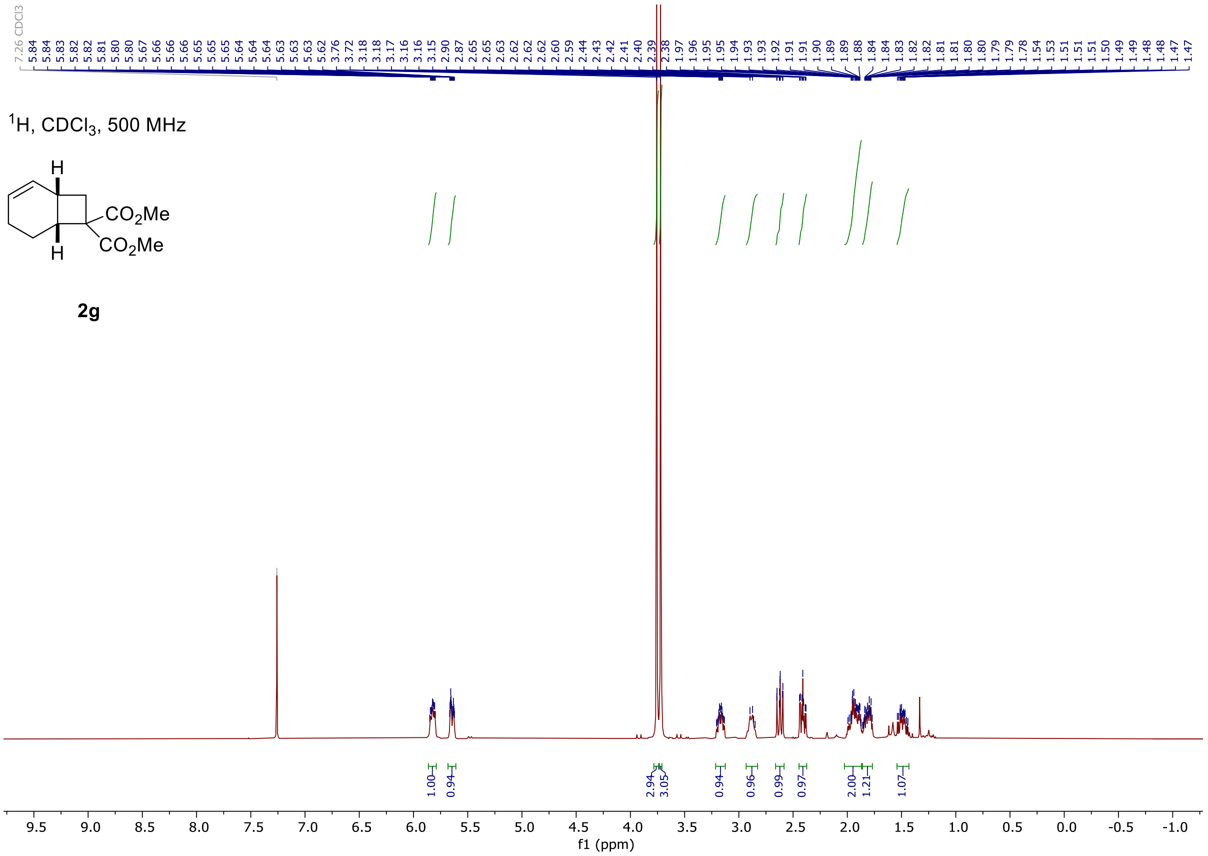

^{1}H, CDCl$_3$, 500 MHz
CO$_2$Me
CO$_2$Me
H
H
2g
f1 (ppm)

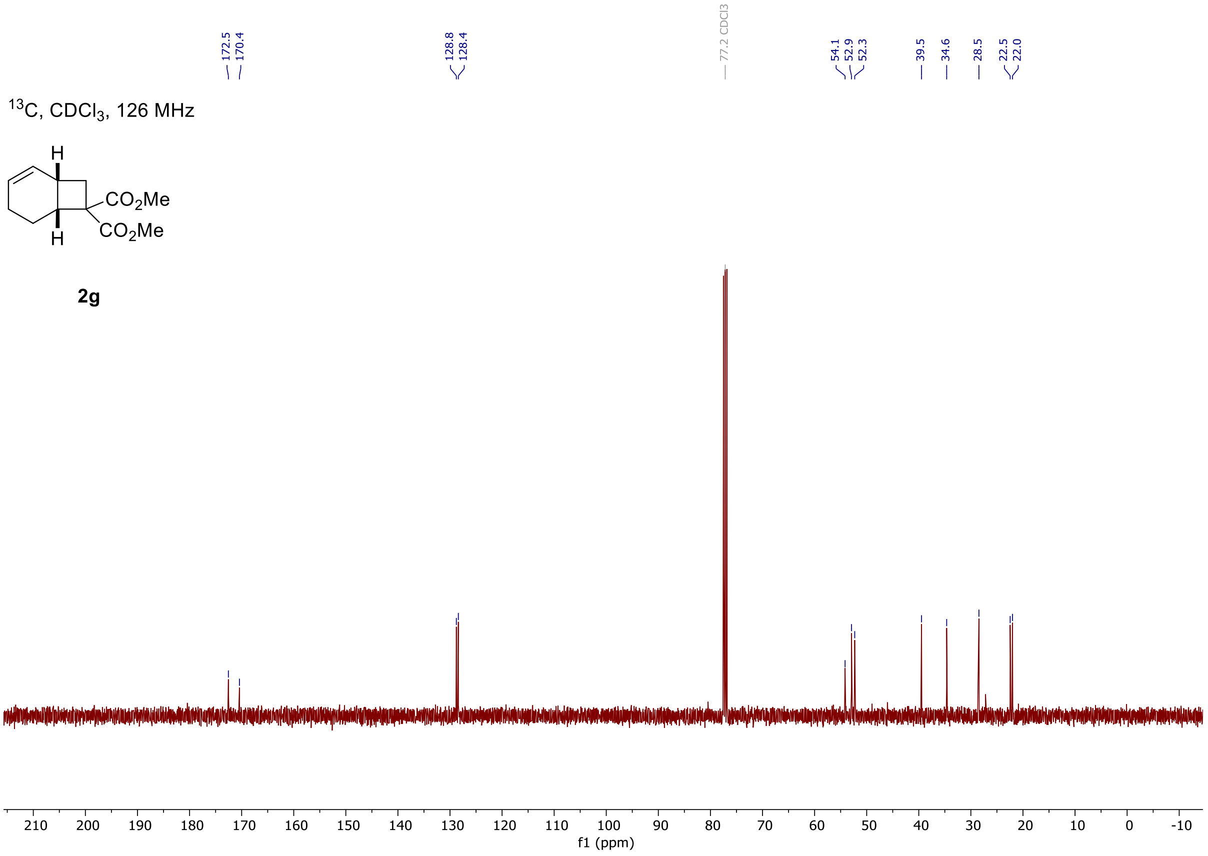

^{13}C, CDCl$_3$, 126 MHz
CO$_2$Me
CO$_2$Me
H
H
2g
172.5
170.4
128.8
128.4
77.2 CDCl3
54.1
52.9
52.3
39.5
34.6
28.5
22.5
22.0
f1 (ppm)

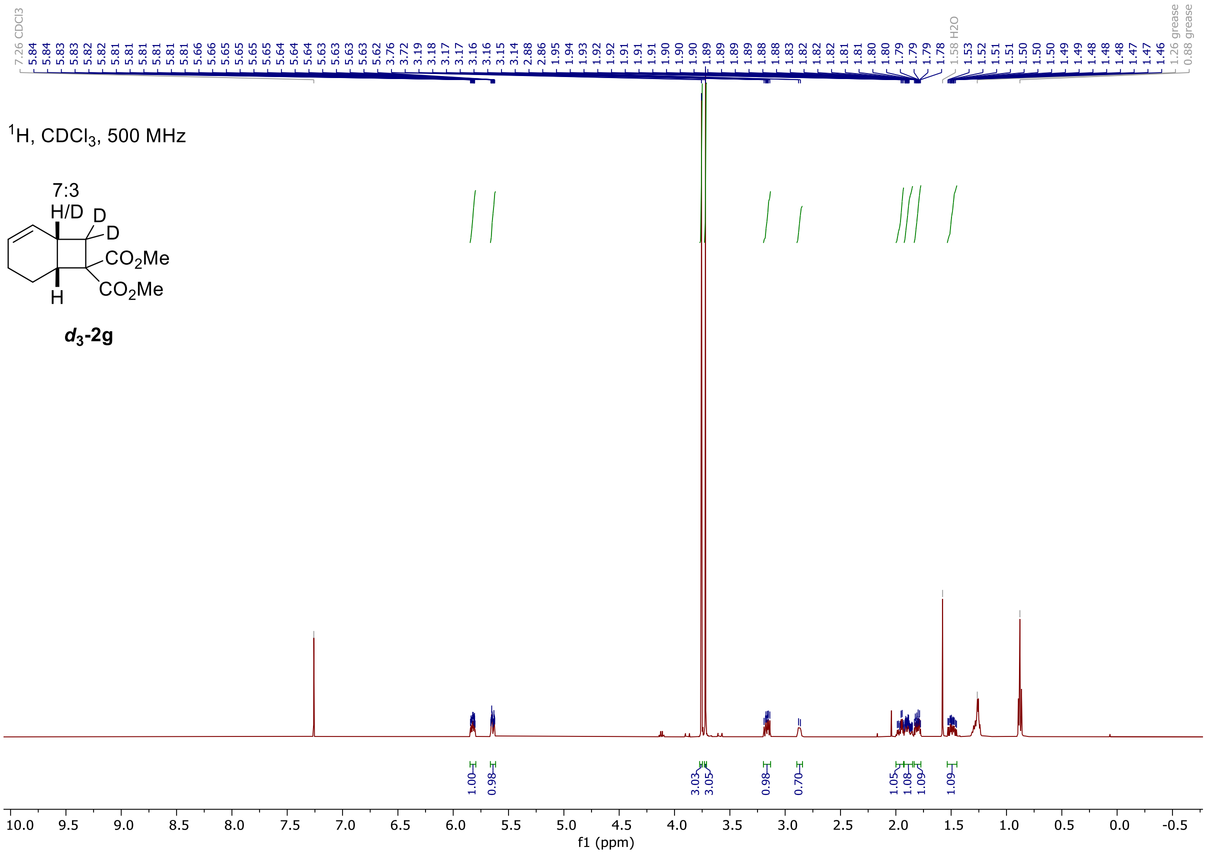
7.26 CDCl3
5.84 5.84 5.83 5.83 5.82 5.82 5.81 5.81 5.81 5.81 5.81 5.66 5.65 5.65 5.65 5.64 5.64 5.64 5.63 5.63 5.63 5.63 5.62 3.76 3.72 3.19 3.18 3.17 3.17 3.16 3.16 3.15 3.14 2.88 2.86 1.95 1.94 1.93 1.92 1.92 1.91 1.91 1.91 1.90 1.90 1.90 1.89 1.89 1.89 1.89 1.88 1.88 1.83 1.82 1.82 1.81 1.81 1.80 1.80 1.79 1.79 1.79 1.78
1.58 H2O
1.53 1.52 1.51 1.51 1.50 1.50 1.50 1.49 1.49 1.48 1.48 1.47 1.47 1.46
1.26 grease
0.88 grease
1H, CDCl3, 500 MHz
7:3
H/D
D
D
CO2Me
CO2Me
H
d3-2g
1.00
0.98
3.03
3.05
0.98
0.70
1.05
1.08
1.09
1.09
10.0 9.5 9.0 8.5 8.0 7.5 7.0 6.5 6.0 5.5 5.0 4.5 4.0 3.5 3.0 2.5 2.0 1.5 1.0 0.5 0.0 -0.5
f1 (ppm)

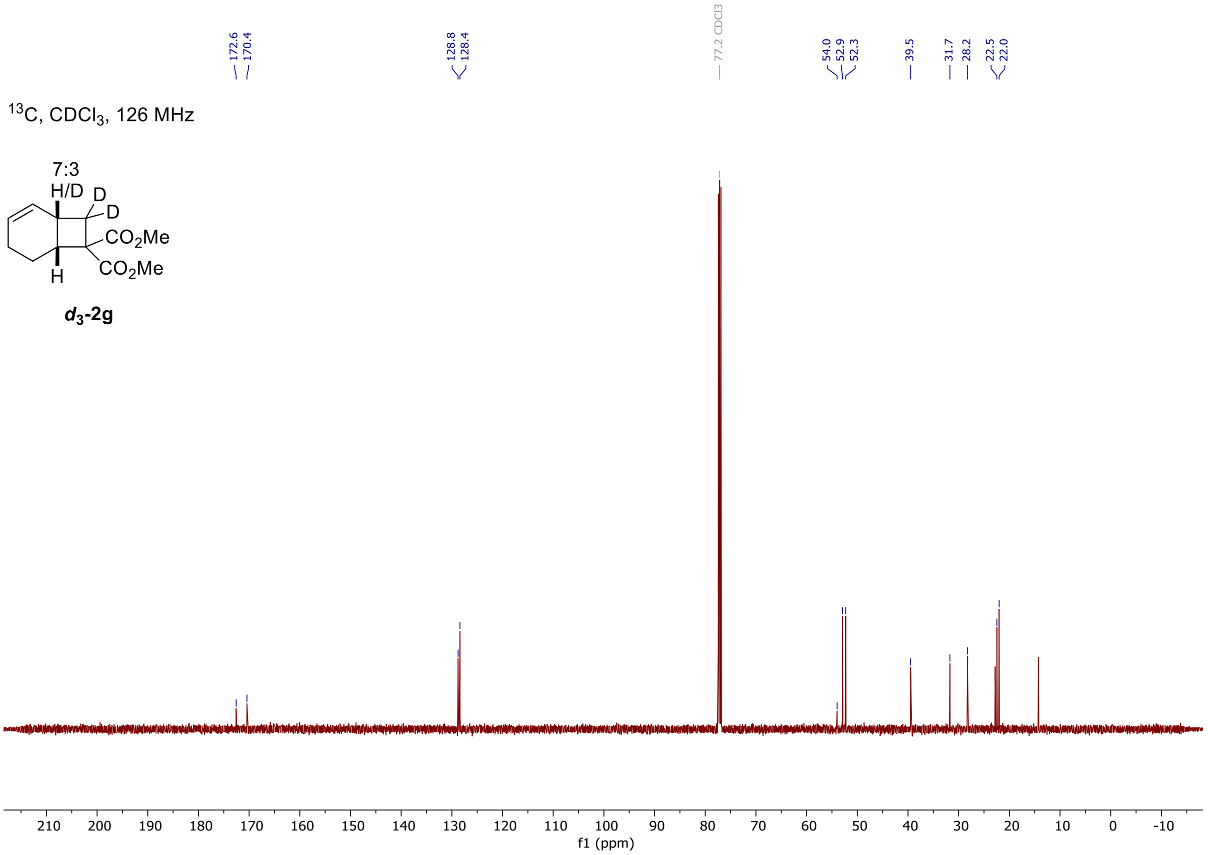
172.6
170.4
128.8
128.4
77.2 CDCl3
54.0
52.9
52.3
39.5
31.7
28.2
22.5
22.0
13C, CDCl3, 126 MHz
7:3
H/D
D
D
CO2Me
CO2Me
H
d3-2g
210 200 190 180 170 160 150 140 130 120 110 100 90 80 70 60 50 40 30 20 10 0 -10
f1 (ppm)

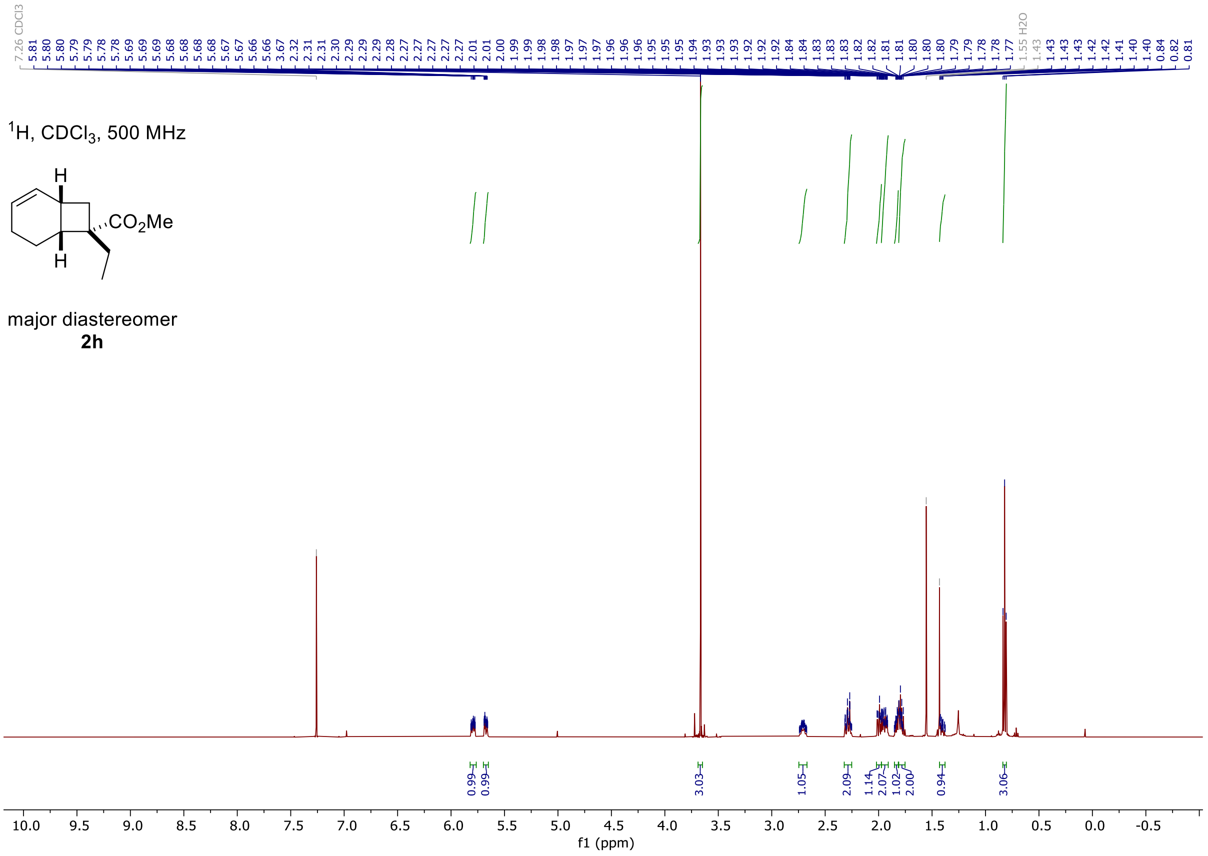

7.26 CDCl3
5.81 5.80 5.79 5.79 5.78 5.78 5.69 5.69 5.68 5.68 5.68 5.67 5.67 5.66 3.67 2.32 2.31 2.31 2.30 2.29 2.29 2.29 2.28 2.27 2.27 2.27 2.27 2.27 2.01 2.01 2.00 1.99 1.99 1.98 1.98 1.97 1.97 1.97 1.96 1.96 1.96 1.95 1.95 1.95 1.94 1.93 1.93 1.93 1.93 1.92 1.92 1.92 1.84 1.84 1.83 1.83 1.83 1.82 1.82 1.81 1.81 1.80 1.80 1.80 1.79 1.79 1.78 1.78 1.77
1.55 H2O
1.43 1.43 1.43 1.43 1.43 1.42 1.42 1.41 1.40 0.84 0.82 0.81
1H, CDCl3, 500 MHz
H
CO2Me
H
major diastereomer
2h
0.99 0.99 3.03 1.05 2.09 1.14 2.07 1.02 2.00 0.94 3.06
10.0 9.5 9.0 8.5 8.0 7.5 7.0 6.5 6.0 5.5 5.0 4.5 4.0 3.5 3.0 2.5 2.0 1.5 1.0 0.5 0.0 -0.5
f1 (ppm)

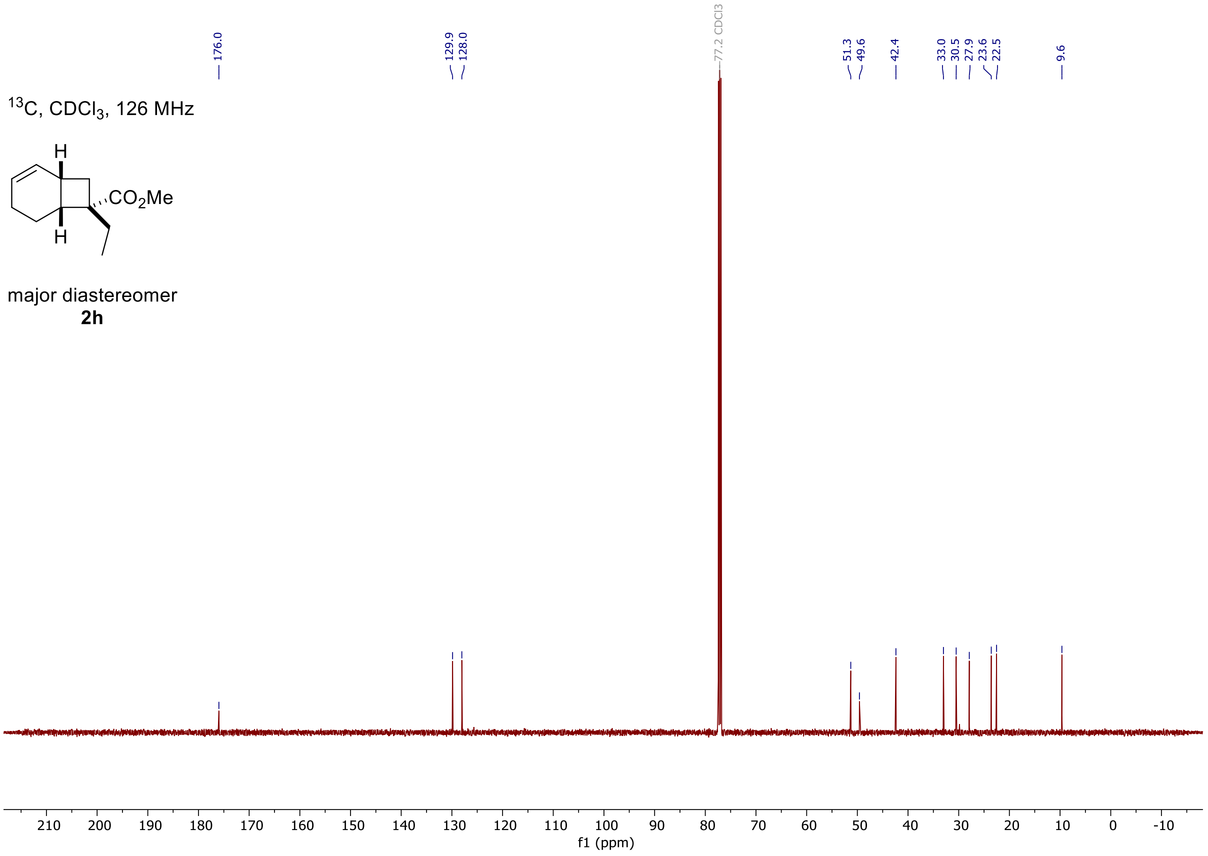

176.0
129.9 128.0
77.2 CDCl3
51.3 49.6 42.4 33.0 30.5 27.9 23.6 22.5 9.6
13C, CDCl3, 126 MHz
H
CO2Me
H
major diastereomer
2h
210 200 190 180 170 160 150 140 130 120 110 100 90 80 70 60 50 40 30 20 10 0 -10
f1 (ppm)

NOESY of **2h** (zoomed in).

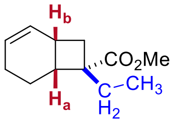

2h

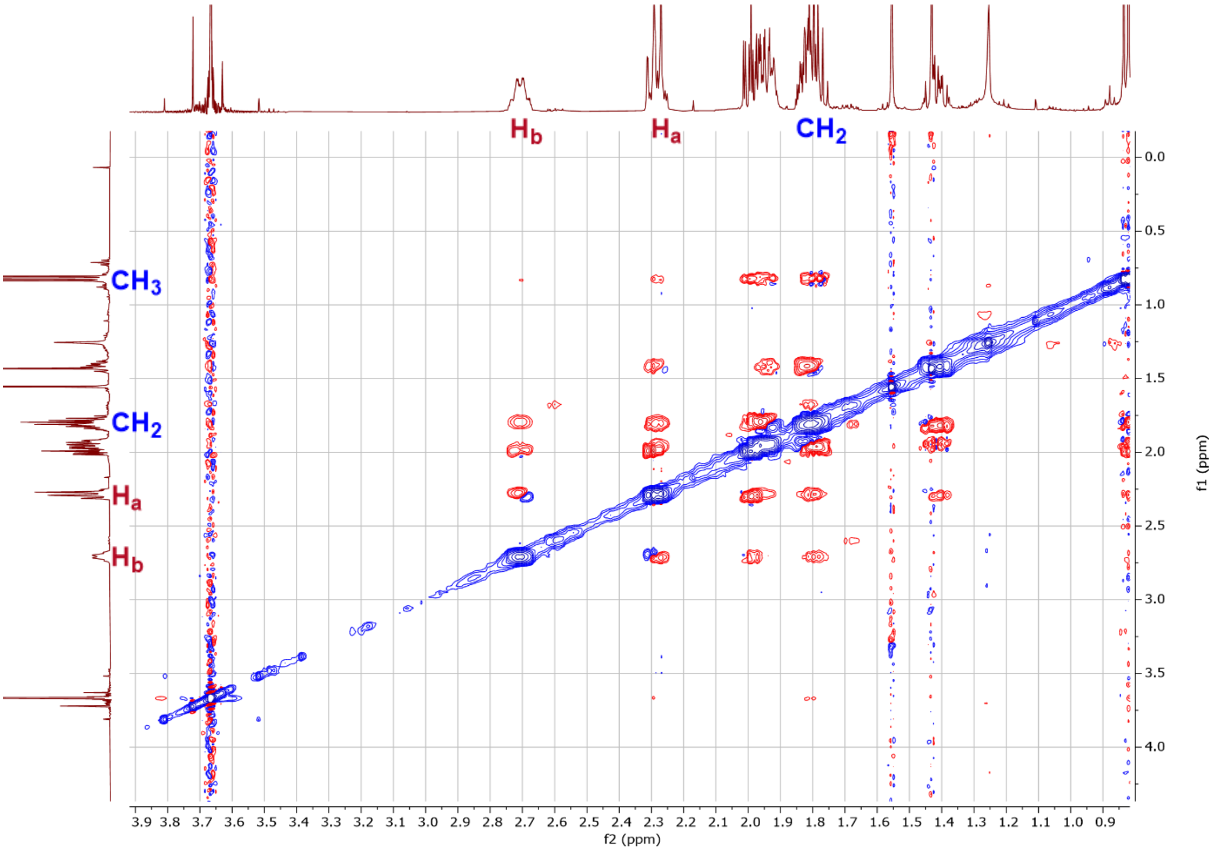

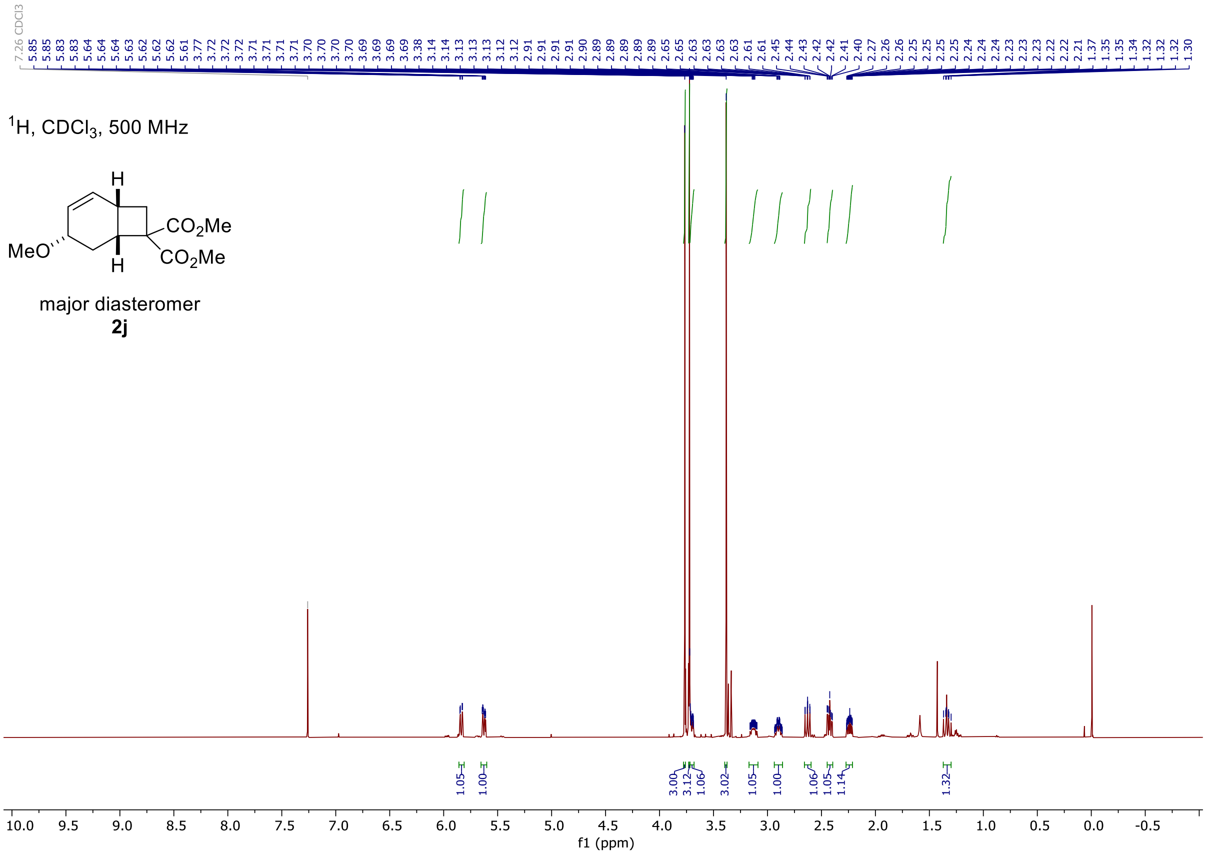
7.26 CDCl3
5.85 5.85 5.83 5.83 5.64 5.64 5.63 5.62 5.62 5.61 3.77 3.72 3.72 3.72 3.71 3.71 3.71 3.70 3.70 3.70 3.70 3.69 3.69 3.69 3.38 3.14 3.13 3.13 3.13 3.12 3.12 2.91 2.91 2.91 2.91 2.90 2.89 2.89 2.89 2.89 2.65 2.63 2.63 2.63 2.63 2.61 2.61 2.45 2.44 2.43 2.42 2.42 2.41 2.40 2.27 2.26 2.26 2.25 2.25 2.25 2.25 2.24 2.24 2.24 2.23 2.23 2.22 2.22 2.21 1.37 1.35 1.35 1.34 1.32 1.32 1.32 1.30
1H, CDCl3, 500 MHz
H
CO2Me
MeO
H
CO2Me
major diasteromer
2j
1.05
1.00
3.00
3.12
1.06
3.02
1.05
1.00
1.06
1.05
1.14
1.32
10.0 9.5 9.0 8.5 8.0 7.5 7.0 6.5 6.0 5.5 5.0 4.5 4.0 3.5 3.0 2.5 2.0 1.5 1.0 0.5 0.0 -0.5
f1 (ppm)

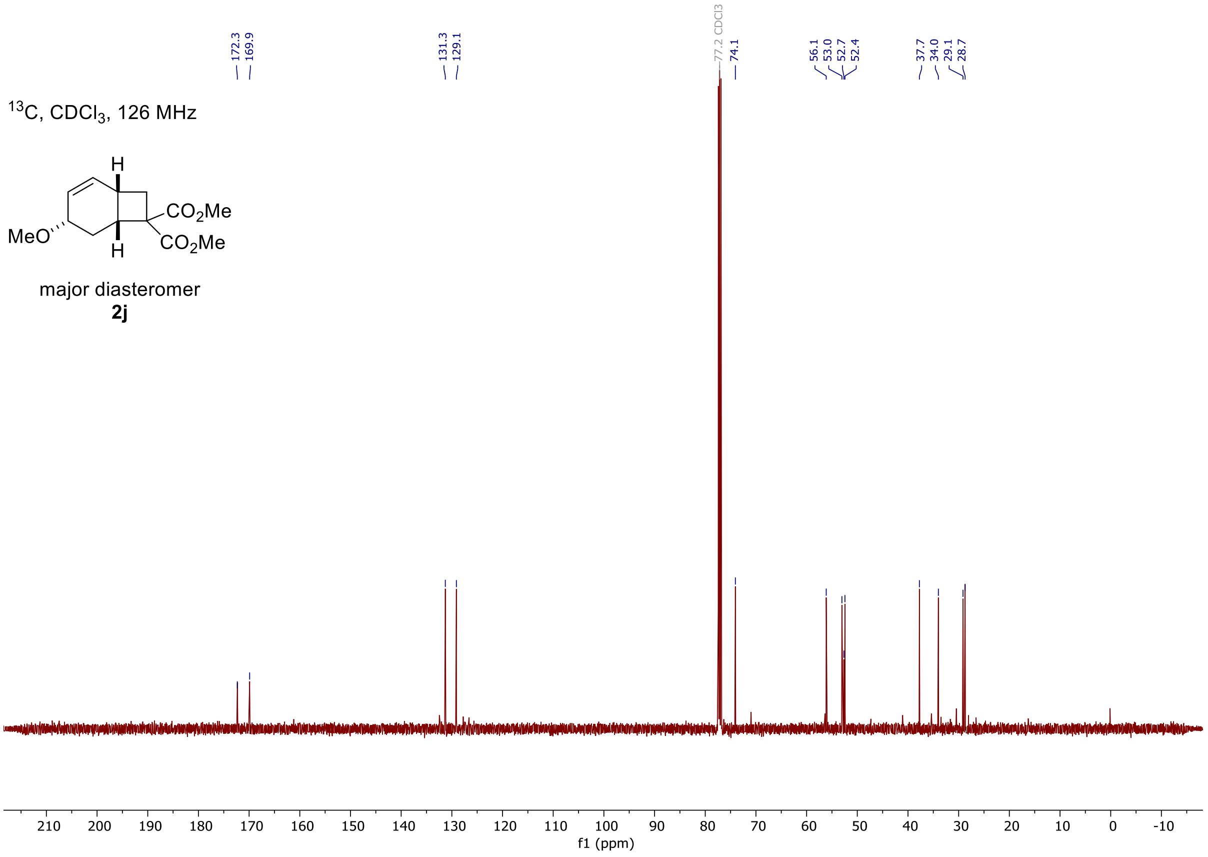
172.3
169.9
131.3
129.1
77.2 CDCl3
74.1
56.1
53.0
52.7
52.4
37.7
34.0
29.1
28.7
13C, CDCl3, 126 MHz
H
CO2Me
MeO
H
CO2Me
major diasteromer
2j
210 200 190 180 170 160 150 140 130 120 110 100 90 80 70 60 50 40 30 20 10 0 -10
f1 (ppm)

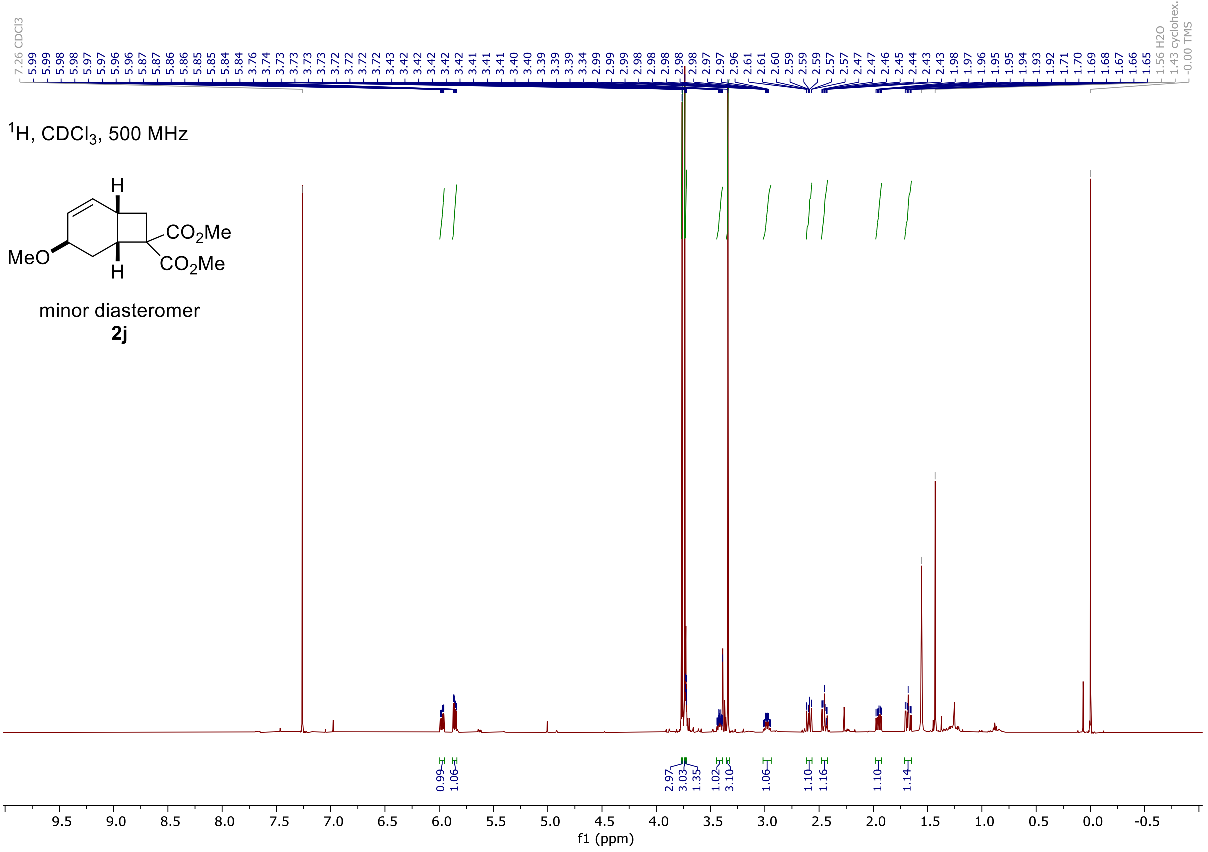

^{1}H, CDCl$_3$, 500 MHz
CO$_2$Me
CO$_2$Me
MeO
H
H
minor diasteromer
2j
7.26 CDCl3
1.56 H2O
1.43 cyclohex.
-0.00 TMS
0.99
1.06
2.97
3.03
1.35
1.02
3.10
1.06
1.10
1.16
1.10
1.14
f1 (ppm)

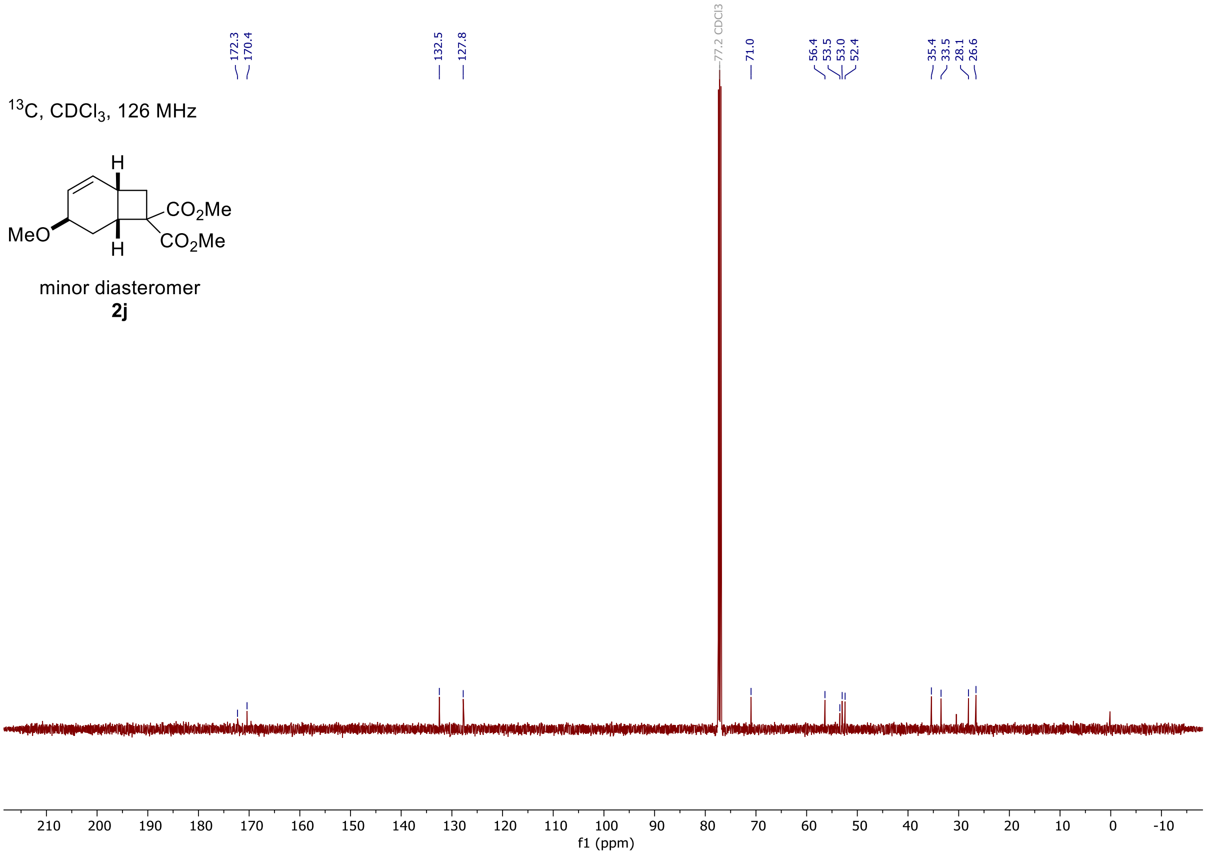

^{13}C, CDCl$_3$, 126 MHz
CO$_2$Me
CO$_2$Me
MeO
H
H
minor diasteromer
2j
172.3
170.4
132.5
127.8
77.2 CDCl3
71.0
56.4
53.5
53.0
52.4
35.4
33.5
28.1
26.6
f1 (ppm)

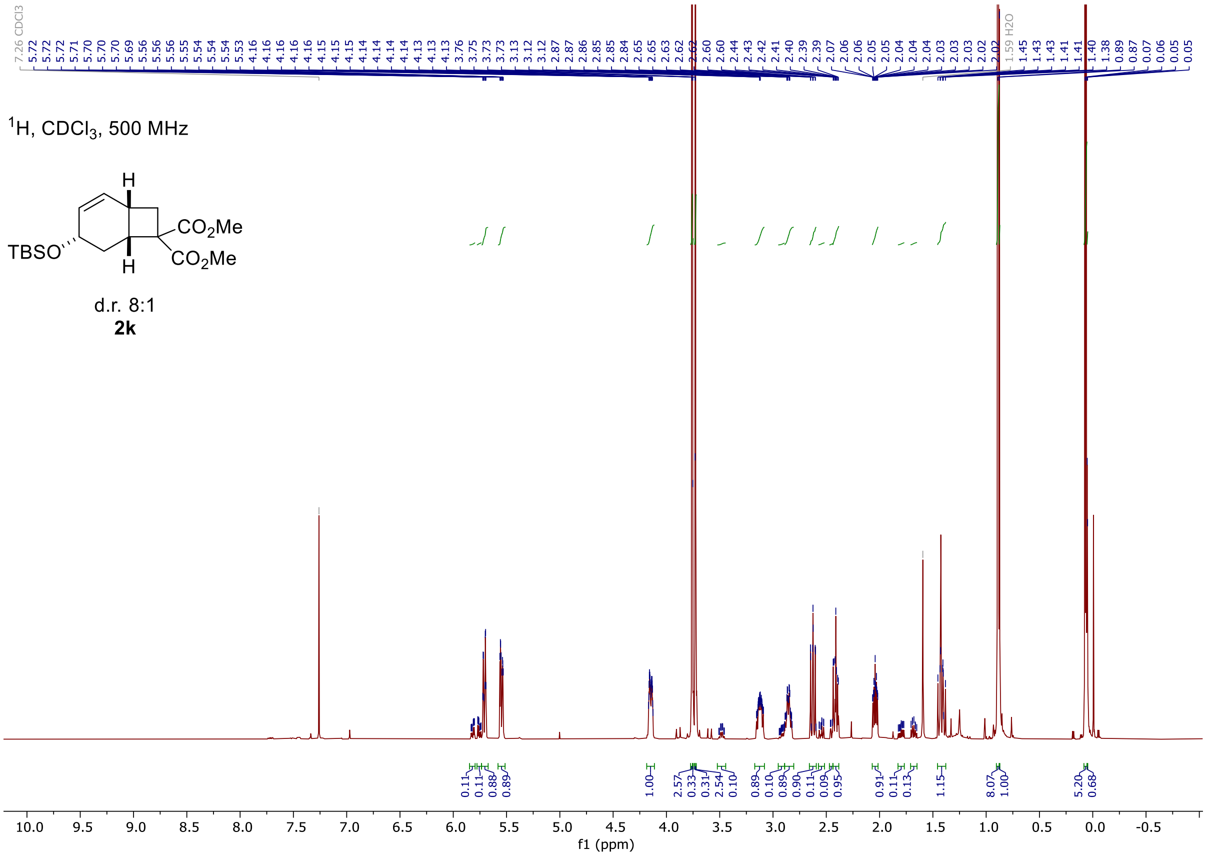
7.26 CDCl3
5.72 5.72 5.72 5.71 5.70 5.70 5.70 5.69 5.56 5.56 5.56 5.55 5.54 5.54 5.54 5.53 4.16 4.16 4.16 4.16 4.16 4.15 4.15 4.15 4.14 4.14 4.14 4.13 4.13 4.13 3.76 3.75 3.73 3.73 3.13 3.12 3.12 2.87 2.86 2.85 2.85 2.84 2.65 2.63 2.62 2.62 2.60 2.60 2.44 2.43 2.42 2.41 2.40 2.39 2.39 2.07 2.06 2.06 2.05 2.05 2.04 2.04 2.04 2.03 2.03 2.03 2.02 2.02
1.59 H2O
1.45 1.43 1.43 1.41 1.40 1.38 0.89 0.87 0.07 0.06 0.05 0.05
1H, CDCl3, 500 MHz
H
CO2Me
TBSO
H
CO2Me
d.r. 8:1
2k
0.11 0.11 0.88 0.89 1.00 2.57 0.33 0.31 2.54 0.10 0.89 0.10 0.89 0.90 0.11 0.09 0.95 0.91 0.11 0.13 1.15 8.07 1.00 5.20 0.68
10.0 9.5 9.0 8.5 8.0 7.5 7.0 6.5 6.0 5.5 5.0 4.5 4.0 3.5 3.0 2.5 2.0 1.5 1.0 0.5 0.0 -0.5
f1 (ppm)

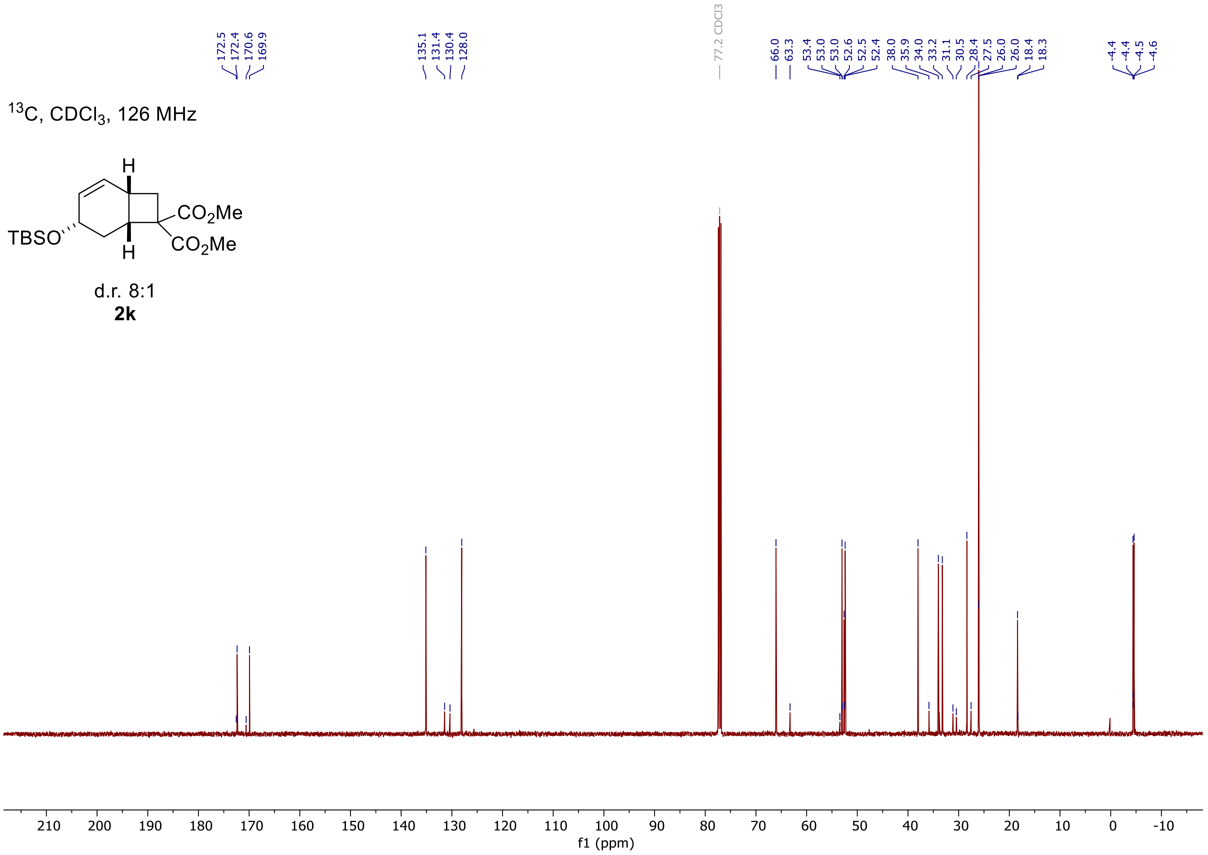
172.5 172.4 170.6 169.9
135.1 131.4 130.4 128.0
77.2 CDCl3
66.0 63.3 53.4 53.0 53.0 52.6 52.5 52.4 38.0 35.9 34.0 33.2 31.1 30.5 28.4 27.5 26.0 26.0 18.4 18.3
-4.4 -4.4 -4.5 -4.6
13C, CDCl3, 126 MHz
H
CO2Me
TBSO
H
CO2Me
d.r. 8:1
2k
210 200 190 180 170 160 150 140 130 120 110 100 90 80 70 60 50 40 30 20 10 0 -10
f1 (ppm)

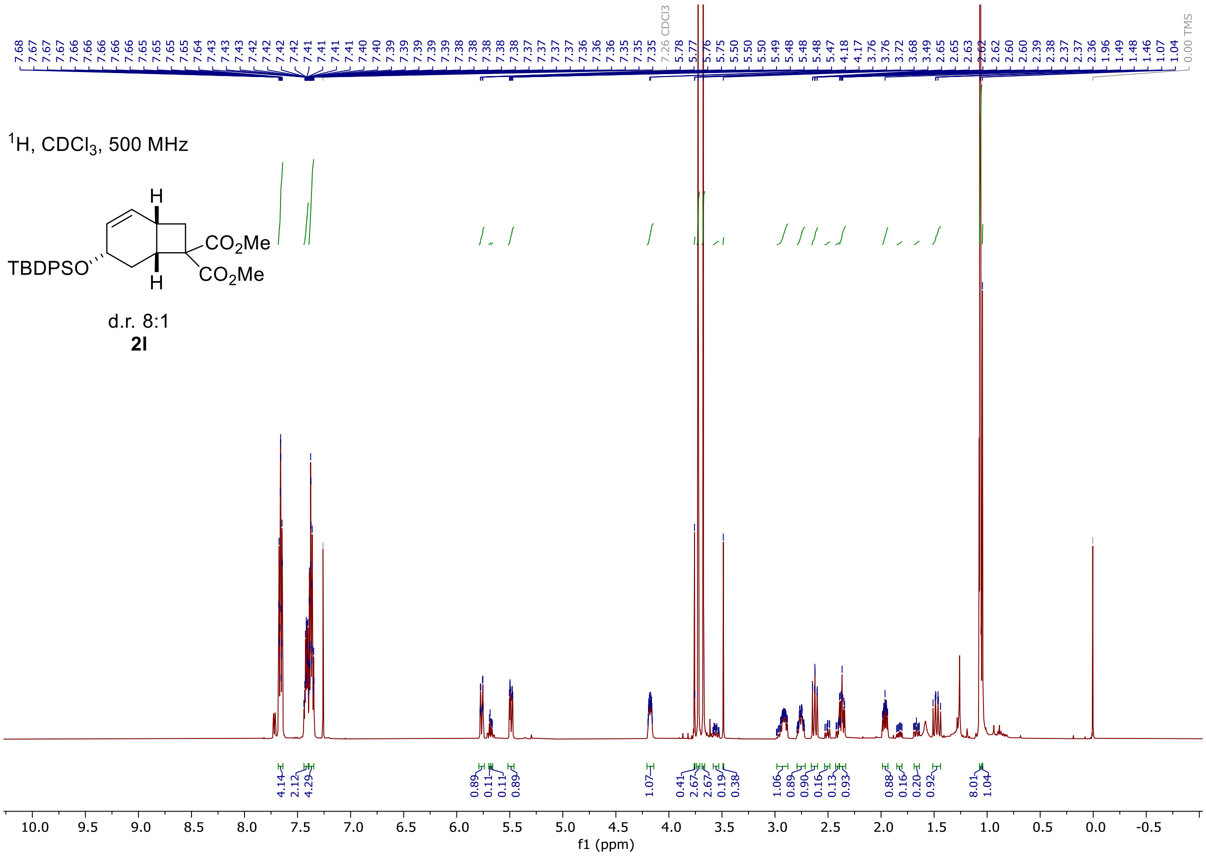

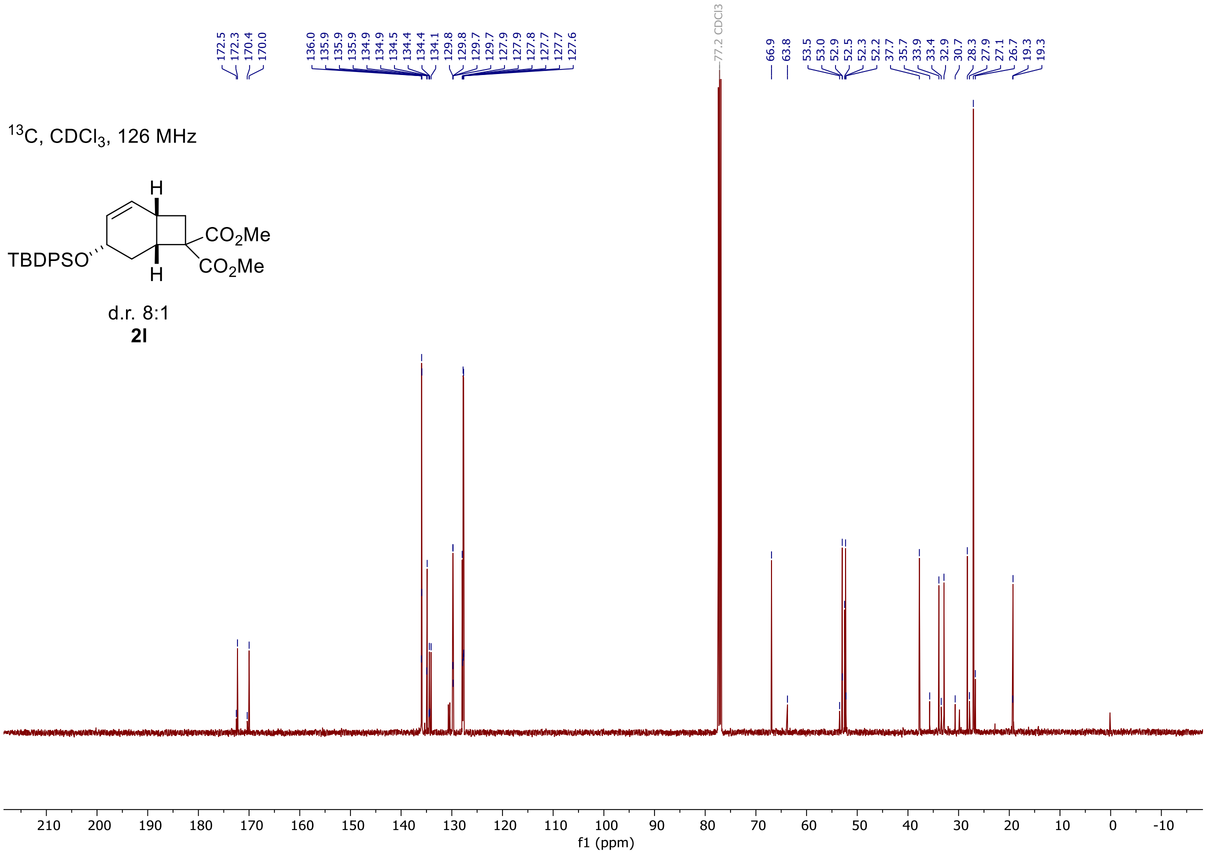

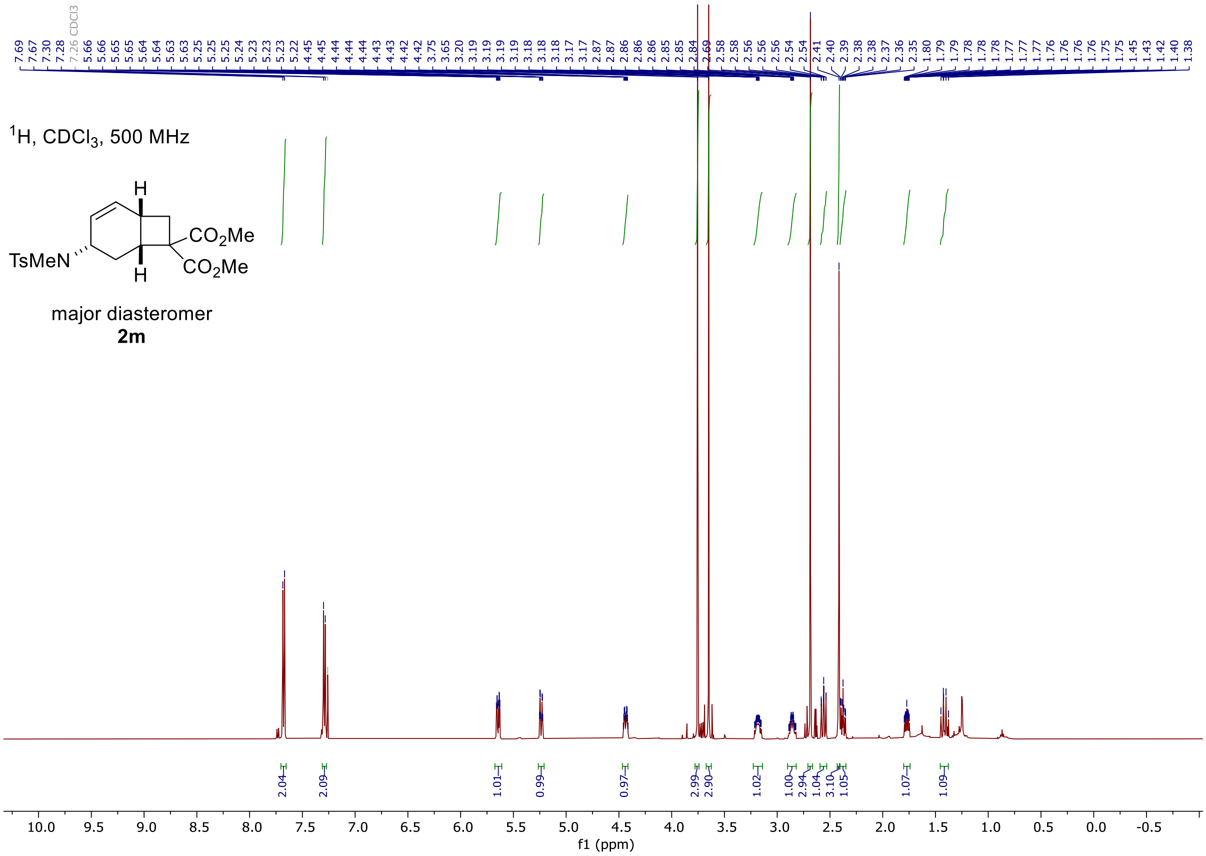

7.69 7.67 7.30 7.28 7.26 CDCl3 5.66 5.66 5.65 5.65 5.64 5.64 5.63 5.63 5.25 5.25 5.25 5.24 5.23 5.23 5.23 5.22 4.45 4.45 4.44 4.44 4.43 4.43 4.42 4.42 3.75 3.65 3.20 3.19 3.19 3.19 3.18 3.18 3.18 3.17 3.17 2.87 2.86 2.86 2.86 2.85 2.85 2.84 2.69 2.58 2.58 2.56 2.56 2.56 2.54 2.54 2.41 2.40 2.39 2.38 2.38 2.38 2.37 2.36 2.35 1.80 1.79 1.79 1.78 1.78 1.78 1.77 1.77 1.77 1.76 1.76 1.76 1.75 1.75 1.45 1.43 1.42 1.40 1.38
^{1}H, CDCl$_3$, 500 MHz
TsMeN
H
H
CO$_2$Me
CO$_2$Me
major diasteromer
2m
2.04 2.09 1.01 0.99 0.97 2.99 2.90 1.02 1.00 2.94 1.04 3.10 1.05 1.07 1.09
10.0 9.5 9.0 8.5 8.0 7.5 7.0 6.5 6.0 5.5 5.0 4.5 4.0 3.5 3.0 2.5 2.0 1.5 1.0 0.5 0.0 -0.5
f1 (ppm)

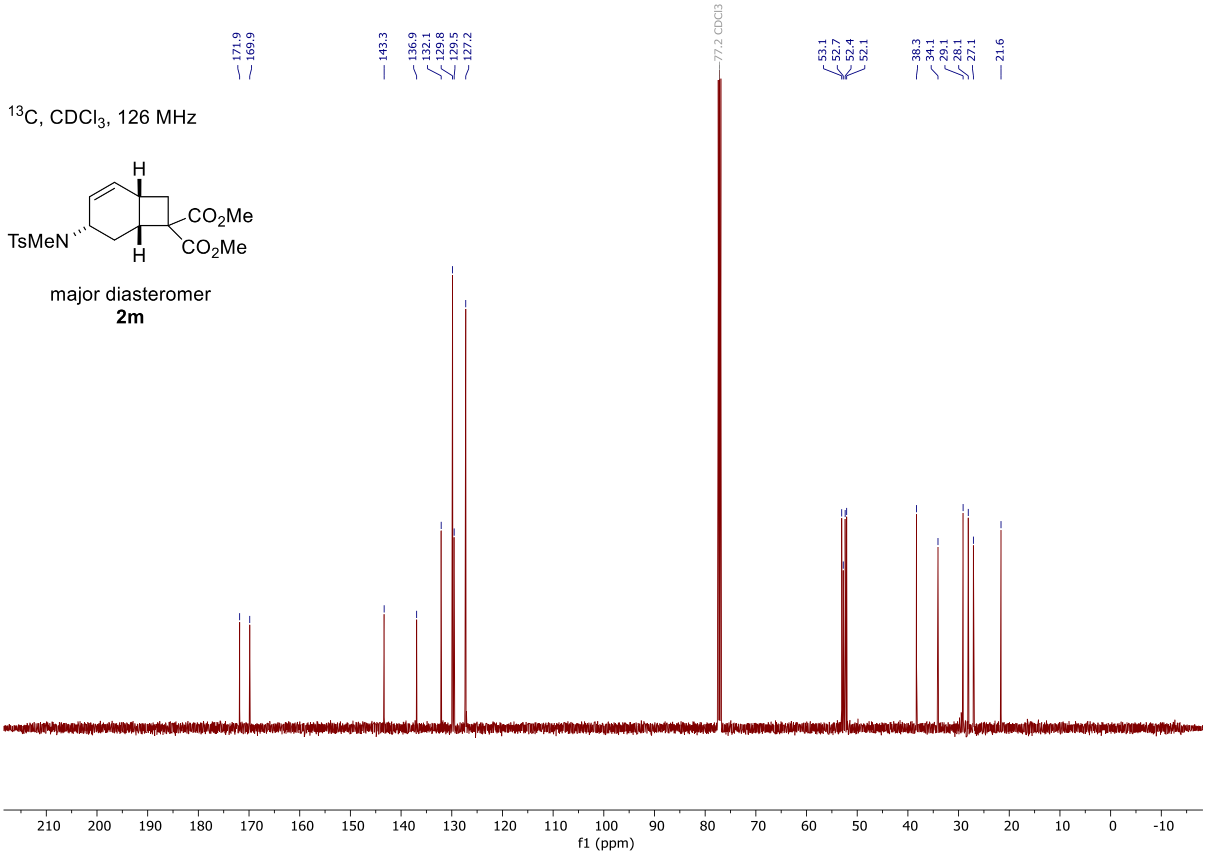

171.9 169.9 143.3 136.9 132.1 129.8 129.5 127.2 77.2 CDCl3 53.1 52.7 52.4 52.1 38.3 34.1 29.1 28.1 27.1 21.6
^{13}C, CDCl$_3$, 126 MHz
TsMeN
H
H
CO$_2$Me
CO$_2$Me
major diasteromer
2m
210 200 190 180 170 160 150 140 130 120 110 100 90 80 70 60 50 40 30 20 10 0 -10
f1 (ppm)

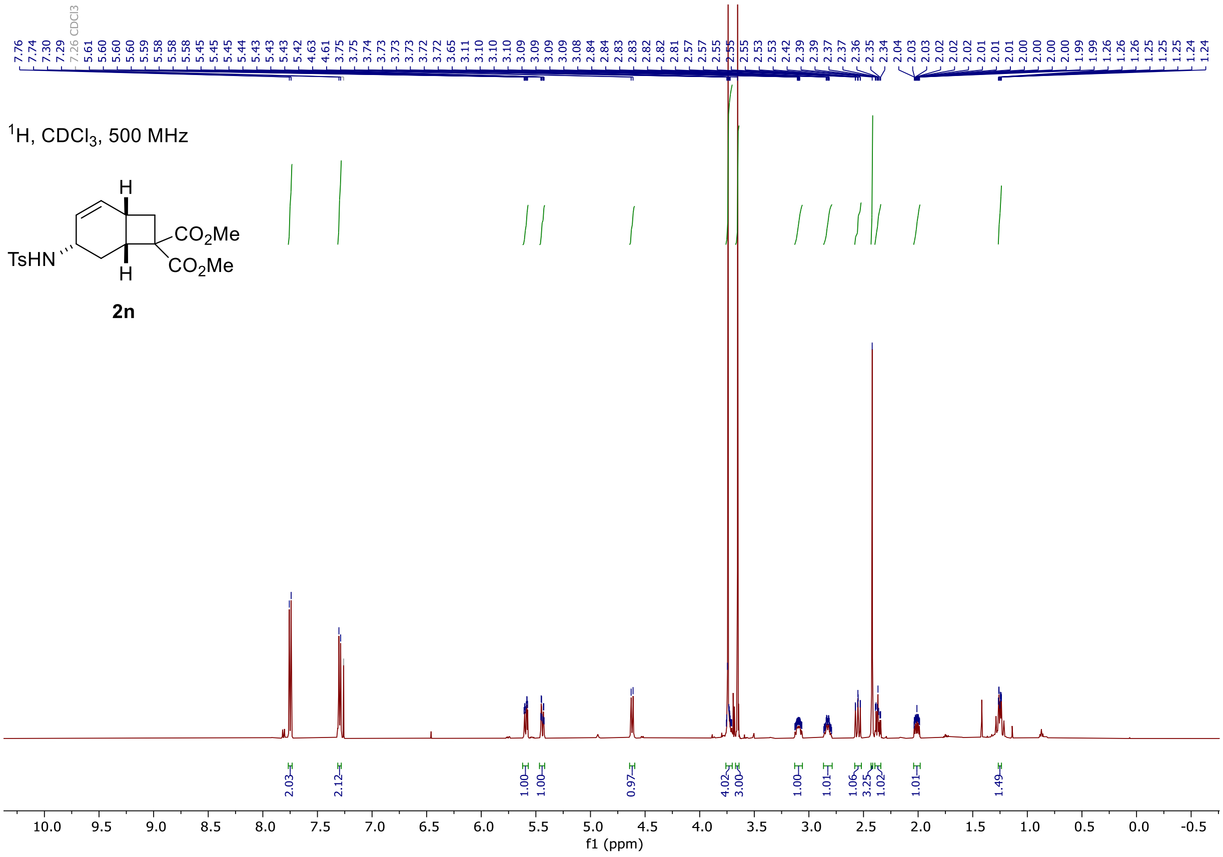
7.76 7.74 7.30 7.29 7.26 CDCl3 5.61 5.60 5.60 5.60 5.59 5.58 5.58 5.58 5.45 5.45 5.45 5.44 5.43 5.43 5.43 5.42 4.63 4.61 3.75 3.75 3.74 3.73 3.73 3.73 3.72 3.65 3.11 3.10 3.10 3.09 3.09 3.09 3.08 2.84 2.84 2.83 2.82 2.82 2.81 2.57 2.55 2.55 2.55 2.53 2.53 2.42 2.39 2.39 2.37 2.37 2.36 2.35 2.34 2.04 2.03 2.03 2.02 2.02 2.02 2.01 2.01 2.00 2.00 2.00 1.99 1.26 1.26 1.25 1.25 1.24 1.24
1H, CDCl3, 500 MHz
H
CO2Me
TsHN
H
CO2Me
2n
2.03 2.12 1.00 1.00 0.97 4.02 3.00 1.00 1.01 1.06 3.25 1.02 1.01 1.49
10.0 9.5 9.0 8.5 8.0 7.5 7.0 6.5 6.0 5.5 5.0 4.5 4.0 3.5 3.0 2.5 2.0 1.5 1.0 0.5 0.0 -0.5
f1 (ppm)

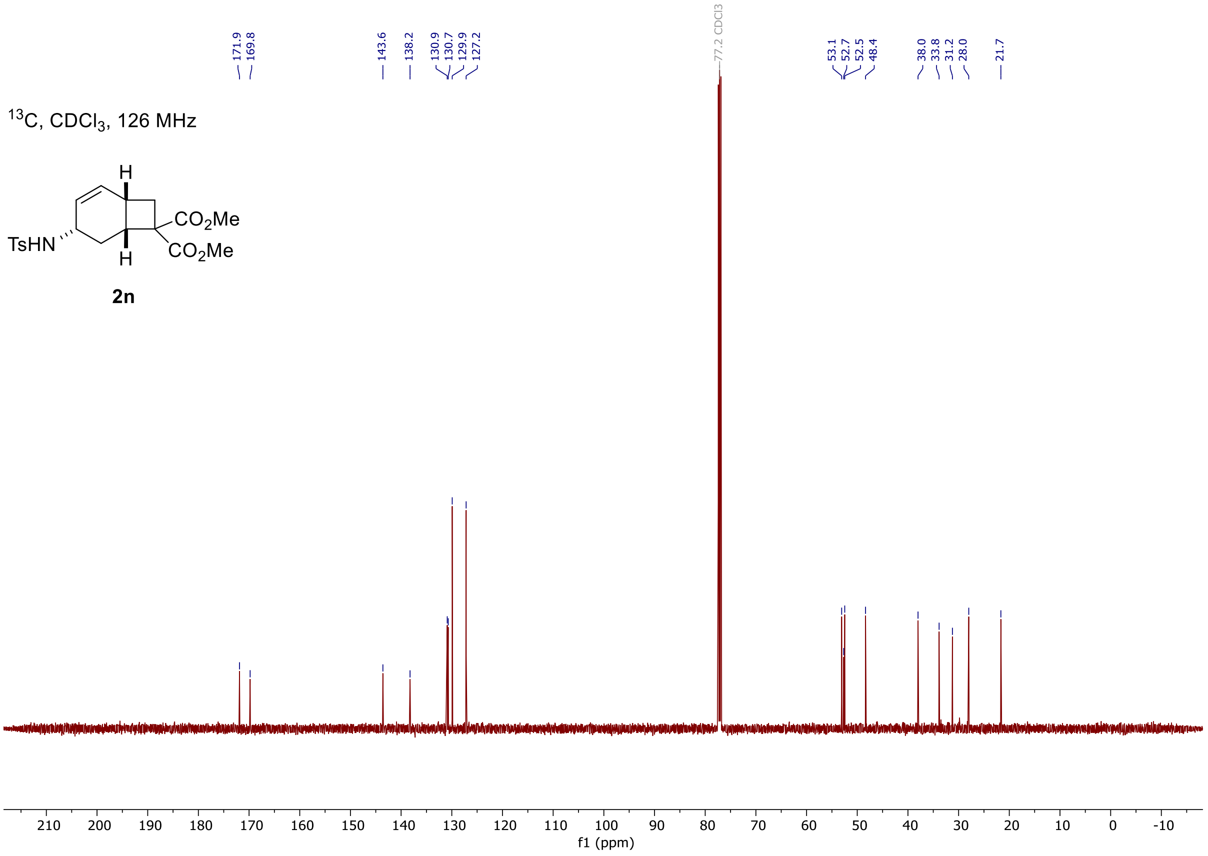
171.9 169.8 143.6 138.2 130.9 130.7 129.9 127.2 77.2 CDCl3 53.1 52.7 52.5 48.4 38.0 33.8 31.2 28.0 21.7
13C, CDCl3, 126 MHz
H
CO2Me
TsHN
H
CO2Me
2n
210 200 190 180 170 160 150 140 130 120 110 100 90 80 70 60 50 40 30 20 10 0 -10
f1 (ppm)

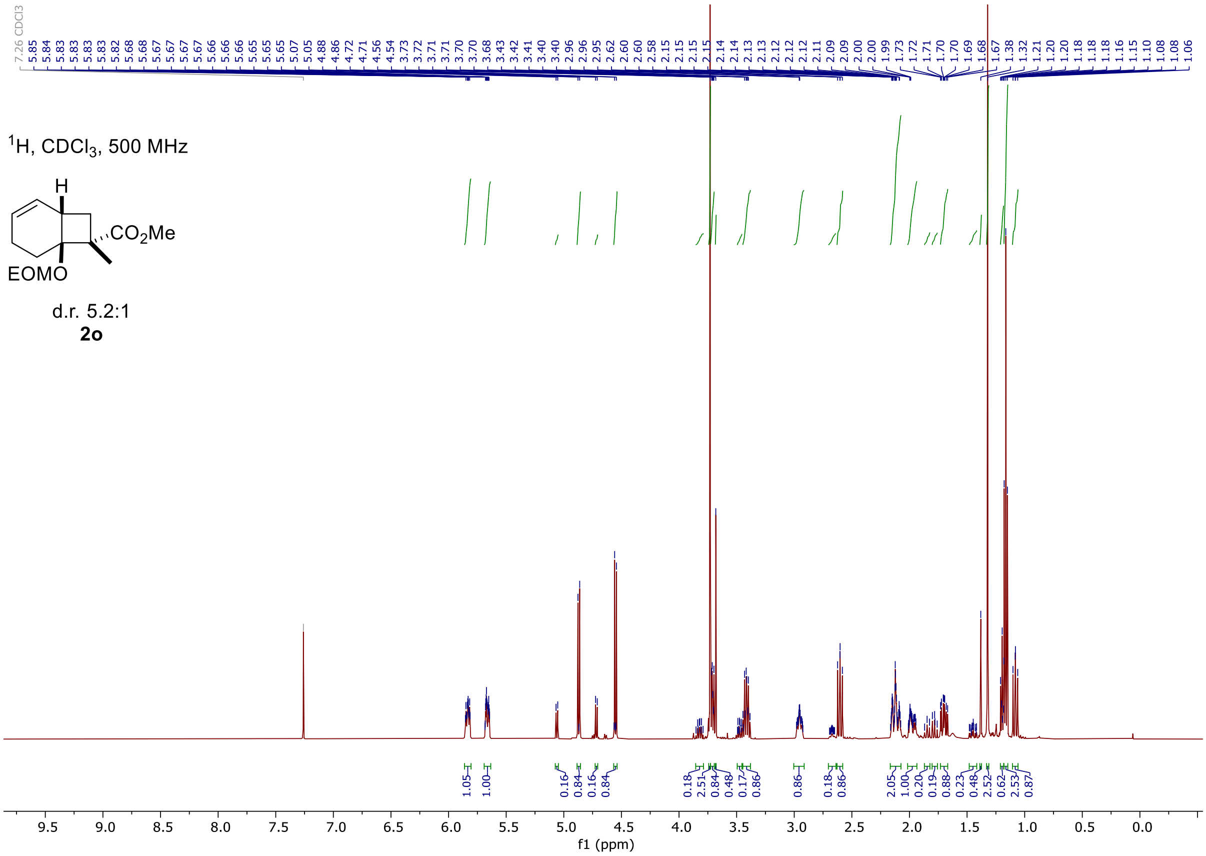
7.26 CDCl3
5.85 5.84 5.83 5.83 5.83 5.82 5.68 5.67 5.67 5.67 5.67 5.66 5.66 5.65 5.65 5.07 5.05 4.88 4.86 4.72 4.71 4.56 4.54 3.73 3.72 3.71 3.71 3.70 3.70 3.68 3.43 3.42 3.41 3.40 2.96 2.95 2.62 2.60 2.60 2.58 2.15 2.15 2.15 2.15 2.14 2.14 2.13 2.13 2.12 2.12 2.12 2.11 2.09 2.09 2.00 2.00 1.99 1.73 1.72 1.71 1.70 1.70 1.69 1.68 1.67 1.38 1.32 1.21 1.20 1.20 1.18 1.18 1.18 1.16 1.15 1.10 1.08 1.06
1H, CDCl3, 500 MHz
H
CO2Me
EOMO
d.r. 5.2:1
2o
1.05 1.00 0.16 0.84 0.16 0.84 0.18 2.51 0.84 0.48 0.17 0.86 0.86 0.18 0.86 2.05 1.00 0.20 0.19 0.88 0.23 0.48 2.52 0.62 2.53 0.87
9.5 9.0 8.5 8.0 7.5 7.0 6.5 6.0 5.5 5.0 4.5 4.0 3.5 3.0 2.5 2.0 1.5 1.0 0.5 0.0
f1 (ppm)

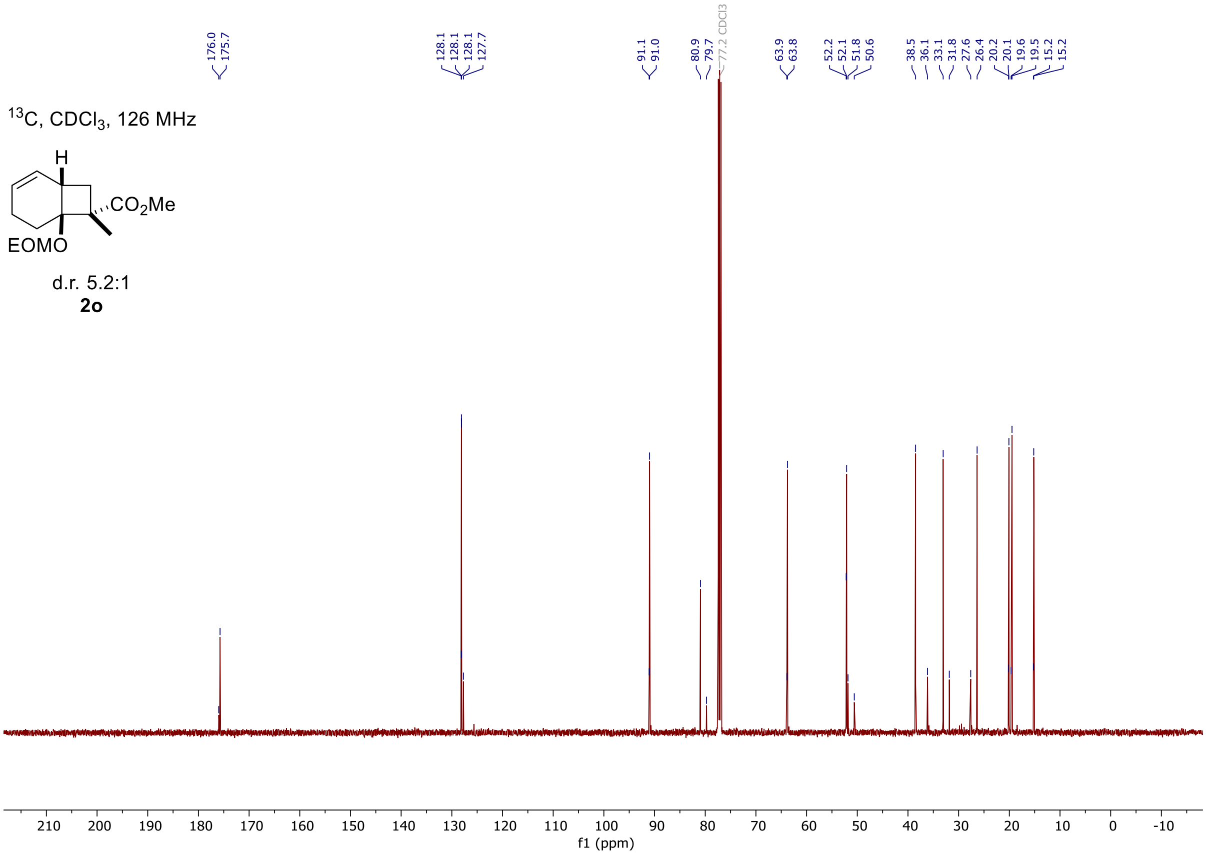
176.0 175.7 128.1 128.1 128.1 127.7 91.1 91.0 80.9 79.7 77.2 CDCl3 63.9 63.8 52.2 52.1 51.8 50.6 38.5 36.1 33.1 31.8 27.6 26.4 20.2 20.1 19.6 19.5 15.2 15.2
13C, CDCl3, 126 MHz
H
CO2Me
EOMO
d.r. 5.2:1
2o
210 200 190 180 170 160 150 140 130 120 110 100 90 80 70 60 50 40 30 20 10 0 -10
f1 (ppm)

NOESY of **2o** (zoomed in).

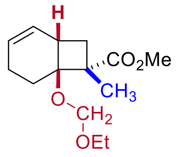

2o

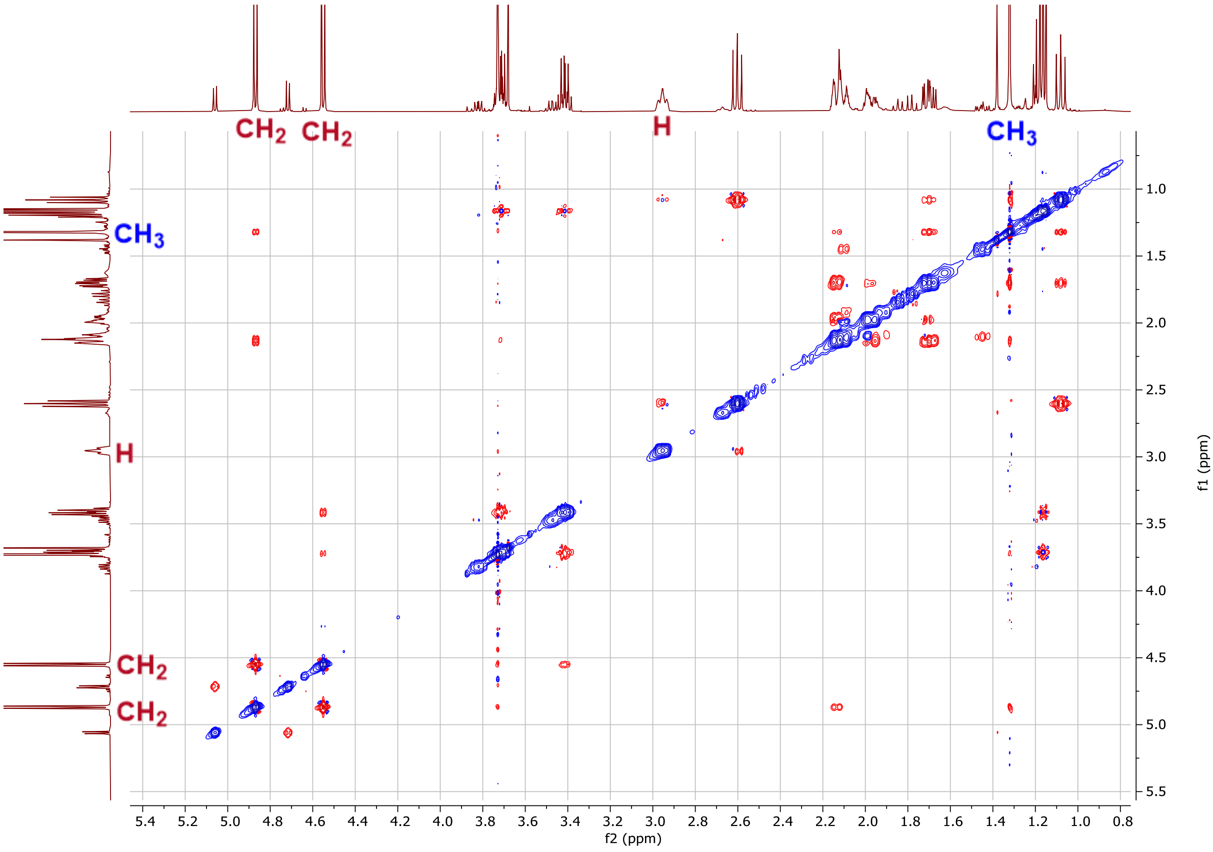

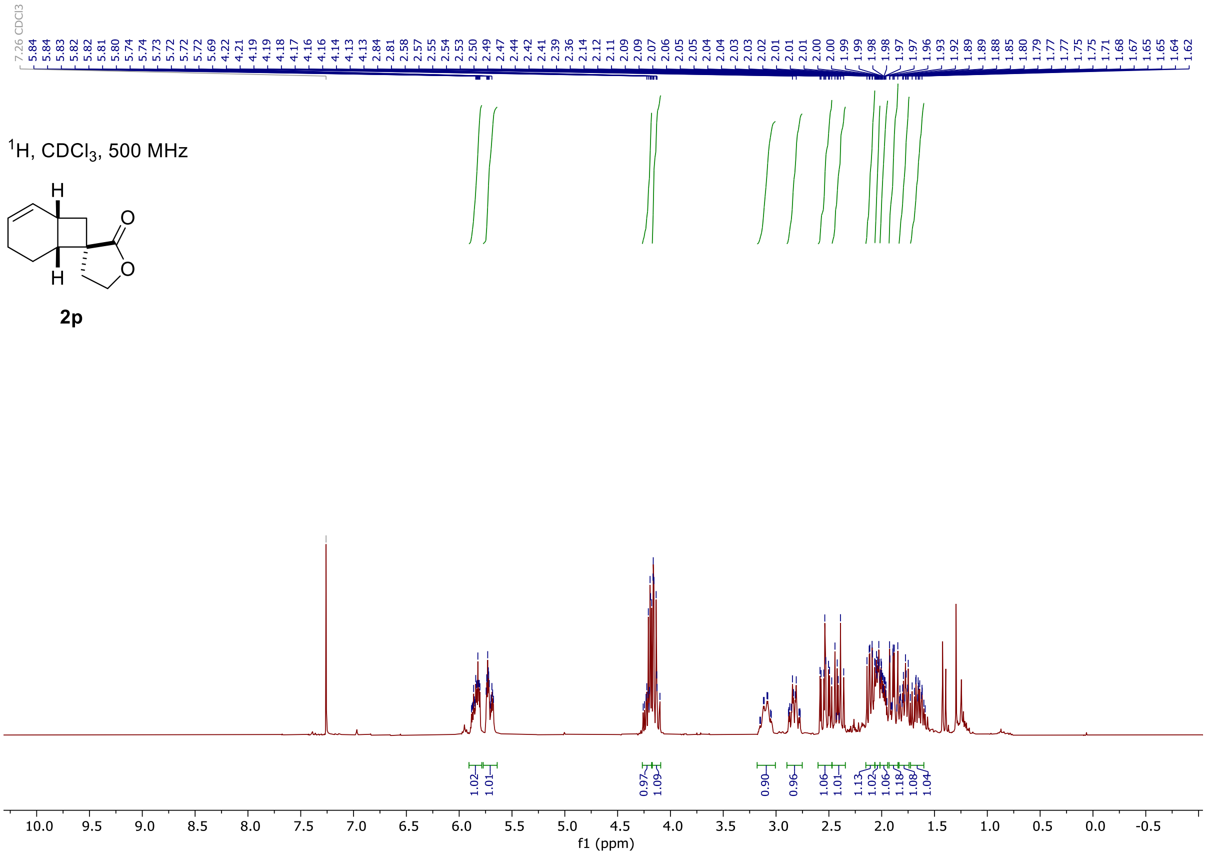
7.26 CDCl3
5.84 5.84 5.83 5.82 5.82 5.81 5.80 5.74 5.74 5.73 5.72 5.72 5.72 5.69 4.22 4.21 4.19 4.19 4.18 4.17 4.16 4.16 4.14 4.13 4.13 2.84 2.81 2.58 2.57 2.55 2.54 2.53 2.50 2.49 2.47 2.44 2.42 2.41 2.39 2.36 2.14 2.12 2.11 2.09 2.07 2.06 2.05 2.05 2.04 2.04 2.03 2.03 2.02 2.01 2.01 2.01 2.00 2.00 1.99 1.99 1.98 1.98 1.97 1.97 1.96 1.93 1.92 1.89 1.89 1.88 1.85 1.80 1.79 1.77 1.77 1.75 1.71 1.68 1.67 1.65 1.65 1.64 1.62
¹H, CDCl₃, 500 MHz
H
O
O
H
2p
1.02 1.01 0.97 1.09 0.90 0.96 1.06 1.01 1.13 1.02 1.06 1.18 1.08 1.04
10.0 9.5 9.0 8.5 8.0 7.5 7.0 6.5 6.0 5.5 5.0 4.5 4.0 3.5 3.0 2.5 2.0 1.5 1.0 0.5 0.0 -0.5
f1 (ppm)

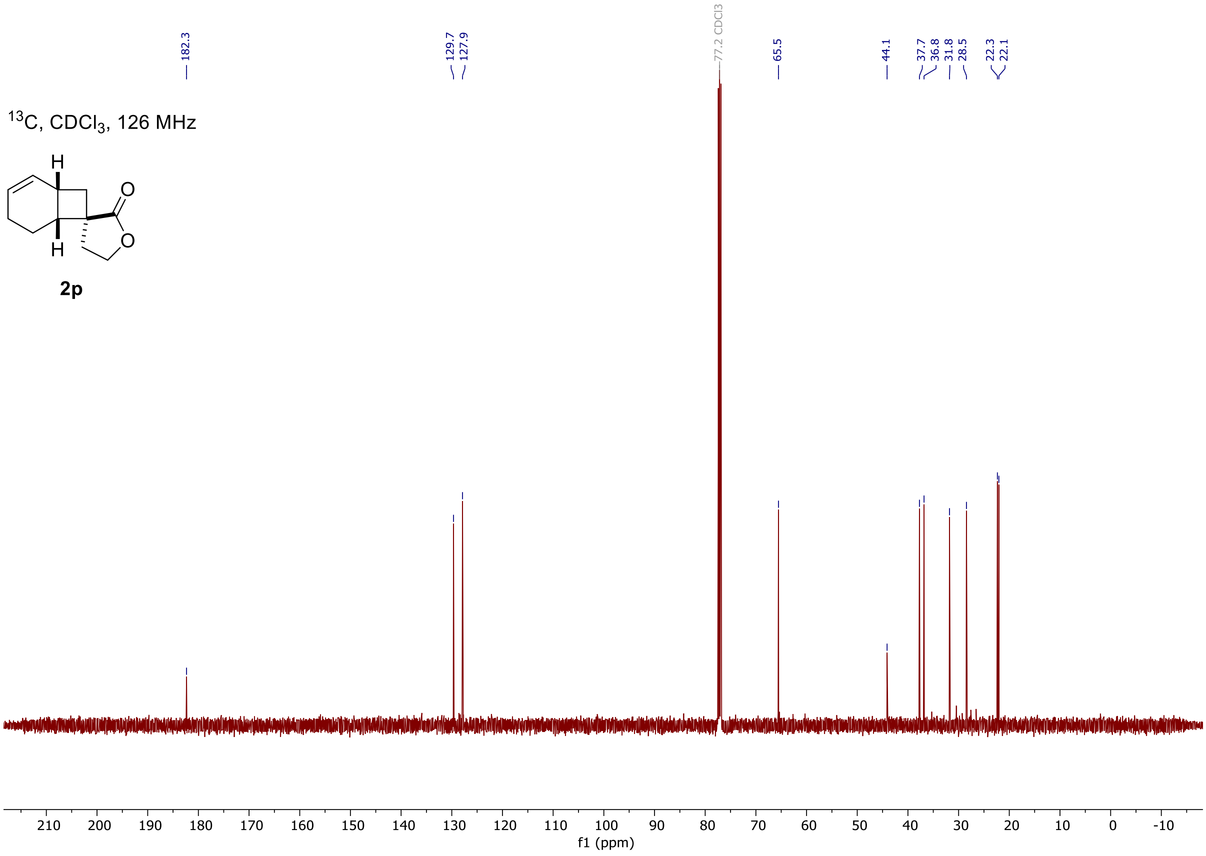
182.3
129.7 127.9
77.2 CDCl3
65.5
44.1
37.7 36.8 31.8 28.5
22.3 22.1
¹³C, CDCl₃, 126 MHz
H
O
O
H
2p
210 200 190 180 170 160 150 140 130 120 110 100 90 80 70 60 50 40 30 20 10 0 -10
f1 (ppm)

NOESY of **2p** (zoomed in).

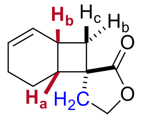

2p

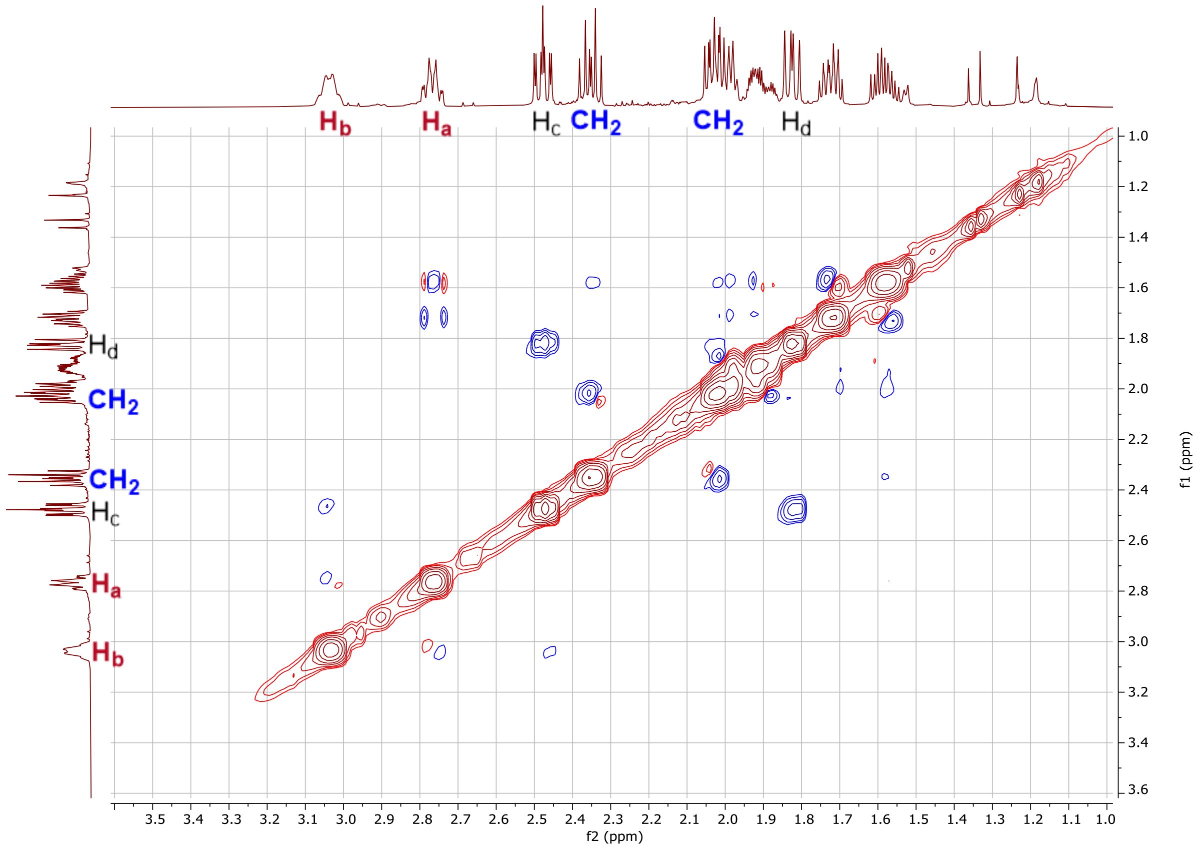

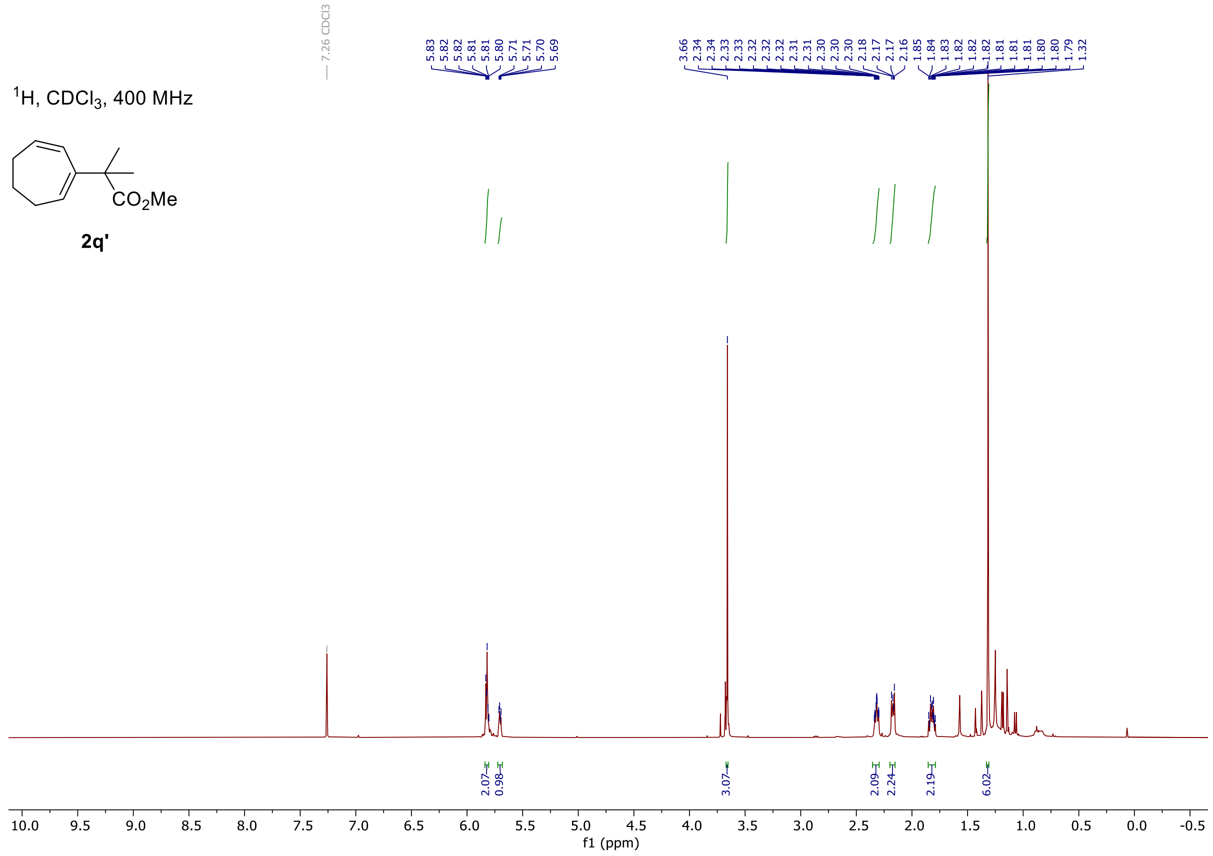
^{1}H, $CDCl_3$, 400 MHz
CO_2Me
2q'
7.26 CDCl3
5.83
5.82
5.82
5.81
5.81
5.80
5.71
5.71
5.70
5.69
3.66
2.34
2.34
2.33
2.33
2.32
2.32
2.32
2.31
2.31
2.30
2.30
2.30
2.18
2.17
2.17
2.16
1.85
1.84
1.83
1.82
1.82
1.82
1.81
1.81
1.81
1.80
1.79
1.32
2.07
0.98
3.07
2.09
2.24
2.19
6.02
f1 (ppm)

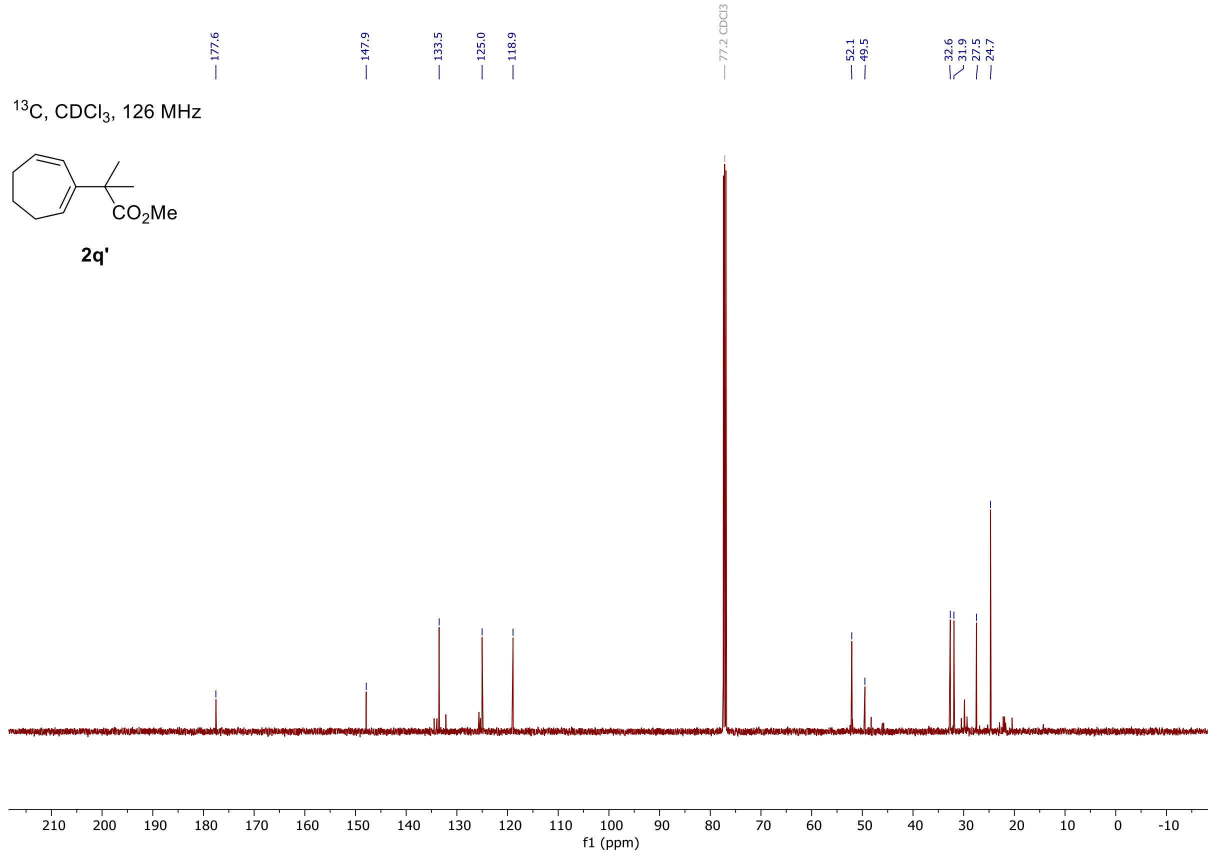
^{13}C, $CDCl_3$, 126 MHz
CO_2Me
2q'
177.6
147.9
133.5
125.0
118.9
77.2 CDCl3
52.1
49.5
32.6
31.9
27.5
24.7
f1 (ppm)

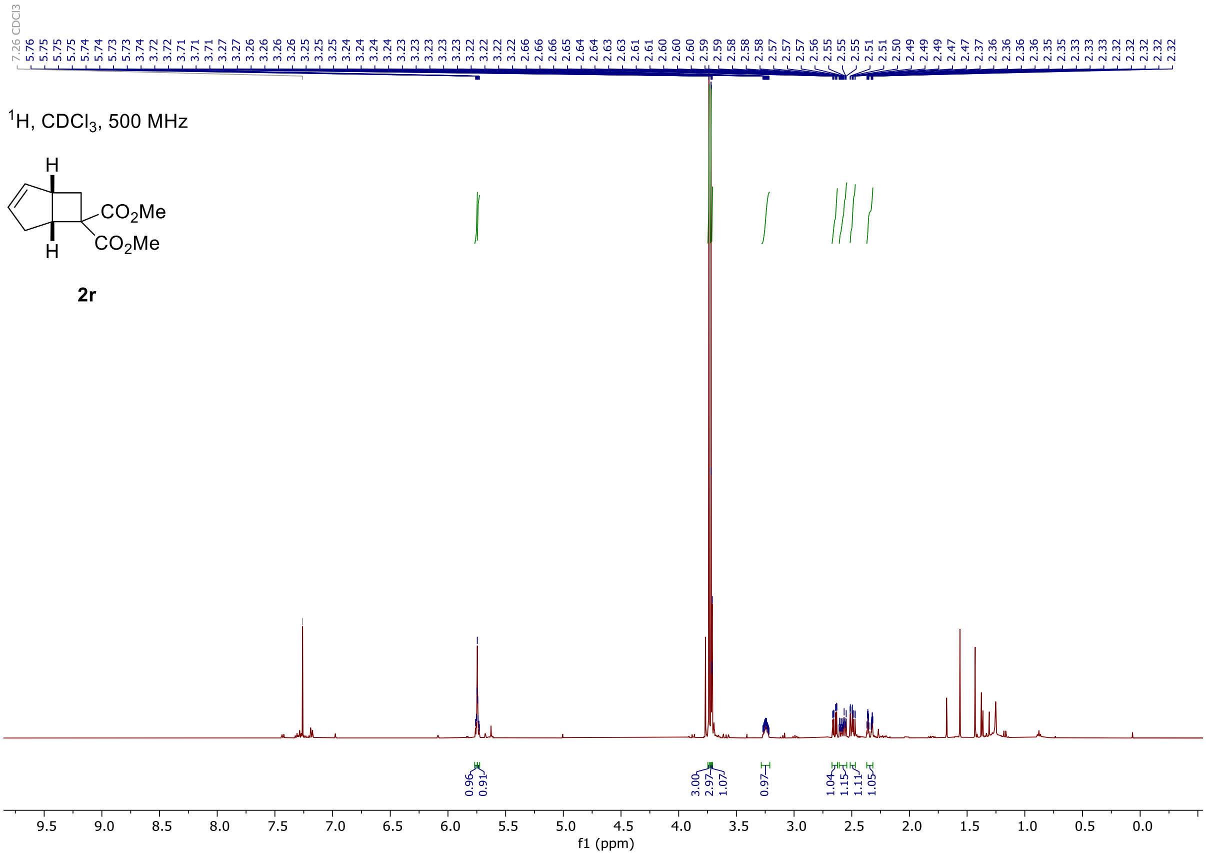
1H, CDCl3, 500 MHz
H
CO2Me
CO2Me
H
2r
0.96
0.91
3.00
2.97
1.07
0.97
1.04
1.15
1.11
1.05
f1 (ppm)

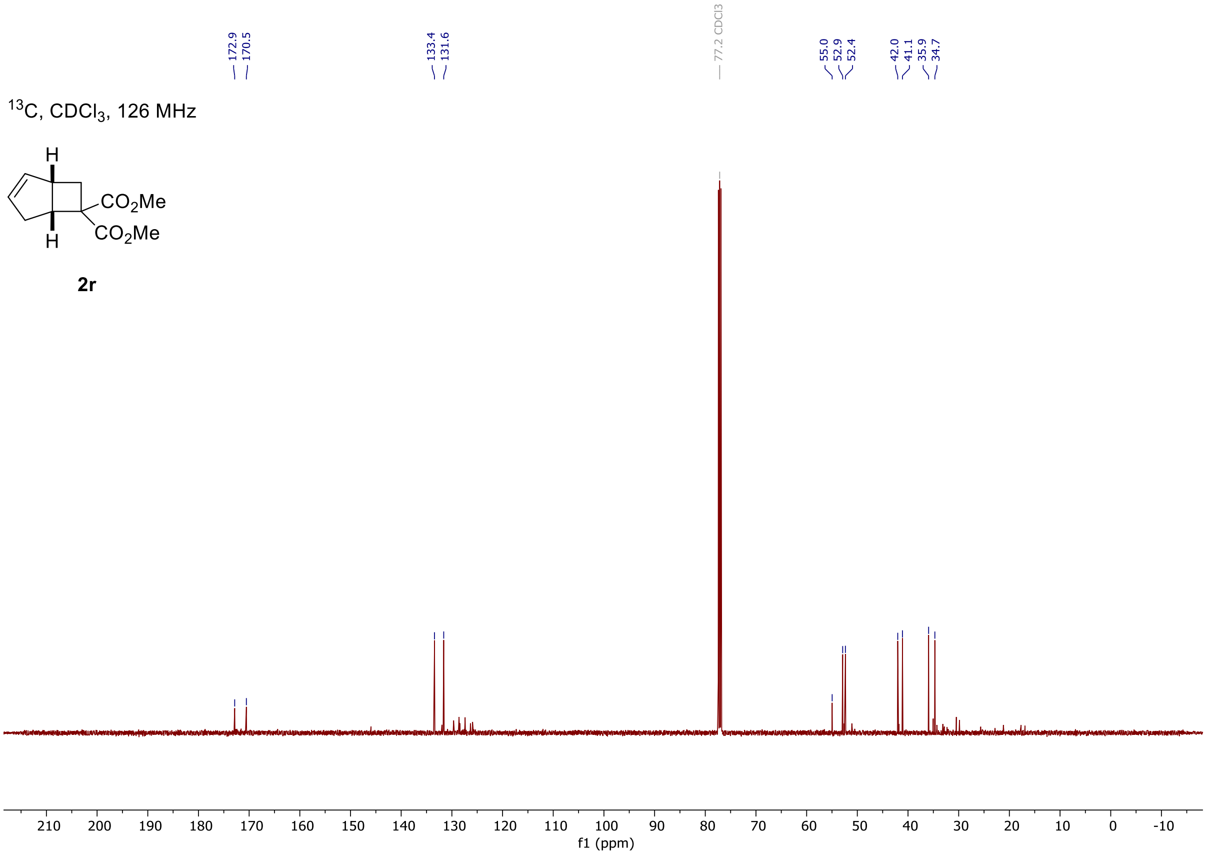
172.9
170.5
133.4
131.6
77.2 CDCl3
55.0
52.9
52.4
42.0
41.1
35.9
34.7
13C, CDCl3, 126 MHz
H
CO2Me
CO2Me
H
2r
f1 (ppm)

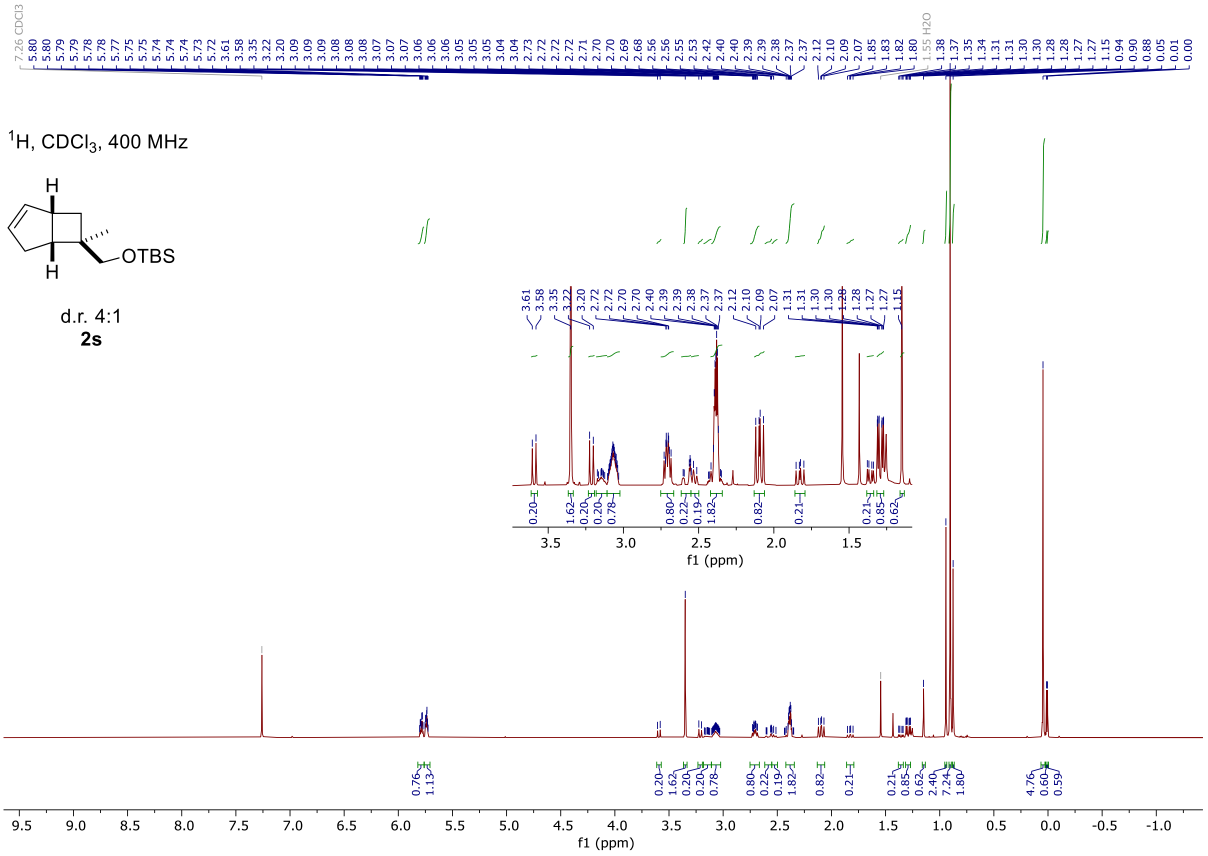
^{1}H, CDCl$_3$, 400 MHz
OTBS
d.r. 4:1
2s
f1 (ppm)

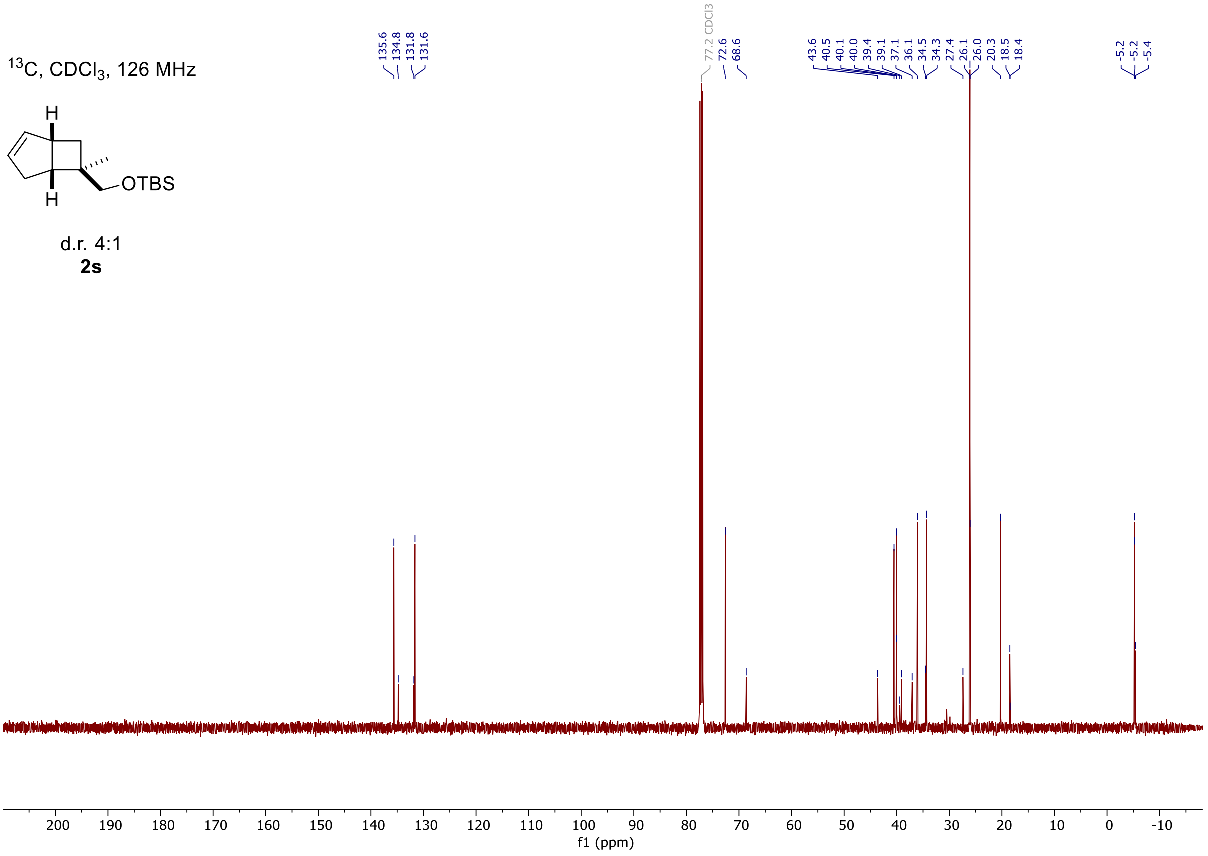
^{13}C, CDCl$_3$, 126 MHz
OTBS
d.r. 4:1
2s
f1 (ppm)

NOESY of **2s** (zoomed in).

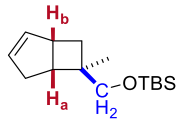

2s

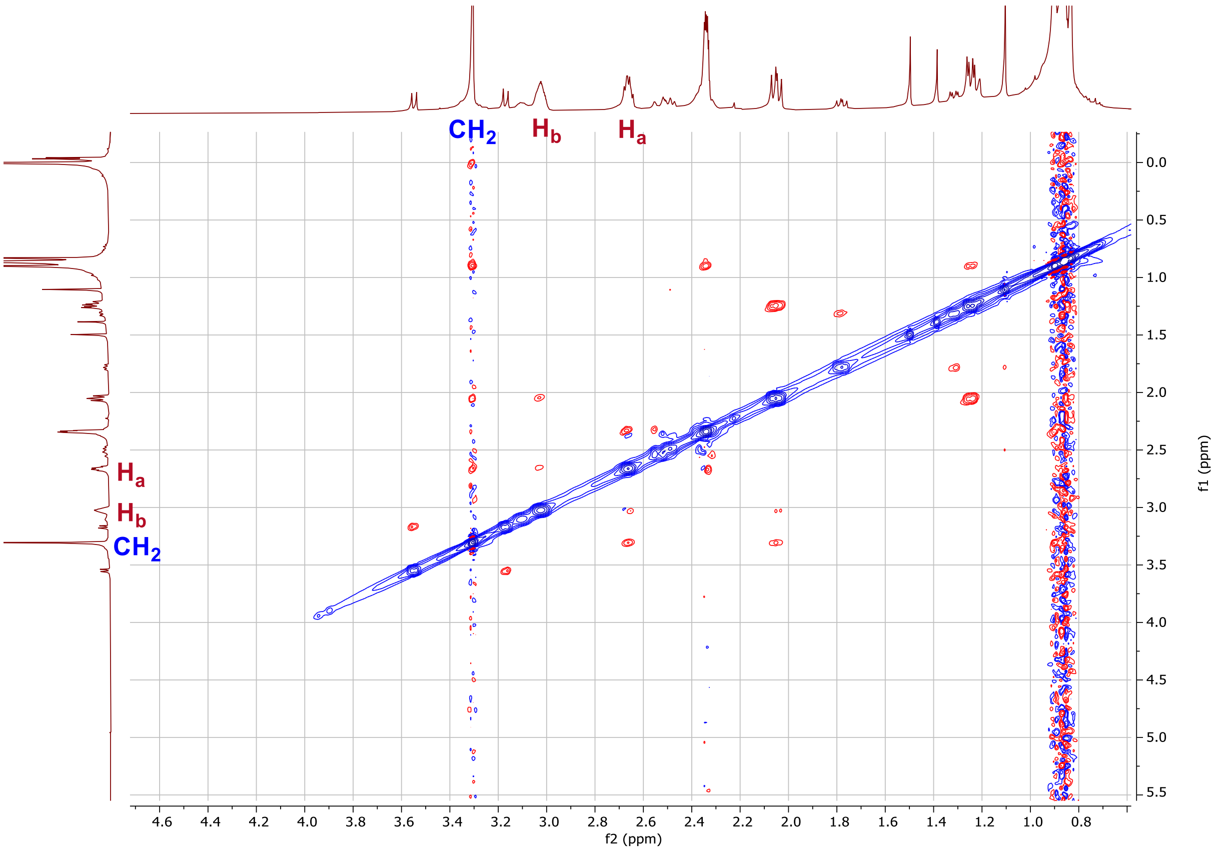

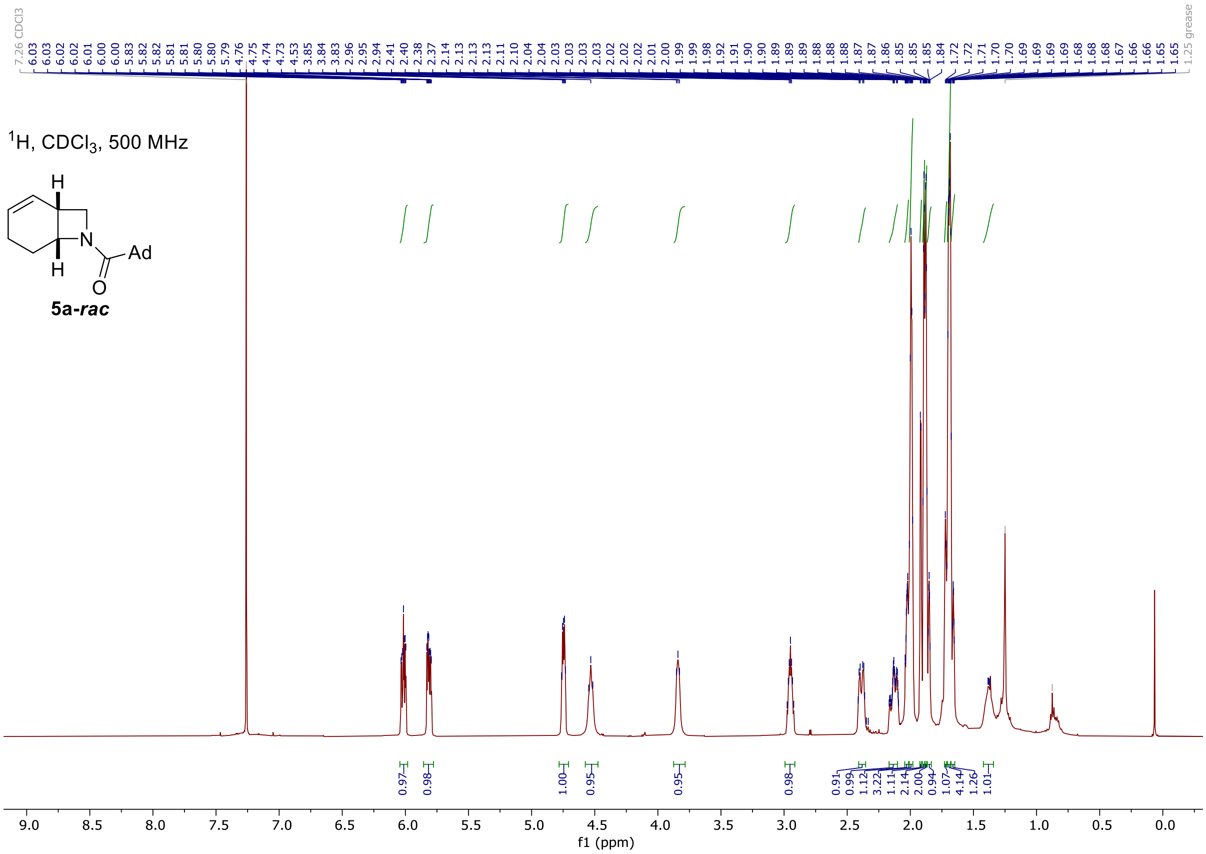

7.26 CDCl3
1.25 grease
^{1}H, $CDCl_3$, 500 MHz
H
N
Ad
H
O
5a-*rac*
0.97
0.98
1.00
0.95
0.95
0.98
0.91
0.99
1.12
3.22
1.11
2.14
2.00
0.94
1.07
4.14
1.26
1.01
9.0
8.5
8.0
7.5
7.0
6.5
6.0
5.5
5.0
4.5
4.0
3.5
3.0
2.5
2.0
1.5
1.0
0.5
0.0
f1 (ppm)

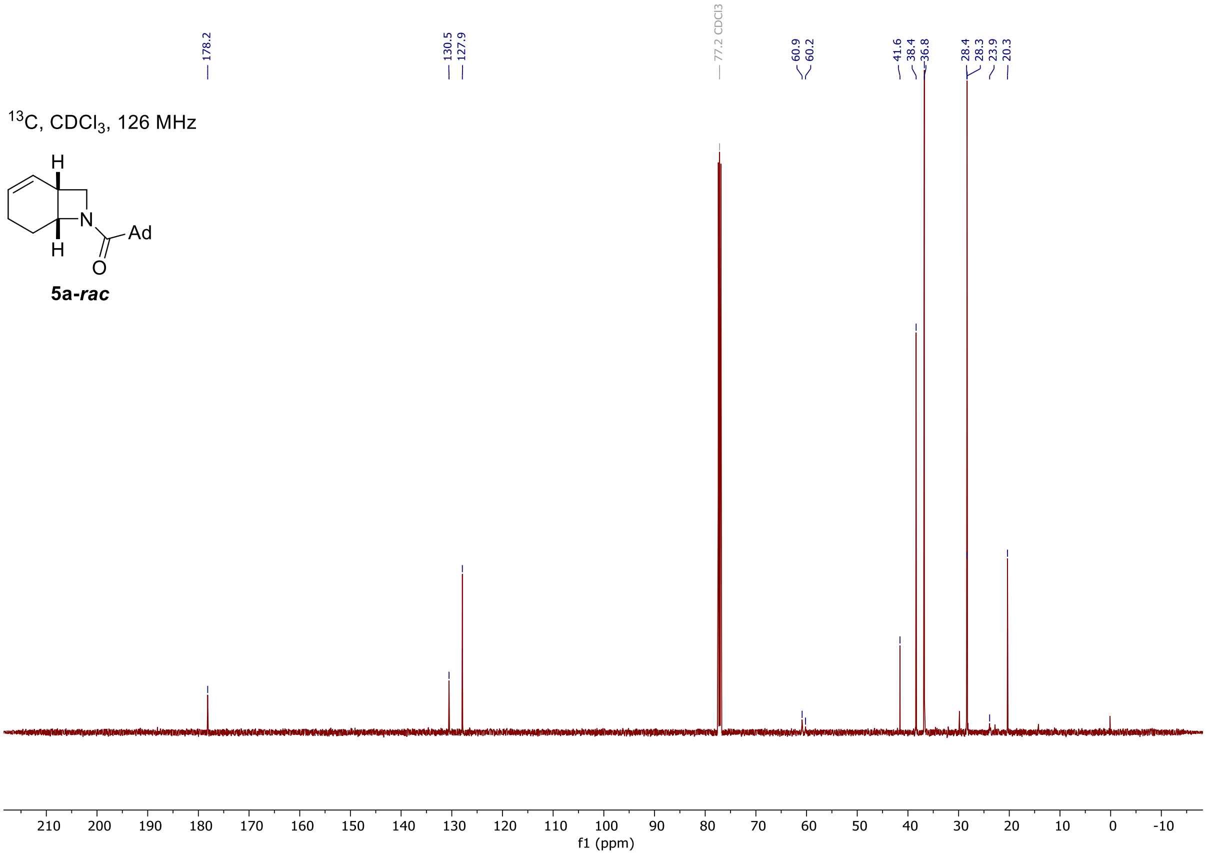

178.2
130.5
127.9
77.2 CDCl3
60.9
60.2
41.6
38.4
36.8
28.4
28.3
23.9
20.3
^{13}C, $CDCl_3$, 126 MHz
H
N
Ad
H
O
5a-*rac*
210
200
190
180
170
160
150
140
130
120
110
100
90
80
70
60
50
40
30
20
10
0
-10
f1 (ppm)

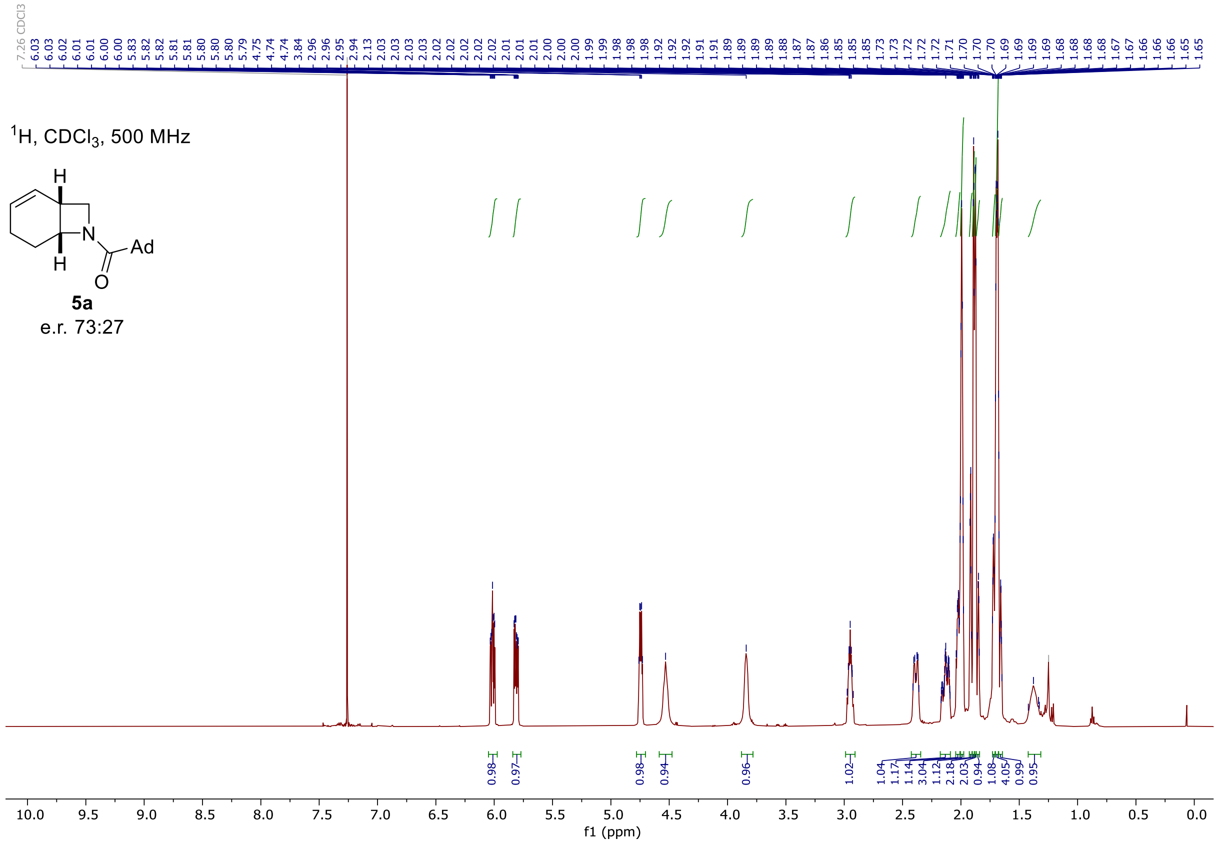

7.26 CDCl3
6.03 6.03 6.02 6.01 6.01 6.00 6.00 5.83 5.82 5.82 5.81 5.81 5.80 5.80 5.80 5.79 4.75 4.74 4.74 3.84 2.96 2.96 2.95 2.94 2.13 2.03 2.03 2.03 2.03 2.02 2.02 2.02 2.02 2.01 2.01 2.01 2.00 2.00 1.99 1.99 1.98 1.98 1.98 1.92 1.92 1.92 1.91 1.91 1.89 1.89 1.89 1.88 1.87 1.87 1.86 1.85 1.85 1.85 1.73 1.73 1.72 1.72 1.72 1.71 1.70 1.70 1.70 1.69 1.69 1.69 1.69 1.68 1.68 1.68 1.67 1.66 1.66 1.66 1.65 1.65
^{1}H, $CDCl_3$, 500 MHz
H
N
Ad
H
O
5a
e.r. 73:27
0.98 0.97 0.98 0.94 0.96 1.02 1.04 1.17 1.14 3.04 1.12 2.18 2.03 0.94 1.08 4.05 0.99 0.95
10.0 9.5 9.0 8.5 8.0 7.5 7.0 6.5 6.0 5.5 5.0 4.5 4.0 3.5 3.0 2.5 2.0 1.5 1.0 0.5 0.0
f1 (ppm)

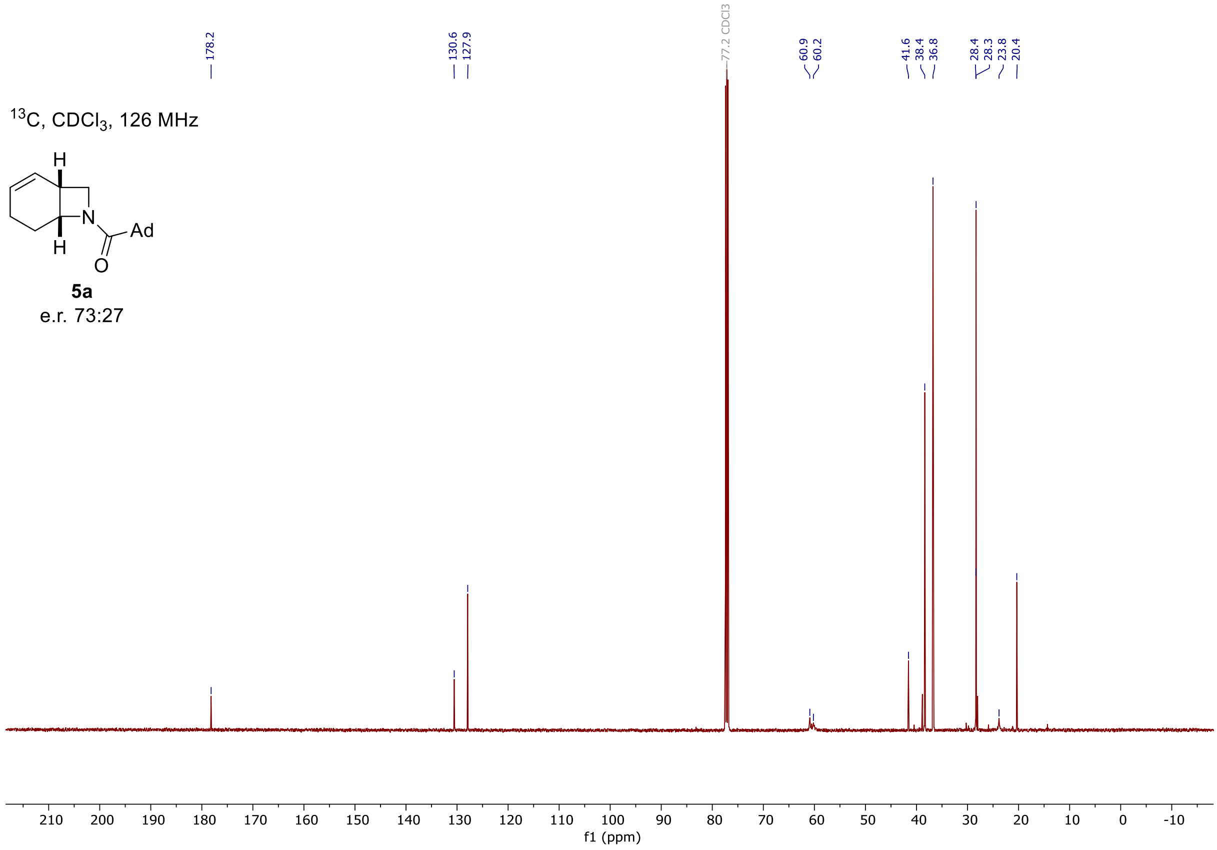

178.2
130.6
127.9
77.2 CDCl3
60.9
60.2
41.6
38.4
36.8
28.4
28.3
23.8
20.4
^{13}C, $CDCl_3$, 126 MHz
H
N
Ad
H
O
5a
e.r. 73:27
210 200 190 180 170 160 150 140 130 120 110 100 90 80 70 60 50 40 30 20 10 0 -10
f1 (ppm)

^1H, $CDCl_3$, 500 MHz

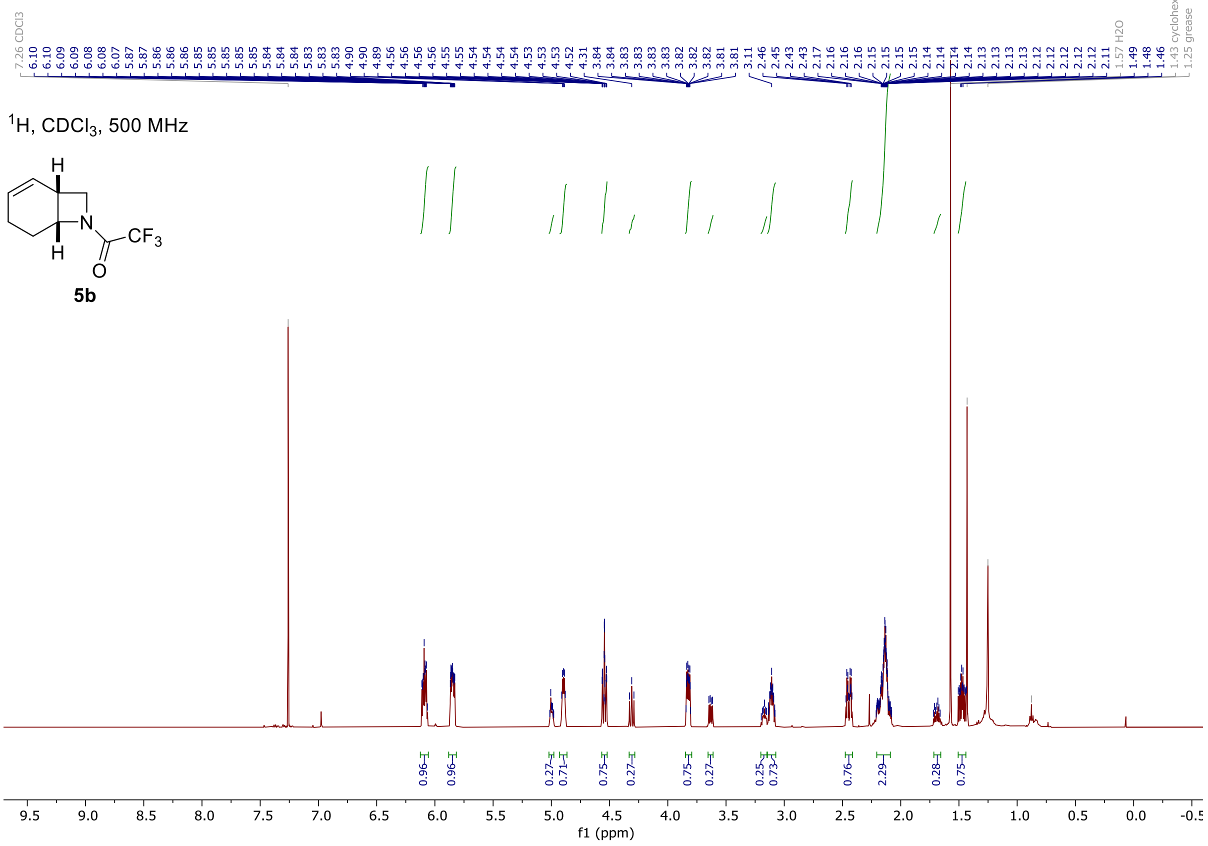

^{13}C, $CDCl_3$, 126 MHz

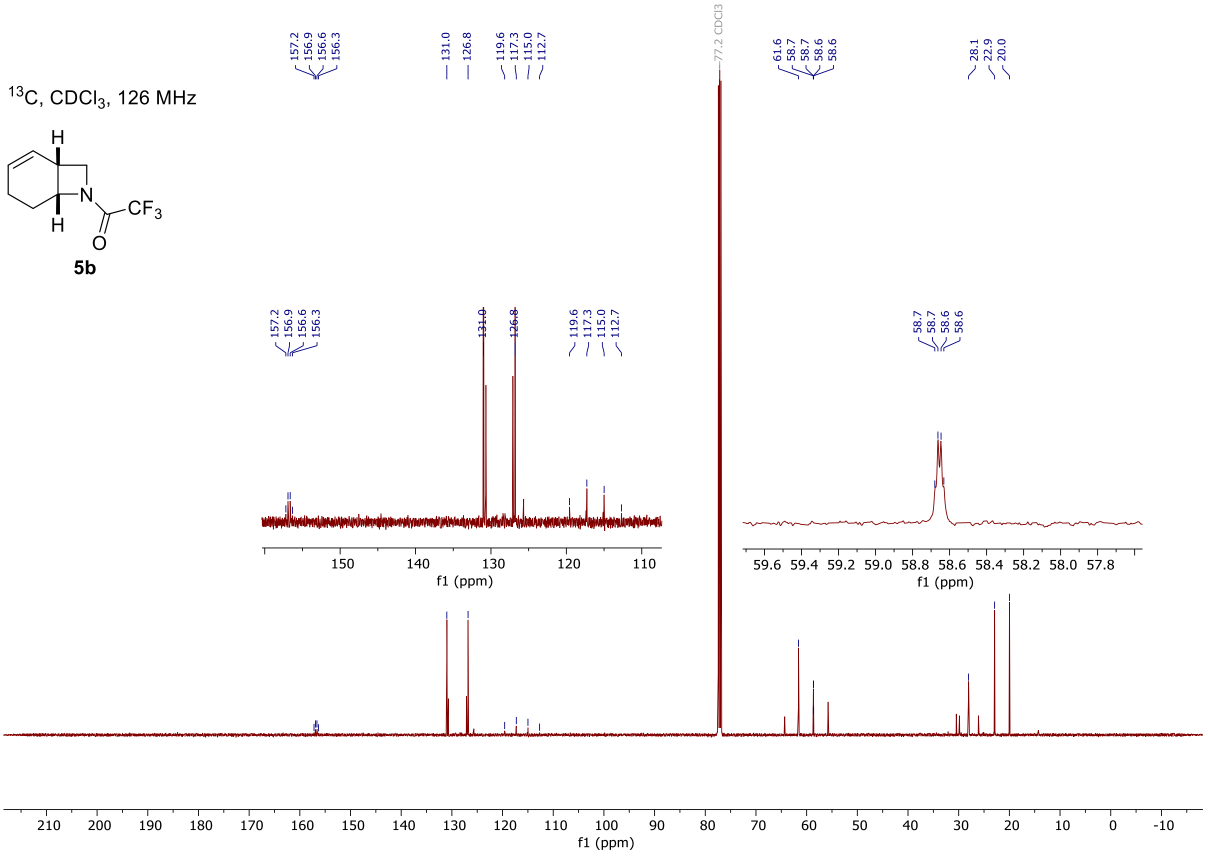

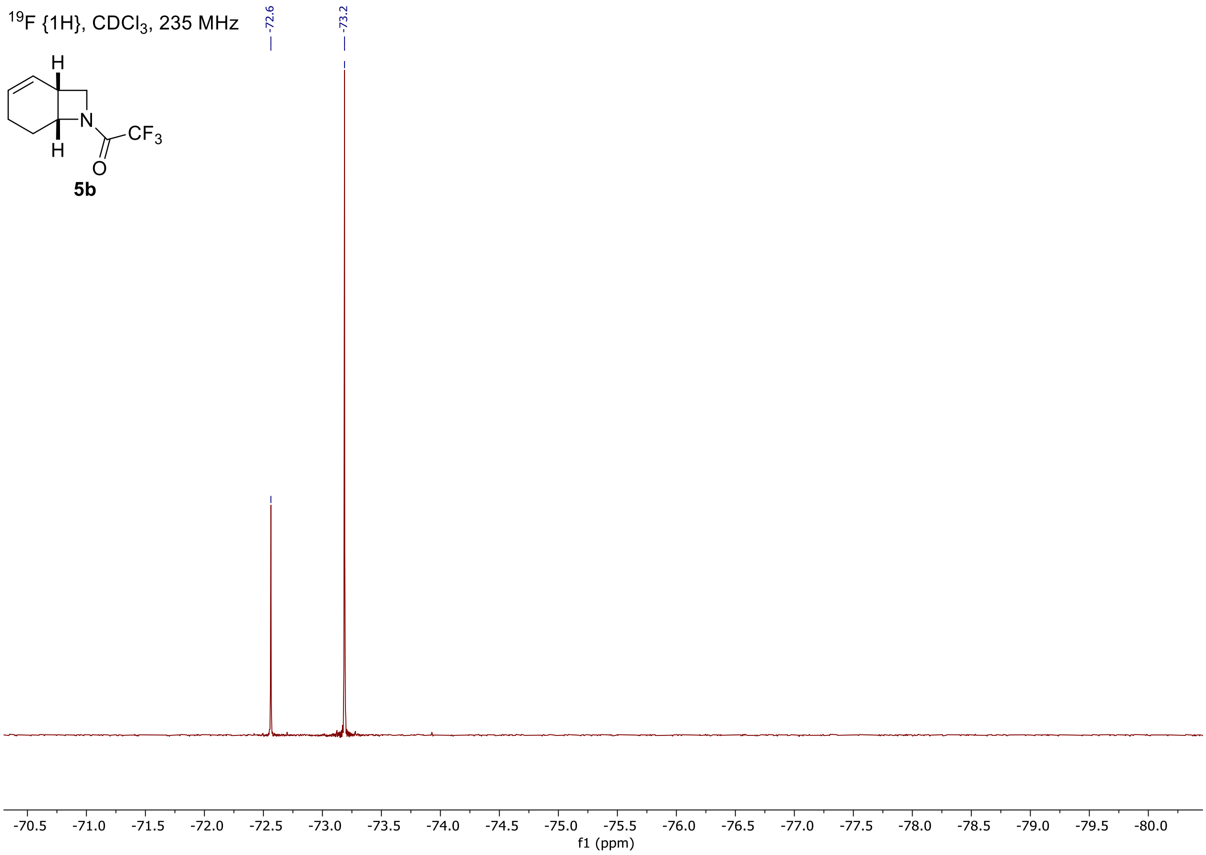

^{19}F {1H}, $CDCl_3$, 235 MHz
-72.6
-73.2
H
N
CF3
H
O
5b
-70.5
-71.0
-71.5
-72.0
-72.5
-73.0
-73.5
-74.0
-74.5
-75.0
-75.5
-76.0
-76.5
-77.0
-77.5
-78.0
-78.5
-79.0
-79.5
-80.0
f1 (ppm)

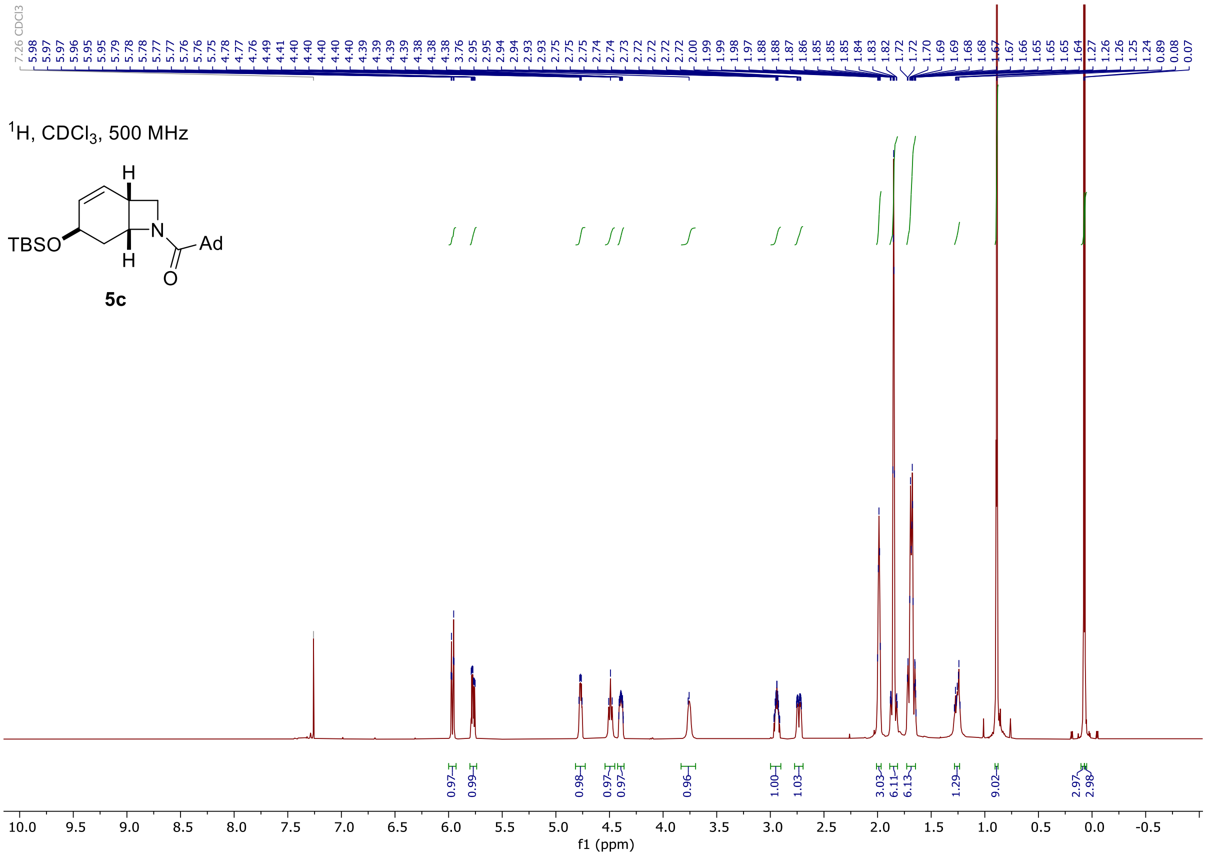

^{1}H, CDCl$_3$, 500 MHz
TBSO
H
N
Ad
O
f1 (ppm)

5c

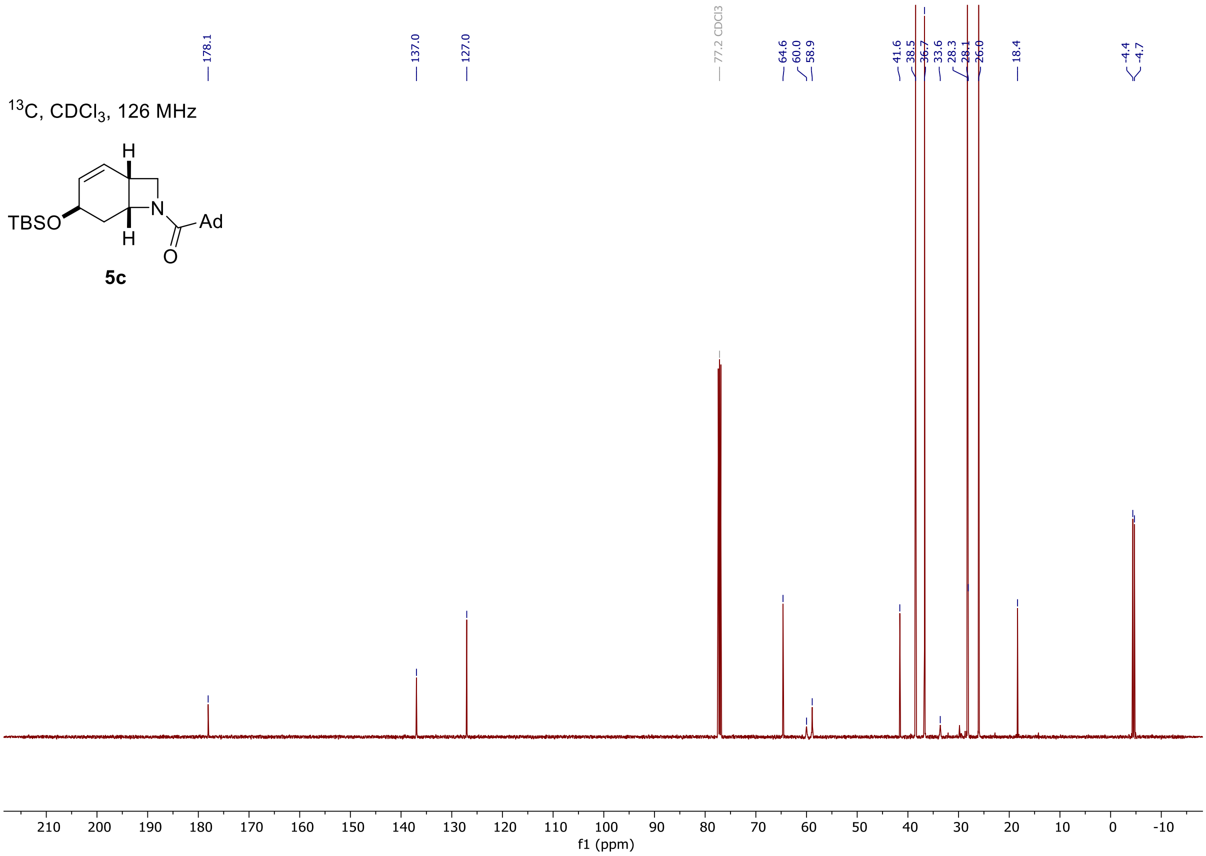

^{13}C, CDCl$_3$, 126 MHz
TBSO
H
N
Ad
O
f1 (ppm)

5c

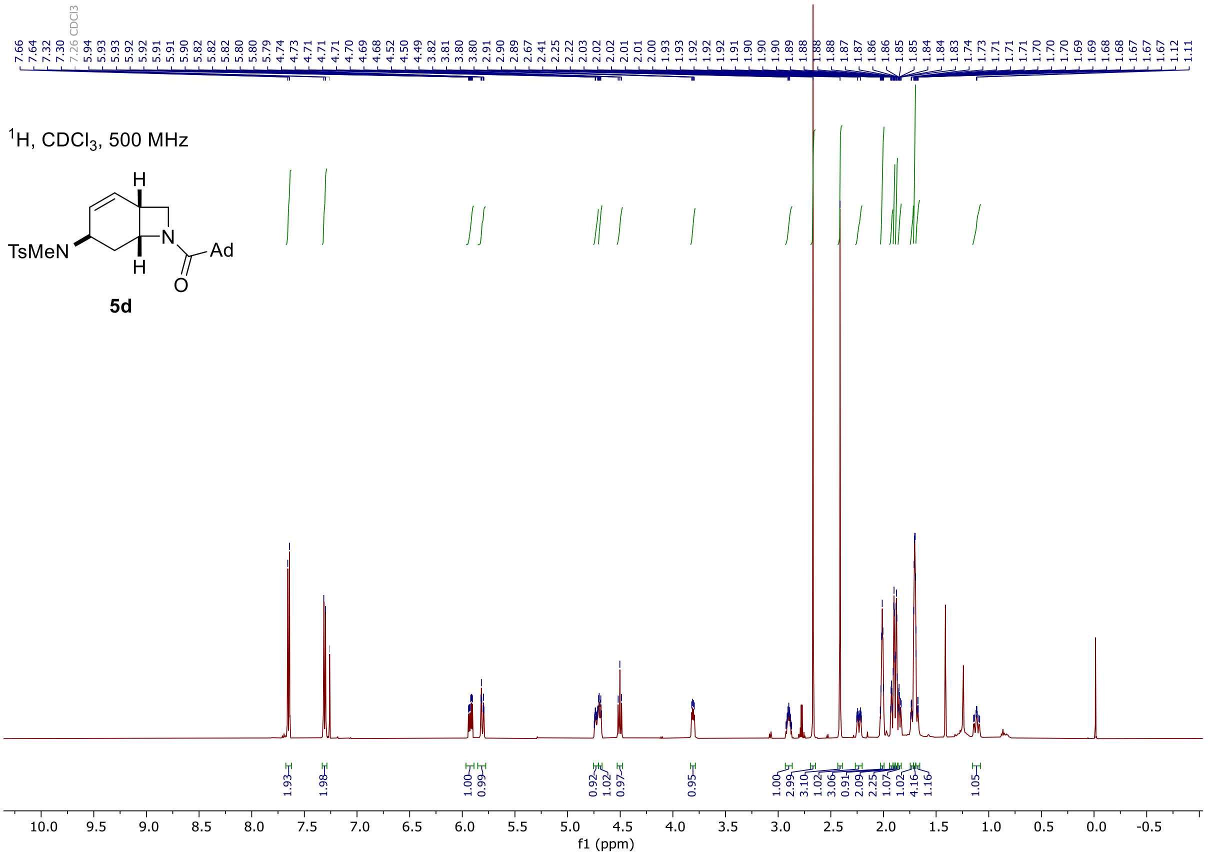
7.66
7.64
7.32
7.30
7.26 CDCl3
1H, CDCl3, 500 MHz
TsMeN
Ad
5d
1.93
1.98
1.00
0.99
0.92
1.02
0.97
0.95
1.00
2.99
3.10
1.02
3.06
0.91
2.09
2.25
1.07
1.02
4.16
1.16
1.05
f1 (ppm)

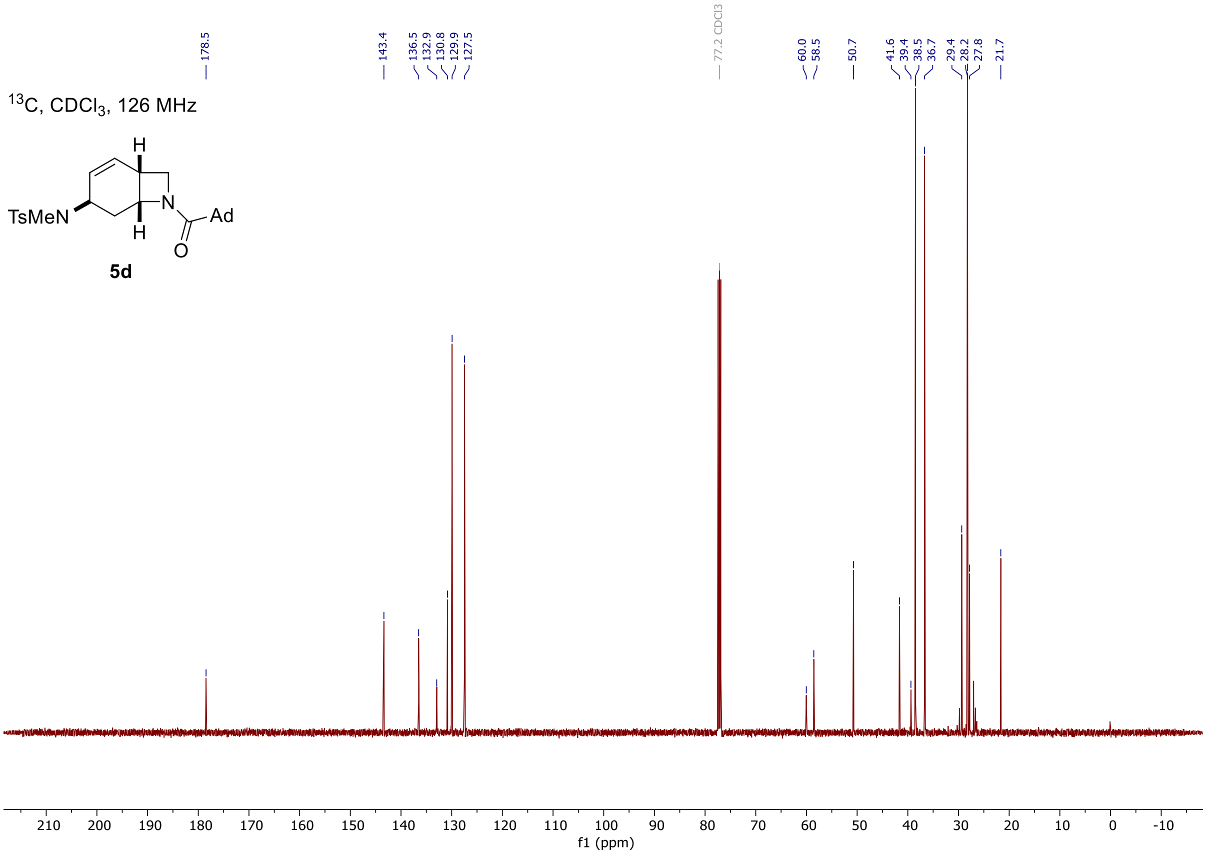
178.5
143.4
136.5
132.9
130.8
129.9
127.5
77.2 CDCl3
60.0
58.5
50.7
41.6
39.4
38.5
36.7
29.4
28.2
27.8
21.7
13C, CDCl3, 126 MHz
TsMeN
Ad
5d
f1 (ppm)

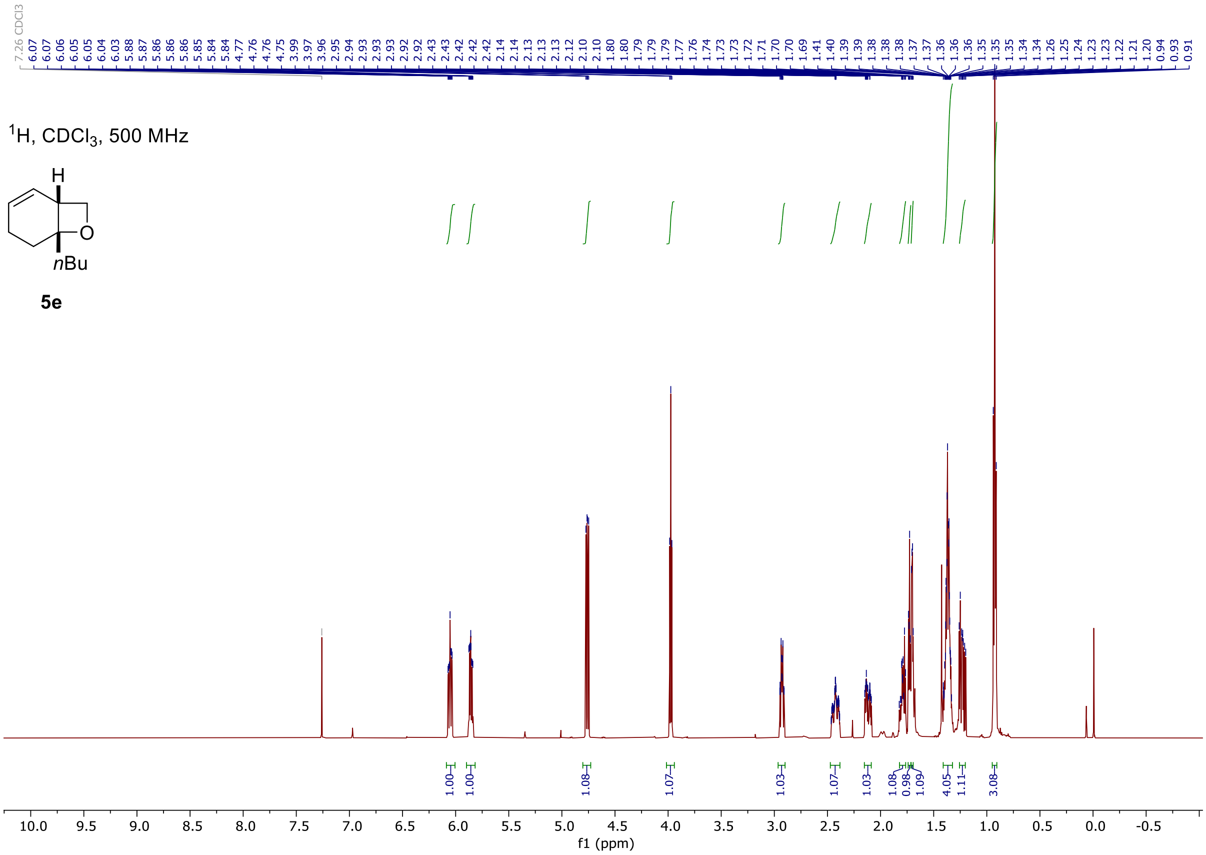

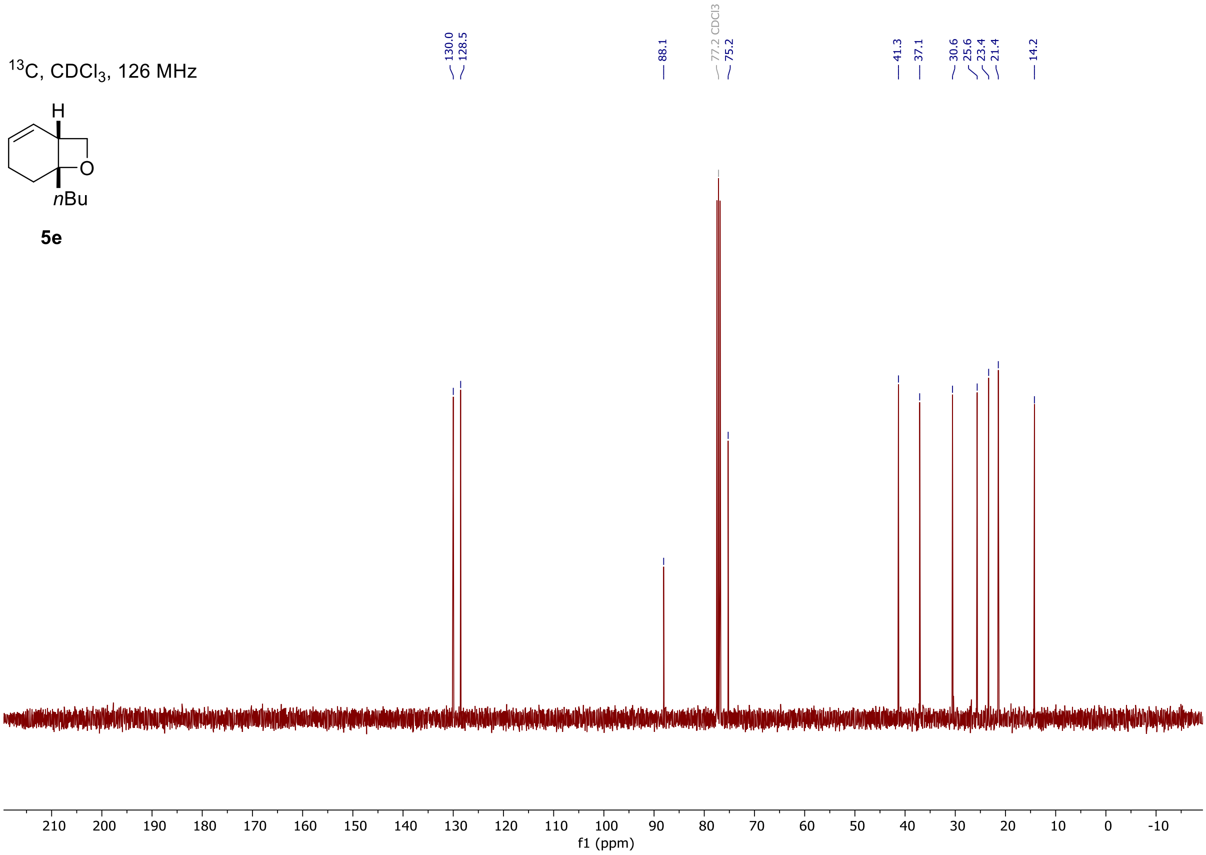

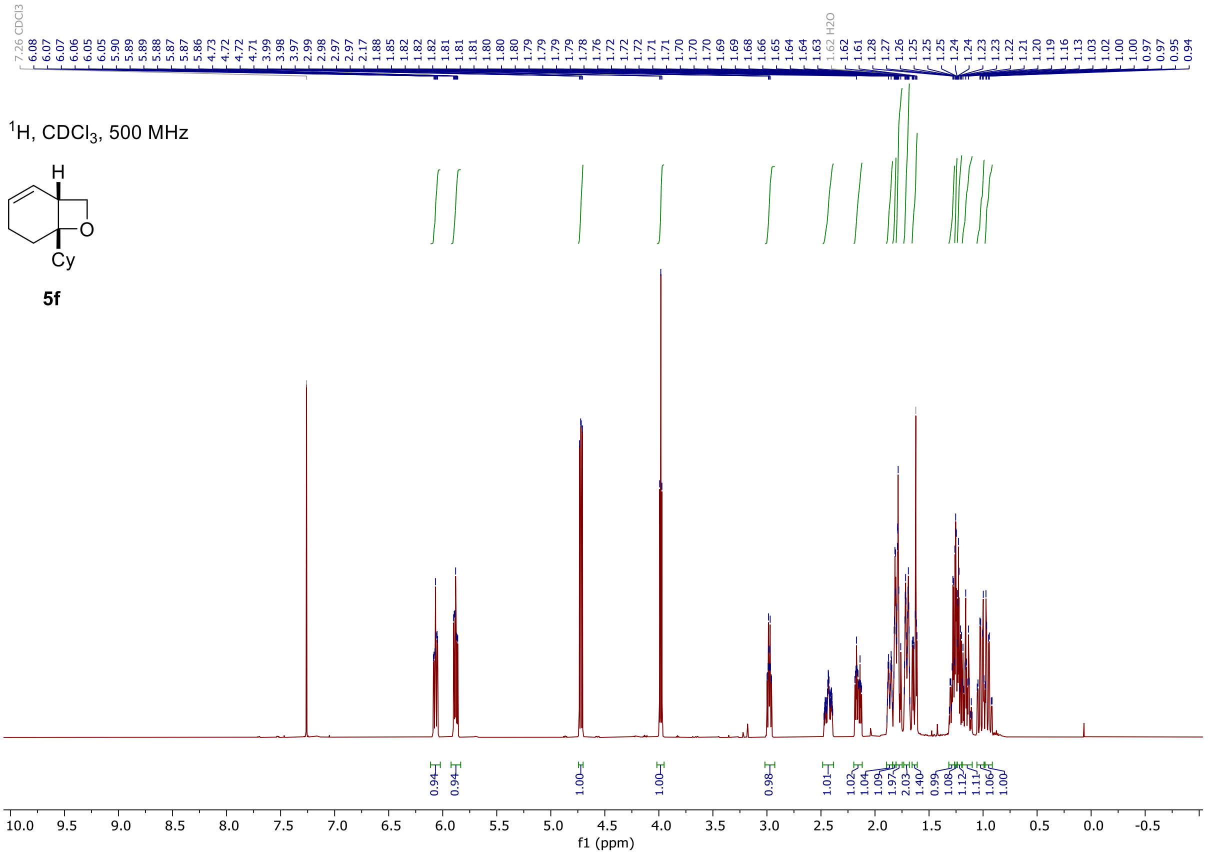

1H, CDCl3, 500 MHz
H
O
Cy
5f
7.26 CDCl3
1.62 H2O
f1 (ppm)

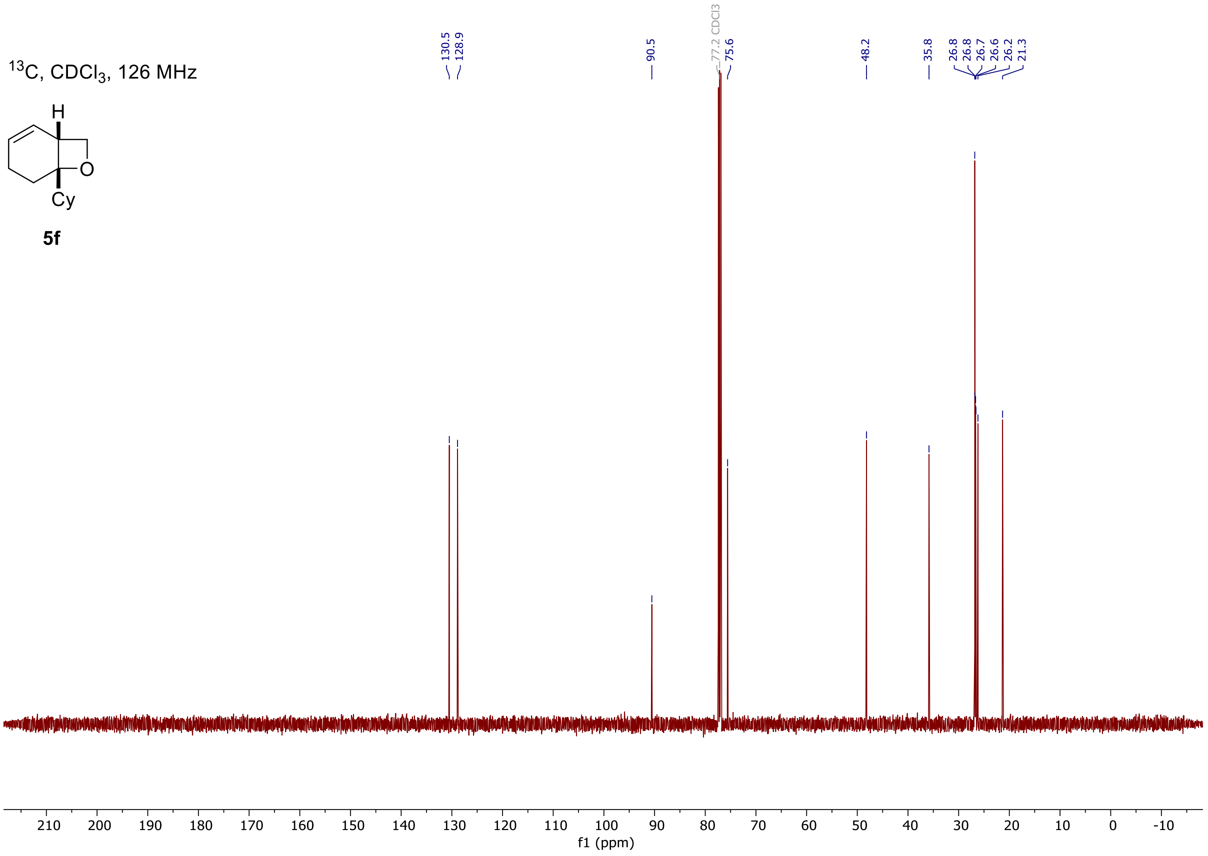

13C, CDCl3, 126 MHz
H
O
Cy
5f
130.5
128.9
90.5
77.2 CDCl3
75.6
48.2
35.8
26.8
26.8
26.7
26.6
26.2
21.3
f1 (ppm)

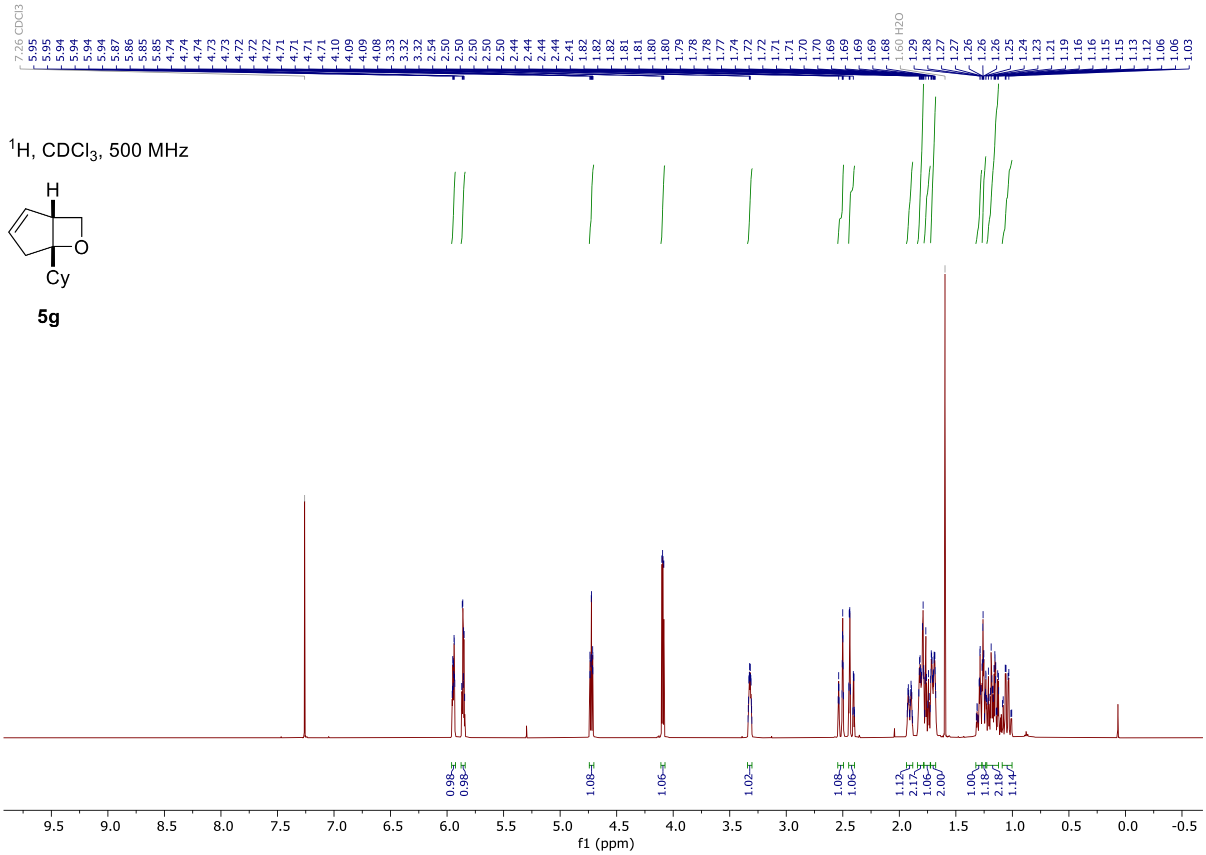
7.26 CDCl3
5.95 5.95 5.94 5.94 5.94 5.94 5.87 5.86 5.85 5.85 4.74 4.74 4.74 4.73 4.73 4.72 4.72 4.72 4.71 4.71 4.71 4.10 4.09 4.09 4.08 3.33 3.32 3.32 2.54 2.50 2.50 2.50 2.50 2.50 2.44 2.44 2.44 2.44 2.41 1.82 1.82 1.82 1.81 1.81 1.80 1.80 1.79 1.78 1.78 1.77 1.74 1.72 1.72 1.71 1.71 1.70 1.70 1.69 1.69 1.69 1.69 1.68
1.60 H2O
1.29 1.28 1.27 1.27 1.26 1.26 1.26 1.25 1.24 1.23 1.21 1.19 1.16 1.16 1.15 1.13 1.12 1.06 1.03
1H, CDCl3, 500 MHz
H
O
Cy
5g
0.98 0.98 1.08 1.06 1.02 1.08 1.06 1.12 2.17 1.06 2.00 1.00 1.18 2.18 1.14
9.5 9.0 8.5 8.0 7.5 7.0 6.5 6.0 5.5 5.0 4.5 4.0 3.5 3.0 2.5 2.0 1.5 1.0 0.5 0.0 -0.5
f1 (ppm)

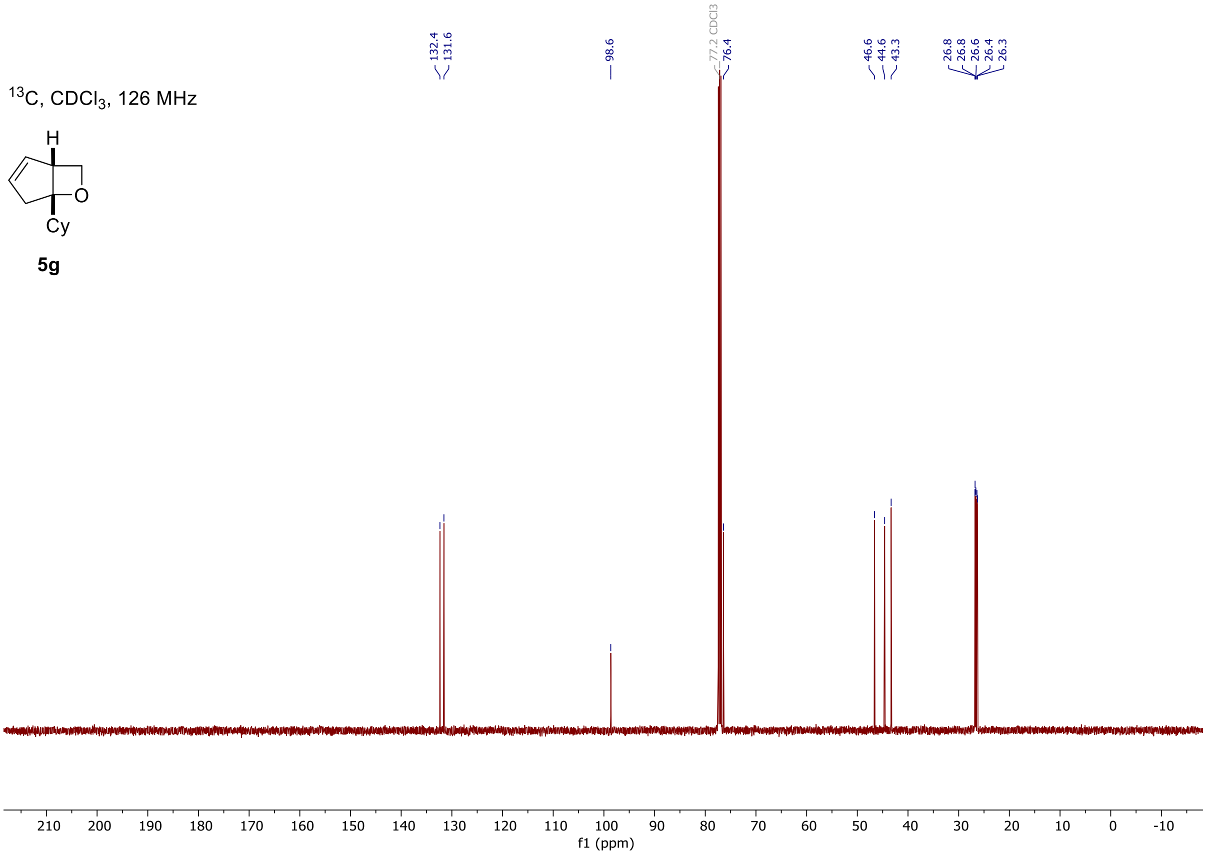
132.4 131.6
98.6
77.2 CDCl3
76.4
46.6 44.6 43.3
26.8 26.8 26.6 26.4 26.3
13C, CDCl3, 126 MHz
H
O
Cy
5g
210 200 190 180 170 160 150 140 130 120 110 100 90 80 70 60 50 40 30 20 10 0 -10
f1 (ppm)
